# The acceptability, safety, and performance of primary cervical screening through self-collected vaginal samples in an urban teaching hospital antenatal clinic setting

**DOI:** 10.1371/journal.pgph.0005149

**Published:** 2025-09-02

**Authors:** Su Pei Khoo, Joanne Johnny Bouniu, Lavanya Sivaram, David Hawkes, Peng Chiong Tan, Marion Saville, Yin Ling Woo

**Affiliations:** 1 Department of Obstetrics and Gynaecology, University of Malaya Medical Centre, Kuala Lumpur, Malaysia; 2 Australian Centre for the Prevention of Cervical Cancer, Carlton, Victoria, Australia; 3 Department of Biochemistry and Pharmacology, University of Melbourne, Parkville, Australia; 4 Department of Pathology, Faculty of Medicine, Universiti Malaya, Kuala Lumpur, Malaysia; 5 Department of Obstetrics and Gynaecology, Faculty of Medicine, Universiti Malaya, Kuala Lumpur, Malaysia; PLOS: Public Library of Science, UNITED STATES OF AMERICA

## Abstract

This study evaluated the feasibility, safety, and acceptability of self-collected vaginal samples for human papillomavirus (HPV) testing among pregnant women in an urban teaching hospital in Malaysia. This cross-sectional study recruited pregnant women aged 30 years and above who attended antenatal care at the University of Malaya Medical Centre. Participants self-collected vaginal samples using FLOQSwabs and completed pre- and post-sampling questionnaires assessing acceptability. Samples were analysed using the Roche cobas 4800 system. HPV-positive participants were referred for postpartum colposcopy. A total of 2,256 eligible pregnant women were invited to participate in the study and 1,603 of them consented to participate, representing 71.1% of uptake. Of the 1,603 participants, 99.6% (1,596) agreed to self-collect, with 98.6% successfully completing the procedure. More than 80% of participants responded positively to acceptability indicators after self-collection procedure, including overall feeling, ease of performance, convenience, and confidence in collecting an accurate sample. The HPV prevalence recorded in the study population was 6.3%, with most infections involving non-HPV16/18 types. No major complications associated with the sampling procedure were reported, and 99.0% of participants expressed willingness to use self-collection for future cervical screening. Self-collection for HPV-based cervical screening during pregnancy is highly acceptable, safe, and feasible, with performance comparable to the general population. Integrating self-collection into antenatal care can enhance cervical cancer screening rates among under-screened populations, contributing to global elimination goals.

## Introduction

Cervical cancer remains a significant global health challenge, with over 80% of new cases, including advanced or metastatic presentations, occurring in low- and middle-income countries (LMICs) [1]. The incidence and mortality rates are disproportionately higher in these regions where limited resources exacerbate health disparities [2]. Tragically, many affected women face financial hardship, and rising deaths leave children orphaned, with long-term social and economic impacts [3]. These outcomes highlight the urgency of prioritising cervical cancer, mainly because practical tools for the elimination of upstream cervical diseases exist. More recently, however, there has been a growing shift in focus — not just toward the long-term goal of elimination, but toward immediate, individual-level prevention efforts. For every dollar invested by 2050, an estimated economic return of USD3.20 is expected, rising to USD26.00 when societal benefits are included [4].

The World Health Organization (WHO) has developed a global strategy to eliminate cervical cancer as a public health issue by 2030, centred on cost-effective, evidence-based interventions. This strategy includes vaccinating 90% of girls against human papillomavirus (HPV) by age 15, screening 70% of eligible women with high-performance tests such as HPV test and treating 90% of those diagnosed with cervical disease [4]. Serrano and colleagues identified 35% of 139 countries with government screening recommendations that promote the use of primary HPV-based screening [5]. In this context, self-collection for HPV-based cervical screening (hereafter referred to as “self-collection”) has emerged as a transformative approach, its utilisation providing a key opportunity for increasing coverage in LMICs, where infrastructure and specialised workforce are limited [5].

Pregnancy presents a critical window for addressing cervical screening gaps, especially in LMICs, where antenatal care is often the primary point of contact with healthcare systems for women of reproductive age [6]. The WHO’s updated antenatal care model emphasizes integrated care delivery, recommending at least eight contacts to enhance women’s health experiences [7]. The antenatal and postnatal periods offer ideal opportunities to incorporate cervical screening for women who may have missed routine screening [8]. Studies suggest that screening during pregnancy or postpartum could avert cervical cancer cases [9,10] and align with guidelines such as those in the Australian National Cervical Screening Program, which advocate screening during antenatal visits when due or overdue [8]. While physiological changes in pregnancy may affect the accuracy of visual inspection with acetic acid [11], HPV test remains effective and reliable as a primary screening tool [12].

In this study, we assessed the feasibility, safety, and acceptability of self-collected vaginal swabs among pregnant women in a Malaysian hospital setting. To our knowledge, this is the first study in Malaysia evaluating self-collection in an antenatal population, aligning with the WHO’s strategy to accelerate the elimination of cervical cancer which includes screening 70% of women by the age of 35 with a high-performance (e.g., HPV nucleic acid amplification) test by 2030 [4].

## Materials and methods

### Ethics statement

This study obtained ethical approval from the Universiti Malaya Medical Centre-Medical Research Ethics Committee (MREC ID 2021810–10466). Written informed consent was obtained from all participants.

### Design and setting

This cross-sectional study was conducted among pregnant women seeking antenatal care at the University of Malaya Medical Centre (UMMC), a government-led tertiary teaching hospital, recruited between September 2021 to October 2023. The study follows the Strengthening the Reporting of Observational Studies in Epidemiology (STROBE) statement checklist [13].

### Study participants

The participants were recruited from pregnant individuals attending the antenatal clinic at UMMC. To be eligible for the study, participants were at least 30 years old, pregnant for a minimum of 15 weeks, and a Malaysian citizen. Eligible women who were willing to self-collect a vaginal sample for HPV-based cervical screening were recruited for the study. The exclusion criteria included bleeding or premature rupture of membrane during the current pregnancy, induction of labour, a history of cervical cancer or precancerous lesions, and a prior history of HPV-based cervical screening within the past five years. In particular, study participants were counselled about the implications of HPV-positive results and were given options of immediate or postnatal follow-up. Furthermore, participants were provided with a dedicated 24/7 study mobile number to contact if there were any concerns.

### Sample collection and analysis

All participants self-collected a vaginal sample using a FLOQSwab (552.80, Copan, Brescia, Italy) [14] enclosed in a plastic packaging tube. Verbal and diagrammatic instructions were provided by trained research staff before the self-collection procedure. Participants were instructed to gently insert the swab, not beyond the red mark on the shaft (80 mm from the tip), and to rotate the swab gently for at least 10 times before returning it to its packaging tube. They could assume any of the following positions: standing with one leg raised, sitting or squatting. Participants who were unable to successfully complete the self-collection were offered the option of having a clinician-collected vaginal sampling with the FLOQSwab. Those who encountered complications during or after self-collection received immediate care and reassurance from the specialist in charge.

All swabs were kept at room temperature for a maximum of two weeks before being analysed in batches, as per the laboratory standard operating procedure. Swabs were broken at the designated breaking point, placed into 5 ml of PreservCyt Solution (Hologic, Bedford, USA), and swirled vigorously for 40 seconds to elute the material from the swab. These samples were then tested using the Roche cobas 4800 HPV test (Roche Molecular Systems, Pleasanton, USA) as per the method validated by Saville and colleagues [15]. The cobas 4800 HPV test is processed using the automated Roche cobas 4800 system and uses polymerase chain reaction to produce a partial genotyping results for HPV16, HPV18, and a pooled ‘Other’ HPV result (HPV31,33, 35, 39. 45, 51, 52, 56, 58, 59, 66, and 68) or ‘non-HPV16/18’.

Notably, the FLOQSwab is a validated device using manufacturer-specific methods for the Roche cobas and cobas 4800, and the BD Onclarity HPV tests but its use in pregnancy is not yet approved.

### Data collection

Before sample collection, all participants were asked to complete a set of questionnaires which included socio-demographic data, cervical screening and HPV vaccination history, reproductive history, and pre-sampling expectations. Immediately after sample collection, the participants were asked if they managed to successfully self-collect vaginal samples, and for their post-sampling feedback. Self-collection is considered successful when the participant reported to have collected her sample by inserting the FLOQSwab into her vagina and rotating it for at least 10 times. However, it was deemed unsuccessful if the participants failed to insert and rotate the swabs to collect samples. Participants who tested positive for HPV were given a colposcopy appointment after delivery if they chose not to have immediate follow-up. The colposcopy appointment attendances and histological diagnoses from biopsy samples were recorded.

### Statistical analysis

Sample size calculation was based on an assumption of a screening uptake of 50%, slightly lower than the uptake rate reported in the general population in a similar setting [16]. Using Open Epi software [17], the calculated sample size was 1,535 at a desired level of confidence interval at 95% and a margin of error of 2.5%.

Statistical analyses were conducted using STATA version 18 (Stata Corp, College Station, USA). The acceptability of the self-collection was evaluated through a 5-point Likert scale [18,19], assessing six indicators: participants’ overall experience, ease of the test, convenience, degree of embarrassment, level of discomfort/pain, and confidence in performing the self-collection (Table 1). A score point of 4 or higher was considered a positive response, indicating acceptance of self-collection procedure. The proportion of participants achieving score of at least 4 was reported as a percentage of total responses with the corresponding 95% confidence interval. The statistical difference between pre-sampling expectations and post-sampling feedback were analysed using McNemar’s test. HPV prevalence estimates and invalid screening outcomes were described using percentages with a 95% confidence interval.

**Table 1:** Indices of the five-item Likert scale for each of the acceptability indicators.

Likert scale	1	2	3	4	5
Overall feeling	Very bad	Bad	Neutral	Good	Very good
Ease of performance	Very hard	Hard	Neutral	Easy	Very easy
Convenience	Very inconvenient	Inconvenient	Neutral	Convenient	Very convenient
Embarrassment	Very embarrassed	Embarrassed	Neutral	Not embarrassed	Not embarrassed at all
Discomfort/ pain	Severe discomfort/ pain	Some discomfort/ pain	Neutral	No discomfort/ pain	No discomfort/ pain at all
Confidence	Not at all confident	Not confident	Neutral	Confident	Very confident

## Results

### Characteristics of study population

**Fig 1:**
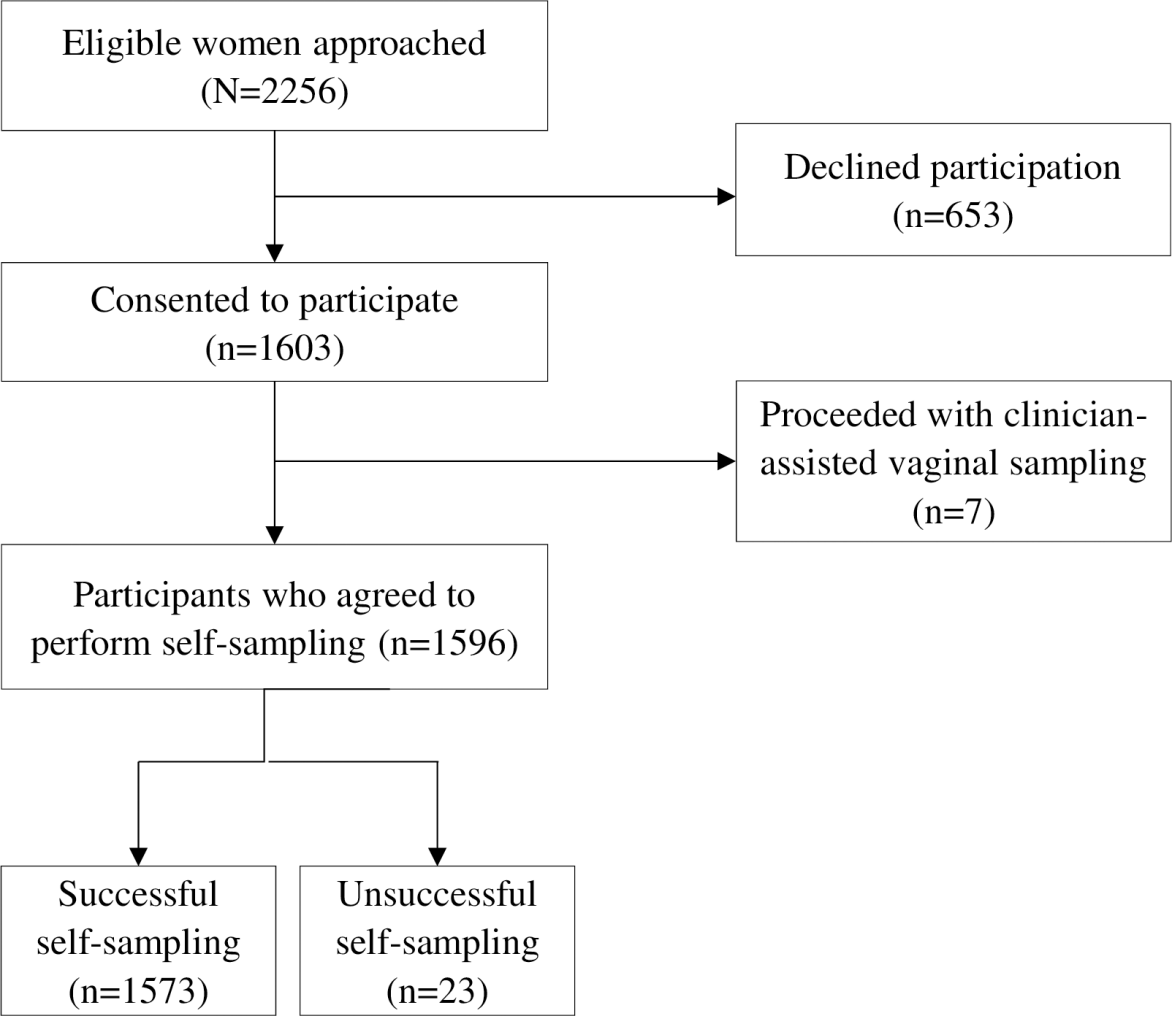
Study recruitment flow diagram.

Two thousand two hundred and fifty-six eligible pregnant women were invited to participate in the study and 71.1% (1603/2256) gave their informed consent to participate. The recruitment flow diagram of study participants is shown in Fig 1. Reasons for declining to participate in the study are detailed in Table 2. Of the 1,603 recruited participants, 1,596 pregnant women (99.6%) were willing to self-collect a sample, while the remaining seven (0.4%) opted for a clinician-collected vaginal sample for HPV-based screening. 1,573 participants (98.6%) successfully completed self-collection.

**Table 2:** Reasons for rejecting HPV-based cervical screening during pregnancy.

Reasons*	n = 653	%
Not interested	270	39.2
Too much time required/inconvenient	79	11.5
Do not want to perform a cervical screening during pregnancy	308	44.7
Fear of pain	16	2.3
Foetal concerns (such as history of threatened miscarriage, amniotic fluid disorder, abdominal pain, etc.)	5	0.7
Others (such as worried about confidentiality, wants to ask husband first, etc.)	11	1.6

*More than one reasons were indicated.

Footnote: HPV, human papillomavirus

Demographic characteristics of the study participants are shown in Table 3. The mean age of the participants was 34.4 years, with 54.2% aged 30–34 years and another 37.7% aged 35–39 years. 64.5% identified as Malay, 20.8% as Chinese and 13.5% as Indian. In terms of education, a significant majority (72.6%) attended tertiary education. Most participants were employed full-time (76.9%), with over half (51.6%) earning between RM5,000 (USD1,125) and RM10,000 (USD2,250) monthly. About half of the study participants (49.8%) reported a previous Pap smear (cervical sample collected for cytology-based screening). At the time of recruitment into the study, 60.6% were in their third trimester (28 + weeks), and 77.1% reported engaging in vaginal intercourse during their current pregnancy. Additionally, 25.8% reported having had a history of miscarriage.

**Table 3:** Demographic characteristics of study cohort.

Variables	n = 1596	%
**Age in years, median (range)**	34.4 (30–47)	
**Age group**		
30–34 years	865	54.2
35–39 years	602	37.7
40–44 years	125	7.8
45 years and above	4	0.3
**Ethnicity**		
Malay	1029	64.5
Chinese	332	20.8
Indian	215	13.5
Other	20	1.2
**Educational level**		
Primary	4	0.2
Secondary	289	18.1
Tertiary	1158	72.6
Postgraduate	145	9.1
**Employment status**		
Full-time	1227	76.9
Part-time	20	1.2
Temporarily unemployed/maternity leave/students	35	2.2
Work from home/housewives	306	19.2
Self-employed	8	0.5
Household income (RM/month) *		
< 1,000	10	0.6
1,001–5,000	446	28.0
5,001–10,000	823	51.6
> 10,000	316	19.8
**Have had a pap smear done previously**		
No	794	49.8
Yes	795	49.8
Don’t know	7	0.4
**History of HPV vaccination**		
Yes	193	12.1
No	1403	87.9
**Pregnancy stage (weeks)**		
15–27	629	39.4
28–40	966	60.5
41 and above	1	0.1
**Vaginal sex during current pregnancy**		
Yes	1230	77.1
No	364	22.8
Decline to respond	2	0.1
**History of miscarriage**		
Yes	412	25.8
No	1184	74.2

*Missing data were excluded (n=1).

Note: RM1,000 is equivalent to ~USD225.

Footnote: RM, ringgit Malaysia; HPV, human papillomavirus.

### Safety and acceptability of self-collection for HPV-based cervical screening

Among 1,596 participants who performed self-collection for HPV-based cervical screening, more than half of the participants (66.4%) reacted positively to the idea of performing a self-collection procedure, although nearly 60% of participants expected the procedure to cause some degree of discomfort or pain prior to the procedure (refer to Table 4). After performing said self-collection, less than 33% of participants thought self-collection caused pain or discomfort. More than 80% of participants responded positively to all other indicators after the self-collection, including how they felt overall, ease of performance, convenience, and confidence in collecting an accurate sample. All changes before and after the procedure were statistically significant (p < 0.001, Table 4). The most common self-collection posture was one leg raised and supported, which was used by 744 participants (47.4%). This was followed by partial or full squatting (607 participants, 38.6%) and sitting (184 participants, 11.7%).

**Table 4:** Assessment of self-collection acceptability before and after self-collection.

Indicators	Pre-sampling expectations		Post-sampling feedback		p-value
	n	% (95% CI)	n	% (95% CI)	
Positive overall feeling	1042	66.4 (64.0–68.7)	1569	100.0	p < 0.001
Easy to perform	1218	77.6 (75.5–79.6)	1393	88.8 (87.1–90.3)	p < 0.001
Thought it was convenient	1471	93.8 (92.4–94.9)	1519	96.8 (95.8–97.6)	p < 0.001
Not embarrassed	1464	93.3 (92.0–94.4)	1535	97.8 (97.0–98.5)	p < 0.001
No discomfort/pain	631	40.2 (37.8–42.7)	1053	67.1 (64.8–69.4)	p < 0.001
Confident in collecting accurate sample	1142	72.8 (70.5–74.9)	1386	88.3 (86.7–89.8)	p < 0.001

* Missing data were excluded (n = 4).

Footnote: CI, confidence interval.

**Fig 2:**
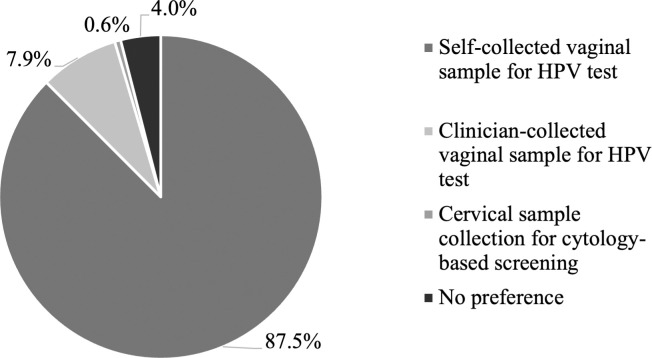
Cervical screening method preference among pregnant women who have undertaken self-collection (n = 1569). A majority of participants (87.5%) chose self-collected vaginal sample for HPV test as the most preferred choice for cervical screening. Among those who preferred clinician-collected method, only a small proportion (0.6%) opted for cytology-based screening. An additional 3.9% of participants had no specific preference for cervical screening methods. Note: Missing data were excluded (n = 4). Footnote: HPV, human papillomavirus.

In terms of cervical screening method preferences, 87.5% of participants favoured the self-collection method, 8.0% opted for clinician-collected HPV-based screening, while 0.6% chose clinician-collected cervical sample for cytology-based screening (Fig 2). Additionally, 3.9% of participants had no specific preference regarding cervical screening methods. 99.0% (1,554/1569) of pregnant women expressed a willingness to repeat a self-collection as their regular cervical screening method in the future (refer to S1 Data).

### Complications during and after self-collection procedure

Of the 1,596 participants who agreed to perform self-collection, 23 (1.4%) of them did not proceed to perform the procedure after researcher’s counselling. Among those who completed self-collection, 16 (1.0%) of them reported experiencing side effects immediately after the sampling procedure. All of them expressed concerns on seeing blood-stained swab and seven of them also complained of discomfort that required clinician’s further examination and consultation (see full list in S1 Table). This was not associated with any significant spotting after self-collection. Most importantly, no adverse pregnancy outcomes or prolonged side effects were reported to the research team throughout the study period.

### Outcomes of self-collection for HPV-based cervical screening during pregnancy

Nearly all participants (1505/1573, 95.7%) who completed self-collection obtained a valid HPV result (refer Table 5). HPV prevalence was reported at 6.3%. Most HPV-positive participants (77/99, 77.8%) had non-HPV16/18 detected. All participants who screened positive for HPV opted for postpartum colposcopy and the attendance rate was 65.6% (65/99). A total of 57.6% (57/99) participants attended within six months, five attended within 12 months, and three attended more than 12 months post-delivery. Additionally, six participants (6.1%) opted to make their own arrangement elsewhere (refer to S2 Table). Among the 65 participants who attended postpartum colposcopy, a total of eight cases (12.3%) of histologically confirmed CIN2 + cases were detected (refer to S3 Table).

**Table 5:** HPV results from successful self-collection for HPV testing (n = 1,573).

Results	n	%
HPV Not Detected	1406	89.4 (93.4)
HPV Detected ^a^	99	6.3 (6.6)
•HPV16	17	1.1 (1.3)
•HPV18	5	0.3 (0.3)
•Non-HPV 16/18	83	5.3 (5.5)
Invalid	68	4.3 (NA)

^a^HPV detection rate presented are rate of valid results (n = 1505) in parentheses.

Note: 5 participants had more than one HPV type infections.

Footnote: HPV, human papillomavirus.

## Discussion

This is the first study that has demonstrated the acceptability and performance of self-collected vaginal FLOQSwabs for HPV-based cervical screening during pregnancy. Over 70% of pregnant women who were invited to participate in this study agreed to perform self-collection. The acceptability of self-collection is high with more than 80% of participants indicated positive responses to at least five (out of six) acceptability indices. HPV prevalence reported in the study population was 6.3% and importantly, *no adverse pregnancy outcomes* were reported in this study. Only a small number of participants (1.0%) experienced immediate self-limiting side effects as part of self-collection.

In this study, 2,256 women above 15 weeks of gestation were invited and 1,603 agreed to participate, indicating an uptake rate of 71.1%. This uptake rate is comparable to what was reported in the general population in a similar setting, where the uptake ranged between 51% and 83% [16,18]. The reason may be associated with several factors, including educational level, cultural lifestyle, and socioeconomic status [20]. Notably, nearly 50% of the participants had never undergone cervical screening, suggesting that integrating self-collection into antenatal care services provides a promising opportunity window to increase cervical screening uptake among the under-screened population. Additionally, almost all participants (99.6%) were willing to self-collect a vaginal sample, with only seven declining. Among those who attempted self-collection, 1.4% were unable to acquire a sample and required assistance from a healthcare practitioner. It is worth noting that two-thirds of the participants were in the third trimester and had significant bump but most managed to perform self-collection.

Due to the high prevalence of first trimester miscarriages, it was decided that no women under 15 weeks into their pregnancy be recruited [21]. However, it is noteworthy that since 2019, Australia has had a governmental recommendation supporting either self-collection or clinician-collected HPV testing during pregnancy, without restrictions on gestational age [22]. In this study, no adverse pregnancy outcomes were reported to the research team among 1,596 study participants (in both 2^nd^ and/or 3^rd^ trimesters) who undertook self-collection. Importantly, no cases of bleeding or premature delivery attributed to self-collection were reported. Obtaining clinician-collected vaginal swabs for different clinical scenarios in pregnancy is common and considered safe [23–25]. To the best of our knowledge, this is the first documented self-collection study using the FLOQSwab for HPV-based cervical screening during pregnancy, and no major complications were observed.

This study demonstrated a high level of acceptance for self-collection among pregnant women. More than 80% of participants reported positive attitudes towards 5 or more acceptability indicators following self-collection. The differences in responses before and after self-collection were statistically significant (p < 0.001). Notably, about 90% of participants reported that self-collection was easy, convenient, not embarrassing and that they were confident in taking their own samples, similar to the findings reported in the general population in similar settings [16,18]. These findings suggest that self-collection during pregnancy can be used as an entry point to improve cervical cancer screening rates, especially among women from the low-income settings. Notably, 91.2% of participants in the lowest income group indicated a preference for self-collected vaginal samples for HPV test, compared to 81.2% in the highest income group (see Table S4). In addition, this study used a simple swab for self-collection, which is no different from the vaginal swab commonly used for bacterial or fungal infection test during pregnancy, hence contributing to a high acceptability of the self-collection procedure. It is also worth acknowledging that all participants received clear, illustrated instructions and had their questions answered before undertaking self-collection. This likely contributed to the high acceptability of self-collection. Previous studies justified that enhancing resources to improve clarity and cultural appropriateness may encourage greater uptake of self-collection [26,27].

Only 4.3% (68/1573) self-collected samples did not yield valid HPV results in this study. The invalid rate recorded in this study falls within the range of invalid rates in other studies of 1.3% to 11.6% [15,28–31] suggesting that pregnancy does not compromise the performance of self-collection. However, it is noteworthy that the invalid rate reported in this study was higher than the recommended rate (<3%) by WHO Target Product Profile for HPV assay. Higher invalid rate is not uncommon in self-collection due to a variety of reasons but one includes the concept of “social invalids” when a women will accept the swab from the clinician but returns it without either taking a sample or notifying the clinician that no sample was taken [32]. This study demonstrated HPV positivity of 6.3% from all participants, or 6.6% in participants with a valid HPV result. Regarding the clinical significance of HPV positivity in pregnancy, we observed that the prevalence in our cohort was not higher than that in the general Malaysian population (6.5%) [32]. This observation suggests that pregnancy does not increase the rate of HPV detection, further supporting the feasibility and potential utility of cervical screening during this period. Nevertheless, we acknowledge that this study does not include a direct comparison with a control group from the general population; rather, the discussion is based on prevalence data reported in published literature on the Malaysian population. The CIN2 + rate for this total cohort was 0.5% (8/1505) which is comparable to about 1% reported in Malaysia [33]. This figure is also similar to other Asian countries such as China and India that reported 1–2% of CIN2 + rate [34]. The CIN2 + detection rate was calculated based on the total number of CIN2 + cases within the overall screening population. However, data were unavailable for 33.3% (33/99) of HPV-positive individuals who were referred for colposcopy. As a result, some CIN2 + cases may have gone undetected and were therefore not included in the final analysis.

While introducing self-collection during pregnancy provides promising opportunity to increase screening coverage, there are considerable challenges in terms of follow-up care. In this study, only 65.7% of participants who were screened HPV-positive attended postpartum colposcopy. This is not surprising as having newborn is challenging for some women to access healthcare services.

### Strength and weakness of the study

This study highlighted the safety, acceptability and performance of self-collection during pregnancy as a potential approach to increase cervical screening participation within antenatal care services. The data reported in this study demonstrated that the accuracy of this method for HPV detection and CIN2 + is comparable to the general population.

However, the findings may not fully represent the broader population, as the study did not assess acceptability among pregnant women residing in rural areas. It is also worth noting that the response rate may underestimate real-world screening participation due to the additional time required for the counselling and questionnaire. Additionally, the use of opportunistic sampling may result in under-representation of under-screened populations, a critical target group for successful screening programs. Lastly, any pregnancy-related outcomes were not systematically collected, but were instead self-reported by participants via a dedicated study mobile number monitored by a study doctor and coordinator, potentially leading to underreporting.

## Conclusion

This study has demonstrated that self-collection in pregnancy using the FLOQSwab provides a safe method of HPV-based cervical screening. These data also indicate that the accuracy of this method for HPV detection and CIN2 + detection are similar to the screening population rate using the same self-collection method.

Antenatal care is a common entry point for women to seek health care especially in resource limited settings. Hence integrating self-collection in antenatal healthcare services is a promising opportunity to improve cervical screening coverage.

## Supporting information

S1 Table Complicated cases associated with self-collection procedure. (PDF)

S2 Table Colposcopy follow up compliance rate and histological outcomes (n = 99). (PDF)

S3 Table Histological diagnosis among participants who attended postpartum colposcopy (n = 65). (PDF)

S4 Table Preference of cervical screening method stratified by household income (n = 1583). (PDF)

S1 Data Dataset. (PDF)
