# Supplementary material for: The acceptability, safety, and performance of primary cervical screening through self-collected vaginal samples in an urban teaching hospital antenatal clinic setting
Source: PLOS Glob Public Health. 2025 Sep 2;5(9):e0005149. doi: 10.1371/journal.pgph.0005149 (PMC12404364; doi:10.1371/journal.pgph.0005149)
Supplement: S1 Table — (PDF) [file pgph.0005149.s001.pdf]

S1 Table. Complicated cases associated with self-collection procedure

No of record	Date of screening	Study ID	Complaint
80	21/09/2021	P1872	Blood on swab tip
402	04/07/2022	P2489	Blood on swab tip
443	01/08/2022	P2524	Blood on swab tip and discomfort
456	09/08/2022	P2536	Blood on swab tip
536	02/11/2022	P2643	Blood on swab tip
578	08/11/2022	P2693	Blood on swab tip
1088	13/02/2023	P3084	Blood on swab tip
1152	20/02/2023	P3123	Blood on swab tip and discomfort
1282	01/03/2023	P3113	Blood on swab tip and discomfort
1362	07/03/2023	P3175	Blood on swab tip and discomfort
1517	22/03/2023	P3312	Blood on swab tip
1569	28/03/2023	P3323	Blood on swab tip and discomfort
1767	13/04/2023	P3447	Blood on swab tip and discomfort
1774	13/04/2023	P3462	Blood on swab tip and discomfort
2308	17/07/2023	P3811	Blood on swab tip
2533	14/09/2023	P3911	Blood on swab tip
