# Supplementary material for: The acceptability, safety, and performance of primary cervical screening through self-collected vaginal samples in an urban teaching hospital antenatal clinic setting
Source: PLOS Glob Public Health. 2025 Sep 2;5(9):e0005149. doi: 10.1371/journal.pgph.0005149 (PMC12404364; doi:10.1371/journal.pgph.0005149)
Supplement: S2 Table — (PDF) [file pgph.0005149.s002.pdf]

S2 Table. Colposcopy follow up compliance rate and histological outcomes (n=99).

Colposcopy follow up status	N (%)
Attended postpartum colposcopy within 6-month time	57 (57.6)
Attended postpartum colposcopy within 7 – 12month time	5 (5.1)
Attended postpartum colposcopy after 12 months	3 (3.0)
Own arrangement	6 (6.1)
Defaulted colposcopy follow up	28 (28.3)

Note: The duration of follow up is calculated (in month) from the date of delivery to the date of colposcopy appointment.
