# Supplementary material for: The acceptability, safety, and performance of primary cervical screening through self-collected vaginal samples in an urban teaching hospital antenatal clinic setting
Source: PLOS Glob Public Health. 2025 Sep 2;5(9):e0005149. doi: 10.1371/journal.pgph.0005149 (PMC12404364; doi:10.1371/journal.pgph.0005149)
Supplement: S3 Table — (PDF) [file pgph.0005149.s003.pdf]

S3 Table. Histological diagnosis among participants who attended postpartum colposcopy (n=65).

Histological diagnosis	N (%)
Normal	39 (60.0)
CIN 1	16 (24.6)
CIN 2+	8 (12.3)
Unsatisfactory evaluation	2 (3.1)

Footnote: CIN, cervical intraepithelial neoplasia
