# Supplementary material for: The acceptability, safety, and performance of primary cervical screening through self-collected vaginal samples in an urban teaching hospital antenatal clinic setting
Source: PLOS Glob Public Health. 2025 Sep 2;5(9):e0005149. doi: 10.1371/journal.pgph.0005149 (PMC12404364; doi:10.1371/journal.pgph.0005149)
Supplement: S4 Table — (PDF) [file pgph.0005149.s004.pdf]

S4 Table. Preference of cervical screening method stratified by household income (n=1583)

	**Cervical Screening Method Preference (N, %)**			
**Household income (RM/month)**	Self-collected vaginal sample for HPV test	Clinician-collected vaginal sample for HPV test	Cervical sample collection for cytology-based screening	No preference
<5,000	413 (91.2)	24 (5.3)	4 (0.9)	12 (2.6)
5,001–10,000	709 (86.8)	70 (8.6)	5 (0.6)	33 (4.0)
>10,000	254 (81.2)	42 (13.4)	1 (0.3)	16 (5.1)

Footnote: RM, ringgit Malaysia; HPV, Human Papillomavirus
