# Supplementary material for: The acceptability, safety, and performance of primary cervical screening through self-collected vaginal samples in an urban teaching hospital antenatal clinic setting
Source: PLOS Glob Public Health. 2025 Sep 2;5(9):e0005149. doi: 10.1371/journal.pgph.0005149 (PMC12404364; doi:10.1371/journal.pgph.0005149)
Supplement: S1 Data — (PDF) [file pgph.0005149.s005.pdf]

No of record	Date of screening	Study ID	Study Initial	Age	Gravida	Para	Miscarriages	Ethnicity	Other ethnicity
1	8/9/2021	P1899	NACK	37	4	3	0	Malay	
2	8/9/2021	P1887	NABMJ	30	3	2	0	Malay	
6	8/9/2021	P1886	AJBMA	42	4	3	0	Malay	
7	8/9/2021	P1885	STMT	34	2	1	0	Malay	
9	8/9/2021	P1898	TYY	37	2	1	0	Chinese	
11	8/9/2021	P1890	NSAS	30	1	0	0	Malay	
12	8/9/2021	P1897	SNBH	30	2	1	0	Malay	
13	8/9/2021	P1895	NSAH	36	5	3	1	Malay	
16	9/9/2021	P1992	NEBMR	34	2	1	0	Malay	
19	9/9/2021	P1989	DGAS	33	2	1	0	Indian	
20	9/9/2021	P1888	NEBMR	30	1	0	0	Malay	
24	9/9/2021	P1889	ABZ	31	2	1	0	Malay	
25	9/9/2021	P1891	NSLBAR	30	3	1	1	Malay	
26	9/9/2021	P1892	AMN	37	3	2	0	Malay	
32	14/9/2021	P1999	SHAPPS	38	2	1	0	Indian	
33	9/9/2021	P1893	NHBB	33	1	0	0	Malay	
34	9/9/2021	P1884	AWH	33	2	1	0	Chinese	
35	9/9/2021	P1896	NABA	33	4	3	0	Malay	
36	13/9/2021	P1986	NAP	38	4	2	1	Indian	
37	13/9/2021	P1987	KBK	38	3	1	1	Malay	
42	13/9/2021	P1995	NYEBMJK	32	6	2	3	Malay	
43	13/9/2021	P1993	NNBI	33	4	2	1	Malay	
44	13/9/2021	P1900	SAK	30	1	0	0	Indian	
46	13/9/2021	P1998	ISBAA	31	3	1	0	Malay	
47	13/9/2021	P1996	NSY	32	2	0	0	Malay	
48	13/9/2021	P1903	NAHBSS	31	5	0	4	Malay	
49	13/9/2021	P1894	RNBRM	35	2	1	0	Malay	
50	14/9/2021	P1991	NBL	32	2	1	0	Malay	
51	14/9/2021	P1901	EAN	30	3	2	0	Others	Bidayuh
52	14/9/2021	P1983	SSBAS	36	4	3	0	Others	Kadazandusun
53	14/9/2021	P1988	NNBAH	35	4	2	0	Malay	
54	14/9/2021	P1984	ANS	34	1	0	0	Indian	

55	14/9/2021	P1997	NBH	30	1	0	0	Malay	
57	14/9/2021	P1985	CMY	37	3	2	0	Chinese	
59	14/9/2021	P1990	NFBK	31	3	2	0	Malay	
61	15/9/2021	P2339	OWY	31	1	0	0	Chinese	
62	15/9/2021	P2338	NABAR	35	1	0	0	Malay	
64	20/9/2021	P2336	NBY	34	3	2	0	Malay	
67	20/9/2021	P2333	NHBMR	35	4	2	1	Malay	
68	20/9/2021	P2334	NBA	35	4	2	1	Malay	
69	20/9/2021	P2337	CCC	38	2	1	0	Chinese	
74	21/9/2021	P1875	NBAA	32	1	0	0	Malay	
76	21/9/2021	P1871	HBZ	31	2	0	1	Malay	
78	21/9/2021	P2000	AYMY	35	1	0	0	Chinese	
80	21/9/2021	P1872	NWW	42	3	2	0	Chinese	
81	22/9/2021	P1910	SFBS	37	3	1	1	Malay	
82	22/9/2021	P1876	NBD	36	3	1	1	Malay	
83	22/9/2021	P1912	TKM	40	3	1	0	Chinese	
84	22/9/2021	P1877	NBR	33	1	0	0	Malay	
85	22/9/2021	P1916	TSK	36	3	0	2	Chinese	
86	22/9/2021	P1873	NSBZ	31	2	1	0	Malay	
88	22/9/2021	P1874	UAG	32	4	2	1	Indian	
89	22/9/2021	P1911	SDBSA	33	1	0	0	Malay	
91	22/9/2021	P2335	NNBA	38	5	2	2	Malay	
92	22/9/2021	P1908	JAPM	36	3	1	1	Indian	
93	22/9/2021	P1915	AAPJ	35	1	0	0	Indian	
96	23/9/2021	P1982	NSBMS	34	1	0	0	Malay	
97	23/9/2021	P1905	NHBH	33	2	0	1	Malay	
98	23/9/2021	P1906	NBZA	35	2	1	0	Malay	
100	23/9/2021	P1909	LMK	33	2	1	0	Chinese	
101	23/9/2021	P1914	NAR	33	5	0	4	Indian	
102	23/9/2021	P1913	NBMT	31	3	2	0	Malay	
104	23/9/2021	P1907	SALL	36	3	2	0	Others	Bidayuh
106	23/9/2021	P1918	VSY	33	2	0	1	Chinese	
107	23/9/2021	P1917	AABR	38	1	0	0	Malay	

108	23/9/2021	P1919	NEYBML	31	3	2	0	Malay
109	23/9/2021	P1920	TAPK	32	2	1	0	Indian
110	27/9/2021	P1904	DI	36	1	0	0	Chinese
111	27/9/2021	P1925	ABA	36	1	0	0	Malay
116	29/9/2021	P1923	NBI	32	6	3	2	Malay
117	29/9/2021	P1924	CSY	35	1	0	0	Chinese
118	29/9/2021	P1921	SBAM	40	2	1	0	Malay
119	29/9/2021	P1922	LHB	34	2	1	0	Chinese
120	29/9/2021	P1926	NBK	33	1	0	0	Malay
121	29/9/2021	P1927	NABA	35	3	2	0	Malay
122	29/9/2021	P1928	KAV	34	2	1	0	Indian
124	29/9/2021	P1929	NBJ	33	2	1	0	Malay
126	29/9/2021	P1932	ZBZ	33	6	2	3	Malay
127	4/10/2021	P1930	NHBJ	36	4	2	1	Malay
129	4/10/2021	P1933	NHBB	32	3	2	0	Malay
130	5/10/2021	P1931	NNBN	35	4	3	0	Malay
132	5/10/2021	P1936	MBMA	39	5	3	1	Malay
135	6/10/2021	P1939	SABS	37	4	3	0	Malay
137	6/10/2021	P1942	SRBMI	38	5	2	2	Malay
138	6/10/2021	P1940	NBA	38	5	4	0	Malay
139	6/10/2021	P1941	FABH	30	1	0	0	Malay
141	6/10/2021	P1938	NSABA	32	2	1	0	Malay
144	6/10/2021	P1937	NABR	30	4	0	3	Malay
145	7/10/2021	P1935	SBM	36	1	0	0	Malay
146	11/10/2021	P1934	MBM	35	4	0	3	Malay
147	11/10/2021	P2351	NHBY	34	1	0	0	Malay
148	11/10/2021	P2350	NNABNO	32	2	1	0	Malay
149	13/10/2021	P2346	NFBS	31	3	2	0	Malay
150	13/10/2021	P2348	SEBMY	31	2	1	0	Malay
151	13/10/2021	P2352	SAU	31	4	1	2	Indian
152	13/10/2021	P2349	NABMZ	32	3	1	1	Malay
153	13/10/2021	P2347	SBAR	32	2	1	0	Malay
154	14/10/2021	P2345	NWBAR	32	1	0	0	Malay

155	14/10/2021	P2344	MNBA	37	1	0	0	Malay
156	14/10/2021	P1943	ZHBZ	31	3	1	1	Malay
157	14/10/2021	P2343	NHBMI	31	2	1	0	Malay
158	14/10/2021	P2342	ZBH	34	3	2	0	Malay
159	14/10/2021	P1944	ABA	31	2	1	0	Malay
160	14/10/2021	P1945	MBM	32	3	2	0	Malay
161	14/10/2021	P1946	NFBS	31	1	0	0	Malay
163	18/10/2021	P1949	FLYF	39	3	1	1	Chinese
164	18/10/2021	P1950	NA	35	6	5	0	Malay
170	20/10/2021	P1952	NIBR	30	2	0	1	Malay
171	20/10/2021	P1954	NBH	30	3	2	0	Malay
173	27/10/2021	P1953	KAP	30	3	1	1	Indian
174	27/10/2021	P1955	JLCF	33	2	0	1	Chinese
175	27/10/2021	P1951	NSBZ	37	3	2	0	Malay
177	1/11/2021	P1969	AAP	32	2	1	0	Indian
179	1/11/2021	P1970	NBS	36	3	2	0	Malay
180	1/11/2021	P1981	CBS	31	1	0	0	Chinese
182	3/11/2021	P1971	NSBMS	30	1	0	0	Malay
183	3/11/2021	P1972	NFBMN	36	4	3	0	Malay
184	3/11/2021	P1973	LKY	32	5	3	1	Chinese
186	3/11/2021	P1974	FABMY	36	4	1	2	Malay
189	8/11/2021	P1975	NBR	31	5	4	0	Malay
190	10/11/2021	P1976	ABA	37	5	4	0	Malay
192	10/11/2021	P1977	NBMA	31	5	4	0	Malay
193	15/11/2021	P1980	NBN	34	3	2	0	Malay
194	15/11/2021	P1968	NABI	30	2	1	0	Malay
195	22/11/2021	P1956	NJBD	30	5	3	1	Malay
196	22/11/2021	P1957	NBR	30	2	1	0	Malay
197	22/11/2021	P1958	ZMBMK	33	1	0	0	Malay
198	22/11/2021	P1959	SBS	34	3	2	0	Malay
199	22/11/2021	P1960	TAR	37	3	2	0	Indian
200	22/11/2021	P1961	NSBM	37	2	1	0	Malay
201	22/11/2021	P1962	NABAJ	33	3	2	0	Malay

202	22/11/2021	P1963	MM	36	2	1	0	Others	Dusun
203	22/11/2021	P1964	NNBAK	33	2	1	0	Malay	
204	22/11/2021	P1965	NHBZ	31	7	4	2	Malay	
207	23/11/2021	P1966	NABA	35	2	1	0	Malay	
209	23/11/2021	P1967	NFHBS	35	3	2	0	Malay	
210	23/11/2021	P2392	NBA	39	5	3	1	Malay	
211	23/11/2021	P2393	WNABWY	37	3	2	0	Malay	
213	23/11/2021	P2394	BKAJS	33	2	1	0	Others	Sikh
214	23/11/2021	P2395	NBM	33	3	2	0	Malay	
215	23/11/2021	P2396	SNZBANZ	32	2	1	0	Malay	
216	23/11/2021	P2397	TBAR	38	5	2	2	Malay	
217	24/11/2021	P2398	MAP	36	6	3	2	Indian	
218	24/11/2021	P2399	ABW	31	2	1	0	Malay	
219	24/11/2021	P2400	NABO	32	2	1	0	Malay	
220	24/11/2021	P2401	NABH	34	4	3	0	Malay	
221	24/11/2021	P2402	RBAR	43	6	4	1	Malay	
222	24/11/2021	P2403	SBN	36	1	0	0	Malay	
223	24/11/2021	P2404	RBMA	31	2	1	0	Malay	
224	24/11/2021	P2366	NSBMH	33	1	0	0	Malay	
225	25/11/2021	P2367	NFBS	35	1	0	0	Malay	
226	25/11/2021	P2368	SSBS	37	6	1	4	Malay	
227	25/11/2021	P2369	NDBI	36	4	2	1	Malay	
228	25/11/2021	P2370	NABB	36	5	3	1	Malay	
229	25/11/2021	P2371	MAS	35	4	1	2	Indian	
230	30/11/2021	P2372	UNBH	30	2	1	0	Malay	
231	30/11/2021	P2373	SKBAL	32	3	2	0	Malay	
232	30/11/2021	P2374	SABMY	35	2	1	0	Malay	
234	2/12/2021	P2375	AHBMM	37	3	2	0	Malay	
235	2/12/2021	P2376	KTY	37	1	0	0	Chinese	
238	8/12/2021	P2377	YBA	36	4	2	1	Malay	
239	8/12/2021	P2378	NABM	36	2	1	0	Malay	
240	8/12/2021	P2365	MBA	39	4	3	0	Malay	
241	9/12/2021	P2353	KAP	32	2	1	0	Indian	

242	9/12/2021	P2354	IFBI	31	3	2	0	Malay	
243	14/12/2021	P2364	NHBM	35	3	0	2	Malay	
246	16/12/2021	P2355	NSBJ	33	2	1	0	Malay	
248	16/12/2021	P2356	NBAK	38	4	3	0	Malay	
250	21/12/2021	P2357	SFNBO	35	3	2	0	Malay	
251	21/12/2021	P2358	NBS	42	6	2	3	Malay	
252	21/12/2021	P2359	NABO	30	2	1	0	Malay	
254	23/12/2021	P2362	CJ	32	1	0	0	Chinese	
255	4/1/2022	P2361	EYBMS	34	2	1	0	Malay	
256	6/1/2022	P2380	NABMA	31	4	2	1	Malay	
257	6/1/2022	P2360	HBAN	35	3	2	0	Malay	
258	6/1/2022	P2379	NZBA	34	3	2	0	Malay	
259	6/1/2022	P2381	YAV	32	6	0	5	Indian	
260	6/1/2022	P2382	NLBAM	33	4	3	0	Malay	
261	11/1/2022	P2383	HSBI	37	3	2	0	Malay	
268	13/1/2022	P2384	LKY	39	2	1	0	Chinese	
270	13/1/2022	P2385	CMK	37	1	0	0	Chinese	
271	13/1/2022	P2386	IABMK	31	6	2	3	Malay	
272	13/1/2022	P2387	JLYY	40	1	0	0	Chinese	
274	20/1/2022	P2388	KABO	34	4	2	1	Malay	
277	20/1/2022	P2389	SBMI	40	4	1	2	Malay	
279	25/1/2022	P2390	NHBMH	34	7	4	1	Malay	
280	25/1/2022	P2391	MPM	33	1	0	0	Chinese	
281	27/1/2022	P2405	HJ	31	3	1	1	Others	Dusun
282	27/1/2022	P2406	SBS	35	5	3	1	Malay	
284	27/1/2022	P2407	NSBK	34	7	2	4	Malay	
285	27/1/2022	P2408	NTBMJ	36	3	1	1	Malay	
286	27/1/2022	P2409	NBI	34	3	2	0	Malay	
288	27/1/2022	P2410	HBH	35	4	3	0	Malay	
290	7/2/2022	P2411	NMBY	38	6	3	2	Malay	
293	8/2/2022	P2412	SSBA	32	3	2	0	Malay	
294	9/2/2022	P2413	NIBBS	30	1	0	0	Malay	
295	9/2/2022	P2414	NBZ	33	4	3	0	Malay	

297	10/2/2022	P2415	MBS	38	5	2	2	Malay
298	10/2/2022	P2416	EBNH	32	3	2	0	Malay
299	10/2/2022	P2418	NHBMG	36	4	2	1	Malay
300	10/2/2022	P2419	JBJ	35	4	2	1	Malay
301	15/2/2022	P2420	NBAG	31	4	2	1	Malay
302	15/2/2022	P2421	NABR	35	5	4	0	Malay
303	15/2/2022	P2422	NFBR	33	3	2	0	Malay
305	24/2/2022	P2423	RBMN	37	4	3	0	Malay
307	24/2/2022	P2424	PATV	30	2	0	1	Indian
309	24/2/2022	P2425	SBAAS	32	3	1	1	Malay
310	24/2/2022	P2426	AIBR	30	1	0	0	Malay
316	8/3/2022	P2427	LYC	38	2	1	0	Chinese
317	8/3/2022	P2428	SNBMH	34	1	0	0	Malay
318	29/3/2022	P2430	ABZ	31	4	3		Malay
323	5/4/2022	P2429	RBMI	38	5	2	2	Malay
326	7/4/2022	P2444	SSM	30	1	0	0	Chinese
328	7/4/2022	P2446	SAM	33	1	0	0	Indian
329	7/4/2022	P2447	MAR	39	1	0	0	Indian
332	10/5/2022	P2448	ABA	38	3	2	0	Malay
334	25/5/2022	P2449	NABNR	34	3	2	0	Malay
335	25/5/2022	P2450	NBS	37	4	3	0	Malay
336	25/5/2022	P2451	NBR	31	2	1	0	Malay
337	31/5/2022	P2452	SSBMR	35	3	1	1	Malay
338	13/6/2022	P2453	NBG	33	3	2	0	Malay
339	13/6/2022	P2454	RABM	30	1	0	0	Malay
341	13/6/2022	P2456	NNBAM	35	4	3	0	Malay
342	13/6/2022	P2455	DABMS	34	3	2	0	Malay
343	13/6/2022	P2431	SABMS	33	3	2	0	Malay
345	13/6/2022	P2433	ABAB	36	3	2	0	Malay
346	15/6/2022	P2432	SNHBAH	35	5	3	1	Malay
347	16/6/2022	P2434	NAQBZ	31	1	0	0	Malay
348	16/6/2022	P2435	NBS	31	1	0	0	Malay
349	17/6/2022	P2443	SNHBAJ	39	5	3	1	Malay

350	17/6/2022	P2442	ZBZA	38	4	2	1	Malay
351	17/6/2022	P2436	SSBS	31	4	2	1	Malay
358	20/6/2022	P2439	WWY	30	1	0	0	Chinese
360	20/6/2022	P2437	ABA	37	2	1	0	Malay
361	20/6/2022	P2438	SSBA	32	5	2	2	Malay
362	20/6/2022	P2440	NDBI	32	2	1	0	Malay
363	20/6/2022	P2441	NBBN	32	5	3	1	Malay
365	21/6/2022	P2457	NHBA	34	4	3	0	Malay
366	21/6/2022	P2458	FSBA	30	3	1	1	Malay
367	22/6/2022	P2459	NBM	33	5	3	1	Malay
368	22/6/2022	P2460	NHBMS	31	3	1	1	Malay
369	22/6/2022	P2461	HBA	41	3	1	1	Malay
370	23/6/2022	P2462	NABZ	30	3	2	0	Malay
371	23/6/2022	P2463	MBM	35	3	2	0	Malay
372	23/6/2022	P2464	NHBAJ	30	2	1	0	Malay
373	23/6/2022	P2465	SNMBJ	30	2	1	0	Malay
374	23/6/2022	P2466	SSBAB	32	3	1	1	Malay
375	23/6/2022	P2467	CABCH	36	5	4	0	Malay
376	24/6/2022	P2468	RBL	32	2	1	0	Malay
377	24/6/2022	P2469	NHBM	33	4	2	1	Malay
379	24/6/2022	P2470	SABAR	35	6	5	0	Malay
380	27/6/2022	P2488	NABZA	33	5	3	1	Malay
381	27/6/2022	P2471	NBAB	32	3	2	0	Malay
382	27/6/2022	P2472	ZBM	32	3	2	0	Malay
386	27/6/2022	P2473	NMRBI	35	4	3	0	Malay
387	27/6/2022	P2474	NHBS	31	3	1	1	Malay
388	27/6/2022	P2475	NJBA	39	11	7	3	Malay
389	28/6/2022	P2476	RBM	37	5	2	2	Malay
390	28/6/2022	P2477	HBMZ	38	3	2	0	Malay
391	28/6/2022	P2478	NABMK	37	3	2	0	Malay
392	28/6/2022	P2479	SSBI	37	4	3	0	Malay
393	28/6/2022	P2480	NHBP	34	4	1	2	Malay
394	28/6/2022	P2481	TAEN	31	3	1	1	Indian

395	28/6/2022	P2482	ZBA	38	3	2	0	Indian
396	28/6/2022	P2483	NBI	34	3	1	1	Malay
397	30/6/2022	P2484	NABA	32	3	2	0	Malay
398	4/7/2022	P2485	NLBMN	36	3	2	0	Malay
399	4/7/2022	P2486	NABMZ	38	4	1	2	Malay
401	4/7/2022	P2487	AZBAK	33	4	2	1	Malay
402	4/7/2022	P2489	KFBS	32	1	0	0	Malay
403	4/7/2022	P2490	RBO	36	3	2	0	Malay
404	4/7/2022	P2491	KBZ	32	2	1	0	Malay
405	4/7/2022	P2492	NBAR	31	3	2	0	Malay
406	5/7/2022	P2493	VAN	33	6	2	3	Indian
407	5/7/2022	P2494	NBH	35	3	2	0	Malay
408	5/7/2022	P2495	NFBS	35	3	2	0	Malay
409	6/7/2022	P2496	NSBM	32	4	3	0	Malay
410	6/7/2022	P2497	SZBB	36	4	2	1	Malay
411	7/7/2022	P2498	LKM	35	4	1	2	Chinese
413	7/7/2022	P2499	NABAH	36	3	0	2	Malay
414	7/7/2022	P2500	NBA	37	1	0	0	Malay
415	8/7/2022	P2501	SKAHS	32	3	2	0	Indian
416	8/7/2022	P2502	ALBN	31	3	2	0	Malay
417	12/7/2022	P2503	NABK	32	3	2	0	Malay
418	12/7/2022	P2504	NABO	30	4	3	0	Malay
419	15/7/2022	P2505	NJBMY	30	3	2	0	Malay
420	16/7/2022	P2506	NABAM	35	3	2	0	Malay
421	16/7/2022	P2507	ZBS	38	3	2	0	Malay
422	18/7/2022	P2508	PCVY	30	1	0	0	Chinese
423	18/7/2022	P2509	SNBM	36	1	0	0	Malay
424	18/7/2022	P2510	LPS	40	3	1	1	Chinese
425	18/7/2022	P2511	RBM	41	9	4	4	Malay
427	18/7/2022	P2512	SNBA	34	1	0	0	Malay
428	18/7/2022	P2513	SBY	31	3	2	0	Malay
429	18/7/2022	P2514	ABAR	43	2	1	0	Malay
433	18/7/2022	P2515	MBAR	31	2	1	0	Malay

434	18/7/2022	P2516	MBM	38	7	4	2	Malay	
435	18/7/2022	P2517	KAN	42	6	0	5	Indian	
436	18/7/2022	P2518	NABMN	33	3	1	1	Malay	
437	22/7/2022	P2519	NABZ	32	1	0	0	Malay	
438	25/7/2022	P2521	CNAEBCNA	41	5	2	2	Malay	
442	1/8/2022	P2523	NHBMA	31	2	1	0	Malay	
443	1/8/2022	P2524	NFBAH	33	1	0	0	Malay	
444	1/8/2022	P2525	NABAS	33	3	2	0	Malay	
445	1/8/2022	P2526	IAP	31	4	3	0	Others	Iban
446	1/8/2022	P2527	NSBMM	30	1	0	0	Malay	
447	1/8/2022	P2528	NABH	34	4	2	1	Malay	
448	1/8/2022	P2529	SDBU	40	3	2	0	Malay	
449	1/8/2022	P2530	NBI	41	3	2	0	Malay	
451	5/8/2022	P2522	IKBZ	36	2	1	0	Malay	
452	8/8/2022	P2532	NABA	31	2	1	0	Malay	
453	8/8/2022	P2533	NBI	33	3	1	1	Malay	
454	9/8/2022	P2534	MBMM	30	1	0	0	Malay	
455	9/8/2022	P2535	NBAN	31	3	2	0	Malay	
456	9/8/2022	P2536	UAV	32	3	1	1	Indian	
458	15/8/2022	P2537	NFBMJ	33	2	0	1	Malay	
460	15/8/2022	P2538	NBI	37	3	1	1	Malay	
461	15/8/2022	P2539	NBM	38	2	1	0	Malay	
462	15/8/2022	P2540	SAL	32	4	1	2	Indian	
463	16/8/2022	P2561	SFBM	30	1	0	0	Malay	
464	16/8/2022	P2562	SFBSSB	30	2	1	0	Malay	
465	16/8/2022	P2563	NSBYK	30	1	0	0	Malay	
467	22/8/2022	P2564	NBAA	35	3	2	0	Malay	
468	22/8/2022	P2565	IABR	32	1	0	0	Malay	
469	22/8/2022	P2566	KBR	31	4	2	1	Malay	
470	22/8/2022	P2567	RBMS	35	4	1	2	Malay	
472	22/8/2022	P2568	HSASS	30	2	0	1	Indian	
473	22/8/2022	P2569	LIBI	30	3	2	0	Malay	
474	22/8/2022	P2570	VAK	37	2	1	0	Indian	

476	29/8/2022	P2572	ANBN	37	2	1	0	Malay
477	29/8/2022	P2571	NBAR	31	1	0	0	Malay
478	29/8/2022	P2574	NHBS	30	4	2	1	Malay
479	29/8/2022	P2575	NBN	34	4	2	1	Malay
480	29/8/2022	P2576	NIBM	35	1	0	0	Malay
481	29/8/2022	P2577	NHBNAR	39	4	2	1	Malay
482	29/8/2022	P2578	NSBS	31	2	1	0	Malay
483	30/8/2022	P2579	FNBA	41	7	3	3	Malay
484	30/8/2022	P2580	SMBM	31	2	1	0	Malay
485	30/8/2022	P2581	UIBMY	30	3	1	1	Malay
486	30/8/2022	P2582	NBMM	38	3	2	0	Malay
487	31/8/2022	P2583	NBM	38	5	4	0	Malay
488	31/8/2022	P2584	MBM	36	4	2	1	Malay
491	8/9/2022	P2585	NABJ	37	5	3	1	Malay
492	8/9/2022	P2586	SRBH	30	3	2	0	Malay
493	14/9/2022	P2588	NDBMI	36	3	2	0	Malay
494	14/9/2022	P2589	SNBWN	34	3	2	0	Malay
495	14/9/2022	P2590	WNBWAH	35	1	0	0	Malay
496	26/9/2022	P2591	MBMS	43	3	1	1	Malay
498	28/9/2022	P2592	RBB	36	5	4	0	Malay
499	3/10/2022	P2593	NNBA	32	3	2	0	Malay
500	3/10/2022	P2594	MBT	41	7	6		Malay
501	4/10/2022	P2595	NABMK	31	3	1	0	Malay
502	4/10/2022	P2596	HBA	32	3	2	0	Malay
504	25/10/2022	P2597	INBI	34	1	0	0	Malay
506	25/10/2022	P2598	SSBS	36	5	1	1	Malay
507	26/10/2022	P2606	NBMS	32	1	0	0	Malay
508	27/10/2022	P2604	SABI	32	2	1	0	Malay
510	27/10/2022	P2603	BAO	36	2	1	0	Indian
513	28/10/2022	P2599	NABMS	33	2	1	0	Malay
514	31/10/2022	P2602	NBAH	33	1	0	0	Malay
515	31/10/2022	P2600	NFBAR	36	4	2	1	Malay
516	31/10/2022	P2601	NBMK	36	2	1	0	Malay

517	31/10/2022	P2605	NBA	42	5	4	0	Malay
521	1/11/2022	P2607	NBA	34	9	7	1	Malay
522	1/11/2022	P2608	LPS	40	2	1	0	Chinese
523	1/11/2022	P2609	NSBMS	32	5	3	1	Malay
525	1/11/2022	P2611	RBB	34	4	3	0	Malay
526	1/11/2022	P2612	MAS	37	2	1	0	Indian
527	2/11/2022	P2618	SHBZ	34	5	4	0	Malay
528	2/11/2022	P2619	RBAW	30	2	0	1	Malay
531	2/11/2022	P2641	CSW	39	3	2	0	Chinese
532	2/11/2022	P2642	USAR	39	1	0	0	Indian
533	2/11/2022	P2617	MAV	41	3	2	0	Indian
536	2/11/2022	P2643	NMBY	37	1	0	0	Malay
537	2/11/2022	P2616	GAM	38	2	1	0	Indian
538	2/11/2022	P2644	SNJBAL	30	5	2	0	Malay
539	2/11/2022	P2645	NABMA	39	7	5	1	Malay
540	2/11/2022	P2613	SAB	30	1	0	0	Indian
541	3/11/2022	P2633	MBJ	41	5	3	1	Malay
542	3/11/2022	P2634	TZJ	31	1	0	0	Chinese
543	3/11/2022	P2635	BAP	34	2	1	1	Indian
544	3/11/2022	P2636	SMBH	36	5	3	1	Malay
545	3/11/2022	P2637	CSM	30	2	0	1	Chinese
546	3/11/2022	P2638	SKBB	37	3	1	1	Malay
547	4/11/2022	P2610	JL	33	5	1	3	Indian
548	4/11/2022	P2639	SBAH	41	3	2	0	Malay
549	4/11/2022	P2640	RBY	37	4	3	0	Malay
550	4/11/2022	P2685	SNFBA	30	3	3	0	Malay
551	4/11/2022	P2686	NAR	33	5	1	3	Indian
571	7/11/2022	P2687	MBMR	34	2	1	0	Malay
572	7/11/2022	P2688	NACA	33	3	1	1	Malay
573	7/11/2022	P2690	LWL	31	1	0	0	Chinese
574	7/11/2022	P2691	NBMN	36	2	1	0	Malay
575	7/11/2022	P2689	NABS	41	7	6	0	Malay
576	7/11/2022	P2692	YKS	38	2	1	0	Chinese

578	8/11/2022	P2693	VKASAS	37	2	1	0	Indian
579	8/11/2022	P2694	NBI	31	2	1	0	Malay
580	8/11/2022	P2695	FIBZ	30	2	1	0	Malay
581	8/11/2022	P2696	KPW	35	2	1	0	Chinese
582	8/11/2022	P2697	NBAZ	34	3	2	0	Malay
583	8/11/2022	P2659	YTC	31	2	1	0	Chinese
585	9/11/2022	P2660	BBR	31	1	0	0	Malay
586	9/11/2022	P2661	CSS	40	1	0	0	Chinese
587	9/11/2022	P2662	MNBM	30	3	1	1	Malay
588	9/11/2022	P2663	KAV	32	1	0	0	Indian
589	9/11/2022	P2664	CSY	30	3	1	1	Chinese
590	9/11/2022	P2665	KYV	36	2	1	0	Chinese
591	9/11/2022	P2666	LYL	34	2	1	0	Chinese
592	9/11/2022	P2615	BAB	36	2	1	0	Indian
593	9/11/2022	P2614	HHBM	37	4	2	1	Malay
594	9/11/2022	P2620	SMSBA	32	1	0	0	Malay
595	10/11/2022	P2621	NABM	33	6	2	3	Malay
596	10/11/2022	P2622	ABA	37	4	1	2	Malay
597	10/11/2022	P2667	SAIBS	31	3	1	1	Malay
598	10/11/2022	P2623	NIBMA	31	3	2	0	Malay
599	10/11/2022	P2668	CMK	34	2	1	0	Chinese
600	10/11/2022	P2624	RBW	41	1	0	0	Malay
601	10/11/2022	P2669	RAM	32	2	0	1	Indian
602	10/11/2022	P2625	JBK	36	4	3	0	Malay
603	10/11/2022	P2670	NBMB	37	4	2	1	Malay
604	10/11/2022	P2671	NBA	36	3	2	0	Malay
605	10/11/2022	P2698	YMK	33	2	1	0	Chinese
606	10/11/2022	P2699	SBAR	34	3	2	0	Malay
607	10/11/2022	P2626	EZBM	39	5	3	1	Malay
608	10/11/2022	P2627	KBA	36	2	0	1	Malay
609	10/11/2022	P2628	NSABM	34	4	3	0	Malay
610	11/11/2022	P2700	ABS	36	9	2	6	Malay
611	11/11/2022	P2701	NHBNA	31	3	0	2	Malay

612	11/11/2022	P2702	NBH	31	4	3	0	Malay	
613	11/11/2022	P2703	SNBMS	37	3	3	0	Malay	
614	12/11/2022	P2629	SABAM	37	2	1	0	Malay	
616	13/11/2022	P2630	ABMY	31	3	2	0	Malay	
617	14/11/2022	P2704	NBAM	32	1	0	0	Malay	
619	14/11/2022	P2705	SBI	35	1	0	0	Malay	
620	14/11/2022	P2706	HBH	32	3	2	0	Malay	
621	14/11/2022	P2707	OHH	36	1	0	0	Chinese	
622	15/11/2022	P2708	VOBH	32	2	1	0	Chinese	
623	14/11/2022	P2709	LPY	36	3	1	0	Chinese	
631	15/11/2022	P2710	LSE	31	1	0	0	Chinese	
632	15/11/2022	P2745	ASBAB	30	3	1	1	Malay	
633	17/11/2022	P2746	NFZBB	32	3	2	0	Malay	
635	15/11/2022	P2747	DNSBAS	30	1	0	0	Malay	
637	15/11/2022	P2748	NBH	34	4	3	0	Malay	
640	16/11/2022	P2749	NDSBA	31	1	0	0	Malay	
641	16/11/2022	P2737	VAS	32	3	1	1	Indian	
642	16/11/2022	P2738	FPK	31	4	1	2	Chinese	
643	16/11/2022	P2739	PAM	30	1	0	0	Indian	
644	16/11/2022	P2631	NABA	31	1	0	0	Malay	
645	16/11/2022	P2740	VAB	37	5	2	2	Indian	
646	16/11/2022	P2741	SBBSH	35	7	3	3	Malay	
647	16/11/2022	P2742	CKL	32	3	2	0	Chinese	
648	16/11/2022	P2632	ASBA	34	3	2	0	Malay	
649	16/11/2022	P2743	NHBM	30	1	0	0	Malay	
652	17/11/2022	P2744	NBR	32	4	2	1	Malay	
653	17/11/2022	P2646	NBL	39	5	3	1	Malay	
655	17/11/2022	P2647	NABN	35	4	3	0	Malay	
656	17/11/2022	P2728	NSBMM	30	1	0	0	Malay	
657	17/11/2022	P2648	RBMS	31	5	2	2	Malay	
658	17/11/2022	P2729	TYC	33	6	2	3	Chinese	
660	17/11/2022	P2730	SAKM	30	2	0	1	Indian	
661	17/11/2022	P2649	NBABL	35	5	2	2	Others	Bidayuh

662	17/11/2022	P2731	CSY	36	2	0	1	Chinese
663	17/11/2022	P2650	SAK	30	1	0	0	Indian
666	21/11/2022	P2732	SABAH	32	4	3	0	Malay
668	21/11/2022	P2733	CSN	30	5	2	2	Chinese
669	21/11/2022	P2734	NSBM	37	4	3	0	Malay
670	22/11/2022	P2735	NABN	35	2	1	0	Malay
671	22/11/2022	P2736	OYSE	34	2	1	0	Chinese
672	22/11/2022	P2724	FHSH	33	1	0	0	Chinese
673	22/11/2022	P2725	NABI	35	3	2	0	Malay
674	10/3/2023	P2726	CZX	33	3	2	0	Chinese
675	22/11/2022	P2727	TSW	32	1	0	0	Chinese
676	17/1/2023	P2750	NBS	31	3	1	1	Malay
677	23/11/2022	P2651	NABJ	32	3	2	0	Malay
678	23/11/2022	P2751	WFBWA	32	4	2	1	Malay
679	23/11/2022	P2752	RAR	37	3	2	0	Indian
680	23/11/2022	P2753	NPW	34	3	2	0	Chinese
681	23/11/2022	P2652	BAB	35	1	0	0	Indian
682	23/11/2022	P2754	ENSBMS	31	2	1	0	Malay
683	23/11/2022	P2755	CCC	31	1	0	0	Chinese
688	23/11/2022	P2653	NHBP	32	1	0	0	Malay
689	23/11/2022	P2654	INBM	33	2	1	0	Malay
690	24/11/2022	P2756	HHBK	34	3	2	0	Malay
691	24/11/2022	P2757	KAS	31	4	2	1	Indian
692	24/11/2022	P2758	LSJ	35	2	0	1	Chinese
693	24/11/2022	P2655	FIBZ	37	3	1	0	Malay
694	24/11/2022	P2759	LJM	30	1	0	0	Chinese
695	24/11/2022	P2656	FBMR	37	2	1	0	Malay
698	24/11/2022	P2657	NBJ	36	3	2	0	Malay
701	24/11/2022	P2760	GMAG	36	1	0	0	Indian
702	24/11/2022	P2658	NNBMA	34	3	1	1	Malay
703	24/11/2022	P2673	KAK	35	4	2	1	Indian
704	24/11/2022	P2672	SBMA	35	4	3	0	Malay
706	25/11/2022	P2761	LAGK	39	3	1	1	Indian

708	25/11/2022	P2762	LPW	35	2	1	0	Chinese	
709	25/11/2022	P2763	RNBRR	36	7	1	5	Malay	
710	25/11/2022	P2764	NLABMZ	30	2	1	0	Malay	
716	29/11/2022	P2765	NFBM	36	7	2	4	Malay	
717	29/11/2022	P2766	SAS	32	1	0	0	Indian	
718	29/11/2022	P2768	NKY	34	2	1	0	Chinese	
720	29/11/2022	P2767	FBN	36	1	0	0	Malay	
721	29/11/2022	P2769	NBMY	34	2	1	0	Malay	
722	29/11/2022	P2770	NSSBAB	30	3	1	1	Malay	
728	30/11/2022	P2771	JWEY	32	1	0	0	Chinese	
729	30/11/2022	P2772	NBMP	30	1	0	0	Malay	
730	30/11/2022	P2773	SRBS	37	1	0	0	Malay	
733	30/11/2022	P2774	EAL	37	3	2	0	Others	Iban
734	30/11/2022	P2775	NBM	36	4	3	0	Malay	
740	1/12/2022	P2776	TAG	30	2	1	0	Indian	
741	1/12/2022	P2778	TZW	37	2	1	0	Chinese	
742	1/12/2022	P2777	NBK	42	9	6	2	Malay	
745	1/12/2022	P2779	SLY	36	3	2	0	Chinese	
746	1/12/2022	P2780	NBP	34	3	2	0	Malay	
747	1/12/2022	P2781	SBA	33	1	0	0	Malay	
748	1/12/2022	P2782	CSV	41	4	1	2	Chinese	
751	2/12/2022	P2783	NABM	39	10	6	3	Malay	
752	2/12/2022	P2784	PAR	38	1	0	0	Indian	
753	2/12/2022	P2785	EJ	30	1	0	0	Others	Dusun
754	2/12/2022	P2786	MBM	35	2	1	0	Malay	
756	5/12/2022	P2787	WMY	32	1	0	0	Chinese	
757	5/12/2022	P2788	SKAMD	35	2	1	0	Indian	
759	5/12/2022	P2789	FBI	31	3	2	0	Malay	
760	5/12/2022	P2791	KSL	34	2	1	0	Chinese	
761	5/12/2022	P2790	CGC	35	2	1	0	Chinese	
762	5/12/2022	P2792	LLS	39	4	2	1	Chinese	
763	5/12/2022	P2793	JBR	35	6	5	0	Malay	
764	5/12/2022	P2794	NWLBMN	31	3	2	0	Malay	

766	6/12/2022	P2801	DASBS	31	1	0	0	Malay	
767	6/12/2022	P2796	CTY	36	2	1	0	Chinese	
768	6/12/2022	P2795	CAF	36	3	1	1	Chinese	
769	6/12/2022	P2797	SNHBAR	34	3	2	0	Malay	
771	6/12/2022	P2798	AMBMN	31	1	0	0	Malay	
772	6/12/2022	P2799	DTKF	34	1	0	0	Chinese	
773	6/12/2022	P2800	STBMW	37	3	1		Malay	
774	6/12/2022	P2802	TSP	33	2	1	0	Chinese	
775	6/12/2022	P2803	RAV	33	4	1	2	Indian	
776	7/12/2022	P2804	CMT	32	3	2	0	Chinese	
777	7/12/2022	P2805	LWY	31	1	0	0	Chinese	
778	7/12/2022	P2806	NABAA	31	3	2	0	Malay	
779	7/12/2022	P2807	SVAR	38	3	2	0	Indian	
780	7/12/2022	P2812	ZBD	35	5	4	0	Malay	
781	7/12/2022	P2813	PMBMY	35	4	2	1	Malay	
782	7/12/2022	P2811	TAT	30	2	1	0	Indian	
790	8/12/2022	P2808	OML	35	1	0	0	Chinese	
791	8/12/2022	P2809	NBS	37	4	3	0	Malay	
792	8/12/2022	P2810	CJY	33	2	1	0	Chinese	
793	8/12/2022	P2823	PNAA	30	1	0	0	Indian	
794	8/12/2022	P2824	SSC	41	3	0	2	Chinese	
795	8/12/2022	P2826	LSJ	45	1	0	0	Chinese	
796	8/12/2022	P2825	RABAR	36	6	5	0	Malay	
797	8/12/2022	P2815	SANS	37	1	0	0	Others	Thai
798	8/12/2022	P2816	NBMR	31	2	1	0	Malay	
799	8/12/2022	P2817	HPY	32	1	0	0	Chinese	
802	9/12/2022	P2818	CMJ	37	3	1	1	Indian	
803	9/12/2022	P2819	AAPM	35	3	2	0	Indian	
806	12/12/2022	P2820	HSH	40	2	1	0	Chinese	
808	12/12/2022	P2821	NBJ	34	3	2	0	Malay	
809	12/12/2022	P2822	HAM	36	3	2	0	Indian	
810	12/12/2022	P2828	HZBARL	35	3	2	0	Malay	
811	12/12/2022	P2829	YBCY	39	10	7	2	Malay	

812	12/12/2022	P2830	MBK	31	3	1	1	Malay	
813	12/12/2022	P2831	SWBAW	35	5	0	4	Malay	
814	12/12/2022	P2832	ISBAA	30	3	2	0	Malay	
815	12/12/2022	P2833	LEBA	35	2	1	0	Malay	
816	13/12/2022	P2836	SBN	31	4	3	0	Malay	
817	13/12/2022	P2834	NABM	30	2	1	0	Malay	
818	13/12/2022	P2835	RBMR	36	2	1		Malay	
819	13/12/2022	P2840	NABR	32	3	2	0	Malay	
821	13/12/2022	P2837	SAP	34	2	1	0	Indian	
822	13/12/2022	P2838	NBB	32	1	0	0	Malay	
824	13/12/2022	P2839	NNBS	34	4	2	1	Malay	
825	14/12/2022	P2827	NABM	33	3	2	0	Malay	
827	14/12/2022	P2841	NSBH	32	3	2	0	Malay	
828	14/12/2022	P2842	LMF	38	4	2	1	Chinese	
829	14/12/2022	P2843	SNEBS	30	2	1	0	Malay	
831	14/12/2022	P2844	ABAS	30	1	0	0	Malay	
832	14/12/2022	P2845	NBMG	30	1	0	0	Malay	
833	14/12/2022	P2846	ENAD	39	3	2	0	Others	Iban
834	15/12/2022	P2848	RABR	35	1	0	0	Malay	
836	15/12/2022	P2847	SBMS	31	2	0	1	Malay	
837	15/12/2022	P2849	SBA	30	2	1	0	Malay	
838	15/12/2022	P2850	RAV	37	3	1	1	Indian	
839	15/12/2022	P2851	NIBI	36	5	3	1	Malay	
840	15/12/2022	P2852	FBY	35	4	3	0	Malay	
842	15/12/2022	P2853	TTP	36	1	0	0	Chinese	
843	15/12/2022	P2854	LCW	36	2	1	0	Chinese	
844	15/12/2022	P2855	ABA	32	1	0	0	Malay	
845	15/12/2022	P2856	SSBMT	32	4	3	0	Malay	
849	16/12/2022	P2857	TMBTS	39	9	2	6	Malay	
850	19/12/2022	P2858	ABH	37	4	3	0	Malay	
851	19/12/2022	P2859	NDBMA	41	4	3	0	Malay	
853	19/12/2022	P2860	NHBA	32	3	1	1	Malay	
854	19/12/2022	P2861	FFBZ	36	4	3	0	Malay	

855	19/12/2022	P2862	MLM	38	6	2	3	Chinese
856	19/12/2022	P2863	NABN	37	4	2	1	Malay
857	19/12/2022	P2865	GAR	32	4	2	1	Indian
858	20/12/2022	P2864	NSBS	31	3	2	0	Malay
859	20/12/2022	P2866	SHBS	34	3	2	0	Malay
860	20/12/2022	P2870	NABAH	36	1	0	0	Malay
861	20/12/2022	P2868	NHBS	35	4	3	0	Malay
862	20/12/2022	P2867	ANAD	34	2	1	0	Chinese
863	20/12/2022	P2869	KSY	40	3	2	0	Chinese
864	20/12/2022	P2871	NABA	38	7	2	4	Malay
865	20/12/2022	P2872	NJBZ	34	2	1	0	Malay
868	3/1/2023	P2873	PYF	32	1	0	0	Chinese
871	3/1/2023	P2874	CYY	37	3	1	1	Chinese
872	3/1/2023	P2875	NFBI	35	4	2	1	Malay
874	3/1/2023	P2876	KSL	30	1	0	0	Chinese
875	3/1/2023	P2877	RAA	31	1	0	0	Indian
876	4/1/2023	P2878	SSBM	41	3	2	0	Malay
878	4/1/2023	P2879	NABB	37	7	3	3	Malay
879	4/1/2023	P2892	RBM	35	2	1	0	Malay
880	4/1/2023	P2891	NBN	35	4	2	1	Malay
881	4/1/2023	P2890	NFBMK	31	1	0	0	Malay
882	4/1/2023	P2889	NSBM	32	4	3	0	Malay
883	4/1/2023	P2880	ABAS	40	5	4	0	Malay
884	4/1/2023	P2881	HWF	38	2	1	0	Chinese
886	5/1/2023	P2882	SBT	32	4	3	0	Malay
887	5/1/2023	P2883	TYT	37	1	0		Chinese
888	5/1/2023	P2884	LER	33	1	0	0	Chinese
890	5/1/2023	P2885	TMBR	39	5	3	1	Malay
891	5/1/2023	P2886	WSC	34	3	2	0	Chinese
892	5/1/2023	P2887	NABB	41	2	1	0	Malay
893	5/1/2023	P2888	VAR	36	2	1	0	Indian
894	5/1/2023	P2893	FNNBD	30	3	1	1	Malay
895	5/1/2023	P2894	ABH	30	2	1	0	Malay

898	6/1/2023	P2895	VAS	31	1	0	0	Indian
899	9/1/2023	P2898	SMBSABAM	32	2	1	0	Malay
900	9/1/2023	P2899	SNBK	40	4	3	0	Malay
901	9/1/2023	P2897	NNBNA	32	4	2	1	Malay
902	9/1/2023	P2900	MBMA	39	1	0		Malay
903	9/1/2023	P2901	ZBI	32	2	1	0	Malay
904	9/1/2023	P2902	UADBU	40	7	4	2	Malay
905	9/1/2023	P2903	JBMJ	34	4	3	0	Malay
906	9/1/2023	P2904	KAS	36	1	0	0	Indian
907	9/1/2023	P2905	NBH	34	5	4	0	Malay
908	9/1/2023	P2915	NMBY	37	5	3	1	Malay
913	11/1/2023	P2916	NSBAOA	32	3	0	2	Malay
914	11/1/2023	P2917	NBJ	34	2	1	0	Malay
915	11/1/2023	P2918	LDD	32	1	0	0	Chinese
917	11/1/2023	P2911	NSABA	31	1	0	0	Malay
918	11/1/2023	P2912	WBC	40	3	1	1	Chinese
919	11/1/2023	P2913	CE	37	4	2	1	Chinese
922	12/1/2023	P2914	NAP	32	1	0		Indian
923	12/1/2023	P2906	NBZ	32	2	1	0	Malay
924	12/1/2023	P2907	SAT	30	1	0	0	Indian
926	12/1/2023	P2908	KAR	31	2	1	0	Indian
927	12/1/2023	P2909	NSBS	34	6	5	0	Malay
928	12/1/2023	P2910	NBZ	31	2	1	0	Malay
929	12/1/2023	P2919	MAP	36	2	1		Indian
930	12/1/2023	P2920	KAK	30	3	1	1	Indian
931	12/1/2023	P2921	FBO	32	4	2	1	Malay
932	13/1/2023	P2896	NSBI	37	4	2	1	Malay
933	13/1/2023	P2922	SABA	32	2	1		Malay
934	13/1/2023	P2923	NBAR	32	2	1	0	Malay
936	16/1/2023	P2924	SBB	41	3	2	0	Malay
937	16/1/2023	P2925	NBM	31	3	1	1	Malay
938	16/1/2023	P2926	NABM	30	4	2	1	Malay
941	16/1/2023	P2927	PATC	31	1	0		Indian

942	16/1/2023	P2928	SLSW	31	3	1	1	Chinese
943	16/1/2023	P2929	KBHM	35	2	1	0	Malay
944	16/1/2023	P2930	TCY	35	2	1	0	Chinese
945	16/1/2023	P2931	LPY	34	3	1	1	Chinese
946	17/1/2023	P2932	SABAR	30	2	1	0	Malay
947	17/1/2023	P2933	NABAB	35	5	4	0	Malay
948	17/1/2023	P2934	SBH	40	2	1		Malay
950	17/1/2023	P2935	SAS	35	6	3	2	Indian
951	17/1/2023	P2936	WYY	31	1	0	0	Chinese
952	17/1/2023	P2937	NBY	34	1	0		Malay
953	17/1/2023	P2939	RBAW	36	3	2		Malay
954	17/1/2023	P2938	ZBS	34	3	2		Malay
956	18/1/2023	P2940	NLBN	32	1	0	0	Malay
958	18/1/2023	P2941	LPX	35	2	0	1	Chinese
960	18/1/2023	P2942	NABA	32	4	1	2	Malay
961	18/1/2023	P2943	LCR	34	2	1	0	Chinese
962	18/1/2023	P2944	CAK	45	2	0	1	Indian
963	18/1/2023	P2945	KHJ	30	1	0	0	Chinese
964	18/1/2023	P2946	VAK	32	3	1	1	Indian
967	19/1/2023	P2947	HSY	36	1	0		Chinese
968	19/1/2023	P2948	CHY	32	1	0		Chinese
969	19/1/2023	P2949	NBN	32	3	1	1	Malay
972	19/1/2023	P2950	SFBSA	41	6	4	1	Malay
973	19/1/2023	P2951	HJJ	31	1	0		Chinese
974	19/1/2023	P2952	NABY	32	2	1	0	Malay
975	19/1/2023	P2953	NBM	39	5	3	1	Malay
976	19/1/2023	P2954	LAR	31	1	0		Indian
977	19/1/2023	P2955	DAM	30	1	0		Indian
978	19/1/2023	P2956	HPF	31	4	2	2	Chinese
981	25/1/2023	P2957	SAAV	32	2	1	0	Indian
983	25/1/2023	P2958	NABR	31	3	2	0	Malay
984	25/1/2023	P2959	NABZ	34	5	3	0	Malay
985	25/1/2023	P2960	YS	34	5	2	2	Chinese

986	25/1/2023	P2961	NABA	32	1	0	0	Malay	
987	25/1/2023	P2962	TAM	35	3	3	0	Indian	
988	26/1/2023	P2963	OJM	30	1	0		Chinese	
989	26/1/2023	P2964	NBB	40	3	2	0	Malay	
990	26/1/2023	P2965	GAY	42	3	2		Chinese	
991	26/1/2023	P2966	SKAKS	32	1	0		Indian	
992	26/1/2023	P2967	TST	31	1	0	0	Chinese	
993	26/1/2023	P2968	NABJ	36	2	1		Malay	
994	26/1/2023	P2969	ABO	38	5	5	0	Malay	
995	26/1/2023	P2970	VAC	34	2	1		Indian	
997	27/1/2023	P2971	AMBR	30	4	1	2	Malay	
1001	30/1/2023	P2972	IAJ	41	4	3		Others	Iban
1003	30/1/2023	P2973	AMBI	39	6	4	1	Malay	
1004	30/1/2023	P2974	NNBN	32	2	1		Malay	
1005	30/1/2023	P2980	PMY	31	2	0	1	Chinese	
1007	30/1/2023	P2981	WNBS	37	5	5		Malay	
1008	30/1/2023	P2982	TLM	36	3	2	0	Chinese	
1009	31/1/2023	P2983	NHBA	36	5	4		Malay	
1010	31/1/2023	P2975	JLMT	31	3	2	0	Chinese	
1011	31/1/2023	P2976	NABSM	33	3	2	0	Malay	
1012	31/1/2023	P2977	LAK	30	3	2	0	Indian	
1013	31/1/2023	P2978	NLABM	32	6	3	2	Malay	
1015	31/1/2023	P2979	IDBMF	32	1	0	0	Malay	
1016	31/1/2023	P2993	SSF	39	1	0	0	Chinese	
1017	31/1/2023	P2994	KAS	30	2	0	1	Indian	
1021	2/2/2023	P2995	NNBF	36	2	1	0	Malay	
1022	2/2/2023	P2996	JYIJ	30	1	0		Chinese	
1023	2/2/2023	P2984	TYL	36	1	0		Chinese	
1027	2/2/2023	P2985	NFBZ	32	2	0	1	Malay	
1028	2/2/2023	P2986	ABM	37	6	4	1	Malay	
1029	2/2/2023	P2987	LYY	40	1	0	0	Chinese	
1031	3/2/2023	P2988	SPH	34	1	0		Chinese	
1032	3/2/2023	P2989	AAGRS	38	1	0	0	Indian	

1033	3/2/2023	P2990	FHBK	32	2	1	0	Malay
1034	3/2/2023	P2991	AAH	39	3	2		Chinese
1035	7/2/2023	P2992	NLBR	32	2	1	0	Malay
1037	7/2/2023	P2998	NYBMY	31	1	0	0	Malay
1038	7/2/2023	P2997	TYP	35	2	1	0	Chinese
1039	7/2/2023	P3005	LMY	30	2	1	0	Chinese
1044	7/2/2023	P3004	AMBMJ	37	1	0		Malay
1045	7/2/2023	P3000	SNBSH	39	4	2	1	Malay
1046	7/2/2023	P3002	NBMN	34	5	3	1	Malay
1047	7/2/2023	P3003	LPC	34	1	0	0	Chinese
1048	7/2/2023	P3001	NBN	38	3	1	1	Malay
1049	7/2/2023	P3008	WFBS	34	1	0	0	Malay
1050	7/2/2023	P3009	SABS	40	4	3	0	Malay
1051	8/2/2023	P3007	NSBAN	32	1	0	0	Malay
1052	8/2/2023	P3006	NNABNII	39	1	0	0	Malay
1053	8/2/2023	P3028	NFBM	35	4	3	0	Malay
1055	8/2/2023	P3034	TWW	36	1	0	0	Chinese
1056	8/2/2023	P3032	NSBZ	34	5	3	1	Malay
1057	8/2/2023	P3035	PATK	32	3	2	0	Indian
1058	8/2/2023	P3033	KSK	39	2	0	1	Chinese
1059	8/2/2023	P3031	TYS	41	3	2	0	Chinese
1060	8/2/2023	P3023	JAA	32	3	2		Indian
1063	8/2/2023	P3024	NABI	35	3	1	1	Malay
1066	8/2/2023	P3025	SIBMZA	34	4	3	0	Malay
1067	8/2/2023	P3026	LYY	36	2	1	1	Chinese
1068	8/2/2023	P3030	CSH	42	1		0	Chinese
1069	9/2/2023	P3027	SAS	31	1	0	0	Indian
1070	9/2/2023	P3029	DBW	30	2	1	0	Malay
1071	9/2/2023	P3036	CYL	33	2	0	1	Chinese
1075	9/2/2023	P3039	NBMY	34	4	3	0	Malay
1076	9/2/2023	P3040	RABS	33	1	0	0	Malay
1077	9/2/2023	P3041	CCX	33	2	0	1	Chinese
1078	9/2/2023	P3042	NBMA	30	1	0	0	Malay

1079	9/2/2023	P3043	HBH	35	4	2	1	Malay
1080	9/2/2023	P3044	ANBMR	31	2	1		Malay
1081	9/2/2023	P3045	NBA	31	3	1	1	Malay
1086	10/2/2023	P3047	KSR	35	3	1	1	Chinese
1087	10/2/2023	P3048	DAJ	30	1	0	0	Indian
1088	13/2/2023	P3084	NBAM	32	2	1	0	Malay
1089	13/2/2023	P3083	LHBAW	41	7	4	1	Malay
1090	13/2/2023	P3086	SABI	30	3	2		Malay
1091	13/2/2023	P3077	LSM	30	1	0		Chinese
1092	13/2/2023	P3076	RBMTC	30	4	2	1	Malay
1093	13/2/2023	P3074	KVAS	32	2	1	0	Indian
1094	13/2/2023	P3078	MST	37	2	0	1	Chinese
1095	13/2/2023	P3075	NFBAL	30	3	1	1	Malay
1096	13/2/2023	P3085	NKBLAA	40	3	2	0	Malay
1097	13/2/2023	P3079	MAPP	32	1	0	0	Indian
1098	13/2/2023	P3081	NBBK	30	3	1	1	Malay
1099	13/2/2023	P3080	ABA	32	2	1		Malay
1101	13/2/2023	P3017	PAL	38	4	1	2	Indian
1102	13/2/2023	P3015	SAR	34	3	2	0	Indian
1103	13/2/2023	P3082	NHBY	38	7	4	2	Malay
1104	14/2/2023	P3014	NBSZ	32	2	1	0	Malay
1105	14/2/2023	P3016	LCL	31	1	0	0	Chinese
1109	14/2/2023	P3018	NNBAR	30	3	3	0	Malay
1110	14/2/2023	P3020	AKBZ	32	2	1	0	Malay
1111	14/2/2023	P3022	DAA	32	1	0	0	Indian
1115	15/2/2023	P3019	LYV	31	1	0	0	Chinese
1119	15/2/2023	P3021	NSBA	30	2	1		Malay
1120	15/2/2023	P3010	NBMS	31	1	0	0	Malay
1121	15/2/2023	P3011	LPS	32	1	0	0	Chinese
1122	15/2/2023	P3013	CPW	38	2	1		Chinese
1123	15/2/2023	P3057	NHBM	35	3	2	0	Malay
1124	15/2/2023	P3056	NSBJ	32	4	2	1	Malay
1125	15/2/2023	P3050	NBM	38	2	1	1	Malay

1126	15/2/2023	P3051	VLST	31	1	0	0	Chinese
1128	15/2/2023	P3052	AMBKJ	33	2	1	0	Malay
1130	16/2/2023	P3055	LCW	36	1		0	Chinese
1131	16/2/2023	P3054	NBN	32	4	2	1	Malay
1132	16/2/2023	P3059	FABS	31	3	2	0	Malay
1133	16/2/2023	P3053	NBY	35	1	0	0	Malay
1134	16/2/2023	P3060	FIBN	34	2	1	0	Malay
1135	16/2/2023	P3058	ZBJ	32	2	1	0	Malay
1136	16/2/2023	P3049	NFBR	31	1		0	Malay
1137	16/2/2023	P3068	IFBA	30	1	0	0	Malay
1138	16/2/2023	P3066	GAM	38	1		0	Indian
1139	16/2/2023	P3065	NFBMY	35	4	2	1	Malay
1141	16/2/2023	P3064	TKY	39	3	2	0	Chinese
1143	20/2/2023	P3062	PAS	30	1	0	0	Indian
1144	20/2/2023	P3067	NSBMY	32	4	3	0	Malay
1145	20/2/2023	P3063	OSC	35	1	0	0	Chinese
1146	20/2/2023	P3071	NABA	33	3	2	0	Malay
1147	20/2/2023	P3061	AFBA	32	1	0	0	Malay
1148	20/2/2023	P3073	NABMA	37	6	4	1	Malay
1149	20/2/2023	P3070	NBMA	34	2	1	0	Malay
1150	20/2/2023	P3072	FBA	42	8	6	1	Malay
1152	20/2/2023	P3123	LFM	30	4	2	1	Chinese
1153	20/2/2023	P3121	FKN	36	2	1	0	Chinese
1154	20/2/2023	P3120	SFBSO	32	2	1	0	Malay
1155	20/2/2023	P3119	NBA	31	2	1	0	Malay
1156	20/2/2023	P3122	LYW	35	2	1	0	Chinese
1157	20/2/2023	P3125	NSBS	33	3	2	0	Malay
1158	21/2/2023	P3134	NABWT	30	2	0	1	Malay
1159	21/2/2023	P3135	SMBJ	37	6	4	1	Malay
1160	21/2/2023	P3136	NPE	35	4	2	1	Chinese
1162	21/2/2023	P3133	TPF	36	3	2	0	Chinese
1163	21/2/2023	P3137	AABM	31	1	0	0	Malay
1165	21/2/2023	P3132	HBM	35	5	4	0	Malay

1166	21/2/2023	P3128	YCW	38	3	2		Chinese	
1167	21/2/2023	P3130	NABS	41	6	4	1	Malay	
1169	21/2/2023	P3129	HBA	31	2	1	0	Malay	
1170	21/2/2023	P3131	GTFBI	30	2	0	1	Malay	
1171	21/2/2023	P3124	SBM	33	3	2	0	Malay	
1173	21/2/2023	P3139	SNAN	37	1	0	0	Indian	
1178	22/2/2023	P3140	LKC	30	3	2	0	Chinese	
1179	22/2/2023	P3118	NAJBA	30	1	0	0	Malay	
1180	22/2/2023	P3138	ASAAJ	41	4	1	2	Indian	
1181	22/2/2023	P3165	QKL	35	4	2	1	Chinese	
1182	22/2/2023	P3154	NABT	30	3	2	0	Malay	
1183	22/2/2023	P3117	LXS	30	1	0	0	Chinese	
1184	22/2/2023	P3164	HATR	34	2	1	0	Indian	
1187	22/2/2023	P3162	NABH	34	3	2		Malay	
1188	22/2/2023	P3166	KAM	42	5	1	3	Indian	
1189	22/2/2023	P3161	NABAN	30	1	0	0	Malay	
1190	22/2/2023	P3160	NFBN	35	3	2	0	Malay	
1192	22/2/2023	P3115	NSBN	30	5	3	1	Malay	
1193	22/2/2023	P3158	NABMA	30	2	1	0	Malay	
1194	22/2/2023	P3114	NBJ	30	2	0	1	Malay	
1196	22/2/2023	P3116	ZBZ	33	3	2		Malay	
1198	23/2/2023	P3126	LXY	33	2	1	0	Chinese	
1199	23/2/2023	P3163	LSL	33	2	0	1	Chinese	
1200	23/2/2023	P3155	NFBB	38	1	0	0	Malay	
1204	23/2/2023	P3156	CPY	30	1	0	0	Chinese	
1207	23/2/2023	P3153	SNBA	31	5	2	2	Malay	
1208	23/2/2023	P3152	NBJ	36	6	3	2	Malay	
1210	23/2/2023	P3143	NBM	33	2	1	0	Malay	
1211	23/2/2023	P3145	SNABZ	32	3	2	0	Malay	
1212	23/2/2023	P3142	NBM	40	7	5	1	Malay	
1216	24/2/2023	P3159	SMP	36	2	1	0	Indian	
1217	24/2/2023	P3150	ABA	34	8	4	3	Malay	
1231	27/2/2023	P3069	SBK	34	4	2	1	Others	Dusun

1232	27/2/2023	P3141	ABO	31	1	0	0	Malay
1233	27/2/2023	P3147	CMT	30	3	1	1	Chinese
1237	27/2/2023	P3149	ZBM	36	4	3	0	Malay
1239	27/2/2023	P3144	NRBAH	33	1	0		Malay
1240	27/2/2023	P3157	NASBR	31	3	1	1	Malay
1247	28/2/2023	P3146	YWK	32	1	0		Chinese
1248	28/2/2023	P3151	LSM	30	2	0	1	Chinese
1249	28/2/2023	P3148	NBA	37	1	0	0	Malay
1250	28/2/2023	P3095	SNBMA	30	3	2	0	Malay
1257	28/2/2023	P3094	TMY	38	1	0	0	Chinese
1260	28/2/2023	P3093	NFBAB	37	3	2	0	Malay
1261	28/2/2023	P3090	NBY	32	7	2	4	Malay
1262	28/2/2023	P3092	NABM	35	2	1	0	Malay
1263	28/2/2023	P3091	NSBR	31	1	0	0	Malay
1264	28/2/2023	P3089	NSBZ	31	3	1	1	Malay
1265	28/2/2023	P3097	AZ	31	3	1	1	Chinese
1266	28/2/2023	P3100	SABMI	30	2	1	0	Malay
1267	28/2/2023	P3088	NLRBMA	34	5	3	1	Malay
1268	28/2/2023	P3096	CSY	34	1	0	0	Chinese
1269	28/2/2023	P3098	NAG	38	2	1	0	Indian
1277	1/3/2023	P3099	COYH	30	2	0	1	Chinese
1278	1/3/2023	P3101	BST	40	2	1	0	Chinese
1279	1/3/2023	P3102	NBS	38	2	1	0	Malay
1281	1/3/2023	P3104	NJSBA	30	1		0	Malay
1282	1/3/2023	P3113	SBA	38	3	1	1	Malay
1283	1/3/2023	P3109	ZRBZA	32	2	1	0	Malay
1284	1/3/2023	P3107	SSBNI	34	3	2	0	Malay
1285	1/3/2023	P3110	NBMS	30	1	0	0	Malay
1286	1/3/2023	P3103	TTT	41	1	0	0	Chinese
1287	1/3/2023	P3105	NBMN	33	3	2	0	Malay
1289	1/3/2023	P3111	NDBR	32	4	2	1	Malay
1290	1/3/2023	P3106	ZBZ	32	4	2	1	Malay
1291	1/3/2023	P3112	SVY	37	2	1		Chinese

1299	1/3/2023	P3108	SBMR	41	1	0	0	Malay
1306	2/3/2023	P3191	TAK	30	1	0	0	Indian
1307	2/3/2023	P3186	NIBMN	30	2	1	0	Malay
1308	2/3/2023	P3190	NARBN	30	1	0	0	Malay
1309	2/3/2023	P3189	HFBZ	38	6	4	1	Malay
1310	2/3/2023	P3208	NABK	30	3	2	0	Malay
1315	2/3/2023	P3184	SAC	31	3	2		Indian
1316	2/3/2023	P3183	NABMA	30	2	1	0	Malay
1319	2/3/2023	P3219	SAI	39	2	1		Indian
1320	2/3/2023	P3181	NABR	30	1	0	0	Malay
1321	2/3/2023	P3185	THC	32	2	0	1	Chinese
1322	2/3/2023	P3187	KMY	32	1	0	0	Chinese
1323	2/3/2023	P3193	NHBR	32	1	0	0	Malay
1324	2/3/2023	P3213	NHBH	35	3	1	1	Malay
1325	2/3/2023	P3211	NSBMN	41	3	2	0	Malay
1327	2/3/2023	P3188	NBIP	31	1	0	0	Malay
1329	2/3/2023	P3215	TYN	32	3	2	0	Chinese
1336	3/3/2023	P3192	NABI	37	3	2	0	Malay
1338	3/3/2023	P3178	CYY	36	2	1	0	Chinese
1339	3/3/2023	P3182	NABMS	33	4	2	1	Malay
1344	6/3/2023	P3218	NHBA	32	3	2	0	Malay
1345	6/3/2023	P3210	MNBM	31	1	0	0	Malay
1347	6/3/2023	P3216	RBAR	39	4	2	1	Malay
1349	6/3/2023	P3209	SNABS	31	4	3	0	Malay
1350	6/3/2023	P3207	SSBD	30	2	1	0	Malay
1353	6/3/2023	P3217	NKBMS	35	1	0	0	Malay
1354	6/3/2023	P3212	NHBAB	32	2	1	0	Malay
1355	6/3/2023	P3214	NBN	35	5	2	2	Malay
1358	6/3/2023	P3168	YAG	30	1	0		Indian
1359	6/3/2023	P3171	JUBU	37	5	0	3	Malay
1360	6/3/2023	P3180	SBAA	35	5	2	2	Malay
1362	7/3/2023	P3175	SIBA	37	4	3	0	Malay
1363	7/3/2023	P3221	CYM	38	4	3	0	Chinese

1364	7/3/2023	P3177	DMBJ	31	2	1	0	Malay
1367	7/3/2023	P3170	CMEW	31	2	1		Chinese
1368	7/3/2023	P3169	NFBM	32	4	3	0	Malay
1369	7/3/2023	P3174	NYZ	36	3	1	1	Chinese
1370	7/3/2023	P3179	WZY	38	1	0		Chinese
1372	7/3/2023	P3173	TPY	32	1	0		Chinese
1373	7/3/2023	P3172	NSBJ	38	3	2	0	Malay
1377	8/3/2023	P3225	LSW	33	1	0	0	Chinese
1378	8/3/2023	P3176	DNBAM	32	1	0	0	Malay
1383	8/3/2023	P3227	KAV	32	2	1		Indian
1384	8/3/2023	P3224	TAA	36	2	1	0	Indian
1390	8/3/2023	P3228	GAM	31	2	1	0	Indian
1391	8/3/2023	P3223	NBMN	36	3	2	0	Malay
1393	8/3/2023	P3226	SAMBSR	33	2	1	0	Malay
1400	9/3/2023	P3230	HBM	33	3	0	2	Malay
1402	9/3/2023	P3234	MBA	40	6	3	2	Malay
1403	9/3/2023	P3233	NAN	34	3	1	1	Indian
1404	9/3/2023	P3203	WSF	42	2	0	1	Chinese
1405	9/3/2023	P3232	GLM	35	1	0	0	Chinese
1411	9/3/2023	P3206	NBK	37	3	2	0	Malay
1412	9/3/2023	P3204	NSBM	32	3	2	0	Malay
1413	9/3/2023	P3201	PAJ	38	1	0	0	Indian
1418	9/3/2023	P3199	FBR	33	1	0	0	Malay
1419	9/3/2023	P3205	SRBAG	41	4	1	2	Malay
1423	10/3/2023	P3200	DAG	33	1	0		Malay
1425	10/3/2023	P3229	AABJ	31	3	2	1	Malay
1426	10/3/2023	P3202	CYY	35	3	2	0	Chinese
1430	13/3/2023	P3198	FY	34	1	0	0	Chinese
1431	13/3/2023	P3195	NIBNT	31	4	2	1	Malay
1437	13/3/2023	P3284	NMBZ	37	1	0		Malay
1438	13/3/2023	P3194	SFSBSF	31	1	0	0	Malay
1439	13/3/2023	P3196	JKCAS	42	3	1	1	Indian
1442	13/3/2023	P3197	NABJ	32	2	0	1	Malay

1443	13/3/2023	P3283	CAJ	35	2	1		Indian
1444	13/3/2023	P3285	LBH	40	1	0	0	Malay
1452	14/3/2023	P3282	WSY	39	3	2	0	Chinese
1453	14/3/2023	P3280	NABMS	38	3	2		Malay
1454	14/3/2023	P3281	WFNBWI	32	3	0	2	Malay
1455	14/3/2023	P3286	TSY	30	1	0	0	Chinese
1460	14/3/2023	P3240	SBMI	33	3	2	0	Malay
1461	14/3/2023	P3278	PYT	30	1	0	0	Chinese
1463	14/3/2023	P3276	THL	31	1	0	0	Chinese
1464	14/3/2023	P3241	RBAR	39	5	3	1	Malay
1467	14/3/2023	P3277	AKW	33	2	1	0	Chinese
1468	14/3/2023	P3239	HHBM	33	3	2	0	Malay
1469	14/3/2023	P3237	PIIBI	38	9	5	3	Malay
1470	14/3/2023	P3242	NBT	30	2	1	0	Malay
1473	15/3/2023	P3236	ATWL	35	2	1	0	Chinese
1474	15/3/2023	P3238	NHBM	30	1	0	0	Malay
1478	20/3/2023	P3308	NFBA	35	3	2	0	Malay
1481	20/3/2023	P3244	NHBH	35	4	1	2	Malay
1482	20/3/2023	P3243	SNBAN	31	4	0	1	Malay
1483	20/3/2023	P3307	NSQBMN	30	3	2	0	Malay
1486	20/3/2023	P3246	NZBH	30	1	0	0	Malay
1488	20/3/2023	P3305	ABMN	30	4	2	1	Malay
1489	20/3/2023	P3247	NBA	37	4	3	0	Malay
1491	20/3/2023	P3306	AFBAA	38	2	1	0	Malay
1492	20/3/2023	P3304	SNBAH	38	2	1	0	Malay
1493	20/3/2023	P3245	AFBA	34	2	1	0	Malay
1494	20/3/2023	P3310	SAS	41	3	2	0	Indian
1498	21/3/2023	P3302	NLT	34	1	0	0	Chinese
1499	21/3/2023	P3303	MAP	32	1	0	0	Indian
1501	21/3/2023	P3235	NHX	30	1	0	0	Chinese
1502	21/3/2023	P3370	NBMD	39	5	4		Chinese
1504	21/3/2023	P3301	TAM	33	2	1	0	Indian
1505	21/3/2023	p3369	TCT	34	2	1	0	Chinese

1506	21/3/2023	P3300	LABMK	40	3	2	0	Malay	
1508	21/3/2023	P3368	NALBAK	33	2	1	0	Malay	
1509	21/3/2023	P3371	YKH	32	2	0	0	Chinese	
1510	22/3/2023	P3309	ZBB	30	1	0	0	Malay	
1511	22/3/2023	P3365	HBMH	37	1	0	0	Malay	
1514	22/3/2023	P3366	JAVK	30	1	0	0	Indian	
1517	22/3/2023	P3312	RABAZ	32	2	1	0	Malay	
1518	22/3/2023	P3374	NABMS	32	3	2	0	Malay	
1521	22/3/2023	P3372	NABCH	31	2	1	0	Malay	
1524	22/3/2023	P3373	NBR	37	6	2	3	Malay	
1525	22/3/2023	P3367	AAS	34	2	1	0	Indian	
1526	22/3/2023	P3316	KAT	31	1	0	0	Indian	
1527	22/3/2023	P3319	LMF	34	1	0	0	Chinese	
1528	22/3/2023	P3314	NALBNAF	33	3	1	1	Malay	
1531	22/3/2023	P3313	SBS	37	4		3	Malay	
1532	22/3/2023	P3315	LCY	35	2	1	0	Chinese	
1533	23/3/2023	P3251	RBMK	33	4	3	0	Malay	
1534	23/3/2023	P3378	HMM	33	1	0	0	Chinese	
1535	23/3/2023	P3254	NBA	32	3	2	0	Malay	
1536	23/3/2023	P3249	KSF	35	1	0	0	Chinese	
1537	23/3/2023	P3257	YAA	32	1	0	0	Indian	
1538	23/3/2023	P3325	NLY	37	4	3	0	Chinese	
1539	23/3/2023	P3248	NABS	37	2	1	0	Malay	
1540	23/3/2023	P3255	NSBR	31	1	0	0	Malay	
1542	23/3/2023	P3317	EAP	36	1	0	0	Indian	
1543	23/3/2023	P3250	NPAR	30	1	0	0	Indian	
1544	23/3/2023	P3252	NNBR	30	2	1	0	Malay	
1546	23/3/2023	P3376	LPY	41	1	0	0	Chinese	
1547	23/3/2023	P3253	NKBAM	34	2	1	0	Malay	
1551	23/3/2023	P3311	RBMR	34	2	0	1	Malay	
1552	23/3/2023	P3320	FG	31	1	0	0	Others	Kadazan
1556	27/3/2023	P3324	MIBMN	32	3	2	0	Malay	
1560	27/3/2023	P3321	SNABA	33	3	2	0	Malay	

1563	27/3/2023	P3375	SHBAA	32	2	1	0	Malay
1565	27/3/2023	P3318	HCY	30	1	0	0	Chinese
1567	28/3/2023	P3322	KKAHS	34	1	0	0	Indian
1568	28/3/2023	P3360	NBI	37	3	2	1	Malay
1569	28/3/2023	P3323	LPL	38	2	1	0	Chinese
1571	28/3/2023	P3352	LJW	32	3	1	1	Chinese
1572	28/3/2023	P3354	SSH	33	4	3	0	Chinese
1577	28/3/2023	P3357	NSBA	31	2	1	0	Malay
1579	28/3/2023	P3256	SHBI	36	3	2	0	Malay
1580	28/3/2023	P3358	NHBAK	33	4	3	0	Malay
1581	28/3/2023	P3359	NYW	37	1	0	0	Chinese
1582	28/3/2023	P3355	LYC	35	3	2	0	Chinese
1587	29/3/2023	P3361	FYJ	33	1	0	0	Chinese
1588	29/3/2023	P3364	NBA	30	1	0	0	Malay
1589	29/3/2023	P3363	SHBA	39	4	2	1	Malay
1590	29/3/2023	P3353	NBH	35	5	3	1	Malay
1591	29/3/2023	P3331	SNSBMR	30	1	0	0	Malay
1592	29/3/2023	P3362	LHY	30	1	0	0	Chinese
1593	29/3/2023	P3334	NBAR	30	2	1	0	Malay
1595	29/3/2023	P3328	NHBI	40	4	3	0	Malay
1598	29/3/2023	P3356	BKY	34	3	1	1	Chinese
1600	30/3/2023	P3333	WSW	30	1	0	0	Chinese
1601	30/3/2023	P3336	SFHBW	31	1	0	0	Malay
1602	30/3/2023	P3327	ASH	39	1	0	0	Chinese
1608	30/3/2023	P3335	NBH	30	2	1	0	Malay
1610	30/3/2023	P3329	NBMI	44	5	3	1	Malay
1611	30/3/2023	P3337	TYN	32	2	0	1	Chinese
1612	30/3/2023	P3338	ESBAS	42	6	3	2	Malay
1614	30/3/2023	P3330	NABS	31	2	1		Malay
1615	30/3/2023	P3341	KPL	35	1	0	0	Chinese
1616	30/3/2023	P3340	SNBN	33	3	1	1	Malay
1619	31/3/2023	P3332	SNWBA	32	4	1	2	Malay
1622	3/4/2023	P3339	TFM	30	1	0		Chinese

1624	3/4/2023	P3348	JBJ	41	5	2	2	Malay
1625	3/4/2023	P3326	ABMA	30	2	1	0	Malay
1628	3/4/2023	P3350	SAG	34	2	1	0	Indian
1630	3/4/2023	P3346	VAP	33	1	0	0	Indian
1635	3/4/2023	P3342	PAS	30	2	0	1	Indian
1640	3/4/2023	P3347	NHBMF	31	3	1	1	Malay
1643	4/4/2023	P3344	WLK	30	1	0	0	Chinese
1645	4/4/2023	P3351	KKY	34	2	1	0	Chinese
1646	4/4/2023	P3343	GVF	33	1	0	0	Chinese
1648	4/4/2023	P3345	SDAJ	32	2	1	0	Indian
1649	4/4/2023	P3389	RAA	36	4	1	2	Indian
1650	4/4/2023	P3388	LAA	38	4	3	0	Indian
1651	4/4/2023	P3391	RBS	30	2	1	0	Malay
1652	4/4/2023	P3390	NSBB	42	9	7	1	Malay
1657	5/4/2023	P3385	SZBB	32	1	0	0	Malay
1660	5/4/2023	P3387	TSE	30	1	0	0	Chinese
1662	5/4/2023	P3392	NBMA	34	1	0	0	Malay
1663	5/4/2023	P3383	PNBAH	35	5	2	2	Malay
1664	5/4/2023	P3394	CSH	34	2	1	0	Chinese
1668	5/4/2023	P3393	NABAA	36	4	2	1	Malay
1669	5/4/2023	P3379	HBH	36	9	8	0	Malay
1670	5/4/2023	P3384	LAL	39	3	2	0	Chinese
1673	6/4/2023	P3386	NABAR	35	2	1	0	Malay
1674	6/4/2023	P3395	NBR	35	3	2	0	Malay
1680	6/4/2023	P3382	IJBM	31	2	1	0	Malay
1682	6/4/2023	P3398	SSBI	34	1	0	0	Malay
1683	6/4/2023	P3380	AABMR	36	3	2	0	Malay
1685	6/4/2023	P3397	KAS	41	1	0	0	Indian
1688	6/4/2023	P3399	NIBA	30	2	1	0	Malay
1689	6/4/2023	P3396	LM	32	1	0	0	Chinese
1690	6/4/2023	P3404	SBS	30	4	2	1	Malay
1691	6/4/2023	P3400	TCS	41	2	1	0	Chinese
1692	6/4/2023	P3401	MSBA	30	5	2	2	Malay

1693	6/4/2023	P3402	FBMY	35	3	2		Malay
1694	6/4/2023	P3403	NBS	39	7	3	3	Malay
1696	7/4/2023	P3409	HBH	37	1	0	0	Malay
1697	7/4/2023	P3411	NFBM	30	2	1	0	Malay
1698	7/4/2023	P3410	JWYS	35	1	0	0	Chinese
1701	10/4/2023	P3406	NBY	41	3	2	0	Malay
1707	10/4/2023	P3413	NABMY	34	2	1	0	Malay
1708	10/4/2023	P3381	NBI	33	3	2	0	Malay
1712	10/4/2023	P3412	NABA	31	3	2	0	Malay
1715	10/4/2023	p3405	MEBA	32	5	1	3	Malay
1716	10/4/2023	p3414	CSY	34	1	0		Chinese
1717	11/4/2023	P3407	FSBD	34	2	0	1	Malay
1718	11/4/2023	P3415	RBAR	38	1	0	0	Malay
1719	11/4/2023	P3408	LST	33	1	0	0	Chinese
1720	11/4/2023	P3417	ENHBE	30	2	1	0	Malay
1721	11/4/2023	P3428	FSBR	32	2	1	0	Malay
1722	11/4/2023	P3416	NYBM	32	3	1	1	Malay
1723	11/4/2023	P3426	GHL	33	1	0	0	Chinese
1727	11/4/2023	P3429	GSL	37	1	0	0	Chinese
1729	11/4/2023	P3430	CMM	33	3	2	0	Chinese
1730	11/4/2023	P3427	SMBMD	32	2	1	0	Malay
1732	11/4/2023	P3424	SSHM	32	1	0	0	Chinese
1734	11/4/2023	P3418	LSM	33	1	0	0	Chinese
1736	11/4/2023	P3425	NFLBAR	33	3	2	0	Malay
1738	12/4/2023	P3423	NSBR	38	2	1	0	Malay
1739	12/4/2023	P3422	SRBS	40	8	6	1	Malay
1744	12/4/2023	P3449	NBA	34	3	0	2	Malay
1747	12/4/2023	P3420	NBAH	31	2	1		Malay
1748	12/4/2023	P3419	SNBS	30	2	1	0	Malay
1750	12/4/2023	P3456	SSRBS	44	10	6	3	Malay
1751	12/4/2023	P3451	NBMH	35	4	3	0	Malay
1752	12/4/2023	P3421	NBR	30	1	0	0	Malay
1753	12/4/2023	P3454	YAG	34	3	1	1	Indian

1754	12/4/2023	P3455	LBM	40	4	3		Malay	
1755	12/4/2023	P3445	ZBCA	33	3	2	0	Malay	
1758	12/4/2023	P3453	NBAG	35	3	2	0	Malay	
1759	12/4/2023	P3444	LSF	36	5	3	1	Chinese	
1760	12/4/2023	P3452	SSBA	33	4	2	1	Malay	
1764	13/4/2023	P3446	NABAB	36	3	2	0	Malay	
1765	13/4/2023	P3459	NABA	37	1	0	0	Malay	
1767	13/4/2023	P3447	JP	35	2	1		Others	Murut
1768	13/4/2023	P3448	NBY	30	1	0	0	Malay	
1769	13/4/2023	P3458	SWZBWM	41	6	5	0	Malay	
1770	13/4/2023	P3457	NAO	32	3	2	0	Indian	
1774	13/4/2023	P3462	MBS	33	4	2	1	Malay	
1776	13/4/2023	P3464	NBI	30	1	0	0	Malay	
1778	13/4/2023	P3450	NKBMJ	37	3	2	0	Malay	
1779	13/4/2023	P3467	JSAA	31	2	1	0	Indian	
1780	13/4/2023	P3468	NABR	33	4	3	0	Malay	
1784	14/4/2023	P3461	YPF	34	1	0	0	Chinese	
1785	14/4/2023	P3460	PAN	33	1	0	0	Indian	
1786	14/4/2023	P3463	SSBMN	33	5	3	1	Malay	
1788	17/4/2023	P3466	CMZ	32	6	1	4	Chinese	
1789	17/4/2023	P3465	OJJ	30	1	0	0	Chinese	
1791	17/4/2023	P3500	TLC	34	1	0	0	Chinese	
1793	17/4/2023	P3498	LSW	37	3	2	0	Chinese	
1794	17/4/2023	P3499	SNBSO	30	2	0	1	Malay	
1797	18/4/2023	P3497	LGS	39	2	1	0	Chinese	
1798	18/4/2023	P3496	LSW	32	1	0	0	Chinese	
1799	18/4/2023	P3507	SMAG	30	2	1	0	Indian	
1802	18/4/2023	P3508	NSBZZ	35	4	2	1	Malay	
1803	18/4/2023	p3503	CNS	34	1	0	0	Indian	
1804	18/4/2023	P3502	IABAN	33	1	0	0	Malay	
1805	19/4/2023	P3510	UKBY	34	3	2	0	Malay	
1806	19/4/2023	P3501	HAS	30	2	0	1	Indian	
1807	19/4/2023	P3514	NBMS	32	3	2	0	Malay	

1808	19/4/2023	P3509	NLS	36	2	1	0	Chinese
1812	19/4/2023	P3506	KSY	34	3	1	1	Chinese
1813	19/4/2023	P3505	CPM	37	3	1	1	Chinese
1814	19/4/2023	P3512	FBI	43	2	1	0	Malay
1815	19/4/2023	P3511	NABCS	37	4	2	1	Malay
1816	19/4/2023	P3504	LCW	38	3	1	1	Chinese
1820	19/4/2023	P3515	NWM	36	2	1	0	Chinese
1822	20/4/2023	P3516	SADB	32	2	0	1	Indian
1823	20/4/2023	P3518	SBAB	32	2	1	0	Malay
1824	20/4/2023	P3517	CAS	38	3	2	0	Indian
1828	20/4/2023	P3519	ELSY	33	1	0	0	Chinese
1830	20/4/2023	P3520	NBA	38	2	0	1	Malay
1832	25/4/2023	P3521	TYL	37	1	0	0	Chinese
1836	25/4/2023	P3513	NABMA	30	4	1	2	Malay
1838	26/4/2023	P3538	MBA	38	5	4	0	Malay
1839	26/4/2023	P3539	SHBZ	42	8	5	2	Malay
1840	26/4/2023	P3540	SSMBN	30	4	2	1	Malay
1841	26/4/2023	P3541	MTSY	31	1	0	0	Chinese
1842	27/4/2023	P3535	KAA	31	1	0	0	Indian
1843	27/4/2023	P3536	NBR	39	5	3	1	Malay
1844	27/4/2023	P3537	NJBS	32	3	2	0	Malay
1845	27/4/2023	P3542	NABM	32	3	2	0	Malay
1850	8/5/2023	P3543	NSBMS	30	2	0	1	Malay
1851	8/5/2023	P3544	YLP	38	3	1	1	Chinese
1852	8/5/2023	P3545	KSL	32	2	1	0	Chinese
1853	8/5/2023	P3546	NJBMK	34	3	2	0	Malay
1854	8/5/2023	P3547	SNBMA	34	4	2	2	Malay
1857	8/5/2023	P3528	NSBMN	36	3	0	2	Malay
1858	8/5/2023	P3527	NINBR	31	2	1	0	Malay
1863	9/5/2023	P3531	LPY	34	1	0		Chinese
1867	9/5/2023	P3526	FBK	34	4	2	1	Malay
1868	9/5/2023	P3530	AHBA	34	4	3		Malay
1869	9/5/2023	P3522	HYT	38	4	1	2	Chinese

1872	9/5/2023	P3524	DLBMR	36	1	0		Malay
1873	9/5/2023	P3529	CJY	31	3	1	1	Chinese
1874	9/5/2023	P3523	LAAL	38	3	3	0	Indian
1876	9/5/2023	P3534	FBMS	31	3	1	1	Malay
1878	10/5/2023	P3525	HBI	34	3	2	0	Malay
1879	10/5/2023	P3532	NBMZ	35	3	2	0	Malay
1880	10/5/2023	P3556	NNFBA	32	1	0	0	Malay
1882	10/5/2023	P3533	RBR	35	4	3		Malay
1886	10/5/2023	P3560	SBC	33	2	1		Chinese
1888	10/5/2023	P3555	NIBW	32	3	2	0	Malay
1889	10/5/2023	P3559	NIBA	35	3	2	0	Malay
1895	11/5/2023	P3558	NFBAJ	30	2	0	1	Malay
1896	11/5/2023	P3552	SBY	33	4	3		Malay
1897	11/5/2023	P3557	MBS	31	3	2	0	Malay
1898	11/5/2023	P3551	LLC	33	1	0		Chinese
1899	11/5/2023	P3554	TAR	31	1	0		Indian
1904	11/5/2023	P3548	WNFBWR	33	1	0	0	Malay
1907	11/5/2023	P3553	LBSH	35	5	1	3	Malay
1908	11/5/2023	P3549	NFBMN	39	5	3	1	Malay
1912	15/5/2023	P3489	SHF	39	2	0	1	Chinese
1913	15/5/2023	p3550	NBH	30	2	1	0	Malay
1915	15/5/2023	P3494	SSBWN	38	6	1	4	Malay
1918	15/5/2023	P3491	ABI	38	3	2	0	Malay
1919	15/5/2023	P3492	NHABAM	32	1	0		Malay
1920	15/5/2023	P3490	APAJP	33	1	0	0	Indian
1922	15/5/2023	P3493	MBMP	39	6	4	1	Malay
1923	15/5/2023	P3495	NZBH	33	4	4	0	Malay
1926	16/5/2023	P3487	MNBMI	34	4	3	0	Malay
1927	16/5/2023	P3485	NABMF	33	5	2	2	Malay
1931	16/5/2023	P3486	MSK	38	5	2	2	Chinese
1932	16/5/2023	p3484	NBA	40	4	2	1	Malay
1933	16/5/2023	P3488	BAY	37	1	0	0	Indian
1936	17/5/2023	P3483	ZBZA	33	4	3	0	Malay

1937	17/5/2023	P3568	TAR	33	4	1	2	Indian
1938	17/5/2023	P3563	NBMA	31	2	1	0	Malay
1939	17/5/2023	P3569	NBR	37	5	3	1	Malay
1941	17/5/2023	P3561	SBK	33	3	2	0	Malay
1942	17/5/2023	P3562	LWK	40	4	2	1	Chinese
1943	17/5/2023	P3567	KBS	37	5	3	1	Malay
1944	17/5/2023	P3565	ANBCR	32	1	0	0	Malay
1945	17/5/2023	P3564	SNABCDA	35	4	2	1	Malay
1951	18/5/2023	P3571	NSBZ	32	3	1	1	Malay
1952	18/5/2023	P3566	NBA	42	1	0		Malay
1953	18/5/2023	P3573	LPC	35	3	1	1	Chinese
1956	18/5/2023	p3482	TLT	37	2	0	1	Chinese
1957	18/5/2023	P3572	TAK	32	1	0	0	Indian
1958	18/5/2023	P3570	NLBMS	37	3	2	0	Malay
1960	18/5/2023	P3481	NFBH	31	2	1	0	Malay
1962	18/5/2023	P3479	KBA	37	5	2	1	Malay
1963	18/5/2023	P3478	OPN	32	1	0		Chinese
1964	18/5/2023	P3480	GYY	36	1	0	0	Chinese
1965	18/5/2023	P3475	HAK	35	1	0	0	Indian
1969	18/5/2023	P3474	NHBJ	38	2	0	1	Malay
1971	19/5/2023	P3473	VAJ	36	5	2	2	Indian
1973	22/5/2023	P3472	RAN	30	2	1	0	Indian
1974	22/5/2023	P3471	LKY	32	1	0		Chinese
1976	22/5/2023	P3476	HBHS	35	1	0	0	Malay
1977	22/5/2023	P3470	SDAB	41	3	2	0	Indian
1979	22/5/2023	P3477	SMBMA	35	2	1	0	Malay
1980	22/5/2023	P3586	IDBMS	35	4	3	0	Malay
1981	22/5/2023	P3582	NABR	36	3	2	0	Malay
1982	22/5/2023	P3581	NSBS	38	7	4	2	Malay
1984	22/5/2023	P3583	SBS	39	6	3	2	Malay
1985	22/5/2023	P3584	JJAR	38	2	1	0	Indian
1986	22/5/2023	P3579	NABAT	33	3	2	0	Malay
1988	23/5/2023	P3575	NABMN	32	2	1	0	Malay

1990	23/5/2023	P3574	GSC	40	1	0		Chinese
1991	23/5/2023	P3577	SBMS	43	5	3	1	Malay
1992	23/5/2023	P3580	ABS	34	4	3	0	Malay
1993	23/5/2023	P3578	SNBMI	37	2	1	0	Malay
1994	23/5/2023	P3585	FFBAH	30	3	1	1	Malay
1995	23/5/2023	P3576	NIBF	37	5	3	1	Malay
1996	23/5/2023	P3595	NSBB	30	2	1	0	Malay
1997	23/5/2023	P3594	NZBB	34	4	3	0	Malay
1998	23/5/2023	P3590	TYL	33	2	1	0	Chinese
1999	23/5/2023	P3587	MBMF	45	5	3	1	Malay
2001	23/5/2023	P3596	NFBS	31	3	2	0	Malay
2002	23/5/2023	P3589	SBS	33	6	3	2	Malay
2003	23/5/2023	P3593	SASR	31	2	0	1	Indian
2008	24/5/2023	P3598	KAS	42	4	3	0	Indian
2009	24/5/2023	P3592	LAP	34	2	1	0	Indian
2010	24/5/2023	P3588	RAP	36	1	0	0	Indian
2011	24/5/2023	P3597	LYJ	33	2	1	0	Chinese
2012	24/5/2023	P3591	NFABMF	32	1	0	0	Malay
2013	24/5/2023	P3599	AABAK	34	6	3	2	Malay
2014	24/5/2023	P3608	NBS	35	3	1	1	Malay
2015	25/5/2023	P3611	ABA	35	4	3	0	Malay
2017	25/5/2023	P3606	STJP	31	1	0		Chinese
2020	25/5/2023	P3612	LSL	35	2	1	0	Chinese
2021	25/5/2023	P3605	TSS	40	2	1	0	Chinese
2024	25/5/2023	P3604	FSH	36	2	0	1	Chinese
2026	25/5/2023	P3610	CWH	32	2	1	0	Chinese
2029	25/5/2023	P3607	LAA	32	2	1	0	Indian
2030	25/5/2023	P3601	NMM	35	3	2	0	Malay
2033	25/5/2023	P3600	NBT	35	2	2	0	Chinese
2035	25/5/2023	P3622	BBA	44	6	3	2	Malay
2036	26/5/2023	P3609	MSOBAH	43	5	4	0	Malay
2037	29/5/2023	P3603	TSJC	34	5	2	3	Chinese
2040	29/5/2023	P3602	NHBMY	35	1	0	0	Malay

2041	29/5/2023	P3620	SNFBMR	38	5	4	0	Malay
2042	29/5/2023	P3621	SMBM	30	3	2	0	Malay
2043	29/5/2023	P3624	NABMS	30	2	1	0	Malay
2044	29/5/2023	P3623	TKY	35	3	1	1	Chinese
2045	29/5/2023	P3625	FBAT	30	1	0	0	Malay
2047	29/5/2023	P3616	NIBMR	30	1	0	0	Malay
2048	29/5/2023	P3617	ASBT	33	2	1	0	Malay
2049	29/5/2023	P3618	TBZA	30	1	0	0	Malay
2050	29/5/2023	P3613	ILBH	31	2	1	0	Malay
2051	30/5/2023	P3614	RBA	37	2	1	0	Malay
2052	30/5/2023	P3631	LSL	31	3	2	0	Chinese
2054	30/5/2023	P3619	SSBAH	30	1	0	0	Malay
2055	30/5/2023	P3632	RAKV	33	2	1	0	Indian
2056	30/5/2023	P3615	SBBAS	32	3	2	0	Malay
2057	30/5/2023	P3630	ZABGH	39	4	2	1	Malay
2058	30/5/2023	P3626	NAFBAH	34	1	0	0	Malay
2059	30/5/2023	P3627	NSBAR	35	5	4	0	Malay
2061	30/5/2023	P3629	SNBN	32	1	0	0	Malay
2063	30/5/2023	P3628	CNAC	40	3	2	0	Indian
2064	30/5/2023	P3633	NBD	30	2	1	0	Malay
2065	30/5/2023	P3634	IMBO	34	2	0	1	Malay
2066	30/5/2023	P3635	KSG	33	1	0		Chinese
2068	31/5/2023	P3636	LLP	31	1	0	0	Chinese
2071	31/5/2023	P3653	NABNW	37	5	2	1	Malay
2073	31/5/2023	P3655	AFBAF	33	1	0	0	Malay
2074	31/5/2023	P3637	TMA	33	2	1	0	Chinese
2076	31/5/2023	p3638	HJP	31	1	0	0	Chinese
2077	31/5/2023	P3654	NABA	33	2	1	0	Malay
2080	31/5/2023	P3657	FBMZ	37	5	3	1	Malay
2081	31/5/2023	P3650	NBSH	33	1	0	0	Malay
2082	1/6/2023	P3656	NHBAA	38	4	3	0	Malay
2083	1/6/2023	P3648	NIBK	38	4	0	3	Malay
2085	1/6/2023	P3658	SSABJ	37	5	3	1	Malay

2086	1/6/2023	P3646	VAK	38	6	1	4	Indian
2087	1/6/2023	P3652	PJAA	31	3	0	2	Indian
2088	1/6/2023	P3649	LMB	31	1	0	0	Chinese
2089	1/6/2023	P3651	SSMBS	36	2	1	0	Malay
2090	1/6/2023	P3647	NABD	37	4	2	1	Malay
2091	1/6/2023	P3665	KHT	34	3	2	0	Chinese
2092	1/6/2023	P3663	CAL	32	1	0	0	Chinese
2094	1/6/2023	P3671	KAV	36	3	1	1	Indian
2095	1/6/2023	P3661	LGY	35	1	0	0	Chinese
2096	6/6/2023	P3660	SAS	31	1	0	0	Indian
2099	6/6/2023	P3662	TNFBTAR	38	4	3	0	Malay
2100	6/6/2023	P3670	NBM	35	3	2	0	Malay
2101	6/6/2023	p3664	SFBCA	41	5	4	0	Malay
2102	6/6/2023	P3669	NNIBIJ	32	2	1	0	Malay
2103	6/6/2023	P3666	ABZ	41	5	3	1	Malay
2104	6/6/2023	P3668	TAM	31	2	1	0	Indian
2105	6/6/2023	P3659	NBA	30	2	1	0	Malay
2106	6/6/2023	P3667	TDAV	36	3	1	1	Indian
2108	7/6/2023	P3682	GJC	37	1	0	0	Chinese
2109	7/6/2023	P3675	NBAH	30	1	0	0	Malay
2110	7/6/2023	P3683	NFBHF	34	1	0	0	Malay
2111	7/6/2023	P3681	HNBMH	33	4	1	2	Malay
2114	7/6/2023	P3684	NABA	30	4	3	0	Malay
2116	7/6/2023	P3678	NBMH	39	7	2	4	Malay
2117	7/6/2023	P3679	SNHBZ	32	4	2	1	Malay
2118	7/6/2023	P3680	LZY	30	1	0	0	Chinese
2119	7/6/2023	P3677	NNBAH	31	2	1	0	Malay
2120	7/6/2023	P3676	SBS	31	4	3	0	Malay
2121	8/6/2023	P3672	ZBAB	40	5	3	2	Malay
2123	8/6/2023	P3673	SABF	32	3	2	0	Malay
2124	8/6/2023	P3674	TMAP	36	1	0	0	Indian
2125	8/6/2023	P3689	AMBIW	33	2	1	0	Malay
2127	8/6/2023	P3695	NBI	43	6	4	1	Malay

2128	8/6/2023	P3690	NBH	40	6	2	3	Malay
2129	8/6/2023	P3696	NNBG	31	1	0	0	Malay
2130	8/6/2023	P3697	KAK	32	3	0	2	Indian
2131	8/6/2023	P3691	NSBMH	34	1	0	0	Malay
2135	12/6/2023	P3685	TJA	32	1	0	0	Chinese
2136	12/6/2023	P3688	TSY	32	2	1	0	Chinese
2137	12/6/2023	P3687	YAG	36	4	2	1	Indian
2138	12/6/2023	P3693	NHBM	35	2	1	0	Malay
2140	12/6/2023	P3694	TAJR	31	2	1	0	Indian
2142	12/6/2023	P3705	AFBAT	31	2	1	0	Malay
2143	12/6/2023	P3706	NBS	31	3	2	0	Malay
2144	12/6/2023	P3704	NABS	34	3	2	0	Malay
2145	12/6/2023	P3699	SBS	38	4	2	1	Malay
2146	12/6/2023	P3698	NSBMA	31	3	1	1	Malay
2150	13/6/2023	P3708	DAK	30	1	0	0	Indian
2154	13/6/2023	P3707	SFBMF	34	3	2	0	Malay
2155	13/6/2023	P3710	SBZ	32	3	2	0	Malay
2158	14/6/2023	P3715	CKB	32	3	0	2	Chinese
2159	14/6/2023	P3702	NSBMA	32	1	0	0	Malay
2160	14/6/2023	P3703	SNABR	32	2	1	0	Malay
2161	14/6/2023	P3701	NIBH	30	1	0	0	Malay
2162	14/6/2023	P3714	GKM	35	2	0	1	Chinese
2163	14/6/2023	P3716	NAR	34	2	1	0	Indian
2166	15/6/2023	P3712	NBABA	32	1	0	0	Malay
2169	15/6/2023	P3713	NFBA	38	4	3	0	Malay
2170	15/6/2023	P3711	NABMI	32	2	1	0	Malay
2171	15/6/2023	P3717	SMBS	34	3	1	0	Malay
2173	15/6/2023	P3718	NHBT	35	2	1	0	Malay
2176	15/6/2023	P3721	FLBAH	33	1	0	0	Malay
2177	15/6/2023	P3720	MBRA	35	1	0	0	Malay
2178	15/6/2023	P3723	PST	37	3	1	1	Chinese
2182	16/6/2023	P3735	ZBZ	35	3	1	1	Malay
2183	19/6/2023	P3722	MBM	37	5	4	0	Malay

2184	19/6/2023	P3734	TFK	32	1	0	0	Chinese	
2187	19/6/2023	P3736	MBJ	36	9	4	4	Malay	
2188	19/6/2023	P3726	ABMD	31	2	1	0	Malay	
2192	19/6/2023	P3724	WWC	35	2	1	0	Chinese	
2195	20/6/2023	P3725	NNABS	31	4	2	1	Malay	
2196	20/6/2023	P3730	ILWR	33	1	0	0	Chinese	
2197	20/6/2023	P3728	MAS	35	2	0	0	Indian	
2198	20/6/2023	P3729	KAM	32	1	0	0	Indian	
2201	21/6/2023	P3731	MAV	30	2	1	0	Indian	
2203	21/6/2023	P3732	AABI	31	3	2	0	Malay	
2204	21/6/2023	P3733	TAM	31	1	0	0	Indian	
2205	21/6/2023	P3748	SASR	31	2	0	1	Indian	
2207	21/6/2023	P3747	ZBZ	39	4	3	0	Malay	
2211	22/6/2023	P3749	SSBG	33	3	2	0	Malay	
2213	22/6/2023	P3727	SNBMR	32	1	0	0	Malay	
2215	22/6/2023	P3744	AABMY	31	2	1	0	Malay	
2218	26/6/2023	P3741	PESBMT	31	1	0	0	Malay	
2219	26/6/2023	P3742	SFSR	36	7	3	3	Others	Kadazan
2220	26/6/2023	P3743	ADV	38	3	1	1	Others	Kadazan
2221	26/6/2023	P3746	YE	32	1	0	0	Chinese	
2222	26/6/2023	P3745	NYZ	34	2	1	0	Chinese	
2224	27/6/2023	P3761	NHBH	38	3	2	1	Malay	
2226	27/6/2023	P3740	TJV	36	2	0	1	Chinese	
2228	27/6/2023	P3755	ABMR	41	6	5	0	Malay	
2229	27/6/2023	P3756	CPE	39	4	2	1	Chinese	
2231	27/6/2023	P3750	SAM	32	1	0	0	Indian	
2233	27/6/2023	P3751	NBZ	31	2	1	0	Malay	
2235	28/6/2023	P3754	NAG	36	1	0	0	Indian	
2236	28/6/2023	P3762	CYT	47	1	0	0	Chinese	
2237	28/6/2023	P3752	WFABWN	32	1	0	0	Malay	
2239	3/7/2023	P3753	LBK	37	5	4	0	Malay	
2241	3/7/2023	P3758	QABZF	30	4	3	0	Malay	
2242	3/7/2023	P3757	MBAM	38	6	5	0	Malay	

2243	3/7/2023	P3759	KATV	36	2	1	0	Indian	
2245	3/7/2023	P3772	FRBCS	31	3	1	1	Malay	
2246	3/7/2023	P3770	GAS	32	3	0	2	Indian	
2247	4/7/2023	P3773	NABA	33	3	1	1	Malay	
2248	4/7/2023	P3774	YYH	36	3	1	1	Chinese	
2250	4/7/2023	P3775	SNABMN	38	4	3	0	Malay	
2252	4/7/2023	P3771	WRBWS	35	4	3	0	Malay	
2253	4/7/2023	P3768	SAZBZA	37	5	4	0	Malay	
2257	4/7/2023	P3767	NSBMP	34	2	1	0	Malay	
2259	5/7/2023	P3765	NIBH	30	2	2	0	Malay	
2260	5/7/2023	P3764	TANBTS	31	3	1	1	Malay	
2261	5/7/2023	P3763	NAT	31	1	0	0	Indian	
2262	5/7/2023	P3766	NABK	32	2	1	0	Malay	
2263	5/7/2023	P3769	PMJ	30	1	0	0	Chinese	
2265	5/7/2023	P3787	NABMAB	32	2	0	1	Malay	
2266	5/7/2023	P3788	PATR	31	1	0	0	Indian	
2269	6/7/2023	P3786	SAA	35	2	1	0	Indian	
2270	6/7/2023	P3782	RAC	35	4	2	1	Indian	
2272	6/7/2023	P3780	SHBMG	35	4	3	0	Malay	
2273	6/7/2023	P3781	HP	30	3	1	1	Others	kadazandusun
2274	10/7/2023	P3779	ESBS	35	2	0	1	Malay	
2275	10/7/2023	P3776	NBM	35	2	1	0	Malay	
2276	10/7/2023	P3777	NANBMZ	32	4	0	3	Malay	
2277	10/7/2023	P3778	NABMJ	34	2	1	0	Malay	
2278	10/7/2023	P3785	CAA	35	1	0	0	Indian	
2280	11/7/2023	P3783	ILBS	36	3	1	1	Malay	
2282	11/7/2023	P3784	OBH	32	1	0	0	Chinese	
2283	11/7/2023	P3800	YLL	40	1	0	0	Chinese	
2284	11/7/2023	P3799	SBAN	40	5	3	1	Malay	
2286	11/7/2023	P3801	JO	36	5	2	2	Chinese	
2288	12/7/2023	P3792	CAC	31	2	0	1	Others	Eurasian
2289	12/7/2023	P3790	SKBMR	39	6	3	2	Malay	
2290	12/7/2023	P3791	YPL	43	1	0	0	Chinese	

2299	13/7/2023	P3789	DAS	30	1	0	0	Indian
2300	13/7/2023	P3795	TKY	32	2	1	0	Chinese
2301	13/7/2023	P3793	ISBHS	33	1	0	0	Malay
2302	13/7/2023	P3794	NHBZM	37	8	3	4	Malay
2304	17/7/2023	P3814	MAN	30	1	0	0	Indian
2305	17/7/2023	P3797	FMBH	38	5	3	1	Malay
2306	17/7/2023	P3796	NSSBN	36	6	3	2	Malay
2307	17/7/2023	P3812	HBM	40	5	4	0	Malay
2308	17/7/2023	P3811	TKH	30	1	0	0	Chinese
2309	17/7/2023	P3813	ENBMAL	30	2	1	0	Malay
2312	18/7/2023	P3808	NBAR	30	4	0	3	Malay
2314	18/7/2023	P3806	NBS	34	1	0	0	Malay
2315	18/7/2023	P3807	NABN	31	1	0	0	Malay
2317	18/7/2023	P3810	YSBZ	30	2	1	0	Malay
2319	18/7/2023	P3809	LBBMR	37	4	2	1	Malay
2321	20/7/2023	P3804	CSW	36	2	0	1	Chinese
2322	20/7/2023	P3805	UAT	37	2	1	0	Indian
2323	20/7/2023	P3802	PSL	31	1	0	0	Chinese
2325	20/7/2023	P3803	PAS	31	3	1	0	Indian
2332	24/7/2023	P3827	ABY	31	1	0	0	Malay
2333	24/7/2023	P3825	NABAZ	33	2	1	0	Malay
2336	24/7/2023	P3826	AAL	40	2	1	0	Chinese
2338	25/7/2023	P3824	AL	33	2	0	1	Chinese
2342	25/7/2023	P3822	ZBK	30	1	0	0	Malay
2344	25/7/2023	P3823	UAP	40	1	0	0	Indian
2345	25/7/2023	P3821	NWBW	38	7	2	4	Malay
2352	26/7/2023	P3819	SABH	38	1	0	0	Malay
2355	26/7/2023	P3820	NBAG	33	1	0	0	Malay
2360	26/7/2023	P3815	NBCMZ	39	5	2	2	Malay
2362	27/7/2023	P3818	NFBAK	39	4	3	0	Malay
2366	27/7/2023	P3816	NHBI	32	3	2	0	Malay
2368	27/7/2023	P3817	LSL	33	1	0	0	Chinese
2369	31/7/2023	P3840	ABAN	39	4	3	0	Malay

2372	31/7/2023	P3839	NBN	37	2	0	1	Malay
2374	31/7/2023	P3838	WNABWB	32	1	0	0	Malay
2376	1/8/2023	P3834	MBM	35	4	3	0	Malay
2377	1/8/2023	P3832	TSF	34	4	0	3	Chinese
2378	1/8/2023	P3833	SIBA	32	1	0	0	Malay
2379	1/8/2023	P3837	MAP	32	2	0	1	Indian
2380	2/8/2023	P3836	CTL	35	1	0	0	Chinese
2383	2/8/2023	P3835	NABAS	33	3	2	0	Malay
2385	3/8/2023	P3831	SKBZ	42	5	3	1	Malay
2386	3/8/2023	P3830	HBD	32	2	1	0	Malay
2388	7/8/2023	P3828	PAS	34	3	2	0	Indian
2389	7/8/2023	P3848	LAM	31	2	1	0	Indian
2391	7/8/2023	P3849	LAN	31	2	0	1	Indian
2392	7/8/2023	P3829	YLS	36	1	0	0	Chinese
2394	8/8/2023	P3852	JTSS	33	1	0	0	Chinese
2396	8/8/2023	P3853	NZBY	37	6	4	1	Malay
2397	8/8/2023	P3851	SSARS	33	1	0	0	Indian
2398	8/8/2023	P3847	WNBMAA	35	5	3	1	Malay
2399	8/8/2023	P3845	NBS	38	3	2	0	Malay
2400	8/8/2023	P3846	HZBZ	34	1	0	0	Malay
2401	9/8/2023	P3844	CYC	37	4	2	1	Chinese
2406	10/8/2023	P3842	NFBKA	34	3	2	0	Malay
2407	10/8/2023	P3843	NHBN	32	5	3	1	Malay
2408	10/8/2023	P3841	LYC	34	2	1	0	Chinese
2409	10/8/2023	P3866	NBZ	35	1	0	0	Malay
2410	14/8/2023	P3864	SAP	34	1	0	0	Indian
2411	14/8/2023	P3865	YAM	34	1	0	0	Indian
2414	15/8/2023	P3862	SFBAH	39	5	3	1	Malay
2416	15/8/2023	P3860	LLL	32	1	0	0	Chinese
2421	15/8/2023	P3859	SBJ	34	3	2	0	Malay
2422	16/8/2023	P3861	GPY	30	2	0	1	Chinese
2423	16/8/2023	P3858	NBS	39	5	4	0	Malay
2424	16/8/2023	P3876	CLJ	30	2	1	0	Chinese

2425	16/8/2023	P3874	NJBK	31	5	3	1	Malay
2426	16/8/2023	P3875	ABA	36	2	1	0	Malay
2427	16/8/2023	P3879	LMS	32	2	1	0	Chinese
2428	16/8/2023	P3878	NBAJ	32	4	2	1	Malay
2429	17/8/2023	P3873	CC	43	2	1	0	Chinese
2430	17/8/2023	P3871	SABMA	31	2	0	1	Malay
2437	17/8/2023	P3877	NBMS	35	2	1	0	Malay
2438	21/8/2023	P3870	NV	37	3	1	1	Indian
2439	21/8/2023	P3863	NSBMA	31	2	0	1	Malay
2442	21/8/2023	P3872	MAMM	34	4	1	2	Indian
2444	22/8/2023	P3867	ANBG	33	2	1	0	Malay
2445	22/8/2023	P3868	NSBMS	32	3	2	0	Malay
2446	22/8/2023	P3869	AP	33	2	0	1	Indian
2448	22/8/2023	P3857	ABA	38	5	3	1	Malay
2456	23/8/2023	P3856	PAKS	35	4	3	0	Indian
2459	23/8/2023	P3854	NBS	39	2	0	1	Malay
2461	24/8/2023	P3855	EABR	31	3	2	0	Malay
2462	24/8/2023	P3892	ASBN	35	1	0	0	Malay
2463	24/8/2023	P3891	NPS	35	2	1	0	Chinese
2465	24/8/2023	P3890	AABAR	31	3	2	0	Malay
2466	24/8/2023	P3889	NNBT	36	5	4	0	Malay
2469	28/8/2023	P3888	SNBI	32	1	0	0	Malay
2470	28/8/2023	P3887	ABA	39	3	1	1	Malay
2474	28/8/2023	P3885	NSBR	34	4	3	0	Malay
2475	28/8/2023	P3884	SHBR	33	2	1	0	Malay
2476	29/8/2023	P3886	NSBMIA	33	2	1	0	Malay
2477	29/8/2023	P3883	INSBO	32	3	2	0	Malay
2478	29/8/2023	P3882	HBAH	39	9	4	4	Malay
2479	30/8/2023	P3880	NSW	32	2	1	0	Chinese
2480	30/8/2023	P3881	SDAM	36	5	3	1	Indian
2483	30/8/2023	P3905	FSBS	30	1	0	0	Malay
2487	4/9/2023	P3904	AJJ	32	2	1	0	Chinese
2492	4/9/2023	P3901	NHBM	30	1	0	0	Malay

2499	7/9/2023	P3902	NBAR	33	3	2	0	Malay
2503	7/9/2023	P3899	MBS	33	2	1	0	Malay
2504	7/9/2023	P3897	ZBH	38	2	0	1	Malay
2505	11/9/2023	P3900	NIBI	30	2	1	0	Malay
2506	11/9/2023	P3893	YKY	38	5	1	2	Chinese
2507	11/9/2023	P3898	SLW	34	1	0	0	Chinese
2508	11/9/2023	P3894	YAVP	31	1	0	0	Indian
2509	11/9/2023	P3895	NSBAR	30	1	0	0	Malay
2511	11/9/2023	P3896	KWBN	30	1	0	0	Malay
2512	12/9/2023	P3903	LJL	30	1	0	0	Chinese
2514	12/9/2023	P3918	VAG	36	3	2	0	Indian
2516	12/9/2023	P3917	NAC	32	2	1	0	Indian
2517	12/9/2023	P3916	ANBMN	33	2	1	0	Malay
2519	12/9/2023	P3913	NIBK	36	4	3	0	Malay
2521	12/9/2023	P3914	SKL	37	2	1	0	Chinese
2525	13/9/2023	P3915	SABMA	30	4	2	1	Malay
2526	13/9/2023	P3912	LSL	32	1	0	0	Chinese
2528	13/9/2023	P3910	OJBJ	39	4	3	0	Malay
2532	13/9/2023	P3909	YAN	39	2	1	0	Chinese
2533	14/9/2023	P3911	NJBK	33	1	0	0	Malay
2535	14/9/2023	P3850	NABA	31	2	1	0	Malay
2536	14/9/2023	P3798	JMAR	41	3	2	0	Indian
2539	14/9/2023	P3719	FNBA	30	1	0	0	Malay
2540	14/9/2023	P3908	SBS	41	8	6	1	Malay
2541	18/9/2023	P3907	GXY	33	2	0	1	Chinese
2543	18/9/2023	P3906	CHX	30	1	0	0	Chinese
2544	18/9/2023	P3931	NBI	37	5	4	0	Malay
2545	18/9/2023	P3929	NISBY	30	3	1	1	Malay
2547	18/9/2023	P3930	HBH	33	3	2	0	Malay
2548	19/9/2023	P3923	HAG	30	4	1	2	Indian
2550	19/9/2023	P3925	HSS	34	3	1	1	Chinese
2556	21/9/2023	P3924	NLBAW	32	4	2	1	Malay
2559	21/9/2023	P3919	NBR	34	2	1	0	Malay

2561	21/9/2023	P3920	SAGA	32	2	1	0	Indian
2563	25/9/2023	P3922	SNBK	35	5	3	1	Malay
2567	25/9/2023	P3921	NWYR	35	2	1	0	Chinese
2569	25/9/2023	P3942	HABMN	40	7	4	2	Malay
2575	27/9/2023	P3943	NBCN	31	3	1	1	Malay
2577	27/9/2023	P3944	CYL	31	2	1	0	Chinese
2583	27/9/2023	P3936	NBMR	34	1	0	0	Malay
2585	16/10/2023	P3937	TGC	38	2	1	0	Chinese
2589	16/10/2023	P3938	SPAR	30	1	0	0	Indian
2590	16/10/2023	P3941	NSBMY	36	4	3	0	Malay
2592	16/10/2023	P3940	MBM	33	3	2	0	Malay
2594	16/10/2023	P3935	TCS	37	4	3	0	Chinese
2595	16/10/2023	P3939	RAK	33	2	0	1	Indian
2596	16/10/2023	P3934	LCL	38	2	1	0	Chinese
2598	18/10/2023	P3932	NBMN	40	4	2	1	Malay
2599	18/10/2023	P3956	MMAS	41	5	3	1	Indian
2601	18/10/2023	P3933	SWX	30	1	0	0	Chinese
2602	18/10/2023	P3955	SABAB	39	1	0	0	Malay
2604	18/10/2023	P3951	NFBY	32	2	1	0	Malay
2605	18/10/2023	P3949	UNBM	30	1	0	0	Malay

Highest level of education	Household income	Employment status
Tertiary (including Polytechnics, MARA Colleges, University Colleges, and University)	RM 5,001 - RM 10,000 per month	Work from home/housewife
Tertiary (including Polytechnics, MARA Colleges, University Colleges, and University)	RM 1,001 to 5,000 per month	Work from home/housewife
Secondary	RM 1,001 to 5,000 per month	Employed, full time
Tertiary (including Polytechnics, MARA Colleges, University Colleges, and University)	RM 5,001 - RM 10,000 per month	Work from home/housewife
Secondary	RM 5,001 - RM 10,000 per month	Work from home/housewife
Tertiary (including Polytechnics, MARA Colleges, University Colleges, and University)	RM 1,001 to 5,000 per month	Work from home/housewife
Post-graduates	RM 1,001 to 5,000 per month	Employed, full time
Tertiary (including Polytechnics, MARA Colleges, University Colleges, and University)	RM 5,001 - RM 10,000 per month	Work from home/housewife
Secondary	RM 1,001 to 5,000 per month	Work from home/housewife
Tertiary (including Polytechnics, MARA Colleges, University Colleges, and University)	RM 1,001 to 5,000 per month	Employed, full time
Tertiary (including Polytechnics, MARA Colleges, University Colleges, and University)	RM 5,001 - RM 10,000 per month	Employed, full time
Tertiary (including Polytechnics, MARA Colleges, University Colleges, and University)	RM 5,001 - RM 10,000 per month	Employed, full time
Tertiary (including Polytechnics, MARA Colleges, University Colleges, and University)	RM 5,001 - RM 10,000 per month	Work from home/housewife
Tertiary (including Polytechnics, MARA Colleges, University Colleges, and University)	RM 5,001 - RM 10,000 per month	Employed, full time
Tertiary (including Polytechnics, MARA Colleges, University Colleges, and University)	RM 1,001 to 5,000 per month	Work from home/housewife
Tertiary (including Polytechnics, MARA Colleges, University Colleges, and University)	RM 1,001 to 5,000 per month	Employed, full time
Tertiary (including Polytechnics, MARA Colleges, University Colleges, and University)	RM 5,001 - RM 10,000 per month	Work from home/housewife
Secondary	RM 1,001 to 5,000 per month	Work from home/housewife
Post-graduates	RM 5,001 - RM 10,000 per month	Employed, full time
Tertiary (including Polytechnics, MARA Colleges, University Colleges, and University)	>RM 10,000 per month	Employed, part time
Tertiary (including Polytechnics, MARA Colleges, University Colleges, and University)	RM 5,001 - RM 10,000 per month	Employed, full time
Tertiary (including Polytechnics, MARA Colleges, University Colleges, and University)	RM 1,001 to 5,000 per month	Employed, full time
Tertiary (including Polytechnics, MARA Colleges, University Colleges, and University)	RM 5,001 - RM 10,000 per month	Temporarily unemployed/medical leave/maternity
Tertiary (including Polytechnics, MARA Colleges, University Colleges, and University)	RM 1,001 to 5,000 per month	Work from home/housewife
Secondary	RM 5,001 - RM 10,000 per month	Employed, part time
Secondary	RM 1,001 to 5,000 per month	Employed, full time
Tertiary (including Polytechnics, MARA Colleges, University Colleges, and University)	RM 1,001 to 5,000 per month	Work from home/housewife
Secondary	RM 1,001 to 5,000 per month	Employed, full time
Secondary	RM 1,001 to 5,000 per month	Work from home/housewife
Secondary	RM 5,001 - RM 10,000 per month	Work from home/housewife
Tertiary (including Polytechnics, MARA Colleges, University Colleges, and University)	RM 1,001 to 5,000 per month	Employed, full time
Tertiary (including Polytechnics, MARA Colleges, University Colleges, and University)	RM 5,001 - RM 10,000 per month	Work from home/housewife

Tertiary (including Polytechnics, MARA Colleges, University Colleges, and University)	RM 1,001 to 5,000 per month	Work from home/housewife
Tertiary (including Polytechnics, MARA Colleges, University Colleges, and University)	RM 5,001 - RM 10,000 per month	Employed, full time
Tertiary (including Polytechnics, MARA Colleges, University Colleges, and University)	RM 1,001 to 5,000 per month	Work from home/housewife
Tertiary (including Polytechnics, MARA Colleges, University Colleges, and University)	>RM 10,000 per month	Work from home/housewife
Tertiary (including Polytechnics, MARA Colleges, University Colleges, and University)	RM 5,001 - RM 10,000 per month	Employed, full time
Secondary	RM 1,001 to 5,000 per month	Employed, full time
Post-graduates	>RM 10,000 per month	Employed, full time
Secondary	>RM 10,000 per month	Employed, full time
Secondary	RM 1,001 to 5,000 per month	Employed, part time
Secondary	RM 1,001 to 5,000 per month	Work from home/housewife
Tertiary (including Polytechnics, MARA Colleges, University Colleges, and University)	RM 5,001 - RM 10,000 per month	Employed, full time
Post-graduates	RM 1,001 to 5,000 per month	Work from home/housewife
Tertiary (including Polytechnics, MARA Colleges, University Colleges, and University)	>RM 10,000 per month	Work from home/housewife
Tertiary (including Polytechnics, MARA Colleges, University Colleges, and University)	RM 5,001 - RM 10,000 per month	Employed, full time
Tertiary (including Polytechnics, MARA Colleges, University Colleges, and University)	RM 5,001 - RM 10,000 per month	Employed, full time
Tertiary (including Polytechnics, MARA Colleges, University Colleges, and University)	RM 5,001 - RM 10,000 per month	Employed, full time
Tertiary (including Polytechnics, MARA Colleges, University Colleges, and University)	RM 1,001 to 5,000 per month	Employed, full time
Tertiary (including Polytechnics, MARA Colleges, University Colleges, and University)	RM 1,001 to 5,000 per month	Employed, full time
Tertiary (including Polytechnics, MARA Colleges, University Colleges, and University)	RM 1,001 to 5,000 per month	Temporarily unemployed/medical leave/maternity
Tertiary (including Polytechnics, MARA Colleges, University Colleges, and University)	RM 1,001 to 5,000 per month	Employed, full time
Tertiary (including Polytechnics, MARA Colleges, University Colleges, and University)	RM 1,001 to 5,000 per month	Work from home/housewife
Tertiary (including Polytechnics, MARA Colleges, University Colleges, and University)	>RM 10,000 per month	Work from home/housewife
Tertiary (including Polytechnics, MARA Colleges, University Colleges, and University)	>RM 10,000 per month	Work from home/housewife
Tertiary (including Polytechnics, MARA Colleges, University Colleges, and University)	>RM 10,000 per month	Work from home/housewife
Tertiary (including Polytechnics, MARA Colleges, University Colleges, and University)	RM 1,001 to 5,000 per month	Employed, full time
Tertiary (including Polytechnics, MARA Colleges, University Colleges, and University)	RM 5,001 - RM 10,000 per month	Employed, full time
Post-graduates	RM 1,001 to 5,000 per month	Work from home/housewife
Tertiary (including Polytechnics, MARA Colleges, University Colleges, and University)	>RM 10,000 per month	Employed, full time
Secondary	<RM 1,000 per month	Work from home/housewife
Tertiary (including Polytechnics, MARA Colleges, University Colleges, and University)	RM 5,001 - RM 10,000 per month	Employed, full time
Tertiary (including Polytechnics, MARA Colleges, University Colleges, and University)	>RM 10,000 per month	Work from home/housewife
Secondary	RM 1,001 to 5,000 per month	Employed, part time
Tertiary (including Polytechnics, MARA Colleges, University Colleges, and University)	>RM 10,000 per month	Employed, full time

Secondary	RM 5,001 - RM 10,000 per month	Employed, full time
Secondary	RM 5,001 - RM 10,000 per month	Work from home/housewife
Tertiary (including Polytechnics, MARA Colleges, University Colleges, and University)	RM 1,001 to 5,000 per month	Employed, full time
Tertiary (including Polytechnics, MARA Colleges, University Colleges, and University)	RM 5,001 - RM 10,000 per month	Employed, full time
Secondary	RM 1,001 to 5,000 per month	Employed, full time
Tertiary (including Polytechnics, MARA Colleges, University Colleges, and University)	RM 5,001 - RM 10,000 per month	Employed, full time
Tertiary (including Polytechnics, MARA Colleges, University Colleges, and University)	RM 5,001 - RM 10,000 per month	Employed, full time
Tertiary (including Polytechnics, MARA Colleges, University Colleges, and University)	RM 5,001 - RM 10,000 per month	Work from home/housewife
Tertiary (including Polytechnics, MARA Colleges, University Colleges, and University)	RM 1,001 to 5,000 per month	Employed, full time
Tertiary (including Polytechnics, MARA Colleges, University Colleges, and University)	RM 5,001 - RM 10,000 per month	Employed, full time
Tertiary (including Polytechnics, MARA Colleges, University Colleges, and University)	RM 5,001 - RM 10,000 per month	Employed, full time
Tertiary (including Polytechnics, MARA Colleges, University Colleges, and University)	RM 1,001 to 5,000 per month	Employed, full time
Secondary	RM 1,001 to 5,000 per month	Employed, full time
Tertiary (including Polytechnics, MARA Colleges, University Colleges, and University)	RM 5,001 - RM 10,000 per month	Employed, full time
Post-graduates	RM 5,001 - RM 10,000 per month	Work from home/housewife
Tertiary (including Polytechnics, MARA Colleges, University Colleges, and University)	RM 1,001 to 5,000 per month	Employed, full time
Secondary	RM 1,001 to 5,000 per month	Employed, full time
Tertiary (including Polytechnics, MARA Colleges, University Colleges, and University)	>RM 10,000 per month	Employed, full time
Tertiary (including Polytechnics, MARA Colleges, University Colleges, and University)	>RM 10,000 per month	Employed, full time
Tertiary (including Polytechnics, MARA Colleges, University Colleges, and University)	RM 5,001 - RM 10,000 per month	Employed, full time
Post-graduates	RM 1,001 to 5,000 per month	Work from home/housewife
Tertiary (including Polytechnics, MARA Colleges, University Colleges, and University)	RM 1,001 to 5,000 per month	Employed, full time
Tertiary (including Polytechnics, MARA Colleges, University Colleges, and University)	RM 1,001 to 5,000 per month	Employed, full time
Tertiary (including Polytechnics, MARA Colleges, University Colleges, and University)	RM 5,001 - RM 10,000 per month	Employed, full time
Secondary	RM 1,001 to 5,000 per month	Work from home/housewife
Tertiary (including Polytechnics, MARA Colleges, University Colleges, and University)	RM 5,001 - RM 10,000 per month	Employed, full time
Tertiary (including Polytechnics, MARA Colleges, University Colleges, and University)	RM 5,001 - RM 10,000 per month	Work from home/housewife
Post-graduates	RM 1,001 to 5,000 per month	Employed, full time
Tertiary (including Polytechnics, MARA Colleges, University Colleges, and University)	RM 5,001 - RM 10,000 per month	Employed, full time
Tertiary (including Polytechnics, MARA Colleges, University Colleges, and University)	RM 5,001 - RM 10,000 per month	Employed, full time
Tertiary (including Polytechnics, MARA Colleges, University Colleges, and University)	RM 1,001 to 5,000 per month	Employed, full time
Tertiary (including Polytechnics, MARA Colleges, University Colleges, and University)	RM 5,001 - RM 10,000 per month	Employed, full time
Tertiary (including Polytechnics, MARA Colleges, University Colleges, and University)	RM 1,001 to 5,000 per month	Work from home/housewife

Tertiary (including Polytechnics, MARA Colleges, University Colleges, and University)	>RM 10,000 per month	Employed, part time
Tertiary (including Polytechnics, MARA Colleges, University Colleges, and University)	RM 1,001 to 5,000 per month	Work from home/housewife
Tertiary (including Polytechnics, MARA Colleges, University Colleges, and University)	RM 1,001 to 5,000 per month	Employed, full time
Secondary	RM 5,001 - RM 10,000 per month	Employed, full time
Tertiary (including Polytechnics, MARA Colleges, University Colleges, and University)	RM 5,001 - RM 10,000 per month	Work from home/housewife
Tertiary (including Polytechnics, MARA Colleges, University Colleges, and University)	RM 1,001 to 5,000 per month	Employed, full time
Post-graduates	RM 5,001 - RM 10,000 per month	Work from home/housewife
Tertiary (including Polytechnics, MARA Colleges, University Colleges, and University)	RM 1,001 to 5,000 per month	Employed, full time
Secondary	RM 1,001 to 5,000 per month	Work from home/housewife
Tertiary (including Polytechnics, MARA Colleges, University Colleges, and University)	RM 1,001 to 5,000 per month	Employed, full time
Tertiary (including Polytechnics, MARA Colleges, University Colleges, and University)	RM 1,001 to 5,000 per month	Work from home/housewife
Secondary	<RM 1,000 per month	Work from home/housewife
Tertiary (including Polytechnics, MARA Colleges, University Colleges, and University)	RM 5,001 - RM 10,000 per month	Employed, full time
Secondary	RM 1,001 to 5,000 per month	Employed, full time
Secondary	<RM 1,000 per month	Work from home/housewife
Tertiary (including Polytechnics, MARA Colleges, University Colleges, and University)	RM 1,001 to 5,000 per month	Employed, full time
Tertiary (including Polytechnics, MARA Colleges, University Colleges, and University)	RM 5,001 - RM 10,000 per month	Employed, full time
Secondary	RM 5,001 - RM 10,000 per month	Employed, full time
Tertiary (including Polytechnics, MARA Colleges, University Colleges, and University)	RM 1,001 to 5,000 per month	Work from home/housewife
Tertiary (including Polytechnics, MARA Colleges, University Colleges, and University)	RM 5,001 - RM 10,000 per month	Work from home/housewife
Tertiary (including Polytechnics, MARA Colleges, University Colleges, and University)	RM 1,001 to 5,000 per month	Work from home/housewife
Secondary	RM 1,001 to 5,000 per month	Work from home/housewife
Tertiary (including Polytechnics, MARA Colleges, University Colleges, and University)	RM 5,001 - RM 10,000 per month	Employed, full time
Tertiary (including Polytechnics, MARA Colleges, University Colleges, and University)	RM 5,001 - RM 10,000 per month	Work from home/housewife
Tertiary (including Polytechnics, MARA Colleges, University Colleges, and University)	>RM 10,000 per month	Employed, full time
Secondary	RM 1,001 to 5,000 per month	Employed, full time
Tertiary (including Polytechnics, MARA Colleges, University Colleges, and University)	RM 5,001 - RM 10,000 per month	Employed, full time
Tertiary (including Polytechnics, MARA Colleges, University Colleges, and University)	RM 1,001 to 5,000 per month	Employed, full time
Tertiary (including Polytechnics, MARA Colleges, University Colleges, and University)	>RM 10,000 per month	Employed, full time
Secondary	RM 1,001 to 5,000 per month	Work from home/housewife
Secondary	RM 1,001 to 5,000 per month	Employed, full time
Tertiary (including Polytechnics, MARA Colleges, University Colleges, and University)	RM 5,001 - RM 10,000 per month	Work from home/housewife
Tertiary (including Polytechnics, MARA Colleges, University Colleges, and University)	RM 1,001 to 5,000 per month	Employed, full time

Tertiary (including Polytechnics, MARA Colleges, University Colleges, and University)	RM 5,001 - RM 10,000 per month	Work from home/housewife
Tertiary (including Polytechnics, MARA Colleges, University Colleges, and University)	RM 1,001 to 5,000 per month	Employed, full time
Secondary	RM 1,001 to 5,000 per month	Work from home/housewife
Post-graduates	>RM 10,000 per month	Employed, full time
Secondary	RM 5,001 - RM 10,000 per month	Employed, full time
Tertiary (including Polytechnics, MARA Colleges, University Colleges, and University)	RM 1,001 to 5,000 per month	Work from home/housewife
Tertiary (including Polytechnics, MARA Colleges, University Colleges, and University)	RM 5,001 - RM 10,000 per month	Employed, full time
Secondary	RM 1,001 to 5,000 per month	Work from home/housewife
Tertiary (including Polytechnics, MARA Colleges, University Colleges, and University)	>RM 10,000 per month	Work from home/housewife
Tertiary (including Polytechnics, MARA Colleges, University Colleges, and University)	RM 5,001 - RM 10,000 per month	Work from home/housewife
Tertiary (including Polytechnics, MARA Colleges, University Colleges, and University)	>RM 10,000 per month	Work from home/housewife
Secondary	RM 1,001 to 5,000 per month	Work from home/housewife
Tertiary (including Polytechnics, MARA Colleges, University Colleges, and University)	RM 1,001 to 5,000 per month	Work from home/housewife
Tertiary (including Polytechnics, MARA Colleges, University Colleges, and University)	RM 5,001 - RM 10,000 per month	Employed, full time
Tertiary (including Polytechnics, MARA Colleges, University Colleges, and University)	>RM 10,000 per month	Employed, full time
Tertiary (including Polytechnics, MARA Colleges, University Colleges, and University)	RM 5,001 - RM 10,000 per month	Employed, full time
Tertiary (including Polytechnics, MARA Colleges, University Colleges, and University)	>RM 10,000 per month	Employed, full time
Tertiary (including Polytechnics, MARA Colleges, University Colleges, and University)	RM 5,001 - RM 10,000 per month	Employed, full time
Tertiary (including Polytechnics, MARA Colleges, University Colleges, and University)	RM 1,001 to 5,000 per month	Employed, full time
Post-graduates	RM 1,001 to 5,000 per month	Work from home/housewife
Tertiary (including Polytechnics, MARA Colleges, University Colleges, and University)	RM 5,001 - RM 10,000 per month	Employed, full time
Tertiary (including Polytechnics, MARA Colleges, University Colleges, and University)	RM 5,001 - RM 10,000 per month	Employed, full time
Tertiary (including Polytechnics, MARA Colleges, University Colleges, and University)	RM 5,001 - RM 10,000 per month	Employed, full time
Secondary	RM 1,001 to 5,000 per month	Employed, full time
Tertiary (including Polytechnics, MARA Colleges, University Colleges, and University)	RM 5,001 - RM 10,000 per month	Employed, full time
Tertiary (including Polytechnics, MARA Colleges, University Colleges, and University)	RM 1,001 to 5,000 per month	Work from home/housewife
Tertiary (including Polytechnics, MARA Colleges, University Colleges, and University)	RM 5,001 - RM 10,000 per month	Employed, full time
Tertiary (including Polytechnics, MARA Colleges, University Colleges, and University)	RM 5,001 - RM 10,000 per month	Work from home/housewife
Tertiary (including Polytechnics, MARA Colleges, University Colleges, and University)	>RM 10,000 per month	Employed, full time
Secondary	<RM 1,000 per month	Work from home/housewife
Tertiary (including Polytechnics, MARA Colleges, University Colleges, and University)	RM 1,001 to 5,000 per month	Employed, full time
Tertiary (including Polytechnics, MARA Colleges, University Colleges, and University)	RM 5,001 - RM 10,000 per month	Work from home/housewife
Tertiary (including Polytechnics, MARA Colleges, University Colleges, and University)	>RM 10,000 per month	Employed, full time

Secondary	RM 1,001 to 5,000 per month	Employed, full time
Tertiary (including Polytechnics, MARA Colleges, University Colleges, and University)	>RM 10,000 per month	Work from home/housewife
Tertiary (including Polytechnics, MARA Colleges, University Colleges, and University)	>RM 10,000 per month	Employed, full time
Secondary	RM 5,001 - RM 10,000 per month	Employed, full time
Tertiary (including Polytechnics, MARA Colleges, University Colleges, and University)	RM 5,001 - RM 10,000 per month	Employed, full time
Secondary	RM 5,001 - RM 10,000 per month	Employed, full time
Tertiary (including Polytechnics, MARA Colleges, University Colleges, and University)	RM 1,001 to 5,000 per month	Employed, full time
Tertiary (including Polytechnics, MARA Colleges, University Colleges, and University)	RM 5,001 - RM 10,000 per month	Employed, full time
Tertiary (including Polytechnics, MARA Colleges, University Colleges, and University)	RM 5,001 - RM 10,000 per month	Employed, full time
Secondary	RM 5,001 - RM 10,000 per month	Employed, full time
Secondary	RM 1,001 to 5,000 per month	Employed, full time
Tertiary (including Polytechnics, MARA Colleges, University Colleges, and University)	RM 1,001 to 5,000 per month	Employed, full time
Tertiary (including Polytechnics, MARA Colleges, University Colleges, and University)	RM 1,001 to 5,000 per month	Employed, full time
Tertiary (including Polytechnics, MARA Colleges, University Colleges, and University)	RM 5,001 - RM 10,000 per month	Employed, full time
Tertiary (including Polytechnics, MARA Colleges, University Colleges, and University)	RM 5,001 - RM 10,000 per month	Employed, full time
Tertiary (including Polytechnics, MARA Colleges, University Colleges, and University)	RM 5,001 - RM 10,000 per month	Employed, full time
Tertiary (including Polytechnics, MARA Colleges, University Colleges, and University)	RM 5,001 - RM 10,000 per month	Employed, full time
Tertiary (including Polytechnics, MARA Colleges, University Colleges, and University)	RM 1,001 to 5,000 per month	Work from home/housewife
Tertiary (including Polytechnics, MARA Colleges, University Colleges, and University)	>RM 10,000 per month	Employed, full time
Post-graduates	>RM 10,000 per month	Employed, full time
Tertiary (including Polytechnics, MARA Colleges, University Colleges, and University)	RM 5,001 - RM 10,000 per month	Work from home/housewife
Secondary	RM 1,001 to 5,000 per month	Employed, full time
Secondary	RM 5,001 - RM 10,000 per month	Employed, full time
Tertiary (including Polytechnics, MARA Colleges, University Colleges, and University)	RM 5,001 - RM 10,000 per month	Work from home/housewife
Tertiary (including Polytechnics, MARA Colleges, University Colleges, and University)	RM 5,001 - RM 10,000 per month	Employed, full time
Tertiary (including Polytechnics, MARA Colleges, University Colleges, and University)	RM 5,001 - RM 10,000 per month	Employed, full time
Tertiary (including Polytechnics, MARA Colleges, University Colleges, and University)	RM 5,001 - RM 10,000 per month	Employed, full time
Tertiary (including Polytechnics, MARA Colleges, University Colleges, and University)	RM 5,001 - RM 10,000 per month	Employed, full time
Secondary	>RM 10,000 per month	Employed, full time
Tertiary (including Polytechnics, MARA Colleges, University Colleges, and University)	RM 5,001 - RM 10,000 per month	Employed, full time
Tertiary (including Polytechnics, MARA Colleges, University Colleges, and University)	RM 1,001 to 5,000 per month	Employed, full time
Post-graduates	RM 5,001 - RM 10,000 per month	Work from home/housewife
Tertiary (including Polytechnics, MARA Colleges, University Colleges, and University)	RM 5,001 - RM 10,000 per month	Employed, full time

Post-graduates	RM 5,001 - RM 10,000 per month	Employed, full time
Tertiary (including Polytechnics, MARA Colleges, University Colleges, and University)	RM 1,001 to 5,000 per month	Employed, full time
Tertiary (including Polytechnics, MARA Colleges, University Colleges, and University)	>RM 10,000 per month	Others
Secondary	RM 1,001 to 5,000 per month	Employed, part time
Tertiary (including Polytechnics, MARA Colleges, University Colleges, and University)	>RM 10,000 per month	Employed, full time
Tertiary (including Polytechnics, MARA Colleges, University Colleges, and University)	RM 5,001 - RM 10,000 per month	Employed, full time
Tertiary (including Polytechnics, MARA Colleges, University Colleges, and University)	>RM 10,000 per month	Employed, full time
Tertiary (including Polytechnics, MARA Colleges, University Colleges, and University)	RM 5,001 - RM 10,000 per month	Employed, full time
Tertiary (including Polytechnics, MARA Colleges, University Colleges, and University)	>RM 10,000 per month	Employed, full time
Tertiary (including Polytechnics, MARA Colleges, University Colleges, and University)	>RM 10,000 per month	Employed, full time
Post-graduates	RM 1,001 to 5,000 per month	Temporarily unemployed/medical leave/maternity
Tertiary (including Polytechnics, MARA Colleges, University Colleges, and University)	>RM 10,000 per month	Employed, full time
Tertiary (including Polytechnics, MARA Colleges, University Colleges, and University)	RM 5,001 - RM 10,000 per month	Employed, full time
Tertiary (including Polytechnics, MARA Colleges, University Colleges, and University)	RM 5,001 - RM 10,000 per month	Employed, full time
Secondary	RM 5,001 - RM 10,000 per month	Employed, full time
Tertiary (including Polytechnics, MARA Colleges, University Colleges, and University)	RM 5,001 - RM 10,000 per month	Employed, full time
Tertiary (including Polytechnics, MARA Colleges, University Colleges, and University)	>RM 10,000 per month	Employed, full time
Tertiary (including Polytechnics, MARA Colleges, University Colleges, and University)	RM 1,001 to 5,000 per month	Work from home/housewife
Tertiary (including Polytechnics, MARA Colleges, University Colleges, and University)	RM 5,001 - RM 10,000 per month	Employed, full time
Tertiary (including Polytechnics, MARA Colleges, University Colleges, and University)	RM 1,001 to 5,000 per month	Employed, full time
Tertiary (including Polytechnics, MARA Colleges, University Colleges, and University)	RM 5,001 - RM 10,000 per month	Employed, full time
Tertiary (including Polytechnics, MARA Colleges, University Colleges, and University)	RM 5,001 - RM 10,000 per month	Employed, full time
Tertiary (including Polytechnics, MARA Colleges, University Colleges, and University)	>RM 10,000 per month	Temporarily unemployed/medical leave/maternity
Tertiary (including Polytechnics, MARA Colleges, University Colleges, and University)	RM 1,001 to 5,000 per month	Employed, full time
Tertiary (including Polytechnics, MARA Colleges, University Colleges, and University)	>RM 10,000 per month	Employed, full time
Tertiary (including Polytechnics, MARA Colleges, University Colleges, and University)	RM 5,001 - RM 10,000 per month	Employed, full time
Tertiary (including Polytechnics, MARA Colleges, University Colleges, and University)	RM 5,001 - RM 10,000 per month	Employed, full time
Tertiary (including Polytechnics, MARA Colleges, University Colleges, and University)	RM 5,001 - RM 10,000 per month	Employed, full time
Tertiary (including Polytechnics, MARA Colleges, University Colleges, and University)	RM 5,001 - RM 10,000 per month	Temporarily unemployed/medical leave/maternity
Secondary	RM 5,001 - RM 10,000 per month	Employed, full time
Tertiary (including Polytechnics, MARA Colleges, University Colleges, and University)	RM 1,001 to 5,000 per month	Employed, full time
Tertiary (including Polytechnics, MARA Colleges, University Colleges, and University)	RM 5,001 - RM 10,000 per month	Employed, full time
Secondary	RM 5,001 - RM 10,000 per month	Employed, full time

Tertiary (including Polytechnics, MARA Colleges, University Colleges, and University)	RM 5,001 - RM 10,000 per month	Employed, full time
Tertiary (including Polytechnics, MARA Colleges, University Colleges, and University)	RM 5,001 - RM 10,000 per month	Employed, full time
Secondary	RM 1,001 to 5,000 per month	Work from home/housewife
Secondary	RM 1,001 to 5,000 per month	Work from home/housewife
Tertiary (including Polytechnics, MARA Colleges, University Colleges, and University)	RM 5,001 - RM 10,000 per month	Employed, full time
Tertiary (including Polytechnics, MARA Colleges, University Colleges, and University)	RM 5,001 - RM 10,000 per month	Employed, full time
Secondary	RM 1,001 to 5,000 per month	Employed, full time
Secondary	RM 1,001 to 5,000 per month	Employed, full time
Tertiary (including Polytechnics, MARA Colleges, University Colleges, and University)	RM 1,001 to 5,000 per month	Work from home/housewife
Tertiary (including Polytechnics, MARA Colleges, University Colleges, and University)	RM 5,001 - RM 10,000 per month	Employed, full time
Tertiary (including Polytechnics, MARA Colleges, University Colleges, and University)	RM 1,001 to 5,000 per month	Employed, full time
Tertiary (including Polytechnics, MARA Colleges, University Colleges, and University)	>RM 10,000 per month	Work from home/housewife
Tertiary (including Polytechnics, MARA Colleges, University Colleges, and University)	RM 5,001 - RM 10,000 per month	Employed, full time
Secondary	RM 1,001 to 5,000 per month	Work from home/housewife
Tertiary (including Polytechnics, MARA Colleges, University Colleges, and University)	RM 5,001 - RM 10,000 per month	Employed, full time
Tertiary (including Polytechnics, MARA Colleges, University Colleges, and University)	RM 5,001 - RM 10,000 per month	Work from home/housewife
Secondary	RM 1,001 to 5,000 per month	Work from home/housewife
Tertiary (including Polytechnics, MARA Colleges, University Colleges, and University)	RM 5,001 - RM 10,000 per month	Temporarily unemployed/medical leave/maternity
Post-graduates	RM 5,001 - RM 10,000 per month	Temporarily unemployed/medical leave/maternity
Tertiary (including Polytechnics, MARA Colleges, University Colleges, and University)	RM 5,001 - RM 10,000 per month	Employed, full time
Tertiary (including Polytechnics, MARA Colleges, University Colleges, and University)	RM 1,001 to 5,000 per month	Work from home/housewife
Tertiary (including Polytechnics, MARA Colleges, University Colleges, and University)	RM 1,001 to 5,000 per month	Employed, full time
Tertiary (including Polytechnics, MARA Colleges, University Colleges, and University)	RM 1,001 to 5,000 per month	Work from home/housewife
Tertiary (including Polytechnics, MARA Colleges, University Colleges, and University)	RM 5,001 - RM 10,000 per month	Employed, full time
Secondary	RM 5,001 - RM 10,000 per month	Employed, full time
Tertiary (including Polytechnics, MARA Colleges, University Colleges, and University)	RM 1,001 to 5,000 per month	Work from home/housewife
Secondary	RM 1,001 to 5,000 per month	Work from home/housewife
Secondary	RM 1,001 to 5,000 per month	Employed, full time
Tertiary (including Polytechnics, MARA Colleges, University Colleges, and University)	RM 5,001 - RM 10,000 per month	Work from home/housewife
Tertiary (including Polytechnics, MARA Colleges, University Colleges, and University)	RM 5,001 - RM 10,000 per month	Employed, full time
Tertiary (including Polytechnics, MARA Colleges, University Colleges, and University)	RM 5,001 - RM 10,000 per month	Employed, full time
Tertiary (including Polytechnics, MARA Colleges, University Colleges, and University)	RM 1,001 to 5,000 per month	Work from home/housewife
Tertiary (including Polytechnics, MARA Colleges, University Colleges, and University)	RM 5,001 - RM 10,000 per month	Others

Tertiary (including Polytechnics, MARA Colleges, University Colleges, and University)	RM 1,001 to 5,000 per month	Work from home/housewife
Tertiary (including Polytechnics, MARA Colleges, University Colleges, and University)	RM 1,001 to 5,000 per month	Employed, full time
Tertiary (including Polytechnics, MARA Colleges, University Colleges, and University)	RM 5,001 - RM 10,000 per month	Employed, full time
Tertiary (including Polytechnics, MARA Colleges, University Colleges, and University)	RM 1,001 to 5,000 per month	Employed, full time
Tertiary (including Polytechnics, MARA Colleges, University Colleges, and University)	>RM 10,000 per month	Employed, full time
Tertiary (including Polytechnics, MARA Colleges, University Colleges, and University)	RM 5,001 - RM 10,000 per month	Employed, full time
Post-graduates	RM 5,001 - RM 10,000 per month	Employed, full time
Secondary	RM 1,001 to 5,000 per month	Employed, full time
Tertiary (including Polytechnics, MARA Colleges, University Colleges, and University)	RM 5,001 - RM 10,000 per month	Employed, full time
Secondary	RM 1,001 to 5,000 per month	Work from home/housewife
Secondary	RM 5,001 - RM 10,000 per month	Employed, full time
Tertiary (including Polytechnics, MARA Colleges, University Colleges, and University)	RM 1,001 to 5,000 per month	Work from home/housewife
Tertiary (including Polytechnics, MARA Colleges, University Colleges, and University)	RM 5,001 - RM 10,000 per month	Employed, full time
Secondary	RM 1,001 to 5,000 per month	Work from home/housewife
Tertiary (including Polytechnics, MARA Colleges, University Colleges, and University)	RM 5,001 - RM 10,000 per month	Employed, full time
Post-graduates	RM 5,001 - RM 10,000 per month	Employed, full time
Secondary	RM 1,001 to 5,000 per month	Work from home/housewife
Tertiary (including Polytechnics, MARA Colleges, University Colleges, and University)	RM 1,001 to 5,000 per month	Work from home/housewife
Tertiary (including Polytechnics, MARA Colleges, University Colleges, and University)	RM 1,001 to 5,000 per month	Employed, full time
Tertiary (including Polytechnics, MARA Colleges, University Colleges, and University)	RM 5,001 - RM 10,000 per month	Employed, full time
Tertiary (including Polytechnics, MARA Colleges, University Colleges, and University)	RM 5,001 - RM 10,000 per month	Employed, full time
Tertiary (including Polytechnics, MARA Colleges, University Colleges, and University)	RM 5,001 - RM 10,000 per month	Employed, full time
Post-graduates	RM 5,001 - RM 10,000 per month	Employed, full time
Tertiary (including Polytechnics, MARA Colleges, University Colleges, and University)	RM 5,001 - RM 10,000 per month	Employed, full time
Tertiary (including Polytechnics, MARA Colleges, University Colleges, and University)	RM 5,001 - RM 10,000 per month	Employed, full time
Tertiary (including Polytechnics, MARA Colleges, University Colleges, and University)	RM 1,001 to 5,000 per month	Employed, full time
Tertiary (including Polytechnics, MARA Colleges, University Colleges, and University)	RM 5,001 - RM 10,000 per month	Employed, full time
Tertiary (including Polytechnics, MARA Colleges, University Colleges, and University)	>RM 10,000 per month	Employed, full time
Tertiary (including Polytechnics, MARA Colleges, University Colleges, and University)	RM 5,001 - RM 10,000 per month	Employed, full time
Tertiary (including Polytechnics, MARA Colleges, University Colleges, and University)	>RM 10,000 per month	Employed, full time
Tertiary (including Polytechnics, MARA Colleges, University Colleges, and University)	RM 5,001 - RM 10,000 per month	Employed, full time
Secondary	RM 1,001 to 5,000 per month	Employed, full time
Tertiary (including Polytechnics, MARA Colleges, University Colleges, and University)	RM 5,001 - RM 10,000 per month	Employed, full time

Secondary	RM 1,001 to 5,000 per month	Work from home/housewife
Post-graduates	RM 1,001 to 5,000 per month	Employed, full time
Post-graduates	RM 1,001 to 5,000 per month	Employed, full time
Tertiary (including Polytechnics, MARA Colleges, University Colleges, and University)	RM 5,001 - RM 10,000 per month	Employed, full time
Tertiary (including Polytechnics, MARA Colleges, University Colleges, and University)	RM 5,001 - RM 10,000 per month	Employed, full time
Tertiary (including Polytechnics, MARA Colleges, University Colleges, and University)	RM 5,001 - RM 10,000 per month	Employed, full time
Post-graduates	RM 5,001 - RM 10,000 per month	Employed, full time
Tertiary (including Polytechnics, MARA Colleges, University Colleges, and University)	RM 5,001 - RM 10,000 per month	Employed, full time
Secondary	RM 5,001 - RM 10,000 per month	Employed, full time
Tertiary (including Polytechnics, MARA Colleges, University Colleges, and University)	RM 5,001 - RM 10,000 per month	Employed, full time
Tertiary (including Polytechnics, MARA Colleges, University Colleges, and University)	RM 5,001 - RM 10,000 per month	Employed, full time
Secondary	RM 1,001 to 5,000 per month	Work from home/housewife
Tertiary (including Polytechnics, MARA Colleges, University Colleges, and University)	>RM 10,000 per month	Employed, full time
Post-graduates	RM 5,001 - RM 10,000 per month	Employed, full time
Tertiary (including Polytechnics, MARA Colleges, University Colleges, and University)	RM 5,001 - RM 10,000 per month	Employed, full time
Tertiary (including Polytechnics, MARA Colleges, University Colleges, and University)	RM 1,001 to 5,000 per month	Employed, full time
Tertiary (including Polytechnics, MARA Colleges, University Colleges, and University)	RM 1,001 to 5,000 per month	Work from home/housewife
Tertiary (including Polytechnics, MARA Colleges, University Colleges, and University)	RM 1,001 to 5,000 per month	Employed, full time
Tertiary (including Polytechnics, MARA Colleges, University Colleges, and University)	>RM 10,000 per month	Employed, full time
Tertiary (including Polytechnics, MARA Colleges, University Colleges, and University)	RM 1,001 to 5,000 per month	Employed, full time
Tertiary (including Polytechnics, MARA Colleges, University Colleges, and University)	RM 1,001 to 5,000 per month	Work from home/housewife
Secondary	RM 1,001 to 5,000 per month	Employed, full time
Tertiary (including Polytechnics, MARA Colleges, University Colleges, and University)	RM 5,001 - RM 10,000 per month	Employed, full time
Secondary	RM 1,001 to 5,000 per month	Employed, full time
Tertiary (including Polytechnics, MARA Colleges, University Colleges, and University)	RM 1,001 to 5,000 per month	Employed, full time
Tertiary (including Polytechnics, MARA Colleges, University Colleges, and University)	RM 5,001 - RM 10,000 per month	Employed, full time
Tertiary (including Polytechnics, MARA Colleges, University Colleges, and University)	>RM 10,000 per month	Employed, full time
Tertiary (including Polytechnics, MARA Colleges, University Colleges, and University)	RM 5,001 - RM 10,000 per month	Employed, full time
Secondary	>RM 10,000 per month	Employed, full time
Tertiary (including Polytechnics, MARA Colleges, University Colleges, and University)	RM 5,001 - RM 10,000 per month	Others
Tertiary (including Polytechnics, MARA Colleges, University Colleges, and University)	RM 1,001 to 5,000 per month	Employed, full time
Tertiary (including Polytechnics, MARA Colleges, University Colleges, and University)	>RM 10,000 per month	Employed, full time
Tertiary (including Polytechnics, MARA Colleges, University Colleges, and University)	>RM 10,000 per month	Employed, full time

Post-graduates	RM 5,001 - RM 10,000 per month	Employed, full time
Secondary	RM 5,001 - RM 10,000 per month	Employed, full time
Tertiary (including Polytechnics, MARA Colleges, University Colleges, and University)	RM 5,001 - RM 10,000 per month	Employed, full time
Tertiary (including Polytechnics, MARA Colleges, University Colleges, and University)	RM 1,001 to 5,000 per month	Employed, full time
Post-graduates	>RM 10,000 per month	Employed, full time
Tertiary (including Polytechnics, MARA Colleges, University Colleges, and University)	RM 5,001 - RM 10,000 per month	Employed, full time
Tertiary (including Polytechnics, MARA Colleges, University Colleges, and University)	RM 5,001 - RM 10,000 per month	Employed, full time
Tertiary (including Polytechnics, MARA Colleges, University Colleges, and University)	RM 5,001 - RM 10,000 per month	Employed, full time
Tertiary (including Polytechnics, MARA Colleges, University Colleges, and University)	RM 1,001 to 5,000 per month	Employed, full time
Tertiary (including Polytechnics, MARA Colleges, University Colleges, and University)	RM 1,001 to 5,000 per month	Work from home/housewife
Tertiary (including Polytechnics, MARA Colleges, University Colleges, and University)	RM 5,001 - RM 10,000 per month	Employed, full time
Tertiary (including Polytechnics, MARA Colleges, University Colleges, and University)	RM 1,001 to 5,000 per month	Employed, full time
Tertiary (including Polytechnics, MARA Colleges, University Colleges, and University)	>RM 10,000 per month	Employed, full time
Tertiary (including Polytechnics, MARA Colleges, University Colleges, and University)	RM 5,001 - RM 10,000 per month	Employed, full time
Tertiary (including Polytechnics, MARA Colleges, University Colleges, and University)	RM 5,001 - RM 10,000 per month	Employed, full time
Tertiary (including Polytechnics, MARA Colleges, University Colleges, and University)	RM 5,001 - RM 10,000 per month	Employed, full time
Secondary	>RM 10,000 per month	Work from home/housewife
Tertiary (including Polytechnics, MARA Colleges, University Colleges, and University)	RM 5,001 - RM 10,000 per month	Work from home/housewife
Secondary	RM 1,001 to 5,000 per month	Work from home/housewife
Tertiary (including Polytechnics, MARA Colleges, University Colleges, and University)	RM 1,001 to 5,000 per month	Work from home/housewife
Tertiary (including Polytechnics, MARA Colleges, University Colleges, and University)	RM 1,001 to 5,000 per month	Employed, full time
Post-graduates	RM 5,001 - RM 10,000 per month	Employed, full time
Tertiary (including Polytechnics, MARA Colleges, University Colleges, and University)	RM 5,001 - RM 10,000 per month	Employed, full time
Tertiary (including Polytechnics, MARA Colleges, University Colleges, and University)	RM 5,001 - RM 10,000 per month	Employed, full time
Tertiary (including Polytechnics, MARA Colleges, University Colleges, and University)	RM 5,001 - RM 10,000 per month	Employed, full time
Tertiary (including Polytechnics, MARA Colleges, University Colleges, and University)	>RM 10,000 per month	Others
Tertiary (including Polytechnics, MARA Colleges, University Colleges, and University)	RM 1,001 to 5,000 per month	Employed, full time
Tertiary (including Polytechnics, MARA Colleges, University Colleges, and University)	RM 5,001 - RM 10,000 per month	Employed, full time
Secondary	>RM 10,000 per month	Employed, full time
Post-graduates	>RM 10,000 per month	Employed, full time
Secondary	RM 1,001 to 5,000 per month	Employed, full time
Tertiary (including Polytechnics, MARA Colleges, University Colleges, and University)	RM 1,001 to 5,000 per month	Employed, full time
Secondary	RM 5,001 - RM 10,000 per month	Employed, full time

Tertiary (including Polytechnics, MARA Colleges, University Colleges, and University)	RM 5,001 - RM 10,000 per month	Employed, full time
Tertiary (including Polytechnics, MARA Colleges, University Colleges, and University)	RM 1,001 to 5,000 per month	Work from home/housewife
Secondary	RM 1,001 to 5,000 per month	Employed, full time
Tertiary (including Polytechnics, MARA Colleges, University Colleges, and University)	RM 1,001 to 5,000 per month	Work from home/housewife
Tertiary (including Polytechnics, MARA Colleges, University Colleges, and University)	RM 1,001 to 5,000 per month	Employed, full time
Tertiary (including Polytechnics, MARA Colleges, University Colleges, and University)	RM 1,001 to 5,000 per month	Work from home/housewife
Secondary	RM 1,001 to 5,000 per month	Employed, full time
Secondary	RM 1,001 to 5,000 per month	Employed, full time
Secondary	RM 1,001 to 5,000 per month	Work from home/housewife
Secondary	RM 5,001 - RM 10,000 per month	Employed, full time
Tertiary (including Polytechnics, MARA Colleges, University Colleges, and University)	RM 5,001 - RM 10,000 per month	Employed, full time
Post-graduates	>RM 10,000 per month	Employed, full time
Tertiary (including Polytechnics, MARA Colleges, University Colleges, and University)	RM 1,001 to 5,000 per month	Employed, full time
Tertiary (including Polytechnics, MARA Colleges, University Colleges, and University)	RM 5,001 - RM 10,000 per month	Employed, full time
Tertiary (including Polytechnics, MARA Colleges, University Colleges, and University)	RM 5,001 - RM 10,000 per month	Employed, part time
Tertiary (including Polytechnics, MARA Colleges, University Colleges, and University)	>RM 10,000 per month	Work from home/housewife
Tertiary (including Polytechnics, MARA Colleges, University Colleges, and University)	RM 5,001 - RM 10,000 per month	Employed, full time
Tertiary (including Polytechnics, MARA Colleges, University Colleges, and University)	>RM 10,000 per month	Employed, full time
Tertiary (including Polytechnics, MARA Colleges, University Colleges, and University)	RM 5,001 - RM 10,000 per month	Employed, full time
Secondary	RM 1,001 to 5,000 per month	Work from home/housewife
Tertiary (including Polytechnics, MARA Colleges, University Colleges, and University)	RM 5,001 - RM 10,000 per month	Employed, full time
Tertiary (including Polytechnics, MARA Colleges, University Colleges, and University)	RM 5,001 - RM 10,000 per month	Employed, full time
Tertiary (including Polytechnics, MARA Colleges, University Colleges, and University)	RM 1,001 to 5,000 per month	Employed, part time
Tertiary (including Polytechnics, MARA Colleges, University Colleges, and University)	>RM 10,000 per month	Employed, full time
Tertiary (including Polytechnics, MARA Colleges, University Colleges, and University)	RM 5,001 - RM 10,000 per month	Employed, full time
Tertiary (including Polytechnics, MARA Colleges, University Colleges, and University)	RM 5,001 - RM 10,000 per month	Employed, full time
Tertiary (including Polytechnics, MARA Colleges, University Colleges, and University)	>RM 10,000 per month	Employed, full time
Tertiary (including Polytechnics, MARA Colleges, University Colleges, and University)	>RM 10,000 per month	Employed, full time
Secondary	RM 1,001 to 5,000 per month	Temporarily unemployed/medical leave/maternity
Tertiary (including Polytechnics, MARA Colleges, University Colleges, and University)	RM 5,001 - RM 10,000 per month	Employed, full time
Tertiary (including Polytechnics, MARA Colleges, University Colleges, and University)	RM 5,001 - RM 10,000 per month	Work from home/housewife
Post-graduates	>RM 10,000 per month	Employed, full time
Tertiary (including Polytechnics, MARA Colleges, University Colleges, and University)	RM 5,001 - RM 10,000 per month	Temporarily unemployed/medical leave/maternity

Tertiary (including Polytechnics, MARA Colleges, University Colleges, and University)	RM 5,001 - RM 10,000 per month	Employed, part time
Tertiary (including Polytechnics, MARA Colleges, University Colleges, and University)	RM 1,001 to 5,000 per month	Work from home/housewife
Secondary	RM 1,001 to 5,000 per month	Employed, full time
Post-graduates	>RM 10,000 per month	Employed, full time
Post-graduates	RM 5,001 - RM 10,000 per month	Temporarily unemployed/medical leave/maternity
Secondary	RM 5,001 - RM 10,000 per month	Employed, full time
Tertiary (including Polytechnics, MARA Colleges, University Colleges, and University)	RM 5,001 - RM 10,000 per month	Work from home/housewife
Post-graduates	>RM 10,000 per month	Employed, full time
Tertiary (including Polytechnics, MARA Colleges, University Colleges, and University)	RM 5,001 - RM 10,000 per month	Employed, full time
Tertiary (including Polytechnics, MARA Colleges, University Colleges, and University)	RM 5,001 - RM 10,000 per month	Employed, full time
Tertiary (including Polytechnics, MARA Colleges, University Colleges, and University)	RM 5,001 - RM 10,000 per month	Employed, full time
Tertiary (including Polytechnics, MARA Colleges, University Colleges, and University)	>RM 10,000 per month	Employed, full time
Tertiary (including Polytechnics, MARA Colleges, University Colleges, and University)	RM 5,001 - RM 10,000 per month	Employed, full time
Tertiary (including Polytechnics, MARA Colleges, University Colleges, and University)	RM 5,001 - RM 10,000 per month	Employed, full time
Tertiary (including Polytechnics, MARA Colleges, University Colleges, and University)	RM 5,001 - RM 10,000 per month	Employed, full time
Tertiary (including Polytechnics, MARA Colleges, University Colleges, and University)	>RM 10,000 per month	Employed, full time
Tertiary (including Polytechnics, MARA Colleges, University Colleges, and University)	RM 5,001 - RM 10,000 per month	Employed, full time
Tertiary (including Polytechnics, MARA Colleges, University Colleges, and University)	RM 1,001 to 5,000 per month	Employed, full time
Tertiary (including Polytechnics, MARA Colleges, University Colleges, and University)	RM 1,001 to 5,000 per month	Employed, full time
Tertiary (including Polytechnics, MARA Colleges, University Colleges, and University)	RM 1,001 to 5,000 per month	Employed, full time
Tertiary (including Polytechnics, MARA Colleges, University Colleges, and University)	RM 1,001 to 5,000 per month	Employed, full time
Tertiary (including Polytechnics, MARA Colleges, University Colleges, and University)	RM 5,001 - RM 10,000 per month	Employed, full time
Secondary	RM 5,001 - RM 10,000 per month	Temporarily unemployed/medical leave/maternity
Secondary	RM 1,001 to 5,000 per month	Work from home/housewife
Tertiary (including Polytechnics, MARA Colleges, University Colleges, and University)	RM 5,001 - RM 10,000 per month	Employed, full time
Secondary	RM 5,001 - RM 10,000 per month	Work from home/housewife
Tertiary (including Polytechnics, MARA Colleges, University Colleges, and University)	RM 5,001 - RM 10,000 per month	Employed, full time
Tertiary (including Polytechnics, MARA Colleges, University Colleges, and University)	RM 5,001 - RM 10,000 per month	Employed, full time
Tertiary (including Polytechnics, MARA Colleges, University Colleges, and University)	RM 1,001 to 5,000 per month	Employed, full time
Secondary	RM 1,001 to 5,000 per month	Work from home/housewife
Tertiary (including Polytechnics, MARA Colleges, University Colleges, and University)	RM 1,001 to 5,000 per month	Employed, full time
Tertiary (including Polytechnics, MARA Colleges, University Colleges, and University)	RM 5,001 - RM 10,000 per month	Employed, full time
Tertiary (including Polytechnics, MARA Colleges, University Colleges, and University)	RM 5,001 - RM 10,000 per month	Employed, full time

Secondary	RM 1,001 to 5,000 per month	Work from home/housewife
Secondary	RM 1,001 to 5,000 per month	Employed, full time
Secondary	RM 5,001 - RM 10,000 per month	Employed, full time
Tertiary (including Polytechnics, MARA Colleges, University Colleges, and University)	RM 1,001 to 5,000 per month	Employed, full time
Tertiary (including Polytechnics, MARA Colleges, University Colleges, and University)	RM 1,001 to 5,000 per month	Employed, part time
Tertiary (including Polytechnics, MARA Colleges, University Colleges, and University)	>RM 10,000 per month	Employed, full time
Tertiary (including Polytechnics, MARA Colleges, University Colleges, and University)	RM 5,001 - RM 10,000 per month	Employed, full time
Tertiary (including Polytechnics, MARA Colleges, University Colleges, and University)	>RM 10,000 per month	Employed, full time
Post-graduates	RM 1,001 to 5,000 per month	Work from home/housewife
Tertiary (including Polytechnics, MARA Colleges, University Colleges, and University)	>RM 10,000 per month	Employed, full time
Tertiary (including Polytechnics, MARA Colleges, University Colleges, and University)	RM 5,001 - RM 10,000 per month	Employed, full time
Tertiary (including Polytechnics, MARA Colleges, University Colleges, and University)	RM 5,001 - RM 10,000 per month	Employed, full time
Tertiary (including Polytechnics, MARA Colleges, University Colleges, and University)	>RM 10,000 per month	Employed, full time
Tertiary (including Polytechnics, MARA Colleges, University Colleges, and University)	RM 5,001 - RM 10,000 per month	Employed, full time
Tertiary (including Polytechnics, MARA Colleges, University Colleges, and University)	RM 5,001 - RM 10,000 per month	Employed, full time
Tertiary (including Polytechnics, MARA Colleges, University Colleges, and University)	RM 5,001 - RM 10,000 per month	Employed, full time
Secondary	>RM 10,000 per month	Temporarily unemployed/medical leave/maternity
Tertiary (including Polytechnics, MARA Colleges, University Colleges, and University)	RM 5,001 - RM 10,000 per month	Employed, full time
Tertiary (including Polytechnics, MARA Colleges, University Colleges, and University)	RM 1,001 to 5,000 per month	Employed, full time
Tertiary (including Polytechnics, MARA Colleges, University Colleges, and University)	RM 5,001 - RM 10,000 per month	Work from home/housewife
Post-graduates	RM 5,001 - RM 10,000 per month	Employed, full time
Tertiary (including Polytechnics, MARA Colleges, University Colleges, and University)	RM 1,001 to 5,000 per month	Work from home/housewife
Post-graduates	RM 5,001 - RM 10,000 per month	Employed, full time
Tertiary (including Polytechnics, MARA Colleges, University Colleges, and University)	RM 5,001 - RM 10,000 per month	Employed, full time
Post-graduates	>RM 10,000 per month	Employed, full time
Tertiary (including Polytechnics, MARA Colleges, University Colleges, and University)	RM 5,001 - RM 10,000 per month	Employed, full time
Secondary	RM 1,001 to 5,000 per month	Employed, full time
Tertiary (including Polytechnics, MARA Colleges, University Colleges, and University)	RM 1,001 to 5,000 per month	Employed, full time
Tertiary (including Polytechnics, MARA Colleges, University Colleges, and University)	RM 5,001 - RM 10,000 per month	Employed, full time
Tertiary (including Polytechnics, MARA Colleges, University Colleges, and University)	RM 5,001 - RM 10,000 per month	Employed, full time
Secondary	RM 5,001 - RM 10,000 per month	Work from home/housewife
Tertiary (including Polytechnics, MARA Colleges, University Colleges, and University)	RM 5,001 - RM 10,000 per month	Employed, full time
Tertiary (including Polytechnics, MARA Colleges, University Colleges, and University)	RM 5,001 - RM 10,000 per month	Work from home/housewife

Tertiary (including Polytechnics, MARA Colleges, University Colleges, and University)	>RM 10,000 per month	Employed, full time
Secondary	RM 1,001 to 5,000 per month	Work from home/housewife
Tertiary (including Polytechnics, MARA Colleges, University Colleges, and University)	RM 5,001 - RM 10,000 per month	Employed, full time
Secondary	RM 5,001 - RM 10,000 per month	Employed, full time
Tertiary (including Polytechnics, MARA Colleges, University Colleges, and University)	>RM 10,000 per month	Employed, full time
Tertiary (including Polytechnics, MARA Colleges, University Colleges, and University)	RM 5,001 - RM 10,000 per month	Employed, full time
Tertiary (including Polytechnics, MARA Colleges, University Colleges, and University)	RM 5,001 - RM 10,000 per month	Employed, full time
Tertiary (including Polytechnics, MARA Colleges, University Colleges, and University)	RM 5,001 - RM 10,000 per month	Work from home/housewife
Tertiary (including Polytechnics, MARA Colleges, University Colleges, and University)	RM 5,001 - RM 10,000 per month	Employed, full time
Tertiary (including Polytechnics, MARA Colleges, University Colleges, and University)	>RM 10,000 per month	Temporarily unemployed/medical leave/maternity
Post-graduates	RM 5,001 - RM 10,000 per month	Employed, full time
Tertiary (including Polytechnics, MARA Colleges, University Colleges, and University)	RM 5,001 - RM 10,000 per month	Employed, full time
Tertiary (including Polytechnics, MARA Colleges, University Colleges, and University)	RM 1,001 to 5,000 per month	Work from home/housewife
Secondary	RM 1,001 to 5,000 per month	Work from home/housewife
Tertiary (including Polytechnics, MARA Colleges, University Colleges, and University)	>RM 10,000 per month	Employed, full time
Tertiary (including Polytechnics, MARA Colleges, University Colleges, and University)	>RM 10,000 per month	Employed, full time
Tertiary (including Polytechnics, MARA Colleges, University Colleges, and University)	>RM 10,000 per month	Employed, full time
Tertiary (including Polytechnics, MARA Colleges, University Colleges, and University)	RM 1,001 to 5,000 per month	Employed, full time
Tertiary (including Polytechnics, MARA Colleges, University Colleges, and University)	>RM 10,000 per month	Employed, full time
Tertiary (including Polytechnics, MARA Colleges, University Colleges, and University)	RM 1,001 to 5,000 per month	Employed, full time
Secondary	RM 1,001 to 5,000 per month	Employed, full time
Tertiary (including Polytechnics, MARA Colleges, University Colleges, and University)	RM 1,001 to 5,000 per month	Employed, full time
Tertiary (including Polytechnics, MARA Colleges, University Colleges, and University)	RM 5,001 - RM 10,000 per month	Employed, full time
Post-graduates	>RM 10,000 per month	Employed, full time
Post-graduates	RM 5,001 - RM 10,000 per month	Employed, full time
Tertiary (including Polytechnics, MARA Colleges, University Colleges, and University)	>RM 10,000 per month	Employed, full time
Tertiary (including Polytechnics, MARA Colleges, University Colleges, and University)	RM 5,001 - RM 10,000 per month	Employed, full time
Tertiary (including Polytechnics, MARA Colleges, University Colleges, and University)	RM 5,001 - RM 10,000 per month	Work from home/housewife
Tertiary (including Polytechnics, MARA Colleges, University Colleges, and University)	>RM 10,000 per month	Employed, full time
Tertiary (including Polytechnics, MARA Colleges, University Colleges, and University)	RM 1,001 to 5,000 per month	Work from home/housewife
Tertiary (including Polytechnics, MARA Colleges, University Colleges, and University)	>RM 10,000 per month	Work from home/housewife
Secondary	RM 5,001 - RM 10,000 per month	Employed, full time
Tertiary (including Polytechnics, MARA Colleges, University Colleges, and University)	RM 5,001 - RM 10,000 per month	Employed, full time

Tertiary (including Polytechnics, MARA Colleges, University Colleges, and University)	>RM 10,000 per month	Employed, full time
Tertiary (including Polytechnics, MARA Colleges, University Colleges, and University)	RM 5,001 - RM 10,000 per month	Employed, full time
Tertiary (including Polytechnics, MARA Colleges, University Colleges, and University)	RM 5,001 - RM 10,000 per month	Employed, full time
Tertiary (including Polytechnics, MARA Colleges, University Colleges, and University)	RM 5,001 - RM 10,000 per month	Employed, full time
Tertiary (including Polytechnics, MARA Colleges, University Colleges, and University)	RM 5,001 - RM 10,000 per month	Employed, full time
Secondary	RM 5,001 - RM 10,000 per month	Temporarily unemployed/medical leave/maternity
Secondary	RM 5,001 - RM 10,000 per month	Employed, full time
Secondary	RM 5,001 - RM 10,000 per month	Employed, full time
Tertiary (including Polytechnics, MARA Colleges, University Colleges, and University)	RM 1,001 to 5,000 per month	Employed, full time
Tertiary (including Polytechnics, MARA Colleges, University Colleges, and University)	RM 5,001 - RM 10,000 per month	Employed, full time
Tertiary (including Polytechnics, MARA Colleges, University Colleges, and University)	RM 1,001 to 5,000 per month	Employed, full time
Tertiary (including Polytechnics, MARA Colleges, University Colleges, and University)	RM 5,001 - RM 10,000 per month	Employed, full time
Tertiary (including Polytechnics, MARA Colleges, University Colleges, and University)	RM 5,001 - RM 10,000 per month	Employed, full time
Tertiary (including Polytechnics, MARA Colleges, University Colleges, and University)	>RM 10,000 per month	Employed, full time
Tertiary (including Polytechnics, MARA Colleges, University Colleges, and University)	RM 5,001 - RM 10,000 per month	Employed, full time
Tertiary (including Polytechnics, MARA Colleges, University Colleges, and University)	>RM 10,000 per month	Work from home/housewife
Tertiary (including Polytechnics, MARA Colleges, University Colleges, and University)	RM 5,001 - RM 10,000 per month	Employed, full time
Post-graduates	RM 1,001 to 5,000 per month	Work from home/housewife
Tertiary (including Polytechnics, MARA Colleges, University Colleges, and University)	RM 5,001 - RM 10,000 per month	Employed, full time
Post-graduates	RM 5,001 - RM 10,000 per month	Work from home/housewife
Tertiary (including Polytechnics, MARA Colleges, University Colleges, and University)	RM 5,001 - RM 10,000 per month	Work from home/housewife
Tertiary (including Polytechnics, MARA Colleges, University Colleges, and University)	RM 5,001 - RM 10,000 per month	Work from home/housewife
Secondary	RM 1,001 to 5,000 per month	Employed, full time
Tertiary (including Polytechnics, MARA Colleges, University Colleges, and University)	RM 1,001 to 5,000 per month	Employed, full time
Tertiary (including Polytechnics, MARA Colleges, University Colleges, and University)	RM 5,001 - RM 10,000 per month	Employed, full time
Tertiary (including Polytechnics, MARA Colleges, University Colleges, and University)	RM 5,001 - RM 10,000 per month	Employed, full time
Post-graduates	RM 5,001 - RM 10,000 per month	Employed, full time
Tertiary (including Polytechnics, MARA Colleges, University Colleges, and University)	RM 5,001 - RM 10,000 per month	Employed, full time
Tertiary (including Polytechnics, MARA Colleges, University Colleges, and University)	>RM 10,000 per month	Employed, full time
Post-graduates	RM 5,001 - RM 10,000 per month	Employed, full time
Secondary	RM 5,001 - RM 10,000 per month	Employed, full time
Secondary	RM 5,001 - RM 10,000 per month	Employed, full time
Tertiary (including Polytechnics, MARA Colleges, University Colleges, and University)	RM 5,001 - RM 10,000 per month	Employed, full time

Tertiary (including Polytechnics, MARA Colleges, University Colleges, and University)	RM 5,001 - RM 10,000 per month	Employed, full time
Tertiary (including Polytechnics, MARA Colleges, University Colleges, and University)	>RM 10,000 per month	Employed, full time
Tertiary (including Polytechnics, MARA Colleges, University Colleges, and University)	RM 5,001 - RM 10,000 per month	Employed, full time
Tertiary (including Polytechnics, MARA Colleges, University Colleges, and University)	RM 5,001 - RM 10,000 per month	Employed, full time
Secondary	RM 1,001 to 5,000 per month	Employed, full time
Tertiary (including Polytechnics, MARA Colleges, University Colleges, and University)	>RM 10,000 per month	Employed, full time
Tertiary (including Polytechnics, MARA Colleges, University Colleges, and University)	RM 5,001 - RM 10,000 per month	Employed, full time
Tertiary (including Polytechnics, MARA Colleges, University Colleges, and University)	RM 5,001 - RM 10,000 per month	Employed, full time
Secondary	RM 1,001 to 5,000 per month	Employed, full time
Tertiary (including Polytechnics, MARA Colleges, University Colleges, and University)	RM 1,001 to 5,000 per month	Employed, full time
Tertiary (including Polytechnics, MARA Colleges, University Colleges, and University)	RM 5,001 - RM 10,000 per month	Employed, full time
Tertiary (including Polytechnics, MARA Colleges, University Colleges, and University)	RM 1,001 to 5,000 per month	Work from home/housewife
Tertiary (including Polytechnics, MARA Colleges, University Colleges, and University)	RM 5,001 - RM 10,000 per month	Work from home/housewife
Secondary	RM 1,001 to 5,000 per month	Work from home/housewife
Secondary	RM 1,001 to 5,000 per month	Work from home/housewife
Tertiary (including Polytechnics, MARA Colleges, University Colleges, and University)	RM 5,001 - RM 10,000 per month	Employed, full time
Post-graduates	>RM 10,000 per month	Employed, full time
Secondary	RM 1,001 to 5,000 per month	Work from home/housewife
Secondary	>RM 10,000 per month	Employed, full time
Tertiary (including Polytechnics, MARA Colleges, University Colleges, and University)	RM 5,001 - RM 10,000 per month	Temporarily unemployed/medical leave/maternity
Post-graduates	>RM 10,000 per month	Employed, full time
Tertiary (including Polytechnics, MARA Colleges, University Colleges, and University)	>RM 10,000 per month	Employed, full time
Secondary	RM 5,001 - RM 10,000 per month	Work from home/housewife
Tertiary (including Polytechnics, MARA Colleges, University Colleges, and University)	RM 5,001 - RM 10,000 per month	Temporarily unemployed/medical leave/maternity
Tertiary (including Polytechnics, MARA Colleges, University Colleges, and University)	RM 1,001 to 5,000 per month	Employed, full time
Tertiary (including Polytechnics, MARA Colleges, University Colleges, and University)	RM 5,001 - RM 10,000 per month	Employed, full time
Tertiary (including Polytechnics, MARA Colleges, University Colleges, and University)	>RM 10,000 per month	Employed, full time
Secondary	RM 1,001 to 5,000 per month	Work from home/housewife
Tertiary (including Polytechnics, MARA Colleges, University Colleges, and University)	>RM 10,000 per month	Employed, full time
Tertiary (including Polytechnics, MARA Colleges, University Colleges, and University)		Employed, full time
Tertiary (including Polytechnics, MARA Colleges, University Colleges, and University)	RM 5,001 - RM 10,000 per month	Employed, full time
Post-graduates	RM 5,001 - RM 10,000 per month	Work from home/housewife
Tertiary (including Polytechnics, MARA Colleges, University Colleges, and University)	RM 5,001 - RM 10,000 per month	Work from home/housewife

Tertiary (including Polytechnics, MARA Colleges, University Colleges, and University)	RM 5,001 - RM 10,000 per month	Employed, full time
Tertiary (including Polytechnics, MARA Colleges, University Colleges, and University)	>RM 10,000 per month	Employed, full time
Post-graduates	RM 5,001 - RM 10,000 per month	Employed, full time
Tertiary (including Polytechnics, MARA Colleges, University Colleges, and University)	RM 5,001 - RM 10,000 per month	Employed, full time
Tertiary (including Polytechnics, MARA Colleges, University Colleges, and University)	RM 1,001 to 5,000 per month	Work from home/housewife
Tertiary (including Polytechnics, MARA Colleges, University Colleges, and University)	RM 5,001 - RM 10,000 per month	Employed, full time
Tertiary (including Polytechnics, MARA Colleges, University Colleges, and University)	RM 1,001 to 5,000 per month	Employed, full time
Secondary	RM 5,001 - RM 10,000 per month	Employed, full time
Tertiary (including Polytechnics, MARA Colleges, University Colleges, and University)	RM 5,001 - RM 10,000 per month	Employed, full time
Post-graduates	RM 5,001 - RM 10,000 per month	Employed, full time
Tertiary (including Polytechnics, MARA Colleges, University Colleges, and University)	RM 5,001 - RM 10,000 per month	Employed, full time
Tertiary (including Polytechnics, MARA Colleges, University Colleges, and University)	RM 5,001 - RM 10,000 per month	Employed, full time
Tertiary (including Polytechnics, MARA Colleges, University Colleges, and University)	RM 5,001 - RM 10,000 per month	Employed, full time
Tertiary (including Polytechnics, MARA Colleges, University Colleges, and University)	RM 5,001 - RM 10,000 per month	Temporarily unemployed/medical leave/maternity
Secondary	RM 1,001 to 5,000 per month	Work from home/housewife
Tertiary (including Polytechnics, MARA Colleges, University Colleges, and University)	RM 5,001 - RM 10,000 per month	Employed, full time
Tertiary (including Polytechnics, MARA Colleges, University Colleges, and University)	RM 1,001 to 5,000 per month	Employed, full time
Secondary	RM 5,001 - RM 10,000 per month	Work from home/housewife
Post-graduates	>RM 10,000 per month	Employed, full time
Tertiary (including Polytechnics, MARA Colleges, University Colleges, and University)	RM 5,001 - RM 10,000 per month	Employed, full time
Tertiary (including Polytechnics, MARA Colleges, University Colleges, and University)	>RM 10,000 per month	Work from home/housewife
Post-graduates	RM 5,001 - RM 10,000 per month	Employed, full time
Secondary	RM 1,001 to 5,000 per month	Employed, full time
Tertiary (including Polytechnics, MARA Colleges, University Colleges, and University)	RM 5,001 - RM 10,000 per month	Employed, full time
Tertiary (including Polytechnics, MARA Colleges, University Colleges, and University)	>RM 10,000 per month	Employed, full time
Secondary	RM 5,001 - RM 10,000 per month	Employed, full time
Secondary	RM 1,001 to 5,000 per month	Employed, full time
Tertiary (including Polytechnics, MARA Colleges, University Colleges, and University)	RM 1,001 to 5,000 per month	Employed, full time
Secondary	RM 5,001 - RM 10,000 per month	Employed, full time
Tertiary (including Polytechnics, MARA Colleges, University Colleges, and University)	RM 5,001 - RM 10,000 per month	Employed, full time
Secondary	RM 5,001 - RM 10,000 per month	Employed, full time
Tertiary (including Polytechnics, MARA Colleges, University Colleges, and University)	RM 5,001 - RM 10,000 per month	Employed, full time
Tertiary (including Polytechnics, MARA Colleges, University Colleges, and University)	RM 5,001 - RM 10,000 per month	Employed, full time

Secondary	RM 5,001 - RM 10,000 per month	Employed, full time
Tertiary (including Polytechnics, MARA Colleges, University Colleges, and University)	>RM 10,000 per month	Employed, full time
Secondary	RM 1,001 to 5,000 per month	Employed, full time
Tertiary (including Polytechnics, MARA Colleges, University Colleges, and University)	RM 5,001 - RM 10,000 per month	Employed, full time
Tertiary (including Polytechnics, MARA Colleges, University Colleges, and University)	RM 5,001 - RM 10,000 per month	Employed, full time
Tertiary (including Polytechnics, MARA Colleges, University Colleges, and University)	RM 5,001 - RM 10,000 per month	Employed, full time
Post-graduates	RM 1,001 to 5,000 per month	Employed, full time
Tertiary (including Polytechnics, MARA Colleges, University Colleges, and University)	>RM 10,000 per month	Employed, full time
Primary	>RM 10,000 per month	Work from home/housewife
Tertiary (including Polytechnics, MARA Colleges, University Colleges, and University)	RM 1,001 to 5,000 per month	Work from home/housewife
Tertiary (including Polytechnics, MARA Colleges, University Colleges, and University)	RM 5,001 - RM 10,000 per month	Employed, full time
Tertiary (including Polytechnics, MARA Colleges, University Colleges, and University)	RM 5,001 - RM 10,000 per month	Employed, full time
Tertiary (including Polytechnics, MARA Colleges, University Colleges, and University)	RM 5,001 - RM 10,000 per month	Employed, full time
Tertiary (including Polytechnics, MARA Colleges, University Colleges, and University)	RM 5,001 - RM 10,000 per month	Employed, full time
Post-graduates	>RM 10,000 per month	Employed, full time
Tertiary (including Polytechnics, MARA Colleges, University Colleges, and University)	RM 5,001 - RM 10,000 per month	Employed, full time
Secondary	RM 1,001 to 5,000 per month	Employed, full time
Secondary	RM 1,001 to 5,000 per month	Employed, full time
Tertiary (including Polytechnics, MARA Colleges, University Colleges, and University)	RM 5,001 - RM 10,000 per month	Employed, full time
Tertiary (including Polytechnics, MARA Colleges, University Colleges, and University)	RM 5,001 - RM 10,000 per month	Employed, full time
Tertiary (including Polytechnics, MARA Colleges, University Colleges, and University)	RM 5,001 - RM 10,000 per month	Employed, full time
Secondary	RM 1,001 to 5,000 per month	Work from home/housewife
Tertiary (including Polytechnics, MARA Colleges, University Colleges, and University)	RM 5,001 - RM 10,000 per month	Employed, full time
Secondary	>RM 10,000 per month	Work from home/housewife
Secondary	RM 5,001 - RM 10,000 per month	Employed, full time
Secondary	>RM 10,000 per month	Employed, full time
Tertiary (including Polytechnics, MARA Colleges, University Colleges, and University)	>RM 10,000 per month	Employed, full time
Secondary	RM 5,001 - RM 10,000 per month	Employed, full time
Secondary	RM 5,001 - RM 10,000 per month	Temporarily unemployed/medical leave/maternity
Secondary	RM 5,001 - RM 10,000 per month	Employed, full time
Tertiary (including Polytechnics, MARA Colleges, University Colleges, and University)	RM 5,001 - RM 10,000 per month	Employed, full time
Tertiary (including Polytechnics, MARA Colleges, University Colleges, and University)	RM 1,001 to 5,000 per month	Employed, full time
Tertiary (including Polytechnics, MARA Colleges, University Colleges, and University)	RM 5,001 - RM 10,000 per month	Employed, full time

Tertiary (including Polytechnics, MARA Colleges, University Colleges, and University)	RM 5,001 - RM 10,000 per month	Employed, full time
Post-graduates	RM 5,001 - RM 10,000 per month	Work from home/housewife
Tertiary (including Polytechnics, MARA Colleges, University Colleges, and University)	RM 1,001 to 5,000 per month	Employed, full time
Post-graduates	>RM 10,000 per month	Employed, full time
Tertiary (including Polytechnics, MARA Colleges, University Colleges, and University)	RM 5,001 - RM 10,000 per month	Employed, full time
Tertiary (including Polytechnics, MARA Colleges, University Colleges, and University)	RM 5,001 - RM 10,000 per month	Employed, full time
Tertiary (including Polytechnics, MARA Colleges, University Colleges, and University)	RM 5,001 - RM 10,000 per month	Employed, full time
Tertiary (including Polytechnics, MARA Colleges, University Colleges, and University)	RM 5,001 - RM 10,000 per month	Employed, full time
Tertiary (including Polytechnics, MARA Colleges, University Colleges, and University)	RM 1,001 to 5,000 per month	Employed, full time
Secondary	RM 1,001 to 5,000 per month	Work from home/housewife
Tertiary (including Polytechnics, MARA Colleges, University Colleges, and University)	RM 5,001 - RM 10,000 per month	Employed, full time
Tertiary (including Polytechnics, MARA Colleges, University Colleges, and University)	>RM 10,000 per month	Employed, full time
Post-graduates	>RM 10,000 per month	Employed, full time
Post-graduates	RM 5,001 - RM 10,000 per month	Employed, full time
Tertiary (including Polytechnics, MARA Colleges, University Colleges, and University)	>RM 10,000 per month	Employed, full time
Secondary	RM 5,001 - RM 10,000 per month	Employed, full time
Tertiary (including Polytechnics, MARA Colleges, University Colleges, and University)	RM 5,001 - RM 10,000 per month	Employed, full time
Secondary	RM 1,001 to 5,000 per month	Work from home/housewife
Tertiary (including Polytechnics, MARA Colleges, University Colleges, and University)	RM 5,001 - RM 10,000 per month	Employed, full time
Tertiary (including Polytechnics, MARA Colleges, University Colleges, and University)	>RM 10,000 per month	Employed, full time
Secondary	>RM 10,000 per month	Work from home/housewife
Tertiary (including Polytechnics, MARA Colleges, University Colleges, and University)	RM 5,001 - RM 10,000 per month	Work from home/housewife
Tertiary (including Polytechnics, MARA Colleges, University Colleges, and University)	RM 1,001 to 5,000 per month	Employed, full time
Tertiary (including Polytechnics, MARA Colleges, University Colleges, and University)	RM 1,001 to 5,000 per month	Work from home/housewife
Tertiary (including Polytechnics, MARA Colleges, University Colleges, and University)	>RM 10,000 per month	Employed, full time
Tertiary (including Polytechnics, MARA Colleges, University Colleges, and University)	RM 1,001 to 5,000 per month	Employed, full time
Secondary	RM 1,001 to 5,000 per month	Work from home/housewife
Tertiary (including Polytechnics, MARA Colleges, University Colleges, and University)	RM 5,001 - RM 10,000 per month	Employed, full time
Tertiary (including Polytechnics, MARA Colleges, University Colleges, and University)	>RM 10,000 per month	Employed, full time
Secondary	RM 1,001 to 5,000 per month	Employed, full time
Post-graduates	RM 5,001 - RM 10,000 per month	Employed, full time
Secondary	RM 1,001 to 5,000 per month	Employed, full time
Tertiary (including Polytechnics, MARA Colleges, University Colleges, and University)	RM 1,001 to 5,000 per month	Employed, full time

Tertiary (including Polytechnics, MARA Colleges, University Colleges, and University)	>RM 10,000 per month	Employed, full time
Tertiary (including Polytechnics, MARA Colleges, University Colleges, and University)	RM 1,001 to 5,000 per month	Employed, full time
Secondary	RM 1,001 to 5,000 per month	Work from home/housewife
Tertiary (including Polytechnics, MARA Colleges, University Colleges, and University)	RM 5,001 - RM 10,000 per month	Employed, full time
Tertiary (including Polytechnics, MARA Colleges, University Colleges, and University)	RM 5,001 - RM 10,000 per month	Employed, full time
Secondary	RM 1,001 to 5,000 per month	Employed, full time
Tertiary (including Polytechnics, MARA Colleges, University Colleges, and University)	RM 5,001 - RM 10,000 per month	Employed, full time
Secondary	RM 1,001 to 5,000 per month	Work from home/housewife
Tertiary (including Polytechnics, MARA Colleges, University Colleges, and University)	RM 5,001 - RM 10,000 per month	Employed, full time
Tertiary (including Polytechnics, MARA Colleges, University Colleges, and University)	RM 5,001 - RM 10,000 per month	Employed, full time
Tertiary (including Polytechnics, MARA Colleges, University Colleges, and University)	RM 5,001 - RM 10,000 per month	Employed, full time
Tertiary (including Polytechnics, MARA Colleges, University Colleges, and University)	>RM 10,000 per month	Employed, full time
Post-graduates	RM 5,001 - RM 10,000 per month	Employed, full time
Tertiary (including Polytechnics, MARA Colleges, University Colleges, and University)	RM 5,001 - RM 10,000 per month	Employed, full time
Secondary	RM 5,001 - RM 10,000 per month	Work from home/housewife
Tertiary (including Polytechnics, MARA Colleges, University Colleges, and University)	>RM 10,000 per month	Employed, full time
Tertiary (including Polytechnics, MARA Colleges, University Colleges, and University)	RM 5,001 - RM 10,000 per month	Employed, full time
Tertiary (including Polytechnics, MARA Colleges, University Colleges, and University)	RM 5,001 - RM 10,000 per month	Employed, full time
Secondary	RM 1,001 to 5,000 per month	Employed, full time
Tertiary (including Polytechnics, MARA Colleges, University Colleges, and University)	>RM 10,000 per month	Employed, full time
Tertiary (including Polytechnics, MARA Colleges, University Colleges, and University)	RM 5,001 - RM 10,000 per month	Employed, full time
Tertiary (including Polytechnics, MARA Colleges, University Colleges, and University)	RM 5,001 - RM 10,000 per month	Employed, full time
Tertiary (including Polytechnics, MARA Colleges, University Colleges, and University)	RM 5,001 - RM 10,000 per month	Employed, full time
Tertiary (including Polytechnics, MARA Colleges, University Colleges, and University)	RM 5,001 - RM 10,000 per month	Temporarily unemployed/medical leave/maternity
Secondary	RM 5,001 - RM 10,000 per month	Employed, full time
Secondary	RM 5,001 - RM 10,000 per month	Employed, full time
Tertiary (including Polytechnics, MARA Colleges, University Colleges, and University)	RM 5,001 - RM 10,000 per month	Employed, full time
Tertiary (including Polytechnics, MARA Colleges, University Colleges, and University)	>RM 10,000 per month	Employed, full time
Secondary	RM 1,001 to 5,000 per month	Employed, full time
Tertiary (including Polytechnics, MARA Colleges, University Colleges, and University)	RM 1,001 to 5,000 per month	Employed, full time
Tertiary (including Polytechnics, MARA Colleges, University Colleges, and University)	RM 5,001 - RM 10,000 per month	Employed, full time
Tertiary (including Polytechnics, MARA Colleges, University Colleges, and University)	>RM 10,000 per month	Employed, full time
Tertiary (including Polytechnics, MARA Colleges, University Colleges, and University)	RM 5,001 - RM 10,000 per month	Employed, full time

Tertiary (including Polytechnics, MARA Colleges, University Colleges, and University)	RM 5,001 - RM 10,000 per month	Employed, full time
Tertiary (including Polytechnics, MARA Colleges, University Colleges, and University)	RM 5,001 - RM 10,000 per month	Work from home/housewife
Tertiary (including Polytechnics, MARA Colleges, University Colleges, and University)	>RM 10,000 per month	Employed, full time
Secondary	RM 1,001 to 5,000 per month	Employed, full time
Tertiary (including Polytechnics, MARA Colleges, University Colleges, and University)	>RM 10,000 per month	Work from home/housewife
Tertiary (including Polytechnics, MARA Colleges, University Colleges, and University)	RM 5,001 - RM 10,000 per month	Employed, full time
Tertiary (including Polytechnics, MARA Colleges, University Colleges, and University)	RM 5,001 - RM 10,000 per month	Employed, full time
Post-graduates	RM 5,001 - RM 10,000 per month	Employed, full time
Tertiary (including Polytechnics, MARA Colleges, University Colleges, and University)	>RM 10,000 per month	Employed, full time
Post-graduates	>RM 10,000 per month	Employed, full time
Tertiary (including Polytechnics, MARA Colleges, University Colleges, and University)	RM 1,001 to 5,000 per month	Employed, full time
Secondary	RM 1,001 to 5,000 per month	Employed, full time
Tertiary (including Polytechnics, MARA Colleges, University Colleges, and University)	>RM 10,000 per month	Employed, full time
Tertiary (including Polytechnics, MARA Colleges, University Colleges, and University)	RM 1,001 to 5,000 per month	Employed, full time
Secondary	RM 5,001 - RM 10,000 per month	Work from home/housewife
Tertiary (including Polytechnics, MARA Colleges, University Colleges, and University)	RM 5,001 - RM 10,000 per month	Employed, full time
Tertiary (including Polytechnics, MARA Colleges, University Colleges, and University)	>RM 10,000 per month	Employed, full time
Tertiary (including Polytechnics, MARA Colleges, University Colleges, and University)	RM 1,001 to 5,000 per month	Employed, full time
Tertiary (including Polytechnics, MARA Colleges, University Colleges, and University)	>RM 10,000 per month	Work from home/housewife
Secondary	RM 1,001 to 5,000 per month	Work from home/housewife
Secondary	RM 1,001 to 5,000 per month	Work from home/housewife
Tertiary (including Polytechnics, MARA Colleges, University Colleges, and University)	RM 1,001 to 5,000 per month	Work from home/housewife
Tertiary (including Polytechnics, MARA Colleges, University Colleges, and University)	RM 5,001 - RM 10,000 per month	Employed, full time
Tertiary (including Polytechnics, MARA Colleges, University Colleges, and University)	>RM 10,000 per month	Employed, full time
Post-graduates	RM 5,001 - RM 10,000 per month	Employed, full time
Tertiary (including Polytechnics, MARA Colleges, University Colleges, and University)	RM 1,001 to 5,000 per month	Employed, full time
Tertiary (including Polytechnics, MARA Colleges, University Colleges, and University)	>RM 10,000 per month	Employed, full time
Primary	RM 1,001 to 5,000 per month	Employed, full time
Post-graduates	RM 1,001 to 5,000 per month	Work from home/housewife
Tertiary (including Polytechnics, MARA Colleges, University Colleges, and University)	RM 5,001 - RM 10,000 per month	Employed, full time
Tertiary (including Polytechnics, MARA Colleges, University Colleges, and University)	RM 1,001 to 5,000 per month	Employed, full time
Tertiary (including Polytechnics, MARA Colleges, University Colleges, and University)	>RM 10,000 per month	Employed, full time
Tertiary (including Polytechnics, MARA Colleges, University Colleges, and University)	>RM 10,000 per month	Temporarily unemployed/medical leave/maternity

Tertiary (including Polytechnics, MARA Colleges, University Colleges, and University)	RM 5,001 - RM 10,000 per month	Employed, full time
Tertiary (including Polytechnics, MARA Colleges, University Colleges, and University)	RM 5,001 - RM 10,000 per month	Employed, full time
Tertiary (including Polytechnics, MARA Colleges, University Colleges, and University)	RM 1,001 to 5,000 per month	Work from home/housewife
Tertiary (including Polytechnics, MARA Colleges, University Colleges, and University)	RM 5,001 - RM 10,000 per month	Employed, full time
Tertiary (including Polytechnics, MARA Colleges, University Colleges, and University)	RM 1,001 to 5,000 per month	Work from home/housewife
Secondary	RM 5,001 - RM 10,000 per month	Employed, full time
Tertiary (including Polytechnics, MARA Colleges, University Colleges, and University)	RM 5,001 - RM 10,000 per month	Employed, full time
Tertiary (including Polytechnics, MARA Colleges, University Colleges, and University)	>RM 10,000 per month	Employed, full time
Tertiary (including Polytechnics, MARA Colleges, University Colleges, and University)	RM 5,001 - RM 10,000 per month	Employed, full time
Tertiary (including Polytechnics, MARA Colleges, University Colleges, and University)	RM 5,001 - RM 10,000 per month	Employed, full time
Tertiary (including Polytechnics, MARA Colleges, University Colleges, and University)	RM 5,001 - RM 10,000 per month	Employed, full time
Tertiary (including Polytechnics, MARA Colleges, University Colleges, and University)	RM 5,001 - RM 10,000 per month	Employed, full time
Tertiary (including Polytechnics, MARA Colleges, University Colleges, and University)	RM 5,001 - RM 10,000 per month	Employed, full time
Tertiary (including Polytechnics, MARA Colleges, University Colleges, and University)	RM 1,001 to 5,000 per month	Work from home/housewife
Tertiary (including Polytechnics, MARA Colleges, University Colleges, and University)	RM 1,001 to 5,000 per month	Employed, full time
Post-graduates	RM 5,001 - RM 10,000 per month	Work from home/housewife
Tertiary (including Polytechnics, MARA Colleges, University Colleges, and University)	RM 5,001 - RM 10,000 per month	Employed, full time
Tertiary (including Polytechnics, MARA Colleges, University Colleges, and University)	RM 1,001 to 5,000 per month	Employed, full time
Tertiary (including Polytechnics, MARA Colleges, University Colleges, and University)	RM 5,001 - RM 10,000 per month	Employed, full time
Tertiary (including Polytechnics, MARA Colleges, University Colleges, and University)	RM 1,001 to 5,000 per month	Work from home/housewife
Tertiary (including Polytechnics, MARA Colleges, University Colleges, and University)	RM 5,001 - RM 10,000 per month	Employed, full time
Tertiary (including Polytechnics, MARA Colleges, University Colleges, and University)	RM 5,001 - RM 10,000 per month	Employed, full time
Secondary	RM 5,001 - RM 10,000 per month	Employed, full time
Tertiary (including Polytechnics, MARA Colleges, University Colleges, and University)	>RM 10,000 per month	Employed, full time
Tertiary (including Polytechnics, MARA Colleges, University Colleges, and University)	RM 5,001 - RM 10,000 per month	Employed, full time
Tertiary (including Polytechnics, MARA Colleges, University Colleges, and University)	RM 5,001 - RM 10,000 per month	Work from home/housewife
Tertiary (including Polytechnics, MARA Colleges, University Colleges, and University)	>RM 10,000 per month	Employed, full time
Secondary	RM 5,001 - RM 10,000 per month	Employed, full time
Tertiary (including Polytechnics, MARA Colleges, University Colleges, and University)	>RM 10,000 per month	Employed, full time
Tertiary (including Polytechnics, MARA Colleges, University Colleges, and University)	RM 1,001 to 5,000 per month	Work from home/housewife
Tertiary (including Polytechnics, MARA Colleges, University Colleges, and University)	RM 5,001 - RM 10,000 per month	Employed, full time
Tertiary (including Polytechnics, MARA Colleges, University Colleges, and University)	>RM 10,000 per month	Employed, full time
Tertiary (including Polytechnics, MARA Colleges, University Colleges, and University)	RM 1,001 to 5,000 per month	Employed, full time

Tertiary (including Polytechnics, MARA Colleges, University Colleges, and University)	>RM 10,000 per month	Employed, full time
Tertiary (including Polytechnics, MARA Colleges, University Colleges, and University)	>RM 10,000 per month	Employed, full time
Secondary	RM 1,001 to 5,000 per month	Employed, full time
Tertiary (including Polytechnics, MARA Colleges, University Colleges, and University)	RM 5,001 - RM 10,000 per month	Employed, full time
Tertiary (including Polytechnics, MARA Colleges, University Colleges, and University)	RM 5,001 - RM 10,000 per month	Employed, full time
Tertiary (including Polytechnics, MARA Colleges, University Colleges, and University)	RM 5,001 - RM 10,000 per month	Employed, full time
Tertiary (including Polytechnics, MARA Colleges, University Colleges, and University)	RM 1,001 to 5,000 per month	Employed, full time
Tertiary (including Polytechnics, MARA Colleges, University Colleges, and University)	RM 5,001 - RM 10,000 per month	Employed, full time
Tertiary (including Polytechnics, MARA Colleges, University Colleges, and University)	RM 5,001 - RM 10,000 per month	Employed, full time
Tertiary (including Polytechnics, MARA Colleges, University Colleges, and University)	RM 1,001 to 5,000 per month	Employed, full time
Secondary	RM 5,001 - RM 10,000 per month	Work from home/housewife
Tertiary (including Polytechnics, MARA Colleges, University Colleges, and University)	RM 5,001 - RM 10,000 per month	Employed, full time
Tertiary (including Polytechnics, MARA Colleges, University Colleges, and University)	RM 5,001 - RM 10,000 per month	Employed, full time
Tertiary (including Polytechnics, MARA Colleges, University Colleges, and University)	RM 1,001 to 5,000 per month	Employed, full time
Tertiary (including Polytechnics, MARA Colleges, University Colleges, and University)	RM 5,001 - RM 10,000 per month	Employed, full time
Tertiary (including Polytechnics, MARA Colleges, University Colleges, and University)	RM 5,001 - RM 10,000 per month	Employed, full time
Tertiary (including Polytechnics, MARA Colleges, University Colleges, and University)	RM 5,001 - RM 10,000 per month	Employed, full time
Tertiary (including Polytechnics, MARA Colleges, University Colleges, and University)	RM 1,001 to 5,000 per month	Employed, full time
Tertiary (including Polytechnics, MARA Colleges, University Colleges, and University)	>RM 10,000 per month	Employed, full time
Secondary	RM 1,001 to 5,000 per month	Employed, full time
Tertiary (including Polytechnics, MARA Colleges, University Colleges, and University)	RM 5,001 - RM 10,000 per month	Employed, full time
Tertiary (including Polytechnics, MARA Colleges, University Colleges, and University)	RM 5,001 - RM 10,000 per month	Employed, full time
Tertiary (including Polytechnics, MARA Colleges, University Colleges, and University)	RM 1,001 to 5,000 per month	Employed, full time
Secondary	RM 5,001 - RM 10,000 per month	Employed, full time
Tertiary (including Polytechnics, MARA Colleges, University Colleges, and University)	RM 1,001 to 5,000 per month	Employed, full time
Tertiary (including Polytechnics, MARA Colleges, University Colleges, and University)	RM 5,001 - RM 10,000 per month	Employed, full time
Tertiary (including Polytechnics, MARA Colleges, University Colleges, and University)	RM 1,001 to 5,000 per month	Employed, full time
Tertiary (including Polytechnics, MARA Colleges, University Colleges, and University)	RM 5,001 - RM 10,000 per month	Employed, full time
Tertiary (including Polytechnics, MARA Colleges, University Colleges, and University)	RM 5,001 - RM 10,000 per month	Employed, full time
Tertiary (including Polytechnics, MARA Colleges, University Colleges, and University)	>RM 10,000 per month	Employed, full time
Tertiary (including Polytechnics, MARA Colleges, University Colleges, and University)	RM 1,001 to 5,000 per month	Employed, full time
Tertiary (including Polytechnics, MARA Colleges, University Colleges, and University)	RM 5,001 - RM 10,000 per month	Employed, full time
Secondary	RM 1,001 to 5,000 per month	Work from home/housewife

Tertiary (including Polytechnics, MARA Colleges, University Colleges, and University)	RM 5,001 - RM 10,000 per month	Employed, full time
Post-graduates	>RM 10,000 per month	Employed, full time
Tertiary (including Polytechnics, MARA Colleges, University Colleges, and University)	RM 1,001 to 5,000 per month	Work from home/housewife
Tertiary (including Polytechnics, MARA Colleges, University Colleges, and University)	RM 1,001 to 5,000 per month	Employed, full time
Tertiary (including Polytechnics, MARA Colleges, University Colleges, and University)	RM 5,001 - RM 10,000 per month	Employed, full time
Tertiary (including Polytechnics, MARA Colleges, University Colleges, and University)	RM 5,001 - RM 10,000 per month	Employed, full time
Tertiary (including Polytechnics, MARA Colleges, University Colleges, and University)	RM 5,001 - RM 10,000 per month	Employed, full time
Tertiary (including Polytechnics, MARA Colleges, University Colleges, and University)	RM 1,001 to 5,000 per month	Employed, full time
Tertiary (including Polytechnics, MARA Colleges, University Colleges, and University)	>RM 10,000 per month	Employed, full time
Tertiary (including Polytechnics, MARA Colleges, University Colleges, and University)	RM 5,001 - RM 10,000 per month	Employed, full time
Tertiary (including Polytechnics, MARA Colleges, University Colleges, and University)	RM 5,001 - RM 10,000 per month	Employed, full time
Tertiary (including Polytechnics, MARA Colleges, University Colleges, and University)	RM 5,001 - RM 10,000 per month	Work from home/housewife
Tertiary (including Polytechnics, MARA Colleges, University Colleges, and University)	RM 5,001 - RM 10,000 per month	Work from home/housewife
Post-graduates	RM 5,001 - RM 10,000 per month	Employed, full time
Tertiary (including Polytechnics, MARA Colleges, University Colleges, and University)	>RM 10,000 per month	Employed, full time
Secondary	RM 5,001 - RM 10,000 per month	Employed, full time
Post-graduates	RM 5,001 - RM 10,000 per month	Employed, full time
Post-graduates	RM 5,001 - RM 10,000 per month	Employed, full time
Tertiary (including Polytechnics, MARA Colleges, University Colleges, and University)	RM 1,001 to 5,000 per month	Work from home/housewife
Tertiary (including Polytechnics, MARA Colleges, University Colleges, and University)	RM 5,001 - RM 10,000 per month	Employed, full time
Tertiary (including Polytechnics, MARA Colleges, University Colleges, and University)	RM 5,001 - RM 10,000 per month	Employed, full time
Secondary	>RM 10,000 per month	Work from home/housewife
Tertiary (including Polytechnics, MARA Colleges, University Colleges, and University)	RM 5,001 - RM 10,000 per month	Employed, full time
Tertiary (including Polytechnics, MARA Colleges, University Colleges, and University)	RM 1,001 to 5,000 per month	Temporarily unemployed/medical leave/maternity
Tertiary (including Polytechnics, MARA Colleges, University Colleges, and University)	RM 1,001 to 5,000 per month	Employed, full time
Tertiary (including Polytechnics, MARA Colleges, University Colleges, and University)	>RM 10,000 per month	Employed, full time
Tertiary (including Polytechnics, MARA Colleges, University Colleges, and University)	RM 5,001 - RM 10,000 per month	Employed, full time
Tertiary (including Polytechnics, MARA Colleges, University Colleges, and University)	>RM 10,000 per month	Employed, full time
Tertiary (including Polytechnics, MARA Colleges, University Colleges, and University)	RM 5,001 - RM 10,000 per month	Employed, full time
Tertiary (including Polytechnics, MARA Colleges, University Colleges, and University)	>RM 10,000 per month	Employed, full time
Tertiary (including Polytechnics, MARA Colleges, University Colleges, and University)	>RM 10,000 per month	Employed, full time
Tertiary (including Polytechnics, MARA Colleges, University Colleges, and University)	RM 5,001 - RM 10,000 per month	Employed, full time
Tertiary (including Polytechnics, MARA Colleges, University Colleges, and University)	RM 5,001 - RM 10,000 per month	Employed, full time

Tertiary (including Polytechnics, MARA Colleges, University Colleges, and University)	>RM 10,000 per month	Employed, full time
Secondary	RM 1,001 to 5,000 per month	Employed, full time
Secondary	RM 5,001 - RM 10,000 per month	Employed, full time
Tertiary (including Polytechnics, MARA Colleges, University Colleges, and University)	RM 5,001 - RM 10,000 per month	Employed, full time
Tertiary (including Polytechnics, MARA Colleges, University Colleges, and University)	RM 5,001 - RM 10,000 per month	Employed, full time
Post-graduates	>RM 10,000 per month	Employed, full time
Tertiary (including Polytechnics, MARA Colleges, University Colleges, and University)	RM 5,001 - RM 10,000 per month	Employed, full time
Tertiary (including Polytechnics, MARA Colleges, University Colleges, and University)	RM 5,001 - RM 10,000 per month	Employed, full time
Post-graduates	>RM 10,000 per month	Employed, full time
Tertiary (including Polytechnics, MARA Colleges, University Colleges, and University)	RM 5,001 - RM 10,000 per month	Employed, full time
Secondary	RM 1,001 to 5,000 per month	Work from home/housewife
Tertiary (including Polytechnics, MARA Colleges, University Colleges, and University)	RM 5,001 - RM 10,000 per month	Employed, full time
Tertiary (including Polytechnics, MARA Colleges, University Colleges, and University)	>RM 10,000 per month	Employed, full time
Tertiary (including Polytechnics, MARA Colleges, University Colleges, and University)	RM 5,001 - RM 10,000 per month	Employed, full time
Tertiary (including Polytechnics, MARA Colleges, University Colleges, and University)	>RM 10,000 per month	Employed, full time
Tertiary (including Polytechnics, MARA Colleges, University Colleges, and University)	>RM 10,000 per month	Employed, full time
Tertiary (including Polytechnics, MARA Colleges, University Colleges, and University)	RM 1,001 to 5,000 per month	Work from home/housewife
Tertiary (including Polytechnics, MARA Colleges, University Colleges, and University)	RM 1,001 to 5,000 per month	Employed, full time
Tertiary (including Polytechnics, MARA Colleges, University Colleges, and University)	>RM 10,000 per month	Employed, full time
Secondary	RM 1,001 to 5,000 per month	Employed, full time
Tertiary (including Polytechnics, MARA Colleges, University Colleges, and University)	RM 5,001 - RM 10,000 per month	Employed, full time
Tertiary (including Polytechnics, MARA Colleges, University Colleges, and University)	RM 5,001 - RM 10,000 per month	Employed, full time
Tertiary (including Polytechnics, MARA Colleges, University Colleges, and University)	RM 5,001 - RM 10,000 per month	Employed, full time
Secondary	RM 5,001 - RM 10,000 per month	Employed, full time
Secondary	RM 5,001 - RM 10,000 per month	Employed, full time
Tertiary (including Polytechnics, MARA Colleges, University Colleges, and University)	RM 1,001 to 5,000 per month	Employed, full time
Tertiary (including Polytechnics, MARA Colleges, University Colleges, and University)	RM 1,001 to 5,000 per month	Employed, full time
Tertiary (including Polytechnics, MARA Colleges, University Colleges, and University)	RM 1,001 to 5,000 per month	Work from home/housewife
Tertiary (including Polytechnics, MARA Colleges, University Colleges, and University)	RM 1,001 to 5,000 per month	Work from home/housewife
Tertiary (including Polytechnics, MARA Colleges, University Colleges, and University)	RM 5,001 - RM 10,000 per month	Employed, full time
Post-graduates	>RM 10,000 per month	Employed, full time
Secondary	<RM 1,000 per month	Work from home/housewife
Secondary	RM 1,001 to 5,000 per month	Employed, full time

Tertiary (including Polytechnics, MARA Colleges, University Colleges, and University)	RM 5,001 - RM 10,000 per month	Employed, full time
Secondary	RM 5,001 - RM 10,000 per month	Employed, full time
Tertiary (including Polytechnics, MARA Colleges, University Colleges, and University)	>RM 10,000 per month	Work from home/housewife
Tertiary (including Polytechnics, MARA Colleges, University Colleges, and University)	RM 1,001 to 5,000 per month	Employed, full time
Tertiary (including Polytechnics, MARA Colleges, University Colleges, and University)	RM 1,001 to 5,000 per month	Employed, full time
Tertiary (including Polytechnics, MARA Colleges, University Colleges, and University)	>RM 10,000 per month	Employed, full time
Tertiary (including Polytechnics, MARA Colleges, University Colleges, and University)	>RM 10,000 per month	Employed, full time
Post-graduates	RM 5,001 - RM 10,000 per month	Employed, full time
Tertiary (including Polytechnics, MARA Colleges, University Colleges, and University)	RM 5,001 - RM 10,000 per month	Employed, full time
Tertiary (including Polytechnics, MARA Colleges, University Colleges, and University)	RM 1,001 to 5,000 per month	Employed, full time
Secondary	RM 1,001 to 5,000 per month	Employed, full time
Tertiary (including Polytechnics, MARA Colleges, University Colleges, and University)	RM 5,001 - RM 10,000 per month	Employed, full time
Tertiary (including Polytechnics, MARA Colleges, University Colleges, and University)	RM 5,001 - RM 10,000 per month	Employed, full time
Tertiary (including Polytechnics, MARA Colleges, University Colleges, and University)	RM 5,001 - RM 10,000 per month	Employed, full time
Tertiary (including Polytechnics, MARA Colleges, University Colleges, and University)	RM 5,001 - RM 10,000 per month	Employed, full time
Tertiary (including Polytechnics, MARA Colleges, University Colleges, and University)	RM 5,001 - RM 10,000 per month	Employed, full time
Tertiary (including Polytechnics, MARA Colleges, University Colleges, and University)	RM 5,001 - RM 10,000 per month	Employed, full time
Tertiary (including Polytechnics, MARA Colleges, University Colleges, and University)	RM 1,001 to 5,000 per month	Work from home/housewife
Secondary	RM 5,001 - RM 10,000 per month	Employed, full time
Tertiary (including Polytechnics, MARA Colleges, University Colleges, and University)	RM 5,001 - RM 10,000 per month	Employed, full time
Tertiary (including Polytechnics, MARA Colleges, University Colleges, and University)	RM 5,001 - RM 10,000 per month	Work from home/housewife
Tertiary (including Polytechnics, MARA Colleges, University Colleges, and University)	>RM 10,000 per month	Employed, full time
Tertiary (including Polytechnics, MARA Colleges, University Colleges, and University)	RM 5,001 - RM 10,000 per month	Employed, full time
Tertiary (including Polytechnics, MARA Colleges, University Colleges, and University)	RM 5,001 - RM 10,000 per month	Employed, full time
Tertiary (including Polytechnics, MARA Colleges, University Colleges, and University)	RM 1,001 to 5,000 per month	Employed, full time
Post-graduates	>RM 10,000 per month	Employed, full time
Tertiary (including Polytechnics, MARA Colleges, University Colleges, and University)	RM 1,001 to 5,000 per month	Work from home/housewife
Post-graduates	RM 5,001 - RM 10,000 per month	Employed, full time
Tertiary (including Polytechnics, MARA Colleges, University Colleges, and University)	>RM 10,000 per month	Temporarily unemployed/medical leave/maternity
Tertiary (including Polytechnics, MARA Colleges, University Colleges, and University)	RM 5,001 - RM 10,000 per month	Employed, full time
Tertiary (including Polytechnics, MARA Colleges, University Colleges, and University)	>RM 10,000 per month	Employed, full time
Post-graduates	RM 5,001 - RM 10,000 per month	Employed, full time
Tertiary (including Polytechnics, MARA Colleges, University Colleges, and University)	>RM 10,000 per month	Employed, full time

Tertiary (including Polytechnics, MARA Colleges, University Colleges, and University)	RM 5,001 - RM 10,000 per month	Employed, full time
Tertiary (including Polytechnics, MARA Colleges, University Colleges, and University)	RM 5,001 - RM 10,000 per month	Employed, full time
Tertiary (including Polytechnics, MARA Colleges, University Colleges, and University)	RM 5,001 - RM 10,000 per month	Employed, full time
Tertiary (including Polytechnics, MARA Colleges, University Colleges, and University)	>RM 10,000 per month	Employed, full time
Tertiary (including Polytechnics, MARA Colleges, University Colleges, and University)	RM 5,001 - RM 10,000 per month	Work from home/housewife
Tertiary (including Polytechnics, MARA Colleges, University Colleges, and University)	RM 1,001 to 5,000 per month	Work from home/housewife
Secondary	RM 5,001 - RM 10,000 per month	Work from home/housewife
Tertiary (including Polytechnics, MARA Colleges, University Colleges, and University)	RM 1,001 to 5,000 per month	Employed, full time
Tertiary (including Polytechnics, MARA Colleges, University Colleges, and University)	RM 1,001 to 5,000 per month	Work from home/housewife
Post-graduates	RM 5,001 - RM 10,000 per month	Employed, full time
Secondary	RM 5,001 - RM 10,000 per month	Employed, full time
Tertiary (including Polytechnics, MARA Colleges, University Colleges, and University)	RM 5,001 - RM 10,000 per month	Employed, full time
Tertiary (including Polytechnics, MARA Colleges, University Colleges, and University)	RM 5,001 - RM 10,000 per month	Employed, full time
Tertiary (including Polytechnics, MARA Colleges, University Colleges, and University)	RM 5,001 - RM 10,000 per month	Work from home/housewife
Tertiary (including Polytechnics, MARA Colleges, University Colleges, and University)	RM 5,001 - RM 10,000 per month	Employed, full time
Tertiary (including Polytechnics, MARA Colleges, University Colleges, and University)	RM 5,001 - RM 10,000 per month	Employed, full time
Secondary	RM 1,001 to 5,000 per month	Work from home/housewife
Tertiary (including Polytechnics, MARA Colleges, University Colleges, and University)	RM 5,001 - RM 10,000 per month	Temporarily unemployed/medical leave/maternity
Secondary	RM 1,001 to 5,000 per month	Employed, part time
Tertiary (including Polytechnics, MARA Colleges, University Colleges, and University)	RM 5,001 - RM 10,000 per month	Employed, full time
Tertiary (including Polytechnics, MARA Colleges, University Colleges, and University)	RM 5,001 - RM 10,000 per month	Employed, full time
Secondary	RM 1,001 to 5,000 per month	Employed, full time
Secondary	RM 1,001 to 5,000 per month	Work from home/housewife
Tertiary (including Polytechnics, MARA Colleges, University Colleges, and University)	RM 5,001 - RM 10,000 per month	Employed, full time
Tertiary (including Polytechnics, MARA Colleges, University Colleges, and University)	RM 5,001 - RM 10,000 per month	Employed, full time
Tertiary (including Polytechnics, MARA Colleges, University Colleges, and University)	RM 5,001 - RM 10,000 per month	Employed, full time
Tertiary (including Polytechnics, MARA Colleges, University Colleges, and University)	RM 5,001 - RM 10,000 per month	Employed, full time
Tertiary (including Polytechnics, MARA Colleges, University Colleges, and University)	>RM 10,000 per month	Employed, full time
Post-graduates	RM 5,001 - RM 10,000 per month	Employed, full time
Tertiary (including Polytechnics, MARA Colleges, University Colleges, and University)	RM 5,001 - RM 10,000 per month	Others
Tertiary (including Polytechnics, MARA Colleges, University Colleges, and University)	RM 5,001 - RM 10,000 per month	Employed, full time
Tertiary (including Polytechnics, MARA Colleges, University Colleges, and University)	RM 5,001 - RM 10,000 per month	Employed, full time
Secondary	RM 5,001 - RM 10,000 per month	Employed, full time

Tertiary (including Polytechnics, MARA Colleges, University Colleges, and University)	RM 1,001 to 5,000 per month	Employed, full time
Tertiary (including Polytechnics, MARA Colleges, University Colleges, and University)	>RM 10,000 per month	Employed, full time
Tertiary (including Polytechnics, MARA Colleges, University Colleges, and University)	RM 1,001 to 5,000 per month	Work from home/housewife
Tertiary (including Polytechnics, MARA Colleges, University Colleges, and University)	RM 5,001 - RM 10,000 per month	Employed, full time
Tertiary (including Polytechnics, MARA Colleges, University Colleges, and University)	RM 5,001 - RM 10,000 per month	Employed, full time
Tertiary (including Polytechnics, MARA Colleges, University Colleges, and University)	>RM 10,000 per month	Employed, full time
Post-graduates	>RM 10,000 per month	Employed, full time
Tertiary (including Polytechnics, MARA Colleges, University Colleges, and University)	>RM 10,000 per month	Employed, full time
Tertiary (including Polytechnics, MARA Colleges, University Colleges, and University)	RM 5,001 - RM 10,000 per month	Employed, full time
Secondary	RM 1,001 to 5,000 per month	Work from home/housewife
Post-graduates	RM 5,001 - RM 10,000 per month	Employed, full time
Tertiary (including Polytechnics, MARA Colleges, University Colleges, and University)	RM 5,001 - RM 10,000 per month	Employed, full time
Tertiary (including Polytechnics, MARA Colleges, University Colleges, and University)	RM 5,001 - RM 10,000 per month	Employed, full time
Tertiary (including Polytechnics, MARA Colleges, University Colleges, and University)	RM 1,001 to 5,000 per month	Temporarily unemployed/medical leave/maternity
Tertiary (including Polytechnics, MARA Colleges, University Colleges, and University)	RM 1,001 to 5,000 per month	Employed, full time
Secondary	RM 1,001 to 5,000 per month	Employed, full time
Tertiary (including Polytechnics, MARA Colleges, University Colleges, and University)	RM 5,001 - RM 10,000 per month	Employed, full time
Tertiary (including Polytechnics, MARA Colleges, University Colleges, and University)	RM 1,001 to 5,000 per month	Employed, full time
Tertiary (including Polytechnics, MARA Colleges, University Colleges, and University)	>RM 10,000 per month	Employed, full time
Tertiary (including Polytechnics, MARA Colleges, University Colleges, and University)	RM 5,001 - RM 10,000 per month	Employed, full time
Tertiary (including Polytechnics, MARA Colleges, University Colleges, and University)	>RM 10,000 per month	Employed, full time
Post-graduates	>RM 10,000 per month	Employed, full time
Tertiary (including Polytechnics, MARA Colleges, University Colleges, and University)	RM 1,001 to 5,000 per month	Work from home/housewife
Secondary	RM 5,001 - RM 10,000 per month	Work from home/housewife
Post-graduates	RM 5,001 - RM 10,000 per month	Employed, full time
Tertiary (including Polytechnics, MARA Colleges, University Colleges, and University)	>RM 10,000 per month	Employed, full time
Secondary	RM 5,001 - RM 10,000 per month	Work from home/housewife
Tertiary (including Polytechnics, MARA Colleges, University Colleges, and University)	RM 5,001 - RM 10,000 per month	Employed, full time
Tertiary (including Polytechnics, MARA Colleges, University Colleges, and University)	RM 1,001 to 5,000 per month	Employed, full time
Tertiary (including Polytechnics, MARA Colleges, University Colleges, and University)	>RM 10,000 per month	Employed, full time
Tertiary (including Polytechnics, MARA Colleges, University Colleges, and University)	RM 5,001 - RM 10,000 per month	Employed, full time
Post-graduates	>RM 10,000 per month	Employed, full time
Tertiary (including Polytechnics, MARA Colleges, University Colleges, and University)	RM 1,001 to 5,000 per month	Employed, full time

Post-graduates	RM 1,001 to 5,000 per month	Employed, full time
Secondary	RM 1,001 to 5,000 per month	Work from home/housewife
Tertiary (including Polytechnics, MARA Colleges, University Colleges, and University)	>RM 10,000 per month	Employed, full time
Tertiary (including Polytechnics, MARA Colleges, University Colleges, and University)	RM 5,001 - RM 10,000 per month	Employed, full time
Post-graduates	>RM 10,000 per month	Employed, full time
Tertiary (including Polytechnics, MARA Colleges, University Colleges, and University)	>RM 10,000 per month	Employed, full time
Tertiary (including Polytechnics, MARA Colleges, University Colleges, and University)	RM 5,001 - RM 10,000 per month	Employed, full time
Tertiary (including Polytechnics, MARA Colleges, University Colleges, and University)	RM 5,001 - RM 10,000 per month	Employed, full time
Secondary	RM 1,001 to 5,000 per month	Employed, full time
Tertiary (including Polytechnics, MARA Colleges, University Colleges, and University)	RM 1,001 to 5,000 per month	Work from home/housewife
Post-graduates	>RM 10,000 per month	Employed, full time
Post-graduates	>RM 10,000 per month	Employed, full time
Tertiary (including Polytechnics, MARA Colleges, University Colleges, and University)	>RM 10,000 per month	Employed, full time
Secondary	RM 5,001 - RM 10,000 per month	Employed, full time
Tertiary (including Polytechnics, MARA Colleges, University Colleges, and University)	>RM 10,000 per month	Employed, full time
Tertiary (including Polytechnics, MARA Colleges, University Colleges, and University)	RM 5,001 - RM 10,000 per month	Employed, full time
Tertiary (including Polytechnics, MARA Colleges, University Colleges, and University)	RM 5,001 - RM 10,000 per month	Work from home/housewife
Tertiary (including Polytechnics, MARA Colleges, University Colleges, and University)	RM 5,001 - RM 10,000 per month	Employed, full time
Tertiary (including Polytechnics, MARA Colleges, University Colleges, and University)	RM 1,001 to 5,000 per month	Work from home/housewife
Tertiary (including Polytechnics, MARA Colleges, University Colleges, and University)	RM 1,001 to 5,000 per month	Employed, full time
Secondary	RM 5,001 - RM 10,000 per month	Employed, full time
Tertiary (including Polytechnics, MARA Colleges, University Colleges, and University)	RM 5,001 - RM 10,000 per month	Employed, full time
Tertiary (including Polytechnics, MARA Colleges, University Colleges, and University)	>RM 10,000 per month	Employed, full time
Tertiary (including Polytechnics, MARA Colleges, University Colleges, and University)	RM 5,001 - RM 10,000 per month	Employed, full time
Tertiary (including Polytechnics, MARA Colleges, University Colleges, and University)	>RM 10,000 per month	Work from home/housewife
Secondary	>RM 10,000 per month	Employed, full time
Tertiary (including Polytechnics, MARA Colleges, University Colleges, and University)	RM 1,001 to 5,000 per month	Temporarily unemployed/medical leave/maternity
Secondary	RM 5,001 - RM 10,000 per month	Employed, full time
Tertiary (including Polytechnics, MARA Colleges, University Colleges, and University)	RM 1,001 to 5,000 per month	Employed, full time
Tertiary (including Polytechnics, MARA Colleges, University Colleges, and University)	>RM 10,000 per month	Employed, full time
Tertiary (including Polytechnics, MARA Colleges, University Colleges, and University)	RM 1,001 to 5,000 per month	Work from home/housewife
Post-graduates	RM 5,001 - RM 10,000 per month	Employed, full time
Tertiary (including Polytechnics, MARA Colleges, University Colleges, and University)	>RM 10,000 per month	Employed, full time

Tertiary (including Polytechnics, MARA Colleges, University Colleges, and University)	RM 5,001 - RM 10,000 per month	Employed, full time
Post-graduates	RM 5,001 - RM 10,000 per month	Employed, full time
Tertiary (including Polytechnics, MARA Colleges, University Colleges, and University)	>RM 10,000 per month	Employed, full time
Tertiary (including Polytechnics, MARA Colleges, University Colleges, and University)	RM 1,001 to 5,000 per month	Employed, full time
Tertiary (including Polytechnics, MARA Colleges, University Colleges, and University)	RM 5,001 - RM 10,000 per month	Employed, full time
Secondary	RM 1,001 to 5,000 per month	Employed, full time
Tertiary (including Polytechnics, MARA Colleges, University Colleges, and University)	RM 5,001 - RM 10,000 per month	Employed, full time
Secondary	RM 1,001 to 5,000 per month	Work from home/housewife
Tertiary (including Polytechnics, MARA Colleges, University Colleges, and University)	RM 5,001 - RM 10,000 per month	Employed, full time
Tertiary (including Polytechnics, MARA Colleges, University Colleges, and University)	RM 5,001 - RM 10,000 per month	Employed, full time
Tertiary (including Polytechnics, MARA Colleges, University Colleges, and University)	RM 5,001 - RM 10,000 per month	Employed, full time
Tertiary (including Polytechnics, MARA Colleges, University Colleges, and University)	>RM 10,000 per month	Employed, full time
Tertiary (including Polytechnics, MARA Colleges, University Colleges, and University)	RM 5,001 - RM 10,000 per month	Employed, full time
Tertiary (including Polytechnics, MARA Colleges, University Colleges, and University)	RM 5,001 - RM 10,000 per month	Employed, full time
Tertiary (including Polytechnics, MARA Colleges, University Colleges, and University)	RM 1,001 to 5,000 per month	Employed, full time
Secondary	>RM 10,000 per month	Employed, full time
Tertiary (including Polytechnics, MARA Colleges, University Colleges, and University)	RM 5,001 - RM 10,000 per month	Employed, full time
Tertiary (including Polytechnics, MARA Colleges, University Colleges, and University)	RM 5,001 - RM 10,000 per month	Employed, full time
Tertiary (including Polytechnics, MARA Colleges, University Colleges, and University)	RM 5,001 - RM 10,000 per month	Employed, full time
Tertiary (including Polytechnics, MARA Colleges, University Colleges, and University)	RM 5,001 - RM 10,000 per month	Employed, full time
Tertiary (including Polytechnics, MARA Colleges, University Colleges, and University)	RM 1,001 to 5,000 per month	Work from home/housewife
Tertiary (including Polytechnics, MARA Colleges, University Colleges, and University)	RM 1,001 to 5,000 per month	Employed, full time
Secondary	RM 5,001 - RM 10,000 per month	Employed, full time
Tertiary (including Polytechnics, MARA Colleges, University Colleges, and University)	RM 5,001 - RM 10,000 per month	Employed, full time
Secondary	RM 1,001 to 5,000 per month	Employed, full time
Post-graduates	RM 5,001 - RM 10,000 per month	Employed, full time
Tertiary (including Polytechnics, MARA Colleges, University Colleges, and University)	RM 1,001 to 5,000 per month	Employed, full time
Tertiary (including Polytechnics, MARA Colleges, University Colleges, and University)	>RM 10,000 per month	Employed, full time
Post-graduates	>RM 10,000 per month	Employed, full time
Tertiary (including Polytechnics, MARA Colleges, University Colleges, and University)	RM 5,001 - RM 10,000 per month	Employed, full time
Secondary	RM 1,001 to 5,000 per month	Work from home/housewife
Tertiary (including Polytechnics, MARA Colleges, University Colleges, and University)	RM 1,001 to 5,000 per month	Work from home/housewife
Post-graduates	RM 5,001 - RM 10,000 per month	Employed, full time

Tertiary (including Polytechnics, MARA Colleges, University Colleges, and University)	RM 5,001 - RM 10,000 per month	Employed, full time
Tertiary (including Polytechnics, MARA Colleges, University Colleges, and University)	RM 1,001 to 5,000 per month	Work from home/housewife
Tertiary (including Polytechnics, MARA Colleges, University Colleges, and University)	RM 5,001 - RM 10,000 per month	Employed, full time
Tertiary (including Polytechnics, MARA Colleges, University Colleges, and University)	>RM 10,000 per month	Employed, full time
Tertiary (including Polytechnics, MARA Colleges, University Colleges, and University)	>RM 10,000 per month	Employed, full time
Tertiary (including Polytechnics, MARA Colleges, University Colleges, and University)	>RM 10,000 per month	Work from home/housewife
Tertiary (including Polytechnics, MARA Colleges, University Colleges, and University)	RM 5,001 - RM 10,000 per month	Employed, full time
Tertiary (including Polytechnics, MARA Colleges, University Colleges, and University)	RM 5,001 - RM 10,000 per month	Work from home/housewife
Secondary	RM 5,001 - RM 10,000 per month	Employed, full time
Tertiary (including Polytechnics, MARA Colleges, University Colleges, and University)	RM 5,001 - RM 10,000 per month	Employed, full time
Secondary	RM 5,001 - RM 10,000 per month	Employed, full time
Tertiary (including Polytechnics, MARA Colleges, University Colleges, and University)	RM 5,001 - RM 10,000 per month	Employed, full time
Post-graduates	>RM 10,000 per month	Employed, full time
Post-graduates	RM 5,001 - RM 10,000 per month	Employed, full time
Tertiary (including Polytechnics, MARA Colleges, University Colleges, and University)	RM 1,001 to 5,000 per month	Work from home/housewife
Primary	RM 1,001 to 5,000 per month	Work from home/housewife
Tertiary (including Polytechnics, MARA Colleges, University Colleges, and University)	RM 5,001 - RM 10,000 per month	Employed, full time
Secondary	RM 5,001 - RM 10,000 per month	Work from home/housewife
Tertiary (including Polytechnics, MARA Colleges, University Colleges, and University)	RM 1,001 to 5,000 per month	Employed, full time
Post-graduates	>RM 10,000 per month	Employed, full time
Tertiary (including Polytechnics, MARA Colleges, University Colleges, and University)	>RM 10,000 per month	Employed, full time
Tertiary (including Polytechnics, MARA Colleges, University Colleges, and University)	RM 5,001 - RM 10,000 per month	Employed, full time
Tertiary (including Polytechnics, MARA Colleges, University Colleges, and University)	RM 5,001 - RM 10,000 per month	Employed, full time
Tertiary (including Polytechnics, MARA Colleges, University Colleges, and University)	>RM 10,000 per month	Employed, full time
Tertiary (including Polytechnics, MARA Colleges, University Colleges, and University)	RM 1,001 to 5,000 per month	Employed, full time
Secondary	RM 1,001 to 5,000 per month	Work from home/housewife
Tertiary (including Polytechnics, MARA Colleges, University Colleges, and University)	RM 5,001 - RM 10,000 per month	Employed, full time
Tertiary (including Polytechnics, MARA Colleges, University Colleges, and University)	RM 5,001 - RM 10,000 per month	Employed, full time
Tertiary (including Polytechnics, MARA Colleges, University Colleges, and University)	RM 5,001 - RM 10,000 per month	Employed, full time
Post-graduates	>RM 10,000 per month	Employed, full time
Tertiary (including Polytechnics, MARA Colleges, University Colleges, and University)	RM 1,001 to 5,000 per month	Employed, full time
Tertiary (including Polytechnics, MARA Colleges, University Colleges, and University)	RM 1,001 to 5,000 per month	Work from home/housewife
Tertiary (including Polytechnics, MARA Colleges, University Colleges, and University)	RM 5,001 - RM 10,000 per month	Employed, full time

Tertiary (including Polytechnics, MARA Colleges, University Colleges, and University)	RM 5,001 - RM 10,000 per month	Employed, full time
Post-graduates	<RM 1,000 per month	Work from home/housewife
Tertiary (including Polytechnics, MARA Colleges, University Colleges, and University)	RM 1,001 to 5,000 per month	Employed, full time
Tertiary (including Polytechnics, MARA Colleges, University Colleges, and University)	RM 1,001 to 5,000 per month	Work from home/housewife
Secondary	RM 5,001 - RM 10,000 per month	Employed, full time
Tertiary (including Polytechnics, MARA Colleges, University Colleges, and University)	RM 5,001 - RM 10,000 per month	Employed, full time
Post-graduates	RM 5,001 - RM 10,000 per month	Employed, full time
Secondary	>RM 10,000 per month	Employed, full time
Tertiary (including Polytechnics, MARA Colleges, University Colleges, and University)	RM 5,001 - RM 10,000 per month	Employed, full time
Tertiary (including Polytechnics, MARA Colleges, University Colleges, and University)	>RM 10,000 per month	Employed, full time
Secondary	RM 5,001 - RM 10,000 per month	Employed, full time
Tertiary (including Polytechnics, MARA Colleges, University Colleges, and University)	RM 1,001 to 5,000 per month	Work from home/housewife
Secondary	RM 1,001 to 5,000 per month	Employed, full time
Tertiary (including Polytechnics, MARA Colleges, University Colleges, and University)	>RM 10,000 per month	Employed, full time
Tertiary (including Polytechnics, MARA Colleges, University Colleges, and University)	RM 5,001 - RM 10,000 per month	Employed, full time
Tertiary (including Polytechnics, MARA Colleges, University Colleges, and University)	>RM 10,000 per month	Employed, full time
Post-graduates	RM 5,001 - RM 10,000 per month	Employed, full time
Tertiary (including Polytechnics, MARA Colleges, University Colleges, and University)	>RM 10,000 per month	Work from home/housewife
Tertiary (including Polytechnics, MARA Colleges, University Colleges, and University)	RM 5,001 - RM 10,000 per month	Employed, full time
Tertiary (including Polytechnics, MARA Colleges, University Colleges, and University)	RM 5,001 - RM 10,000 per month	Work from home/housewife
Secondary	RM 1,001 to 5,000 per month	Work from home/housewife
Post-graduates	>RM 10,000 per month	Employed, full time
Tertiary (including Polytechnics, MARA Colleges, University Colleges, and University)	RM 5,001 - RM 10,000 per month	Employed, full time
Tertiary (including Polytechnics, MARA Colleges, University Colleges, and University)	RM 5,001 - RM 10,000 per month	Employed, full time
Secondary	RM 5,001 - RM 10,000 per month	Employed, full time
Tertiary (including Polytechnics, MARA Colleges, University Colleges, and University)	>RM 10,000 per month	Employed, full time
Tertiary (including Polytechnics, MARA Colleges, University Colleges, and University)	RM 5,001 - RM 10,000 per month	Employed, full time
Tertiary (including Polytechnics, MARA Colleges, University Colleges, and University)	>RM 10,000 per month	Employed, full time
Tertiary (including Polytechnics, MARA Colleges, University Colleges, and University)	RM 1,001 to 5,000 per month	Work from home/housewife
Tertiary (including Polytechnics, MARA Colleges, University Colleges, and University)	>RM 10,000 per month	Employed, full time
Secondary	RM 1,001 to 5,000 per month	Work from home/housewife
Post-graduates	>RM 10,000 per month	Employed, full time
Tertiary (including Polytechnics, MARA Colleges, University Colleges, and University)	RM 5,001 - RM 10,000 per month	Work from home/housewife

Tertiary (including Polytechnics, MARA Colleges, University Colleges, and University)	RM 5,001 - RM 10,000 per month	Employed, full time
Secondary	RM 1,001 to 5,000 per month	Employed, full time
Post-graduates	RM 5,001 - RM 10,000 per month	Employed, full time
Secondary	RM 5,001 - RM 10,000 per month	Employed, full time
Post-graduates	RM 5,001 - RM 10,000 per month	Work from home/housewife
Tertiary (including Polytechnics, MARA Colleges, University Colleges, and University)	RM 1,001 to 5,000 per month	Work from home/housewife
Tertiary (including Polytechnics, MARA Colleges, University Colleges, and University)	RM 1,001 to 5,000 per month	Employed, full time
Tertiary (including Polytechnics, MARA Colleges, University Colleges, and University)	RM 5,001 - RM 10,000 per month	Employed, full time
Tertiary (including Polytechnics, MARA Colleges, University Colleges, and University)	RM 1,001 to 5,000 per month	Temporarily unemployed/medical leave/maternity
Tertiary (including Polytechnics, MARA Colleges, University Colleges, and University)	RM 5,001 - RM 10,000 per month	Employed, part time
Tertiary (including Polytechnics, MARA Colleges, University Colleges, and University)	RM 5,001 - RM 10,000 per month	Employed, full time
Tertiary (including Polytechnics, MARA Colleges, University Colleges, and University)	RM 1,001 to 5,000 per month	Employed, full time
Tertiary (including Polytechnics, MARA Colleges, University Colleges, and University)	RM 5,001 - RM 10,000 per month	Employed, full time
Post-graduates	RM 5,001 - RM 10,000 per month	Employed, full time
Tertiary (including Polytechnics, MARA Colleges, University Colleges, and University)	RM 5,001 - RM 10,000 per month	Employed, full time
Tertiary (including Polytechnics, MARA Colleges, University Colleges, and University)	RM 5,001 - RM 10,000 per month	Employed, full time
Tertiary (including Polytechnics, MARA Colleges, University Colleges, and University)	>RM 10,000 per month	Employed, full time
Tertiary (including Polytechnics, MARA Colleges, University Colleges, and University)	>RM 10,000 per month	Employed, full time
Tertiary (including Polytechnics, MARA Colleges, University Colleges, and University)	>RM 10,000 per month	Employed, full time
Secondary	RM 5,001 - RM 10,000 per month	Employed, full time
Tertiary (including Polytechnics, MARA Colleges, University Colleges, and University)	RM 5,001 - RM 10,000 per month	Employed, full time
Tertiary (including Polytechnics, MARA Colleges, University Colleges, and University)	RM 5,001 - RM 10,000 per month	Employed, full time
Tertiary (including Polytechnics, MARA Colleges, University Colleges, and University)	RM 5,001 - RM 10,000 per month	Employed, full time
Tertiary (including Polytechnics, MARA Colleges, University Colleges, and University)	RM 5,001 - RM 10,000 per month	Employed, full time
Tertiary (including Polytechnics, MARA Colleges, University Colleges, and University)	RM 5,001 - RM 10,000 per month	Employed, full time
Tertiary (including Polytechnics, MARA Colleges, University Colleges, and University)	RM 1,001 to 5,000 per month	Employed, full time
Tertiary (including Polytechnics, MARA Colleges, University Colleges, and University)	RM 1,001 to 5,000 per month	Employed, full time
Tertiary (including Polytechnics, MARA Colleges, University Colleges, and University)	RM 1,001 to 5,000 per month	Work from home/housewife
Tertiary (including Polytechnics, MARA Colleges, University Colleges, and University)	RM 5,001 - RM 10,000 per month	Employed, full time
Tertiary (including Polytechnics, MARA Colleges, University Colleges, and University)	>RM 10,000 per month	Employed, full time
Tertiary (including Polytechnics, MARA Colleges, University Colleges, and University)	RM 5,001 - RM 10,000 per month	Employed, full time
Tertiary (including Polytechnics, MARA Colleges, University Colleges, and University)	RM 1,001 to 5,000 per month	Employed, full time
Tertiary (including Polytechnics, MARA Colleges, University Colleges, and University)	RM 5,001 - RM 10,000 per month	Employed, full time

Tertiary (including Polytechnics, MARA Colleges, University Colleges, and University)	RM 5,001 - RM 10,000 per month	Employed, full time
Tertiary (including Polytechnics, MARA Colleges, University Colleges, and University)	RM 1,001 to 5,000 per month	Employed, full time
Tertiary (including Polytechnics, MARA Colleges, University Colleges, and University)	RM 1,001 to 5,000 per month	Employed, full time
Tertiary (including Polytechnics, MARA Colleges, University Colleges, and University)	>RM 10,000 per month	Work from home/housewife
Tertiary (including Polytechnics, MARA Colleges, University Colleges, and University)	>RM 10,000 per month	Employed, full time
Tertiary (including Polytechnics, MARA Colleges, University Colleges, and University)	RM 5,001 - RM 10,000 per month	Employed, full time
Tertiary (including Polytechnics, MARA Colleges, University Colleges, and University)	>RM 10,000 per month	Employed, full time
Secondary	RM 1,001 to 5,000 per month	Employed, full time
Tertiary (including Polytechnics, MARA Colleges, University Colleges, and University)	RM 1,001 to 5,000 per month	Employed, full time
Primary	RM 5,001 - RM 10,000 per month	Employed, full time
Tertiary (including Polytechnics, MARA Colleges, University Colleges, and University)	RM 1,001 to 5,000 per month	Work from home/housewife
Secondary	RM 5,001 - RM 10,000 per month	Employed, full time
Post-graduates	RM 5,001 - RM 10,000 per month	Employed, full time
Secondary	RM 1,001 to 5,000 per month	Temporarily unemployed/medical leave/maternity
Tertiary (including Polytechnics, MARA Colleges, University Colleges, and University)	RM 5,001 - RM 10,000 per month	Employed, full time
Tertiary (including Polytechnics, MARA Colleges, University Colleges, and University)	RM 1,001 to 5,000 per month	Employed, full time
Tertiary (including Polytechnics, MARA Colleges, University Colleges, and University)	RM 5,001 - RM 10,000 per month	Work from home/housewife
Tertiary (including Polytechnics, MARA Colleges, University Colleges, and University)	>RM 10,000 per month	Employed, full time
Tertiary (including Polytechnics, MARA Colleges, University Colleges, and University)	RM 5,001 - RM 10,000 per month	Employed, full time
Tertiary (including Polytechnics, MARA Colleges, University Colleges, and University)	RM 5,001 - RM 10,000 per month	Employed, full time
Tertiary (including Polytechnics, MARA Colleges, University Colleges, and University)	RM 1,001 to 5,000 per month	Employed, full time
Tertiary (including Polytechnics, MARA Colleges, University Colleges, and University)	>RM 10,000 per month	Employed, full time
Tertiary (including Polytechnics, MARA Colleges, University Colleges, and University)	RM 5,001 - RM 10,000 per month	Employed, full time
Tertiary (including Polytechnics, MARA Colleges, University Colleges, and University)	RM 5,001 - RM 10,000 per month	Employed, full time
Tertiary (including Polytechnics, MARA Colleges, University Colleges, and University)	>RM 10,000 per month	Employed, full time
Tertiary (including Polytechnics, MARA Colleges, University Colleges, and University)	RM 1,001 to 5,000 per month	Work from home/housewife
Tertiary (including Polytechnics, MARA Colleges, University Colleges, and University)	RM 5,001 - RM 10,000 per month	Employed, full time
Secondary	RM 5,001 - RM 10,000 per month	Employed, full time
Tertiary (including Polytechnics, MARA Colleges, University Colleges, and University)	RM 5,001 - RM 10,000 per month	Employed, full time
Tertiary (including Polytechnics, MARA Colleges, University Colleges, and University)	RM 5,001 - RM 10,000 per month	Employed, full time
Tertiary (including Polytechnics, MARA Colleges, University Colleges, and University)	RM 5,001 - RM 10,000 per month	Employed, full time
Tertiary (including Polytechnics, MARA Colleges, University Colleges, and University)	RM 1,001 to 5,000 per month	Employed, full time
Tertiary (including Polytechnics, MARA Colleges, University Colleges, and University)	RM 5,001 - RM 10,000 per month	Employed, part time

Post-graduates	>RM 10,000 per month	Employed, full time
Tertiary (including Polytechnics, MARA Colleges, University Colleges, and University)	>RM 10,000 per month	Work from home/housewife
Tertiary (including Polytechnics, MARA Colleges, University Colleges, and University)	>RM 10,000 per month	Employed, full time
Post-graduates	>RM 10,000 per month	Employed, full time
Tertiary (including Polytechnics, MARA Colleges, University Colleges, and University)	RM 5,001 - RM 10,000 per month	Employed, full time
Tertiary (including Polytechnics, MARA Colleges, University Colleges, and University)	RM 5,001 - RM 10,000 per month	Employed, full time
Tertiary (including Polytechnics, MARA Colleges, University Colleges, and University)	RM 5,001 - RM 10,000 per month	Work from home/housewife
Tertiary (including Polytechnics, MARA Colleges, University Colleges, and University)	>RM 10,000 per month	Employed, full time
Post-graduates	>RM 10,000 per month	Employed, full time
Secondary	RM 1,001 to 5,000 per month	Employed, full time
Tertiary (including Polytechnics, MARA Colleges, University Colleges, and University)	>RM 10,000 per month	Employed, full time
Post-graduates	RM 5,001 - RM 10,000 per month	Employed, full time
Tertiary (including Polytechnics, MARA Colleges, University Colleges, and University)	RM 5,001 - RM 10,000 per month	Employed, full time
Tertiary (including Polytechnics, MARA Colleges, University Colleges, and University)	>RM 10,000 per month	Employed, full time
Tertiary (including Polytechnics, MARA Colleges, University Colleges, and University)	RM 5,001 - RM 10,000 per month	Employed, full time
Secondary	RM 5,001 - RM 10,000 per month	Employed, full time
Tertiary (including Polytechnics, MARA Colleges, University Colleges, and University)	RM 5,001 - RM 10,000 per month	Employed, full time
Tertiary (including Polytechnics, MARA Colleges, University Colleges, and University)	RM 5,001 - RM 10,000 per month	Employed, full time
Tertiary (including Polytechnics, MARA Colleges, University Colleges, and University)	RM 5,001 - RM 10,000 per month	Employed, full time
Secondary	RM 5,001 - RM 10,000 per month	Employed, full time
Secondary	RM 1,001 to 5,000 per month	Work from home/housewife
Tertiary (including Polytechnics, MARA Colleges, University Colleges, and University)	RM 5,001 - RM 10,000 per month	Employed, full time
Tertiary (including Polytechnics, MARA Colleges, University Colleges, and University)	RM 5,001 - RM 10,000 per month	Employed, full time
Tertiary (including Polytechnics, MARA Colleges, University Colleges, and University)	>RM 10,000 per month	Employed, full time
Tertiary (including Polytechnics, MARA Colleges, University Colleges, and University)	RM 5,001 - RM 10,000 per month	Employed, full time
Post-graduates	>RM 10,000 per month	Employed, full time
Secondary	<RM 1,000 per month	Work from home/housewife
Tertiary (including Polytechnics, MARA Colleges, University Colleges, and University)	RM 1,001 to 5,000 per month	Employed, full time
Tertiary (including Polytechnics, MARA Colleges, University Colleges, and University)	RM 5,001 - RM 10,000 per month	Employed, full time
Secondary	RM 5,001 - RM 10,000 per month	Employed, full time
Tertiary (including Polytechnics, MARA Colleges, University Colleges, and University)	RM 1,001 to 5,000 per month	Employed, full time
Tertiary (including Polytechnics, MARA Colleges, University Colleges, and University)	RM 5,001 - RM 10,000 per month	Employed, full time
Tertiary (including Polytechnics, MARA Colleges, University Colleges, and University)	RM 5,001 - RM 10,000 per month	Employed, full time

Tertiary (including Polytechnics, MARA Colleges, University Colleges, and University)	RM 5,001 - RM 10,000 per month	Employed, full time
Secondary	RM 5,001 - RM 10,000 per month	Employed, full time
Tertiary (including Polytechnics, MARA Colleges, University Colleges, and University)	RM 1,001 to 5,000 per month	Work from home/housewife
Tertiary (including Polytechnics, MARA Colleges, University Colleges, and University)	RM 1,001 to 5,000 per month	Employed, full time
Tertiary (including Polytechnics, MARA Colleges, University Colleges, and University)	RM 5,001 - RM 10,000 per month	Employed, full time
Post-graduates	>RM 10,000 per month	Employed, full time
Tertiary (including Polytechnics, MARA Colleges, University Colleges, and University)	RM 5,001 - RM 10,000 per month	Employed, full time
Tertiary (including Polytechnics, MARA Colleges, University Colleges, and University)	RM 1,001 to 5,000 per month	Work from home/housewife
Secondary	>RM 10,000 per month	Employed, full time
Tertiary (including Polytechnics, MARA Colleges, University Colleges, and University)	>RM 10,000 per month	Employed, full time
Secondary	RM 5,001 - RM 10,000 per month	Employed, full time
Post-graduates	RM 5,001 - RM 10,000 per month	Employed, full time
Tertiary (including Polytechnics, MARA Colleges, University Colleges, and University)	RM 1,001 to 5,000 per month	Employed, full time
Tertiary (including Polytechnics, MARA Colleges, University Colleges, and University)	RM 5,001 - RM 10,000 per month	Employed, full time
Tertiary (including Polytechnics, MARA Colleges, University Colleges, and University)	RM 5,001 - RM 10,000 per month	Employed, full time
Tertiary (including Polytechnics, MARA Colleges, University Colleges, and University)	RM 5,001 - RM 10,000 per month	Employed, full time
Tertiary (including Polytechnics, MARA Colleges, University Colleges, and University)	RM 5,001 - RM 10,000 per month	Employed, full time
Tertiary (including Polytechnics, MARA Colleges, University Colleges, and University)	RM 5,001 - RM 10,000 per month	Work from home/housewife
Secondary	RM 1,001 to 5,000 per month	Work from home/housewife
Post-graduates	>RM 10,000 per month	Employed, full time
Tertiary (including Polytechnics, MARA Colleges, University Colleges, and University)	RM 5,001 - RM 10,000 per month	Employed, full time
Tertiary (including Polytechnics, MARA Colleges, University Colleges, and University)	RM 5,001 - RM 10,000 per month	Work from home/housewife
Tertiary (including Polytechnics, MARA Colleges, University Colleges, and University)	RM 5,001 - RM 10,000 per month	Employed, full time
Tertiary (including Polytechnics, MARA Colleges, University Colleges, and University)	>RM 10,000 per month	Employed, full time
Tertiary (including Polytechnics, MARA Colleges, University Colleges, and University)	RM 5,001 - RM 10,000 per month	Employed, full time
Tertiary (including Polytechnics, MARA Colleges, University Colleges, and University)	RM 1,001 to 5,000 per month	Work from home/housewife
Tertiary (including Polytechnics, MARA Colleges, University Colleges, and University)	>RM 10,000 per month	Employed, full time
Secondary	RM 1,001 to 5,000 per month	Work from home/housewife
Tertiary (including Polytechnics, MARA Colleges, University Colleges, and University)	RM 5,001 - RM 10,000 per month	Employed, full time
Secondary	RM 1,001 to 5,000 per month	Employed, full time
Secondary	RM 1,001 to 5,000 per month	Employed, full time
Tertiary (including Polytechnics, MARA Colleges, University Colleges, and University)	RM 5,001 - RM 10,000 per month	Employed, full time
Tertiary (including Polytechnics, MARA Colleges, University Colleges, and University)	RM 1,001 to 5,000 per month	Employed, full time

Tertiary (including Polytechnics, MARA Colleges, University Colleges, and University)	RM 5,001 - RM 10,000 per month	Employed, full time
Tertiary (including Polytechnics, MARA Colleges, University Colleges, and University)	RM 1,001 to 5,000 per month	Employed, full time
Tertiary (including Polytechnics, MARA Colleges, University Colleges, and University)	RM 1,001 to 5,000 per month	Employed, full time
Tertiary (including Polytechnics, MARA Colleges, University Colleges, and University)	>RM 10,000 per month	Employed, full time
Tertiary (including Polytechnics, MARA Colleges, University Colleges, and University)	RM 5,001 - RM 10,000 per month	Employed, full time
Tertiary (including Polytechnics, MARA Colleges, University Colleges, and University)	RM 5,001 - RM 10,000 per month	Employed, full time
Tertiary (including Polytechnics, MARA Colleges, University Colleges, and University)	RM 5,001 - RM 10,000 per month	Employed, full time
Tertiary (including Polytechnics, MARA Colleges, University Colleges, and University)	RM 1,001 to 5,000 per month	Employed, full time
Tertiary (including Polytechnics, MARA Colleges, University Colleges, and University)	RM 5,001 - RM 10,000 per month	Employed, full time
Tertiary (including Polytechnics, MARA Colleges, University Colleges, and University)	RM 5,001 - RM 10,000 per month	Employed, full time
Tertiary (including Polytechnics, MARA Colleges, University Colleges, and University)	<RM 1,000 per month	Work from home/housewife
Tertiary (including Polytechnics, MARA Colleges, University Colleges, and University)	RM 5,001 - RM 10,000 per month	Employed, full time
Tertiary (including Polytechnics, MARA Colleges, University Colleges, and University)	RM 5,001 - RM 10,000 per month	Employed, full time
Tertiary (including Polytechnics, MARA Colleges, University Colleges, and University)	RM 5,001 - RM 10,000 per month	Employed, full time
Tertiary (including Polytechnics, MARA Colleges, University Colleges, and University)	RM 5,001 - RM 10,000 per month	Employed, full time
Tertiary (including Polytechnics, MARA Colleges, University Colleges, and University)	RM 1,001 to 5,000 per month	Employed, full time
Tertiary (including Polytechnics, MARA Colleges, University Colleges, and University)	RM 5,001 - RM 10,000 per month	Employed, full time
Tertiary (including Polytechnics, MARA Colleges, University Colleges, and University)	>RM 10,000 per month	Employed, full time
Post-graduates	RM 5,001 - RM 10,000 per month	Employed, full time
Tertiary (including Polytechnics, MARA Colleges, University Colleges, and University)	RM 5,001 - RM 10,000 per month	Employed, full time
Secondary	RM 5,001 - RM 10,000 per month	Employed, full time
Tertiary (including Polytechnics, MARA Colleges, University Colleges, and University)	RM 5,001 - RM 10,000 per month	Employed, full time
Tertiary (including Polytechnics, MARA Colleges, University Colleges, and University)	RM 5,001 - RM 10,000 per month	Employed, full time
Post-graduates	RM 5,001 - RM 10,000 per month	Employed, full time
Secondary	RM 5,001 - RM 10,000 per month	Employed, full time
Tertiary (including Polytechnics, MARA Colleges, University Colleges, and University)	RM 5,001 - RM 10,000 per month	Employed, full time
Tertiary (including Polytechnics, MARA Colleges, University Colleges, and University)	RM 1,001 to 5,000 per month	Employed, full time
Tertiary (including Polytechnics, MARA Colleges, University Colleges, and University)	RM 5,001 - RM 10,000 per month	Employed, full time
Secondary	RM 5,001 - RM 10,000 per month	Employed, full time
Tertiary (including Polytechnics, MARA Colleges, University Colleges, and University)	>RM 10,000 per month	Employed, full time
Tertiary (including Polytechnics, MARA Colleges, University Colleges, and University)	RM 1,001 to 5,000 per month	Employed, full time
Tertiary (including Polytechnics, MARA Colleges, University Colleges, and University)	RM 5,001 - RM 10,000 per month	Employed, full time
Tertiary (including Polytechnics, MARA Colleges, University Colleges, and University)	RM 5,001 - RM 10,000 per month	Employed, full time

Tertiary (including Polytechnics, MARA Colleges, University Colleges, and University)	>RM 10,000 per month	Employed, full time
Secondary	RM 1,001 to 5,000 per month	Employed, full time
Tertiary (including Polytechnics, MARA Colleges, University Colleges, and University)	RM 5,001 - RM 10,000 per month	Employed, full time
Secondary	RM 5,001 - RM 10,000 per month	Work from home/housewife
Tertiary (including Polytechnics, MARA Colleges, University Colleges, and University)	>RM 10,000 per month	Employed, full time
Tertiary (including Polytechnics, MARA Colleges, University Colleges, and University)	>RM 10,000 per month	Employed, full time
Post-graduates	RM 5,001 - RM 10,000 per month	Employed, full time
Tertiary (including Polytechnics, MARA Colleges, University Colleges, and University)	RM 1,001 to 5,000 per month	Employed, full time
Tertiary (including Polytechnics, MARA Colleges, University Colleges, and University)	RM 5,001 - RM 10,000 per month	Employed, part time
Tertiary (including Polytechnics, MARA Colleges, University Colleges, and University)	>RM 10,000 per month	Work from home/housewife
Tertiary (including Polytechnics, MARA Colleges, University Colleges, and University)	RM 5,001 - RM 10,000 per month	Employed, full time
Tertiary (including Polytechnics, MARA Colleges, University Colleges, and University)	RM 5,001 - RM 10,000 per month	Employed, full time
Tertiary (including Polytechnics, MARA Colleges, University Colleges, and University)	RM 1,001 to 5,000 per month	Employed, full time
Secondary	RM 1,001 to 5,000 per month	Work from home/housewife
Post-graduates	RM 1,001 to 5,000 per month	Employed, full time
Tertiary (including Polytechnics, MARA Colleges, University Colleges, and University)	RM 5,001 - RM 10,000 per month	Employed, full time
Tertiary (including Polytechnics, MARA Colleges, University Colleges, and University)	RM 5,001 - RM 10,000 per month	Employed, full time
Post-graduates	>RM 10,000 per month	Work from home/housewife
Secondary	RM 5,001 - RM 10,000 per month	Employed, part time
Tertiary (including Polytechnics, MARA Colleges, University Colleges, and University)	RM 5,001 - RM 10,000 per month	Employed, full time
Tertiary (including Polytechnics, MARA Colleges, University Colleges, and University)	RM 5,001 - RM 10,000 per month	Employed, full time
Tertiary (including Polytechnics, MARA Colleges, University Colleges, and University)	>RM 10,000 per month	Employed, full time
Tertiary (including Polytechnics, MARA Colleges, University Colleges, and University)	>RM 10,000 per month	Employed, full time
Tertiary (including Polytechnics, MARA Colleges, University Colleges, and University)	RM 5,001 - RM 10,000 per month	Employed, full time
Secondary	>RM 10,000 per month	Employed, full time
Tertiary (including Polytechnics, MARA Colleges, University Colleges, and University)	>RM 10,000 per month	Employed, full time
Tertiary (including Polytechnics, MARA Colleges, University Colleges, and University)	RM 1,001 to 5,000 per month	Work from home/housewife
Secondary	RM 5,001 - RM 10,000 per month	Employed, full time
Secondary	RM 1,001 to 5,000 per month	Employed, full time
Tertiary (including Polytechnics, MARA Colleges, University Colleges, and University)	>RM 10,000 per month	Employed, full time
Tertiary (including Polytechnics, MARA Colleges, University Colleges, and University)	RM 1,001 to 5,000 per month	Employed, full time
Tertiary (including Polytechnics, MARA Colleges, University Colleges, and University)	>RM 10,000 per month	Employed, full time
Tertiary (including Polytechnics, MARA Colleges, University Colleges, and University)	RM 5,001 - RM 10,000 per month	Employed, full time

Tertiary (including Polytechnics, MARA Colleges, University Colleges, and University)	>RM 10,000 per month	Employed, full time
Tertiary (including Polytechnics, MARA Colleges, University Colleges, and University)	RM 5,001 - RM 10,000 per month	Employed, full time
Secondary	RM 1,001 to 5,000 per month	Employed, full time
Tertiary (including Polytechnics, MARA Colleges, University Colleges, and University)	RM 5,001 - RM 10,000 per month	Employed, full time
Tertiary (including Polytechnics, MARA Colleges, University Colleges, and University)	RM 5,001 - RM 10,000 per month	Employed, full time
Tertiary (including Polytechnics, MARA Colleges, University Colleges, and University)	RM 1,001 to 5,000 per month	Employed, full time
Tertiary (including Polytechnics, MARA Colleges, University Colleges, and University)	RM 5,001 - RM 10,000 per month	Work from home/housewife
Tertiary (including Polytechnics, MARA Colleges, University Colleges, and University)	RM 5,001 - RM 10,000 per month	Employed, full time
Tertiary (including Polytechnics, MARA Colleges, University Colleges, and University)	RM 5,001 - RM 10,000 per month	Employed, full time
Tertiary (including Polytechnics, MARA Colleges, University Colleges, and University)	RM 5,001 - RM 10,000 per month	Employed, full time
Secondary	RM 5,001 - RM 10,000 per month	Employed, full time
Tertiary (including Polytechnics, MARA Colleges, University Colleges, and University)	RM 5,001 - RM 10,000 per month	Employed, full time
Tertiary (including Polytechnics, MARA Colleges, University Colleges, and University)	RM 5,001 - RM 10,000 per month	Work from home/housewife
Tertiary (including Polytechnics, MARA Colleges, University Colleges, and University)	RM 5,001 - RM 10,000 per month	Employed, full time
Tertiary (including Polytechnics, MARA Colleges, University Colleges, and University)	RM 1,001 to 5,000 per month	Employed, full time
Tertiary (including Polytechnics, MARA Colleges, University Colleges, and University)	RM 1,001 to 5,000 per month	Employed, full time
Tertiary (including Polytechnics, MARA Colleges, University Colleges, and University)	RM 5,001 - RM 10,000 per month	Employed, full time
Post-graduates	RM 5,001 - RM 10,000 per month	Employed, full time
Tertiary (including Polytechnics, MARA Colleges, University Colleges, and University)	RM 5,001 - RM 10,000 per month	Employed, part time
Tertiary (including Polytechnics, MARA Colleges, University Colleges, and University)	RM 1,001 to 5,000 per month	Employed, full time
Post-graduates	>RM 10,000 per month	Employed, full time
Tertiary (including Polytechnics, MARA Colleges, University Colleges, and University)	>RM 10,000 per month	Employed, full time
Tertiary (including Polytechnics, MARA Colleges, University Colleges, and University)	>RM 10,000 per month	Employed, full time
Tertiary (including Polytechnics, MARA Colleges, University Colleges, and University)	RM 1,001 to 5,000 per month	Employed, full time
Tertiary (including Polytechnics, MARA Colleges, University Colleges, and University)	RM 1,001 to 5,000 per month	Employed, part time
Tertiary (including Polytechnics, MARA Colleges, University Colleges, and University)	RM 5,001 - RM 10,000 per month	Employed, full time
Tertiary (including Polytechnics, MARA Colleges, University Colleges, and University)	>RM 10,000 per month	Employed, full time
Tertiary (including Polytechnics, MARA Colleges, University Colleges, and University)	RM 5,001 - RM 10,000 per month	Work from home/housewife
Tertiary (including Polytechnics, MARA Colleges, University Colleges, and University)	RM 5,001 - RM 10,000 per month	Employed, full time
Tertiary (including Polytechnics, MARA Colleges, University Colleges, and University)	>RM 10,000 per month	Employed, full time
Tertiary (including Polytechnics, MARA Colleges, University Colleges, and University)	>RM 10,000 per month	Employed, full time
Tertiary (including Polytechnics, MARA Colleges, University Colleges, and University)	RM 1,001 to 5,000 per month	Employed, full time
Tertiary (including Polytechnics, MARA Colleges, University Colleges, and University)	>RM 10,000 per month	Employed, full time

Tertiary (including Polytechnics, MARA Colleges, University Colleges, and University)	>RM 10,000 per month	Employed, full time
Tertiary (including Polytechnics, MARA Colleges, University Colleges, and University)	RM 5,001 - RM 10,000 per month	Employed, full time
Tertiary (including Polytechnics, MARA Colleges, University Colleges, and University)	RM 5,001 - RM 10,000 per month	Employed, full time
Tertiary (including Polytechnics, MARA Colleges, University Colleges, and University)	RM 5,001 - RM 10,000 per month	Employed, full time
Post-graduates	RM 5,001 - RM 10,000 per month	Employed, full time
Secondary	RM 5,001 - RM 10,000 per month	Employed, full time
Tertiary (including Polytechnics, MARA Colleges, University Colleges, and University)	RM 5,001 - RM 10,000 per month	Employed, full time
Tertiary (including Polytechnics, MARA Colleges, University Colleges, and University)	RM 5,001 - RM 10,000 per month	Employed, full time
Tertiary (including Polytechnics, MARA Colleges, University Colleges, and University)	RM 5,001 - RM 10,000 per month	Employed, full time
Tertiary (including Polytechnics, MARA Colleges, University Colleges, and University)	RM 5,001 - RM 10,000 per month	Employed, full time
Tertiary (including Polytechnics, MARA Colleges, University Colleges, and University)	RM 5,001 - RM 10,000 per month	Work from home/housewife
Tertiary (including Polytechnics, MARA Colleges, University Colleges, and University)	RM 5,001 - RM 10,000 per month	Employed, full time
Secondary	RM 1,001 to 5,000 per month	Work from home/housewife
Tertiary (including Polytechnics, MARA Colleges, University Colleges, and University)	RM 5,001 - RM 10,000 per month	Employed, full time
Secondary	RM 1,001 to 5,000 per month	Employed, full time
Secondary	RM 1,001 to 5,000 per month	Employed, full time
Tertiary (including Polytechnics, MARA Colleges, University Colleges, and University)	RM 1,001 to 5,000 per month	Work from home/housewife
Post-graduates	RM 5,001 - RM 10,000 per month	Employed, full time
Tertiary (including Polytechnics, MARA Colleges, University Colleges, and University)	RM 5,001 - RM 10,000 per month	Employed, full time
Tertiary (including Polytechnics, MARA Colleges, University Colleges, and University)	RM 5,001 - RM 10,000 per month	Employed, full time
Post-graduates	>RM 10,000 per month	Employed, full time
Secondary	RM 5,001 - RM 10,000 per month	Employed, full time
Secondary	RM 5,001 - RM 10,000 per month	Employed, full time
Secondary	RM 1,001 to 5,000 per month	Employed, full time
Tertiary (including Polytechnics, MARA Colleges, University Colleges, and University)	RM 1,001 to 5,000 per month	Employed, full time
Secondary	RM 5,001 - RM 10,000 per month	Employed, full time
Tertiary (including Polytechnics, MARA Colleges, University Colleges, and University)	RM 1,001 to 5,000 per month	Work from home/housewife
Tertiary (including Polytechnics, MARA Colleges, University Colleges, and University)	>RM 10,000 per month	Employed, full time
Post-graduates	>RM 10,000 per month	Employed, full time
Tertiary (including Polytechnics, MARA Colleges, University Colleges, and University)	RM 1,001 to 5,000 per month	Employed, full time
Tertiary (including Polytechnics, MARA Colleges, University Colleges, and University)	RM 5,001 - RM 10,000 per month	Employed, part time
Tertiary (including Polytechnics, MARA Colleges, University Colleges, and University)	>RM 10,000 per month	Temporarily unemployed/medical leave/maternity
Tertiary (including Polytechnics, MARA Colleges, University Colleges, and University)	RM 5,001 - RM 10,000 per month	Employed, full time

Post-graduates	RM 5,001 - RM 10,000 per month	Employed, full time
Tertiary (including Polytechnics, MARA Colleges, University Colleges, and University)	RM 5,001 - RM 10,000 per month	Employed, full time
Post-graduates	RM 5,001 - RM 10,000 per month	Employed, full time
Post-graduates	RM 5,001 - RM 10,000 per month	Employed, full time
Tertiary (including Polytechnics, MARA Colleges, University Colleges, and University)	>RM 10,000 per month	Employed, full time
Secondary	<RM 1,000 per month	Work from home/housewife
Tertiary (including Polytechnics, MARA Colleges, University Colleges, and University)	>RM 10,000 per month	Employed, full time
Post-graduates	>RM 10,000 per month	Employed, full time
Tertiary (including Polytechnics, MARA Colleges, University Colleges, and University)	RM 5,001 - RM 10,000 per month	Temporarily unemployed/medical leave/maternity
Tertiary (including Polytechnics, MARA Colleges, University Colleges, and University)	>RM 10,000 per month	Work from home/housewife
Secondary	RM 5,001 - RM 10,000 per month	Employed, full time
Tertiary (including Polytechnics, MARA Colleges, University Colleges, and University)	RM 5,001 - RM 10,000 per month	Employed, full time
Secondary	RM 1,001 to 5,000 per month	Work from home/housewife
Tertiary (including Polytechnics, MARA Colleges, University Colleges, and University)	>RM 10,000 per month	Work from home/housewife
Tertiary (including Polytechnics, MARA Colleges, University Colleges, and University)	RM 5,001 - RM 10,000 per month	Employed, full time
Tertiary (including Polytechnics, MARA Colleges, University Colleges, and University)	RM 1,001 to 5,000 per month	Employed, full time
Post-graduates	RM 5,001 - RM 10,000 per month	Employed, full time
Post-graduates	RM 5,001 - RM 10,000 per month	Temporarily unemployed/medical leave/maternity
Tertiary (including Polytechnics, MARA Colleges, University Colleges, and University)	RM 5,001 - RM 10,000 per month	Employed, full time
Secondary	>RM 10,000 per month	Employed, full time
Tertiary (including Polytechnics, MARA Colleges, University Colleges, and University)	RM 1,001 to 5,000 per month	Work from home/housewife
Secondary	RM 5,001 - RM 10,000 per month	Employed, full time
Secondary	RM 5,001 - RM 10,000 per month	Work from home/housewife
Tertiary (including Polytechnics, MARA Colleges, University Colleges, and University)	RM 5,001 - RM 10,000 per month	Employed, full time
Secondary	RM 1,001 to 5,000 per month	Employed, full time
Tertiary (including Polytechnics, MARA Colleges, University Colleges, and University)	RM 1,001 to 5,000 per month	Employed, full time
Secondary	RM 1,001 to 5,000 per month	Employed, full time
Post-graduates	>RM 10,000 per month	Employed, full time
Tertiary (including Polytechnics, MARA Colleges, University Colleges, and University)	RM 5,001 - RM 10,000 per month	Employed, full time
Tertiary (including Polytechnics, MARA Colleges, University Colleges, and University)	RM 1,001 to 5,000 per month	Employed, full time
Secondary	RM 5,001 - RM 10,000 per month	Employed, full time
Tertiary (including Polytechnics, MARA Colleges, University Colleges, and University)	RM 5,001 - RM 10,000 per month	Employed, full time
Tertiary (including Polytechnics, MARA Colleges, University Colleges, and University)	>RM 10,000 per month	Employed, full time

Tertiary (including Polytechnics, MARA Colleges, University Colleges, and University)	RM 5,001 - RM 10,000 per month	Employed, full time
Secondary	RM 1,001 to 5,000 per month	Work from home/housewife
Tertiary (including Polytechnics, MARA Colleges, University Colleges, and University)	RM 1,001 to 5,000 per month	Work from home/housewife
Tertiary (including Polytechnics, MARA Colleges, University Colleges, and University)	>RM 10,000 per month	Employed, full time
Tertiary (including Polytechnics, MARA Colleges, University Colleges, and University)	RM 5,001 - RM 10,000 per month	Employed, full time
Tertiary (including Polytechnics, MARA Colleges, University Colleges, and University)	RM 1,001 to 5,000 per month	Employed, full time
Tertiary (including Polytechnics, MARA Colleges, University Colleges, and University)	RM 5,001 - RM 10,000 per month	Employed, full time
Tertiary (including Polytechnics, MARA Colleges, University Colleges, and University)	RM 5,001 - RM 10,000 per month	Employed, full time
Tertiary (including Polytechnics, MARA Colleges, University Colleges, and University)	RM 5,001 - RM 10,000 per month	Employed, full time
Tertiary (including Polytechnics, MARA Colleges, University Colleges, and University)	RM 1,001 to 5,000 per month	Work from home/housewife
Tertiary (including Polytechnics, MARA Colleges, University Colleges, and University)	RM 5,001 - RM 10,000 per month	Work from home/housewife
Tertiary (including Polytechnics, MARA Colleges, University Colleges, and University)	>RM 10,000 per month	Temporarily unemployed/medical leave/maternity
Tertiary (including Polytechnics, MARA Colleges, University Colleges, and University)	RM 5,001 - RM 10,000 per month	Employed, full time
Tertiary (including Polytechnics, MARA Colleges, University Colleges, and University)	>RM 10,000 per month	Employed, full time
Tertiary (including Polytechnics, MARA Colleges, University Colleges, and University)	RM 5,001 - RM 10,000 per month	Employed, full time
Tertiary (including Polytechnics, MARA Colleges, University Colleges, and University)	RM 5,001 - RM 10,000 per month	Employed, full time
Secondary	RM 1,001 to 5,000 per month	Work from home/housewife
Tertiary (including Polytechnics, MARA Colleges, University Colleges, and University)	RM 5,001 - RM 10,000 per month	Work from home/housewife
Tertiary (including Polytechnics, MARA Colleges, University Colleges, and University)	RM 5,001 - RM 10,000 per month	Work from home/housewife
Tertiary (including Polytechnics, MARA Colleges, University Colleges, and University)	RM 5,001 - RM 10,000 per month	Employed, full time
Tertiary (including Polytechnics, MARA Colleges, University Colleges, and University)	RM 5,001 - RM 10,000 per month	Work from home/housewife
Secondary	RM 5,001 - RM 10,000 per month	Employed, full time
Tertiary (including Polytechnics, MARA Colleges, University Colleges, and University)	>RM 10,000 per month	Employed, full time
Secondary	RM 1,001 to 5,000 per month	Employed, full time
Tertiary (including Polytechnics, MARA Colleges, University Colleges, and University)	>RM 10,000 per month	Employed, full time
Tertiary (including Polytechnics, MARA Colleges, University Colleges, and University)	RM 1,001 to 5,000 per month	Employed, full time
Tertiary (including Polytechnics, MARA Colleges, University Colleges, and University)	RM 5,001 - RM 10,000 per month	Employed, full time
Tertiary (including Polytechnics, MARA Colleges, University Colleges, and University)	>RM 10,000 per month	Others
Tertiary (including Polytechnics, MARA Colleges, University Colleges, and University)	>RM 10,000 per month	Employed, full time
Tertiary (including Polytechnics, MARA Colleges, University Colleges, and University)	>RM 10,000 per month	Employed, full time
Secondary	RM 5,001 - RM 10,000 per month	Employed, full time
Tertiary (including Polytechnics, MARA Colleges, University Colleges, and University)	RM 1,001 to 5,000 per month	Work from home/housewife
Post-graduates	RM 5,001 - RM 10,000 per month	Employed, full time

Tertiary (including Polytechnics, MARA Colleges, University Colleges, and University)	RM 5,001 - RM 10,000 per month	Employed, full time
Tertiary (including Polytechnics, MARA Colleges, University Colleges, and University)	RM 5,001 - RM 10,000 per month	Employed, full time
Post-graduates	RM 5,001 - RM 10,000 per month	Employed, full time
Tertiary (including Polytechnics, MARA Colleges, University Colleges, and University)	RM 5,001 - RM 10,000 per month	Employed, full time
Tertiary (including Polytechnics, MARA Colleges, University Colleges, and University)	RM 5,001 - RM 10,000 per month	Employed, full time
Tertiary (including Polytechnics, MARA Colleges, University Colleges, and University)	RM 5,001 - RM 10,000 per month	Employed, full time
Tertiary (including Polytechnics, MARA Colleges, University Colleges, and University)	>RM 10,000 per month	Employed, full time
Tertiary (including Polytechnics, MARA Colleges, University Colleges, and University)	RM 5,001 - RM 10,000 per month	Work from home/housewife
Tertiary (including Polytechnics, MARA Colleges, University Colleges, and University)	RM 5,001 - RM 10,000 per month	Employed, full time
Secondary	RM 1,001 to 5,000 per month	Employed, full time
Tertiary (including Polytechnics, MARA Colleges, University Colleges, and University)	RM 1,001 to 5,000 per month	Employed, full time
Tertiary (including Polytechnics, MARA Colleges, University Colleges, and University)	RM 5,001 - RM 10,000 per month	Employed, full time
Secondary	RM 5,001 - RM 10,000 per month	Employed, full time
Tertiary (including Polytechnics, MARA Colleges, University Colleges, and University)	RM 5,001 - RM 10,000 per month	Employed, full time
Tertiary (including Polytechnics, MARA Colleges, University Colleges, and University)	>RM 10,000 per month	Employed, full time
Tertiary (including Polytechnics, MARA Colleges, University Colleges, and University)	RM 1,001 to 5,000 per month	Employed, part time
Tertiary (including Polytechnics, MARA Colleges, University Colleges, and University)	RM 5,001 - RM 10,000 per month	Employed, full time
Secondary	RM 1,001 to 5,000 per month	Employed, full time
Tertiary (including Polytechnics, MARA Colleges, University Colleges, and University)	RM 1,001 to 5,000 per month	Work from home/housewife
Secondary	RM 1,001 to 5,000 per month	Employed, full time
Tertiary (including Polytechnics, MARA Colleges, University Colleges, and University)	>RM 10,000 per month	Employed, full time
Secondary	<RM 1,000 per month	Work from home/housewife
Tertiary (including Polytechnics, MARA Colleges, University Colleges, and University)	RM 5,001 - RM 10,000 per month	Employed, full time
Tertiary (including Polytechnics, MARA Colleges, University Colleges, and University)	RM 5,001 - RM 10,000 per month	Employed, full time
Secondary	RM 5,001 - RM 10,000 per month	Employed, full time
Tertiary (including Polytechnics, MARA Colleges, University Colleges, and University)	RM 5,001 - RM 10,000 per month	Employed, full time
Tertiary (including Polytechnics, MARA Colleges, University Colleges, and University)	>RM 10,000 per month	Employed, full time
Secondary	RM 1,001 to 5,000 per month	Work from home/housewife
Tertiary (including Polytechnics, MARA Colleges, University Colleges, and University)	RM 5,001 - RM 10,000 per month	Employed, full time
Tertiary (including Polytechnics, MARA Colleges, University Colleges, and University)	>RM 10,000 per month	Employed, full time
Tertiary (including Polytechnics, MARA Colleges, University Colleges, and University)	RM 1,001 to 5,000 per month	Temporarily unemployed/medical leave/maternity
Tertiary (including Polytechnics, MARA Colleges, University Colleges, and University)	RM 5,001 - RM 10,000 per month	Employed, full time
Tertiary (including Polytechnics, MARA Colleges, University Colleges, and University)	>RM 10,000 per month	Employed, full time

Tertiary (including Polytechnics, MARA Colleges, University Colleges, and University)	RM 5,001 - RM 10,000 per month	Employed, full time
Tertiary (including Polytechnics, MARA Colleges, University Colleges, and University)	>RM 10,000 per month	Employed, full time
Tertiary (including Polytechnics, MARA Colleges, University Colleges, and University)	RM 1,001 to 5,000 per month	Employed, full time
Tertiary (including Polytechnics, MARA Colleges, University Colleges, and University)	RM 1,001 to 5,000 per month	Employed, full time
Tertiary (including Polytechnics, MARA Colleges, University Colleges, and University)	RM 5,001 - RM 10,000 per month	Employed, full time
Post-graduates	RM 5,001 - RM 10,000 per month	Employed, full time
Tertiary (including Polytechnics, MARA Colleges, University Colleges, and University)	>RM 10,000 per month	Employed, full time
Tertiary (including Polytechnics, MARA Colleges, University Colleges, and University)	RM 1,001 to 5,000 per month	Employed, full time
Tertiary (including Polytechnics, MARA Colleges, University Colleges, and University)	RM 5,001 - RM 10,000 per month	Employed, full time
Tertiary (including Polytechnics, MARA Colleges, University Colleges, and University)	RM 5,001 - RM 10,000 per month	Employed, full time
Secondary	RM 1,001 to 5,000 per month	Work from home/housewife
Tertiary (including Polytechnics, MARA Colleges, University Colleges, and University)	RM 5,001 - RM 10,000 per month	Employed, full time
Post-graduates	RM 5,001 - RM 10,000 per month	Employed, full time
Tertiary (including Polytechnics, MARA Colleges, University Colleges, and University)	>RM 10,000 per month	Employed, full time
Tertiary (including Polytechnics, MARA Colleges, University Colleges, and University)	RM 5,001 - RM 10,000 per month	Employed, full time
Post-graduates	>RM 10,000 per month	Employed, full time
Tertiary (including Polytechnics, MARA Colleges, University Colleges, and University)	>RM 10,000 per month	Employed, full time
Tertiary (including Polytechnics, MARA Colleges, University Colleges, and University)	>RM 10,000 per month	Employed, full time
Secondary	RM 1,001 to 5,000 per month	Work from home/housewife
Tertiary (including Polytechnics, MARA Colleges, University Colleges, and University)	RM 1,001 to 5,000 per month	Work from home/housewife
Post-graduates	RM 1,001 to 5,000 per month	Employed, full time
Tertiary (including Polytechnics, MARA Colleges, University Colleges, and University)	RM 5,001 - RM 10,000 per month	Work from home/housewife
Tertiary (including Polytechnics, MARA Colleges, University Colleges, and University)	>RM 10,000 per month	Employed, full time
Tertiary (including Polytechnics, MARA Colleges, University Colleges, and University)	RM 5,001 - RM 10,000 per month	Employed, full time
Tertiary (including Polytechnics, MARA Colleges, University Colleges, and University)	RM 5,001 - RM 10,000 per month	Employed, full time
Tertiary (including Polytechnics, MARA Colleges, University Colleges, and University)	RM 1,001 to 5,000 per month	Employed, full time
Tertiary (including Polytechnics, MARA Colleges, University Colleges, and University)	>RM 10,000 per month	Employed, full time
Tertiary (including Polytechnics, MARA Colleges, University Colleges, and University)	RM 5,001 - RM 10,000 per month	Work from home/housewife
Tertiary (including Polytechnics, MARA Colleges, University Colleges, and University)	RM 5,001 - RM 10,000 per month	Employed, full time
Tertiary (including Polytechnics, MARA Colleges, University Colleges, and University)	>RM 10,000 per month	Employed, full time
Secondary	RM 1,001 to 5,000 per month	Employed, full time
Tertiary (including Polytechnics, MARA Colleges, University Colleges, and University)	>RM 10,000 per month	Employed, full time
Post-graduates	>RM 10,000 per month	Employed, full time

Tertiary (including Polytechnics, MARA Colleges, University Colleges, and University)	RM 5,001 - RM 10,000 per month	Employed, full time
Tertiary (including Polytechnics, MARA Colleges, University Colleges, and University)	>RM 10,000 per month	Employed, full time
Secondary	RM 5,001 - RM 10,000 per month	Work from home/housewife
Tertiary (including Polytechnics, MARA Colleges, University Colleges, and University)	>RM 10,000 per month	Employed, full time
Tertiary (including Polytechnics, MARA Colleges, University Colleges, and University)	>RM 10,000 per month	Employed, full time
Secondary	RM 1,001 to 5,000 per month	Employed, full time
Post-graduates	>RM 10,000 per month	Employed, full time
Secondary	RM 1,001 to 5,000 per month	Work from home/housewife
Secondary	RM 5,001 - RM 10,000 per month	Temporarily unemployed/medical leave/maternity
Tertiary (including Polytechnics, MARA Colleges, University Colleges, and University)	RM 1,001 to 5,000 per month	Employed, full time
Tertiary (including Polytechnics, MARA Colleges, University Colleges, and University)	RM 1,001 to 5,000 per month	Work from home/housewife
Tertiary (including Polytechnics, MARA Colleges, University Colleges, and University)	RM 5,001 - RM 10,000 per month	Work from home/housewife
Secondary	RM 5,001 - RM 10,000 per month	Work from home/housewife
Tertiary (including Polytechnics, MARA Colleges, University Colleges, and University)	RM 5,001 - RM 10,000 per month	Employed, full time
Post-graduates	>RM 10,000 per month	Employed, full time
Secondary	RM 1,001 to 5,000 per month	Employed, full time
Tertiary (including Polytechnics, MARA Colleges, University Colleges, and University)	RM 5,001 - RM 10,000 per month	Employed, full time
Tertiary (including Polytechnics, MARA Colleges, University Colleges, and University)	RM 5,001 - RM 10,000 per month	Employed, full time
Secondary	RM 5,001 - RM 10,000 per month	Employed, full time
Tertiary (including Polytechnics, MARA Colleges, University Colleges, and University)	RM 5,001 - RM 10,000 per month	Employed, full time
Tertiary (including Polytechnics, MARA Colleges, University Colleges, and University)	>RM 10,000 per month	Employed, full time
Tertiary (including Polytechnics, MARA Colleges, University Colleges, and University)	RM 1,001 to 5,000 per month	Work from home/housewife
Tertiary (including Polytechnics, MARA Colleges, University Colleges, and University)	RM 1,001 to 5,000 per month	Employed, full time
Tertiary (including Polytechnics, MARA Colleges, University Colleges, and University)	RM 5,001 - RM 10,000 per month	Employed, full time
Tertiary (including Polytechnics, MARA Colleges, University Colleges, and University)	RM 5,001 - RM 10,000 per month	Work from home/housewife
Post-graduates	RM 1,001 to 5,000 per month	Work from home/housewife
Secondary	RM 1,001 to 5,000 per month	Employed, full time
Tertiary (including Polytechnics, MARA Colleges, University Colleges, and University)	RM 5,001 - RM 10,000 per month	Work from home/housewife
Tertiary (including Polytechnics, MARA Colleges, University Colleges, and University)	>RM 10,000 per month	Employed, full time
Tertiary (including Polytechnics, MARA Colleges, University Colleges, and University)	RM 5,001 - RM 10,000 per month	Employed, full time
Tertiary (including Polytechnics, MARA Colleges, University Colleges, and University)	RM 5,001 - RM 10,000 per month	Employed, full time
Secondary	RM 1,001 to 5,000 per month	Work from home/housewife
Tertiary (including Polytechnics, MARA Colleges, University Colleges, and University)	>RM 10,000 per month	Employed, full time

Tertiary (including Polytechnics, MARA Colleges, University Colleges, and University)	RM 5,001 - RM 10,000 per month	Employed, full time
Tertiary (including Polytechnics, MARA Colleges, University Colleges, and University)	RM 5,001 - RM 10,000 per month	Employed, full time
Tertiary (including Polytechnics, MARA Colleges, University Colleges, and University)	>RM 10,000 per month	Employed, full time
Tertiary (including Polytechnics, MARA Colleges, University Colleges, and University)	RM 5,001 - RM 10,000 per month	Employed, full time
Post-graduates	>RM 10,000 per month	Employed, part time
Tertiary (including Polytechnics, MARA Colleges, University Colleges, and University)	RM 1,001 to 5,000 per month	Employed, full time
Tertiary (including Polytechnics, MARA Colleges, University Colleges, and University)	RM 5,001 - RM 10,000 per month	Work from home/housewife
Tertiary (including Polytechnics, MARA Colleges, University Colleges, and University)	>RM 10,000 per month	Work from home/housewife
Tertiary (including Polytechnics, MARA Colleges, University Colleges, and University)	RM 5,001 - RM 10,000 per month	Employed, full time
Tertiary (including Polytechnics, MARA Colleges, University Colleges, and University)	RM 5,001 - RM 10,000 per month	Employed, full time
Tertiary (including Polytechnics, MARA Colleges, University Colleges, and University)	RM 5,001 - RM 10,000 per month	Employed, full time
Tertiary (including Polytechnics, MARA Colleges, University Colleges, and University)	RM 1,001 to 5,000 per month	Work from home/housewife
Tertiary (including Polytechnics, MARA Colleges, University Colleges, and University)	>RM 10,000 per month	Employed, full time
Tertiary (including Polytechnics, MARA Colleges, University Colleges, and University)	RM 5,001 - RM 10,000 per month	Employed, full time
Tertiary (including Polytechnics, MARA Colleges, University Colleges, and University)	RM 5,001 - RM 10,000 per month	Work from home/housewife
Tertiary (including Polytechnics, MARA Colleges, University Colleges, and University)	RM 5,001 - RM 10,000 per month	Employed, full time
Tertiary (including Polytechnics, MARA Colleges, University Colleges, and University)	RM 1,001 to 5,000 per month	Employed, full time
Tertiary (including Polytechnics, MARA Colleges, University Colleges, and University)	RM 5,001 - RM 10,000 per month	Employed, full time
Tertiary (including Polytechnics, MARA Colleges, University Colleges, and University)	>RM 10,000 per month	Employed, full time
Tertiary (including Polytechnics, MARA Colleges, University Colleges, and University)	>RM 10,000 per month	Employed, full time
Tertiary (including Polytechnics, MARA Colleges, University Colleges, and University)	RM 5,001 - RM 10,000 per month	Employed, full time
Tertiary (including Polytechnics, MARA Colleges, University Colleges, and University)	RM 1,001 to 5,000 per month	Work from home/housewife
Tertiary (including Polytechnics, MARA Colleges, University Colleges, and University)	RM 1,001 to 5,000 per month	Employed, full time
Tertiary (including Polytechnics, MARA Colleges, University Colleges, and University)	RM 5,001 - RM 10,000 per month	Employed, full time
Post-graduates	>RM 10,000 per month	Work from home/housewife
Tertiary (including Polytechnics, MARA Colleges, University Colleges, and University)	RM 5,001 - RM 10,000 per month	Employed, full time
Tertiary (including Polytechnics, MARA Colleges, University Colleges, and University)	RM 5,001 - RM 10,000 per month	Employed, full time
Tertiary (including Polytechnics, MARA Colleges, University Colleges, and University)	>RM 10,000 per month	Employed, full time
Secondary	RM 5,001 - RM 10,000 per month	Work from home/housewife
Tertiary (including Polytechnics, MARA Colleges, University Colleges, and University)	RM 5,001 - RM 10,000 per month	Employed, full time
Tertiary (including Polytechnics, MARA Colleges, University Colleges, and University)	>RM 10,000 per month	Employed, full time
Tertiary (including Polytechnics, MARA Colleges, University Colleges, and University)	RM 5,001 - RM 10,000 per month	Work from home/housewife
Tertiary (including Polytechnics, MARA Colleges, University Colleges, and University)	RM 5,001 - RM 10,000 per month	Employed, full time

Tertiary (including Polytechnics, MARA Colleges, University Colleges, and University)	RM 5,001 - RM 10,000 per month	Employed, full time
Post-graduates	RM 5,001 - RM 10,000 per month	Temporarily unemployed/medical leave/maternity
Secondary	RM 1,001 to 5,000 per month	Employed, full time
Tertiary (including Polytechnics, MARA Colleges, University Colleges, and University)	RM 5,001 - RM 10,000 per month	Employed, full time
Tertiary (including Polytechnics, MARA Colleges, University Colleges, and University)	RM 5,001 - RM 10,000 per month	Employed, full time
Tertiary (including Polytechnics, MARA Colleges, University Colleges, and University)	>RM 10,000 per month	Employed, full time
Tertiary (including Polytechnics, MARA Colleges, University Colleges, and University)	RM 1,001 to 5,000 per month	Employed, full time
Tertiary (including Polytechnics, MARA Colleges, University Colleges, and University)	RM 5,001 - RM 10,000 per month	Employed, full time
Secondary	RM 5,001 - RM 10,000 per month	Employed, full time
Tertiary (including Polytechnics, MARA Colleges, University Colleges, and University)	>RM 10,000 per month	Employed, full time
Tertiary (including Polytechnics, MARA Colleges, University Colleges, and University)	>RM 10,000 per month	Employed, full time
Tertiary (including Polytechnics, MARA Colleges, University Colleges, and University)	RM 1,001 to 5,000 per month	Work from home/housewife
Tertiary (including Polytechnics, MARA Colleges, University Colleges, and University)	RM 5,001 - RM 10,000 per month	Work from home/housewife
Tertiary (including Polytechnics, MARA Colleges, University Colleges, and University)	RM 5,001 - RM 10,000 per month	Employed, full time
Post-graduates	>RM 10,000 per month	Employed, full time
Tertiary (including Polytechnics, MARA Colleges, University Colleges, and University)	RM 5,001 - RM 10,000 per month	Employed, full time
Tertiary (including Polytechnics, MARA Colleges, University Colleges, and University)	RM 5,001 - RM 10,000 per month	Employed, full time
Tertiary (including Polytechnics, MARA Colleges, University Colleges, and University)	RM 5,001 - RM 10,000 per month	Employed, full time
Secondary	RM 5,001 - RM 10,000 per month	Temporarily unemployed/medical leave/maternity
Tertiary (including Polytechnics, MARA Colleges, University Colleges, and University)	RM 5,001 - RM 10,000 per month	Employed, full time
Tertiary (including Polytechnics, MARA Colleges, University Colleges, and University)	RM 1,001 to 5,000 per month	Employed, full time
Tertiary (including Polytechnics, MARA Colleges, University Colleges, and University)	RM 1,001 to 5,000 per month	Work from home/housewife
Tertiary (including Polytechnics, MARA Colleges, University Colleges, and University)	>RM 10,000 per month	Employed, full time
Tertiary (including Polytechnics, MARA Colleges, University Colleges, and University)	RM 5,001 - RM 10,000 per month	Employed, full time
Tertiary (including Polytechnics, MARA Colleges, University Colleges, and University)	RM 5,001 - RM 10,000 per month	Employed, full time
Tertiary (including Polytechnics, MARA Colleges, University Colleges, and University)	RM 5,001 - RM 10,000 per month	Work from home/housewife
Tertiary (including Polytechnics, MARA Colleges, University Colleges, and University)	RM 1,001 to 5,000 per month	Work from home/housewife
Tertiary (including Polytechnics, MARA Colleges, University Colleges, and University)	RM 5,001 - RM 10,000 per month	Work from home/housewife
Secondary	RM 1,001 to 5,000 per month	Work from home/housewife
Tertiary (including Polytechnics, MARA Colleges, University Colleges, and University)	>RM 10,000 per month	Others
Tertiary (including Polytechnics, MARA Colleges, University Colleges, and University)	>RM 10,000 per month	Employed, full time
Tertiary (including Polytechnics, MARA Colleges, University Colleges, and University)	RM 5,001 - RM 10,000 per month	Employed, full time
Tertiary (including Polytechnics, MARA Colleges, University Colleges, and University)	RM 5,001 - RM 10,000 per month	Employed, full time

Tertiary (including Polytechnics, MARA Colleges, University Colleges, and University)	RM 5,001 - RM 10,000 per month	Employed, full time
Post-graduates	RM 5,001 - RM 10,000 per month	Employed, full time
Tertiary (including Polytechnics, MARA Colleges, University Colleges, and University)	RM 5,001 - RM 10,000 per month	Others
Secondary	RM 5,001 - RM 10,000 per month	Work from home/housewife
Tertiary (including Polytechnics, MARA Colleges, University Colleges, and University)	RM 5,001 - RM 10,000 per month	Employed, full time
Tertiary (including Polytechnics, MARA Colleges, University Colleges, and University)	RM 5,001 - RM 10,000 per month	Employed, full time
Post-graduates	RM 5,001 - RM 10,000 per month	Employed, full time
Post-graduates	RM 5,001 - RM 10,000 per month	Employed, full time
Tertiary (including Polytechnics, MARA Colleges, University Colleges, and University)	RM 5,001 - RM 10,000 per month	Employed, full time
Tertiary (including Polytechnics, MARA Colleges, University Colleges, and University)	RM 5,001 - RM 10,000 per month	Employed, full time
Tertiary (including Polytechnics, MARA Colleges, University Colleges, and University)	RM 1,001 to 5,000 per month	Work from home/housewife
Secondary	RM 1,001 to 5,000 per month	Employed, full time
Tertiary (including Polytechnics, MARA Colleges, University Colleges, and University)	RM 1,001 to 5,000 per month	Work from home/housewife
Tertiary (including Polytechnics, MARA Colleges, University Colleges, and University)	RM 5,001 - RM 10,000 per month	Employed, full time
Tertiary (including Polytechnics, MARA Colleges, University Colleges, and University)	RM 5,001 - RM 10,000 per month	Employed, full time
Secondary	RM 5,001 - RM 10,000 per month	Work from home/housewife
Tertiary (including Polytechnics, MARA Colleges, University Colleges, and University)	RM 5,001 - RM 10,000 per month	Employed, full time
Tertiary (including Polytechnics, MARA Colleges, University Colleges, and University)	RM 1,001 to 5,000 per month	Employed, full time
Tertiary (including Polytechnics, MARA Colleges, University Colleges, and University)	RM 5,001 - RM 10,000 per month	Employed, full time
Tertiary (including Polytechnics, MARA Colleges, University Colleges, and University)	RM 5,001 - RM 10,000 per month	Employed, full time

Other Employment Status	Estimated Date of Delivery (EDD) or revised EDD (rEDD)	Gestation on recruitment	History of Pap smear	History of HPV vaccination
	15/9/2021	39 weeks	Yes	No
	26/9/2021	37+3 weeks	No	No
	1/10/2021	36+5 weeks	Yes	No
	30/10/2021	32+4 weeks	Yes	No
	1/10/2021	36+5 weeks	No	No
	25/10/2021	33+2 weeks	No	No
	12/10/2021	35+1 weeks	No	No
	24/9/2021	37+5 weeks	Yes	No
	26/9/2021	37+4 weeks	Yes	No
	18/10/2021	34+3 weeks	Yes	No
	17/9/2021	38+6 weeks	No	No
	17/10/2021	34+4 weeks	No	No
	2/10/2021	36+5 weeks	Yes	No
	14/10/2021	35 weeks	Yes	No
	27/9/2021	38+1 weeks	No	No
	19/10/2021	34+2 weeks	No	No
	14/12/2021	26+2 weeks	No	Yes
	3/10/2021	36+4 weeks	No	No
	8/10/2021	36+3 weeks	Yes	No
	14/11/2021	31+1 weeks	Yes	No
	12/10/2021	35+6 weeks	Yes	No
	11/10/2021	36 weeks	Yes	No
/ leave/temporarily disabled/st	20/10/2021	34+5 weeks	Yes	Yes
	30/9/2021	37+4 weeks	Yes	No
	3/10/2021	37+1 weeks	Yes	Yes
	11/10/2021	36 weeks	Yes	No
	5/10/2021	36+6 weeks	No	No
	29/9/2021	37+6 weeks	No	No
	20/9/2021	39+1 WEEKS	No	No
	20/10/2021	34+6 WEEKS	Yes	No
	15/10/2021	35+4 WEEKS	No	No
	23/10/2021	34+3 WEEKS	Yes	No

	1/10/2021	37+4 WEEKS	No	No
	28/9/2021	38 WEEKS	Yes	Yes
	5/10/2021	37 WEEKS	No	No
	27/9/2021	38+2 WEEKS	No	No
	2/10/2021	37+4 WEEKS	No	Yes
	31/10/2021	34+1 WEEKS	No	No
	31/10/2021	34+1 WEEKS	No	No
	11/10/2021	37 WEEKS	Yes	No
	7/10/2021	37+4 WEEKS	Yes	Yes
	15/10/2021	36+4 WEEKS	No	No
	23/1/2022	22+2 WEEKS	No	No
	28/9/2021	39 WEEKS	No	No
	8/2/2022	20+1 WEEKS	Yes	Yes
	27/10/2021	35 WEEKS	Yes	No
	17/12/2021	27+5 WEEKS	Yes	No
	9/3/2022	16+1 WEEKS	Yes	No
	21/1/2022	22+5 WEEKS	Yes	No
	5/12/2021	29+3 WEEKS	No	No
y leave/temporarily disabled/st	9/11/2021	33+! WEEKS	Yes	No
	22/10/2021	35+5 WEEKS	Yes	No
	20/12/2021	27+2 WEEKS	Yes	No
	14/11/2021	32+3 WEEKS	Yes	No
	25/10/2021	35+2 WEEKS	No	No
	26/12/2021	26+3 WEEKS	Yes	No
	2/3/2022	17+2 WEEKS	No	No
	11/12/2021	28+5 WEEKS	No	No
	14/11/2021	32+4 WEEKS	No	No
	15/11/2021	32+3 WEEKS	No	No
	24/1/2022	22+3 WEEKS	No	No
	20/12/2021	27+3 WEEKS	No	No
	12/12/2021	28+4 WEEKS	Yes	No
	21/11/2021	31+4 WEEKS	Yes	No
	29/11/2021	30+3 WEEKS	No	No

19/2/2022	18+6 WEEKS	Yes	No
14/10/2021	37 WEEKS	No	No
25/10/2021	36 WEEKS	No	No
1/12/2021	30+5 WEEKS	No	No
18/10/2021	37+2 WEEKS	Yes	No
9/11/2021	34+1 WEEKS	Yes	No
11/2/2022	20+5 WEEKS	No	No
29/11/2021	31+2 WEEKS	Yes	No
16/12/2021	28+6 WEEKS	Yes	No
11/10/2021	38+2 weeks	Yes	No
27/2/2022	18+4 weeks	Yes	No
2/10/2021	39+4 weeks	No	No
2/12/2021	30+6 weeks	No	No
18/11/2021	35 weeks	Yes	No
27/10/2021	36+5 weeks	No	No
24/12/2021	28+4 weeks	Yes	No
15/12/2021	29+6 weeks	Yes	No
24/1/2022	24+2 weeks	No	No
29/10/2021	36+5 weeks	Yes	No
29/11/2021	32+2 weeks	Yes	No
14/1/2022	25+5 weeks	No	No
17/1/2022	25+2 weeks	No	No
10/2/2022	21+6 weeks	No	No
19/11/2021	33+6 weeks	Yes	No
18/1/2022	25+6 weeks	No	No
12/1/2022	26+5 weeks	No	No
9/11/2021	35+6 weeks	Yes	No
11/10/2021	40+2 weeks	No	No
26/10/2021	38+1 weeks	No	No
17/11/2021	35 weeks	Yes	No
24/12/2021	29+5 weeks	Yes	No
29/11/2021	33+2 weeks	Yes	No
24/2/2022	21 weeks	No	No

25/11/2021	34 weeks	Yes	No
18/1/2022	26+2 weeks	No	No
3/2/2022	24 weeks	No	No
21/3/2022	17+4 weeks	No	No
13/11/2021	35+5 weeks	No	No
8/1/2022	27+5 weeks	No	No
4/1/2022	28+2 weeks	No	No
23/11/2021	34+6 weeks	Yes	No
13/2/2022	23+1 weeks	No	No
9/11/2021	37+1 weeks	No	No
15/11/2021	36+2 weeks	Yes	No
8/11/2021	38+2 weeks	Yes	No
4/2/2022	25+5 weeks	No	No
12/1/2022	29 weeks	Yes	No
14/3/2022	21 weeks	No	No
27/12/2021	32 weeks	No	No
17/12/2021	33+3 weeks	No	Yes
19/2/2022	24+4 weeks	No	No
17/11/2021	38 weeks	Yes	No
6/12/2021	35+2 weeks	Yes	No
11/12/2021	34+4 weeks	No	No
6/2/2022	27+1 weeks	Yes	No
15/12/2021	35 weeks	Yes	No
6/3/2022	23+3 weeks	Yes	No
2/3/2022	24+5 weeks	No	No
16/12/2021	35+4 weeks	Yes	No
24/4/2022	18+2 weeks	No	No
8/4/2022	20+3 weeks	Yes	No
14/12/2021	36+6 weeks	No	No
7/1/2022	33+3 weeks	No	No
15/3/2022	23+6 weeks	Yes	No
13/1/2022	32+4 weeks	Yes	No
15/3/2022	23+6 weeks	Yes	No

13/1/2022	32+4 weeks	Yes	No
3/5/2022	17 weeks	Yes	No
22/3/2022	22+6 weeks	Yes	No
28/2/2022	26+1 weeks	Yes	No
30/12/2021	34+5 weeks	Yes	No
6/3/2022	25+2 weeks	No	No
6/1/2022	33+5 weeks	No	No
10/4/2022	20+2 weeks	No	No
4/12/2021	38+3 weeks	Yes	No
21/12/2021	36 weeks	Yes	No
25/12/2021	35+3 weeks	Yes	No
26/12/2021	35+3 weeks	Yes	No
18/2/2022	27+5 weeks	No	No
25/1/2022	31+1 weeks	Yes	No
2/12/2021	38+6 weeks	No	No
13/3/2022	24+3 weeks	Yes	No
6/1/2022	33+6 weeks	No	No
31/12/2021	34+5 weeks	No	No
26/3/2022	22+4 weeks	No	No
6/12/2021	38+3 weeks	No	No
31/12/2021	34+6 weeks	Yes	No
17/2/2022	28 weeks	No	No
29/12/2021	35+1 weeks	No	No
20/3/2022	23+4 weeks	No	No
13/1/2022	33+5 weeks	No	No
19/2/2022	28+3 weeks	No	No
19/12/2021	37+2 weeks	No	No
20/2/2022	28+4 weeks	Yes	No
7/5/2022	17+6 weeks	Yes	Yes
4/1/2022	36+1 weeks	No	No
16/4/2022	21+4 weeks	No	No
24/3/2022	24+6 weeks	Yes	No
16/2/2022	30+1 weeks	No	No

23/2/2022	29+1 weeks	Yes	No
25/2/2022	29+4 weeks	No	No
8/3/2022	28+2 weeks	Yes	Yes
28/3/2022	25+3 weeks	Yes	No
18/2/2022	31+4 weeks	No	No
11/3/2022	28+4 weeks	No	No
14/2/2022	32+1 weeks	Yes	No
25/1/2022	35+2 weeks	Yes	No
2/2/2022	35+6 weeks	No	No
15/3/2022	30+2 weeks	Yes	No
19/2/2022	33+5 weeks	No	No
20/2/2022	33+4 weeks	No	No
21/5/2022	20+5 weeks	No	No
3/5/2022	23+2 weeks	Yes	No
3/4/2022	28+4 weeks	Yes	No
3/3/2022	33 weeks	Yes	No
31/3/2022	29 weeks	Yes	No
14/3/2022	31+3 weeks	Yes	No
4/5/2022	24+1 weeks	Yes	No
12/2/2022	36+5 weeks	No	No
10/2/2022	37 weeks	No	No
8/3/2022	34 weeks	Yes	No
28/2/2022	35+4 weeks	Yes	No
9/5/2022	25+3 weeks	No	No
10/2/2022	38 weeks	Yes	No
9/3/2022	34+1 weeks	No	No
2/5/2022	26+3 weeks	No	No
25/3/2022	31+6 weeks	Yes	No
23/3/2022	32+1 weeks	Yes	No
16/4/2022	30+2 weeks	No	No
22/4/2022	29+5 weeks	No	No
11/4/2022	31+2 weeks	No	No
21/3/2022	34+2 weeks	No	No

	9/3/2022	36+1 weeks	Yes	No
	7/3/2022	36+3 weeks	Yes	No
	18/3/2022	34+6 weeks	Yes	No
	11/3/2022	35+6 weeks	No	No
	6/5/2022	28+4 weeks	No	No
	27/4/2022	29+6 weeks	Yes	No
	21/7/2022	18+4 weeks	Yes	No
	7/3/2022	38+3 weeks	No	No
	12/5/2022	29 weeks	Yes	No
	17/3/2022	37 weeks	No	No
/ leave/temporarily disabled/st	21/5/2022	27+5 weeks	No	No
	3/5/2022	32 weeks	No	No
	26/4/2022	33 weeks	No	No
	27/6/2022	27+1 weeks	No	No
	20/7/2022	24 +6 weeks	Yes	No
	1/5/2022	36+4 weeks	No	Yes
	15/6/2022	30+1 weeks	No	No
	30/5/2022	32+3 weeks	No	No
	11/8/2022	26weeks + 5day	No	No
	13/8/2022	28weeks + 4 days	Yes	No
	30/8/2022	26weeks + 1day	Yes	No
	20/7/2022	32 weeks	No	Yes
/ leave/temporarily disabled/st	6/7/2022	34weeks + 6 days	No	No
	3/7/2022	37weeks + 1 day	No	No
	9/7/2022	36weeks 2 days	No	Yes
	5/7/2022	36weeks 6 days	No	No
	9/7/2022	36 weeks 2 days	No	No
	10/8/2022	31weeks 5days	Yes	No
/ leave/temporarily disabled/st	13/7/2022	35weeks 5days	No	No
	7/8/2022	32weeks 3 days	Yes	No
	12/8/2022	31 weeks + 6 days	No	No
	16/7/2022	35weeks 5 days	Yes	No
	17/9/2022	26weeks 6days	Yes	No

	5/9/2022	28weeks 4days	Yes	No
	15/11/2022	18weeks 3days	No	No
	19/9/2022	27weeks	No	No
	8/11/2022	19weeks 6days	Yes	No
	25/9/2022	26weeks 1 day	No	No
	22/10/2022	22weeks 2 days	No	No
	6/9/2022	28weeks 6days	Yes	No
	13/9/2022	28weeks	Yes	No
	20/11/2022	18weeks 2days	No	No
	18/8/2022	31weeks 6days	Yes	No
	19/8/2022	31weeks 5days	No	No
	10/7/2022	37weeks 3days	Yes	No
	15/8/2022	32weeks 3days	No	No
	18/10/2022	23weeks 2days	Yes	No
	30/6/2022	39weeks 0days	No	No
	11/10/2022	24weeks 2days	No	No
	27/9/2022	26weeks 2days	No	No
y leave/temporarily disabled/st	1/8/2022	34weeks 3days	No	No
y leave/temporarily disabled/st	28/7/2022	35weeks 1 day	No	No
	10/9/2022	28weeks 6days	Yes	No
	9/9/2022	29weeks 0days	Yes	No
	25/10/2022	22weeks 6days	Yes	Yes
	8/12/2022	16weeks 4days	Yes	No
	29/7/2022	35weeks 3days	Yes	No
	2/11/2022	21weeks 5days	Yes	No
	3/7/2022	39weeks 1day	No	No
	2/7/2022	39weeks 2days	Yes	No
	4/11/2022	21weeks 4days	Yes	No
	31/8/2022	30weeks 6days	Yes	No
	20/10/2022	23weeks 5days	Yes	No
	19/8/2022	32weeks 4days	No	No
	19/7/2022	37weeks	Yes	No
Business	12/7/2022	38weeks	Yes	No

11/7/2022	38weeks 1day	No	No
5/7/2022	39weeks	Yes	No
13/8/2022	33weeks 5days	Yes	No
2/11/2022	22weeks 5days	Yes	No
24/8/2022	32weeks 5days	No	No
20/8/2022	33weeks 2days	No	No
9/11/2022	21weeks 5days	No	No
11/11/2022	21weeks 3days	Yes	No
25/8/2022	32weeks 4days	No	No
15/10/2022	25weeks 2days	Yes	No
17/7/2022	38weeks 2days	Yes	No
5/7/2022	40weeks 0days	Yes	No
28/7/2022	36weeks 5days	Yes	No
3/9/2022	31weeks 4days	Yes	No
29/7/2022	36weeks 5days	No	No
30/11/2022	19weeks 1day	Yes	No
2/10/2022	27weeks 4days	Yes	No
23/7/2022	37weeks 5days	Yes	No
24/7/2022	37weeks 5days	No	No
25/8/2022	33weeks 1day	No	No
13/7/2022	39weeks 6days	No	No
20/7/2022	38weeks 6days	No	No
19/7/2022	39weeks 4days	Yes	No
15/10/2022	27weeks 0days	No	No
9/9/2022	32weeks 1day	Yes	No
10/10/2022	28weeks	No	No
25/9/2022	30weeks 1day	No	No
10/10/2022	28weeks	Yes	No
7/8/2022	37weeks 1day	No	No
15/11/2022	22weeks 6days	Yes	No
19/8/2022	35weeks 3days	Yes	No
27/11/2022	21weeks 1day	Yes	No
3/8/2022	37weeks 5days	Yes	No

	2/8/2022	37weeks 6days	Yes	No
	6/8/2022	37weeks 2days	Yes	No
	22/7/2022	39weeks 2days	No	No
	30/7/2022	38weeks 6days	No	No
	13/11/2022	24weeks 1day	No	No
	22/8/2022	37weeks 0day	No	No
	9/10/2022	30weeks 1day	No	No
	24/9/2022	32weeks 2days	Yes	No
	14/11/2022	25week 0days	No	No
	28/8/2022	36weeks 1day	No	Yes
	23/10/2022	28weeks 1day	Yes	No
	7/11/2022	26weeks 0days	Yes	No
	25/9/2022	32weeks 1day	Yes	No
	23/8/2022	37weeks 3days	Yes	No
	18/9/2022	34weeks 1day	No	No
	20/8/2022	38weeks 4days	No	No
	30/8/2022	37weeks	Yes	No
	25/10/2022	29weeks	Yes	No
	28/8/2022	37weeks 2days	No	No
	7/9/2022	36weeks 5days	No	No
	10/9/2022	36weeks 2days	Yes	No
	3/11/2022	28weeks 4days	No	No
	10/11/2022	27weeks 4days	No	No
	6/9/2022	37weeks	No	No
	23/8/2022	39weeks	Yes	No
	22/8/2022	39weeks 1day	No	No
	28/10/2022	30weeks 3days	Yes	No
	30/11/2022	25weeks 5days	No	No
	18/10/2022	31weeks 6days	Yes	No
Business	22/9/2022	35weeks 4days	Yes	No
	14/11/2022	28weeks	Yes	No
	22/8/2022	40weeks	No	No
	27/8/2022	39weeks 2days	No	No

24/9/2022	36weeks 2day	Yes	No
26/9/2022	36weeks	No	No
28/1/2023	18weeks 2days	No	No
19/2/2023	15weeks 1day	Yes	No
20/9/2022	37weeks 0days	No	No
25/10/2022	31weeks 6days	Yes	No
5/9/2022	39weeks	No	No
21/11/2022	28weeks 1day	Yes	Yes
6/12/2022	26weeks 0days	No	No
11/1/2023	20weeks 6days	Yes	No
8/12/2022	25weeks 5days	No	No
14/9/2022	38weeks 0days	Yes	No
16/9/2022	37weeks 5days	No	No
22/11/2022	29weeks 2days	Yes	No
9/9/2022	39weeks 6days	No	No
8/1/2023	23weeks 3days	Yes	No
28/9/2022	38weeks 0days	No	No
30/9/2022	37weeks 6days	No	No
3/11/2022	34weeks 3days	No	No
29/1/2023	22weeks 3days	Yes	No
17/10/2022	38weeks 0days	No	No
4/10/2022	37weeks 0days	Yes	No
5/11/2022	35weeks 3days	Yes	Yes
25/10/2022	37weeks 0days	No	No
8/12/2022	33weeks 5days	No	No
8/11/2022	38weeks 0days	Yes	No
19/4/2023	15weeks	No	No
22/12/2022	32weeks 0days	Yes	Yes
11/12/2022	33weeks 4days	No	No
8/12/2022	34weeks 1day	Yes	No
7/3/2023	21weeks 6days	No	No
12/1/2023	29weeks 4days	No	No
21/2/2023	23weeks 6days	No	No

	18/11/2022	37weeks 3days	Yes	No
	26/11/2022	36 weeks 3 days	Yes	No
	6/11/2022	39+1	Yes	No
	20/3/2023	20+2	Yes	No
	19/11/2022	37+3	Yes	No
	13/12/2022	34weeks 0days	Yes	No
	15/2/2023	25weeks 0days	Yes	No
	21/1/2023	28weeks 4days	No	No
	12/2/2023	25+3	No	No
	9/11/2022	39 weeks	Yes	No
	10/4/2023	17weeks 2days	Yes	No
	12/1/2023	30 weeks	No	No
	25/11/2022	36weeks 5days	Yes	No
	5/4/2023	18 weeks	Yes	No
	21/12/2022	34 weeks + 2 days	Yes	No
	20/11/2022	37weeks 3days	No	No
	17/12/2022	35+1	Yes	No
	7/3/2023	22 weeks 2 days	No	Yes
	17/12/2022	19 weeks 6 days	No	No
	4/1/2023	28 weeks 3 days	Yes	No
	8/2/2023	26 weeks	No	Yes
	5/4/2023	18 weeks + 1 day	No	No
	20/12/2022	33 weeks 3 days	Yes	No
	20/1/2023	29 weeks.	Yes	No
	31/1/2023	27 weeks 3 days	Yes	No
	4/2/2023	26+6	No	No
	14/3/2023	21 weeks 3 days	Yes	No
	23/3/2023	20+4	Yes	No
y leave/temporarily disabled/st	3/1/2023	31+6	Yes	No
	27/11/2022	37+1	No	No
	5/2/2023	27+1	Yes	No
	25/4/2023	16 weeks	Yes	No
y leave/temporarily disabled/st	6/3/2023	23 weeks	Yes	No

	19/11/2022	38+2	Yes	No
	9/4/2023	18+2	No	No
	22/2/2023	24+6	Yes	No
	27/12/2022	33 weeks	Yes	Yes
/ leave/temporarily disabled/st	4/1/2023	31+6	Yes	No
	18/2/2023	25+3	Yes	No
	15/12/2022	34+6	No	No
	11/2/2023	26+4	Yes	No
	11/4/2023	18+1	Yes	No
	5/12/2022	36+2	No	No
	2/2/2023	27+6	No	No
	25/1/2023	29 weeks	Yes	Yes
	2/12/2022	36+6	No	Yes
	18/12/2022	34weeks 3days	No	No
	27/11/2022	37weeks 3 days	Yes	No
	5/12/2022	36weeks 2days	Yes	No
	19/1/2023	30weeks 0days	No	No
	29/3/2023	20weeks 1 day	Yes	No
	11/11/2022	39+6	No	No
	22/2/2023	25weeks 1day	Yes	No
	28/11/2022	37+3	Yes	Yes
	7/2/2023	27weeks 2days	No	No
/ leave/temporarily disabled/st	27/11/2022	37+4	Yes	No
	28/1/2023	28weeks 5days	Yes	No
	20/12/2022	34+2	Yes	No
	17/1/2023	30+2	Yes	No
	10/11/2022	40 weeks	No	Yes
	30/3/2023	20 weeks	No	No
	25/11/2022	37weeks 6days	Yes	No
	28/11/2022	37weeks 3days	No	No
	1/12/2022	37weeks 0days	Yes	No
	19/4/2023	17+2	Yes	No
	29/1/2023	37+3	No	No

	23/2/2023	25+1	Yes	No
	2/3/2023	24+1	Yes	No
	27/3/2023	20weeks 5days	No	No
	4/12/2022	37weeks 0days	No	No
	26/12/2022	34 weeks	No	Yes
	23/4/2023	17+1	No	No
	6/4/2023	19+4	Yes	No
	8/12/2022	36+4	Yes	No
	16/1/2023	31+1	No	Yes
	14/1/2023	31+2	Yes	No
	2/2/2023	28+5	No	No
	22/12/2022	34+5	No	No
	29/11/2022	38+2	Yes	No
	10/2/2023	27+4	No	No
	29/11/2022	38 weeks	No	No
	13/2/2023	27+2	No	No
y leave/temporarily disabled/st	28/12/2022	34 weeks	Yes	No
	30/3/2023	20+6	Yes	No
	4/2/2023	28+4	No	No
	2/2/2023	28weeks 6 days	No	No
	26/4/2023	17 weeks	Yes	No
	8/2/2023	28 weeks	Yes	No
	26/11/2022	38+4	No	No
	28/11/2022	38weeks 2 days	No	Yes
	13/2/2023	27+2	Yes	No
	22/3/2023	22+1	Yes	No
	9/3/2023	24weeks 0days	Yes	No
	13/12/2022	36weeks 2days	Yes	No
	11/3/2023	23+5	No	No
	19/3/2023	22weeks 4days	No	No
	6/3/2023	24+3	Yes	No
	23/3/2023	22 weeks	No	No
	5/2/2023	28weeks 4days	No	No

	19/3/2023	22+4	Yes	Yes
	3/12/2022	37weeks 5days	No	No
	10/1/2023	32+6	Yes	No
	14/2/2023	27+6	Yes	No
	24/1/2023	30+6	No	No
	11/1/2023	32+6	Yes	No
	9/12/2022	37+4	Yes	Yes
	29/4/2023	17+3	Yes	No
	12/12/2022	37+1	Yes	No
/ leave/temporarily disabled/st	1/5/2023	17+1	No	Yes
	2/4/2023	21+2	No	No
	30/1/2023	30+1	No	No
	27/12/2022	35weeks 1day	Yes	No
	8/2/2023	29 weeks	No	No
	27/1/2023	30+5	No	No
	17/12/2022	36+4	Yes	No
	4/2/2023	29weeks 4days	Yes	Yes
	19/1/2023	31+6	No	No
	20/12/2022	36+1	Yes	Yes
	30/12/2022	34weeks 5days	No	No
	16/11/2022	41weeks 0days	No	No
	14/2/2023	28+2	No	No
	21/2/2023	27+2	No	No
	15/12/2022	37 weeks	No	No
	15/12/2022	37weeks 0days	Yes	No
	12/4/2023	20+1	No	Yes
	20/12/2022	36weeks 2days	Yes	No
	7/1/2023	33weeks 5days	Yes	Yes
	7/1/2023	33+5	No	No
	28/12/2022	35weeks 1day	Yes	No
	16/1/2023	32weeks 3days	Yes	No
	30/11/2022	39weeks 1day	Yes	No
	14/12/2022	37+2	Yes	Yes

	7/1/2023	33+6	Yes	No
	3/3/2023	26 weeks	Yes	No
	10/2/2023	29 weeks	No	No
	31/12/2022	35+3	Yes	No
	7/1/2023	34+3	Yes	No
ι leave/temporarily disabled/st	20/12/2022	37 weeks	Yes	Yes
	18/1/2023	32+6	No	No
	24/2/2023	27+4	Yes	No
	5/1/2023	34+5	No	No
	25/2/2023	27+4	No	Yes
	26/2/2023	27+3	No	No
	30/4/2023	18+3	Yes	No
	3/3/2023	26+5	Yes	No
	27/4/2023	18+6	Yes	No
	17/2/2023	28+6	Yes	No
	25/4/2023	19+3	Yes	No
	22/2/2023	28+1	Yes	No
	26/12/2022	36+3	Yes	No
	12/3/2023	25+4	No	No
	20/12/2022	37+2	Yes	No
	27/4/2023	19 weeks	Yes	No
	21/2/2023	28+3	Yes	No
	11/5/2023	17+1	No	No
	3/4/2023	22+4	No	Yes
	14/2/2023	29+3	Yes	No
	8/1/2023	35+1	Yes	Yes
	3/2/2023	31+3	No	No
	31/1/2023	31+6	Yes	No
	19/3/2023	25+1	Yes	No
	30/12/2022	36+3	No	No
	12/1/2023	34+4	No	No
	2/1/2023	36 weeks	Yes	No
	24/1/2023	32+6	No	No

	23/4/2022	20+2	No	No
	8/4/2023	22+3	No	No
	3/1/2023	36 weeks	No	No
	10/2/2023	30+4	Yes	No
	8/5/2023	18+1	Yes	No
	29/3/2023	23+6	Yes	No
	27/1/2023	32+4	No	No
	9/3/2023	26+5	No	Yes
	21/12/2022	37+6	Yes	No
	25/12/2022	37+3	No	Yes
	28/2/2023	28+1	Yes	No
	4/2/2023	31+4	No	No
	11/2/2023	30+4	Yes	No
	17/12/2022	38+4	No	No
	25/4/2023	20 weeks	No	No
	9/4/2023	22+3	No	No
	3/2/2023	31+6	No	Yes
	9/3/2023	27 weeks	Yes	No
	3/3/2023	27+6	Yes	Yes
/ leave/temporarily disabled/st	26/12/2022	37+3	No	No
	11/5/2023	18 weeks	Yes	No
	2/5/2023	19+2	Yes	No
	19/2/2023	29+4	Yes	No
/ leave/temporarily disabled/st	1/4/2023	23+5	Yes	No
	6/1/2023	35+6	No	No
	15/2/2023	30+1	No	No
	5/2/2023	31+5	No	No
	15/2/2023	30+2	Yes	No
	6/1/2023	36+3	Yes	No
	24/2/2023	29+3	No	No
	21/3/2023	25+6	Yes	No
	26/1/2023	33+4	Yes	No
	3/5/2023	19+5	No	No

	30/12/2022	37+3	No	No
	25/1/2023	33+5	Yes	No
	21/3/2023	25+6	Yes	No
	31/5/2023	17+1	No	No
	31/1/2023	33 weeks	Yes	No
	15/5/2023	18+1	Yes	No
	22/2/2023	29+6	No	No
	17/2/2023	30+4	No	No
	16/5/2023	18 weeks	Yes	No
	25/12/2022	38+2	Yes	No
	5/2/2023	32+2	No	No
	23/12/2022	38+5	No	No
	5/3/2023	28+3	Yes	No
y leave/temporarily disabled/st	7/2/2023	32+1	No	No
	26/2/2023	29+3	No	No
	24/4/2023	21+2	No	Yes
	2/4/2023	24+3	No	No
	9/5/2023	19+1	Yes	No
	4/3/2023	28+5	Yes	No
	12/2/2023	31+4	No	No
	20/4/2023	22 weeks	Yes	No
	19/2/2023	30+4	Yes	No
	27/4/2023	21 weeks	No	No
	13/2/2023	31 + 3	Yes	No
	27/3/2023	25+3	No	No
	31/1/2023	33+2	No	No
	9/1/2023	36+3	No	No
	6/3/2023	28+3	Yes	Yes
	8/3/2023	28+2	Yes	No
	5/3/2023	29+1	Yes	No
	25/2/2023	30+2	Yes	No
	10/3/2023	28+3	No	No
	29/3/2023	25+5	No	No

	24/3/2023	26+3		Yes	No
	28/12/2022	38+5		Yes	No
	7/2/2023	32+6		No	No
	10/4/2023	24+1		Yes	No
	14/5/2023	19+2		No	No
	31/5/2023	16+2		No	No
	18/3/2023	27+3		Yes	No
	5/5/2023	20+4		Yes	No
	31/5/2023	16+6		Yes	No
	9/6/2023	15+4		Yes	No
	28/3/2023	26 weeks		No	No
	12/4/2023	25+6		No	No
	31/1/2023	36 weeks		Yes	No
	17/2/2023	33+4		No	No
	28/1/2023	36+3		No	No
	13/3/2023	30+1		No	No
	17/4/2023	25+2		Yes	No
	24/2/2023	32+5		Yes	No
	16/3/2023	29+6		Yes	No
	5/2/2023	35+3		Yes	No
	8/5/2023	20 weeks		No	Yes
	5/2/2023	35+3		Yes	No
	24/2/2023	32+5		Yes	No
	27/2/2023	32+2		Yes	No
	15/5/2023	21+3		Yes	No
	30/1/2023	36+3		No	No
	19/5/2023	20+6		No	No
	20/2/2023	33+3		Yes	No
y leave/temporarily disabled/st	2/2/2023		36	Yes	No
	26/3/2023	28+4		Yes	No
	9/4/2023	26+4		Yes	No
	17/3/2023	29+6		Yes	No
	13/4/2023	26 weeks		Yes	No

19/6/2023	16+4	No	No
27/2/2023	33 weeks	Yes	Yes
2/4/2023	28+1	Yes	No
30/3/2023	28+4	No	Yes
5/3/2023	32+1	No	No
17/2/2023	34+3	No	No
26/5/2023	20+3	Yes	No
1/3/2023	32+6	Yes	No
2/6/2023	19+3	No	No
1/6/2023	19+4	Yes	No
9/6/2023	18+3	Yes	No
15/6/2023	17+6	No	Yes
8/3/2023	32 weeks	No	No
21/5/2023	21+3	Yes	Yes
17/2/2023	34+5	No	No
2/2/2023	36+6	No	No
10/3/2023	31+5	Yes	Yes
29/1/2023	37+4	No	Yes
28/1/2023	37+5	No	Yes
8/4/2023	27+5	No	Yes
23/3/2023	30 weeks	No	No
11/4/2023	27+2	Yes	No
19/5/2023	21+6	Yes	No
19/6/2023	17+3	I don't know	No
25/3/2023	29+5	Yes	Yes
21/6/2023	17+1	Yes	No
20/3/2023	30+4	Yes	No
8/4/2023	27+6	No	No
19/6/2023	17+4	Yes	Yes
22/5/2023	22 weeks	Yes	No
22/4/2023	26+2	No	No
5/7/2023	15+5	Yes	No
1/3/2023	33+5	No	No

	1/7/2023	16+2	No	No
	23/6/2023	17+3	No	No
	28/4/2023	25+3	No	No
	30/3/2023	29+4	No	No
	8/4/2023	28+3	No	No
	16/2/2023	35+5	Yes	No
	3/3/2023	33+4	No	No
	25/5/2023	21+5	Yes	No
	8/4/2023	28+3	No	Yes
	4/4/2023	29 weeks	Yes	No
	11/3/2023	32+3	No	No
	14/4/2023	27+4	Yes	No
	23/3/2023	30+6	Yes	No
	24/4/2023	22 weeks	Yes	No
	14/5/2023	23+3	No	No
	19/4/2023	27 weeks	Yes	Yes
	4/7/2023	28+5	No	No
	1/3/2023	34weeks	No	No
	27/3/2023	30+2	No	No
	18/4/2023	27+2	Yes	Yes
	3/3/2023	33+6	Yes	No
	17/5/2023	23+1	No	No
	25/3/2023	30+5	Yes	No
y leave/temporarily disabled/st	9/5/2023	24+2	Yes	Yes
	25/4/2023	26+2	No	No
	18/5/2023	23 weeks	Yes	No
	17/6/2023	18+5	No	No
	12/2/2023	36+4	No	Yes
	19/2/2023	35+4	Yes	No
	7/3/2023	34+1	No	No
	2/3/2023	34+6	No	No
	24/3/2023	31+5	Yes	No
	8/3/2023	34 weeks	Yes	Yes

	18/3/2023	32+4		No	No
	6/2/2023	38+2		No	No
	21/3/2023	32+2		Yes	No
	26/3/2023	31+4		Yes	No
	14/5/2023	24+4		Yes	No
	24/4/2023	27+2		Yes	No
	19/4/2023	28+1		No	Yes
	2/6/2023	21+6		Yes	No
	4/6/2023	21+4		Yes	No
	16/3/2023	33 weeks		No	No
	5/3/2023	34+5		Yes	Yes
	14/3/2023	33+6		Yes	No
	17/3/2023	33+3		Yes	No
	19/7/2023	15+5		No	No
	1/3/2023	35+5		Yes	No
	22/6/2023	19+4		Yes	No
	18/4/2023	28+6		Yes	No
	11/5/2023	25+5		Yes	No
	28/2/2023	36weeks		Yes	No
	20/3/2023	33+1		No	No
	3/5/2023	26+6		Yes	No
	25/2/2023	36+3		Yes	No
	4/5/2023	26+5		Yes	No
	10/3/2023	34+4		No	No
	17/2/2023	37+4		No	No
	18/2/2023	37+5		Yes	No
	29/5/2023	23+3		No	Yes
	18/4/2023	29+2		No	No
	27/7/2023		15	No	No
	26/2/2023	36+4		No	No
	11/3/2023	34+5		Yes	No
	22/5/2023	24+6		Yes	Yes
y leave/temporarily disabled/st	5/5/2023	27 weeks		No	No

27/5/2023	23+6		Yes	No
26/3/2023	32+5		No	No
12/5/2023	26+4		Yes	No
16/5/2023		26	No	Yes
6/4/2023	31+5		Yes	Yes
22/2/2023	37+6		No	No
16/3/2023	34+5		No	No
10/7/2023	18+1		No	No
3/5/2023	27+6		Yes	No
18/2/2023	38+3		Yes	No
16/2/2023	38+5		Yes	No
29/3/2023	32+6		No	No
17/6/2023	21+3		Yes	No
20/5/2023	25+4		No	No
1/7/2023	19+4		Yes	No
16/7/2023	17+3		Yes	No
23/6/2023	20+5		Yes	Yes
7/6/2023	23 weeks		Yes	No
27/5/2023	24+4		Yes	No
8/3/2023	36+0		Yes	No
19/3/2023	34+3		Yes	No
1/3/2023	37weeks		No	No
16/3/2023	34+6		No	No
13/4/2023	30+6		No	Yes
19/4/2023		30	Yes	No
9/4/2023	31+3		Yes	No
17/6/2023	21+5		No	No
16/6/2023	21+6		No	No
30/4/2023	28+4		No	No
10/5/2023	27+1		Yes	No
3/5/2023	28+1		Yes	No
11/4/2023	31+2		Yes	No
6/3/2023	36+3		No	No

13/4/2023	31 weeks + 0	No	No
9/5/2023	27+2	Yes	No
13/3/2023	35+3	Yes	No
28/6/2023	20+2	Yes	No
25/3/2023	33+6	No	No
14/3/2023	35+6	No	Yes
8/4/2023	32+2	Yes	No
28/2/2023	37+6	No	No
2/4/2023	33+1	No	Yes
31/5/2023	24+5	Yes	Yes
5/3/2023	37+1	Yes	No
28/6/2023	20+5	Yes	No
13/5/2023	27+2	No	No
6/3/2023	37+0	No	No
21/6/2023	21+5	No	No
10/6/2023	23+2	No	No
4/3/2023	37+2	No	No
15/4/2023	31+2	No	No
27/7/2023	16+4	No	No
8/5/2023	28+0	Yes	No
31/5/2023	24+6	Yes	No
3/4/2023	33+1 day	No	Yes
7/5/2023	28+2	Yes	Yes
26/4/2023	29+4 weeks	Yes	No
17/2/2023	39+4	Yes	No
19/5/2023	26+5	Yes	No
27/5/2023	25+4	No	Yes
13/7/2023	18+6	No	No
20/6/2023	22+1	No	Yes
6/6/2023	24+1	Yes	No
13/4/2023	31+6	No	No
15/7/2023	18+4	Yes	No
21/4/2023	30+5	Yes	No

	26/6/2023	21+2	No	No
	1/5/2023	29+2	No	No
	3/5/2023	29 week+1day	Yes	No
	1/8/2023	16 weeks + 2 days	No	No
	5/5/2023	28 weeks + 6 days	No	No
	28/4/2023	29+6	No	No
	15/4/2023	31 weeks + 5 days	Yes	Yes
	15/7/2023	18 weeks + 5 days	No	No
	16/4/2023	31 weeks + 4 days	No	No
	7/6/2023	24 weeks + 1 days	No	No
	25/2/2023	38 weeks + 5 days	No	No
	17/6/2023	22 weeks+ 5 days	Yes	No
	11/5/2023	28	Yes	No
	23/3/2023	35+4	No	No
	13/3/2023	37+0	No	No
	23/4/2023	31+1	No	No
	8/5/2023	29	No	No
	27/5/2023	26+2	No	No
	26/3/2023	35+1	No	No
	22/4/2023	31+2	Yes	No
	1/4/2023	34+1	Yes	No
	21/7/2023	18+3	Yes	No
	13/6/2023	23+6	Yes	No
leave/temporarily disabled/st	28/6/2023	21+5	No	No
	4/3/2023	38+2	Yes	No
	24/4/2023	31	Yes	Yes
	12/5/2023	28+3	Yes	No
	7/6/2023	24+6	No	No
	19/4/2023	31+6	Yes	No
	20/4/2023	31+5	Yes	No
	9/4/2023	33+2	No	No
	20/4/2023	31+5	No	No
	21/4/2023	31+4	Yes	No

24/2/2023	39+4weeks		Yes	Yes
18/5/2023	27+5		Yes	No
3/3/2023	38+4		No	No
12/5/2023	28+4		Yes	No
8/6/2023	24+5		Yes	No
12/3/2023	37+2		Yes	No
29/3/2023		35	Yes	Yes
28/3/2023	35+1		No	No
29/4/2023	30+4		Yes	No
11/5/2023	28+6		No	No
3/5/2023		30	Yes	Yes
6/5/2023	29+4		Yes	Yes
30/6/2023	21+5		No	No
16/5/2023	28+1		No	No
25/6/2023	22+3		Yes	Yes
11/3/2023	37+4		No	No
10/3/2023	37+5		No	No
21/7/2023	18+5		No	No
22/4/2023	31+4		No	Yes
7/3/2023	38+1		No	No
18/6/2023	23+3		No	No
20/3/2023	36+3		No	No
13/5/2023	28+5		Yes	No
27/6/2023	22+2		Yes	No
17/4/2023	32+3		Yes	No
15/3/2023	37+1		Yes	No
8/4/2023	33+5		No	No
21/4/2023	31+6		No	No
20/4/2023		32	Yes	No
24/3/2023	35+6		Yes	No
1/8/2023	17+3		Yes	No
13/5/2023	28+6		Yes	No
2/6/2023	26+3		No	No

	18/3/2023	37+2		No	No
	9/4/2023	34+1		Yes	No
	2/4/2023	35+1		Yes	No
	24/6/2023	23+2		Yes	No
	12/6/2023		25	Yes	No
	24/4/2023	32+1		Yes	Yes
	27/5/2023	27+3		No	Yes
	25/3/2023	36+3		No	No
	29/6/2023	22+5		No	Yes
	22/4/2023	32+3		Yes	No
	1/4/2023	35+3		No	No
	14/6/2023	24+6		Yes	Yes
	19/7/2023	19+6		Yes	No
	12/5/2023	29+4		No	No
	7/3/2023	39+0		No	No
	27/7/2023	18+5		No	No
	10/5/2023	29+6		No	No
	29/3/2023	35+6		No	No
	24/5/2023	27+6		No	No
	5/4/2023	34+6		No	No
	25/5/2023	27+6		Yes	No
	6/5/2023	30+4		Yes	No
	3/6/2023	26+4		No	No
	29/6/2023	22+6		No	Yes
	19/4/2023	33 weeks		Yes	No
	13/3/2023	38+2		No	No
	26/3/2023	36+3		No	No
	2/7/2023	22+3		No	No
/ leave/temporarily disabled/st	31/3/2023	35+5		Yes	No
	17/6/2023	24+4		No	Yes
	19/3/2023	37+3		Yes	No
	11/4/2023	34+1		No	No
	9/7/2023	21+3		Yes	Yes

	9/5/2023	30+1		Yes	No
	16/3/2023	38weeks		No	No
	15/3/2023	38+1		No	Yes
	14/5/2023	29+4		No	Yes
	30/3/2023	36 weeks		Yes	No
	6/4/2023	35 WEEKS		No	Yes
	3/4/2023	35+3		Yes	No
	17/3/2023	37+6		No	No
	25/6/2023	23+4		No	No
	8/6/2023		26	No	No
	2/4/2023	35+4		No	No
	29/3/2023	36+1		No	No
	17/4/2023	33+3		No	No
	14/5/2023	29+4		Yes	No
	13/5/2023	29+5		Yes	No
	26/5/2023	27+6		No	No
	23/5/2023	28+2		No	No
leave/temporarily disabled/st	23/8/2023	15+2		Yes	No
	10/6/2023	25+6		Yes	No
	30/3/2023	36+1		No	No
	30/7/2023	19+1		Yes	No
	18/8/2023	16+2		No	No
	24/7/2023	20 weeks		No	No
	12/5/2023	30+3		Yes	No
	4/5/2023	31+4		No	No
	13/5/2023	30+2		No	No
	12/5/2023	30+3		No	No
	7/4/2023	35+3		Yes	Yes
	28/5/2023	28+1		No	No
tuition	15/5/2023		30	Yes	No
	2/4/2023	36+1		Yes	No
	13/4/2023	34+5		Yes	No
	8/4/2023	35+3		No	No

	26/6/2023	24+1	No	No
	29/6/2023	23+5	No	No
	8/7/2023	22+3	Yes	No
	26/8/2023	15+3	Yes	No
	6/8/2023	18+2	Yes	No
	22/8/2023	16 weeks	No	No
	1/7/2023	23+3	Yes	No
	14/5/2023	30+3	Yes	Yes
	24/4/2023	33+2	No	No
	11/8/2023	17+5	No	No
	21/5/2023	29+3	No	No
	28/8/2023	15+2	Yes	Yes
	12/6/2023	26+2	Yes	No
y leave/temporarily disabled/st	27/5/2023	28+4	Yes	No
	21/7/2023	20+6	No	No
	17/7/2023	21+3	Yes	No
	29/5/2023	28+3	No	No
	5/5/2023	31+6	Yes	No
	1/8/2023	19+2	Yes	No
	29/3/2023	37+1	No	No
	28/4/2023	32+6	No	No
	26/6/2023	24+3	No	No
	17/5/2023	30+1	Yes	No
	18/8/2023	16+6	No	No
	12/6/2023	26+4	No	No
	11/4/2023	35+3	No	No
	23/4/2023	33+5	Yes	No
	20/6/2023	25+6	Yes	No
	1/8/2023	19+6	Yes	No
	30/8/2023	15+5	No	No
	1/5/2023	33+0	No	No
	2/5/2023	32+6	Yes	No
	19/8/2023	17+2	Yes	Yes

	10/8/2023	18+4		No	No
	2/9/2023	15+2		Yes	No
	29/4/2023	33+3		Yes	No
	26/6/2023	25+1		No	No
	12/7/2023	22+6		No	No
	29/6/2023	24+5		No	No
	20/5/2023	30+3		Yes	No
	19/4/2023	34+6		No	Yes
	24/7/2023	21+1		No	No
	13/7/2023	22+5		Yes	No
	19/7/2023	21+6		No	Yes
	8/7/2023	23+3		No	No
	4/6/2023	28+2		No	No
	6/6/2023	28+0		No	No
	1/6/2023	28+6		Yes	Yes
	1/4/2023	37+4		No	No
	18/4/2023	35+6		Yes	No
	8/8/2023	19+6		Yes	No
	31/8/2023	16+4		No	No
	26/6/2023		26	No	No
	30/7/2023	21+2		No	No
	6/5/2023	33+2		No	Yes
	8/7/2023	24+2		Yes	No
	16/7/2023	23+1		Yes	No
	29/7/2023	21+2		Yes	No
	14/4/2023	36+3		No	No
y leave/temporarily disabled/st	11/6/2023	28+1		No	No
	11/9/2023	15+1		No	No
	22/6/2023	26+5		No	No
	28/5/2023	32+5		No	Yes
	26/4/2023	34+6		Yes	No
	26/7/2023	21+6		Yes	No
	23/7/2023	22+2		No	No

11/6/2023	28+2		Yes	No
19/4/2023	35+6		No	No
4/4/2023	38 weeks		Yes	No
16/5/2023	32+1		No	No
16/6/2023	27+5		Yes	No
9/5/2023	33+1		No	No
17/4/2023	36+2		Yes	Yes
10/5/2023	33+0		No	No
29/6/2023	25+6		No	Yes
26/7/2023	22+0		Yes	No
17/7/2023	23+2		Yes	No
16/7/2023	23+3		No	No
22/4/2023	35+4		Yes	No
31/5/2023		30	No	No
19/6/2023	27+2		No	No
18/5/2023	31+6		Yes	Yes
30/6/2023	25+6		Yes	No
3/9/2023	16+4		No	Yes
10/6/2023	28+5		Yes	Yes
27/4/2023	35 weeks		Yes	Yes
11/7/2023	24+2		No	No
11/7/2023	24+2		Yes	No
17/6/2023	27+5		No	No
21/6/2023	27+1		No	No
7/7/2023	24+6		Yes	No
4/4/2023	38+2		No	No
18/5/2023	23+0		No	No
13/5/2023	32+3		Yes	No
11/5/2023	33+0		No	No
15/4/2023	36+5		No	No
20/7/2023		23	No	No
14/5/2023	33+1		Yes	No
3/6/2023	30+2		No	No

12/5/2023	33+3	No	No
30/5/2023	30+6	No	Yes
5/5/2023	34+4	No	No
29/6/2023	26+5	Yes	No
19/5/2023	32+4	Yes	No
5/7/2023	25+6	No	No
5/6/2023	30+1	No	No
11/6/2023	29+2	No	No
30/7/2023	22+2	No	No
17/4/2023	37+1	Yes	No
12/6/2023	29+1	No	No
30/4/2023	35+2	Yes	No
1/5/2023	35+2	No	No
12/7/2023	25+0	No	Yes
22/5/2023	32+2	Yes	No
31/7/2023	22+2	Yes	No
22/4/2023	36+4	No	Yes
2/7/2023	26+3	No	No
31/7/2023	22+2	No	Yes
9/7/2023	25+3	Yes	No
21/5/2023	28+6	Yes	No
14/4/2023	37+6	No	Yes
15/7/2023	24+5	No	No
30/7/2023	23+4	Yes	Yes
25/5/2023	32 weeks.	No	No
21/8/2023	19+3	Yes	No
20/6/2023	28+2	Yes	No
16/7/2023	24+4	Yes	No
17/5/2023	33+1	No	No
31/5/2023	31+1	No	Yes
20/6/2023	28+2	No	No
26/6/2023	27+4	Yes	No
30/6/2023	27+3	Yes	No

4/6/2023	31+1	Yes	No
9/6/2023	30+3	Yes	No
30/5/2023	31+6	No	Yes
24/6/2023	28+2	No	No
29/5/2023	32+0	No	No
27/7/2023	23+4	Yes	No
7/9/2023	17+5	No	No
8/6/2023	30+5	Yes	No
12/6/2023	30+1	Yes	No
24/5/2023	32+6	Yes	Yes
19/6/2023	29+1	No	No
14/6/2023	29+6	Yes	No
22/6/2023	28+5	Yes	No
24/9/2023	15+2	Yes	No
25/6/2023	28+3	No	No
10/7/2023	26+2	No	Yes
27/6/2023	28+1	No	No
10/7/2023	26+2	Yes	No
17/6/2023	29+4	No	No
28/6/2023	28+0	No	No
16/9/2023	16+4	Yes	No
1/5/2023	36+2	No	No
12/6/2023	30+3	No	No
25/7/2023	24+2	Yes	No
8/5/2023	35+3	No	No
15/4/2023	38+5	No	No
13/8/2023	21+4	Yes	No
25/7/2023	24+2	Yes	No
10/6/2023	30+5	No	No
2/6/2023	31+6	Yes	Yes
14/4/2023	38+6	Yes	No
26/6/2023	28+3	Yes	No
24/5/2023	33+1	Yes	No

	21/7/2023	24+6		Yes	No
	29/5/2023	32+3		No	No
	25/5/2023	33+1		No	No
	14/6/2023	30+2		Yes	No
	5/5/2023		36	Yes	No
	31/7/2023	24+0		Yes	No
	19/6/2023	30 weeks.		No	No
	14/6/2023	30+5		Yes	No
/ leave/temporarily disabled/st	10/5/2023	35+5		No	No
	6/7/2023	27+4		Yes	No
	17/7/2023		26	Yes	No
	28/7/2023	24+4		No	No
	13/5/2023	35+3		No	No
	7/8/2023	23+1		Yes	No
	16/7/2023	26+2		No	Yes
	14/9/2023	17+5		No	No
	15/6/2023	30+5		Yes	No
	17/8/2023	21+5		No	No
	18/8/2023	21+4		No	No
	8/6/2023	31+5		Yes	No
	16/7/2023	26+2		No	No
	1/9/2023	19+4		No	Yes
	5/5/2023	36+4		No	No
	29/5/2023	33+1weeks		No	No
	5/6/2023	32+2		No	No
	30/5/2023	33+1		No	No
	30/5/2023	33+1		No	No
	27/8/2023	20+4		No	Yes
	5/6/2023	36+4		No	Yes
	7/8/2023	23+2		Yes	No
	30/7/2023	24+3		No	No
	7/9/2023	18+6		No	No
	11/9/2023	18+2		Yes	No

	28/6/2023	29	Yes	No
	25/7/2023	25+1	No	No
	2/6/2023	32+5	Yes	No
	11/5/2023	35+6	No	Yes
	25/8/2023	20+5	Yes	No
	4/5/2023	37 weeks	No	No
	9/7/2023	27+4	Yes	No
	20/7/2023	26	No	No
	27/7/2023	25weeks	No	Yes
	2/8/2023	24+1	Yes	No
	10/8/2023	23	No	No
	14/8/2023	22+3	No	No
	20/5/2023	34+5	No	Yes
y leave/temporarily disabled/st	16/6/2023	30+6	Yes	No
	21/5/2023	34+4	No	No
	20/5/2023	34+5	No	No
	26/9/2023	16+3	Yes	No
	2/6/2023	33 weeks	No	No
	16/6/2023	31+0	Yes	No
	4/10/2023	15+5	No	No
	15/9/2023	18+3	Yes	Yes
	1/7/2023	29+2	No	No
	1/5/2023	38 weeks.	Yes	No
	21/5/2023	35+1	No	Yes
	22/7/2023	26+3	Yes	No
	16/6/2023	31+4	Yes	Yes
	2/9/2023	20+3	No	No
	16/6/2023	31+4	Yes	No
	11/6/2023	32+2	No	No
	20/6/2023	31 weeks.	Yes	No
	13/8/2023	23+3	Yes	No
	29/6/2023	29+6	No	Yes
	23/8/2023	22+0	Yes	Yes

19/9/2023	18+1	No	No
29/9/2023	16+5	Yes	Yes
17/8/2023	22+6	No	No
1/7/2023	29+4	No	No
26/5/2023	34+5	No	No
16/8/2023	23 weeks.	No	Yes
11/6/2023	34+5	Yes	No
1/9/2023	20+6	No	No
10/7/2023	28+3	Yes	Yes
24/8/2023	22 weeks.	Yes	No
20/9/2023	18+1	Yes	Yes
30/5/2023	34+2	Yes	No
20/7/2023	27+5	Yes	No
28/5/2023	35+2	No	No
2/10/2023	17+2	No	No
17/7/2023	28+2	Yes	No
22/6/2023	31+6	No	No
20/6/2023	32+1	No	No
17/5/2023	37+1	Yes	No
11/7/2023	29	Yes	No
1/7/2023	30+5	No	No
1/7/2023	30+5	No	No
30/7/2023	28+1	No	Yes
5/10/2023	18+4	Yes	No
16/6/2023	34+3	Yes	No
5/10/2023	18+4 weeks	No	No
15/10/2023	17+1 weeks	No	No
8/8/2023	26+6 WEEKS	No	No
7/7/2023	31+3 WEEKS	I don't know	No
27/7/2023	28+5	No	No
24/7/2023	29+1 WEEKS	Yes	No
25/6/2023	33+2	Yes	No
19/8/2023	25+3 WEEKS	Yes	No

25/8/2023	24+4 WEEKS	No	No
28/7/2023	28+4	Yes	No
9/10/2023	18+1	Yes	No
20/10/2023	16+4	No	No
19/8/2023	25+4 WEEKS	No	No
7/6/2023	36weeks.	Yes	No
3/7/2023	32+2 WEEKS	No	No
18/6/2023	34+3	Yes	No
3/6/2023	36+4	Yes	No
23/10/2023	16+2 WEEKS	Yes	No
27/7/2023	29 weeks.	No	No
5/10/2023	19 weeks.	No	Yes
12/8/2023	26+5 weeks	No	No
8/6/2023	36 weeks.	No	No
7/8/2023	27+3 WEEKS	No	Yes
6/9/2023	23+1	No	No
13/8/2023	26+4	Yes	No
14/8/2023	26+3 WEEKS	Yes	Yes
16/10/2023	17+3	Yes	No
5/10/2023	19+4 WEEKS	No	No
30/6/2023	33+3	No	Yes
3/10/2023	19+6	Yes	Yes
23/8/2023	25+5 WEEKS	Yes	No
26/10/2023	16+4	No	Yes
28/6/2023	33+5 WEEKS	No	No
23/6/2023	34+4	Yes	No
28/7/2023	29+3 WEEKS	Yes	No
23/6/2023	34+4 WEEKS	Yes	No
23/10/2023	17+1 weeks.	Yes	Yes
25/5/2023	38+5 WEEKS	No	No
21/9/2023	21+5	Yes	No
23/6/2023	35 weeks	No	No
11/8/2023	27+5 WEEKS	Yes	No

14/7/2023	31+5 WEEKS.	Yes	No
2/9/2023	24+4 WEEKS	No	No
16/7/2023	31+3	Yes	No
1/8/2023	29+1 weeks	Yes	No
12/10/2023	18+6 weeks	Yes	No
14/8/2023	27+2	No	No
11/10/2023	19 WEEKS	Yes	No
2/8/2023	29 WEEKS	Yes	No
1/8/2023	29+2 WEEKS	Yes	No
1/11/2023	16+1	No	No
28/6/2023	34+1 WEEKS	Yes	Yes
9/7/2023	32+4	Yes	No
9/10/2023	19+3 WEEKS	Yes	Yes
19/7/2023	31+1 WEEKS	Yes	No
23/10/2023	17+3	No	No
9/10/2023	19+3 WEEKS	Yes	No
10/6/2023	37+6	Yes	No
7/9/2023	24 weeks	Yes	Yes
4/8/2023	28+6	No	No
29/10/2023	16+4 WEEKS	Yes	No
18/9/2023	22+4 WEEKS.	No	No
16/6/2023	36+3 WEEKS	No	No
28/10/2023	17+2	Yes	No
24/6/2023	35+2 WEEKS	No	No
2/11/2023	16+4	Yes	No
9/9/2023	24+2	Yes	No
5/8/2023	29+2	No	No
29/9/2023	21+3 WEEKS	No	No
1/8/2023	29+6 WEEKS	Yes	No
20/8/2023	27+1 WEEKS	Yes	No
28/10/2023	17+2	Yes	No
29/10/2023	17+1 weeks	Yes	No
24/6/2023	35+3 weeks	Yes	No

29/9/2023	21+4	I don't know	Yes
24/6/2023	35+3 WEEKS	Yes	No
15/7/2023	32+3	Yes	No
26/10/2023	17+5	No	No
12/7/2023	32+6 WEEKS	Yes	No
10/6/2023	37+3 WEEKS	Yes	No
3/10/2023	21 WEEKS.	Yes	Yes
8/9/2023	24+4	Yes	No
22/10/2023	18+2 WEEKS	No	Yes
3/8/2023	29+5 WEEKS	Yes	No
19/9/2023	23 weeks.	Yes	Yes
11/9/2023	24+1	Yes	No
8/11/2023	15+6 weeks	No	No
15/10/2023	19+3 WEEKS	No	No
22/6/2023	35+6	Yes	No
12/7/2023	33 weeks.	No	No
12/10/2023	19+6 WEEKS	Yes	No
22/8/2023	27+ 1 WEEKS.	No	No
5/9/2023	25+1 WEEKS	No	No
15/8/2023	28+1 weeks	Yes	No
24/9/2023	22+4 WEEKS	Yes	No
1/10/2023	21+4	Yes	No
11/7/2023	33+2 WEEKS	Yes	No
29/10/2023	17+4	Yes	No
28/7/2023	30+6 WEEKS.	No	No
4/10/2023	21+1	No	Yes
17/7/2023	32+3 WEEKS	No	No
4/7/2023	34+2	Yes	No
5/8/2023	29+5	No	No
28/8/2023	27 WEEKS.	Yes	No
26/8/2023	26+6 weeks	Yes	No
1/9/2023	26+3 WEEKS	Yes	Yes
2/10/2023	22 WEEKS	No	No

30/10/2023	18 weeks.	Yes	No
28/10/2023	18+2 WEEKS.	No	No
16/8/2023	28+5WEEKS	No	No
5/9/2023	25+6	No	No
30/6/2023	35+3 WEEKS	No	No
2/8/2023	30+5 WEEKS.	No	Yes
22/8/2023	27+6 WEEKS	No	No
6/9/2023	25+5 WEEKS	No	No
15/11/2023	15+5	No	No
30/6/2023	35+4	No	No
23/10/2023	19+1 WEEKS	Yes	No
8/7/2023	34+3 WEEKS.	No	Yes
23/8/2023	27+6 WEEKS	Yes	No
23/7/2023	32+2	No	No
22/7/2023	32+3 WEEKS	No	No
12/6/2023	38+1 WEEKS	No	No
14/10/2023	20+3	Yes	No
29/9/2023	22+6	No	Yes
27/7/2023	31+5 WEEKS	No	No
25/8/2023	27+4	No	No
25/9/2023	23+1 WEEKS	Yes	No
24/8/2023	27+5	Yes	Yes
22/7/2023	32+4	Yes	No
19/7/2023	33 WEEKS	Yes	No
22/11/2023	15 WEEKS	Yes	Yes
9/11/2023	16+6	Yes	Yes
1/10/2023	22+3	No	Yes
18/7/2023	33+1 WEEKS	No	No
17/7/2023	33+2	Yes	No
14/8/2023	29+2 WEEKS	Yes	No
9/7/2023	34+4	Yes	No
20/11/2023	15+3 WEEKS	Yes	No
16/10/2023	20+3	Yes	No

	18/10/2023	20+1 WEEKS	Yes	No
	23/7/2023	32+4	No	No
	12/11/2023	16+4 WEEKS	Yes	No
	25/9/2023	23+3	No	No
	28/7/2023	31+6 WEEKS	Yes	No
	4/11/2023	17+5	Yes	No
	29/9/2023	23+3	Yes	Yes
	6/7/2023	35 WEEKS	No	No
	15/8/2023	29+2 WEEKS.	Yes	No
	7/8/2023	31+1	No	Yes
	10/9/2023	26+2	Yes	No
	8/9/2023	26+4 weeks	Yes	No
	9/7/2023	35+2	Yes	No
	25/9/2023	24+1 weeks	Yes	No
	5/11/2023	18+2	Yes	No
	11/10/2023	21+6	No	No
	24/10/2023	20 weeks	No	No
	11/8/2023	30+4 weeks	Yes	No
	25/11/2023	15+4	Yes	Yes
	30/9/2023	23+4 weeks	No	No
	29/6/2023	36+6 WEEKS	No	No
	29/7/2023	32+4 WEEKS.	No	No
	25/9/2023	24+2 WEEKS.	Yes	Yes
	1/11/2023	19 weeks	Yes	No
	2/8/2023	32 WEEKS.	No	No
	16/7/2023	34+3 weeks.	Yes	Yes
	17/8/2023	29+6 weeks	No	No
	4/7/2023	36+1	No	No
	21/7/2023	33+6 WEEKS	Yes	No
	30/8/2023	28+1 WEEKS	Yes	No
	26/10/2023	20 WEEKS	No	No
y leave/temporarily disabled/st	30/6/2023	36+6	Yes	Yes
	3/10/2023	23+2 WEEKS	Yes	No

	21/9/2023	25 weeks.	Yes	No
	10/10/2023	22+2 WEEKS	No	No
	1/7/2023	36+5 WEEKS	No	No
	8/9/2023	26+6 weeks.	Yes	No
	1/7/2023	37+2 WEEKS	No	Yes
	14/7/2023	35+3 weeks	Yes	No
	30/8/2023	28+5 WEEKS	Yes	No
	3/9/2023	28+1 WEEKS	No	No
/ leave/temporarily disabled/st	20/7/2023	34+4 WEEKS	Yes	No
	13/8/2023	31+1 WEEKS	Yes	No
	20/7/2023	34+4 WEEKS	No	No
	24/9/2023	25+1 WEEKS	Yes	No
	15/8/2023	30+6 WEEKS	No	No
	17/7/2023	35 WEEKS	Yes	No
	22/10/2023	21+2 WEEKS	No	No
	9/7/2023	36+2 WEEKS	Yes	No
	25/9/2023	25+1 WEEKS	No	No
/ leave/temporarily disabled/st	8/10/2023	23+3 weeks	Yes	No
	6/10/2023	23+5 weeks	No	No
	4/8/2023	32+5 WEEKS	No	No
	29/9/2023	24+5 WEEKS	No	No
	13/7/2023	35+6 weeks	No	No
	28/6/2023	38 weeks	No	No
	27/10/2023	20+6 WEEKS	No	No
	23/11/2023	17 WEEKS	No	No
	13/7/2023	36 WEEKS	Yes	No
	20/11/2023	17+3 WEEKS	No	No
	3/11/2023	19+6 WEEKS	No	No
	7/11/2023	19+2 WEEKS	No	No
	23/8/2023	30+1 WEEKS	No	No
	17/7/2023	35+3 WEEKS	No	No
	14/7/2023	36 WEEKS	No	No
	28/9/2023	25+4 weeks	No	No

	14/8/2023	32 WEEKS	No	No
	6/12/2023	15+5 WEEKS	Yes	No
	11/8/2023	32+3 WEEKS	Yes	No
	10/11/2023	19+3 WEEKS	No	Yes
	17/11/2023	18+4 weeks	Yes	No
	29/6/2023	38+5 weeks	Yes	No
	24/7/2023	35+1 weeks	Yes	No
	13/8/2023	32+2 weeks	No	No
	31/7/2023	34+2 WEEKS	No	No
	24/8/2023	30+6 WEEKS	No	No
	8/9/2023	28+5 WEEKS	No	No
y leave/temporarily disabled/st	17/7/2023	36+2 WEEKS	Yes	No
	19/8/2023	31+4 WEEKS	Yes	No
	7/8/2023	33+3 WEEKS	No	No
	21/10/2023	22+5 WEEKS	Yes	No
	5/10/2023	25 WEEKS	No	No
	19/8/2023	32+2 WEEKS	No	No
	1/10/2023	26+1 WEEKS	Yes	No
	31/10/2023	21+6 WEEKS	Yes	No
	30/8/2023	30+5 WEEKS	No	No
	24/9/2023	27+1 WEEKS	Yes	No
	16/9/2023	28+3 weeks	No	No
	27/7/2023	35+5 WEEKS	Yes	No
	3/8/2023	34+5 WEEKS	Yes	No
	1/9/2023	30+4 WEEKS	Yes	No
	22/7/2023	36+3 WEEKS	No	No
	10/7/2023	38+1 WEEKS	I don't know	No
Business	22/8/2023	32+1 WEEKS	No	No
	1/8/2023	35+1 WEEKS	Yes	No
	31/8/2023	30+6 WEEKS	No	No
	3/8/2023	35+4 weeks	Yes	No
	26/7/2023	36+5 WEEKS	Yes	No
	25/10/2023	23+5 WEEKS	Yes	No

	21/9/2023	28+4 WEEKS	I don't know	No
	18/9/2023	29 WEEKS	No	No
	6/11/2023	22 WEEKS	No	No
	15/11/2023	20+6 weeks	No	No
	1/11/2023	22+6 WEEKS	Yes	No
	30/9/2023	27+3 WEEKS	Yes	No
	9/9/2023	30+3 WEEKS	Yes	No
	2/11/2023	22+5 weeks	No	No
	24/8/2023	32+5 WEEKS	No	No
	30/10/2023	23+2 WEEKS	Yes	No
	16/9/2023	29+4 WEEKS	No	No
	17/11/2023	20+5 WEEKS	No	Yes
	1/11/2023	23 WEEKS	No	No
	1/10/2023	27+3 WEEKS	No	Yes
	25/7/2023	37+1 weeks	No	No
	8/10/2023	26+3 weeks	No	No
	21/10/2023	24+5 weeks	No	No
	18/7/2023	38+2 WEEKS	Yes	No
	23/8/2023	33+1 WEEKS	Yes	No
	17/8/2023	34 WEEKS	No	No
	30/11/2023	19+4 WEEKS	Yes	No
	13/11/2023	22 WEEKS	No	No
	22/8/2023	33+6 WEEKS	Yes	No
	30/10/2023	24 WEEKS	No	No
	1/11/2023	23+5 WEEKS	No	No
	19/9/2023	30 weeks	No	No
	27/9/2023	29 WEEKS	No	No
	14/8/2023	35+2 WEEKS	Yes	No
	4/8/2023	36+5 WEEKS	Yes	No
	26/9/2023	29+1 WEEKS	Yes	Yes
y leave/temporarily disabled/st	8/9/2023	31+6 weeks	Yes	No
	17/12/2023	17+4 WEEKS	Yes	No
	25/11/2023	20+5 WEEKS	Yes	Yes

18/9/2023	30+4 WEEKS	No	Yes
8/10/2023	27+5 WEEKS	Yes	Yes
26/9/2023	29+3 WEEKS	No	No
21/10/2023	25+6 WEEKS	Yes	No
3/11/2023	24+4 WEEKS	No	No
25/11/2023	21+3 WEEKS	Yes	No
13/11/2023	23+1 WEEKS	Yes	Yes
30/11/2023	20+5 WEEKS	Yes	No
11/12/2023	19+1 WEEKS	No	No
25/12/2023	17+1 WEEKS	Yes	No
11/12/2023	19+2 WEEKS	No	No
19/11/2023	22+3 weeks	No	No
5/9/2023	33+1 weeks	No	Yes
12/9/2023	32+1 weeks	No	Yes
9/9/2023	32+4 weeks	No	No
17/8/2023	36+1 WEEKS	No	Yes
4/9/2023	33+4 WEEKS	Yes	No
5/11/2023	24+5 WEEKS	No	No
29/9/2023	30 WEEKS	No	No
12/9/2023	33 WEEKS	No	No
7/10/2023	29+3 WEEKS	No	No
25/10/2023	26+6 WEEKS	Yes	No
6/10/2023	29+5 WEEKS	Yes	No
15/10/2023	28+3 WEEKS	No	No
17/9/2023	32+3 WEEKS	Yes	No
26/8/2023	35+4 WEEKS	Yes	No
29/10/2023	26+3 WEEKS	No	No
18/10/2023	28 WEEKS	Yes	No
16/9/2023	32+4 WEEKS	Yes	No
3/10/2023	30+2 WEEKS	No	No
28/9/2023	31 WEEKS	Yes	No
28/8/2023	35+3 WEEKS	Yes	No
13/11/2023	25 WEEKS	Yes	Yes

	13/12/2023	20+5 WEEKS	No	No
	11/8/2023	38+3 WEEKS	Yes	No
	20/10/2023	28+4 WEEKS	Yes	No
	15/10/2023	29+3 weeks	No	Yes
	10/12/2023	21+2 WEEKS	Yes	No
	9/9/2023	34+4 weeks	No	No
	18/10/2023	29 WEEKS	No	No
	21/8/2023	37+2 WEEKS	No	No
y leave/temporarily disabled/st	30/12/2023	18+5 WEEKS	Yes	No
	7/9/2023	35 WEEKS	Yes	No
	12/11/2023	26+1 WEEKS	Yes	No
	28/1/2024	15+1 WEEKS	No	No
	10/9/2023	35+1 WEEKS	No	No
	21/10/2023	29+2 WEEKS	Yes	Yes
	1/10/2023	32+2 WEEKS	No	Yes
	21/12/2023	20+5 WEEKS	Yes	No
	19/12/2023	21 WEEKS	Yes	No
	8/12/2023	22+4 WEEKS	Yes	No
	1/10/2023	32+2 WEEKS	Yes	No
	3/9/2023	36+2 WEEKS	No	No
	3/1/2024	19 WEEKS	Yes	No
	13/10/2023	30+6 WEEKS	Yes	No
	9/12/2023	22+5 WEEKS	No	No
	18/1/2024	17 WEEKS	Yes	No
	14/10/2023	30+5 WEEKS	Yes	No
	15/1/2024	18 WEEKS	No	No
	11/1/2024	18+4 WEEKS	No	No
	9/10/2023	32+1 WEEKS	Yes	No
	14/12/2023	22+5 weeks	No	No
	7/9/2023	36+5 WEEKS	Yes	No
	7/11/2023	28+1 WEEKS	Yes	No
	27/10/2023	29+5 WEEKS	Yes	No
	23/9/2023	34+4 weeks	Yes	Yes

25/12/2023	21+2 WEEKS	Yes	No
28/9/2023	33+6 WEEKS	No	No
11/11/2023	27+4 WEEKS	Yes	No
26/9/2023	34+1 WEEKS	No	No
28/11/2023	25+2 WEEKS	Yes	Yes
30/9/2023	33+5 WEEKS	No	No
28/8/2023	38+3 WEEKS	No	No
13/11/2023	28 WEEKS	Yes	No
2/9/2023	38+2 WEEKS	Yes	No
19/9/2023	35+6 WEEKS	No	No
27/1/2024	17+3 WEEKS	I don't know	No
29/10/2023	30+2 WEEKS	No	No
19/12/2023	23 WEEKS	No	No
5/10/2023	33+5 WEEKS	No	No
10/11/2023	28+5 WEEKS	No	No
8/10/2023	33+3 WEEKS	Yes	No
27/12/2023	22+1 WEEKS	Yes	No
9/10/2023	33+3 WEEKS	No	No
18/9/2023	36+3 WEEKS	Yes	No
20/10/2023	31+6 WEEKS	No	No
30/10/2023	30+3 WEEKS	Yes	No
8/1/2024	21 WEEKS	No	No
30/9/2023	35+2 WEEKS	No	No
17/11/2023	28+3 WEEKS	No	No
20/9/2023	36+5 WEEKS	No	Yes
4/10/2023	34+6 WEEKS	I don't know	No
18/10/2023	32+6 WEEKS	No	No
27/9/2023	35+6 WEEKS	No	No
18/11/2023	28+4 WEEKS	No	Yes
22/11/2023	28 WEEKS	Yes	No
18/9/2023	37+2 WEEKS	No	No
26/11/2023	28+1 WEEKS	No	Yes
18/10/2023	33+5 WEEKS	No	No

	5/11/2023	31+4 WEEKS	Yes	No
y leave/temporarily disabled/st	24/10/2023	33+2 WEEKS	No	No
	17/11/2023	29+6 WEEKS	No	No
	21/12/2023	25+4 WEEKS	No	Yes
	15/11/2023	30+5 WEEKS	No	No
	9/10/2023	36 WEEKS	No	Yes
	13/11/2023	31 WEEKS	No	No
	4/11/2023	32+2 WEEKS	No	No
	17/10/2023	34+6 WEEKS	No	No
	9/10/2023	36+1 WEEKS	No	Yes
	25/12/2023	25+1 WEEKS	No	No
	19/1/2024	21+4 WEEKS	No	No
	4/10/2023	36+6 WEEKS	No	No
	24/11/2023	29+4 WEEKS	Yes	No
	30/11/2023	28+5 WEEKS	Yes	No
	12/11/2023	31+3 WEEKS	Yes	Yes
	9/1/2024	23+1 WEEKS	No	No
	20/2/2024	17+1 WEEKS	Yes	No
y leave/temporarily disabled/st	20/1/2024	21+4 WEEKS	No	No
	11/10/2023	36+1 WEEKS	No	No
	30/11/2023	29 WEEKS	No	No
	8/12/2023	27+6 WEEKS	Yes	No
	6/10/2023	36+6 WEEKS	Yes	No
	13/11/2023	31+3 WEEKS	No	No
	3/2/2024	20+2 WEEKS	Yes	No
	18/12/2023	27 WEEKS	Yes	No
	25/1/2024	21+4 WEEKS	Yes	No
	2/10/2023	38 WEEKS	No	Yes
	13/10/2023	36+3 WEEKS	Yes	No
Business	24/12/2023	26+2 weeks	Yes	Yes
	4/12/2023	29+1 weeks	Yes	No
	29/1/2024	21+3 WEEKS	No	No
	26/10/2023	35 WEEKS	No	No

	11/10/2023	37+1 WEEKS	No	No
	13/10/2023	37+3 WEEKS	Yes	No
Business	30/11/2023	30+4 WEEKS	Yes	No
	29/1/2024	22 WEEKS	Yes	No
	25/10/2023	36 WEEKS	No	No
	22/1/2024	23+2 WEEKS	Yes	No
	17/11/2023	32+5 WEEKS	No	No
	10/1/2024	27+5 weeks	Yes	No
	24/12/2023	30+1 WEEKS	No	No
	15/3/2024	18+3 WEEKS	Yes	No
	28/12/2023	29+4 WEEKS	No	No
	31/12/2023	29+1 WEEKS	Yes	No
	7/12/2023	32+4 WEEKS	No	No
	10/2/2024	23+2 WEEKS	Yes	No
	20/12/2023	31 WEEKS	No	No
	19/1/2024	26+5 WEEKS	Yes	No
	23/12/2023	30+4 WEEKS	No	No
	21/3/2024	17+6 WEEKS	No	No
	20/12/2023	31 WEEKS	No	No
	17/3/2024	18+4 WEEKS	No	No

Vaginal intercourse during pregnancy	Overall, how do you feel about taking your own vaginal s	Do you think that self-sample collection is easy to perforr
Yes	Neutral	Neutral
Yes	Good	Easy
Yes	Good	Very easy
Yes	Neutral	Hard
No	Good	Neutral
Yes	Neutral	Neutral
No	Good	Neutral
No	Good	Easy
Yes	Good	Easy
No	Good	Easy
Yes	Neutral	Hard
Yes	Neutral	Easy
Yes	Good	Easy
Yes	Very good	Easy
Yes	Good	Easy
Yes	Good	Easy
No	Good	Neutral
Yes	Neutral	Easy
Yes	Neutral	Neutral
No	Good	Easy
Yes	Neutral	Easy
Yes	Neutral	Easy
Yes	Good	Very easy
No	Good	Neutral
Yes	Neutral	Neutral
Yes	Good	Easy
Yes	Good	Easy
Yes	Good	Easy
Yes	Neutral	Easy
No	Good	Easy
Yes	Good	Easy
No	Neutral	Neutral

No	Neutral	Easy
No	Good	Easy
Yes	Good	Neutral
No	Neutral	Easy
Yes	Neutral	Easy
Yes	Good	Easy
Yes	Good	Easy
Yes	Neutral	Easy
Yes	Neutral	Neutral
Yes	Neutral	Neutral
Yes	Neutral	Easy
Yes	Neutral	Neutral
Yes	Good	Easy
Yes	Good	Easy
Yes	Neutral	Easy
Yes	Neutral	Neutral
No	Neutral	Neutral
Yes	Good	Easy
Yes	Neutral	Neutral
Yes	Good	Easy
Yes	Good	Easy
Yes	Very good	Easy
No	Good	Easy
Yes	Good	Very easy
Yes	Good	Easy
Yes	Neutral	Easy
Yes	Neutral	Easy
Yes	Bad	Hard
No	Very good	Very easy
Yes	Good	Easy
Yes	Very good	Very easy
No	Neutral	Easy
Yes	Good	Easy

Yes	Neutral	Easy
Yes	Good	Easy
Yes	Neutral	Neutral
No	Neutral	Easy
Yes	Neutral	Easy
Yes	Good	Easy
Yes	Good	Easy
No	Neutral	Easy
Yes	Good	Very easy
Yes	Neutral	Easy
No	Good	Easy
Yes	Neutral	Neutral
Yes	Neutral	Neutral
Yes	Good	Neutral
Yes	Very good	Neutral
Yes	Good	Hard
Yes	Good	Easy
Yes	Neutral	Easy
Yes	Good	Easy
Yes	Good	Neutral
Yes	Neutral	Neutral
Yes	Bad	Hard
Yes	Very good	Very easy
Yes	Very good	Very easy
Yes	Neutral	Neutral
Yes	Neutral	Easy
Yes	Good	Easy
yes	Good	Easy
Yes	Neutral	Easy
Yes	Very good	Easy
No	Very good	Easy
Yes	Bad	Neutral
Yes	Good	Easy

No	Good	Neutral
Yes	Neutral	Easy
Yes	Very good	Easy
Yes	Neutral	Easy
Yes	Neutral	Neutral
Yes	Neutral	Easy
Yes	Neutral	Easy
Yes	Neutral	Neutral
No	Neutral	Easy
Yes	Neutral	Easy
Yes	Good	Easy
Yes	Good	Neutral
Yes	Bad	Easy
Yes	Neutral	Easy
No	Neutral	Easy
Yes	Bad	Hard
No	Good	Easy
Yes	Bad	Hard
Yes	Neutral	Easy
No	Good	Hard
Yes	Neutral	Neutral
Yes	Good	Easy
Yes	Neutral	Easy
Yes	Neutral	Easy
Yes	Bad	Neutral
Yes	Neutral	Neutral
Yes	Neutral	Neutral
Yes	Bad	Neutral
Yes	Neutral	Easy
Yes	Very good	Easy
No	Good	Easy
No	Neutral	Easy
Yes	Good	Easy

Yes	Neutral	Easy
Yes	Neutral	Easy
Yes	Good	Easy
Yes	Neutral	Easy
Yes	Very good	Easy
Yes	Neutral	Easy
Yes	Neutral	Hard
No	Neutral	Neutral
Yes	Neutral	Easy
No	Neutral	Neutral
Yes	Bad	Hard
Yes	Good	Easy
Yes	Good	Hard
Yes	Very good	Easy
Yes	Neutral	Easy
Yes	Neutral	Easy
Yes	Neutral	Neutral
No	Neutral	Neutral
Yes	Good	Easy
Yes	Good	Neutral
No	Bad	Hard
Yes	Bad	Neutral
Yes	Bad	Hard
Yes	Good	Easy
Yes	Neutral	Easy
Yes	Neutral	Easy
Yes	Neutral	Hard
Yes	Neutral	Neutral
No	Good	Easy
Yes	Neutral	Neutral
Yes	Neutral	Easy
No	Neutral	Neutral
Yes	Bad	Easy

Yes	Neutral	Easy
Yes	Very good	Easy
Yes	Neutral	Easy
Yes	Good	Easy
Yes	Good	Neutral
Yes	Good	Neutral
Yes	Neutral	Easy
Yes	Good	Very easy
Yes	Neutral	Neutral
Yes	Good	Neutral
Yes	Bad	Hard
Yes	Neutral	Neutral
No	Good	Easy
Yes	Neutral	Neutral
Yes	Neutral	Easy
No	Neutral	Easy
No	Neutral	Easy
Yes	Very good	Neutral
Yes	Neutral	Easy
Yes	Good	Easy
Yes	Bad	Hard
Yes	Neutral	Neutral
No	Good	Easy
Yes	Neutral	Easy
Yes	Good	Easy
Yes	Neutral	Neutral
Yes	Good	Easy
Yes	Neutral	Neutral
Yes	Good	Easy
Yes	Good	Neutral
Yes	Good	Easy
Yes	Neutral	Neutral
Yes	Good	Easy

Yes	Neutral	Easy
Yes	Good	Easy
Yes	Good	Easy
Yes	Good	Easy
Yes	Good	Easy
Yes	Neutral	Easy
Yes	Neutral	Hard
Yes	Good	Easy
Yes	Neutral	Neutral
Yes	Neutral	Hard
Yes	Neutral	Easy
No	Neutral	Easy
No	Good	Easy
Yes	Good	Easy
Yes	Very good	Easy
Yes	Neutral	Easy
No	Good	Neutral
Yes	Good	Hard
Yes	Good	Neutral
Yes	Neutral	Neutral
Yes	Neutral	Neutral
Yes	Neutral	Hard
Yes	Neutral	Easy
Yes	Neutral	Hard
Yes	Good	Neutral
Yes	Neutral	Easy
Yes	Neutral	Easy
Yes	Neutral	Easy
Yes	Neutral	Easy
Yes	Very good	Easy
Yes	Good	Easy
Yes	Neutral	Easy
Yes	Bad	Neutral

No	Bad	Easy
Yes	Good	Easy
Yes	Neutral	Neutral
Yes	Neutral	Easy
Yes	Neutral	Easy
Yes	Bad	Hard
Yes	Neutral	Hard
Yes	Neutral	Hard
No	Very good	Easy
Yes	Good	Easy
Yes	Good	Neutral
Yes	Good	Easy
Yes	Good	Easy
Yes	Good	Easy
Yes	Very good	Easy
Yes	Good	Neutral
Yes	Good	Easy
Yes	Good	Easy
Yes	Neutral	Easy
No	Good	Neutral
Yes	Neutral	Easy
Yes	Neutral	Hard
Yes	Very good	Easy
Yes	Good	Easy
Yes	Neutral	Easy
Yes	Good	Very easy
Yes	Neutral	Easy
Yes	Good	Easy
Yes	Good	Easy
Yes	Good	Easy
Yes	Neutral	Easy
Yes	Neutral	Neutral
Yes	Very good	Easy

Yes	Good	Easy
Yes	Neutral	Neutral
Yes	Neutral	Easy
Yes	Good	Easy
No	Good	Easy
Yes	Neutral	Hard
Yes	Neutral	Neutral
Yes	Neutral	Easy
Yes	Good	Neutral
Yes	Neutral	Easy
No	Neutral	Neutral
Yes	Good	Neutral
Yes	Neutral	Easy
Yes	Good	Easy
Yes	Neutral	Neutral
Yes	Very bad	Neutral
Yes	Good	Neutral
Yes	Neutral	Easy
Yes	Good	Neutral
Yes	Neutral	Hard
Yes	Neutral	Easy
Yes	Neutral	Easy
Yes	Good	Easy
Yes	Neutral	Neutral
Yes	Neutral	Neutral
Yes	Good	Easy
Yes	Very good	Neutral
No	Neutral	Neutral
Yes	Neutral	Easy
Yes	Neutral	Neutral
Yes	Neutral	Easy
Yes	Neutral	Easy
Yes	Neutral	Easy

Yes	Neutral	Neutral
No	Neutral	Neutral
Yes	Very bad	Hard
Yes	Very good	Easy
No	Bad	Neutral
Yes	Good	Easy
Yes	Neutral	Neutral
Yes	Neutral	Easy
Yes	Good	Easy
No	Neutral	Easy
Yes	Neutral	Neutral
Yes	Bad	Hard
Yes	Good	Easy
Yes	Good	Easy
Yes	Neutral	Hard
Yes	Neutral	Easy
Yes	Good	Easy
Yes	Good	Easy
Yes	Neutral	Easy
Yes	Good	Neutral
Yes	Good	Easy
Yes	Neutral	Easy
Yes	Very good	Easy
Yes	Good	Neutral
Yes	Good	Easy
Yes	Neutral	Neutral
Yes	Bad	Very hard
Yes	Good	Easy
Yes	Very good	Easy
Yes	Good	Easy
No	Good	Neutral
Yes	Very good	Easy
No	Neutral	Neutral

Yes	Neutral	Hard
Yes	Neutral	Neutral
Yes	Bad	Hard
No	Very good	Neutral
Yes	Neutral	Easy
Yes	Neutral	Very easy
Yes	Good	Easy
Yes	Good	Easy
Yes	Very good	Easy
Yes	Neutral	Neutral
Yes	Bad	Hard
Yes	Neutral	Easy
Yes	Bad	Easy
Yes	Good	Easy
Yes	Neutral	Hard
Yes	Good	Neutral
Yes	Neutral	Easy
Yes	Neutral	Hard
Yes	Neutral	Easy
Yes	Very good	Easy
Yes	Neutral	Easy
Yes	Very good	Very easy
Yes	Very good	Easy
Yes	Good	Easy
Yes	Good	Hard
Yes	Bad	Hard
Yes	Neutral	Neutral
Yes	Good	Hard
Yes	Good	Easy
Yes	Bad	Neutral
Yes	Good	Neutral
Yes	Good	Easy
Yes	Good	Neutral

Yes	Neutral	Easy
Yes	Very good	Very easy
No	Good	Easy
Yes	Neutral	Easy
Yes	Good	Easy
Yes	Neutral	Easy
Yes	Good	Easy
Yes	Good	Neutral
No	Neutral	Easy
No	Neutral	Easy
Yes	Very bad	Very hard
Yes	Neutral	Hard
No	Neutral	Easy
Yes	Neutral	Easy
Yes	Good	Easy
Yes	Bad	Hard
Yes	Good	Easy
Yes	Neutral	Neutral
Yes	Very good	Easy
Yes	Good	Easy
No	Neutral	Neutral
Yes	Neutral	Easy
Yes	Good	Easy
Yes	Very good	Very easy
Yes	Neutral	Very easy
Yes	Good	Easy
Yes	Neutral	Neutral
Yes	Neutral	Hard
Yes	Neutral	Very easy
Yes	Neutral	Very hard
Yes	Good	Easy
Yes	Good	Easy
Yes	Neutral	Easy

No	Good	Easy
Yes	Neutral	Very easy
Yes	Good	Easy
Yes	Good	Easy
Yes	Very good	Very easy
Yes	Neutral	Easy
No	Very good	Very easy
No	Neutral	Easy
Yes	Good	Easy
Yes	Neutral	Neutral
No	Neutral	Easy
Yes	Neutral	Easy
No	Very good	Easy
Yes	Neutral	Hard
Yes	Good	Easy
Yes	Neutral	Neutral
Yes	Good	Easy
Yes	Very good	Easy
Yes	Neutral	Easy
Yes	Neutral	Easy
No	Neutral	Very easy
Yes	Neutral	Neutral
No	Good	Easy
Yes	Good	Neutral
Yes	Neutral	Hard
Yes	Bad	Hard
Yes	Neutral	Hard
No	Good	Easy
Yes	Good	Easy
Yes	Neutral	Neutral
Yes	Neutral	Easy
Yes	Neutral	Very easy
No	Neutral	Easy

Yes	Neutral	Very easy
Yes	Very good	Very easy
Yes	Good	Easy
Yes	Very good	Easy
Yes	Good	Easy
Yes	Neutral	Easy
Yes	Neutral	Easy
No	Bad	Neutral
Yes	Very good	Easy
Yes	Very good	Very easy
Yes	Neutral	Easy
Yes	Very good	Very easy
Yes	Very good	Very easy
Yes	Very good	Very easy
Yes	Neutral	Very easy
Yes	Very good	Very easy
No	Very good	Very easy
Yes	Bad	Neutral
Yes	Neutral	Neutral
Yes	Good	Easy
Yes	Neutral	Neutral
No	Good	Easy
Yes	Bad	Hard
Yes	Good	Easy
Yes	Neutral	Easy
Yes	Neutral	Easy
Yes	Neutral	Neutral
Yes	Good	Easy
No	Good	Very easy
Yes	Neutral	Neutral
Yes	Neutral	Very easy
Yes	Good	Easy
Yes	Good	Easy

No	Very good	Easy
Yes	Neutral	Easy
Yes	Good	Easy
Yes	Very good	Very easy
Yes	Neutral	Very easy
Yes	Bad	Easy
No	Neutral	Easy
Yes	Neutral	Easy
No	Neutral	Easy
Yes	Very good	Very easy
No	Neutral	Neutral
Yes	Good	Very easy
Yes	Good	Easy
Yes	Very good	Very easy
Yes	Good	Easy
Yes	Good	Easy
Yes	Neutral	Hard
Yes	Neutral	Easy
No	Neutral	Easy
Yes	Bad	Hard
Yes	Bad	Hard
Yes	Very good	Easy
No	Good	Easy
No	Neutral	Easy
No	Bad	Hard
Yes	Very good	Easy
Yes	Neutral	Easy
Yes	Good	Easy
Yes	Good	Easy
Yes	Neutral	Neutral
Yes	Good	Easy
Yes	Neutral	Easy
No	Good	Easy

Yes	Very good	Very easy
Yes	Good	Easy
Yes	Very good	Very easy
Yes	Good	Very easy
No	Good	Easy
No	Neutral	Easy
Yes	Very good	Very easy
Yes	Good	Easy
Yes	Good	Easy
Yes	Neutral	Easy
Yes	Neutral	Neutral
No	Good	Very easy
Yes	Good	Easy
Yes	Neutral	Hard
No	Neutral	Neutral
Yes	Bad	Neutral
Yes	Good	Easy
Yes	Very good	Very easy
Yes	Good	Easy
Yes	Good	Very easy
No	Good	Hard
Yes	Good	Very easy
No	Good	Easy
Yes	Good	Very easy
Yes	Neutral	Easy
Yes	Neutral	Easy
No	Very good	Very easy
Yes	Very good	Very easy
Yes	Neutral	Easy
No	Good	Neutral
Yes	Good	Very easy
Yes	Neutral	Easy
Yes	Neutral	Easy

No	Neutral	Easy
Yes	Neutral	Very easy
No	Very good	Very easy
Yes	Neutral	Easy
Yes	Very good	Very easy
Yes	Very good	Easy
Yes	Good	Easy
Yes	Neutral	Neutral
No	Good	Easy
No	Very good	Very easy
No	Neutral	Easy
No	Neutral	Very easy
Yes	Neutral	Easy
Yes	Very good	Very easy
No	Good	Easy
No	Very good	Very easy
No	Good	Easy
Yes	Very good	Very easy
No	Very good	Very easy
Yes	Good	Easy
No	Good	Easy
Yes	Neutral	Neutral
Yes	Very good	Very easy
Yes	Good	Easy
Yes	Good	Easy
Yes	Neutral	Easy
Yes	Good	Easy
Yes	Good	Easy
No	Neutral	Easy
Yes	Good	Very easy
Yes	Good	Easy
Yes	Neutral	Easy
Yes	Very good	Easy

Yes	Good	Very easy
Yes	Very good	Easy
Yes	Good	Easy
Yes	Good	Neutral
Yes	Good	Easy
Yes	Neutral	Very easy
Yes	Very good	Easy
Yes	Good	Very easy
No	Neutral	Easy
Yes	Good	Neutral
Yes	Good	Very easy
Yes	Good	Easy
Yes	Good	Easy
Yes	Good	Very easy
Yes	Good	Easy
Yes	Very bad	Very easy
Yes	Very good	Very easy
Yes	Good	Easy
Yes	Good	Easy
Yes	Good	Very hard
Yes	Very good	Very easy
No	Neutral	Easy
Yes	Very good	Very easy
Yes	Very good	Easy
No	Very good	Easy
No	Neutral	Easy
Yes	Very good	Easy
Yes	Good	Very easy
Yes	Very good	Very easy
Yes	Very good	Very easy
No	Very good	Very easy
Yes	Good	Very easy
Yes	Good	Neutral

Yes	Very good	Very easy
Yes	Very good	Easy
Yes	Good	Neutral
Yes	Good	Very easy
Yes	Very good	Very easy
Yes	Neutral	Easy
Yes	Good	Very easy
Yes	Neutral	Neutral
Yes	Very bad	Easy
Yes	Good	Very easy
Yes	Neutral	Easy
Yes	Neutral	Easy
Yes	Good	Neutral
Yes	Good	Very easy
No	Good	Easy
No	Neutral	Neutral
Yes	Neutral	Neutral
Yes	Good	Easy
Yes	Very good	Very easy
Yes	Good	Neutral
Yes	Very good	Very easy
Yes	Very good	Very easy
Yes	Very good	Easy
No	Good	Easy
Yes	Good	Easy
Yes	Good	Easy
Yes	Very good	Very easy
Yes	Good	Easy
Yes	Very good	Easy
No	Good	Easy
No	Very good	Very hard
Yes	Good	Easy
Yes	Good	Very easy

No	Good	Easy
Yes	Good	Easy
Yes	Good	Easy
Yes	Very good	Very easy
No	Good	Easy
Yes	Neutral	Easy
Yes	Very good	Very easy
Yes	Good	Very easy
No	Neutral	Easy
Yes	Good	Easy
Yes	Very good	Very easy
Yes	Very good	Easy
No	Very good	Easy
Yes	Neutral	Hard
No	Very good	Easy
No	Very good	Very easy
Yes	Good	Easy
No	Good	Very easy
Yes	Neutral	Easy
No	Good	Neutral
Yes	Good	Very easy
Yes	Good	Easy
Yes	Very good	Easy
No	Very good	Very easy
Yes	Very good	Very easy
Yes	Good	Very easy
Yes	Good	Easy
Yes	Good	Easy
Yes	Very good	Neutral
Yes	Good	Easy
Yes	Very good	Very easy
Yes	Good	Very easy
Yes	Good	Easy

Yes	Very good	Very easy
Yes	Good	Very easy
Yes	Very good	Very easy
Yes	Neutral	Easy
Yes	Good	Easy
Yes	Good	Easy
Yes	Very good	Very easy
No	Good	Easy
No	Neutral	Easy
Yes	Good	Easy
Yes	Good	Easy
Yes	Very good	Very easy
Yes	Good	Easy
Yes	Neutral	Easy
Yes	Very good	Very easy
Yes	Neutral	Neutral
No	Good	Easy
No	Very good	Very easy
No	Good	Easy
No	Very good	Very easy
No	Good	Easy
Yes	Very good	Very easy
Yes	Very good	Easy
Yes	Neutral	Easy
Yes	Good	Neutral
Yes	Very good	Easy
Yes	Very good	Easy
No	Neutral	Easy
Yes	Good	Easy
Yes	Good	Easy
Yes	Good	Easy
Yes	Very good	Very easy
Yes	Neutral	Neutral

Yes	Good	Neutral
Yes	Good	Easy
Yes	Very good	Very easy
Yes	Good	Easy
Yes	Very good	Very easy
Yes	Neutral	Neutral
No	Neutral	Hard
Yes	Very good	Very easy
Yes	Very good	Very easy
No	Good	Easy
Yes	Neutral	Easy
Yes	Good	Very easy
Yes	Very good	Very easy
Yes	Good	Very easy
Yes	Neutral	Very easy
Yes	Good	Neutral
No	Good	Easy
Yes	Good	Easy
Yes	Good	Easy
Yes	Very good	Easy
Yes	Neutral	Easy
Yes	Good	Very easy
Yes	Good	Easy
No	Good	Easy
No	Neutral	Easy
No	Very good	Very easy
No	Very good	Easy
No	Good	Hard
Yes	Good	Easy
Yes	Good	Easy
No	Neutral	Easy
No	Very good	Very easy
No	Neutral	Easy

Yes	Good	Neutral
Yes	Good	Easy
Yes	Neutral	Easy
Yes	Neutral	Neutral
Yes	Good	Easy
No	Good	Neutral
No	Very good	Hard
Yes	Neutral	Easy
No	Good	Easy
No	Very good	Easy
Yes	Very good	Very easy
Yes	Good	Easy
Yes	Very good	Very easy
No	Very good	Easy
No	Very good	Easy
Yes	Neutral	Easy
Yes	Very good	Very easy
Yes	Very good	Very easy
Yes	Good	Easy
No	Good	Easy
Yes	Neutral	Easy
No	Neutral	Easy
No	Very good	Easy
Yes	Very good	Very easy
Yes	Very good	Very easy
No	Neutral	Easy
No	Very good	Neutral
Yes	Very good	Very easy
Yes	Good	Easy
Yes	Good	Easy
Yes	Very good	Easy
No	Good	Easy
Yes	Good	Very easy

Yes	Good	Easy
No	Good	Easy
Yes	Good	Easy
No	Neutral	Easy
Yes	Good	Easy
Yes	Very good	Easy
Yes	Very good	Easy
Yes	Good	Easy
No	Neutral	Neutral
Yes	Very good	Very easy
Yes	Very good	Easy
No	Neutral	Easy
Yes	Good	Easy
No	Good	Easy
No	Good	Easy
Yes	Good	Easy
Yes	Very good	Easy
No	Good	Neutral
Yes	Good	Easy
Yes	Neutral	Easy
Yes	Very good	Easy
No	Very good	Very easy
Yes	Very good	Easy
Yes	Very good	Very easy
Yes	Very good	Very easy
No	Neutral	Easy
Yes	Very good	Very easy
Yes	Very good	Very easy
Decline to respond	Good	Neutral
No	Neutral	Easy
Yes	Very good	Easy
Yes	Good	Easy
Yes	Very good	Easy

Yes	Neutral	Hard
Yes	Good	Easy
Yes	Good	Easy
Yes	Very good	Easy
Yes	Neutral	Easy
No	Very good	Easy
Yes	Very good	Very easy
Yes	Good	Easy
Yes	Neutral	Neutral
Yes	Good	Easy
No	Neutral	Neutral
Yes	Neutral	Hard
Yes	Neutral	Neutral
Yes	Neutral	Easy
Yes	Good	Easy
No	Very good	Very easy
Yes	Very good	Easy
Yes	Very good	Easy
Yes	Good	Easy
Yes	Good	Easy
Yes	Good	Easy
Yes	Very bad	Easy
No	Good	Neutral
Yes	Very good	Very easy
Yes	Neutral	Easy
No	Good	Easy
Yes	Good	Easy
Yes	Very good	Very easy
Yes	Very good	Very easy
No	Very good	Easy
Yes	Good	Easy
Yes	Neutral	Easy
Yes	Neutral	Easy

Decline to respond	Neutral	Easy
Yes	Good	Easy
Yes	Good	Easy
Yes	Neutral	Easy
Yes	Very good	Very easy
No	Good	Easy
No	Good	Easy
No	Very good	Easy
No	Good	Easy
Yes	Good	Easy
Yes	Good	Easy
No	Neutral	Easy
Yes	Good	Easy
Yes	Good	Easy
Yes	Neutral	Easy
Yes	Good	Very easy
Yes	Good	Easy
Yes	Neutral	Easy
Yes	Very good	Easy
Yes	Very good	Easy
No	Good	Easy
Yes	Neutral	Easy
No	Neutral	Hard
Yes	Very good	Easy
Yes	Very good	Easy
Yes	Very good	Easy
Yes	Good	Easy
No	Very good	Easy
Yes	Neutral	Easy
Yes	Neutral	Neutral
Yes	Good	Easy
Yes	Very bad	Neutral
Yes	Good	Very easy

Yes	Good	Neutral
Yes	Neutral	Easy
Yes	Very good	Very easy
No	Good	Easy
Yes	Very good	Easy
Yes	Neutral	Easy
No	Very good	Very easy
Yes	Very good	Easy
Yes	Very good	Easy
No	Good	Easy
No	Good	Easy
Yes	Good	Easy
Yes	Good	Easy
Yes	Very good	Easy
Yes	Very good	Very easy
Yes	Neutral	Easy
No	Very good	Very easy
Yes	Good	Easy
No	Good	Easy
No	Neutral	Neutral
No	Neutral	Neutral
No	Good	Easy
Yes	Very good	Easy
Yes	Very good	Very easy
No	Very good	Easy
Yes	Good	Easy
Yes	Good	Hard
Yes	Very good	Very easy
No	Good	Easy
Yes	Very good	Very easy
Yes	Neutral	Easy
Yes	Very good	Very easy
No	Very good	Very easy

No	Neutral	Neutral
No	Very good	Easy
Yes	Good	Easy
Yes	Very good	Easy
Yes	Very good	Easy
Yes	Very good	Neutral
Yes	Very good	Easy
Yes	Good	Easy
No	Very good	Easy
Yes	Very good	Very easy
No	Very good	Very easy
No	Good	Neutral
Yes	Good	Easy
No	Very good	Easy
Yes	Good	Neutral
Yes	Very good	Very easy
Yes	Neutral	Easy
Yes	Neutral	Easy
Yes	Good	Very easy
Yes	Good	Easy
Yes	Very good	Easy
Yes	Very good	Easy
Yes	Neutral	Neutral
Yes	Good	Easy
Yes	Very good	Easy
No	Neutral	Very easy
Yes	Very good	Very easy
No	Very good	Very easy
Yes	Very good	Easy
Yes	Very good	Hard
Yes	Good	Easy
Yes	Good	Easy
Yes	Very good	Easy

Yes	Very good	Easy
Yes	Good	Easy
Yes	Very good	Easy
No	Very bad	Hard
No	Good	Neutral
No	Good	Easy
Yes	Good	Easy
No	Good	Easy
Yes	Good	Neutral
No	Good	Hard
No	Good	Hard
No	Neutral	Hard
Yes	Good	Easy
Yes	Neutral	Easy
Yes	Very good	Easy
No	Good	Easy
Yes	Very good	Easy
Yes	Very good	Very easy
Yes	Good	Very easy
Yes	Very good	Very easy
Yes	Good	Easy
No	Neutral	Easy
No	Very good	Very easy
Yes	Good	Easy
Yes	Very good	Easy
Yes	Very good	Very easy
Yes	Neutral	Easy
No	Good	Very easy
Yes	Good	Easy
Yes	Good	Easy
Yes	Very good	Very easy
No	Bad	Neutral
Yes	Bad	Neutral

No	Neutral	Easy
No	Very good	Very easy
No	Good	Easy
Yes	Very good	Very easy
No	Bad	Neutral
No	Good	Easy
Yes	Good	Hard
Yes	Neutral	Easy
No	Good	Easy
Yes	Good	Easy
No	Very good	Neutral
Yes	Good	Easy
Yes	Very good	Very easy
Yes	Very good	Very easy
Yes	Good	Very easy
Yes	Neutral	Neutral
Yes	Very good	Very easy
Yes	Very good	Easy
No	Good	Easy
No	Very good	Easy
Yes	Very good	Very easy
Yes	Neutral	Hard
Yes	Good	Very easy
Yes	Neutral	Neutral
Yes	Good	Easy
Yes	Very good	Very easy
Yes	Good	Easy
No	Good	Easy
No	Very good	Neutral
No	Very good	Easy
Yes	Good	Easy
No	Neutral	Easy
Yes	Neutral	Very easy

Yes	Very good	Easy
Yes	Neutral	Neutral
No	Good	Easy
Yes	Good	Easy
No	Good	Easy
No	Neutral	Easy
Yes	Good	Easy
Yes	Very good	Very easy
Yes	Very good	Very easy
Yes	Good	Very easy
Yes	Very good	Easy
Yes	Neutral	Easy
Yes	Good	Easy
Yes	Good	Easy
Yes	Good	Easy
No	Good	Easy
Yes	Very good	Very easy
Yes	Good	Easy
Yes	Good	Easy
Yes	Neutral	Neutral
No	Very good	Very easy
Yes	Very good	Easy
Yes	Good	Easy
Yes	Very good	Very easy
Yes	Good	Easy
No	Good	Easy
Yes	Very good	Very easy
Yes	Good	Easy
Yes	Very good	Very easy
No	Neutral	Easy
Yes	Very good	Very easy
Yes	Good	Easy
Yes	Very good	Very easy

No	Neutral	Neutral
No	Neutral	Easy
No	Neutral	Easy
Yes	Good	Easy
No	Very good	Easy
No	Very good	Easy
No	Good	Easy
Yes	Good	Easy
Yes	Very good	Very easy
Yes	Very good	Easy
Yes	Very good	Easy
Yes	Good	Easy
Yes	Very good	Neutral
Yes	Good	Easy
Yes	Very good	Easy
Yes	Very good	Very easy
Yes	Very good	Easy
Yes	Neutral	Easy
Yes	Very good	Easy
Yes	Good	Easy
Yes	Very good	Easy
Yes	Good	Neutral
Yes	Very good	Very easy
No	Neutral	Easy
Yes	Good	Very easy
Yes	Very good	Neutral
No	Neutral	Easy
Yes	Good	Easy
Yes	Good	Easy
Yes	Good	Neutral
Yes	Neutral	Neutral
Yes	Good	Easy
Yes	Neutral	Neutral

Yes	Neutral	Easy
Yes	Neutral	Neutral
No	Good	Easy
No	Good	Easy
No	Good	Easy
Yes	Very good	Easy
No	Good	Easy
Yes	Very good	Easy
No	Good	Easy
No	Very good	Easy
No	Good	Easy
No	Neutral	Easy
Yes	Very good	Neutral
No	Neutral	Easy
Yes	Good	Easy
Yes	Neutral	Neutral
Yes	Neutral	Neutral
Yes	Neutral	Easy
Yes	Neutral	Easy
Yes	Good	Easy
Yes	Good	Easy
Yes	Neutral	Easy
Yes	Good	Easy
Yes	Good	Easy
Yes	Very good	Very easy
Yes	Good	Easy
Yes	Very good	Very easy
No	Neutral	Easy
Yes	Good	Easy
Yes	Good	Easy
Yes	Good	Easy
Yes	Good	Easy
Yes	Very good	Very easy

Yes	Neutral	Easy
Yes	Very good	Neutral
Yes	Good	Easy
Yes	Very good	Very easy
Yes	Neutral	Easy
Yes	Good	Easy
Yes	Good	Easy
Yes	Good	Easy
Yes	Very good	Easy
Yes	Good	Easy
Yes	Good	Easy
No	Very good	Easy
Yes	Good	Easy
No	Neutral	Easy
Yes	Good	Easy
No	Good	Easy
Yes	Neutral	Easy
Yes	Neutral	Easy
Yes	Neutral	Neutral
Yes	Good	Easy
Yes	Very good	Very easy
Yes	Neutral	Neutral
Yes	Very good	Easy
Yes	Good	Easy
Yes	Good	Neutral
Yes	Good	Easy
Yes	Neutral	Neutral
Yes	Very good	Easy
Yes	Good	Easy
Yes	Good	Easy
Yes	Very good	Neutral
Yes	Very good	Easy
No	Very good	Easy

Yes	Very good	Easy
Yes	Good	Easy
Yes	Very good	Very easy
No	Very good	Very easy
Yes	Good	Easy
Yes	Good	Easy
No	Very good	Very easy
No	Good	Easy
Yes	Good	Easy
Yes	Good	Easy
No	Neutral	Neutral
Yes	Neutral	Neutral
Yes	Neutral	Neutral
Yes	Good	Easy
No	Good	Easy
Yes	Neutral	Easy
No	Good	Easy
No	Good	Easy
No	Very good	Very easy
No	Good	Easy
Yes	Neutral	Neutral
No	Good	Easy
Yes	Very good	Easy
Yes	Very good	Neutral
No	Very good	Easy
Yes	Very good	Very easy
Yes	Neutral	Easy
No	Good	Easy
No	Good	Easy
Yes	Very good	Easy
Yes	Good	Easy
Yes	Good	Easy
Yes	Very good	Very easy

No	Good	Easy
No	Bad	Hard
No	Neutral	Easy
Yes	Neutral	Easy
No	Very good	Neutral
No	Very good	Very easy
No	Neutral	Easy
Yes	Very good	Easy
Yes	Good	Very easy
No	Good	Easy
Yes	Good	Very easy
Yes	Neutral	Neutral
No	Good	Easy
Yes	Very good	Easy
Yes	Very good	Easy
Yes	Very good	Easy
Yes	Good	Easy
Yes	Good	Easy
Yes	Good	Easy
Yes	Good	Easy
Yes	Very good	Easy
Yes	Good	Easy
Yes	Good	Very easy
No	Neutral	Easy
Yes	Neutral	Neutral
Yes	Bad	Hard
Yes	Neutral	Neutral
Yes	Good	Neutral
Yes	Neutral	Easy
No	Very good	Easy
Yes	Neutral	Easy
Yes	Good	Hard
No	Good	Easy

Yes	Good	Very easy
Yes	Very good	Easy
Yes	Good	Easy
Yes	Very good	Easy
Yes	Very good	Very easy
Yes	Neutral	Easy
Yes	Neutral	Easy
Yes	Very good	Easy
Yes	Very good	Very easy
Yes	Neutral	Neutral
Yes	Good	Easy
Yes	Neutral	Easy
Yes	Good	Easy
Yes	Very good	Easy
No	Good	Very easy
Yes	Neutral	Easy
Yes	Very good	Very easy
No	Bad	Neutral
Yes	Good	Easy
No	Neutral	Easy
Yes	Very good	Easy
No	Very good	Very easy
Yes	Good	Easy
No	Good	Easy
Yes	Good	Easy
Yes	Neutral	Easy
Yes	Neutral	Neutral
Yes	Very good	Easy
No	Good	Easy
No	Neutral	Very easy
Yes	Very good	Hard
Yes	Neutral	Easy
Yes	Neutral	Easy

Yes	Neutral	Easy
No	Good	Hard
Yes	Very good	Easy
Yes	Neutral	Easy
Yes	Good	Easy
Yes	Very good	Easy
Yes	Good	Very easy
Yes	Neutral	Neutral
Yes	Good	Easy
Yes	Very good	Easy
Yes	Neutral	Hard
Yes	Very good	Easy
Yes	Neutral	Neutral
Yes	Good	Easy
Yes	Neutral	Neutral
Yes	Very good	Easy
Yes	Good	Easy
No	Very good	Very easy
No	Neutral	Neutral
Yes	Very good	Easy
No	Good	Easy
No	Neutral	Neutral
Yes	Good	Easy
Yes	Neutral	Neutral
Yes	Good	Easy
Yes	Good	Neutral
Yes	Neutral	Easy
Yes	Good	Easy
Yes	Good	Easy
Yes	Good	Neutral
No	Very good	Very easy
Yes	Very good	Easy
Yes	Very good	Easy

No	Good	Easy
No	Good	Easy
Yes	Very good	Very easy
Yes	Very good	Easy
Yes	Very good	Easy
Yes	Neutral	Easy
Yes	Very good	Very easy
Yes	Good	Easy
No	Neutral	Hard
Yes	Good	Easy
Yes	Very good	Very easy
Yes	Good	Easy
Yes	Good	Easy
No	Good	Easy
No	Neutral	Neutral
No	Neutral	Easy
No	Good	Neutral
Yes	Good	Easy
Yes	Good	Easy
Yes	Good	Easy
Yes	Good	Hard
No	Good	Easy
No	Good	Very easy
No	Neutral	Neutral
No	Good	Easy
No	Neutral	Easy
Yes	Good	Easy
Yes	Good	Easy
No	Good	Easy
Yes	Very good	Easy
Yes	Good	Very easy
Yes	Neutral	Easy
No	Good	Easy

Yes	Very good	Very easy
Yes	Very good	Easy
Yes	Good	Easy
No	Bad	Easy
Yes	Neutral	Easy
Yes	Good	Easy
Yes	Good	Easy
Yes	Good	Neutral
No	Very good	Very easy
No	Good	Easy
Yes	Good	Easy
Yes	Very good	Easy
Yes	Good	Easy
No	Good	Easy
Yes	Good	Easy
Yes	Good	Easy
Yes	Very good	Easy
Yes	Neutral	Easy
Yes	Neutral	Easy
Yes	Good	Easy
No	Good	Neutral
Yes	Good	Easy
Yes	Good	Easy
Yes	Good	Easy
Yes	Neutral	Neutral
Yes	Neutral	Neutral
No	Neutral	Easy
Yes	Good	Easy
Yes	Very good	Very easy
Yes	Neutral	Neutral
Yes	Good	Easy
No	Good	Easy
Yes	Very bad	Neutral

No	Good	Easy
No	Very good	Neutral
Yes	Neutral	Neutral
No	Bad	Easy
Yes	Good	Easy
Yes	Good	Very easy
No	Neutral	Hard
Yes	Neutral	Easy
Yes	Very bad	Very easy
No	Very good	Easy
No	Good	Easy
Yes	Good	Easy
Yes	Good	Very easy
Yes	Neutral	Easy
Yes	Very good	Very easy
No	Very good	Very easy
No	Good	Easy
Yes	Good	Easy
Yes	Neutral	Neutral
Yes	Very good	Easy
Yes	Good	Neutral
Yes	Neutral	Easy
Yes	Good	Easy
Yes	Very good	Easy
No	Very good	Easy
Yes	Good	Very easy
Yes	Good	Easy
Yes	Good	Easy
Yes	Neutral	Easy
Yes	Very good	Easy
No	Good	Easy
No	Very good	Easy
Yes	Good	Easy

Yes	Neutral	Easy
Yes	Good	Easy
No	Neutral	Easy
Yes	Good	Easy
Yes	Very good	Very easy
No	Very good	Easy
Yes	Neutral	Neutral
Yes	Neutral	Easy
Yes	Neutral	Easy
Yes	Neutral	Easy
Yes	Good	Hard
No	Neutral	Easy
Yes	Good	Easy
Yes	Neutral	Easy
No	Good	Easy
Yes	Good	Easy
Yes	Good	Easy
Yes	Neutral	Hard
No	Good	Easy
Yes	Good	Easy
Yes	Neutral	Neutral
No	Neutral	Easy
Yes	Good	Easy
No	Good	Easy
Yes	Good	Easy
Yes	Good	Neutral
Yes	Good	Easy
Yes	Good	Easy
Yes	Good	Easy
Yes	Good	Neutral
No	Good	Easy
Yes	Good	Easy
Yes	Very good	Easy

Yes	Neutral	Easy
Yes	Very good	Easy
Yes	Good	Easy
No	Neutral	Hard
Yes	Neutral	Neutral
Yes	Good	Easy
No	Good	Neutral
No	Neutral	Neutral
Yes	Neutral	Easy
Yes	Neutral	Easy
Yes	Good	Easy
Yes	Neutral	Easy
Yes	Good	Neutral
Yes	Neutral	Neutral
Yes	Neutral	Easy
Yes	Good	Easy
Yes	Very good	Easy
Yes	Neutral	Neutral
Yes	Neutral	Neutral
Yes	Neutral	Easy
No	Good	Easy
Yes	Very good	Very easy
Yes	Neutral	Easy
Yes	Good	Easy
Yes	Good	Hard
Yes	Good	Easy
Yes	Good	Neutral
No	Very good	Easy
No	Good	Easy
Yes	Good	Easy
Yes	Good	Easy
Yes	Neutral	Easy
Yes	Good	Neutral

No	Neutral	Easy
Yes	Good	Easy
No	Good	Easy
Yes	Good	Easy
Yes	Neutral	Neutral
Yes	Neutral	Easy
Yes	Good	Easy
Yes	Good	Easy
Yes	Neutral	Easy
No	Good	Easy
Yes	Good	Easy
No	Neutral	Neutral
Yes	Very good	Easy
Yes	Good	Easy
Yes	Neutral	Neutral
Yes	Good	Easy
Yes	Good	Easy
Yes	Good	Hard
Yes	Good	Easy
Yes	Good	Easy
No	Neutral	Easy
Yes	Good	Easy
Yes	Neutral	Easy
Yes	Good	Easy
Yes	Good	Easy
Yes	Good	Easy
No	Neutral	Easy
Yes	Neutral	Easy
Yes	Good	Easy
No	Neutral	Easy
No	Very good	Easy
Yes	Good	Easy
No	Neutral	Hard

No	Neutral	Neutral
Yes	Good	Neutral
Yes	Very good	Easy
Yes	Good	Easy
No	Good	Easy
Yes	Good	Easy
Yes	Very good	Easy
Yes	Neutral	Easy
Yes	Good	Easy
Yes	Good	Neutral
Yes	Good	Easy
Yes	Good	Neutral
Yes	Neutral	Easy
Yes	Very good	Easy
Yes	Good	Easy
No	Good	Easy
No	Good	Neutral
Yes	Neutral	Easy
Yes	Good	Neutral
Yes	Very good	Easy
Yes	Very good	Easy
Yes	Neutral	Easy
Yes	Neutral	Easy
Yes	Good	Neutral
No	Good	Neutral
Yes	Good	Hard
Yes	Very good	Neutral
Yes	Good	Easy
Yes	Good	Easy
Yes	Very good	Hard
Yes	Very good	Easy
No	Neutral	Easy
Yes	Good	Easy

Yes	Good	Easy
Yes	Neutral	Neutral
Yes	Very good	Easy
No	Neutral	Easy
Yes	Neutral	Easy
Yes	Neutral	Neutral
Yes	Neutral	Neutral
Yes	Very good	Easy
Yes	Good	Easy
Yes	Very good	Easy
Yes	Good	Hard
Yes	Very good	Easy
Yes	Neutral	Easy
No	Good	Neutral
No	Neutral	Neutral
No	Neutral	Neutral
No	Neutral	Easy
Yes	Good	Easy
Yes	Good	Easy
No	Good	Easy
Yes	Bad	Easy
Yes	Very good	Easy
Yes	Good	Hard
Yes	Neutral	Easy
Yes	Very good	Easy
Yes	Neutral	Easy
No	Very good	Easy
Yes	Good	Easy
No	Neutral	Neutral
Yes	Good	Easy
Yes	Good	Easy
Yes	Good	Easy
Yes	Neutral	Easy

Yes	Neutral	Easy
Yes	Very good	Easy
No	Neutral	Neutral
Yes	Good	Neutral
Yes	Very good	Easy
Yes	Good	Neutral
Yes	Very good	Easy
No	Good	Easy
Yes	Very good	Easy
Yes	Neutral	Easy
Yes	Very good	Easy
Yes	Good	Easy
Yes	Neutral	Neutral
Yes	Very good	Easy
Yes	Good	Easy
Yes	Very good	Easy
Yes	Very good	Easy
Yes	Good	Easy
Yes	Neutral	Easy
Yes	Good	Easy
Yes	Good	Easy
Yes	Very good	Easy
Yes	Very good	Easy
Yes	Very good	Easy
Yes	Good	Easy
Yes	Good	Easy
Yes	Good	Easy
Yes	Good	Very easy
No	Very good	Neutral
No	Neutral	Easy
Yes	Good	Easy
No	Good	Easy
Yes	Good	Easy

Yes	Good	Easy
Yes	Very good	Easy
No	Good	Easy
Yes	Very good	Neutral
Yes	Very good	Easy
Yes	Very good	Easy
No	Very good	Easy
Yes	Good	Easy
Yes	Good	Easy
No	Very good	Easy
Yes	Very good	Easy
Yes	Good	Hard
Yes	Good	Easy
Yes	Neutral	Easy
No	Neutral	Hard
Yes	Very good	Easy
Yes	Neutral	Easy
No	Good	Easy
Yes	Good	Easy
Yes	Good	Neutral
Yes	Very good	Easy
No	Very good	Easy
Yes	Very good	Easy
Yes	Very good	Easy
No	Good	Easy
Yes	Neutral	Neutral
Yes	Very good	Easy
Yes	Very good	Easy
Yes	Good	Easy
Yes	Neutral	Neutral
Yes	Good	Neutral
Yes	Very good	Easy
No	Good	Neutral

Yes	Good	Easy
Yes	Very good	Easy
Yes	Good	Easy
Yes	Good	Easy
Yes	Very good	Easy
No	Good	Easy
Yes	Very good	Easy
Yes	Neutral	Easy
Yes	Good	Neutral
Yes	Good	Easy
Yes	Very good	Neutral
No	Good	Easy
Yes	Very good	Easy
Yes	Very good	Easy
Yes	Good	Easy
Yes	Good	Neutral
Yes	Neutral	Hard
Yes	Very good	Easy
Yes	Neutral	Neutral
Yes	Very good	Hard

Do you think self-sampling collection is a convenient met	Will you feel embarrassed to collect your own sample?	Do you think self-sampling collection will cause discomfo
Neutral	Not embarrassed	No discomfort/pain
Convenient	Not embarrassed	No discomfort/pain
Very convenient	Not embarrassed at all	No discomfort/pain
Very convenient	Not embarrassed at all	Neutral
Convenient	Not embarrassed	Neutral
Convenient	Not embarrassed	Some discomfort/pain
Convenient	Not embarrassed	No discomfort/pain
Very convenient	Not embarrassed at all	Some discomfort/pain
Convenient	Not embarrassed	Neutral
Convenient	Not embarrassed	No discomfort/pain
Very convenient	Not embarrassed	Some discomfort/pain
Convenient	Not embarrassed	No discomfort/pain
Convenient	Not embarrassed	Some discomfort/pain
Convenient	Not embarrassed	Some discomfort/pain
Convenient	Not embarrassed	No discomfort/pain
Convenient	Not embarrassed	No discomfort/pain
Convenient	Not embarrassed	Some discomfort/pain
Convenient	Not embarrassed	Neutral
Neutral	Not embarrassed	Neutral
Convenient	Not embarrassed	Some discomfort/pain
Convenient	Not embarrassed	No discomfort/pain
Convenient	Not embarrassed	Some discomfort/pain
Very convenient	Not embarrassed at all	No discomfort/pain
Neutral	Not embarrassed	No discomfort/pain
Convenient	Not embarrassed at all	Some discomfort/pain
Convenient	Not embarrassed	No discomfort/pain
Convenient	Not embarrassed	No discomfort/pain at all
Convenient	Not embarrassed	Some discomfort/pain
Neutral	Not embarrassed	No discomfort/pain
Very convenient	Not embarrassed at all	Neutral
Convenient	Not embarrassed	No discomfort/pain
Convenient	Not embarrassed	Some discomfort/pain

Convenient	Neutral	Some discomfort/pain
Convenient	Not embarrassed	Some discomfort/pain
Neutral	Not embarrassed	Neutral
Convenient	Not embarrassed	No discomfort/pain
Convenient	Not embarrassed at all	Neutral
Convenient	Not embarrassed	No discomfort/pain
Convenient	Not embarrassed at all	Some discomfort/pain
Convenient	Not embarrassed	Neutral
Convenient	Neutral	No discomfort/pain
Convenient	Not embarrassed	Neutral
Convenient	Not embarrassed	No discomfort/pain
Convenient	Not embarrassed	No discomfort/pain
Convenient	Not embarrassed	No discomfort/pain
Convenient	Not embarrassed	No discomfort/pain
Convenient	Not embarrassed at all	Neutral
Convenient	Not embarrassed	Some discomfort/pain
Convenient	Not embarrassed	Some discomfort/pain
Convenient	Not embarrassed	No discomfort/pain
Convenient	Not embarrassed	Some discomfort/pain
Neutral	Not embarrassed at all	Some discomfort/pain
Convenient	Not embarrassed	No discomfort/pain
Convenient	Not embarrassed at all	Neutral
Neutral	Not embarrassed at all	Neutral
Convenient	Not embarrassed at all	No discomfort/pain
Convenient	Not embarrassed	Neutral
Convenient	Not embarrassed at all	Some discomfort/pain
Convenient	Not embarrassed	Some discomfort/pain
Convenient	Not embarrassed	Neutral
Convenient	Not embarrassed at all	No discomfort/pain
Convenient	Not embarrassed at all	No discomfort/pain
Convenient	Not embarrassed	Some discomfort/pain
Neutral	Not embarrassed	Some discomfort/pain
Very convenient	Not embarrassed at all	No discomfort/pain

Convenient	Not embarrassed	No discomfort/pain
Convenient	Not embarrassed	No discomfort/pain
Very convenient	Not embarrassed at all	Some discomfort/pain
Convenient	Not embarrassed at all	No discomfort/pain
Convenient	Not embarrassed	No discomfort/pain
Convenient	Not embarrassed at all	No discomfort/pain
Convenient	Not embarrassed	Neutral
Convenient	Not embarrassed	Some discomfort/pain
Very convenient	Not embarrassed at all	No discomfort/pain
Convenient	Neutral	No discomfort/pain
Very convenient	Not embarrassed at all	No discomfort/pain
Convenient	Not embarrassed	Some discomfort/pain
Convenient	Not embarrassed at all	Neutral
Convenient	Embarrassed	Some discomfort/pain
Convenient	Not embarrassed at all	Some discomfort/pain
Convenient	Not embarrassed	Some discomfort/pain
Convenient	Not embarrassed at all	No discomfort/pain
Convenient	Not embarrassed at all	Neutral
Convenient	Not embarrassed at all	No discomfort/pain
Convenient	Not embarrassed at all	Neutral
Very convenient	Not embarrassed at all	Neutral
Convenient	Not embarrassed at all	No discomfort/pain
Very convenient	Not embarrassed at all	Some discomfort/pain
Very convenient	Not embarrassed at all	No discomfort/pain
Convenient	Not embarrassed at all	Some discomfort/pain
Convenient	Not embarrassed at all	Neutral
Convenient	Not embarrassed at all	No discomfort/pain
Convenient	Not embarrassed at all	No discomfort/pain
Convenient	Not embarrassed	Some discomfort/pain
Very convenient	Not embarrassed at all	Some discomfort/pain
Convenient	Not embarrassed at all	No discomfort/pain
Convenient	Not embarrassed at all	Some discomfort/pain
Convenient	Not embarrassed at all	No discomfort/pain

Convenient	Not embarrassed	Neutral
Convenient	Not embarrassed at all	No discomfort/pain
Convenient	Not embarrassed at all	Some discomfort/pain
Convenient	Not embarrassed at all	No discomfort/pain
Convenient	Not embarrassed at all	Some discomfort/pain
Convenient	Not embarrassed at all	No discomfort/pain
Convenient	Not embarrassed at all	Some discomfort/pain
Convenient	Not embarrassed	No discomfort/pain
Convenient	Not embarrassed at all	No discomfort/pain
Convenient	Not embarrassed at all	No discomfort/pain
Convenient	Not embarrassed at all	No discomfort/pain
Convenient	Not embarrassed at all	Neutral
Very convenient	Not embarrassed at all	No discomfort/pain
Convenient	Not embarrassed at all	No discomfort/pain
Not convenient	Not embarrassed	No discomfort/pain
Convenient	Not embarrassed at all	Severe discomfort/pain
Convenient	Not embarrassed at all	No discomfort/pain
Convenient	Not embarrassed at all	Some discomfort/pain
Convenient	Not embarrassed at all	Neutral
Convenient	Not embarrassed at all	No discomfort/pain
Convenient	Not embarrassed	Some discomfort/pain
Convenient	Not embarrassed	No discomfort/pain
Convenient	Not embarrassed at all	No discomfort/pain
Convenient	Not embarrassed at all	Some discomfort/pain
Convenient	Not embarrassed at all	Some discomfort/pain
Convenient	Not embarrassed at all	Some discomfort/pain
Convenient	Not embarrassed at all	No discomfort/pain
Convenient	Embarrassed	Neutral
Convenient	Not embarrassed at all	No discomfort/pain
Convenient	Not embarrassed at all	No discomfort/pain
Convenient	Embarrassed	Neutral
Convenient	Not embarrassed at all	Neutral
Convenient	Not embarrassed at all	No discomfort/pain

Convenient	Not embarrassed at all	Some discomfort/pain
Convenient	Not embarrassed at all	No discomfort/pain
Convenient	Not embarrassed at all	Some discomfort/pain
Convenient	Not embarrassed at all	No discomfort/pain
Convenient	Not embarrassed at all	No discomfort/pain
Convenient	Embarrassed	Some discomfort/pain
Convenient	Not embarrassed at all	Some discomfort/pain
Neutral	Not embarrassed	Neutral
Very convenient	Not embarrassed at all	No discomfort/pain
Convenient	Not embarrassed at all	Some discomfort/pain
Neutral	Not embarrassed at all	Some discomfort/pain
Convenient	Not embarrassed at all	No discomfort/pain
Very convenient	Not embarrassed at all	Neutral
Convenient	Not embarrassed at all	No discomfort/pain
Convenient	Not embarrassed at all	Neutral
Convenient	Not embarrassed at all	No discomfort/pain
Neutral	Not embarrassed at all	Neutral
Convenient	Not embarrassed at all	Neutral
Convenient	Not embarrassed at all	Neutral
Convenient	Not embarrassed	Neutral
Convenient	Not embarrassed	Some discomfort/pain
Convenient	Embarrassed	Some discomfort/pain
Convenient	Not embarrassed at all	Some discomfort/pain
Convenient	Not embarrassed	No discomfort/pain
Convenient	Not embarrassed at all	Some discomfort/pain
Convenient	Not embarrassed at all	Some discomfort/pain
Convenient	Not embarrassed at all	Some discomfort/pain
Convenient	Neutral	Some discomfort/pain
Convenient	Not embarrassed	No discomfort/pain
Convenient	Not embarrassed at all	Neutral
Convenient	Not embarrassed at all	No discomfort/pain
Convenient	Not embarrassed	Some discomfort/pain
Convenient	Not embarrassed at all	Some discomfort/pain

Convenient	Not embarrassed at all	Severe discomfort/pain
Convenient	Not embarrassed at all	No discomfort/pain
Convenient	Neutral	Some discomfort/pain
Convenient	Not embarrassed at all	No discomfort/pain
Convenient	Embarrassed	Neutral
Convenient	Not embarrassed at all	No discomfort/pain
Convenient	Not embarrassed at all	No discomfort/pain
Convenient	Not embarrassed	Neutral
Convenient	Not embarrassed	Some discomfort/pain
Convenient	Not embarrassed at all	Neutral
Convenient	Not embarrassed at all	Some discomfort/pain
Convenient	Not embarrassed at all	No discomfort/pain
Convenient	Not embarrassed	No discomfort/pain
Convenient	Not embarrassed at all	No discomfort/pain
Neutral	Not embarrassed at all	Neutral
Convenient	Not embarrassed at all	No discomfort/pain
Convenient	Not embarrassed at all	No discomfort/pain
Convenient	Not embarrassed at all	Neutral
Convenient	Not embarrassed at all	Neutral
Convenient	Not embarrassed	Neutral
Neutral	Not embarrassed	Some discomfort/pain
Convenient	Not embarrassed at all	Neutral
Convenient	Not embarrassed at all	No discomfort/pain
Convenient	Not embarrassed at all	No discomfort/pain
Convenient	Not embarrassed at all	Neutral
Convenient	Not embarrassed at all	Some discomfort/pain
Convenient	Not embarrassed at all	Neutral
Convenient	Neutral	Neutral
Convenient	Not embarrassed at all	No discomfort/pain
Convenient	Not embarrassed at all	No discomfort/pain
Convenient	Not embarrassed at all	No discomfort/pain
Convenient	Neutral	Neutral
Convenient	Not embarrassed at all	No discomfort/pain

Convenient	Not embarrassed at all	Some discomfort/pain
Convenient	Not embarrassed at all	No discomfort/pain
Convenient	Not embarrassed at all	No discomfort/pain
Convenient	Not embarrassed at all	No discomfort/pain
Convenient	Not embarrassed at all	No discomfort/pain
Convenient	Not embarrassed at all	Some discomfort/pain
Convenient	Not embarrassed at all	Some discomfort/pain
Convenient	Not embarrassed at all	Neutral
Convenient	Not embarrassed	Some discomfort/pain
Convenient	Not embarrassed	Some discomfort/pain
Convenient	Not embarrassed at all	Some discomfort/pain
Convenient	Not embarrassed	Neutral
Convenient	Not embarrassed	Neutral
Convenient	Not embarrassed at all	Neutral
Convenient	Not embarrassed at all	No discomfort/pain
Convenient	Embarrassed	No discomfort/pain
Convenient	Not embarrassed at all	Some discomfort/pain
Neutral	Embarrassed	Neutral
Convenient	Not embarrassed	Some discomfort/pain
Convenient	Neutral	Neutral
Convenient	Not embarrassed at all	Some discomfort/pain
Convenient	Not embarrassed	Some discomfort/pain
Very convenient	Not embarrassed at all	Some discomfort/pain
Convenient	Not embarrassed at all	Some discomfort/pain
Convenient	Not embarrassed at all	Some discomfort/pain
Very convenient	Not embarrassed at all	No discomfort/pain
Convenient	Not embarrassed at all	Some discomfort/pain
Convenient	Not embarrassed at all	Some discomfort/pain
Convenient	Not embarrassed at all	Some discomfort/pain
Convenient	Neutral	Some discomfort/pain
Convenient	Neutral	Some discomfort/pain
Convenient	Not embarrassed at all	No discomfort/pain
Convenient	Not embarrassed at all	No discomfort/pain

Convenient	Not embarrassed	No discomfort/pain
Convenient	Not embarrassed at all	Neutral
Neutral	Neutral	Some discomfort/pain
Convenient	Not embarrassed at all	No discomfort/pain
Convenient	Not embarrassed at all	Neutral
Convenient	Not embarrassed at all	Some discomfort/pain
Very convenient	Not embarrassed at all	Neutral
Convenient	Not embarrassed at all	Some discomfort/pain
Very convenient	Not embarrassed at all	Some discomfort/pain
Convenient	Not embarrassed at all	Some discomfort/pain
Convenient	Not embarrassed at all	Some discomfort/pain
Convenient	Not embarrassed at all	Neutral
Convenient	Not embarrassed at all	Some discomfort/pain
Convenient	Not embarrassed at all	Neutral
Convenient	Not embarrassed at all	Some discomfort/pain
Convenient	Not embarrassed at all	No discomfort/pain
Convenient	Not embarrassed at all	No discomfort/pain
Very convenient	Not embarrassed at all	Some discomfort/pain
Very convenient	Not embarrassed at all	No discomfort/pain
Convenient	Not embarrassed at all	Neutral
Convenient	Not embarrassed at all	Neutral
Neutral	Not embarrassed at all	Some discomfort/pain
Convenient	Not embarrassed at all	Neutral
Convenient	Not embarrassed at all	No discomfort/pain
Convenient	Not embarrassed at all	No discomfort/pain
Very convenient	Not embarrassed at all	No discomfort/pain
Convenient	Neutral	Neutral
Convenient	Not embarrassed at all	No discomfort/pain
Convenient	Not embarrassed at all	No discomfort/pain
Convenient	Not embarrassed at all	Neutral
Convenient	Not embarrassed at all	No discomfort/pain
Neutral	Neutral	Neutral
Convenient	Not embarrassed at all	No discomfort/pain

Convenient	Not embarrassed at all	Neutral
Neutral	Not embarrassed at all	Some discomfort/pain
Convenient	Not embarrassed at all	Neutral
Convenient	Not embarrassed at all	Some discomfort/pain
Convenient	Not embarrassed at all	Neutral
Convenient	Not embarrassed at all	Neutral
Very convenient	Not embarrassed at all	Some discomfort/pain
Convenient	Not embarrassed at all	Neutral
Convenient	Not embarrassed at all	Neutral
Convenient	Not embarrassed at all	Some discomfort/pain
Convenient	Neutral	Some discomfort/pain
Neutral	Not embarrassed	Neutral
Convenient	Not embarrassed at all	Some discomfort/pain
Convenient	Not embarrassed at all	Neutral
Convenient	Not embarrassed at all	Neutral
Convenient	Not embarrassed at all	Some discomfort/pain
Convenient	Not embarrassed at all	Neutral
Convenient	Not embarrassed	No discomfort/pain
Convenient	Not embarrassed at all	Neutral
Convenient	Embarrassed	Some discomfort/pain
Convenient	Not embarrassed at all	Neutral
Convenient	Not embarrassed at all	No discomfort/pain
Convenient	Not embarrassed at all	Neutral
Convenient	Not embarrassed at all	Neutral
Convenient	Not embarrassed at all	Some discomfort/pain
Convenient	Not embarrassed	No discomfort/pain
Convenient	Not embarrassed	Some discomfort/pain
Neutral	Not embarrassed at all	Some discomfort/pain
Convenient	Not embarrassed at all	Neutral
Convenient	Not embarrassed at all	No discomfort/pain
Convenient	Not embarrassed at all	Some discomfort/pain
Convenient	Not embarrassed at all	Some discomfort/pain
Convenient	Not embarrassed at all	Some discomfort/pain

Convenient	Not embarrassed at all	Neutral
Convenient	Not embarrassed at all	Neutral
Convenient	Not embarrassed at all	Severe discomfort/pain
Very convenient	Not embarrassed	No discomfort/pain
Convenient	Not embarrassed at all	Some discomfort/pain
Convenient	Not embarrassed at all	Some discomfort/pain
Convenient	Not embarrassed	Some discomfort/pain
Convenient	Not embarrassed at all	No discomfort/pain
Convenient	Not embarrassed at all	No discomfort/pain
Convenient	Not embarrassed at all	Some discomfort/pain
Convenient	Not embarrassed at all	Some discomfort/pain
Convenient	Not embarrassed at all	No discomfort/pain
Convenient	Not embarrassed at all	Neutral
Convenient	Not embarrassed at all	Some discomfort/pain
Convenient	Not embarrassed	Some discomfort/pain
Neutral	Not embarrassed at all	Neutral
Convenient	Not embarrassed at all	Some discomfort/pain
Convenient	Not embarrassed at all	Neutral
Convenient	Not embarrassed at all	No discomfort/pain
Convenient	Not embarrassed at all	Neutral
Convenient	Not embarrassed at all	Neutral
Convenient	Not embarrassed at all	Some discomfort/pain
Convenient	Neutral	No discomfort/pain
Convenient	Not embarrassed at all	Neutral
Very convenient	Not embarrassed at all	No discomfort/pain
Convenient	Not embarrassed at all	Severe discomfort/pain
Convenient	Embarrassed	Some discomfort/pain
Convenient	Not embarrassed	No discomfort/pain
Convenient	Not embarrassed	No discomfort/pain
Convenient	Not embarrassed	No discomfort/pain
Convenient	Not embarrassed	Neutral
Convenient	Not embarrassed	No discomfort/pain
Convenient	Not embarrassed	No discomfort/pain

Convenient	Not embarrassed	Some discomfort/pain
Convenient	Not embarrassed	No discomfort/pain
Convenient	Embarrassed	Some discomfort/pain
Convenient	Not embarrassed	No discomfort/pain
Convenient	Not embarrassed	Some discomfort/pain
Convenient	Not embarrassed	Some discomfort/pain
Very convenient	Not embarrassed	No discomfort/pain
Convenient	Not embarrassed	Neutral
Convenient	Not embarrassed	No discomfort/pain
Convenient	Not embarrassed	Some discomfort/pain
Convenient	Not embarrassed	Neutral
Convenient	Not embarrassed	Neutral
Convenient	Not embarrassed	No discomfort/pain
Convenient	Not embarrassed	No discomfort/pain
Convenient	Not embarrassed	Neutral
Neutral	Not embarrassed	Some discomfort/pain
Convenient	Not embarrassed	Neutral
Convenient	Not embarrassed	Neutral
Convenient	Not embarrassed	Neutral
Convenient	Not embarrassed	Neutral
Convenient	Not embarrassed	Neutral
Very convenient	Not embarrassed at all	No discomfort/pain
Convenient	Not embarrassed	Neutral
Convenient	Not embarrassed	No discomfort/pain
Convenient	Not embarrassed	Some discomfort/pain
Convenient	Not embarrassed	Neutral
Convenient	Not embarrassed at all	Some discomfort/pain
Convenient	Not embarrassed	Neutral
Convenient	Not embarrassed	No discomfort/pain
Very convenient	Not embarrassed at all	Some discomfort/pain
Convenient	Not embarrassed	Neutral
Convenient	Not embarrassed	No discomfort/pain
Convenient	Not embarrassed	Some discomfort/pain

Very convenient	Not embarrassed at all	No discomfort/pain
Very convenient	Not embarrassed at all	No discomfort/pain at all
Very convenient	Not embarrassed at all	No discomfort/pain
Convenient	Not embarrassed	Some discomfort/pain
Very convenient	Not embarrassed at all	Neutral
Convenient	Not embarrassed	Neutral
Convenient	Not embarrassed	No discomfort/pain
Convenient	Not embarrassed at all	Neutral
Convenient	Not embarrassed	No discomfort/pain
Convenient	Not embarrassed at all	No discomfort/pain
Not convenient	Very embarrassed	Severe discomfort/pain
Neutral	Not embarrassed	Some discomfort/pain
Convenient	Not embarrassed	Neutral
Convenient	Not embarrassed at all	Some discomfort/pain
Convenient	Not embarrassed at all	Neutral
Convenient	Not embarrassed at all	Severe discomfort/pain
Convenient	Not embarrassed	No discomfort/pain
Convenient	Not embarrassed	No discomfort/pain
Very convenient	Not embarrassed at all	Neutral
Convenient	Not embarrassed	Neutral
Convenient	Not embarrassed at all	Some discomfort/pain
Convenient	Not embarrassed	Some discomfort/pain
Very convenient	Not embarrassed at all	No discomfort/pain
Very convenient	Not embarrassed at all	No discomfort/pain at all
Very convenient	Not embarrassed at all	Neutral
Convenient	Not embarrassed	Neutral
Convenient	Not embarrassed	Neutral
Very convenient	Not embarrassed	Some discomfort/pain
Convenient	Not embarrassed	Neutral
Not convenient	Not embarrassed	Some discomfort/pain
Convenient	Not embarrassed at all	No discomfort/pain
Very convenient	Not embarrassed at all	Neutral
Neutral	Not embarrassed at all	Neutral

Convenient	Not embarrassed at all	No discomfort/pain at all
Very convenient	Not embarrassed at all	No discomfort/pain at all
Very convenient	Not embarrassed at all	Some discomfort/pain
Convenient	Not embarrassed	Neutral
Very convenient	Not embarrassed at all	Neutral
Convenient	Embarrassed	Some discomfort/pain
Very convenient	Not embarrassed at all	No discomfort/pain at all
Convenient	Not embarrassed at all	Neutral
Very convenient	Not embarrassed at all	No discomfort/pain
Convenient	Not embarrassed at all	Some discomfort/pain
Convenient	Not embarrassed	Neutral
Convenient	Not embarrassed at all	No discomfort/pain
Convenient	Not embarrassed at all	No discomfort/pain
Convenient	Not embarrassed	Some discomfort/pain
Convenient	Not embarrassed at all	No discomfort/pain
Convenient	Not embarrassed at all	Neutral
Convenient	Not embarrassed at all	Some discomfort/pain
Convenient	Not embarrassed at all	No discomfort/pain
Convenient	Embarrassed	No discomfort/pain
Convenient	Not embarrassed at all	Neutral
Convenient	Not embarrassed at all	No discomfort/pain
Convenient	Not embarrassed	Some discomfort/pain
Convenient	Embarrassed	Some discomfort/pain
Convenient	Not embarrassed	No discomfort/pain
Convenient	Not embarrassed at all	Some discomfort/pain
Convenient	Not embarrassed at all	Some discomfort/pain
Not convenient	Not embarrassed at all	Some discomfort/pain
Convenient	Not embarrassed at all	Neutral
Convenient	Not embarrassed	No discomfort/pain
Convenient	Not embarrassed at all	Some discomfort/pain
Convenient	Not embarrassed at all	Neutral
Very convenient	Not embarrassed at all	No discomfort/pain at all
Very convenient	Not embarrassed at all	Some discomfort/pain

Very convenient	Not embarrassed at all	No discomfort/pain
Very convenient	Not embarrassed at all	No discomfort/pain at all
Convenient	Not embarrassed at all	Neutral
Convenient	Not embarrassed	No discomfort/pain
Very convenient	Not embarrassed at all	No discomfort/pain
Convenient	Not embarrassed at all	Some discomfort/pain
Convenient	Neutral	Neutral
Convenient	Not embarrassed	Some discomfort/pain
Convenient	Not embarrassed	Some discomfort/pain
Very convenient	Not embarrassed at all	Neutral
Convenient	Not embarrassed at all	Neutral
Very convenient	Not embarrassed at all	Some discomfort/pain
Very convenient	Not embarrassed at all	No discomfort/pain
Very convenient	Not embarrassed at all	Neutral
Very convenient	Not embarrassed at all	Neutral
Very convenient	Not embarrassed at all	No discomfort/pain at all
Very convenient	Not embarrassed at all	No discomfort/pain at all
Very convenient	Embarrassed	Some discomfort/pain
Convenient	Neutral	Some discomfort/pain
Convenient	Not embarrassed	Neutral
Not convenient	Not embarrassed at all	Some discomfort/pain
Very convenient	Not embarrassed at all	Neutral
Not convenient	Embarrassed	Some discomfort/pain
Convenient	Not embarrassed	No discomfort/pain
Convenient	Not embarrassed	Some discomfort/pain
Convenient	Neutral	Some discomfort/pain
Convenient	Not embarrassed	No discomfort/pain
Convenient	Not embarrassed	No discomfort/pain
Very convenient	Not embarrassed at all	No discomfort/pain at all
Convenient	Not embarrassed at all	Neutral
Very convenient	Not embarrassed at all	No discomfort/pain
Very convenient	Not embarrassed at all	Some discomfort/pain
Convenient	Not embarrassed	No discomfort/pain

Very convenient	Not embarrassed at all	No discomfort/pain at all
Convenient	Not embarrassed	Some discomfort/pain
Very convenient	Not embarrassed at all	No discomfort/pain
Very convenient	Not embarrassed at all	No discomfort/pain
Very convenient	Not embarrassed at all	No discomfort/pain
Very convenient	Not embarrassed at all	No discomfort/pain
Convenient	Not embarrassed	Some discomfort/pain
Convenient	Not embarrassed	Some discomfort/pain
Convenient	Not embarrassed at all	No discomfort/pain
Very convenient	Not embarrassed at all	Some discomfort/pain
Convenient	Not embarrassed at all	Some discomfort/pain
Convenient	Not embarrassed at all	No discomfort/pain
Convenient	Not embarrassed	No discomfort/pain
Very convenient	Not embarrassed at all	No discomfort/pain at all
Neutral	Not embarrassed	Neutral
Convenient	Neutral	No discomfort/pain
Neutral	Not embarrassed	Some discomfort/pain
Convenient	Not embarrassed at all	Some discomfort/pain
Convenient	Not embarrassed at all	Neutral
Convenient	Not embarrassed	Severe discomfort/pain
Neutral	Embarrassed	Some discomfort/pain
Convenient	Not embarrassed	Some discomfort/pain
Very convenient	Not embarrassed at all	No discomfort/pain
Convenient	Not embarrassed at all	Some discomfort/pain
Neutral	Not embarrassed	Some discomfort/pain
Convenient	Not embarrassed at all	No discomfort/pain
Convenient	Neutral	Some discomfort/pain
Convenient	Not embarrassed at all	No discomfort/pain
Convenient	Not embarrassed at all	No discomfort/pain
Convenient	Not embarrassed at all	Some discomfort/pain
Convenient	Not embarrassed	Neutral
Convenient	Not embarrassed	Neutral
Convenient	Not embarrassed	No discomfort/pain

Convenient	Not embarrassed at all	No discomfort/pain
Convenient	Not embarrassed	Neutral
Very convenient	Not embarrassed at all	No discomfort/pain
Very convenient	Not embarrassed at all	No discomfort/pain
Convenient	Not embarrassed at all	No discomfort/pain
Very convenient	Not embarrassed at all	No discomfort/pain
Very convenient	Not embarrassed at all	Some discomfort/pain
Convenient	Not embarrassed at all	Some discomfort/pain
Convenient	Not embarrassed at all	Neutral
Convenient	Not embarrassed	Some discomfort/pain
Neutral	Neutral	Neutral
Very convenient	Not embarrassed at all	No discomfort/pain at all
Convenient	Not embarrassed at all	Neutral
Convenient	Not embarrassed	Some discomfort/pain
Convenient	Not embarrassed at all	Some discomfort/pain
Convenient	Embarrassed	Some discomfort/pain
Convenient	Not embarrassed	No discomfort/pain
Very convenient	Not embarrassed at all	No discomfort/pain at all
Convenient	Not embarrassed at all	Some discomfort/pain
Very convenient	Not embarrassed at all	No discomfort/pain
Convenient	Neutral	Some discomfort/pain
Convenient	Not embarrassed at all	Neutral
Convenient	Not embarrassed	Some discomfort/pain
Convenient	Not embarrassed at all	No discomfort/pain
Convenient	Not embarrassed at all	No discomfort/pain at all
Very convenient	Not embarrassed at all	No discomfort/pain
Very convenient	Not embarrassed at all	No discomfort/pain at all
Very convenient	Not embarrassed at all	Neutral
Very convenient	Not embarrassed	Some discomfort/pain
Very convenient	Not embarrassed	Some discomfort/pain
Convenient	Embarrassed	No discomfort/pain
Convenient	Embarrassed	Some discomfort/pain
Convenient	Not embarrassed at all	Neutral

Neutral	Not embarrassed at all	No discomfort/pain
Very convenient	Not embarrassed at all	Neutral
Very convenient	Not embarrassed at all	No discomfort/pain
Very convenient	Not embarrassed at all	Some discomfort/pain
Very convenient	Not embarrassed at all	No discomfort/pain at all
Very convenient	Not embarrassed at all	Neutral
Convenient	Not embarrassed at all	Some discomfort/pain
Very convenient	Not embarrassed at all	No discomfort/pain
Very convenient	Not embarrassed at all	No discomfort/pain
Very convenient	Not embarrassed at all	Neutral
Convenient	Neutral	Some discomfort/pain
Very convenient	Not embarrassed at all	Some discomfort/pain
Convenient	Not embarrassed at all	Some discomfort/pain
Very convenient	Not embarrassed at all	No discomfort/pain at all
Neutral	Embarrassed	Neutral
Very convenient	Not embarrassed at all	No discomfort/pain at all
Convenient	Not embarrassed at all	Some discomfort/pain
Very convenient	Not embarrassed at all	No discomfort/pain
Very convenient	Not embarrassed at all	No discomfort/pain at all
Convenient	Not embarrassed at all	Neutral
Convenient	Not embarrassed at all	Some discomfort/pain
Convenient	Not embarrassed at all	Some discomfort/pain
Very convenient	Not embarrassed at all	No discomfort/pain
Neutral	Not embarrassed at all	Neutral
Convenient	Not embarrassed at all	No discomfort/pain at all
Convenient	Not embarrassed at all	Neutral
Very convenient	Not embarrassed at all	Some discomfort/pain
Very convenient	Not embarrassed at all	No discomfort/pain
Convenient	Embarrassed	Some discomfort/pain
Very convenient	Not embarrassed at all	Neutral
Convenient	Not embarrassed at all	Neutral
Very convenient	Not embarrassed at all	Some discomfort/pain
Very convenient	Not embarrassed at all	Some discomfort/pain

Very convenient	Not embarrassed at all	Neutral
Convenient	Not embarrassed at all	No discomfort/pain at all
Very convenient	Not embarrassed at all	Some discomfort/pain
Neutral	Not embarrassed at all	Some discomfort/pain
Very convenient	Not embarrassed at all	Neutral
Very convenient	Not embarrassed at all	Neutral
Convenient	Not embarrassed at all	No discomfort/pain
Very convenient	Not embarrassed at all	Neutral
Convenient	Embarrassed	Some discomfort/pain
Convenient	Not embarrassed at all	Neutral
Very convenient	Not embarrassed at all	No discomfort/pain
Convenient	Not embarrassed	Neutral
Neutral	Not embarrassed at all	No discomfort/pain
Very convenient	Not embarrassed at all	No discomfort/pain
Convenient	Not embarrassed at all	No discomfort/pain
Very convenient	Not embarrassed at all	No discomfort/pain at all
Very convenient	Not embarrassed	No discomfort/pain at all
Convenient	Not embarrassed at all	Neutral
Neutral	Not embarrassed at all	Some discomfort/pain
Convenient	Not embarrassed at all	Some discomfort/pain
Very convenient	Not embarrassed at all	No discomfort/pain
Convenient	Very embarrassed	Some discomfort/pain
Very convenient	Not embarrassed at all	No discomfort/pain at all
Very convenient	Not embarrassed at all	Neutral
Very convenient	Not embarrassed at all	Some discomfort/pain
Very convenient	Neutral	Neutral
Convenient	Not embarrassed	Some discomfort/pain
Very convenient	Not embarrassed at all	No discomfort/pain
Very convenient	Not embarrassed at all	No discomfort/pain
Very convenient	Not embarrassed at all	Some discomfort/pain
Very convenient	Not embarrassed at all	Neutral
Convenient	Not embarrassed at all	No discomfort/pain
Convenient	Not embarrassed	Neutral

Very convenient	Not embarrassed at all	No discomfort/pain at all
Very convenient	Not embarrassed at all	No discomfort/pain
Convenient	Not embarrassed	Some discomfort/pain
Convenient	Not embarrassed at all	Neutral
Very convenient	Not embarrassed at all	No discomfort/pain at all
Convenient	Not embarrassed at all	Neutral
Very convenient	Not embarrassed at all	Neutral
Convenient	Not embarrassed at all	Neutral
Very convenient	Not embarrassed at all	No discomfort/pain at all
Very convenient	Not embarrassed at all	Neutral
Convenient	Not embarrassed at all	Some discomfort/pain
Convenient	Not embarrassed at all	Neutral
Convenient	Not embarrassed at all	Neutral
Convenient	Not embarrassed at all	Some discomfort/pain
Convenient	Neutral	Some discomfort/pain
Neutral	Neutral	Some discomfort/pain
Neutral	Not embarrassed at all	Neutral
Very convenient	Not embarrassed at all	No discomfort/pain
Very convenient	Not embarrassed at all	No discomfort/pain at all
Neutral	Not embarrassed at all	Some discomfort/pain
Very convenient	Not embarrassed at all	Neutral
Very convenient	Not embarrassed at all	No discomfort/pain
Convenient	Not embarrassed at all	No discomfort/pain
Convenient	Not embarrassed	Some discomfort/pain
Very convenient	Not embarrassed at all	No discomfort/pain
Very convenient	Not embarrassed	Some discomfort/pain
Very convenient	Not embarrassed at all	Neutral
Very convenient	Not embarrassed at all	Some discomfort/pain
Very convenient	Not embarrassed at all	No discomfort/pain
Convenient	Not embarrassed at all	Neutral
Not convenient	Embarrassed	Some discomfort/pain
Very convenient	Not embarrassed at all	Some discomfort/pain
Very convenient	Not embarrassed at all	Some discomfort/pain

Convenient	Not embarrassed at all	Some discomfort/pain
Convenient	Not embarrassed at all	Some discomfort/pain
Very convenient	Not embarrassed at all	Some discomfort/pain
Very convenient	Not embarrassed at all	No discomfort/pain at all
Convenient	Not embarrassed at all	Some discomfort/pain
Convenient	Not embarrassed	Some discomfort/pain
Very convenient	Not embarrassed at all	No discomfort/pain at all
Very convenient	Not embarrassed at all	Neutral
Convenient	Not embarrassed at all	Neutral
Convenient	Not embarrassed at all	No discomfort/pain
Very convenient	Not embarrassed at all	No discomfort/pain at all
Convenient	Not embarrassed at all	Some discomfort/pain
Convenient	Not embarrassed at all	Neutral
Convenient	Not embarrassed	Some discomfort/pain
Convenient	Not embarrassed at all	Some discomfort/pain
Very convenient	Not embarrassed at all	No discomfort/pain
Very convenient	Neutral	Neutral
Very convenient	Not embarrassed at all	Neutral
Convenient	Not embarrassed at all	Some discomfort/pain
Convenient	Not embarrassed at all	Some discomfort/pain
Very convenient	Not embarrassed at all	No discomfort/pain
Very convenient	Not embarrassed at all	Neutral
Very convenient	Not embarrassed at all	No discomfort/pain
Very convenient	Not embarrassed at all	No discomfort/pain at all
Convenient	Not embarrassed at all	No discomfort/pain at all
Very convenient	Not embarrassed at all	Neutral
Very convenient	Not embarrassed at all	Some discomfort/pain
Convenient	Not embarrassed at all	Neutral
Very convenient	Not embarrassed at all	No discomfort/pain
Very convenient	Not embarrassed at all	Neutral
Very convenient	Not embarrassed at all	Some discomfort/pain
Very convenient	Not embarrassed at all	Neutral
Convenient	Not embarrassed at all	Neutral

Very convenient	Not embarrassed at all	Neutral
Very convenient	Embarrassed	Some discomfort/pain
Convenient	Not embarrassed at all	Neutral
Convenient	Not embarrassed at all	Some discomfort/pain
Convenient	Not embarrassed at all	No discomfort/pain
Very convenient	Not embarrassed at all	Neutral
Very convenient	Not embarrassed at all	Some discomfort/pain
Convenient	Not embarrassed at all	No discomfort/pain
Convenient	Not embarrassed at all	Some discomfort/pain
Very convenient	Not embarrassed at all	No discomfort/pain
Convenient	Not embarrassed at all	Some discomfort/pain
Very convenient	Not embarrassed at all	No discomfort/pain at all
Very convenient	Not embarrassed at all	Some discomfort/pain
Very convenient	Not embarrassed at all	No discomfort/pain
Very convenient	Not embarrassed at all	No discomfort/pain
Convenient	Not embarrassed at all	Neutral
Convenient	Not embarrassed at all	Some discomfort/pain
Very convenient	Not embarrassed at all	No discomfort/pain at all
Very convenient	Not embarrassed at all	No discomfort/pain
Very convenient	Not embarrassed at all	Neutral
Convenient	Not embarrassed at all	Some discomfort/pain
Very convenient	Not embarrassed at all	Neutral
Convenient	Not embarrassed at all	Some discomfort/pain
Convenient	Not embarrassed at all	No discomfort/pain
Convenient	Neutral	Some discomfort/pain
Very convenient	Not embarrassed at all	Neutral
Very convenient	Not embarrassed at all	Some discomfort/pain
Very convenient	Not embarrassed at all	Some discomfort/pain
Convenient	Not embarrassed at all	Neutral
Convenient	Not embarrassed at all	Some discomfort/pain
Convenient	Not embarrassed	Neutral
Very convenient	Not embarrassed at all	No discomfort/pain
Convenient	Not embarrassed at all	Neutral

Convenient	Not embarrassed	Neutral
Convenient	Not embarrassed at all	Some discomfort/pain
Very convenient	Not embarrassed at all	No discomfort/pain at all
Convenient	Not embarrassed at all	No discomfort/pain at all
Very convenient	Not embarrassed at all	Some discomfort/pain
Convenient	Not embarrassed at all	No discomfort/pain
Very convenient	Embarrassed	Some discomfort/pain
Convenient	Not embarrassed at all	Some discomfort/pain
Very convenient	Not embarrassed at all	No discomfort/pain at all
Very convenient	Not embarrassed at all	Some discomfort/pain
Convenient	Not embarrassed at all	No discomfort/pain
Convenient	Not embarrassed at all	No discomfort/pain
Very convenient	Not embarrassed at all	Some discomfort/pain
Very convenient	Neutral	Some discomfort/pain
Convenient	Not embarrassed at all	No discomfort/pain at all
Convenient	Not embarrassed at all	Neutral
Convenient	Not embarrassed at all	Neutral
Convenient	Not embarrassed at all	Some discomfort/pain
Convenient	Not embarrassed at all	Neutral
Very convenient	Not embarrassed at all	Neutral
Very convenient	Not embarrassed at all	Neutral
Very convenient	Not embarrassed at all	Some discomfort/pain
Convenient	Not embarrassed at all	Some discomfort/pain
Convenient	Not embarrassed at all	No discomfort/pain
Convenient	Not embarrassed at all	No discomfort/pain
Very convenient	Not embarrassed at all	No discomfort/pain at all
Very convenient	Not embarrassed at all	Some discomfort/pain
Convenient	Not embarrassed at all	Some discomfort/pain
Very convenient	Not embarrassed at all	Neutral
Convenient	Not embarrassed at all	No discomfort/pain
Very convenient	Not embarrassed at all	Some discomfort/pain
Very convenient	Not embarrassed at all	No discomfort/pain
Convenient	Not embarrassed at all	Some discomfort/pain

Convenient	Not embarrassed	Neutral
Convenient	Not embarrassed at all	Neutral
Convenient	Not embarrassed at all	Some discomfort/pain
Convenient	Not embarrassed at all	Some discomfort/pain
Convenient	Not embarrassed at all	Some discomfort/pain
Convenient	Not embarrassed at all	No discomfort/pain
Very convenient	Not embarrassed	No discomfort/pain
Convenient	Not embarrassed	Some discomfort/pain
Very convenient	Not embarrassed at all	No discomfort/pain
Convenient	Not embarrassed	No discomfort/pain
Very convenient	Not embarrassed at all	No discomfort/pain at all
Convenient	Not embarrassed at all	No discomfort/pain
Very convenient	Not embarrassed at all	No discomfort/pain at all
Convenient	Not embarrassed at all	No discomfort/pain
Convenient	Not embarrassed at all	Neutral
Convenient	Neutral	Neutral
Very convenient	Not embarrassed at all	Neutral
Very convenient	Not embarrassed at all	Some discomfort/pain
Convenient	Not embarrassed	Neutral
Convenient	Not embarrassed at all	Neutral
Very convenient	Not embarrassed at all	No discomfort/pain
Convenient	Not embarrassed at all	Some discomfort/pain
Convenient	Not embarrassed at all	Some discomfort/pain
Very convenient	Not embarrassed at all	Neutral
Very convenient	Not embarrassed at all	No discomfort/pain at all
Convenient	Not embarrassed	Some discomfort/pain
Convenient	Not embarrassed	No discomfort/pain
Very convenient	Not embarrassed at all	No discomfort/pain
Very convenient	Not embarrassed at all	Some discomfort/pain
Very convenient	Not embarrassed at all	Neutral
Very convenient	Not embarrassed at all	No discomfort/pain at all
Convenient	Not embarrassed at all	No discomfort/pain
Very convenient	Not embarrassed at all	Neutral

Convenient	Not embarrassed at all	Neutral
Convenient	Not embarrassed at all	Neutral
Convenient	Not embarrassed at all	Neutral
Convenient	Not embarrassed at all	No discomfort/pain
Convenient	Not embarrassed	No discomfort/pain
Convenient	Not embarrassed at all	No discomfort/pain
Convenient	Not embarrassed at all	No discomfort/pain
Convenient	Not embarrassed at all	Some discomfort/pain
Convenient	Neutral	Some discomfort/pain
Very convenient	Not embarrassed at all	No discomfort/pain
Very convenient	Not embarrassed at all	Neutral
Convenient	Not embarrassed at all	No discomfort/pain
Convenient	Not embarrassed at all	Some discomfort/pain
Convenient	Embarrassed	Some discomfort/pain
Convenient	Not embarrassed at all	No discomfort/pain
Very convenient	Neutral	Some discomfort/pain
Convenient	Not embarrassed at all	Some discomfort/pain
Neutral	Not embarrassed	Some discomfort/pain
Convenient	Not embarrassed at all	Some discomfort/pain
Convenient	Embarrassed	Neutral
Convenient	Not embarrassed at all	Neutral
Very convenient	Not embarrassed at all	Neutral
Very convenient	Not embarrassed at all	Neutral
Very convenient	Not embarrassed at all	No discomfort/pain at all
Very convenient	Not embarrassed at all	No discomfort/pain
Convenient	Not embarrassed	Neutral
Very convenient	Not embarrassed at all	Neutral
Very convenient	Not embarrassed at all	No discomfort/pain
Convenient	Neutral	Some discomfort/pain
Convenient	Not embarrassed at all	No discomfort/pain
Very convenient	Not embarrassed at all	Neutral
Convenient	Not embarrassed	Neutral
Convenient	Not embarrassed at all	No discomfort/pain

Convenient	Not embarrassed at all	Some discomfort/pain
Convenient	Not embarrassed at all	Some discomfort/pain
Convenient	Not embarrassed	Some discomfort/pain
Very convenient	Not embarrassed at all	No discomfort/pain
Very convenient	Not embarrassed at all	No discomfort/pain
Convenient	Not embarrassed	Some discomfort/pain
Very convenient	Not embarrassed at all	Some discomfort/pain
Very convenient	Embarrassed	Neutral
Convenient	Not embarrassed	Some discomfort/pain
Very convenient	Not embarrassed at all	Some discomfort/pain
Convenient	Not embarrassed	Neutral
Convenient	Not embarrassed	Some discomfort/pain
Convenient	Not embarrassed	Some discomfort/pain
Convenient	Not embarrassed	Some discomfort/pain
Convenient	Not embarrassed	Some discomfort/pain
Very convenient	Not embarrassed at all	No discomfort/pain
Convenient	Not embarrassed	No discomfort/pain
Convenient	Not embarrassed at all	Neutral
Convenient	Not embarrassed	Some discomfort/pain
Very convenient	Not embarrassed at all	Neutral
Convenient	Not embarrassed	No discomfort/pain
Convenient	Not embarrassed	Some discomfort/pain
Neutral	Neutral	Neutral
Very convenient	Not embarrassed at all	Neutral
Very convenient	Not embarrassed	Neutral
Very convenient	Not embarrassed at all	No discomfort/pain at all
Convenient	Not embarrassed	No discomfort/pain
Convenient	Not embarrassed at all	Some discomfort/pain
Very convenient	Not embarrassed at all	No discomfort/pain at all
Very convenient	Embarrassed	Neutral
Convenient	Not embarrassed	Some discomfort/pain
Convenient	Not embarrassed	Some discomfort/pain
Very convenient	Not embarrassed at all	No discomfort/pain

Convenient	Not embarrassed at all	Some discomfort/pain
Convenient	Not embarrassed	No discomfort/pain
Convenient	Not embarrassed	Neutral
Convenient	Not embarrassed at all	Some discomfort/pain
Very convenient	Not embarrassed at all	No discomfort/pain
Convenient	Not embarrassed at all	Some discomfort/pain
Convenient	Not embarrassed	Some discomfort/pain
Convenient	Not embarrassed at all	No discomfort/pain
Convenient	Not embarrassed	Neutral
Convenient	Not embarrassed at all	No discomfort/pain
Convenient	Not embarrassed	No discomfort/pain
Convenient	Not embarrassed at all	Some discomfort/pain
Convenient	Not embarrassed at all	Some discomfort/pain
Convenient	Not embarrassed at all	Some discomfort/pain
Convenient	Not embarrassed	No discomfort/pain
Very convenient	Not embarrassed at all	Neutral
Very convenient	Not embarrassed at all	No discomfort/pain at all
Convenient	Not embarrassed	No discomfort/pain
Convenient	Not embarrassed at all	Neutral
Very convenient	Not embarrassed at all	No discomfort/pain
Convenient	Not embarrassed at all	No discomfort/pain
Convenient	Not embarrassed	Neutral
Convenient	Not embarrassed	Some discomfort/pain
Convenient	Not embarrassed	No discomfort/pain
Convenient	Not embarrassed	No discomfort/pain
Convenient	Not embarrassed	No discomfort/pain
Convenient	Not embarrassed	No discomfort/pain
Convenient	Not embarrassed	Neutral
Very convenient	Not embarrassed	No discomfort/pain
Convenient	Not embarrassed	Some discomfort/pain
Convenient	Not embarrassed	Some discomfort/pain
Convenient	Not embarrassed	No discomfort/pain
Very convenient	Not embarrassed at all	No discomfort/pain

Convenient	Neutral	Neutral
Convenient	Not embarrassed	No discomfort/pain
Very convenient	Not embarrassed at all	Neutral
Convenient	Not embarrassed at all	Some discomfort/pain
Convenient	Not embarrassed	Neutral
Convenient	Not embarrassed at all	Some discomfort/pain
Very convenient	Not embarrassed at all	Some discomfort/pain
Convenient	Not embarrassed	Neutral
Convenient	Not embarrassed at all	Some discomfort/pain
Convenient	Not embarrassed	No discomfort/pain
Convenient	Not embarrassed	Neutral
Convenient	Not embarrassed at all	Neutral
Convenient	Not embarrassed	Some discomfort/pain
Very convenient	Not embarrassed at all	No discomfort/pain
Very convenient	Not embarrassed at all	Some discomfort/pain
Convenient	Not embarrassed at all	No discomfort/pain
Very convenient	Not embarrassed at all	No discomfort/pain at all
Convenient	Not embarrassed	Neutral
Convenient	Not embarrassed at all	No discomfort/pain
Convenient	Not embarrassed at all	Neutral
Convenient	Not embarrassed	Some discomfort/pain
Convenient	Not embarrassed at all	Neutral
Convenient	Not embarrassed at all	Some discomfort/pain
Very convenient	Not embarrassed at all	No discomfort/pain
Convenient	Not embarrassed at all	Some discomfort/pain
Convenient	Not embarrassed at all	Some discomfort/pain
Convenient	Not embarrassed	Some discomfort/pain
Very convenient	Not embarrassed at all	Neutral
Convenient	Not embarrassed at all	No discomfort/pain
Very convenient	Not embarrassed at all	No discomfort/pain
Neutral	Not embarrassed at all	Some discomfort/pain
Very convenient	Not embarrassed at all	No discomfort/pain
Very convenient	Not embarrassed at all	No discomfort/pain

Convenient	Neutral	Neutral
Very convenient	Not embarrassed at all	No discomfort/pain
Convenient	Not embarrassed	No discomfort/pain
Convenient	Not embarrassed	No discomfort/pain
Very convenient	Not embarrassed at all	No discomfort/pain
Very convenient	Not embarrassed at all	Neutral
Very convenient	Not embarrassed at all	No discomfort/pain
Convenient	Not embarrassed	Some discomfort/pain
Convenient	Not embarrassed at all	No discomfort/pain
Very convenient	Not embarrassed at all	No discomfort/pain
Very convenient	Not embarrassed at all	No discomfort/pain at all
Convenient	Not embarrassed at all	Some discomfort/pain
Very convenient	Not embarrassed at all	Neutral
Convenient	Not embarrassed	No discomfort/pain
Convenient	Not embarrassed	Neutral
Very convenient	Not embarrassed at all	No discomfort/pain
Convenient	Not embarrassed	Some discomfort/pain
Convenient	Not embarrassed at all	Some discomfort/pain
Very convenient	Not embarrassed at all	No discomfort/pain at all
Convenient	Not embarrassed	No discomfort/pain
Convenient	Not embarrassed	Neutral
Convenient	Not embarrassed at all	Neutral
Convenient	Embarrassed	No discomfort/pain
Convenient	Not embarrassed at all	Some discomfort/pain
Convenient	Not embarrassed	Neutral
Convenient	Not embarrassed	Neutral
Very convenient	Not embarrassed at all	Some discomfort/pain
Very convenient	Not embarrassed at all	Some discomfort/pain
Very convenient	Not embarrassed at all	Some discomfort/pain
Convenient	Embarrassed	Some discomfort/pain
Convenient	Not embarrassed at all	Neutral
Neutral	Not embarrassed	Neutral
Convenient	Not embarrassed at all	No discomfort/pain

Convenient	Not embarrassed	No discomfort/pain
Convenient	Not embarrassed at all	Neutral
Convenient	Not embarrassed at all	Some discomfort/pain
Convenient	Neutral	Some discomfort/pain
Convenient	Neutral	Some discomfort/pain
Convenient	Not embarrassed	Some discomfort/pain
Convenient	Not embarrassed	No discomfort/pain
Convenient	Not embarrassed at all	Some discomfort/pain
Not convenient	Not embarrassed	Some discomfort/pain
Convenient	Not embarrassed	No discomfort/pain
Neutral	Not embarrassed	No discomfort/pain
Neutral	Not embarrassed at all	No discomfort/pain
Convenient	Not embarrassed at all	Neutral
Neutral	Not embarrassed at all	Some discomfort/pain
Convenient	Not embarrassed	No discomfort/pain
Convenient	Not embarrassed	No discomfort/pain
Convenient	Not embarrassed at all	Neutral
Very convenient	Not embarrassed at all	No discomfort/pain at all
Very convenient	Not embarrassed at all	Neutral
Very convenient	Not embarrassed at all	No discomfort/pain at all
Convenient	Not embarrassed	No discomfort/pain
Very convenient	Not embarrassed at all	No discomfort/pain
Very convenient	Not embarrassed at all	No discomfort/pain at all
Convenient	Not embarrassed at all	No discomfort/pain
Convenient	Not embarrassed at all	Some discomfort/pain
Very convenient	Not embarrassed at all	No discomfort/pain at all
Convenient	Not embarrassed	No discomfort/pain
Very convenient	Not embarrassed at all	No discomfort/pain
Convenient	Not embarrassed at all	Neutral
Convenient	Not embarrassed at all	Some discomfort/pain
Very convenient	Not embarrassed at all	No discomfort/pain at all
Convenient	Not embarrassed	Some discomfort/pain
Very convenient	Embarrassed	Some discomfort/pain

Convenient	Not embarrassed at all	Neutral
Very convenient	Not embarrassed at all	No discomfort/pain at all
Convenient	Embarrassed	Some discomfort/pain
Very convenient	Not embarrassed at all	No discomfort/pain
Convenient	Not embarrassed	Some discomfort/pain
Convenient	Not embarrassed	Neutral
Convenient	Not embarrassed at all	No discomfort/pain
Convenient	Not embarrassed at all	Some discomfort/pain
Convenient	Not embarrassed	Some discomfort/pain
Very convenient	Not embarrassed at all	Some discomfort/pain
Convenient	Not embarrassed at all	No discomfort/pain
Very convenient	Not embarrassed at all	Neutral
Very convenient	Not embarrassed at all	No discomfort/pain at all
Very convenient	Not embarrassed at all	No discomfort/pain at all
Very convenient	Not embarrassed at all	No discomfort/pain
Convenient	Not embarrassed	Some discomfort/pain
Very convenient	Not embarrassed at all	Neutral
Neutral	Not embarrassed at all	No discomfort/pain
Convenient	Not embarrassed at all	No discomfort/pain
Convenient	Not embarrassed	No discomfort/pain
Very convenient	Not embarrassed at all	Some discomfort/pain
Convenient	Not embarrassed	No discomfort/pain
Very convenient	Not embarrassed at all	No discomfort/pain
Neutral	Not embarrassed	Some discomfort/pain
Convenient	Not embarrassed at all	No discomfort/pain
Very convenient	Not embarrassed at all	No discomfort/pain at all
Very convenient	Not embarrassed at all	No discomfort/pain
Convenient	Not embarrassed	No discomfort/pain
Convenient	Not embarrassed at all	Some discomfort/pain
Very convenient	Not embarrassed at all	Some discomfort/pain
Very convenient	Not embarrassed at all	No discomfort/pain
Convenient	Not embarrassed at all	Neutral
Very convenient	Not embarrassed at all	Some discomfort/pain

Convenient	Not embarrassed at all	Neutral
Convenient	Not embarrassed	Some discomfort/pain
Very convenient	Not embarrassed at all	Some discomfort/pain
Convenient	Not embarrassed	No discomfort/pain
Convenient	Not embarrassed at all	No discomfort/pain
Convenient	Not embarrassed	Neutral
Convenient	Not embarrassed at all	No discomfort/pain
Very convenient	Not embarrassed at all	Some discomfort/pain
Very convenient	Not embarrassed at all	No discomfort/pain
Convenient	Not embarrassed at all	No discomfort/pain
Very convenient	Not embarrassed at all	No discomfort/pain at all
Convenient	Not embarrassed at all	No discomfort/pain
Convenient	Not embarrassed	No discomfort/pain
Neutral	Not embarrassed at all	Some discomfort/pain
Convenient	Not embarrassed	No discomfort/pain
Convenient	Not embarrassed at all	Some discomfort/pain
Very convenient	Not embarrassed at all	No discomfort/pain
Very convenient	Not embarrassed at all	No discomfort/pain
Convenient	Not embarrassed	Neutral
Convenient	Not embarrassed at all	Some discomfort/pain
Very convenient	Not embarrassed at all	No discomfort/pain
Very convenient	Not embarrassed at all	No discomfort/pain
Convenient	Not embarrassed at all	Some discomfort/pain
Very convenient	Not embarrassed at all	No discomfort/pain
Convenient	Not embarrassed at all	Neutral
Convenient	Not embarrassed at all	Some discomfort/pain
Very convenient	Neutral	No discomfort/pain
Very convenient	Not embarrassed at all	No discomfort/pain
Very convenient	Not embarrassed at all	No discomfort/pain at all
Convenient	Not embarrassed at all	Neutral
Very convenient	Not embarrassed at all	No discomfort/pain
Convenient	Not embarrassed	No discomfort/pain
Very convenient	Not embarrassed at all	Some discomfort/pain

Convenient	Not embarrassed at all	Some discomfort/pain
Convenient	Not embarrassed at all	Some discomfort/pain
Not convenient	Not embarrassed at all	Neutral
Very convenient	Embarrassed	Neutral
Convenient	Not embarrassed	No discomfort/pain
Very convenient	Not embarrassed at all	No discomfort/pain
Convenient	Not embarrassed at all	No discomfort/pain
Convenient	Not embarrassed at all	No discomfort/pain
Very convenient	Not embarrassed at all	No discomfort/pain
Very convenient	Not embarrassed at all	No discomfort/pain
Convenient	Not embarrassed	Neutral
Convenient	Not embarrassed at all	Some discomfort/pain
Convenient	Not embarrassed at all	Some discomfort/pain
Very convenient	Not embarrassed at all	Neutral
Convenient	Not embarrassed at all	No discomfort/pain
Very convenient	Not embarrassed at all	No discomfort/pain at all
Convenient	Not embarrassed at all	No discomfort/pain
Convenient	Neutral	No discomfort/pain
Very convenient	Not embarrassed at all	Neutral
Convenient	Not embarrassed at all	Some discomfort/pain
Convenient	Not embarrassed	No discomfort/pain
Convenient	Not embarrassed	Some discomfort/pain
Very convenient	Not embarrassed at all	No discomfort/pain
Convenient	Not embarrassed at all	Some discomfort/pain
Very convenient	Not embarrassed at all	Some discomfort/pain
Very convenient	Not embarrassed at all	Some discomfort/pain
Convenient	Not embarrassed at all	No discomfort/pain
Convenient	Not embarrassed at all	Neutral
Convenient	Not embarrassed at all	No discomfort/pain
Neutral	Not embarrassed	Neutral
Neutral	Neutral	Neutral
Convenient	Not embarrassed	No discomfort/pain
Convenient	Not embarrassed at all	Neutral

Convenient	Not embarrassed at all	Neutral
Convenient	Not embarrassed at all	Some discomfort/pain
Convenient	Not embarrassed	No discomfort/pain
Convenient	Not embarrassed at all	Some discomfort/pain
Convenient	Not embarrassed	No discomfort/pain
Very convenient	Not embarrassed at all	No discomfort/pain
Convenient	Not embarrassed	Some discomfort/pain
Convenient	Not embarrassed	Neutral
Convenient	Not embarrassed at all	Some discomfort/pain
Convenient	Not embarrassed at all	No discomfort/pain
Convenient	Not embarrassed	Neutral
Convenient	Not embarrassed at all	No discomfort/pain
Convenient	Not embarrassed	No discomfort/pain
Convenient	Not embarrassed at all	Some discomfort/pain
Convenient	Not embarrassed at all	Neutral
Convenient	Neutral	Some discomfort/pain
Convenient	Embarrassed	Some discomfort/pain
Convenient	Not embarrassed at all	Some discomfort/pain
Convenient	Not embarrassed	Neutral
Convenient	Not embarrassed at all	Neutral
Convenient	Not embarrassed at all	No discomfort/pain
Convenient	Neutral	Neutral
Convenient	Not embarrassed	No discomfort/pain
Convenient	Not embarrassed at all	Neutral
Very convenient	Not embarrassed at all	No discomfort/pain at all
Convenient	Not embarrassed	No discomfort/pain
Convenient	Not embarrassed at all	Some discomfort/pain
Convenient	Not embarrassed at all	No discomfort/pain
Convenient	Not embarrassed at all	No discomfort/pain
Convenient	Not embarrassed at all	No discomfort/pain
Convenient	Not embarrassed	No discomfort/pain
Very convenient	Not embarrassed at all	Neutral
Very convenient	Not embarrassed at all	No discomfort/pain at all

Convenient	Not embarrassed at all	No discomfort/pain
Convenient	Not embarrassed	Neutral
Convenient	Not embarrassed at all	Some discomfort/pain
Very convenient	Not embarrassed at all	No discomfort/pain
Convenient	Not embarrassed	No discomfort/pain
Convenient	Not embarrassed at all	Some discomfort/pain
Convenient	Not embarrassed at all	Some discomfort/pain
Convenient	Not embarrassed	No discomfort/pain
Convenient	Not embarrassed at all	Neutral
Very convenient	Not embarrassed at all	Neutral
Convenient	Not embarrassed at all	No discomfort/pain
Convenient	Not embarrassed	Neutral
Convenient	Not embarrassed	Some discomfort/pain
Convenient	Not embarrassed	No discomfort/pain
Convenient	Not embarrassed at all	Some discomfort/pain
Convenient	Not embarrassed at all	Neutral
Convenient	Neutral	Neutral
Convenient	Not embarrassed	No discomfort/pain
Convenient	Neutral	Some discomfort/pain
Convenient	Not embarrassed at all	No discomfort/pain
Very convenient	Not embarrassed at all	No discomfort/pain at all
Convenient	Not embarrassed	No discomfort/pain
Convenient	Not embarrassed	No discomfort/pain
Convenient	Not embarrassed at all	Neutral
Convenient	Not embarrassed	Some discomfort/pain
Convenient	Not embarrassed at all	No discomfort/pain
Convenient	Not embarrassed at all	Neutral
Convenient	Not embarrassed at all	Some discomfort/pain
Convenient	Not embarrassed	No discomfort/pain
Convenient	Not embarrassed at all	No discomfort/pain
Convenient	Not embarrassed at all	Neutral
Convenient	Not embarrassed	Neutral
Convenient	Not embarrassed	No discomfort/pain

Convenient	Not embarrassed	No discomfort/pain
Convenient	Not embarrassed at all	No discomfort/pain
Very convenient	Not embarrassed at all	Neutral
Neutral	Not embarrassed at all	No discomfort/pain
Convenient	Not embarrassed at all	Some discomfort/pain
Convenient	Neutral	Some discomfort/pain
Very convenient	Not embarrassed at all	No discomfort/pain at all
Convenient	Not embarrassed	Neutral
Convenient	Not embarrassed at all	Some discomfort/pain
Convenient	Not embarrassed	No discomfort/pain
Convenient	Not embarrassed	Neutral
Convenient	Not embarrassed	Neutral
Convenient	Not embarrassed	Some discomfort/pain
Convenient	Not embarrassed at all	Some discomfort/pain
Convenient	Not embarrassed	No discomfort/pain
Convenient	Not embarrassed at all	Neutral
Very convenient	Not embarrassed at all	No discomfort/pain
Convenient	Not embarrassed at all	Neutral
Very convenient	Not embarrassed at all	Neutral
Convenient	Not embarrassed at all	No discomfort/pain
Convenient	Neutral	Neutral
Very convenient	Not embarrassed at all	Some discomfort/pain
Very convenient	Not embarrassed at all	No discomfort/pain
Convenient	Not embarrassed at all	Some discomfort/pain
Convenient	Not embarrassed at all	Neutral
Very convenient	Not embarrassed at all	Neutral
Convenient	Not embarrassed	Neutral
Convenient	Neutral	Some discomfort/pain
Convenient	Not embarrassed at all	Some discomfort/pain
Convenient	Not embarrassed at all	No discomfort/pain
Convenient	Embarrassed	No discomfort/pain
Convenient	Not embarrassed at all	No discomfort/pain
Very convenient	Not embarrassed at all	Some discomfort/pain

Convenient	Not embarrassed	No discomfort/pain
Not convenient	Embarrassed	Some discomfort/pain
Convenient	Not embarrassed at all	No discomfort/pain
Convenient	Not embarrassed	Some discomfort/pain
Convenient	Not embarrassed	Neutral
Very convenient	Not embarrassed at all	No discomfort/pain
Convenient	Not embarrassed	Neutral
Very convenient	Not embarrassed at all	No discomfort/pain
Very convenient	Not embarrassed at all	Some discomfort/pain
Very convenient	Not embarrassed at all	No discomfort/pain at all
Very convenient	Not embarrassed at all	No discomfort/pain at all
Not convenient	Not embarrassed	Some discomfort/pain
Convenient	Not embarrassed	Neutral
Convenient	Not embarrassed at all	Neutral
Convenient	Not embarrassed	No discomfort/pain
Convenient	Not embarrassed	No discomfort/pain
Convenient	Not embarrassed	No discomfort/pain
Convenient	Not embarrassed	No discomfort/pain
Convenient	Not embarrassed	No discomfort/pain
Convenient	Not embarrassed	No discomfort/pain
Convenient	Not embarrassed at all	Neutral
Convenient	Not embarrassed	No discomfort/pain
Very convenient	Not embarrassed at all	No discomfort/pain
Convenient	Not embarrassed	No discomfort/pain
Convenient	Embarrassed	Some discomfort/pain
Neutral	Not embarrassed at all	Some discomfort/pain
Convenient	Not embarrassed	Some discomfort/pain
Convenient	Not embarrassed	Some discomfort/pain
Convenient	Not embarrassed at all	No discomfort/pain
Convenient	Not embarrassed at all	Some discomfort/pain
Convenient	Not embarrassed	No discomfort/pain
Convenient	Embarrassed	Some discomfort/pain
Convenient	Not embarrassed at all	No discomfort/pain

Very convenient	Not embarrassed at all	No discomfort/pain
Very convenient	Not embarrassed at all	No discomfort/pain at all
Convenient	Not embarrassed at all	No discomfort/pain
Convenient	Not embarrassed	Some discomfort/pain
Convenient	Not embarrassed at all	No discomfort/pain
Convenient	Not embarrassed at all	Neutral
Convenient	Not embarrassed at all	No discomfort/pain
Convenient	Not embarrassed at all	Some discomfort/pain
Very convenient	Not embarrassed at all	No discomfort/pain
Convenient	Not embarrassed at all	Some discomfort/pain
Convenient	Not embarrassed at all	No discomfort/pain
Convenient	Neutral	Some discomfort/pain
Convenient	Not embarrassed	Some discomfort/pain
Convenient	Not embarrassed at all	No discomfort/pain
Very convenient	Not embarrassed	Some discomfort/pain
Convenient	Not embarrassed at all	Some discomfort/pain
Very convenient	Not embarrassed at all	Neutral
Convenient	Not embarrassed	Neutral
Convenient	Not embarrassed	Neutral
Convenient	Not embarrassed	Some discomfort/pain
Convenient	Not embarrassed at all	Some discomfort/pain
Very convenient	Not embarrassed at all	No discomfort/pain
Convenient	Not embarrassed	No discomfort/pain
Convenient	Not embarrassed at all	Some discomfort/pain
Convenient	Not embarrassed	Neutral
Convenient	Not embarrassed at all	Some discomfort/pain
Neutral	Embarrassed	Neutral
Convenient	Not embarrassed	No discomfort/pain
Very convenient	Not embarrassed at all	No discomfort/pain at all
Convenient	Embarrassed	Some discomfort/pain
Not convenient	Embarrassed	Some discomfort/pain
Convenient	Not embarrassed	Some discomfort/pain
Convenient	Not embarrassed	Neutral

Convenient	Not embarrassed at all	Some discomfort/pain
Neutral	Neutral	Neutral
Convenient	Not embarrassed at all	Some discomfort/pain
Convenient	Not embarrassed	No discomfort/pain
Convenient	Not embarrassed	Neutral
Convenient	Not embarrassed	Neutral
Convenient	Not embarrassed	Some discomfort/pain
Neutral	Neutral	No discomfort/pain
Convenient	Not embarrassed	No discomfort/pain
Convenient	Not embarrassed	Neutral
Not convenient	Embarrassed	Some discomfort/pain
Very convenient	Not embarrassed at all	Some discomfort/pain
Neutral	Not embarrassed at all	Some discomfort/pain
Convenient	Not embarrassed	Some discomfort/pain
Neutral	Not embarrassed	Neutral
Convenient	Not embarrassed	Some discomfort/pain
Convenient	Not embarrassed at all	Neutral
Very convenient	Not embarrassed at all	No discomfort/pain
Convenient	Not embarrassed	No discomfort/pain
Convenient	Not embarrassed	Some discomfort/pain
Convenient	Not embarrassed at all	No discomfort/pain
Convenient	Not embarrassed	Some discomfort/pain
Convenient	Not embarrassed	Neutral
Convenient	Not embarrassed	Some discomfort/pain
Convenient	Not embarrassed at all	No discomfort/pain
Neutral	Not embarrassed	Some discomfort/pain
Convenient	Not embarrassed at all	Some discomfort/pain
Convenient	Not embarrassed	Neutral
Convenient	Not embarrassed	No discomfort/pain
Convenient	Not embarrassed	Some discomfort/pain
Very convenient	Not embarrassed at all	No discomfort/pain
Convenient	Not embarrassed	No discomfort/pain
Convenient	Not embarrassed	No discomfort/pain

Convenient	Not embarrassed at all	Neutral
Convenient	Not embarrassed	Neutral
Very convenient	Not embarrassed at all	No discomfort/pain at all
Convenient	Not embarrassed	Some discomfort/pain
Convenient	Not embarrassed	Some discomfort/pain
Convenient	Not embarrassed	No discomfort/pain
Convenient	Not embarrassed at all	No discomfort/pain at all
Convenient	Not embarrassed at all	No discomfort/pain at all
Convenient	Neutral	Neutral
Convenient	Not embarrassed	No discomfort/pain
Very convenient	Not embarrassed at all	No discomfort/pain
Convenient	Not embarrassed at all	No discomfort/pain
Convenient	Not embarrassed	Some discomfort/pain
Convenient	Not embarrassed	Some discomfort/pain
Convenient	Not embarrassed at all	Neutral
Convenient	Not embarrassed at all	Some discomfort/pain
Convenient	Not embarrassed	Some discomfort/pain
Very convenient	Not embarrassed at all	Some discomfort/pain
Convenient	Not embarrassed	No discomfort/pain
Convenient	Not embarrassed	No discomfort/pain
Convenient	Not embarrassed	Some discomfort/pain
Convenient	Not embarrassed at all	Some discomfort/pain
Convenient	Not embarrassed at all	No discomfort/pain
Convenient	Not embarrassed at all	Some discomfort/pain
Convenient	Not embarrassed at all	No discomfort/pain
Convenient	Not embarrassed at all	Some discomfort/pain
Convenient	Not embarrassed	Some discomfort/pain
Convenient	Not embarrassed at all	No discomfort/pain
Convenient	Embarrassed	No discomfort/pain
Neutral	Not embarrassed at all	Neutral
Very convenient	Not embarrassed	No discomfort/pain
Convenient	Not embarrassed	Neutral
Convenient	Not embarrassed	Some discomfort/pain

Very convenient	Not embarrassed at all	No discomfort/pain at all
Convenient	Not embarrassed at all	No discomfort/pain
Convenient	Not embarrassed	Neutral
Convenient	Not embarrassed	Some discomfort/pain
Convenient	Not embarrassed	No discomfort/pain
Convenient	Not embarrassed at all	Neutral
Convenient	Not embarrassed	No discomfort/pain
Neutral	Not embarrassed	Neutral
Very convenient	Not embarrassed at all	Some discomfort/pain
Convenient	Not embarrassed at all	Neutral
Convenient	Not embarrassed	Neutral
Convenient	Not embarrassed	Some discomfort/pain
Convenient	Not embarrassed	No discomfort/pain
Very convenient	Not embarrassed at all	No discomfort/pain at all
Convenient	Not embarrassed	Some discomfort/pain
Convenient	Not embarrassed	No discomfort/pain
Convenient	Not embarrassed at all	No discomfort/pain
Convenient	Not embarrassed at all	Neutral
Convenient	Not embarrassed	Neutral
Convenient	Embarrassed	No discomfort/pain
Convenient	Not embarrassed	Some discomfort/pain
Convenient	Not embarrassed at all	No discomfort/pain
Convenient	Not embarrassed at all	Some discomfort/pain
Convenient	Not embarrassed	Some discomfort/pain
Not convenient	Not embarrassed	Some discomfort/pain
Convenient	Not embarrassed at all	Some discomfort/pain
Convenient	Not embarrassed at all	No discomfort/pain
Convenient	Not embarrassed	Some discomfort/pain
Very convenient	Not embarrassed at all	Some discomfort/pain
Not convenient	Not embarrassed	No discomfort/pain
Convenient	Not embarrassed at all	Neutral
Convenient	Not embarrassed	Some discomfort/pain
Convenient	Not embarrassed at all	No discomfort/pain

Convenient	Not embarrassed	No discomfort/pain
Convenient	Not embarrassed at all	Neutral
Neutral	Not embarrassed	Some discomfort/pain
Convenient	Not embarrassed at all	Some discomfort/pain
Convenient	Not embarrassed	No discomfort/pain
Very convenient	Not embarrassed at all	No discomfort/pain at all
Neutral	Not embarrassed at all	Neutral
Convenient	Not embarrassed	No discomfort/pain
Very convenient	Not embarrassed at all	Neutral
Very convenient	Not embarrassed at all	Some discomfort/pain
Very convenient	Not embarrassed at all	Some discomfort/pain
Convenient	Not embarrassed	No discomfort/pain
Very convenient	Not embarrassed at all	Neutral
Convenient	Not embarrassed	Some discomfort/pain
Very convenient	Not embarrassed at all	No discomfort/pain at all
Very convenient	Not embarrassed at all	No discomfort/pain
Convenient	Not embarrassed	No discomfort/pain
Convenient	Not embarrassed	No discomfort/pain
Neutral	Not embarrassed	Neutral
Convenient	Not embarrassed	No discomfort/pain at all
Convenient	Not embarrassed	Some discomfort/pain
Convenient	Not embarrassed	No discomfort/pain
Convenient	Not embarrassed	No discomfort/pain
Convenient	Not embarrassed	Some discomfort/pain
Very convenient	Not embarrassed at all	No discomfort/pain
Very convenient	Not embarrassed at all	No discomfort/pain at all
Convenient	Not embarrassed	Neutral
Convenient	Not embarrassed at all	Some discomfort/pain
Convenient	Not embarrassed	Some discomfort/pain
Convenient	Not embarrassed	No discomfort/pain
Convenient	Not embarrassed	Neutral
Very convenient	Not embarrassed at all	Some discomfort/pain
Convenient	Not embarrassed	Neutral

Convenient	Not embarrassed at all	No discomfort/pain
Convenient	Not embarrassed	No discomfort/pain
Convenient	Not embarrassed	Neutral
Convenient	Not embarrassed at all	Neutral
Very convenient	Not embarrassed at all	No discomfort/pain
Convenient	Not embarrassed	Some discomfort/pain
Neutral	Not embarrassed	Some discomfort/pain
Convenient	Not embarrassed	Some discomfort/pain
Convenient	Not embarrassed	No discomfort/pain
Convenient	Not embarrassed	Some discomfort/pain
Convenient	Not embarrassed	No discomfort/pain
Convenient	Not embarrassed	No discomfort/pain
Convenient	Not embarrassed	No discomfort/pain
Convenient	Not embarrassed	Some discomfort/pain
Convenient	Not embarrassed	No discomfort/pain
Convenient	Not embarrassed	Neutral
Convenient	Not embarrassed	Neutral
Neutral	Not embarrassed	Some discomfort/pain
Convenient	Not embarrassed	No discomfort/pain
Convenient	Not embarrassed	Neutral
Convenient	Not embarrassed	Some discomfort/pain
Convenient	Not embarrassed	No discomfort/pain
Convenient	Embarrassed	Neutral
Convenient	Not embarrassed	Neutral
Convenient	Not embarrassed	No discomfort/pain
Convenient	Not embarrassed	Neutral
Convenient	Not embarrassed	No discomfort/pain
Convenient	Not embarrassed	Some discomfort/pain
Convenient	Not embarrassed	Some discomfort/pain
Convenient	Not embarrassed	No discomfort/pain
Neutral	Not embarrassed	No discomfort/pain
Convenient	Not embarrassed	Some discomfort/pain
Convenient	Not embarrassed at all	No discomfort/pain

Convenient	Not embarrassed	Neutral
Convenient	Not embarrassed	No discomfort/pain
Convenient	Not embarrassed	No discomfort/pain
Not convenient	Not embarrassed	Some discomfort/pain
Convenient	Not embarrassed	No discomfort/pain
Convenient	Not embarrassed	No discomfort/pain
Convenient	Not embarrassed	Some discomfort/pain
Convenient	Not embarrassed	Some discomfort/pain
Convenient	Not embarrassed	No discomfort/pain
Convenient	Not embarrassed	No discomfort/pain
Convenient	Not embarrassed	Some discomfort/pain
Very convenient	Not embarrassed	No discomfort/pain
Convenient	Not embarrassed	Neutral
Convenient	Not embarrassed	No discomfort/pain
Convenient	Not embarrassed	Some discomfort/pain
Convenient	Not embarrassed	Some discomfort/pain
Convenient	Not embarrassed	No discomfort/pain
Convenient	Not embarrassed	Some discomfort/pain
Not convenient	Not embarrassed	Some discomfort/pain
Convenient	Not embarrassed	Some discomfort/pain
Convenient	Not embarrassed	No discomfort/pain
Very convenient	Not embarrassed at all	No discomfort/pain
Convenient	Not embarrassed	No discomfort/pain
Convenient	Not embarrassed	No discomfort/pain
Convenient	Not embarrassed	No discomfort/pain
Convenient	Not embarrassed	Some discomfort/pain
Convenient	Not embarrassed	Some discomfort/pain
Neutral	Not embarrassed	Some discomfort/pain
Convenient	Embarrassed	No discomfort/pain
Convenient	Not embarrassed	No discomfort/pain
Convenient	Not embarrassed	Neutral
Convenient	Not embarrassed	No discomfort/pain
Convenient	Not embarrassed	Some discomfort/pain

Convenient	Not embarrassed	Some discomfort/pain
Convenient	Not embarrassed	Some discomfort/pain
Convenient	Not embarrassed	No discomfort/pain
Convenient	Not embarrassed	No discomfort/pain
Convenient	Not embarrassed	Some discomfort/pain
Convenient	Not embarrassed	Some discomfort/pain
Convenient	Not embarrassed	Some discomfort/pain
Convenient	Not embarrassed	Some discomfort/pain
Convenient	Not embarrassed	Some discomfort/pain
Convenient	Not embarrassed	No discomfort/pain
Convenient	Not embarrassed	No discomfort/pain
Not convenient	Not embarrassed	Some discomfort/pain
Convenient	Not embarrassed	No discomfort/pain
Convenient	Not embarrassed	Some discomfort/pain
Convenient	Not embarrassed	Some discomfort/pain
Convenient	Not embarrassed	No discomfort/pain
Convenient	Not embarrassed	No discomfort/pain
Convenient	Neutral	Some discomfort/pain
Convenient	Not embarrassed	Some discomfort/pain
Convenient	Not embarrassed	No discomfort/pain
Convenient	Not embarrassed	No discomfort/pain
Convenient	Not embarrassed	Neutral
Convenient	Not embarrassed	Some discomfort/pain
Convenient	Not embarrassed	Some discomfort/pain
Convenient	Not embarrassed	No discomfort/pain
Convenient	Not embarrassed	No discomfort/pain
Convenient	Not embarrassed	Neutral
Convenient	Not embarrassed	No discomfort/pain
Convenient	Not embarrassed	Some discomfort/pain
Convenient	Not embarrassed	No discomfort/pain
Convenient	Not embarrassed	Some discomfort/pain
Convenient	Not embarrassed	Some discomfort/pain
Not convenient	Embarrassed	Neutral

Convenient	Not embarrassed	Neutral
Convenient	Not embarrassed	Some discomfort/pain
Convenient	Not embarrassed	Some discomfort/pain
Convenient	Not embarrassed	No discomfort/pain
Convenient	Not embarrassed	Some discomfort/pain
Convenient	Not embarrassed	No discomfort/pain
Convenient	Not embarrassed	No discomfort/pain
Convenient	Not embarrassed	Some discomfort/pain
Convenient	Not embarrassed	No discomfort/pain
Convenient	Not embarrassed	Some discomfort/pain
Convenient	Not embarrassed	Some discomfort/pain
Convenient	Embarrassed	Some discomfort/pain
Convenient	Not embarrassed	Some discomfort/pain
Convenient	Not embarrassed	No discomfort/pain
Convenient	Not embarrassed	Neutral
Convenient	Not embarrassed	Neutral
Neutral	Not embarrassed	Some discomfort/pain
Neutral	Not embarrassed	Some discomfort/pain
Convenient	Embarrassed	No discomfort/pain
Convenient	Not embarrassed	Some discomfort/pain
Convenient	Embarrassed	Some discomfort/pain
Convenient	Not embarrassed	No discomfort/pain
Convenient	Not embarrassed	No discomfort/pain
Convenient	Not embarrassed	Neutral
Convenient	Not embarrassed	Some discomfort/pain
Not convenient	Not embarrassed	Some discomfort/pain
Convenient	Not embarrassed	Some discomfort/pain
Convenient	Not embarrassed	Some discomfort/pain
Convenient	Not embarrassed	Some discomfort/pain
Convenient	Not embarrassed	Neutral
Neutral	Not embarrassed	Some discomfort/pain
Not convenient	Not embarrassed	Neutral
Convenient	Not embarrassed	Some discomfort/pain

Convenient	Not embarrassed	Some discomfort/pain
Not convenient	Not embarrassed	Some discomfort/pain
Convenient	Not embarrassed	Some discomfort/pain
Convenient	Not embarrassed	Some discomfort/pain
Convenient	Not embarrassed	Some discomfort/pain
Convenient	Not embarrassed	No discomfort/pain
Convenient	Not embarrassed	Some discomfort/pain
Convenient	Not embarrassed	No discomfort/pain
Convenient	Not embarrassed	Some discomfort/pain
Convenient	Not embarrassed	No discomfort/pain
Not convenient	Not embarrassed	Some discomfort/pain
Very convenient	Not embarrassed at all	Neutral
Convenient	Not embarrassed	No discomfort/pain
Convenient	Not embarrassed	Some discomfort/pain
Not convenient	Not embarrassed	Some discomfort/pain
Convenient	Not embarrassed	Some discomfort/pain
Convenient	Not embarrassed	Some discomfort/pain
Convenient	Not embarrassed	Some discomfort/pain
Convenient	Not embarrassed	Some discomfort/pain
Convenient	Not embarrassed	No discomfort/pain
Convenient	Not embarrassed	Neutral
Convenient	Not embarrassed	Some discomfort/pain
Convenient	Not embarrassed	Some discomfort/pain
Convenient	Not embarrassed	No discomfort/pain
Convenient	Not embarrassed	No discomfort/pain
Neutral	Not embarrassed	Some discomfort/pain
Convenient	Not embarrassed	No discomfort/pain
Convenient	Not embarrassed	No discomfort/pain
Convenient	Neutral	Neutral
Convenient	Not embarrassed	No discomfort/pain
Neutral	Not embarrassed	No discomfort/pain
Convenient	Not embarrassed	Some discomfort/pain
Convenient	Not embarrassed	Some discomfort/pain

Convenient	Not embarrassed	No discomfort/pain
Convenient	Not embarrassed	No discomfort/pain
Convenient	Not embarrassed	Some discomfort/pain
Convenient	Not embarrassed	Some discomfort/pain
Convenient	Not embarrassed	No discomfort/pain
Convenient	Not embarrassed	Some discomfort/pain
Convenient	Not embarrassed	Some discomfort/pain
Convenient	Not embarrassed	No discomfort/pain
Convenient	Not embarrassed	No discomfort/pain
Convenient	Not embarrassed	Some discomfort/pain
Neutral	Not embarrassed	No discomfort/pain
Convenient	Not embarrassed	No discomfort/pain
Convenient	Not embarrassed	Some discomfort/pain
Convenient	Not embarrassed	No discomfort/pain
Convenient	Not embarrassed	No discomfort/pain
Convenient	Not embarrassed	Some discomfort/pain
Convenient	Not embarrassed	No discomfort/pain
Neutral	Not embarrassed	No discomfort/pain
Convenient	Not embarrassed	Some discomfort/pain
Convenient	Not embarrassed	Some discomfort/pain
Convenient	Not embarrassed	Some discomfort/pain
Convenient	Not embarrassed	No discomfort/pain
Convenient	Not embarrassed	No discomfort/pain
Convenient	Not embarrassed	No discomfort/pain
Convenient	Not embarrassed	No discomfort/pain
Convenient	Not embarrassed	Some discomfort/pain
Convenient	Not embarrassed	Neutral
Convenient	Not embarrassed	No discomfort/pain
Convenient	Not embarrassed	Some discomfort/pain
Convenient	Not embarrassed	Some discomfort/pain
Convenient	Not embarrassed	Some discomfort/pain
Convenient	Not embarrassed	Neutral
Convenient	Not embarrassed	Some discomfort/pain

Convenient	Not embarrassed	Some discomfort/pain
Convenient	Not embarrassed	No discomfort/pain
Convenient	Not embarrassed	No discomfort/pain
Convenient	Not embarrassed	Some discomfort/pain
Convenient	Not embarrassed	No discomfort/pain
Convenient	Not embarrassed	Some discomfort/pain
Convenient	Not embarrassed	No discomfort/pain
Very convenient	Not embarrassed	No discomfort/pain
Convenient	Not embarrassed	No discomfort/pain
Convenient	Not embarrassed	No discomfort/pain
Neutral	Not embarrassed	Some discomfort/pain
Not convenient	Not embarrassed	Severe discomfort/pain
Convenient	Not embarrassed	Some discomfort/pain
Convenient	Not embarrassed	Neutral
Neutral	Not embarrassed	Neutral
Convenient	Not embarrassed	No discomfort/pain
Convenient	Not embarrassed	Some discomfort/pain
Convenient	Not embarrassed	Some discomfort/pain
Convenient	Not embarrassed	No discomfort/pain
Convenient	Not embarrassed	Some discomfort/pain
Convenient	Not embarrassed	Some discomfort/pain
Convenient	Not embarrassed	No discomfort/pain
Convenient	Not embarrassed	Some discomfort/pain
Convenient	Not embarrassed	No discomfort/pain
Convenient	Not embarrassed	Some discomfort/pain
Convenient	Not embarrassed	Some discomfort/pain
Convenient	Not embarrassed	Neutral
Convenient	Not embarrassed	No discomfort/pain
Convenient	Not embarrassed	Neutral
Convenient	Not embarrassed	Some discomfort/pain
Convenient	Embarrassed	Some discomfort/pain
Convenient	Not embarrassed	No discomfort/pain
Convenient	Not embarrassed	No discomfort/pain

Convenient	Not embarrassed	Some discomfort/pain
Convenient	Not embarrassed	No discomfort/pain
Convenient	Not embarrassed	No discomfort/pain
Convenient	Not embarrassed	Some discomfort/pain
Convenient	Not embarrassed	Some discomfort/pain
Convenient	Not embarrassed	No discomfort/pain
Convenient	Not embarrassed	Some discomfort/pain
Convenient	Not embarrassed	Neutral
Convenient	Not embarrassed	Some discomfort/pain
Convenient	Not embarrassed	No discomfort/pain
Convenient	Not embarrassed	Some discomfort/pain
Convenient	Not embarrassed	No discomfort/pain
Convenient	Not embarrassed	No discomfort/pain
Convenient	Not embarrassed	No discomfort/pain
Convenient	Not embarrassed	Neutral
Convenient	Not embarrassed	Some discomfort/pain
Convenient	Not embarrassed	Some discomfort/pain
Convenient	Embarrassed	No discomfort/pain
Neutral	Not embarrassed	Some discomfort/pain
Convenient	Not embarrassed	Severe discomfort/pain

How confident are you that you can collect this self-samp	Are you willing to perform a self-sampling for HPV testing	Is the self-sampling successful?
Confident	Yes	Yes
Confident	Yes	Yes
Confident	Yes	Yes
Confident	Yes	Yes
Confident	Yes	Yes
Not confident	No	
Neutral	Yes	Yes
Confident	Yes	Yes
Neutral	Yes	Yes
Confident	Yes	Yes
Not confident	Yes	No
Confident	Yes	Yes
Neutral	Yes	Yes
Confident	Yes	Yes
Confident	Yes	Yes
Confident	Yes	Yes
Confident	Yes	Yes
Confident	Yes	Yes
Confident	Yes	Yes
Confident	Yes	Yes
Confident	Yes	Yes
Confident	Yes	Yes
Confident	Yes	Yes
Neutral	Yes	Yes
Confident	Yes	No
Confident	Yes	Yes
Confident	Yes	Yes
Confident	Yes	Yes
Not confident	Yes	Yes
Confident	Yes	Yes
Confident	Yes	Yes
Confident	Yes	Yes

Not confident	No	
Not confident	No	
Confident	Yes	Yes
Confident	Yes	Yes
Confident	Yes	Yes
Neutral	Yes	Yes
Confident	Yes	Yes
Confident	Yes	Yes
Confident	Yes	Yes
Confident	Yes	Yes
Confident	Yes	Yes
Not confident	Yes	Yes
Confident	Yes	Yes
Confident	Yes	Yes
Confident	Yes	Yes
Confident	Yes	Yes
Neutral	Yes	Yes
Confident	Yes	Yes
Neutral	Yes	Yes
Neutral	Yes	Yes
Confident	Yes	Yes
Confident	Yes	Yes
Confident	Yes	Yes
Confident	Yes	Yes
Confident	Yes	Yes
Confident	Yes	Yes
Confident	Yes	Yes
Not confident	No	
Very confident	Yes	Yes
Confident	Yes	Yes
Confident	Yes	Yes
Confident	Yes	Yes
Confident	Yes	Yes

Confident	Yes	Yes
Not confident	Yes	Yes
Neutral	Yes	Yes
Confident	Yes	Yes
Very confident	Yes	Yes
Confident	Yes	Yes
Confident	Yes	Yes
Not confident	Yes	Yes
Very confident	Yes	Yes
Confident	Yes	Yes
Confident	Yes	Yes
Not confident	Yes	Yes
Confident	Yes	Yes
Neutral	Yes	Yes
Confident	Yes	Yes
Confident	Yes	Yes
Confident	Yes	Yes
Confident	Yes	Yes
Not confident	Yes	Yes
Neutral	Yes	Yes
Confident	Yes	Yes
Neutral	Yes	Yes
Neutral	Yes	Yes
Confident	Yes	Yes
Confident	Yes	Yes
Confident	Yes	Yes
Confident	Yes	Yes
Confident	Yes	Yes
Neutral	Yes	Yes
Confident	Yes	Yes
Confident	Yes	Yes
Confident	Yes	Yes
Confident	Yes	Yes

Confident	Yes	Yes
Confident	Yes	Yes
Neutral	Yes	Yes
Confident	Yes	Yes
Confident	Yes	Yes
Confident	Yes	Yes
Neutral	Yes	Yes
Not confident	Yes	Yes
Confident	Yes	Yes
Confident	Yes	Yes
Not confident	Yes	Yes
Confident	Yes	Yes
Confident	Yes	Yes
Confident	Yes	Yes
Not confident	Yes	Yes
Not confident	Yes	Yes
Very confident	Yes	Yes
Not confident	Yes	Yes
Neutral	Yes	Yes
Not confident	Yes	Yes
Neutral	Yes	Yes
Neutral	Yes	Yes
Confident	Yes	Yes
Confident	Yes	Yes
Confident	Yes	Yes
Not confident	Yes	Yes
Confident	Yes	Yes
Neutral	Yes	Yes
Confident	Yes	Yes
Confident	Yes	Yes
Not confident	Yes	Yes
Confident	Yes	Yes
Confident	Yes	Yes

Confident	Yes	Yes
Confident	Yes	Yes
Confident	Yes	Yes
Confident	Yes	Yes
Confident	Yes	Yes
Not confident	Yes	Yes
Neutral	Yes	Yes
Neutral	Yes	Yes
Confident	Yes	Yes
Not confident	Yes	Yes
Not confident	Yes	Yes
Confident	Yes	Yes
Confident	Yes	Yes
Very confident	Yes	Yes
Confident	Yes	Yes
Confident	Yes	Yes
Neutral	Yes	Yes
Confident	Yes	Yes
Confident	Yes	Yes
Confident	Yes	Yes
Confident	Yes	Yes
Not confident	Yes	Yes
Confident	Yes	Yes
Confident	Yes	Yes
Neutral	Yes	Yes
Confident	Yes	Yes
Not confident	Yes	Yes
Confident	Yes	Yes
Confident	Yes	Yes
Not confident	Yes	Yes
Confident	Yes	Yes
Confident	Yes	Yes
Neutral	Yes	Yes

Confident	Yes	Yes
Confident	Yes	Yes
Confident	Yes	Yes
Neutral	Yes	Yes
Not confident	Yes	Yes
Confident	Yes	Yes
Confident	Yes	Yes
Confident	Yes	Yes
Neutral	Yes	Yes
Neutral	Yes	Yes
Neutral	Yes	Yes
Confident	Yes	Yes
Confident	Yes	Yes
Confident	Yes	Yes
Confident	Yes	Yes
Confident	Yes	Yes
Confident	Yes	Yes
Confident	Yes	Yes
Confident	Yes	Yes
Neutral	Yes	Yes
Not confident	Yes	Yes
Confident	Yes	Yes
Confident	Yes	Yes
Neutral	Yes	Yes
Confident	Yes	Yes
Not confident	Yes	Yes
Confident	Yes	Yes
Neutral	Yes	Yes
Confident	Yes	Yes
Confident	Yes	Yes
Confident	Yes	Yes
Neutral	Yes	Yes
Confident	Yes	Yes

Confident	Yes	Yes
Confident	Yes	Yes
Confident	Yes	Yes
Confident	Yes	Yes
Confident	Yes	Yes
Not confident	Yes	Yes
Confident	Yes	Yes
Confident	Yes	Yes
Neutral	Yes	Yes
Neutral	Yes	Yes
Confident	Yes	Yes
Neutral	Yes	Yes
Confident	Yes	Yes
Confident	Yes	Yes
Confident	Yes	Yes
Confident	Yes	Yes
Neutral	Yes	Yes
Confident	Yes	Yes
Neutral	Yes	Yes
Not confident	Yes	Yes
Confident	Yes	Yes
Not confident	Yes	Yes
Confident	Yes	Yes
Confident	Yes	Yes
Neutral	Yes	Yes
Very confident	Yes	Yes
Confident	Yes	Yes
Confident	Yes	Yes
Confident	Yes	Yes
Confident	Yes	Yes
Confident	Yes	Yes
Confident	Yes	Yes
Confident	Yes	Yes

Not confident	Yes	Yes
Confident	Yes	Yes
Not confident	Yes	Yes
Confident	Yes	Yes
Confident	Yes	Yes
Neutral	Yes	Yes
Not confident	Yes	Yes
Not confident	Yes	Yes
Confident	Yes	Yes
Confident	Yes	Yes
Neutral	Yes	Yes
Confident	Yes	Yes
Confident	Yes	Yes
Confident	Yes	Yes
Confident	Yes	Yes
Confident	Yes	Yes
Confident	Yes	Yes
Confident	Yes	Yes
Confident	Yes	Yes
Neutral	Yes	Yes
Confident	Yes	Yes
Neutral	Yes	Yes
Confident	Yes	Yes
Confident	Yes	Yes
Confident	Yes	Yes
Confident	Yes	Yes
Not confident	Yes	Yes
Confident	Yes	Yes
Confident	Yes	Yes
Confident	Yes	Yes
Confident	Yes	Yes
Neutral	Yes	Yes
Very confident	Yes	Yes

Confident	Yes	Yes
Neutral	Yes	Yes
Confident	Yes	Yes
Confident	Yes	Yes
Confident	Yes	Yes
Confident	Yes	Yes
Confident	Yes	Yes
Confident	Yes	Yes
Confident	Yes	Yes
Confident	Yes	Yes
Not confident	Yes	Yes
Not confident	Yes	Yes
Confident	Yes	Yes
Confident	Yes	Yes
Not confident	Yes	Yes
Confident	Yes	Yes
Neutral	Yes	Yes
Confident	Yes	Yes
Confident	Yes	Yes
Confident	Yes	Yes
Not confident	Yes	Yes
Not confident	Yes	Yes
Confident	Yes	Yes
Confident	Yes	Yes
Not confident	Yes	Yes
Confident	Yes	Yes
Confident	Yes	Yes
Neutral	Yes	Yes
Confident	Yes	Yes
Not confident	Yes	Yes
Confident	Yes	Yes
Confident	Yes	Yes
Confident	Yes	Yes

Confident	Yes	Yes
Confident	Yes	Yes
Confident	Yes	Yes
Confident	Yes	Yes
Not confident	Yes	Yes
Confident	Yes	Yes
Confident	Yes	Yes
Confident	Yes	Yes
Confident	Yes	Yes
Confident	Yes	Yes
Confident	Yes	Yes
Not confident	Yes	Yes
Confident	Yes	Yes
Very confident	Yes	Yes
Confident	Yes	Yes
Confident	Yes	Yes
Confident	Yes	Yes
Confident	Yes	Yes
Confident	Yes	Yes
Neutral	Yes	Yes
Very confident	Yes	Yes
Confident	Yes	Yes
Confident	Yes	Yes
Neutral	Yes	Yes
Confident	Yes	Yes
Not confident	Yes	Yes
Confident	Yes	Yes
Confident	Yes	Yes
Confident	Yes	Yes
Confident	Yes	Yes
Confident	Yes	Yes
Confident	Yes	Yes
Neutral	Yes	Yes

Neutral	Yes	Yes
Confident	Yes	Yes
Confident	Yes	Yes
Confident	Yes	Yes
Confident	Yes	Yes
Confident	Yes	Yes
Confident	Yes	Yes
Confident	Yes	Yes
Confident	Yes	Yes
Confident	Yes	Yes
Not confident	Yes	Yes
Confident	Yes	Yes
Confident	Yes	Yes
Confident	Yes	Yes
Neutral	Yes	Yes
Confident	Yes	Yes
Confident	Yes	Yes
Neutral	Yes	Yes
Neutral	Yes	Yes
Confident	Yes	Yes
Confident	Yes	Yes
Very confident	Yes	Yes
Confident	Yes	Yes
Confident	Yes	Yes
Not confident	Yes	Yes
Not confident	Yes	Yes
Neutral	Yes	Yes
Not confident	Yes	Yes
Confident	Yes	Yes
Not confident	Yes	Yes
Confident	Yes	Yes
Confident	Yes	Yes
Confident	Yes	Yes

Confident	Yes	Yes
Confident	Yes	Yes
Confident	Yes	Yes
Confident	Yes	Yes
Confident	Yes	Yes
Confident	Yes	Yes
Confident	Yes	Yes
Confident	Yes	Yes
Confident	Yes	Yes
Neutral	Yes	Yes
Not confident at all	Yes	Yes
Neutral	Yes	Yes
Confident	Yes	Yes
Neutral	Yes	Yes
Confident	Yes	Yes
Not confident	Yes	Yes
Confident	Yes	Yes
Neutral	Yes	Yes
Neutral	Yes	Yes
Not confident	Yes	Yes
Neutral	Yes	Yes
Not confident	Yes	Yes
Neutral	Yes	Yes
Neutral	Yes	Yes
Confident	Yes	Yes
Neutral	Yes	Yes
Not confident	Yes	Yes
Neutral	Yes	Yes
Confident	Yes	Yes
Confident	Yes	No
Confident	Yes	Yes
Confident	Yes	Yes
Confident	Yes	Yes

Confident	Yes	Yes
Confident	Yes	Yes
Very confident	Yes	Yes
Neutral	Yes	Yes
Confident	Yes	Yes
Not confident	Yes	Yes
Very confident	Yes	Yes
Confident	Yes	Yes
Confident	Yes	Yes
Neutral	Yes	Yes
Confident	Yes	Yes
Confident	Yes	Yes
Confident	Yes	Yes
Neutral	Yes	Yes
Confident	Yes	Yes
Not confident	Yes	Yes
Confident	Yes	Yes
Confident	Yes	Yes
Confident	Yes	Yes
Confident	Yes	Yes
Not confident	Yes	Yes
Very confident	Yes	Yes
Confident	Yes	Yes
Confident	Yes	Yes
Confident	Yes	Yes
Neutral	Yes	Yes
Neutral	Yes	Yes
Confident	Yes	Yes
Confident	Yes	Yes
Neutral	Yes	Yes
Confident	Yes	Yes
Very confident	Yes	Yes
Neutral	Yes	Yes

Very confident	Yes	Yes
Confident	Yes	Yes
Confident	Yes	Yes
Confident	Yes	Yes
Confident	Yes	Yes
Neutral	Yes	Yes
Confident	Yes	Yes
Not confident	Yes	Yes
Neutral	Yes	Yes
Confident	Yes	Yes
Neutral	Yes	Yes
Very confident	Yes	Yes
Confident	Yes	Yes
Confident	Yes	Yes
Confident	Yes	Yes
Confident	Yes	Yes
Very confident	Yes	Yes
Neutral	Yes	Yes
Neutral	Yes	Yes
Confident	Yes	Yes
Neutral	Yes	Yes
Very confident	Yes	Yes
Not confident	Yes	Yes
Confident	Yes	Yes
Confident	Yes	Yes
Confident	Yes	Yes
Confident	Yes	Yes
Confident	Yes	Yes
Very confident	Yes	Yes
Confident	Yes	Yes
Very confident	Yes	Yes
Very confident	Yes	Yes
Confident	Yes	Yes

Confident	Yes	Yes
Confident	Yes	Yes
Confident	Yes	Yes
Very confident	Yes	Yes
Confident	Yes	Yes
Very confident	Yes	Yes
Neutral	Yes	Yes
Confident	Yes	Yes
Neutral	Yes	Yes
Confident	Yes	Yes
Neutral	Yes	Yes
Confident	Yes	Yes
Confident	Yes	Yes
Confident	Yes	Yes
Confident	Yes	Yes
Confident	Yes	Yes
Not confident	Yes	Yes
Confident	Yes	Yes
Confident	Yes	Yes
Neutral	Yes	Yes
Not confident	Yes	Yes
Neutral	Yes	Yes
Confident	Yes	Yes
Neutral	Yes	Yes
Neutral	Yes	Yes
Neutral	Yes	Yes
Confident	Yes	Yes
Confident	Yes	Yes
Confident	Yes	Yes
Confident	Yes	Yes
Confident	Yes	Yes
Confident	Yes	Yes
Confident	Yes	Yes

Very confident	Yes	Yes
Confident	Yes	Yes
Confident	Yes	Yes
Very confident	Yes	Yes
Confident	Yes	Yes
Confident	Yes	Yes
Confident	Yes	Yes
Confident	Yes	Yes
Confident	Yes	Yes
Not confident	Yes	Yes
Confident	Yes	Yes
Confident	Yes	Yes
Confident	Yes	Yes
Neutral	Yes	Yes
Confident	Yes	Yes
Neutral	Yes	Yes
Confident	Yes	Yes
Neutral	Yes	Yes
Neutral	Yes	Yes
Very confident	Yes	Yes
Not confident	Yes	Yes
Confident	Yes	Yes
Confident	Yes	Yes
Very confident	Yes	Yes
Very confident	Yes	Yes
Confident	Yes	Yes
Confident	Yes	Yes
Confident	Yes	Yes
Confident	Yes	Yes
Confident	Yes	Yes
Confident	Yes	Yes
Neutral	Yes	Yes
Neutral	Yes	Yes

Confident	Yes	Yes
Confident	Yes	Yes
Confident	Yes	Yes
Confident	Yes	Yes
Very confident	Yes	Yes
Confident	Yes	Yes
Confident	Yes	Yes
Neutral	Yes	Yes
Confident	Yes	Yes
Confident	Yes	Yes
Confident	Yes	Yes
Neutral	Yes	Yes
Confident	Yes	Yes
Confident	Yes	Yes
Confident	Yes	Yes
Very confident	Yes	Yes
Confident	Yes	Yes
Neutral	Yes	Yes
Very confident	Yes	Yes
Confident	Yes	Yes
Confident	Yes	Yes
Not confident	Yes	Yes
Confident	Yes	Yes
Neutral	Yes	Yes
Confident	Yes	Yes
Neutral	Yes	Yes
Very confident	Yes	Yes
Confident	Yes	Yes
Neutral	Yes	Yes
Confident	Yes	Yes
Very confident	Yes	Yes
Confident	Yes	Yes
Confident	Yes	Yes

Neutral	Yes	Yes
Very confident	Yes	Yes
Confident	Yes	Yes
Confident	Yes	Yes
Confident	Yes	Yes
Neutral	Yes	Yes
Confident	Yes	Yes
Confident	Yes	Yes
Confident	Yes	Yes
Confident	Yes	Yes
Very confident	Yes	Yes
Neutral	Yes	Yes
Confident	Yes	Yes
Confident	Yes	Yes
Confident	Yes	Yes
Confident	Yes	Yes
Confident	Yes	Yes
Confident	Yes	Yes
Confident	Yes	Yes
Confident	Yes	Yes
Confident	Yes	Yes
Not confident	Yes	Yes
Very confident	Yes	Yes
Confident	Yes	Yes
Not confident	Yes	Yes
Not confident	Yes	Yes
Confident	Yes	Yes
Confident	Yes	Yes
Confident	Yes	Yes
Confident	Yes	Yes
Neutral	Yes	Yes
Confident	Yes	Yes
Confident	Yes	Yes

Confident	Yes	Yes
Confident	Yes	Yes
Confident	Yes	Yes
Confident	Yes	Yes
Confident	Yes	Yes
Not confident	Yes	Yes
Confident	Yes	Yes
Confident	Yes	Yes
Confident	Yes	Yes
Confident	Yes	Yes
Neutral	Yes	Yes
Neutral	Yes	Yes
Neutral	Yes	Yes
Confident	Yes	Yes
Confident	Yes	Yes
Not confident	Yes	Yes
Confident	Yes	Yes
Confident	Yes	Yes
Confident	Yes	Yes
Neutral	Yes	Yes
Confident	Yes	Yes
Confident	Yes	Yes
Confident	Yes	Yes
Neutral	Yes	Yes
Neutral	Yes	Yes
Not confident	Yes	Yes
Neutral	Yes	Yes
Confident	Yes	Yes
Confident	Yes	Yes
Confident	Yes	Yes
Not confident	Yes	Yes
Confident	Yes	Yes
Very confident	Yes	Yes

Neutral	Yes	Yes
Neutral	Yes	Yes
Confident	Yes	Yes
Very confident	Yes	Yes
Confident	Yes	Yes
Not confident	Yes	Yes
Very confident	Yes	Yes
Confident	Yes	Yes
Confident	Yes	Yes
Confident	Yes	Yes
Confident	Yes	Yes
Neutral	Yes	Yes
Confident	Yes	Yes
Neutral	Yes	Yes
Not confident	Yes	Yes
Confident	Yes	Yes
Neutral	Yes	Yes
Confident	Yes	Yes
Confident	Yes	Yes
Confident	Yes	Yes
Confident	Yes	Yes
Confident	Yes	Yes
Confident	Yes	Yes
Very confident	Yes	Yes
Very confident	Yes	Yes
Confident	Yes	Yes
Confident	Yes	Yes
Confident	Yes	Yes
Not confident	Yes	Yes
Not confident	Yes	Yes
Confident	Yes	Yes
Confident	Yes	Yes
Neutral	Yes	Yes

Confident	Yes	Yes
Not confident	Yes	Yes
Confident	Yes	Yes
Confident	Yes	Yes
Confident	Yes	Yes
Confident	Yes	Yes
Confident	Yes	Yes
Confident	Yes	Yes
Not confident	Yes	No
Confident	Yes	Yes
Confident	Yes	Yes
Confident	Yes	Yes
Confident	Yes	Yes
Confident	Yes	No
Confident	Yes	Yes
Not confident	Yes	Yes
Confident	Yes	Yes
Confident	Yes	Yes
Confident	Yes	Yes
Very confident	Yes	Yes
Confident	Yes	Yes
Confident	Yes	Yes
Confident	Yes	Yes
Confident	Yes	Yes
Not confident	Yes	Yes
Confident	Yes	Yes
Very confident	Yes	Yes
Confident	Yes	Yes
Confident	Yes	Yes
Neutral	Yes	Yes
Not confident	Yes	Yes
Confident	Yes	Yes
Confident	Yes	Yes

Neutral	Yes	Yes
Confident	Yes	Yes
Very confident	Yes	Yes
Confident	Yes	Yes
Neutral	Yes	Yes
Neutral	Yes	Yes
Not confident	Yes	Yes
Neutral	Yes	Yes
Confident	Yes	Yes
Confident	Yes	Yes
Neutral	Yes	Yes
Confident	Yes	Yes
Neutral	Yes	Yes
Neutral	Yes	Yes
Confident	Yes	Yes
Confident	Yes	Yes
Confident	Yes	Yes
Confident	Yes	Yes
Confident	Yes	Yes
Confident	Yes	Yes
Neutral	Yes	Yes
Confident	Yes	Yes
Confident	Yes	Yes
Confident	Yes	Yes
Confident	Yes	Yes
Confident	Yes	Yes
Confident	Yes	Yes
Not confident	Yes	Yes
Neutral	Yes	Yes
Confident	Yes	Yes
Confident	Yes	Yes
Confident	Yes	Yes
Neutral	Yes	Yes

Neutral	Yes	Yes
Confident	Yes	Yes
Not confident	Yes	Yes
Neutral	Yes	Yes
Confident	Yes	Yes
Neutral	Yes	Yes
Neutral	Yes	Yes
Confident	Yes	Yes
Confident	Yes	Yes
Confident	Yes	Yes
Confident	Yes	Yes
Confident	Yes	Yes
Very confident	Yes	Yes
Very confident	Yes	Yes
Confident	Yes	Yes
Confident	Yes	Yes
Confident	Yes	Yes
Confident	Yes	Yes
Confident	Yes	Yes
Neutral	Yes	Yes
Very confident	Yes	Yes
Confident	Yes	Yes
Not confident	Yes	Yes
Confident	Yes	Yes
Very confident	Yes	Yes
Confident	Yes	Yes
Confident	Yes	Yes
Confident	Yes	Yes
Confident	Yes	Yes
Neutral	Yes	Yes
Confident	Yes	Yes
Confident	Yes	Yes
Confident	Yes	Yes

Neutral	Yes	Yes
Confident	Yes	Yes
Neutral	Yes	Yes
Confident	Yes	Yes
Confident	Yes	Yes
Confident	Yes	Yes
Confident	Yes	Yes
Confident	Yes	Yes
Neutral	Yes	Yes
Confident	Yes	Yes
Confident	Yes	Yes
Neutral	Yes	Yes
Confident	Yes	Yes
Neutral	Yes	Yes
Confident	Yes	Yes
Neutral	Yes	Yes
Neutral	Yes	Yes
Not confident	Yes	Yes
Very confident	Yes	Yes
Confident	Yes	Yes
Confident	Yes	Yes
Confident	Yes	Yes
Confident	Yes	Yes
Confident	Yes	Yes
Confident	Yes	Yes
Confident	Yes	Yes
Confident	Yes	Yes
Confident	Yes	Yes
Confident	Yes	Yes
Confident	Yes	Yes
Neutral	Yes	Yes
Confident	Yes	Yes
Confident	Yes	Yes

Confident	Yes	Yes
Neutral	Yes	Yes
Not confident	Yes	Yes
Very confident	Yes	Yes
Very confident	Yes	Yes
Neutral	Yes	No
Neutral	Yes	Yes
Confident	Yes	Yes
Neutral	Yes	Yes
Confident	Yes	Yes
Confident	Yes	Yes
Confident	Yes	Yes
Not confident	Yes	Yes
Confident	Yes	Yes
Confident	Yes	Yes
Confident	Yes	Yes
Confident	Yes	Yes
Neutral	Yes	Yes
Confident	Yes	Yes
Confident	Yes	Yes
Confident	Yes	Yes
Not confident	Yes	Yes
Neutral	Yes	Yes
Very confident	Yes	Yes
Confident	Yes	Yes
Confident	Yes	Yes
Confident	Yes	Yes
Not confident	Yes	Yes
Confident	Yes	Yes
Confident	Yes	Yes
Neutral	Yes	Yes
Neutral	Yes	Yes
Neutral	Yes	Yes

Confident	Yes	Yes
Confident	Yes	Yes
Confident	Yes	Yes
Neutral	Yes	Yes
Confident	Yes	Yes
Very confident	Yes	Yes
Confident	Yes	Yes
Confident	Yes	Yes
Confident	Yes	Yes
Confident	Yes	Yes
Confident	Yes	Yes
Neutral	Yes	Yes
Neutral	Yes	Yes
Confident	Yes	Yes
Confident	Yes	Yes
Confident	Yes	Yes
Confident	Yes	Yes
Confident	Yes	Yes
Confident	Yes	Yes
Confident	Yes	Yes
Confident	Yes	Yes
Not confident	Yes	Yes
Neutral	Yes	Yes
Confident	Yes	Yes
Confident	Yes	Yes
Confident	Yes	Yes
Confident	Yes	Yes
Confident	Yes	Yes
Confident	Yes	Yes
Neutral	Yes	Yes
Not confident	Yes	Yes
Confident	Yes	Yes
Confident	Yes	Yes

Confident	Yes	Yes
Neutral	Yes	Yes
Confident	Yes	Yes
Neutral	Yes	Yes
Neutral	Yes	Yes
Confident	Yes	Yes
Very confident	Yes	Yes
Confident	Yes	Yes
Confident	Yes	Yes
Confident	Yes	Yes
Confident	Yes	Yes
Confident	Yes	Yes
Confident	Yes	Yes
Confident	Yes	Yes
Neutral	Yes	Yes
Confident	Yes	Yes
Very confident	Yes	Yes
Confident	Yes	Yes
Confident	Yes	Yes
Neutral	Yes	Yes
Neutral	Yes	Yes
Confident	Yes	Yes
Confident	Yes	Yes
Confident	Yes	Yes
Confident	Yes	No
Not confident	Yes	Yes
Neutral	Yes	Yes
Confident	Yes	Yes
Confident	Yes	Yes
Very confident	Yes	Yes
Neutral	Yes	Yes
Confident	Yes	Yes
Confident	Yes	Yes

Neutral	Yes	Yes
Confident	Yes	Yes
Not confident	Yes	Yes
Confident	Yes	Yes
Very confident	Yes	Yes
Confident	Yes	Yes
Confident	Yes	Yes
Confident	Yes	Yes
Confident	Yes	Yes
Confident	Yes	Yes
Very confident	Yes	Yes
Neutral	Yes	Yes
Confident	Yes	Yes
Confident	Yes	Yes
Confident	Yes	Yes
Confident	Yes	Yes
Neutral	Yes	Yes
Not confident	Yes	Yes
Confident	Yes	Yes
Confident	Yes	Yes
Not confident	Yes	Yes
Confident	Yes	Yes
Confident	Yes	Yes
Confident	Yes	Yes
Confident	Yes	Yes
Confident	Yes	Yes
Confident	Yes	Yes
Confident	Yes	Yes
Confident	Yes	Yes
Neutral	Yes	Yes
Confident	Yes	Yes
Confident	Yes	Yes
Confident	Yes	Yes

Confident	Yes	Yes
Confident	Yes	Yes
Confident	Yes	Yes
Neutral	Yes	Yes
Neutral	Yes	Yes
Confident	Yes	Yes
Confident	Yes	Yes
Confident	Yes	No
Not confident	Yes	Yes
Not confident	Yes	Yes
Confident	Yes	Yes
Confident	Yes	Yes
Neutral	Yes	Yes
Not confident	Yes	Yes
Confident	Yes	Yes
Confident	Yes	Yes
Confident	Yes	Yes
Very confident	Yes	Yes
Confident	Yes	Yes
Very confident	Yes	Yes
Confident	Yes	Yes
Confident	Yes	Yes
Very confident	Yes	Yes
Confident	Yes	Yes
Confident	Yes	Yes
Very confident	Yes	Yes
Confident	Yes	Yes
Confident	Yes	Yes
Neutral	Yes	Yes
Confident	Yes	Yes
Confident	Yes	Yes
Not confident	No	
Neutral	Yes	Yes

Confident	Yes	Yes
Very confident	Yes	Yes
Not confident	Yes	Yes
Confident	Yes	Yes
Confident	Yes	Yes
Neutral	Yes	Yes
Not confident	Yes	Yes
Neutral	Yes	Yes
Confident	Yes	Yes
Confident	Yes	Yes
Neutral	Yes	Yes
Confident	Yes	Yes
Confident	Yes	Yes
Very confident	Yes	Yes
Confident	Yes	Yes
Neutral	No	
Confident	Yes	Yes
Confident	Yes	Yes
Confident	Yes	Yes
Confident	Yes	Yes
Confident	Yes	Yes
Not confident	Yes	Yes
Confident	Yes	Yes
Neutral	Yes	Yes
Confident	Yes	Yes
Very confident	Yes	Yes
Confident	Yes	Yes
Confident	Yes	Yes
Confident	Yes	Yes
Confident	Yes	Yes
Confident	Yes	Yes
Confident	Yes	Yes
Confident	Yes	Yes

Confident	Yes	Yes
Not confident	Yes	Yes
Confident	Yes	Yes
Confident	Yes	Yes
Confident	Yes	Yes
Confident	Yes	Yes
Neutral	Yes	Yes
Confident	Yes	Yes
Confident	Yes	Yes
Confident	Yes	Yes
Confident	Yes	Yes
Confident	Yes	Yes
Confident	Yes	Yes
Neutral	Yes	Yes
Confident	Yes	Yes
Not confident	Yes	Yes
Very confident	Yes	Yes
Neutral	Yes	Yes
Confident	Yes	Yes
Confident	Yes	Yes
Confident	Yes	Yes
Confident	Yes	Yes
Confident	Yes	Yes
Confident	Yes	Yes
Neutral	Yes	Yes
Confident	Yes	Yes
Confident	Yes	Yes
Confident	Yes	Yes
Confident	Yes	Yes
Neutral	Yes	Yes
Confident	Yes	Yes
Confident	Yes	Yes
Confident	Yes	Yes

Confident	Yes	Yes
Confident	Yes	Yes
Neutral	Yes	No
Confident	Yes	Yes
Neutral	Yes	Yes
Confident	Yes	Yes
Confident	Yes	Yes
Confident	Yes	Yes
Confident	Yes	Yes
Confident	Yes	Yes
Confident	Yes	Yes
Neutral	Yes	Yes
Not confident	Yes	Yes
Confident	Yes	Yes
Confident	Yes	Yes
Confident	Yes	Yes
Neutral	Yes	Yes
Confident	Yes	Yes
Confident	Yes	Yes
Confident	Yes	Yes
Confident	Yes	Yes
Not confident	Yes	Yes
Confident	Yes	Yes
Confident	Yes	Yes
Confident	Yes	Yes
Confident	Yes	Yes
Neutral	Yes	Yes
Confident	Yes	Yes
Confident	Yes	Yes
Neutral	Yes	Yes
Neutral	Yes	Yes
Confident	Yes	Yes
Neutral	Yes	Yes

Neutral	Yes	Yes
Neutral	Yes	Yes
Confident	Yes	Yes
Confident	Yes	Yes
Confident	Yes	Yes
Confident	Yes	Yes
Confident	Yes	Yes
Confident	Yes	Yes
Confident	Yes	Yes
Confident	Yes	Yes
Confident	Yes	Yes
Confident	Yes	Yes
Confident	Yes	Yes
Neutral	Yes	Yes
Confident	Yes	Yes
Neutral	Yes	Yes
Not confident	Yes	Yes
Confident	Yes	Yes
Confident	Yes	Yes
Confident	Yes	Yes
Confident	Yes	Yes
Confident	Yes	Yes
Confident	Yes	Yes
Neutral	Yes	Yes
Very confident	Yes	Yes
Confident	Yes	Yes
	Yes	Yes
Confident	Yes	Yes
Confident	Yes	Yes
Confident	Yes	Yes
Confident	Yes	Yes
Confident	Yes	Yes
Very confident	Yes	Yes

Confident	Yes	Yes
Not confident	Yes	Yes
Neutral	Yes	Yes
Confident	Yes	Yes
Confident	Yes	Yes
Confident	Yes	Yes
Confident	Yes	Yes
Confident	Yes	Yes
Confident	Yes	Yes
Confident	Yes	Yes
Confident	Yes	Yes
Confident	Yes	Yes
Neutral	Yes	Yes
Confident	Yes	Yes
Neutral	Yes	Yes
Confident	Yes	Yes
Neutral	Yes	Yes
Confident	Yes	Yes
Neutral	Yes	Yes
Confident	Yes	Yes
Very confident	Yes	Yes
Confident	Yes	Yes
Confident	Yes	Yes
Confident	Yes	Yes
Confident	Yes	Yes
Confident	Yes	Yes
Confident	Yes	Yes
Confident	Yes	Yes
Confident	Yes	Yes
Confident	Yes	Yes
Confident	Yes	Yes
Confident	Yes	Yes
Confident	Yes	Yes

Confident	Yes	Yes
Confident	Yes	Yes
Confident	Yes	Yes
Confident	Yes	Yes
Confident	Yes	Yes
Confident	Yes	Yes
Very confident	Yes	Yes
Confident	Yes	Yes
Confident	Yes	Yes
Confident	Yes	Yes
Confident	Yes	Yes
Neutral	Yes	Yes
Neutral	Yes	Yes
Confident	Yes	Yes
Confident	Yes	Yes
Confident	Yes	Yes
Confident	Yes	Yes
Confident	Yes	Yes
Very confident	Yes	Yes
Confident	Yes	Yes
Not confident	Yes	Yes
Confident	Yes	Yes
Confident	Yes	Yes
Neutral	Yes	Yes
Neutral	Yes	Yes
Confident	Yes	Yes
Confident	Yes	Yes
Neutral	Yes	Yes
Neutral	Yes	Yes
Confident	Yes	Yes
Confident	Yes	Yes
Confident	Yes	Yes
Not confident	Yes	Yes

Confident	Yes	Yes
Not confident at all	No	
Confident	Yes	Yes
Confident	Yes	Yes
Neutral	Yes	No
Confident	Yes	Yes
Confident	Yes	Yes
Very confident	Yes	Yes
Not confident	Yes	Yes
Very confident	Yes	Yes
Confident	Yes	Yes
Neutral	Yes	Yes
Confident	Yes	Yes
Confident	Yes	Yes
Neutral	Yes	Yes
Confident	Yes	Yes
Neutral	Yes	Yes
Confident	Yes	Yes
Confident	Yes	Yes
Confident	Yes	Yes
Confident	Yes	Yes
Confident	Yes	Yes
Confident	Yes	Yes
Confident	Yes	Yes
Neutral	Yes	Yes
Neutral	Yes	Yes
Neutral	Yes	Yes
Neutral	Yes	Yes
Confident	Yes	Yes
Confident	Yes	Yes
Neutral	Yes	Yes
Not confident	Yes	Yes
Very confident	Yes	Yes

Very confident	Yes	Yes
Very confident	Yes	Yes
Confident	Yes	Yes
Neutral	Yes	Yes
Very confident	Yes	Yes
Confident	Yes	Yes
Confident	Yes	Yes
Confident	Yes	Yes
Confident	Yes	Yes
Neutral	Yes	Yes
Confident	Yes	Yes
Neutral	Yes	Yes
Neutral	Yes	Yes
Confident	Yes	Yes
Confident	Yes	Yes
Confident	Yes	Yes
Neutral	Yes	Yes
Confident	Yes	Yes
Confident	Yes	Yes
Neutral	Yes	Yes
Confident	Yes	Yes
Confident	Yes	Yes
Not confident	Yes	Yes
Confident	Yes	Yes
Neutral	Yes	Yes
Neutral	Yes	Yes
Not confident	Yes	Yes
Confident	Yes	Yes
Confident	Yes	Yes
Confident	Yes	Yes
Confident	Yes	Yes
Confident	Yes	Yes
Confident	Yes	Yes

Confident	Yes	Yes
Neutral	Yes	Yes
Confident	Yes	Yes
Confident	Yes	Yes
Confident	Yes	Yes
Confident	Yes	Yes
Confident	Yes	Yes
Confident	Yes	Yes
Confident	Yes	Yes
Confident	Yes	Yes
Not confident	Yes	Yes
Confident	Yes	Yes
Neutral	Yes	Yes
Neutral	Yes	Yes
Not confident	Yes	Yes
Neutral	Yes	Yes
Confident	Yes	Yes
Very confident	Yes	Yes
Confident	Yes	Yes
Confident	Yes	Yes
Confident	Yes	Yes
Neutral	Yes	Yes
Confident	Yes	Yes
Confident	Yes	Yes
Confident	Yes	Yes
Confident	Yes	Yes
Neutral	Yes	Yes
Confident	Yes	Yes
Confident	Yes	Yes
Neutral	Yes	Yes
Confident	Yes	Yes
Confident	Yes	Yes
Confident	Yes	Yes

Confident	Yes	Yes
Neutral	Yes	Yes
Very confident	Yes	Yes
Confident	Yes	Yes
Confident	Yes	Yes
Confident	Yes	Yes
Confident	Yes	Yes
Neutral	Yes	Yes
Not confident	Yes	Yes
Confident	Yes	Yes
Confident	Yes	Yes
Confident	Yes	Yes
Neutral	Yes	Yes
Confident	Yes	Yes
Not confident	Yes	Yes
Confident	Yes	Yes
Confident	Yes	Yes
Confident	Yes	Yes
Confident	Yes	Yes
Confident	Yes	Yes
Confident	Yes	Yes
Confident	Yes	Yes
Very confident	Yes	Yes
Not confident	Yes	Yes
Neutral	Yes	Yes
Neutral	Yes	Yes
Confident	Yes	Yes
Confident	Yes	Yes
Confident	Yes	Yes
Confident	Yes	Yes
Very confident	Yes	Yes
Confident	Yes	Yes
Confident	Yes	Yes

Confident	Yes	Yes
Confident	Yes	Yes
Confident	Yes	Yes
Not confident	Yes	Yes
Confident	Yes	Yes
Neutral	Yes	Yes
Confident	Yes	Yes
Neutral	Yes	Yes
Confident	Yes	Yes
Confident	Yes	Yes
Very confident	Yes	Yes
Confident	Yes	Yes
Very confident	Yes	Yes
Confident	Yes	Yes
Confident	Yes	Yes
Confident	Yes	Yes
Confident	Yes	Yes
Confident	Yes	Yes
Confident	Yes	Yes
Confident	Yes	Yes
Neutral	Yes	Yes
Confident	Yes	Yes
Confident	Yes	Yes
Confident	Yes	Yes
Not confident	Yes	Yes
Neutral	Yes	Yes
Neutral	Yes	Yes
Neutral	Yes	Yes
Confident	Yes	Yes
Neutral	Yes	Yes
Confident	Yes	Yes
Confident	Yes	Yes
Confident	Yes	Yes

Very confident	Yes	Yes
Confident	Yes	Yes
Confident	Yes	Yes
Confident	Yes	Yes
Confident	Yes	Yes
Confident	Yes	Yes
Not confident	Yes	Yes
Confident	Yes	Yes
Confident	Yes	Yes
Confident	Yes	Yes
Confident	Yes	Yes
Neutral	Yes	Yes
Confident	Yes	Yes
Confident	Yes	Yes
Confident	Yes	Yes
Very confident	Yes	Yes
Confident	Yes	Yes
Confident	Yes	Yes
Confident	Yes	Yes
Confident	Yes	Yes
Neutral	Yes	Yes
Confident	Yes	Yes
Not confident	Yes	Yes
Confident	Yes	Yes
Confident	Yes	Yes
Confident	Yes	Yes
Confident	Yes	Yes
Confident	Yes	Yes
Neutral	Yes	Yes
Confident	Yes	Yes
Confident	Yes	Yes
Confident	Yes	Yes
Confident	Yes	Yes

Confident	Yes	Yes
Confident	Yes	Yes
Confident	Yes	Yes
Confident	Yes	Yes
Very confident	Yes	Yes
Confident	Yes	Yes
Not confident	Yes	Yes
Neutral	Yes	Yes
Confident	Yes	Yes
Very confident	Yes	Yes
Confident	Yes	Yes
Confident	Yes	Yes
Confident	Yes	Yes
Confident	Yes	Yes
Confident	Yes	Yes
Confident	Yes	Yes
Not confident	Yes	Yes
Neutral	Yes	Yes
Confident	Yes	Yes
Confident	Yes	Yes
Not confident	Yes	Yes
Confident	Yes	Yes
Confident	Yes	Yes
Neutral	Yes	Yes
Confident	Yes	Yes
Confident	Yes	No
Confident	Yes	Yes
Confident	Yes	Yes
Confident	Yes	Yes
Neutral	Yes	Yes
Confident	Yes	Yes
Not confident	Yes	Yes
Very confident	Yes	Yes

Neutral	Yes	Yes
Confident	Yes	Yes
Confident	Yes	Yes
Neutral	Yes	Yes
Confident	Yes	Yes
Confident	Yes	No
Confident	Yes	Yes
Neutral	Yes	Yes
Neutral	Yes	Yes
Confident	Yes	Yes
Confident	Yes	Yes
Confident	Yes	Yes
Confident	Yes	Yes
Confident	Yes	Yes
Confident	Yes	Yes
Confident	Yes	Yes
Confident	Yes	Yes
Neutral	Yes	Yes
Not confident	Yes	Yes
Confident	Yes	Yes
Confident	Yes	Yes
Very confident	Yes	Yes
Very confident	Yes	No
Confident	Yes	Yes
Confident	Yes	Yes
Confident	Yes	Yes
Neutral	Yes	Yes
Very confident	Yes	Yes
Confident	Yes	No
Confident	Yes	Yes
Confident	Yes	Yes
Neutral	Yes	Yes
Neutral	Yes	Yes

Not confident	Yes	Yes
Confident	Yes	Yes
Neutral	Yes	No
Confident	Yes	Yes
Neutral	Yes	Yes
Confident	Yes	Yes
Confident	Yes	Yes
Confident	Yes	Yes
Neutral	Yes	Yes
Confident	Yes	Yes
Confident	Yes	Yes
Neutral	Yes	Yes
Confident	Yes	Yes
Neutral	Yes	Yes
Neutral	Yes	Yes
Very confident	Yes	Yes
Confident	Yes	Yes
Confident	Yes	No
Confident	Yes	Yes
Confident	Yes	Yes
Confident	Yes	Yes
Not confident	Yes	Yes
Confident	Yes	Yes
Confident	Yes	Yes
Confident	Yes	Yes
Confident	Yes	Yes
Neutral	Yes	No
Confident	Yes	Yes
Confident	Yes	Yes
Confident	Yes	Yes
Very confident	Yes	Yes
Confident	Yes	Yes
Not confident	Yes	Yes

Confident	Yes	Yes
Neutral	Yes	Yes
Confident	Yes	Yes
Confident	Yes	Yes
Confident	Yes	Yes
Confident	Yes	Yes
Confident	Yes	Yes
Confident	Yes	Yes
Confident	Yes	Yes
Very confident	Yes	Yes
Neutral	Yes	Yes
Not confident	Yes	Yes
Neutral	Yes	Yes
Not confident	Yes	Yes
Confident	Yes	Yes
Neutral	Yes	No
Neutral	Yes	Yes
Neutral	Yes	Yes
Confident	Yes	Yes
Confident	Yes	Yes
Confident	Yes	Yes
Confident	Yes	Yes
Confident	Yes	Yes
Confident	Yes	Yes
Confident	Yes	Yes
Not confident	Yes	Yes
Confident	Yes	Yes
Confident	Yes	Yes
Very confident	Yes	Yes
Confident	Yes	Yes
Neutral	Yes	Yes
Neutral	Yes	Yes
Confident	Yes	Yes

Confident	Yes	Yes
Neutral	Yes	Yes
Confident	Yes	Yes
Confident	Yes	Yes
Confident	Yes	Yes
Confident	Yes	Yes
Not confident	Yes	Yes
Neutral	Yes	Yes
Confident	Yes	Yes
Confident	Yes	Yes
Not confident	Yes	Yes
Confident	Yes	Yes
Confident	Yes	Yes
Neutral	Yes	Yes
Neutral	Yes	Yes
Neutral	Yes	Yes
Confident	Yes	Yes
Neutral	Yes	Yes
Confident	Yes	Yes
Confident	Yes	Yes
Confident	Yes	Yes
Neutral	Yes	Yes
Confident	Yes	Yes
Confident	Yes	Yes
Confident	Yes	Yes
Not confident	Yes	Yes
Neutral	Yes	Yes
Confident	Yes	Yes
Neutral	Yes	Yes
Confident	Yes	Yes
Neutral	Yes	Yes
Confident	Yes	Yes
Neutral	Yes	Yes

Confident	Yes	Yes
Confident	Yes	Yes
Neutral	Yes	Yes
Neutral	Yes	Yes
Confident	Yes	Yes
Neutral	Yes	Yes
Confident	Yes	Yes
Confident	Yes	Yes
Confident	Yes	No
Confident	Yes	Yes
Neutral	Yes	Yes
Confident	Yes	Yes
Confident	Yes	Yes
Confident	Yes	Yes
Neutral	Yes	Yes
Confident	Yes	Yes
Confident	Yes	Yes
Neutral	Yes	Yes
Neutral	Yes	Yes
Confident	Yes	Yes
Neutral	Yes	Yes
Confident	Yes	Yes
Confident	Yes	Yes
Confident	Yes	Yes
Confident	Yes	Yes
Confident	Yes	Yes
Confident	Yes	Yes
Neutral	Yes	Yes
Confident	Yes	Yes
Confident	Yes	Yes
Confident	Yes	No
Confident	Yes	Yes
Confident	Yes	Yes

Confident	Yes	Yes
Confident	Yes	Yes
Confident	Yes	Yes
Confident	Yes	Yes
Confident	Yes	Yes
Not confident	Yes	Yes
Confident	Yes	Yes
Confident	Yes	Yes
Neutral	Yes	Yes
Confident	Yes	Yes
Confident	Yes	Yes
Neutral	Yes	Yes
Confident	Yes	Yes
Confident	Yes	Yes
Neutral	Yes	Yes
Confident	Yes	Yes
Confident	Yes	Yes
Confident	Yes	Yes
Confident	Yes	Yes
Confident	Yes	Yes
Confident	Yes	Yes
Confident	Yes	Yes
Confident	Yes	Yes
Confident	Yes	Yes
Neutral	Yes	Yes
Confident	Yes	No
Confident	Yes	Yes
Confident	Yes	Yes
Neutral	Yes	Yes
Very confident	Yes	Yes
Not confident	Yes	Yes
Confident	Yes	Yes
Confident	Yes	Yes

Neutral	Yes	Yes
Confident	Yes	Yes
Confident	Yes	Yes
Confident	Yes	Yes
Confident	Yes	Yes
Neutral	Yes	Yes
Confident	Yes	Yes
Neutral	Yes	Yes
Confident	Yes	Yes
Confident	Yes	Yes
Confident	Yes	Yes
Confident	Yes	Yes
Confident	Yes	Yes
Confident	Yes	Yes
Confident	Yes	Yes
Confident	Yes	Yes
Neutral	Yes	Yes
Confident	Yes	Yes
Not confident	Yes	No
Confident	Yes	No

If no, does the participant want to proceed for healthcare	If yes, what was your body posture during self sampling e	Others
	One leg raised and supported	
	Squatting/partial squatting	
	One leg raised and supported	
	Sitting	
	One leg raised and supported	
Yes		
	Sitting	
	Squatting/partial squatting	
	One leg raised and supported	
	One leg raised and supported	
Yes		
	Sitting	
	One leg raised and supported	
	One leg raised and supported	
	Sitting	
	Squatting/partial squatting	
	One leg raised and supported	
	One leg raised and supported	
	Squatting/partial squatting	
	One leg raised and supported	
	Squatting/partial squatting	
	One leg raised and supported	
	Sitting	
	One leg raised and supported	
Yes		
	Squatting/partial squatting	
	One leg raised and supported	
	Squatting/partial squatting	
	One leg raised and supported	
	Squatting/partial squatting	
	One leg raised and supported	
	One leg raised and supported	

Yes	
Yes	
	Squatting/partial squatting
	One leg raised and supported
	Squatting/partial squatting
	One leg raised and supported
	One leg raised and supported
	One leg raised and supported
	One leg raised and supported
	One leg raised and supported
	Squatting/partial squatting
	Squatting/partial squatting
	One leg raised and supported
	One leg raised and supported
	Squatting/partial squatting
	One leg raised and supported
	Sitting
	One leg raised and supported
	Sitting
	Squatting/partial squatting
	Squatting/partial squatting
	Squatting/partial squatting
	Squatting/partial squatting
	Squatting/partial squatting
	Squatting/partial squatting
	One leg raised and supported
	One leg raised and supported
Yes	
	Squatting/partial squatting
	Sitting
	One leg raised and supported
	Squatting/partial squatting
	Sitting

Sitting	
One leg raised and supported	
Squatting/partial squatting	
Sitting	
Squatting/partial squatting	
One leg raised and supported	
Squatting/partial squatting	
Sitting	
One leg raised and supported	
One leg raised and supported	
Squatting/partial squatting	
Squatting/partial squatting	
One leg raised and supported	
Others	Lie down a
One leg raised and supported	
Sitting	
Squatting/partial squatting	
Sitting	
Squatting/partial squatting	
Squatting/partial squatting	
One leg raised and supported	
Squatting/partial squatting	
One leg raised and supported	
One leg raised and supported	
Squatting/partial squatting	
One leg raised and supported	
One leg raised and supported	
One leg raised and supported	
One leg raised and supported	
Squatting/partial squatting	
Sitting	
Squatting/partial squatting	
Squatting/partial squatting	

One leg raised and supported	
Sitting	
Squatting/partial squatting	
Squatting/partial squatting	
One leg raised and supported	
Sitting	
Squatting/partial squatting	
Others	Lying dow
Sitting	
One leg raised and supported	
Squatting/partial squatting	
One leg raised and supported	
Squatting/partial squatting	
One leg raised and supported	
One leg raised and supported	
Sitting	
Squatting/partial squatting	
One leg raised and supported	
One leg raised and supported	
Squatting/partial squatting	
Sitting	
One leg raised and supported	
Squatting/partial squatting	
One leg raised and supported	
One leg raised and supported	
One leg raised and supported	
Squatting/partial squatting	
Squatting/partial squatting	
Sitting	
Squatting/partial squatting	
One leg raised and supported	
One leg raised and supported	
One leg raised and supported	

One leg raised and supported	
One leg raised and supported	
Sitting	
One leg raised and supported	
Squatting/partial squatting	
Squatting/partial squatting	
Squatting/partial squatting	
Others	Stand
One leg raised and supported	
Sitting	
Others	Stand
Squatting/partial squatting	
Squatting/partial squatting	
One leg raised and supported	
One leg raised and supported	
Squatting/partial squatting	
Squatting/partial squatting	
Squatting/partial squatting	
Squatting/partial squatting	
One leg raised and supported	
One leg raised and supported	
Squatting/partial squatting	
One leg raised and supported	
Squatting/partial squatting	
One leg raised and supported	
Others	Stand
Sitting	
One leg raised and supported	
Squatting/partial squatting	
Squatting/partial squatting	
One leg raised and supported	
One leg raised and supported	
One leg raised and supported	

Squatting/partial squatting	
Squatting/partial squatting	
Squatting/partial squatting	
One leg raised and supported	
Sitting	
Sitting	
Sitting	
Squatting/partial squatting	
Others	Stand
One leg raised and supported	
Others	Stand
Squatting/partial squatting	
Sitting	
Sitting	
One leg raised and supported	
Squatting/partial squatting	
Squatting/partial squatting	
Others	Stand
Others	Stand
Squatting/partial squatting	
One leg raised and supported	
One leg raised and supported	
Others	Stand
Sitting	
One leg raised and supported	
Squatting/partial squatting	
One leg raised and supported	
Squatting/partial squatting	
Sitting	
Sitting	
Squatting/partial squatting	
One leg raised and supported	
Squatting/partial squatting	

One leg raised and supported	
Squatting/partial squatting	
Squatting/partial squatting	
Squatting/partial squatting	
Sitting	
One leg raised and supported	
Sitting	
One leg raised and supported	
Squatting/partial squatting	
Squatting/partial squatting	
One leg raised and supported	
One leg raised and supported	
One leg raised and supported	
One leg raised and supported	
Sitting	
Squatting/partial squatting	
Sitting	
Squatting/partial squatting	
Others	Lying on be
One leg raised and supported	
One leg raised and supported	
Sitting	
Sitting	
Squatting/partial squatting	
Squatting/partial squatting	
One leg raised and supported	
One leg raised and supported	
One leg raised and supported	
Sitting	
Squatting/partial squatting	
One leg raised and supported	
Others	Sit and one
Sitting	

Sitting	
Sitting	
Others	Standing
One leg raised and supported	
Squatting/partial squatting	
Sitting	
Squatting/partial squatting	
Sitting	
One leg raised and supported	
One leg raised and supported	
One leg raised and supported	
Squatting/partial squatting	
Sitting	
One leg raised and supported	
Squatting/partial squatting	
One leg raised and supported	
Squatting/partial squatting	
One leg raised and supported	
One leg raised and supported	
One leg raised and supported	
One leg raised and supported	
One leg raised and supported	
One leg raised and supported	
Squatting/partial squatting	
Squatting/partial squatting	
Squatting/partial squatting	
Squatting/partial squatting	
One leg raised and supported	
Sitting	
One leg raised and supported	
Squatting/partial squatting	
One leg raised and supported	
One leg raised and supported	

One leg raised and supported	
One leg raised and supported	
One leg raised and supported	
One leg raised and supported	
Squatting/partial squatting	
One leg raised and supported	
One leg raised and supported	
Squatting/partial squatting	
One leg raised and supported	
Squatting/partial squatting	
Sitting	
One leg raised and supported	
One leg raised and supported	
Sitting	
One leg raised and supported	
Squatting/partial squatting	
One leg raised and supported	
Squatting/partial squatting	
Squatting/partial squatting	
One leg raised and supported	
Sitting	
One leg raised and supported	
Sitting	
Squatting/partial squatting	
One leg raised and supported	
Squatting/partial squatting	
One leg raised and supported	
One leg raised and supported	
Squatting/partial squatting	
Squatting/partial squatting	
Sitting	
One leg raised and supported	
Others	Stand

One leg raised and supported	
Squatting/partial squatting	
One leg raised and supported	
Sitting	
One leg raised and supported	
One leg raised and supported	
Sitting	
Sitting	
One leg raised and supported	
Others	Stand
Squatting/partial squatting	
One leg raised and supported	
One leg raised and supported	
Squatting/partial squatting	
Squatting/partial squatting	
One leg raised and supported	
One leg raised and supported	
One leg raised and supported	
One leg raised and supported	
One leg raised and supported	
Squatting/partial squatting	
Squatting/partial squatting	
One leg raised and supported	
Sitting	
One leg raised and supported	
Sitting	
Squatting/partial squatting	
Sitting	
Squatting/partial squatting	
Others	Stand
Squatting/partial squatting	
One leg raised and supported	
One leg raised and supported	

Sitting	
Squatting/partial squatting	
One leg raised and supported	
One leg raised and supported	
One leg raised and supported	
One leg raised and supported	
One leg raised and supported	
Squatting/partial squatting	
Sitting	
Sitting	
One leg raised and supported	
One leg raised and supported	
Sitting	
Squatting/partial squatting	
One leg raised and supported	
Squatting/partial squatting	
One leg raised and supported	
Sitting	
One leg raised and supported	
One leg raised and supported	
Squatting/partial squatting	
One leg raised and supported	
One leg raised and supported	
One leg raised and supported	
Squatting/partial squatting	
Squatting/partial squatting	
Squatting/partial squatting	
Sitting	
Sitting	
Others	Lying dow
Squatting/partial squatting	
Sitting	
Squatting/partial squatting	

	Squatting/partial squatting	
	Squatting/partial squatting	
	Squatting/partial squatting	
	Squatting/partial squatting	
	Squatting/partial squatting	
	Sitting	
	One leg raised and supported	
	One leg raised and supported	
	Squatting/partial squatting	
	Squatting/partial squatting	
	Others	Lie down s
	Squatting/partial squatting	
	Squatting/partial squatting	
	Squatting/partial squatting	
	Squatting/partial squatting	
	One leg raised and supported	
	Sitting	
	One leg raised and supported	
	Squatting/partial squatting	
	Squatting/partial squatting	
	One leg raised and supported	
	Squatting/partial squatting	
	One leg raised and supported	
	One leg raised and supported	
	One leg raised and supported	
	Squatting/partial squatting	
	Sitting	
	Squatting/partial squatting	
	Squatting/partial squatting	
No		
	Sitting	
	Squatting/partial squatting	
	Squatting/partial squatting	

Sitting	
Squatting/partial squatting	
One leg raised and supported	
Squatting/partial squatting	
Squatting/partial squatting	
Sitting	
Sitting	
One leg raised and supported	
Squatting/partial squatting	
One leg raised and supported	
Squatting/partial squatting	
Squatting/partial squatting	
Squatting/partial squatting	
Squatting/partial squatting	
Squatting/partial squatting	
One leg raised and supported	
Sitting	
Sitting	
One leg raised and supported	
Sitting	
Squatting/partial squatting	
Sitting	
Squatting/partial squatting	
One leg raised and supported	
Sitting	
Squatting/partial squatting	
Others	standing
Sitting	
One leg raised and supported	
One leg raised and supported	
One leg raised and supported	
Squatting/partial squatting	
One leg raised and supported	

One leg raised and supported	
One leg raised and supported	
One leg raised and supported	
Squatting/partial squatting	
One leg raised and supported	
Squatting/partial squatting	
One leg raised and supported	
One leg raised and supported	
Squatting/partial squatting	
One leg raised and supported	
One leg raised and supported	
Squatting/partial squatting	
Squatting/partial squatting	
One leg raised and supported	
Squatting/partial squatting	
One leg raised and supported	
Others	
Sitting	
Squatting/partial squatting	
One leg raised and supported	
One leg raised and supported	
One leg raised and supported	
One leg raised and supported	
One leg raised and supported	
One leg raised and supported	
One leg raised and supported	
Squatting/partial squatting	
Others	Lying with
Squatting/partial squatting	
Squatting/partial squatting	
One leg raised and supported	
One leg raised and supported	
Sitting	

One leg raised and supported
One leg raised and supported
Squatting/partial squatting
One leg raised and supported
Sitting
Squatting/partial squatting
Squatting/partial squatting
Squatting/partial squatting
Squatting/partial squatting
One leg raised and supported
Squatting/partial squatting
Squatting/partial squatting
One leg raised and supported
Sitting
Squatting/partial squatting
Squatting/partial squatting
One leg raised and supported
One leg raised and supported
Squatting/partial squatting
One leg raised and supported
One leg raised and supported
Squatting/partial squatting
Squatting/partial squatting
Squatting/partial squatting
Sitting
One leg raised and supported
Sitting
One leg raised and supported
Squatting/partial squatting
One leg raised and supported
Sitting
One leg raised and supported
One leg raised and supported

Squatting/partial squatting
Squatting/partial squatting
Squatting/partial squatting
Squatting/partial squatting
One leg raised and supported
Squatting/partial squatting
Squatting/partial squatting
Sitting
Squatting/partial squatting
One leg raised and supported
Squatting/partial squatting
One leg raised and supported
One leg raised and supported
Squatting/partial squatting
Squatting/partial squatting
Squatting/partial squatting
Sitting
Squatting/partial squatting
Sitting
One leg raised and supported
Squatting/partial squatting
Sitting
One leg raised and supported
One leg raised and supported
One leg raised and supported
Squatting/partial squatting
Squatting/partial squatting
One leg raised and supported
Squatting/partial squatting
Squatting/partial squatting
Squatting/partial squatting
One leg raised and supported
Squatting/partial squatting

Squatting/partial squatting
Squatting/partial squatting
Squatting/partial squatting
Squatting/partial squatting
One leg raised and supported
Squatting/partial squatting
One leg raised and supported
Squatting/partial squatting
Squatting/partial squatting
One leg raised and supported
Squatting/partial squatting
One leg raised and supported
Sitting
Squatting/partial squatting
One leg raised and supported
Sitting
One leg raised and supported
Squatting/partial squatting
One leg raised and supported
One leg raised and supported
Squatting/partial squatting
Squatting/partial squatting
Squatting/partial squatting
One leg raised and supported
Sitting
One leg raised and supported
Squatting/partial squatting
Squatting/partial squatting
One leg raised and supported
Squatting/partial squatting
One leg raised and supported
One leg raised and supported
One leg raised and supported

One leg raised and supported
Sitting
Sitting
Squatting/partial squatting
Squatting/partial squatting
One leg raised and supported
Squatting/partial squatting
One leg raised and supported
One leg raised and supported
Sitting
One leg raised and supported
One leg raised and supported
Squatting/partial squatting
One leg raised and supported
Squatting/partial squatting
Squatting/partial squatting
Squatting/partial squatting
One leg raised and supported
Squatting/partial squatting
Sitting
One leg raised and supported
Squatting/partial squatting
Squatting/partial squatting
One leg raised and supported
One leg raised and supported
Squatting/partial squatting
One leg raised and supported
Squatting/partial squatting
One leg raised and supported
Squatting/partial squatting
One leg raised and supported
Sitting
Squatting/partial squatting

Squatting/partial squatting
One leg raised and supported
One leg raised and supported
Sitting
Squatting/partial squatting
One leg raised and supported
One leg raised and supported
Sitting
Squatting/partial squatting
Squatting/partial squatting
One leg raised and supported
Squatting/partial squatting
One leg raised and supported
Squatting/partial squatting
Squatting/partial squatting
Squatting/partial squatting
Squatting/partial squatting
Squatting/partial squatting
Sitting
Squatting/partial squatting
Squatting/partial squatting
Squatting/partial squatting
Squatting/partial squatting
Squatting/partial squatting
One leg raised and supported
Squatting/partial squatting
Squatting/partial squatting
One leg raised and supported
One leg raised and supported
One leg raised and supported
Squatting/partial squatting
One leg raised and supported
Squatting/partial squatting

One leg raised and supported
One leg raised and supported
Squatting/partial squatting
Sitting
One leg raised and supported
One leg raised and supported
One leg raised and supported
Squatting/partial squatting
Sitting
Squatting/partial squatting
Squatting/partial squatting
One leg raised and supported
Squatting/partial squatting
One leg raised and supported
One leg raised and supported
Squatting/partial squatting
Squatting/partial squatting
One leg raised and supported
One leg raised and supported
Squatting/partial squatting
Squatting/partial squatting
One leg raised and supported
Squatting/partial squatting
Squatting/partial squatting
Squatting/partial squatting
Squatting/partial squatting
One leg raised and supported
One leg raised and supported
Squatting/partial squatting
Squatting/partial squatting
One leg raised and supported
Squatting/partial squatting
Squatting/partial squatting

	One leg raised and supported
	Squatting/partial squatting
	Squatting/partial squatting
	Squatting/partial squatting
	One leg raised and supported
	Squatting/partial squatting
	Squatting/partial squatting
	One leg raised and supported
Yes	
	Sitting
	Squatting/partial squatting
	Squatting/partial squatting
	One leg raised and supported
Yes	
	One leg raised and supported
	Squatting/partial squatting
	One leg raised and supported
	Squatting/partial squatting
	Squatting/partial squatting
	Squatting/partial squatting
	Squatting/partial squatting
	Sitting
	One leg raised and supported
	Squatting/partial squatting
	One leg raised and supported
	Squatting/partial squatting
	Squatting/partial squatting
	One leg raised and supported
	Squatting/partial squatting
	One leg raised and supported
	One leg raised and supported
	Squatting/partial squatting
	One leg raised and supported

One leg raised and supported
One leg raised and supported
One leg raised and supported
Squatting/partial squatting
One leg raised and supported
One leg raised and supported
Squatting/partial squatting
One leg raised and supported
One leg raised and supported
Squatting/partial squatting
Sitting
One leg raised and supported
One leg raised and supported
Squatting/partial squatting
Sitting
Squatting/partial squatting
Squatting/partial squatting
One leg raised and supported
One leg raised and supported
One leg raised and supported
One leg raised and supported
One leg raised and supported
Squatting/partial squatting
Squatting/partial squatting
Squatting/partial squatting
Sitting
Squatting/partial squatting
Squatting/partial squatting
Squatting/partial squatting
One leg raised and supported
Squatting/partial squatting
Squatting/partial squatting
Sitting

Squatting/partial squatting
Squatting/partial squatting
Squatting/partial squatting
Sitting
One leg raised and supported
Squatting/partial squatting
One leg raised and supported
One leg raised and supported
One leg raised and supported
Squatting/partial squatting
Sitting
Squatting/partial squatting
One leg raised and supported
Squatting/partial squatting
Squatting/partial squatting
Sitting
One leg raised and supported
Squatting/partial squatting
Squatting/partial squatting
Squatting/partial squatting
One leg raised and supported
Squatting/partial squatting
One leg raised and supported
Squatting/partial squatting
Squatting/partial squatting
Squatting/partial squatting
One leg raised and supported
Squatting/partial squatting
Squatting/partial squatting
One leg raised and supported
One leg raised and supported
One leg raised and supported
One leg raised and supported

Sitting
One leg raised and supported
One leg raised and supported
Squatting/partial squatting
Squatting/partial squatting
Squatting/partial squatting
One leg raised and supported
One leg raised and supported
Squatting/partial squatting
Sitting
Sitting
Sitting
One leg raised and supported
Squatting/partial squatting
One leg raised and supported
One leg raised and supported
One leg raised and supported
Squatting/partial squatting
Squatting/partial squatting
Sitting
Sitting
Squatting/partial squatting
Squatting/partial squatting
One leg raised and supported
One leg raised and supported
Squatting/partial squatting
One leg raised and supported
Squatting/partial squatting
Squatting/partial squatting
Squatting/partial squatting
Squatting/partial squatting
Squatting/partial squatting
Squatting/partial squatting

	Squatting/partial squatting	
	One leg raised and supported	
	One leg raised and supported	
	Squatting/partial squatting	
	One leg raised and supported	
No		
	Sitting	
	Squatting/partial squatting	
	Squatting/partial squatting	
	One leg raised and supported	
	Squatting/partial squatting	
	One leg raised and supported	
	Others	Lying dow
	Sitting	
	One leg raised and supported	
	One leg raised and supported	
	Squatting/partial squatting	
	Sitting	
	One leg raised and supported	
	One leg raised and supported	
	One leg raised and supported	
	Squatting/partial squatting	
	Squatting/partial squatting	
	One leg raised and supported	
	Squatting/partial squatting	
	One leg raised and supported	
	Squatting/partial squatting	
	One leg raised and supported	
	Sitting	
	One leg raised and supported	
	Sitting	
	One leg raised and supported	
	One leg raised and supported	

Squatting/partial squatting	
Squatting/partial squatting	
One leg raised and supported	
One leg raised and supported	
One leg raised and supported	
One leg raised and supported	
Squatting/partial squatting	
One leg raised and supported	
Squatting/partial squatting	
One leg raised and supported	
Squatting/partial squatting	
One leg raised and supported	
One leg raised and supported	
Squatting/partial squatting	
One leg raised and supported	
Squatting/partial squatting	
One leg raised and supported	
One leg raised and supported	
One leg raised and supported	
One leg raised and supported	
Squatting/partial squatting	
One leg raised and supported	
Squatting/partial squatting	
Squatting/partial squatting	
Squatting/partial squatting	
Squatting/partial squatting	
Sitting	
Squatting/partial squatting	
One leg raised and supported	
Others	Standing
Squatting/partial squatting	
Squatting/partial squatting	
One leg raised and supported	

	Squatting/partial squatting
	Squatting/partial squatting
	One leg raised and supported
	One leg raised and supported
	Sitting
	One leg raised and supported
	One leg raised and supported
	Squatting/partial squatting
	One leg raised and supported
	Squatting/partial squatting
	Squatting/partial squatting
	Squatting/partial squatting
	Squatting/partial squatting
	One leg raised and supported
	Sitting
	One leg raised and supported
	Sitting
	One leg raised and supported
	Squatting/partial squatting
	One leg raised and supported
	One leg raised and supported
	One leg raised and supported
	One leg raised and supported
	One leg raised and supported
Yes	
	Sitting
	Squatting/partial squatting
	One leg raised and supported
	One leg raised and supported
	One leg raised and supported
	Squatting/partial squatting
	One leg raised and supported
	One leg raised and supported

One leg raised and supported
Squatting/partial squatting
One leg raised and supported
One leg raised and supported
One leg raised and supported
One leg raised and supported
One leg raised and supported
Squatting/partial squatting
One leg raised and supported
One leg raised and supported
Squatting/partial squatting
Sitting
One leg raised and supported
Sitting
One leg raised and supported
One leg raised and supported
One leg raised and supported
One leg raised and supported
Squatting/partial squatting
Squatting/partial squatting
Squatting/partial squatting
Squatting/partial squatting
Squatting/partial squatting
Squatting/partial squatting
Squatting/partial squatting
Squatting/partial squatting
One leg raised and supported
One leg raised and supported
Squatting/partial squatting
Squatting/partial squatting
Squatting/partial squatting
Others
One leg raised and supported

	Squatting/partial squatting	
	One leg raised and supported	
	One leg raised and supported	
	One leg raised and supported	
	Squatting/partial squatting	
	Squatting/partial squatting	
	Squatting/partial squatting	
Yes		
	Others	tried both
	Squatting/partial squatting	
	Squatting/partial squatting	
	One leg raised and supported	
	Squatting/partial squatting	
	Sitting	
	Sitting	
	One leg raised and supported	
	Squatting/partial squatting	
	One leg raised and supported	
	Squatting/partial squatting	
	Squatting/partial squatting	
	Squatting/partial squatting	
	Squatting/partial squatting	
	Sitting	
	One leg raised and supported	
	One leg raised and supported	
	One leg raised and supported	
	One leg raised and supported	
	Squatting/partial squatting	
	One leg raised and supported	
	One leg raised and supported	
	One leg raised and supported	
Yes		
	Squatting/partial squatting	

	Squatting/partial squatting
	Squatting/partial squatting
	Squatting/partial squatting
	One leg raised and supported
	Squatting/partial squatting
	Squatting/partial squatting
	Squatting/partial squatting
	Squatting/partial squatting
	One leg raised and supported
	Squatting/partial squatting
	Squatting/partial squatting
	One leg raised and supported
	One leg raised and supported
	Squatting/partial squatting
	One leg raised and supported
Yes	
	One leg raised and supported
	One leg raised and supported
	One leg raised and supported
	Squatting/partial squatting
	One leg raised and supported
	One leg raised and supported
	One leg raised and supported
	One leg raised and supported
	Squatting/partial squatting
	One leg raised and supported
	Sitting
	One leg raised and supported
	One leg raised and supported
	Squatting/partial squatting
	One leg raised and supported
	Squatting/partial squatting
	Squatting/partial squatting

One leg raised and supported
Squatting/partial squatting
Squatting/partial squatting
One leg raised and supported
One leg raised and supported
One leg raised and supported
Squatting/partial squatting
Squatting/partial squatting
One leg raised and supported
One leg raised and supported
One leg raised and supported
Squatting/partial squatting
One leg raised and supported
Squatting/partial squatting
One leg raised and supported
Squatting/partial squatting
Sitting
Squatting/partial squatting
One leg raised and supported
One leg raised and supported
Squatting/partial squatting
Squatting/partial squatting
One leg raised and supported
Squatting/partial squatting
One leg raised and supported
Squatting/partial squatting
One leg raised and supported
Squatting/partial squatting
One leg raised and supported
Sitting
Squatting/partial squatting
Squatting/partial squatting
One leg raised and supported

	One leg raised and supported
	One leg raised and supported
No	
	One leg raised and supported
	Squatting/partial squatting
	Squatting/partial squatting
	Squatting/partial squatting
	Squatting/partial squatting
	One leg raised and supported
	One leg raised and supported
	Squatting/partial squatting
	One leg raised and supported
	One leg raised and supported
	One leg raised and supported
	Squatting/partial squatting
	One leg raised and supported
	Sitting
	Squatting/partial squatting
	One leg raised and supported
	One leg raised and supported
	Squatting/partial squatting
	One leg raised and supported
	One leg raised and supported
	One leg raised and supported
	One leg raised and supported
	Squatting/partial squatting
	One leg raised and supported
	One leg raised and supported
	Squatting/partial squatting
	Squatting/partial squatting
	Squatting/partial squatting
	One leg raised and supported
	One leg raised and supported

One leg raised and supported
One leg raised and supported
Sitting
One leg raised and supported
One leg raised and supported
Squatting/partial squatting
Squatting/partial squatting
Squatting/partial squatting
Squatting/partial squatting
Sitting
Sitting
One leg raised and supported
Squatting/partial squatting
Squatting/partial squatting
One leg raised and supported
Squatting/partial squatting
Squatting/partial squatting
One leg raised and supported
Squatting/partial squatting
Squatting/partial squatting
One leg raised and supported
One leg raised and supported
Squatting/partial squatting
Squatting/partial squatting
Squatting/partial squatting
One leg raised and supported
Squatting/partial squatting
One leg raised and supported
One leg raised and supported
Squatting/partial squatting
One leg raised and supported
Squatting/partial squatting
One leg raised and supported

One leg raised and supported	
One leg raised and supported	
One leg raised and supported	
One leg raised and supported	
Squatting/partial squatting	
One leg raised and supported	
One leg raised and supported	
Squatting/partial squatting	
Squatting/partial squatting	
One leg raised and supported	
One leg raised and supported	
Squatting/partial squatting	
One leg raised and supported	
One leg raised and supported	
One leg raised and supported	
One leg raised and supported	
One leg raised and supported	
One leg raised and supported	
Squatting/partial squatting	
Squatting/partial squatting	
Squatting/partial squatting	
Squatting/partial squatting	
Squatting/partial squatting	
One leg raised and supported	
Others	Lying dow
One leg raised and supported	
Others	Lying on b
Squatting/partial squatting	
One leg raised and supported	
Squatting/partial squatting	
Squatting/partial squatting	
Squatting/partial squatting	
One leg raised and supported	

Sitting	
One leg raised and supported	
One leg raised and supported	
Squatting/partial squatting	
Squatting/partial squatting	
Squatting/partial squatting	
One leg raised and supported	
Squatting/partial squatting	
One leg raised and supported	
Others	Lying on b
One leg raised and supported	
Squatting/partial squatting	
Squatting/partial squatting	
One leg raised and supported	
One leg raised and supported	
One leg raised and supported	
Squatting/partial squatting	
Squatting/partial squatting	
Squatting/partial squatting	
Squatting/partial squatting	
Squatting/partial squatting	
One leg raised and supported	
One leg raised and supported	
Others	Sitting by t
Squatting/partial squatting	
One leg raised and supported	
Squatting/partial squatting	
One leg raised and supported	
Squatting/partial squatting	
One leg raised and supported	
One leg raised and supported	
Squatting/partial squatting	
One leg raised and supported	

	Squatting/partial squatting	
Yes		
	Squatting/partial squatting	
	Sitting	
Yes		
	Squatting/partial squatting	
	Squatting/partial squatting	
	Others	lying dowr
	One leg raised and supported	
	One leg raised and supported	
	One leg raised and supported	
	Others	lying dowr
	Squatting/partial squatting	
	One leg raised and supported	
	One leg raised and supported	
	One leg raised and supported	
	Squatting/partial squatting	
	Squatting/partial squatting	
	Squatting/partial squatting	
	Squatting/partial squatting	
	One leg raised and supported	
	Squatting/partial squatting	
	One leg raised and supported	
	Others	lying dowr
	One leg raised and supported	
	One leg raised and supported	
	Sitting	
	Squatting/partial squatting	
	Squatting/partial squatting	
	One leg raised and supported	
	Squatting/partial squatting	
	Squatting/partial squatting	
	Squatting/partial squatting	

One leg raised and supported
One leg raised and supported
Squatting/partial squatting
One leg raised and supported
Sitting
One leg raised and supported
Squatting/partial squatting
One leg raised and supported
One leg raised and supported
One leg raised and supported
One leg raised and supported
One leg raised and supported
One leg raised and supported
Squatting/partial squatting
Squatting/partial squatting
Squatting/partial squatting
One leg raised and supported
One leg raised and supported
One leg raised and supported
Squatting/partial squatting
Squatting/partial squatting
Squatting/partial squatting
Squatting/partial squatting
One leg raised and supported
One leg raised and supported
One leg raised and supported
One leg raised and supported
Squatting/partial squatting
Squatting/partial squatting
Squatting/partial squatting
One leg raised and supported
One leg raised and supported
Sitting

One leg raised and supported
One leg raised and supported
Sitting
One leg raised and supported
Sitting
Sitting
One leg raised and supported
One leg raised and supported
One leg raised and supported
One leg raised and supported
Squatting/partial squatting
One leg raised and supported
One leg raised and supported
Squatting/partial squatting
One leg raised and supported
One leg raised and supported
Squatting/partial squatting
Squatting/partial squatting
Squatting/partial squatting
One leg raised and supported
One leg raised and supported
One leg raised and supported
Squatting/partial squatting
One leg raised and supported
One leg raised and supported
One leg raised and supported
Squatting/partial squatting
One leg raised and supported
Squatting/partial squatting
One leg raised and supported
One leg raised and supported
One leg raised and supported
Squatting/partial squatting

Squatting/partial squatting
One leg raised and supported
One leg raised and supported
One leg raised and supported
One leg raised and supported
Sitting
One leg raised and supported
One leg raised and supported
One leg raised and supported
One leg raised and supported
One leg raised and supported
One leg raised and supported
Squatting/partial squatting
One leg raised and supported
Squatting/partial squatting
Squatting/partial squatting
Squatting/partial squatting
One leg raised and supported
One leg raised and supported
Squatting/partial squatting
One leg raised and supported
Squatting/partial squatting
Squatting/partial squatting
Squatting/partial squatting
One leg raised and supported
Squatting/partial squatting
Squatting/partial squatting
Squatting/partial squatting
Squatting/partial squatting
Squatting/partial squatting
One leg raised and supported
One leg raised and supported
One leg raised and supported

One leg raised and supported
One leg raised and supported
One leg raised and supported
One leg raised and supported
One leg raised and supported
One leg raised and supported
One leg raised and supported
One leg raised and supported
One leg raised and supported
One leg raised and supported
One leg raised and supported
Squatting/partial squatting
Sitting
One leg raised and supported
One leg raised and supported
One leg raised and supported
One leg raised and supported
Squatting/partial squatting
Squatting/partial squatting
Squatting/partial squatting
One leg raised and supported
One leg raised and supported
One leg raised and supported
One leg raised and supported
One leg raised and supported
Squatting/partial squatting
One leg raised and supported
One leg raised and supported
Squatting/partial squatting
One leg raised and supported
One leg raised and supported
Sitting
Squatting/partial squatting

Squatting/partial squatting
One leg raised and supported
One leg raised and supported
One leg raised and supported
One leg raised and supported
Squatting/partial squatting
One leg raised and supported
One leg raised and supported
Squatting/partial squatting
Squatting/partial squatting
One leg raised and supported
One leg raised and supported
Sitting
One leg raised and supported
Squatting/partial squatting
One leg raised and supported
Sitting
Squatting/partial squatting
Squatting/partial squatting
One leg raised and supported
Squatting/partial squatting
One leg raised and supported
Squatting/partial squatting
One leg raised and supported
Squatting/partial squatting
Squatting/partial squatting
One leg raised and supported
Squatting/partial squatting
One leg raised and supported
Sitting
Squatting/partial squatting
Sitting
Squatting/partial squatting

	One leg raised and supported
	One leg raised and supported
	Squatting/partial squatting
	One leg raised and supported
	Squatting/partial squatting
	Squatting/partial squatting
	Squatting/partial squatting
	Sitting
	One leg raised and supported
	One leg raised and supported
	Sitting
	One leg raised and supported
	One leg raised and supported
	Sitting
	One leg raised and supported
	Sitting
	One leg raised and supported
	Squatting/partial squatting
	Squatting/partial squatting
	Squatting/partial squatting
	Squatting/partial squatting
	Squatting/partial squatting
	One leg raised and supported
	One leg raised and supported
	Sitting
Yes	
	One leg raised and supported
	One leg raised and supported
	One leg raised and supported
	One leg raised and supported
	One leg raised and supported
	One leg raised and supported
	One leg raised and supported

	Squatting/partial squatting	
	Sitting	
	Squatting/partial squatting	
	One leg raised and supported	
	One leg raised and supported	
Yes		
	One leg raised and supported	
	Sitting	
	Sitting	
	One leg raised and supported	
	Squatting/partial squatting	
	Squatting/partial squatting	
	Sitting	
	Squatting/partial squatting	
	One leg raised and supported	
	Squatting/partial squatting	
	One leg raised and supported	
	Others	Standing a
	One leg raised and supported	
	Squatting/partial squatting	
	One leg raised and supported	
	Squatting/partial squatting	
Yes		
	Squatting/partial squatting	
	Squatting/partial squatting	
	One leg raised and supported	
	One leg raised and supported	
	One leg raised and supported	
Yes		
	Sitting	
	Squatting/partial squatting	
	One leg raised and supported	
	Sitting	

	Squatting/partial squatting
	One leg raised and supported
No	
	One leg raised and supported
	One leg raised and supported
	Squatting/partial squatting
	Sitting
	One leg raised and supported
	One leg raised and supported
	Squatting/partial squatting
	Sitting
	One leg raised and supported
	Squatting/partial squatting
	One leg raised and supported
	Squatting/partial squatting
	One leg raised and supported
	Squatting/partial squatting
Yes	
	One leg raised and supported
	One leg raised and supported
	One leg raised and supported
	Squatting/partial squatting
	Sitting
	One leg raised and supported
	Sitting
	One leg raised and supported
No	
	One leg raised and supported
	Sitting
	Sitting
	Sitting
	Sitting
	Squatting/partial squatting

	Squatting/partial squatting
	Squatting/partial squatting
	One leg raised and supported
	One leg raised and supported
	Squatting/partial squatting
	One leg raised and supported
	One leg raised and supported
	One leg raised and supported
	One leg raised and supported
	One leg raised and supported
	One leg raised and supported
	One leg raised and supported
	Sitting
	Sitting
	Squatting/partial squatting
Yes	
	One leg raised and supported
	Squatting/partial squatting
	Sitting
	One leg raised and supported
	Squatting/partial squatting
	One leg raised and supported
	One leg raised and supported
	One leg raised and supported
	One leg raised and supported
	One leg raised and supported
	One leg raised and supported
	One leg raised and supported
	Squatting/partial squatting
	One leg raised and supported
	One leg raised and supported
	One leg raised and supported
	One leg raised and supported

One leg raised and supported
One leg raised and supported
One leg raised and supported
Squatting/partial squatting
One leg raised and supported
One leg raised and supported
Squatting/partial squatting
One leg raised and supported
Sitting
One leg raised and supported
One leg raised and supported
Squatting/partial squatting
One leg raised and supported
One leg raised and supported
Squatting/partial squatting
One leg raised and supported
One leg raised and supported
One leg raised and supported
Squatting/partial squatting
One leg raised and supported
One leg raised and supported
One leg raised and supported
Squatting/partial squatting
One leg raised and supported
One leg raised and supported
Squatting/partial squatting
Sitting
Squatting/partial squatting
One leg raised and supported
One leg raised and supported
One leg raised and supported
Sitting
One leg raised and supported

	One leg raised and supported	
	Squatting/partial squatting	
	One leg raised and supported	
	Sitting	
	Others	Standing
	One leg raised and supported	
	One leg raised and supported	
	Squatting/partial squatting	
Yes		
	One leg raised and supported	
	One leg raised and supported	
	Squatting/partial squatting	
	Squatting/partial squatting	
	Sitting	
	One leg raised and supported	
	One leg raised and supported	
	One leg raised and supported	
	One leg raised and supported	
	One leg raised and supported	
	One leg raised and supported	
	Sitting	
	One leg raised and supported	
	One leg raised and supported	
	One leg raised and supported	
	One leg raised and supported	
	Sitting	
	One leg raised and supported	
	One leg raised and supported	
	One leg raised and supported	
	One leg raised and supported	
Yes		
	Squatting/partial squatting	
	Squatting/partial squatting	

	Squatting/partial squatting
	Squatting/partial squatting
	One leg raised and supported
	One leg raised and supported
	Squatting/partial squatting
	One leg raised and supported
	One leg raised and supported
	One leg raised and supported
	One leg raised and supported
	One leg raised and supported
	Squatting/partial squatting
	Squatting/partial squatting
	One leg raised and supported
	One leg raised and supported
	One leg raised and supported
	One leg raised and supported
	One leg raised and supported
	One leg raised and supported
	One leg raised and supported
	Squatting/partial squatting
	One leg raised and supported
	One leg raised and supported
	One leg raised and supported
	One leg raised and supported
	One leg raised and supported
Yes	
	One leg raised and supported
	One leg raised and supported
	One leg raised and supported
	Squatting/partial squatting
	One leg raised and supported
	One leg raised and supported
	One leg raised and supported

	Squatting/partial squatting	
	Squatting/partial squatting	
	Others	Standing
	One leg raised and supported	
	One leg raised and supported	
	One leg raised and supported	
	Others	Sitting + le
	One leg raised and supported	
	One leg raised and supported	
	One leg raised and supported	
	One leg raised and supported	
	Squatting/partial squatting	
	One leg raised and supported	
	Squatting/partial squatting	
	Squatting/partial squatting	
	Squatting/partial squatting	
	Sitting	
	Squatting/partial squatting	
No		
No		

Overall, how do you feel about taking your own vaginal s	How easy was it to collect this self-sample?	How convenient was it to collect this self-sample?
Neutral	Easy	Convenient
Good	Easy	Convenient
Good	Very easy	Very convenient
Neutral	Hard	Very convenient
Good	Very easy	Very convenient
Good	Neutral	Convenient
Good	Easy	Convenient
Good	Very easy	Very convenient
Good	Very easy	Very convenient
Neutral	Hard	Neutral
Neutral	Very easy	Very convenient
Good	Easy	Very convenient
Very good	Easy	Very convenient
Very good	Easy	Convenient
Good	Very easy	Very convenient
Good	Very easy	Very convenient
Good	Very easy	Very convenient
Good	Very easy	Convenient
Very good	Very easy	Very convenient
Good	Easy	Convenient
Good	Easy	Convenient
Very good	Very easy	Convenient
Neutral	Neutral	Not convenient
Neutral	Hard	Neutral
Very good	Very easy	Very convenient
Good	Easy	Convenient
Very good	Easy	Very convenient
Good	Easy	Convenient
Very good	Very easy	Very convenient
Very good	Very easy	Very convenient
Neutral	Easy	Convenient

Very good	Easy	Convenient
Good	Very easy	Very convenient
Good	Easy	Very convenient
Good	Easy	Convenient
Good	Neutral	Convenient
Good	Easy	Convenient
Good	Easy	Very convenient
Good	Very easy	Very convenient
Good	Easy	Convenient
Neutral	Easy	Convenient
Bad	Hard	Convenient
Good	Easy	Convenient
Good	Very easy	Convenient
Good	Neutral	Convenient
Neutral	Easy	Convenient
Good	Easy	Convenient
Good	Easy	Very convenient
Neutral	Easy	Convenient
Good	Very easy	Very convenient
Good	Very easy	Convenient
Good	Easy	Convenient
Good	Easy	Convenient
Good	Very easy	Convenient
Good	Neutral	Very convenient
Good	Easy	Convenient
Very good	Very easy	Very convenient
Good	Easy	Convenient
Good	Easy	Convenient
Neutral	Easy	Convenient
Very good	Very easy	Convenient

Good	Very easy	Very convenient
Neutral	Easy	Neutral
Neutral	Easy	Convenient
Good	Easy	Very convenient
Neutral	Easy	Convenient
Good	Very easy	Very convenient
Good	Easy	Convenient
Good	Easy	Convenient
Very good	Easy	Convenient
Good	Very easy	Very convenient
Good	Very easy	Very convenient
Good	Easy	Convenient
Good	Easy	Very convenient
Very good	Easy	Convenient
Very good	Easy	Very convenient
Very good	Easy	Convenient
Good	Very easy	Very convenient
Good	Very easy	Convenient
Good	Very easy	Very convenient
Neutral	Easy	Convenient
Good	Neutral	Very convenient
Neutral	Easy	Convenient
Very good	Easy	Very convenient
Very good	Very easy	Very convenient
Neutral	Hard	Convenient
Neutral	Very easy	Convenient
Good	Easy	Convenient
Good	Easy	Convenient
Neutral	Easy	Very convenient
Very good	Very easy	Very convenient
Very good	Very easy	Very convenient
Neutral	Easy	Very convenient
Neutral	Easy	Very convenient

Good	Easy	Convenient
Good	Very easy	Convenient
Good	Very easy	Very convenient
Neutral	Easy	Convenient
Neutral	Easy	Convenient
Good	Easy	Convenient
Good	Easy	Convenient
Good	Easy	Convenient
Good	Very easy	Very convenient
Neutral	Easy	Convenient
Neutral	Very easy	Very convenient
Good	Easy	Convenient
Good	Easy	Very convenient
Good	Very easy	Very convenient
Good	Easy	Convenient
Neutral	Easy	Convenient
Good	Very easy	Convenient
Good	Easy	Very convenient
Good	Very easy	Very convenient
Good	Very easy	Convenient
Neutral	Neutral	Convenient
Good	Very easy	Very convenient
Good	Very easy	Very convenient
Good	Neutral	Convenient
Good	Very easy	Very convenient
Good	Very easy	Very convenient
Very good	Very easy	Very convenient
Neutral	Easy	Convenient
Good	Very easy	Convenient
Very good	Very easy	Very convenient
Good	Easy	Convenient
Very good	Very easy	Very convenient
Good	Very easy	Convenient

Good	Easy	Convenient
Neutral	Very easy	Very convenient
Good	Very easy	Very convenient
Neutral	Neutral	Convenient
Neutral	Easy	Very convenient
Neutral	Easy	Convenient
Good	Very easy	Very convenient
Neutral	Neutral	Convenient
Neutral	Very easy	Very convenient
Neutral	Hard	Not convenient
Neutral	Neutral	Convenient
Good	Easy	Very convenient
Good	Neutral	Convenient
Good	Hard	Convenient
Good	Very easy	Very convenient
Neutral	Easy	Convenient
Neutral	Easy	Convenient
Neutral	Neutral	Convenient
Good	Easy	Convenient
Very good	Easy	Very convenient
Neutral	Neutral	Convenient
Good	Easy	Convenient
Good	Easy	Convenient
Good	Very easy	Very convenient
Neutral	Easy	Very convenient
Good	Easy	Very convenient
Good	Very easy	Very convenient
Good	Easy	Convenient
Good	Easy	Convenient
Neutral	Easy	Convenient
Neutral	Easy	Convenient
Neutral	Easy	Convenient
Good	Easy	Very convenient

Neutral	Very easy	Very convenient
Very good	Very easy	Very convenient
Bad	Hard	Neutral
Good	Easy	Convenient
Good	Easy	Convenient
Good	Easy	Convenient
Neutral	Easy	Convenient
Neutral	Easy	Convenient
Very bad	Very hard	Not convenient
Neutral	Easy	Convenient
Good	Easy	Convenient
Good	Very easy	Convenient
Good	Easy	Convenient
Good	Easy	Convenient
Neutral	Hard	Convenient
Neutral	Very easy	Very convenient
Neutral	Easy	Very convenient
Good	Very easy	Very convenient
Neutral	Easy	Convenient
Neutral	Easy	Very convenient
Neutral	Easy	Convenient
Good	Very easy	Very convenient
Good	Very easy	Very convenient
Good	Very easy	Very convenient
Neutral	Very easy	Convenient
Good	Very easy	Very convenient
Neutral	Easy	Convenient
Good	Easy	Convenient
Good	Very easy	Very convenient
Good	Very easy	Very convenient
Good	Easy	Convenient
Good	Very easy	Very convenient
Good	Very easy	Very convenient

Good	Very easy	Very convenient
Good	Very easy	Very convenient
Good	Very easy	Very convenient
Very good	Very easy	Very convenient
Very good	Very easy	Very convenient
Bad	Hard	Convenient
Good	Very easy	Very convenient
Very good	Very easy	Convenient
Good	Very easy	Convenient
Good	Very easy	Very convenient
Very good	Very easy	Very convenient
Good	Easy	Convenient
Good	Very easy	Very convenient
Good	Very easy	Very convenient
Good	Very easy	Very convenient
Neutral	Hard	Convenient
Good	Easy	Very convenient
Good	Hard	Convenient
Very good	Very easy	Very convenient
Neutral	Easy	Very convenient
Good	Easy	Very convenient
Neutral	Easy	Very convenient
Good	Very easy	Very convenient
Very good	Very easy	Very convenient
Good	Easy	Convenient
Very good	Very easy	Very convenient
Very good	Very easy	Very convenient
Good	Neutral	Convenient
Neutral	Easy	Very convenient
Good	Very easy	Very convenient
Good	Very easy	Convenient
Good	Easy	Very convenient
Good	Easy	Convenient

Very good	Very easy	Very convenient
Very good	Very easy	Very convenient
Neutral	Neutral	Neutral
Good	Very easy	Convenient
Good	Very easy	Very convenient
Neutral	Neutral	Convenient
Good	Easy	Very convenient
Good	Easy	Convenient
Very good	Very easy	Very convenient
Good	Easy	Convenient
Good	Very easy	Very convenient
Good	Easy	Convenient
Good	Easy	Convenient
Good	Very easy	Very convenient
Very good	Easy	Very convenient
Good	Very easy	Very convenient
Very good	Very easy	Very convenient
Good	Very easy	Very convenient
Good	Very easy	Very convenient
Good	Very easy	Convenient
Very good	Very easy	Convenient
Good	Very easy	Convenient
Very good	Very easy	Very convenient
Very good	Very easy	Very convenient
Good	Very easy	Convenient
Neutral	Easy	Convenient
Neutral	Easy	Convenient
Good	Very easy	Convenient
Good	Very easy	Very convenient
Good	Very easy	Very convenient
Good	Very easy	Very convenient
Neutral	Easy	Neutral
Very good	Easy	Very convenient

Good	Easy	Convenient
Neutral	Easy	Convenient
Neutral	Easy	Convenient
Good	Easy	Convenient
Good	Very easy	Very convenient
Good	Neutral	Convenient
Bad	Easy	Very convenient
Neutral	Easy	Convenient
Good	Easy	Convenient
Good	Very easy	Convenient
Neutral	Easy	Convenient
Very good	Very easy	Convenient
Good	Very easy	Very convenient
Good	Very easy	Convenient
Good	Easy	Convenient
Good	Easy	Convenient
Neutral	Neutral	Convenient
Neutral	Very easy	Very convenient
Good	Very easy	Very convenient
Very good	Very easy	Very convenient
Neutral	Easy	Convenient
Good	Easy	Convenient
Very good	Very easy	Very convenient
Very good	Very easy	Convenient
Good	Easy	Convenient
Neutral	Easy	Convenient
Good	Easy	Very convenient
Neutral	Easy	Convenient
Good	Hard	Convenient
Good	Easy	Convenient
Good	Easy	Convenient
Neutral	Very easy	Very convenient
Good	Hard	Convenient

Good	Easy	Convenient
Good	Neutral	Neutral
Good	Very easy	Very convenient
Very good	Very easy	Very convenient
Good	Easy	Convenient
Good	Easy	Convenient
Bad	Hard	Convenient
Good	Neutral	Convenient
Neutral	Neutral	Convenient
Good	Very easy	Very convenient
Good	Easy	Convenient
Bad	Easy	Convenient
Good	Easy	Convenient
Very good	Easy	Very convenient
Bad	Hard	Convenient
Neutral	Neutral	Convenient
Good	Easy	Convenient
Good	Easy	Convenient
Bad	Neutral	Convenient
Good	Very easy	Very convenient
Good	Very easy	Very convenient
Very good	Very easy	Convenient
Good	Very easy	Very convenient
Good	Easy	Convenient
Very good	Very easy	Very convenient
Good	Easy	Convenient
Good	Easy	Convenient
Very good	Easy	Convenient
Very good	Very easy	Convenient
Good	Hard	Convenient
Good	Easy	Convenient
Very good	Very easy	Convenient
Good	Neutral	Convenient

Good	Easy	Very convenient
Good	Easy	Convenient
Neutral	Hard	Convenient
Good	Easy	Convenient
Neutral	Easy	Convenient
Very good	Very easy	Convenient
Very good	Easy	Convenient
Good	Easy	Convenient
Very good	Very easy	Very convenient
Bad	Easy	Convenient
Good	Hard	Convenient
Good	Very easy	Convenient
Very good	Very easy	Convenient
Very good	Easy	Convenient
Good	Easy	Very convenient
Very good	Very easy	Convenient
Neutral	Easy	Convenient
Neutral	Hard	Neutral
Neutral	Easy	Convenient
Good	Easy	Convenient
Good	Easy	Convenient
Very good	Easy	Very convenient
Very good	Easy	Very convenient
Very good	Very easy	Very convenient
Good	Easy	Convenient
Bad	Hard	Convenient
Good	Easy	Convenient
Good	Hard	Convenient
Good	Easy	Convenient
Good	Very easy	Very convenient
Good	Easy	Convenient
Good	Easy	Convenient
Good	Very easy	Convenient

Good	Very easy	Very convenient
Neutral	Easy	Very convenient
Good	Easy	Very convenient
Good	Easy	Very convenient
Very good	Very easy	Very convenient
Good	Easy	Convenient
Very good	Very easy	Very convenient
Good	Very easy	Convenient
Good	Easy	Convenient
Good	Very easy	Very convenient
Good	Easy	Convenient
Bad	Hard	Not convenient
Good	Easy	Convenient
Neutral	Easy	Convenient
Neutral	Easy	Convenient
Neutral	Easy	Convenient
Very good	Easy	Very convenient
Good	Very easy	Very convenient
Neutral	Hard	Convenient
Neutral	Easy	Convenient
Neutral	Hard	Convenient
Very good	Easy	Convenient
Good	Very easy	Very convenient
Good	Very easy	Very convenient
Good	Easy	Convenient
Very good	Very easy	Very convenient
Good	Easy	Convenient
Neutral	Easy	Convenient
Neutral	Easy	Very convenient
Very bad	Very hard	Neutral
Very good	Very easy	Very convenient
Very good	Very easy	Very convenient
Good	Easy	Convenient

Very good	Very easy	Very convenient
Neutral	Easy	Very convenient
Very good	Very easy	Very convenient
Good	Easy	Convenient
Good	Easy	Convenient
Neutral	Neutral	Convenient
Good	Easy	Convenient
Good	Very easy	Very convenient
Neutral	Very easy	Very convenient
Bad	Hard	Neutral
Good	Easy	Convenient
Good	Easy	Convenient
Very good	Very easy	Very convenient
Very good	Easy	Convenient
Good	Very easy	Very convenient
Good	Easy	Convenient
Neutral	Hard	Convenient
Good	Easy	Convenient
Good	Easy	Convenient
Neutral	Easy	Very convenient
Good	Easy	Convenient
Very good	Neutral	Neutral
Bad	Hard	Not convenient at all
Good	Very easy	Very convenient
Good	Easy	Convenient
Bad	Hard	Convenient
Very good	Very easy	Very convenient
Neutral	Very easy	Very convenient
Good	Easy	Convenient

Good	Very easy	Convenient
Very good	Very easy	Very convenient
Very good	Very easy	Convenient
Good	Easy	Convenient
Neutral	Easy	Convenient
Very good	Very easy	Convenient
Good	Easy	Convenient
Bad	Easy	Convenient
Neutral	Easy	Convenient
Good	Very easy	Very convenient
Good	Easy	Convenient
Good	Very easy	Very convenient
Good	Very easy	Very convenient
Very good	Very easy	Very convenient
Very good	Very easy	Very convenient
Good	Easy	Very convenient
Very good	Very easy	Very convenient
Good	Very easy	Very convenient
Neutral	Neutral	Neutral
Very good	Very easy	Convenient
Good	Easy	Convenient
Very good	Very easy	Very convenient
Good	Very easy	Very convenient
Very good	Very easy	Very convenient
Neutral	Neutral	Neutral
Neutral	Easy	Convenient
Very good	Very easy	Very convenient
Very good	Very easy	Convenient
Very good	Very easy	Very convenient
Neutral	Easy	Convenient
Neutral	Easy	Neutral
Neutral	Easy	Convenient
Good	Hard	Convenient

Good	Very easy	Very convenient
Neutral	Easy	Convenient
Neutral	Easy	Convenient
Good	Very easy	Very convenient
Neutral	Very easy	Very convenient
Very good	Easy	Very convenient
Neutral	Easy	Convenient
Neutral	Easy	Convenient
Very good	Easy	Convenient
Good	Neutral	Very convenient
Neutral	Easy	Convenient
Neutral	Easy	Convenient
Very good	Easy	Convenient
Good	Very easy	Convenient
Good	Easy	Convenient
Neutral	Easy	Convenient
Bad	Hard	Not convenient
Good	Very easy	Very convenient
Neutral	Easy	Convenient
Good	Easy	Convenient
Bad	Hard	Convenient
Good	Neutral	Convenient
Very good	Very easy	Very convenient
Good	Hard	Convenient
Good	Easy	Convenient
Neutral	Hard	Neutral
Good	Easy	Convenient
Good	Very easy	Convenient
Very good	Very easy	Very convenient
Neutral	Neutral	Convenient
Good	Very easy	Very convenient
Good	Easy	Convenient
Good	Very easy	Convenient

Good	Very easy	Very convenient
Very good	Very easy	Very convenient
Good	Very easy	Convenient
Good	Very easy	Very convenient
Good	Easy	Convenient
Neutral	Easy	Convenient
Good	Very easy	Very convenient
Good	Very easy	Very convenient
Good	Very easy	Very convenient
Neutral	Easy	Convenient
Neutral	Easy	Convenient
Very good	Very easy	Very convenient
Very good	Very easy	Very convenient
Good	Very easy	Very convenient
Good	Easy	Convenient
Neutral	Easy	Very convenient
Good	Easy	Convenient
Good	Very easy	Very convenient
Neutral	Easy	Convenient
Very good	Very easy	Very convenient
Neutral	Easy	Convenient
Neutral	Easy	Convenient
Neutral	Easy	Neutral
Neutral	Very easy	Very convenient
Neutral	Easy	Convenient
Neutral	Hard	Convenient
Good	Very easy	Very convenient
Neutral	Very easy	Very convenient
Neutral	Very easy	Very convenient
Neutral	Easy	Convenient
Good	Very easy	Convenient
Good	Very easy	Very convenient
Good	Easy	Convenient

Good	Easy	Very convenient
Good	Very easy	Very convenient
Very good	Neutral	Very convenient
Good	Very easy	Very convenient
Very good	Very easy	Very convenient
Very good	Very easy	Very convenient
Good	Easy	Convenient
Very good	Very easy	Very convenient
Good	Very easy	Very convenient
Good	Very easy	Very convenient
Good	Neutral	Very convenient
Very good	Very easy	Very convenient
Very good	Very easy	Very convenient
Neutral	Very easy	Very convenient
Very good	Very easy	Very convenient
Very good	Easy	Very convenient
Neutral	Easy	Convenient
Very good	Very easy	Very convenient
Very good	Very easy	Very convenient
Good	Easy	Convenient
Very good	Very easy	Very convenient
Good	Easy	Convenient
Neutral	Very easy	Very convenient
Neutral	Neutral	Neutral
Neutral	Easy	Very convenient
Neutral	Very easy	Convenient
Good	Easy	Very convenient
Very good	Very easy	Very convenient
Neutral	Easy	Convenient
Good	Very easy	Very convenient
Very good	Very easy	Very convenient
Very good	Very easy	Very convenient
Very good	Very easy	Very convenient

Very good	Very easy	Very convenient
Neutral	Easy	Convenient
Very good	Very easy	Very convenient
Neutral	Easy	Convenient
Good	Very easy	Very convenient
Neutral	Very easy	Very convenient
Good	Easy	Convenient
Good	Very easy	Very convenient
Good	Easy	Convenient
Very good	Easy	Convenient
Neutral	Easy	Very convenient
Neutral	Easy	Very convenient
Good	Easy	Very convenient
Very good	Easy	Very convenient
Good	Easy	Very convenient
Very good	Very easy	Very convenient
Good	Very easy	Very convenient
Good	Easy	Convenient
Good	Easy	Convenient
Very good	Very easy	Very convenient
Very good	Easy	Convenient
Good	Easy	Convenient
Very good	Very easy	Very convenient
Very good	Very easy	Very convenient
Very good	Easy	Very convenient
Neutral	Easy	Very convenient
Very good	Very easy	Very convenient
Good	Very easy	Very convenient
Good	Very easy	Very convenient
Very good	Very easy	Very convenient
Good	Easy	Very convenient
Very good	Very easy	Very convenient
Very good	Very easy	Very convenient

Good	Easy	Very convenient
Very good	Very easy	Very convenient
Neutral	Neutral	Convenient
Good	Easy	Convenient
Good	Easy	Convenient
Good	Very easy	Very convenient
Good	Very easy	Very convenient
Good	Easy	Convenient
Very good	Very easy	Very convenient
Very good	Easy	Very convenient
Good	Easy	Convenient
Good	Very easy	Very convenient
Good	Easy	Convenient
Very good	Very easy	Very convenient
Neutral	Easy	Very convenient
Neutral	Neutral	Convenient
Good	Very easy	Convenient
Good	Very easy	Very convenient
Very good	Very easy	Very convenient
Neutral	Hard	Neutral
Neutral	Very easy	Very convenient
Very good	Very easy	Very convenient
Very good	Very easy	Convenient
Neutral	Easy	Convenient
Very good	Very easy	Very convenient
Good	Easy	Convenient
Neutral	Very easy	Very convenient
Good	Very easy	Very convenient
Very good	Very easy	Very convenient
Very good	Very easy	Very convenient
Very good	Easy	Neutral
Good	Neutral	Convenient
Neutral	Easy	Convenient

Very good	Easy	Very convenient
Neutral	Neutral	Convenient
Very good	Very easy	Very convenient
Very good	Very easy	Convenient
Very good	Easy	Convenient
Very good	Very easy	Very convenient
Good	Very easy	Very convenient
Good	Easy	Very convenient
Neutral	Easy	Convenient
Good	Easy	Convenient
Good	Easy	Convenient
Very good	Very easy	Very convenient
Good	Neutral	Convenient
Neutral	Hard	Convenient
Good	Easy	Convenient
Very good	Very easy	Very convenient
Neutral	Neutral	Convenient
Good	Neutral	Convenient
Good	Very easy	Very convenient
Neutral	Easy	Convenient
Good	Very easy	Very convenient
Very good	Very easy	Very convenient
Very good	Very easy	Very convenient
Neutral	Neutral	Convenient
Good	Very easy	Convenient
Very good	Very easy	Very convenient
Very good	Very easy	Very convenient
Good	Easy	Very convenient
Neutral	Very easy	Very convenient
Neutral	Very easy	Very convenient
Very good	Very easy	Very convenient
Neutral	Easy	Neutral
Good	Easy	Convenient

Good	Very easy	Very convenient
Very good	Easy	Very convenient
Good	Easy	Very convenient
Good	Very easy	Convenient
Good	Easy	Convenient
Very good	Very easy	Very convenient
Good	Easy	Not convenient
Good	Very easy	Convenient
Neutral	Easy	Convenient
Neutral	Easy	Convenient
Very good	Very easy	Very convenient
Good	Easy	Convenient
Very good	Very easy	Very convenient
Good	Easy	Convenient
Bad	Neutral	Convenient
Good	Very easy	Very convenient
Very good	Easy	Convenient
Good	Easy	Convenient
Neutral	Neutral	Convenient
Very good	Very easy	Very convenient
Very good	Very easy	Very convenient
Good	Easy	Convenient
Neutral	Easy	Convenient
Very good	Neutral	Very convenient
Good	Very easy	Very convenient
Good	Neutral	Very convenient
Good	Easy	Convenient
Very good	Easy	Convenient
Good	Very easy	Very convenient
Good	Easy	Very convenient
Very good	Very easy	Very convenient

Good	Easy	Convenient
Good	Easy	Convenient
Very good	Very easy	Very convenient
Very good	Very easy	Very convenient
Very good	Easy	Very convenient
Good	Easy	Very convenient
Good	Easy	Convenient
Good	Easy	Convenient
Very good	Very easy	Very convenient
Good	Easy	Convenient
Very good	Very easy	Very convenient
Good	Easy	Convenient
Good	Very easy	Very convenient
Very good	Very easy	Very convenient
Neutral	Easy	Convenient
Good	Easy	Convenient
Good	Easy	Very convenient
Neutral	Easy	Convenient
Good	Easy	Convenient
Good	Easy	Convenient
Good	Very easy	Convenient
Very good	Very easy	Very convenient
Neutral	Easy	Convenient
Good	Easy	Convenient
Very good	Very easy	Very convenient
Good	Easy	Convenient
Good	Easy	Convenient
Neutral	Very hard	Convenient
Neutral	Easy	Convenient
Good	Neutral	Convenient
Neutral	Easy	Convenient
Very good	Easy	Very convenient
Good	Easy	Convenient

Good	Easy	Very convenient
Good	Easy	Very convenient
Neutral	Easy	Not convenient
Good	Easy	Convenient
Good	Easy	Convenient
Good	Easy	Convenient
Neutral	Neutral	Very convenient
Very good	Very easy	Very convenient
Very good	Very easy	Very convenient
Good	Easy	Convenient
Very good	Very easy	Very convenient
Very good	Very easy	Very convenient
Very good	Very easy	Very convenient
Neutral	Very easy	Very convenient
Very good	Very easy	Very convenient
Neutral	Neutral	Convenient
Neutral	Easy	Convenient
Good	Neutral	Convenient
Good	Easy	Convenient
Good	Easy	Convenient
Very good	Very easy	Very convenient
Good	Very easy	Convenient
Very good	Easy	Very convenient
Very good	Very easy	Very convenient
Very good	Very easy	Very convenient
Neutral	Hard	Convenient
Good	Very easy	Convenient
Very good	Very easy	Very convenient
Good	Very easy	Very convenient
Good	Easy	Convenient
Good	Very easy	Very convenient
Good	Easy	Convenient
Good	Easy	Convenient

Good	Easy	Convenient
Good	Easy	Very convenient
Good	Easy	Convenient
Very good	Very easy	Very convenient
Good	Easy	Convenient
Neutral	Easy	Convenient
Good	Easy	Very convenient
Neutral	Easy	Convenient
Neutral	Neutral	Convenient
Very good	Very easy	Very convenient
Very good	Very easy	Very convenient
Neutral	Easy	Very convenient
Good	Very easy	Very convenient
Good	Easy	Convenient
Good	Easy	Convenient
Good	Very easy	Very convenient
Good	Easy	Very convenient
Good	Easy	Convenient
Very good	Very easy	Very convenient
Neutral	Easy	Neutral
Good	Easy	Convenient
Neutral	Easy	Convenient
Good	Easy	Very convenient
Good	Very easy	Very convenient
Very good	Very easy	Very convenient
Good	Very easy	Very convenient
Very good	Easy	Very convenient
Good	Very easy	Very convenient
Neutral	Neutral	Neutral
Good	Easy	Very convenient
Very good	Very easy	Very convenient
Good	Easy	Convenient
Very good	Very easy	Very convenient

Good	Neutral	Convenient
Good	Very easy	Very convenient
Good	Easy	Convenient
Very good	Very easy	Very convenient
Very good	Very easy	Very convenient
Neutral	Very hard	Not convenient
Good	Easy	Convenient
Good	Easy	Convenient
Very good	Very easy	Very convenient
Good	Easy	Very convenient
Good	Very easy	Very convenient
Very good	Very easy	Very convenient
Very good	Very easy	Very convenient
Good	Easy	Convenient
Good	Easy	Convenient
Good	Easy	Convenient
Good	Very easy	Very convenient
Very good	Very easy	Very convenient
Good	Easy	Convenient
Good	Very easy	Very convenient
Good	Easy	Convenient
Neutral	Hard	Convenient
Good	Easy	Convenient
Very good	Very easy	Very convenient
Very good	Very easy	Very convenient
Good	Easy	Very convenient
Very good	Very easy	Very convenient
Good	Easy	Convenient
Very good	Very easy	Very convenient
Very good	Very easy	Very convenient
Good	Easy	Convenient
Neutral	Neutral	Convenient
Good	Easy	Very convenient

Good	Easy	Convenient
Good	Very easy	Very convenient
Very good	Very easy	Very convenient
Neutral	Neutral	Convenient
Very good	Very easy	Very convenient
Good	Easy	Very convenient
Good	Easy	Convenient
Good	Easy	Convenient
Very good	Very easy	Very convenient
Good	Easy	Very convenient
Good	Easy	Convenient
Neutral	Neutral	Convenient
Very good	Very easy	Very convenient
Good	Neutral	Very convenient
Neutral	Easy	Convenient
Neutral	Neutral	Convenient
Good	Easy	Convenient
Good	Easy	Convenient
Very good	Very easy	Very convenient
Very good	Very easy	Very convenient
Good	Easy	Convenient
Very good	Very easy	Very convenient
Neutral	Hard	Convenient
Very good	Neutral	Convenient
Good	Easy	Convenient
Very good	Easy	Convenient
Very good	Very easy	Very convenient
Very good	Easy	Convenient
Good	Easy	Convenient
Good	Very easy	Convenient
Good	Easy	Convenient
Very good	Very easy	Very convenient
Very good	Very easy	Very convenient

Good	Easy	Very convenient
Good	Very easy	Very convenient
Very good	Very easy	Very convenient
Good	Easy	Convenient
Very good	Very easy	Very convenient
Good	Easy	Convenient
Very good	Very easy	Very convenient
Very good	Neutral	Convenient
Very good	Very easy	Convenient
Good	Neutral	Convenient
Good	Easy	Convenient
Good	Very easy	Convenient
Very good	Easy	Very convenient
Very good	Very easy	Very convenient
Very good	Very easy	Very convenient
Neutral	Easy	Convenient
Very good	Very easy	Very convenient
Good	Easy	Convenient
Good	Very easy	Very convenient
Good	Easy	Convenient
Neutral	Neutral	Convenient
Neutral	Neutral	Convenient
Good	Easy	Convenient
Good	Hard	Very convenient
Good	Hard	Convenient
Neutral	Neutral	Convenient
Neutral	Hard	Convenient
Very good	Very easy	Very convenient
Good	Easy	Convenient
Good	Neutral	Convenient
Neutral	Easy	Convenient
Very good	Very easy	Very convenient
Neutral	Easy	Convenient

Neutral	Easy	Convenient
Very good	Very easy	Very convenient
Good	Easy	Convenient
Good	Easy	Convenient
Very good	Easy	Very convenient
Very good	Very easy	Very convenient
Very good	Very easy	Very convenient
Neutral	Easy	Convenient
Very good	Very easy	Very convenient
Very good	Very easy	Very convenient
Neutral	Very easy	Very convenient
Good	Easy	Very convenient
Good	Very easy	Convenient
Very good	Very easy	Very convenient
Good	Easy	Convenient
Good	Very easy	Very convenient
Very good	Very easy	Very convenient
Good	Neutral	Convenient
Good	Very easy	Very convenient
Very good	Very easy	Very convenient
Good	Neutral	Convenient
Neutral	Easy	Convenient
Very good	Very easy	Very convenient
Good	Easy	Very convenient
Very good	Very easy	Very convenient
Good	Easy	Very convenient
Very good	Very easy	Very convenient
Good	Easy	Convenient
Very good	Very easy	Very convenient
Very good	Easy	Convenient
Very good	Easy	Very convenient
Good	Hard	Neutral
Good	Easy	Very convenient

Neutral	Easy	Convenient
Good	Very easy	Very convenient
Good	Easy	Convenient
Neutral	Neutral	Convenient
Good	Easy	Convenient
Very good	Very easy	Very convenient
Good	Neutral	Convenient
Neutral	Hard	Convenient
Neutral	Hard	Not convenient
Neutral	Neutral	Convenient
Good	Easy	Convenient
Good	Easy	Very convenient
Good	Easy	Convenient
Very good	Very easy	Very convenient
Very good	Easy	Convenient
Very good	Very easy	Very convenient
Good	Easy	Convenient
Good	Very easy	Very convenient
Good	Very easy	Very convenient
Very good	Very easy	Very convenient
Very good	Very easy	Very convenient
Good	Very easy	Very convenient
Very good	Very easy	Very convenient
Good	Easy	Convenient
Good	Easy	Convenient
Very good	Very easy	Very convenient
Neutral	Easy	Convenient
Good	Easy	Convenient
Very good	Very easy	Very convenient
Very good	Very easy	Very convenient
Very good	Very easy	Very convenient
Very good	Very easy	Very convenient

Good	Easy	Convenient
Very good	Very easy	Very convenient
Neutral	Easy	Convenient
Neutral	Easy	Very convenient
Good	Easy	Convenient
Neutral	Easy	Convenient
Good	Hard	Convenient
Good	Easy	Convenient
Good	Easy	Convenient
Very good	Very easy	Very convenient
Very good	Easy	Convenient
Good	Easy	Very convenient
Very good	Very easy	Very convenient
Very good	Very easy	Very convenient
Neutral	Easy	Convenient
Very good	Very easy	Very convenient
Good	Very easy	Very convenient
Good	Easy	Very convenient
Very good	Very easy	Very convenient
Very good	Very easy	Very convenient
Very good	Very easy	Very convenient
Very good	Very easy	Very convenient
Good	Easy	Convenient
Good	Hard	Convenient
Very good	Very easy	Very convenient
Good	Easy	Very convenient
Good	Easy	Convenient
Very good	Very easy	Convenient
Very good	Very easy	Very convenient
Good	Easy	Convenient
Good	Easy	Convenient
Neutral	Easy	Convenient

Good	Neutral	Convenient
Good	Easy	Convenient
Neutral	Easy	Convenient
Good	Easy	Convenient
Good	Easy	Convenient
Good	Easy	Convenient
Good	Easy	Very convenient
Very good	Very easy	Very convenient
Very good	Very easy	Very convenient
Good	Easy	Convenient
Very good	Very easy	Very convenient
Neutral	Easy	Convenient
Good	Neutral	Convenient
Good	Very easy	Convenient
Very good	Very easy	Very convenient
Good	Easy	Convenient
Good	Very easy	Very convenient
Good	Neutral	Very convenient
Good	Easy	Very convenient
Neutral	Neutral	Neutral
Good	Very easy	Very convenient
Very good	Very easy	Very convenient
Good	Very easy	Very convenient
Good	Easy	Convenient
Good	Neutral	Convenient
Good	Very easy	Very convenient
Very good	Easy	Convenient
Good	Easy	Very convenient
Very good	Very easy	Very convenient
Neutral	Easy	Very convenient
Very good	Very easy	Very convenient
Very good	Very easy	Very convenient
Very good	Very easy	Very convenient

Good	Easy	Convenient
Good	Easy	Convenient
Bad	Hard	Not convenient
Neutral	Easy	Convenient
Bad	Hard	Convenient
Very good	Very easy	Very convenient
Neutral	Neutral	Convenient
Good	Easy	Convenient
Very good	Very easy	Very convenient
Good	Easy	Convenient
Good	Easy	Convenient
Neutral	Hard	Convenient
Good	Easy	Very convenient
Very good	Very easy	Very convenient
Very good	Very easy	Very convenient
Very good	Very easy	Very convenient
Very good	Very easy	Very convenient
Neutral	Easy	Convenient
Very good	Easy	Very convenient
Very good	Very easy	Very convenient
Good	Easy	Convenient
Good	Easy	Convenient
Very good	Very easy	Very convenient
Good	Very easy	Very convenient
Good	Easy	Neutral
Very good	Very easy	Very convenient
Neutral	Easy	Convenient
Very good	Very easy	Convenient
Good	Easy	Convenient
Good	Very easy	Very convenient
Good	Easy	Convenient
Very good	Very easy	Very convenient
Neutral	Easy	Convenient

Neutral	Very easy	Very convenient
Neutral	Neutral	Neutral
Neutral	Hard	Convenient
Very good	Very easy	Very convenient
Good	Easy	Convenient
Neutral	Easy	Neutral
Very good	Very easy	Very convenient
Very good	Very easy	Very convenient
Neutral	Easy	Convenient
Good	Easy	Convenient
Neutral	Hard	Convenient
Neutral	Neutral	Convenient
Good	Easy	Convenient
Neutral	Easy	Convenient
Good	Easy	Convenient
Neutral	Easy	Convenient
Good	Easy	Convenient
Good	Easy	Convenient
Good	Very easy	Very convenient
Good	Neutral	Convenient
Good	Easy	Convenient
Good	Easy	Convenient
Good	Neutral	Convenient
Good	Easy	Very convenient
Very good	Very easy	Very convenient
Good	Easy	Convenient
Good	Easy	Convenient
Good	Easy	Very convenient
Very good	Very easy	Very convenient
Good	Easy	Convenient
Good	Easy	Convenient
Good	Very easy	Very convenient
Very good	Very easy	Very convenient

Good	Very easy	Convenient
Good	Easy	Convenient
Neutral	Easy	Convenient
Very good	Very easy	Very convenient
Good	Easy	Convenient
Good	Easy	Convenient
Good	Easy	Very convenient
Good	Easy	Convenient
Good	Easy	Convenient
Good	Neutral	Convenient
Good	Easy	Convenient
Good	Easy	Convenient
Good	Easy	Convenient
Good	Easy	Convenient
Good	Easy	Convenient
Good	Very easy	Very convenient
Good	Easy	Convenient
Good	Easy	Convenient
Neutral	Easy	Convenient
Good	Easy	Convenient
Very good	Very easy	Very convenient
Good	Easy	Convenient
Very good	Very easy	Very convenient
Good	Easy	Convenient
Good	Easy	Convenient
Good	Easy	Convenient
Very good	Very easy	Very convenient
Very good	Very easy	Very convenient
Good	Easy	Convenient
Good	Easy	Convenient
Very good	Very easy	Very convenient
Good	Easy	Convenient
Good	Easy	Convenient

Very good	Very easy	Very convenient
Good	Very easy	Very convenient
Very good	Very easy	Very convenient
Very good	Very easy	Very convenient
Neutral	Hard	Convenient
Good	Neutral	Convenient
Very good	Very easy	Very convenient
Good	Easy	Convenient
Good	Easy	Convenient
Very good	Very easy	Very convenient
Neutral	Neutral	Convenient
Neutral	Neutral	
Neutral	Neutral	Convenient
Good	Easy	Very convenient
Good	Easy	Convenient
Neutral	Easy	Convenient
Good	Easy	Convenient
Good	Easy	Convenient
Very good	Very easy	Very convenient
Very good	Very easy	Very convenient
Good	Easy	Convenient
Bad	Very easy	Very convenient
Good	Easy	Very convenient
Good	Easy	Convenient
Good	Very easy	Very convenient
Good	Easy	Very convenient
Very good	Very easy	Very convenient
Very good	Easy	Convenient
Good	Easy	Convenient
Good	Easy	Convenient
Good	Hard	Convenient
Neutral	Easy	Convenient
Good	Easy	Convenient

Neutral	Hard	Convenient
Good	Easy	Convenient
Good	Easy	Convenient
Bad	Hard	Not convenient
Good	Easy	Convenient
Good	Easy	Convenient
Good	Easy	Very convenient
Neutral	Hard	Very convenient
Very good	Very easy	Very convenient
Good	Very easy	Very convenient
Good	Easy	Not convenient
Neutral	Easy	Convenient
Good	Easy	Convenient
Very good	Very easy	Very convenient
Very good	Very easy	Very convenient
Neutral	Easy	Convenient
Good	Easy	Convenient
Good	Very easy	Very convenient
Good	Neutral	Convenient
Good	Very easy	Very convenient
Good	Easy	Convenient
Good	Easy	Convenient
Good	Very easy	Very convenient
Good	Easy	Convenient
Very good	Easy	Convenient
Neutral	Very easy	Very convenient
Good	Easy	Convenient
Neutral	Easy	Convenient
Very good	Very easy	Very convenient
Good	Easy	Convenient
Good	Easy	Convenient
Very good	Very easy	Very convenient

Very good	Very easy	Very convenient
Good	Very easy	Very convenient
Good	Easy	Convenient
Good	Easy	Convenient
Very good	Very easy	Very convenient
Very good	Easy	Convenient
Neutral	Easy	Convenient
Very good	Very easy	Convenient
Very good	Very easy	Very convenient
Very good	Easy	Very convenient
Good	Easy	Convenient
Neutral	Neutral	Neutral
Good	Easy	Convenient
Very good	Very easy	Very convenient
Neutral	Easy	Convenient
Very good	Very easy	Very convenient
Very good	Very easy	Convenient
Good	Easy	Convenient
Good	Easy	Convenient
Neutral	Neutral	Convenient
Good	Very easy	Very convenient
Good	Very easy	Very convenient
Good	Easy	Convenient
Good	Easy	Convenient
Neutral	Neutral	Convenient
Good	Easy	Convenient
Good	Easy	Convenient
Good	Easy	Convenient
Neutral	Neutral	Convenient
Neutral	Very easy	Very convenient
Neutral	Neutral	Convenient
Good	Easy	Convenient
Very good	Very easy	Very convenient

Very good	Easy	Convenient
Good	Easy	Convenient
Good	Very easy	Very convenient
Good	Easy	Convenient
Good	Easy	Convenient
Very good	Very easy	Very convenient
Good	Very easy	Very convenient
Neutral	Very easy	Convenient
Good	Easy	Convenient
Good	Easy	Convenient
Neutral	Hard	Neutral
Good	Neutral	Convenient
Good	Easy	Convenient
Good	Easy	Convenient
Neutral	Easy	Convenient
Good	Easy	Convenient
Neutral	Hard	Convenient
Very good	Very easy	Very convenient
Good	Very easy	Very convenient
Good	Easy	Convenient
Very good	Easy	Very convenient
Good	Easy	Convenient
Very good	Very easy	Very convenient
Bad	Hard	Convenient
Very good	Very easy	Very convenient
Very good	Easy	Convenient
Good	Very easy	Very convenient
Good	Easy	Convenient
Good	Easy	Convenient
Good	Easy	Convenient
Very good	Easy	Convenient
Good	Easy	Convenient
Very good	Easy	Convenient

Good	Easy	Convenient
Good	Easy	Convenient
Very good	Very easy	Very convenient
Good	Very easy	Very convenient
Good	Easy	Convenient
Good	Easy	Convenient
Very good	Very easy	Very convenient
Good	Easy	Convenient
Neutral	Neutral	Convenient
Good	Easy	Convenient
Very good	Very easy	Convenient
Good	Very easy	Convenient
Good	Easy	Convenient
Good	Easy	Convenient
Very good	Very easy	Very convenient
Good	Easy	Convenient
Neutral	Neutral	Convenient
Very good	Very easy	Very convenient
Good	Easy	Convenient
Good	Easy	Convenient
Good	Easy	Convenient
Good	Easy	Convenient
Good	Very easy	Very convenient
Good	Easy	Convenient
Good	Very easy	Very convenient
Bad	Hard	Convenient
Good	Easy	Convenient
Very good	Very easy	Very convenient
Very good	Very easy	Very convenient
Very good	Very easy	Very convenient
Very good	Very easy	Very convenient
Good	Easy	Convenient
Good	Easy	Convenient

Very good	Very easy	Very convenient
Good	Very easy	Very convenient
Good	Easy	Convenient
Neutral	Easy	Convenient
Neutral	Neutral	Convenient
Good	Easy	Convenient
Good	Easy	Convenient
Good	Easy	Convenient
Good	Very easy	Convenient
Good	Very easy	Very convenient
Good	Easy	Very convenient
Bad	Neutral	Convenient
Good	Very easy	Convenient
Very good	Very easy	Very convenient
Good	Easy	Convenient
Good	Easy	Convenient
Very good	Very easy	Very convenient
Very good	Very easy	Very convenient
Good	Easy	Convenient
Very good	Very easy	Very convenient
Good	Easy	Convenient
Good	Very easy	Very convenient
Good	Easy	Very convenient
Very good	Easy	Convenient
Bad	Neutral	Not convenient
Good	Easy	Convenient
Neutral	Easy	Convenient
Good	Easy	Convenient
Good	Very easy	Convenient
Good	Easy	Convenient
Very good	Easy	Convenient
Good	Easy	Convenient
Very good	Easy	Very convenient

Good	Easy	Convenient
Neutral	Easy	Convenient
Neutral	Easy	Convenient
Good	Neutral	Convenient
Good	Easy	Convenient
Good	Easy	Not convenient
Very good	Very easy	Very convenient
Good	Easy	Convenient
Good	Very easy	Very convenient
Very good	Very easy	Very convenient
Very good	Easy	Very convenient
Good	Easy	Convenient
Very good	Very easy	Very convenient
Good	Easy	Convenient
Good	Very easy	Very convenient
Very good	Very easy	Very convenient
Good	Easy	Convenient
Good	Easy	Convenient
Neutral	Easy	Convenient
Good	Easy	Convenient
Bad	Easy	Not convenient
Good	Very easy	Very convenient
Good	Easy	Convenient
Very good	Very easy	Very convenient
Good	Easy	Convenient
Very good	Easy	Very convenient
Very good	Easy	Very convenient
Good	Easy	Convenient
Very good	Easy	Convenient
Good	Easy	Convenient
Good	Easy	Very convenient
Good	Very easy	Very convenient
Good	Easy	Convenient

Neutral	Easy	Convenient
Good	Easy	Convenient
Good	Easy	Convenient
Good	Easy	Convenient
Very good	Very easy	Very convenient
Good	Easy	Convenient
Good	Easy	Convenient
Good	Easy	Convenient
Good	Easy	Convenient
Very good	Easy	Convenient
Good	Easy	Convenient
Good	Easy	Convenient
Good	Easy	Convenient
Very good	Very easy	Very convenient
Good	Easy	Convenient
Good	Easy	Convenient
Good	Easy	Convenient
Neutral	Neutral	Neutral
Very good	Very easy	Very convenient
Good	Easy	Convenient
Good	Easy	Very convenient
Good	Easy	Convenient
Good	Easy	Convenient
Very good	Very easy	Very convenient
Good	Easy	Convenient
Good	Easy	Convenient
Good	Easy	Convenient
Good	Neutral	Convenient
Good	Easy	Convenient
Good	Easy	Convenient
Good	Easy	Convenient
Good	Easy	Convenient
Very good	Very easy	Very convenient

Good	Easy	Convenient
Good	Very easy	Convenient
Very good	Easy	Convenient
Good	Easy	Convenient
Good	Easy	Convenient
Good	Easy	Convenient
Good	Easy	Convenient
Good	Easy	Convenient
Neutral	Easy	Convenient
Good	Easy	Convenient
Good	Easy	Convenient
Good	Easy	Convenient
Good	Easy	Convenient
Good	Easy	Convenient
Good	Easy	Convenient
Very good	Easy	Convenient
Very good	Very easy	Very convenient
Bad	Hard	Not convenient
Good	Easy	Very convenient
Neutral	Easy	Convenient
Good	Easy	Convenient
Good	Easy	Convenient
Good	Easy	Very convenient
Very good	Easy	Convenient
Good	Easy	Convenient
Good	Easy	Very convenient
Neutral	Hard	Not convenient
Good	Easy	Neutral
Good	Easy	Convenient
Good	Easy	Convenient
Very good	Easy	Convenient

Good	Easy	Convenient
Very good	Very easy	Very convenient
Bad	Hard	Not convenient
Good	Easy	Convenient
Very good	Very easy	Convenient
Neutral	Easy	Convenient
Good	Easy	Convenient
Good	Very easy	Convenient
Good	Easy	Convenient
Good	Easy	Convenient
Good	Easy	Convenient
Good	Easy	Convenient
Very good	Very easy	Convenient
Neutral	Easy	Convenient
Good	Neutral	Convenient
Good	Easy	Very convenient
Good	Easy	Convenient
Good	Easy	Convenient
Good	Easy	Convenient
Good	Easy	Convenient
Good	Easy	Convenient
Good	Easy	Convenient
Good	Easy	Convenient
Good	Neutral	Convenient
Good	Easy	Convenient
Bad	Hard	Convenient
Good	Easy	Convenient
Good	Easy	Convenient
Good	Easy	Convenient
Good	Hard	Not convenient
Good	Easy	Convenient
Good	Easy	Convenient

Good	Neutral	Convenient
Good	Very easy	Very convenient
Good	Hard	Convenient
Good	Easy	Very convenient
Good	Easy	Convenient
Good	Easy	Very convenient
Good	Easy	Convenient
Very good	Easy	Convenient
Neutral	Hard	Not convenient
Very good	Easy	Convenient
Good	Easy	Convenient
Good	Easy	Convenient
Good	Easy	Convenient
Good	Easy	Convenient
Very good	Very easy	Very convenient
Neutral	Hard	Not convenient
Good	Very easy	Convenient
Good	Easy	Convenient
Good	Easy	Convenient
Very good	Very easy	Very convenient
Very good	Easy	Convenient
Good	Easy	Convenient
Neutral	Easy	Convenient
Very good	Very easy	Very convenient
Good	Easy	Convenient
Bad	Hard	Not convenient
Very good	Easy	Convenient
Good	Easy	Convenient
Good	Easy	Convenient
Good	Hard	Convenient
Very good	Very easy	Very convenient
Good	Easy	Convenient
Very good	Easy	Very convenient

Good	Very easy	Convenient
Good	Very easy	Convenient
Very good	Very easy	Very convenient
Neutral	Hard	Convenient
Neutral	Very easy	Very convenient
Neutral	Easy	Convenient
Good	Easy	Convenient
Good	Easy	Convenient
Good	Easy	Convenient
Good	Easy	Convenient
Good	Easy	Convenient
Good	Neutral	Very convenient
Good	Easy	Convenient
Good	Neutral	Convenient
Good	Easy	Convenient
Good	Easy	Convenient
Neutral	Easy	Convenient
Good	Easy	Convenient
Good	Easy	Convenient
Good	Neutral	Not convenient
Good	Easy	Very convenient
Good	Easy	Convenient
Good	Easy	Convenient
Good	Easy	Convenient
Very good	Easy	Convenient
Good	Neutral	Not convenient
Very good	Easy	Convenient
Very good	Easy	Convenient
Good	Easy	Convenient
Good	Easy	Convenient
Very good	Easy	Very convenient
Good	Easy	Convenient
Neutral	Easy	Convenient

Very good	Easy	Very convenient
Good	Easy	Convenient
Very good	Very easy	Very convenient
Good	Easy	Convenient
Very good	Very easy	Very convenient
Good	Easy	Convenient
Good	Easy	Convenient
Good	Easy	Convenient
Good	Easy	Convenient
Good	Easy	Convenient
Good	Easy	Convenient
Very good	Very easy	Very convenient
Good	Easy	Convenient
Very good	Easy	Convenient
Very good	Easy	Very convenient
Good	Easy	Convenient
Good	Hard	Not convenient
Neutral	Neutral	Convenient
Good	Easy	Convenient
Very good	Very easy	Convenient
Very good	Very easy	Very convenient
Very good	Easy	Convenient
Very good	Very easy	Very convenient
Very good	Very easy	Very convenient
Very good	Easy	Convenient
Good	Easy	Convenient
Good	Very easy	Convenient
Good	Easy	Convenient
Good	Easy	Convenient
Good	Easy	Convenient
Good	Very easy	Convenient

Good	Very easy	Convenient
Good	Easy	Convenient
Good	Very easy	Convenient
Very good	Easy	Convenient
Very good	Very easy	Very convenient
Very good	Easy	Convenient
Very good	Very easy	Very convenient
Very good	Very easy	Very convenient
Very good	Very easy	Convenient
Very good	Very easy	Convenient
Good	Easy	Convenient
Very good	Very easy	Very convenient
Very good	Very easy	Very convenient
Good	Very easy	Convenient
Neutral	Easy	Convenient
Very good	Very easy	Convenient
Good	Easy	Convenient
Very good	Very easy	Very convenient
Good	Easy	Convenient
Good	Easy	Very convenient
Very good	Very easy	Very convenient
Very good	Very easy	Convenient
Very good	Very easy	Very convenient
Good	Very easy	Very convenient
Very good	Very easy	Very convenient
Good	Easy	Convenient
Very good	Very easy	Convenient
Very good	Very easy	Convenient
Very good	Very easy	Very convenient
Good	Very easy	Convenient
Very good	Very easy	Very convenient
Good	Very easy	Convenient

Good	Easy	Convenient
Very good	Very easy	Convenient
Good	Very easy	Very convenient
Good	Very easy	Convenient
Very good	Easy	Convenient
Very good	Very easy	Very convenient
Very good	Very easy	Convenient
Good	Easy	Convenient
Good	Neutral	Convenient
Good	Very easy	Convenient
Very good	Very easy	Convenient
Good	Easy	Convenient
Very good	Very easy	Convenient
Good	Easy	Convenient
Good	Very easy	Convenient
Very good	Very easy	Very convenient
Neutral	Easy	Not convenient
Very good	Very easy	Convenient
Good	Neutral	Convenient
Good	Easy	Not convenient

How embarrassed were you to collect this self-sample?	How much discomfort/pain did you experience while coll	How confident are you that you collected this self-sample
Not embarrassed	No discomfort/pain	Confident
Not embarrassed	No discomfort/pain	Confident
Not embarrassed at all	No discomfort/pain at all	Confident
Not embarrassed at all	Neutral	Confident
Not embarrassed at all	No discomfort/pain at all	Very confident
Not embarrassed	No discomfort/pain at all	Neutral
Not embarrassed	Some discomfort/pain	Confident
Not embarrassed at all	No discomfort/pain	Very confident
Not embarrassed at all	No discomfort/pain at all	Very confident
Not embarrassed	Some discomfort/pain	Not confident
Not embarrassed at all	No discomfort/pain at all	Very confident
Not embarrassed	Neutral	Confident
Not embarrassed at all	Some discomfort/pain	Confident
Not embarrassed at all	No discomfort/pain at all	Confident
Not embarrassed at all	No discomfort/pain at all	Confident
Not embarrassed at all	No discomfort/pain at all	Neutral
Not embarrassed at all	Some discomfort/pain	Neutral
Not embarrassed at all	No discomfort/pain	Neutral
Not embarrassed at all	Some discomfort/pain	Neutral
Not embarrassed at all	No discomfort/pain at all	Confident
Not embarrassed at all	Some discomfort/pain	Confident
Not embarrassed at all	No discomfort/pain	Confident
Not embarrassed	Neutral	Not confident
Not embarrassed	Some discomfort/pain	Not confident
Not embarrassed at all	Neutral	Confident
Not embarrassed	No discomfort/pain	Confident
Not embarrassed at all	No discomfort/pain at all	Very confident
Not embarrassed at all	No discomfort/pain	Confident
Not embarrassed at all	No discomfort/pain at all	Very confident
Not embarrassed at all	Some discomfort/pain	Confident
Not embarrassed at all	Some discomfort/pain	Confident

Not embarrassed at all	No discomfort/pain at all	Confident
Not embarrassed at all	No discomfort/pain at all	Confident
Not embarrassed at all	Some discomfort/pain	Confident
Not embarrassed at all	No discomfort/pain	Confident
Not embarrassed	Some discomfort/pain	Confident
Not embarrassed	Neutral	Confident
Not embarrassed	No discomfort/pain	Confident
Not embarrassed	No discomfort/pain at all	Confident
Not embarrassed	No discomfort/pain at all	Confident
Not embarrassed at all	No discomfort/pain at all	Confident
Not embarrassed	Some discomfort/pain	Not confident
Not embarrassed at all	Some discomfort/pain	Confident
Not embarrassed at all	No discomfort/pain at all	Very confident
Not embarrassed at all	No discomfort/pain	Confident
Not embarrassed at all	No discomfort/pain at all	Confident
Not embarrassed	No discomfort/pain at all	Confident
Not embarrassed at all	No discomfort/pain at all	Very confident
Not embarrassed at all	No discomfort/pain	Confident
Not embarrassed	No discomfort/pain at all	Confident
Not embarrassed at all	Neutral	Very confident
Not embarrassed	Some discomfort/pain	Confident
Not embarrassed	No discomfort/pain at all	Confident
Not embarrassed	Neutral	Confident
Not embarrassed at all	No discomfort/pain	Confident
Not embarrassed at all	Some discomfort/pain	Confident
Not embarrassed at all	No discomfort/pain at all	Very confident
Not embarrassed at all	Some discomfort/pain	Neutral
Not embarrassed	No discomfort/pain	Confident
Not embarrassed	No discomfort/pain	Neutral
Not embarrassed at all	No discomfort/pain at all	Very confident

Not embarrassed at all	No discomfort/pain at all	Very confident
Not embarrassed	Neutral	Confident
Not embarrassed at all	Some discomfort/pain	Not confident
Not embarrassed at all	No discomfort/pain	Very confident
Not embarrassed at all	No discomfort/pain	Confident
Not embarrassed at all	No discomfort/pain at all	Very confident
Not embarrassed at all	No discomfort/pain at all	Confident
Not embarrassed at all	Some discomfort/pain	Not confident
Not embarrassed at all	No discomfort/pain at all	Very confident
Not embarrassed at all	No discomfort/pain at all	Very confident
Not embarrassed at all	No discomfort/pain at all	Very confident
Not embarrassed at all	Some discomfort/pain	Confident
Not embarrassed at all	Some discomfort/pain	Confident
Not embarrassed	Some discomfort/pain	Confident
Not embarrassed at all	No discomfort/pain at all	Very confident
Not embarrassed at all	No discomfort/pain at all	Very confident
Not embarrassed at all	No discomfort/pain at all	Very confident
Not embarrassed at all	No discomfort/pain at all	Very confident
Not embarrassed at all	No discomfort/pain at all	Confident
Not embarrassed at all	No discomfort/pain	Confident
Not embarrassed at all	Some discomfort/pain	Confident
Not embarrassed at all	No discomfort/pain at all	Not confident
Not embarrassed at all	No discomfort/pain	Neutral
Not embarrassed at all	No discomfort/pain	Very confident
Not embarrassed at all	No discomfort/pain	Not confident
Very embarrassed	No discomfort/pain at all	Very confident
Not embarrassed at all	Some discomfort/pain	Confident
Not embarrassed at all	Some discomfort/pain	Confident
Not embarrassed at all	Some discomfort/pain	Confident
Not embarrassed at all	No discomfort/pain at all	Very confident
Not embarrassed at all	No discomfort/pain at all	Very confident
Not embarrassed at all	No discomfort/pain	Very confident
Not embarrassed at all	Some discomfort/pain	Very confident

Not embarrassed	No discomfort/pain	Confident
Not embarrassed at all	No discomfort/pain at all	Very confident
Not embarrassed at all	Neutral	Confident
Not embarrassed at all	Some discomfort/pain	Confident
Not embarrassed at all	Some discomfort/pain	Confident
Not embarrassed at all	No discomfort/pain at all	Confident
Not embarrassed at all	Some discomfort/pain	Confident
Not embarrassed at all	No discomfort/pain at all	Confident
Not embarrassed at all	No discomfort/pain at all	Very confident
Not embarrassed at all	No discomfort/pain	Confident
Not embarrassed at all	No discomfort/pain	Neutral
Not embarrassed at all	Neutral	Confident
Not embarrassed at all	No discomfort/pain	Very confident
Not embarrassed at all	No discomfort/pain at all	Very confident
Not embarrassed at all	No discomfort/pain at all	Very confident
Not embarrassed at all	Some discomfort/pain	Confident
Not embarrassed at all	No discomfort/pain at all	Confident
Not embarrassed at all	Neutral	Confident
Not embarrassed at all	Some discomfort/pain	Confident
Not embarrassed at all	No discomfort/pain	Confident
Not embarrassed at all	Some discomfort/pain	Confident
Not embarrassed at all	No discomfort/pain	Confident
Not embarrassed at all	No discomfort/pain	Very confident
Not embarrassed at all	Some discomfort/pain	Neutral
Not embarrassed at all	No discomfort/pain at all	Very confident
Not embarrassed at all	Neutral	Confident
Not embarrassed at all	No discomfort/pain at all	Very confident
Not embarrassed at all	No discomfort/pain	Very confident
Not embarrassed at all	No discomfort/pain at all	Very confident
Not embarrassed at all	No discomfort/pain at all	Very confident
Embarrassed	No discomfort/pain	Confident
Not embarrassed at all	No discomfort/pain at all	Very confident
Not embarrassed at all	No discomfort/pain	Confident

Not embarrassed at all	No discomfort/pain at all	Very confident
Not embarrassed at all	No discomfort/pain at all	Very confident
Not embarrassed at all	No discomfort/pain at all	Very confident
Not embarrassed at all	No discomfort/pain	Confident
Not embarrassed at all	Some discomfort/pain	Confident
Not embarrassed	Neutral	Not confident
Not embarrassed at all	No discomfort/pain at all	Confident
Not embarrassed	Some discomfort/pain	Neutral
Not embarrassed at all	No discomfort/pain at all	Very confident
Not embarrassed at all	Some discomfort/pain	Not confident
Not embarrassed at all	Some discomfort/pain	Very confident
Not embarrassed at all	No discomfort/pain at all	Very confident
Not embarrassed at all	Some discomfort/pain	Confident
Not embarrassed at all	No discomfort/pain at all	Confident
Not embarrassed at all	No discomfort/pain at all	Very confident
Not embarrassed at all	No discomfort/pain	Confident
Not embarrassed at all	No discomfort/pain at all	Confident
Not embarrassed at all	No discomfort/pain	Confident
Not embarrassed at all	No discomfort/pain	Confident
Not embarrassed at all	No discomfort/pain	Confident
Not embarrassed	Neutral	Confident
Not embarrassed	No discomfort/pain	Confident
Not embarrassed at all	No discomfort/pain at all	Very confident
Not embarrassed at all	No discomfort/pain at all	Very confident
Not embarrassed at all	Neutral	Very confident
Not embarrassed at all	Neutral	Very confident
Not embarrassed at all	No discomfort/pain at all	Very confident
Not embarrassed at all	No discomfort/pain	Confident
Not embarrassed	No discomfort/pain	Confident
Not embarrassed at all	Neutral	Neutral
Not embarrassed at all	Some discomfort/pain	Confident
Not embarrassed at all	Some discomfort/pain	Confident
Not embarrassed at all	Some discomfort/pain	Confident

Not embarrassed at all	No discomfort/pain at all	Very confident
Not embarrassed at all	No discomfort/pain at all	Very confident
Not embarrassed at all	Some discomfort/pain	Neutral
Not embarrassed at all	Some discomfort/pain	Confident
Embarrassed	No discomfort/pain at all	Confident
Not embarrassed at all	No discomfort/pain at all	Confident
Not embarrassed at all	No discomfort/pain at all	Confident
Not embarrassed at all	Neutral	Not confident
Not embarrassed	Some discomfort/pain	Not confident
Not embarrassed at all	Some discomfort/pain	Neutral
Not embarrassed at all	Some discomfort/pain	Confident
Not embarrassed at all	No discomfort/pain at all	Confident
Not embarrassed at all	Some discomfort/pain	Confident
Not embarrassed at all	Some discomfort/pain	Confident
Not embarrassed at all	Some discomfort/pain	Confident
Not embarrassed at all	No discomfort/pain at all	Very confident
Not embarrassed at all	Some discomfort/pain	Confident
Not embarrassed at all	No discomfort/pain at all	Very confident
Not embarrassed at all	No discomfort/pain	Very confident
Not embarrassed	No discomfort/pain at all	Very confident
Not embarrassed at all	Some discomfort/pain	Confident
Not embarrassed at all	No discomfort/pain	Very confident
Not embarrassed at all	No discomfort/pain at all	Very confident
Not embarrassed at all	No discomfort/pain at all	Neutral
Not embarrassed at all	Some discomfort/pain	Very confident
Not embarrassed at all	Some discomfort/pain	Confident
Not embarrassed at all	No discomfort/pain	Very confident
Not embarrassed at all	No discomfort/pain	Confident
Not embarrassed at all	No discomfort/pain	Very confident
Not embarrassed at all	No discomfort/pain at all	Very confident
Not embarrassed at all	Some discomfort/pain	Very confident
Not embarrassed at all	No discomfort/pain at all	Confident
Not embarrassed at all	No discomfort/pain at all	Very confident

Not embarrassed at all	No discomfort/pain	Confident
Not embarrassed at all	Neutral	Very confident
Not embarrassed at all	No discomfort/pain at all	Very confident
Not embarrassed at all	No discomfort/pain	Very confident
Not embarrassed at all	No discomfort/pain at all	Very confident
Not embarrassed at all	Some discomfort/pain	Confident
Not embarrassed at all	No discomfort/pain at all	Very confident
Not embarrassed at all	No discomfort/pain at all	Confident
Not embarrassed at all	No discomfort/pain at all	Very confident
Not embarrassed at all	Some discomfort/pain	Confident
Not embarrassed at all	No discomfort/pain at all	Very confident
Not embarrassed at all	Some discomfort/pain	Very confident
Not embarrassed at all	Neutral	Very confident
Not embarrassed at all	No discomfort/pain at all	Very confident
Not embarrassed at all	No discomfort/pain at all	Very confident
Not embarrassed at all	Some discomfort/pain	Confident
Not embarrassed at all	Some discomfort/pain	Confident
Not embarrassed	No discomfort/pain at all	Very confident
Not embarrassed at all	No discomfort/pain at all	Very confident
Not embarrassed at all	No discomfort/pain	Neutral
Not embarrassed at all	Neutral	Confident
Not embarrassed at all	Neutral	Confident
Not embarrassed at all	No discomfort/pain at all	Confident
Not embarrassed at all	No discomfort/pain at all	Very confident
Not embarrassed at all	No discomfort/pain	Confident
Not embarrassed at all	Some discomfort/pain	Very confident
Not embarrassed at all	No discomfort/pain at all	Very confident
Not embarrassed at all	Neutral	Confident
Not embarrassed at all	Some discomfort/pain	Confident
Not embarrassed at all	Some discomfort/pain	Very confident
Not embarrassed at all	Some discomfort/pain	Very confident
Not embarrassed at all	Some discomfort/pain	Very confident
Not embarrassed at all	No discomfort/pain	Confident

Not embarrassed at all	No discomfort/pain at all	Very confident
Not embarrassed at all	No discomfort/pain	Very confident
Not embarrassed at all	Some discomfort/pain	Confident
Not embarrassed at all	No discomfort/pain at all	Very confident
Not embarrassed at all	No discomfort/pain	Very confident
Not embarrassed at all	Some discomfort/pain	Very confident
Not embarrassed at all	No discomfort/pain	Very confident
Not embarrassed at all	No discomfort/pain	Very confident
Not embarrassed at all	No discomfort/pain at all	Very confident
Not embarrassed at all	Some discomfort/pain	Confident
Not embarrassed at all	No discomfort/pain at all	Confident
Not embarrassed at all	No discomfort/pain	Confident
Not embarrassed at all	No discomfort/pain at all	Very confident
Not embarrassed at all	No discomfort/pain	Confident
Not embarrassed at all	Some discomfort/pain	Very confident
Not embarrassed at all	No discomfort/pain at all	Very confident
Not embarrassed at all	No discomfort/pain at all	Very confident
Not embarrassed at all	Some discomfort/pain	Very confident
Not embarrassed at all	No discomfort/pain at all	Very confident
Not embarrassed at all	Some discomfort/pain	Very confident
Not embarrassed at all	No discomfort/pain at all	Very confident
Not embarrassed at all	No discomfort/pain at all	Very confident
Not embarrassed at all	No discomfort/pain at all	Very confident
Not embarrassed at all	No discomfort/pain at all	Confident
Not embarrassed at all	No discomfort/pain at all	Confident
Not embarrassed at all	Neutral	Confident
Not embarrassed at all	Neutral	Confident
Not embarrassed at all	No discomfort/pain at all	Very confident
Not embarrassed at all	No discomfort/pain at all	Very confident
Not embarrassed at all	Some discomfort/pain	Very confident
Not embarrassed at all	No discomfort/pain at all	Very confident
Not embarrassed	Some discomfort/pain	Confident
Not embarrassed at all	No discomfort/pain at all	Very confident

Not embarrassed	Some discomfort/pain	Confident
Not embarrassed at all	Some discomfort/pain	Confident
Not embarrassed at all	Some discomfort/pain	Confident
Not embarrassed at all	No discomfort/pain at all	Very confident
Not embarrassed at all	Some discomfort/pain	Very confident
Not embarrassed at all	No discomfort/pain	Very confident
Not embarrassed at all	Some discomfort/pain	Not confident
Not embarrassed at all	Neutral	Confident
Not embarrassed at all	No discomfort/pain	Very confident
Not embarrassed at all	No discomfort/pain at all	Very confident
Not embarrassed	Some discomfort/pain	Neutral
Not embarrassed at all	No discomfort/pain	Very confident
Not embarrassed at all	Some discomfort/pain	Very confident
Not embarrassed at all	No discomfort/pain	Very confident
Not embarrassed at all	No discomfort/pain	Not confident
Not embarrassed at all	Some discomfort/pain	Confident
Not embarrassed at all	No discomfort/pain at all	Confident
Not embarrassed at all	No discomfort/pain at all	Very confident
Not embarrassed at all	No discomfort/pain at all	Very confident
Neutral	No discomfort/pain	Very confident
Not embarrassed at all	Neutral	Neutral
Not embarrassed at all	No discomfort/pain	Very confident
Not embarrassed at all	No discomfort/pain at all	Very confident
Not embarrassed at all	Some discomfort/pain	Confident
Not embarrassed at all	No discomfort/pain	Confident
Not embarrassed	No discomfort/pain	Confident
Not embarrassed at all	No discomfort/pain	Confident
Not embarrassed at all	No discomfort/pain	Confident
Not embarrassed at all	No discomfort/pain	Confident
Not embarrassed at all	No discomfort/pain at all	Very confident
Not embarrassed at all	Some discomfort/pain	Confident
Not embarrassed at all	No discomfort/pain at all	Very confident
Not embarrassed at all	Some discomfort/pain	Confident

Not embarrassed at all	No discomfort/pain	Very confident
Not embarrassed at all	No discomfort/pain	Neutral
Not embarrassed at all	Some discomfort/pain	Very confident
Not embarrassed at all	No discomfort/pain at all	Very confident
Not embarrassed at all	No discomfort/pain	Confident
Not embarrassed at all	No discomfort/pain at all	Very confident
Not embarrassed at all	Severe discomfort/pain	Confident
Not embarrassed at all	Some discomfort/pain	Very confident
Not embarrassed at all	No discomfort/pain	Confident
Not embarrassed at all	Some discomfort/pain	Confident
Not embarrassed at all	No discomfort/pain at all	Very confident
Not embarrassed at all	Some discomfort/pain	Not confident
Not embarrassed at all	No discomfort/pain at all	Very confident
Not embarrassed at all	Some discomfort/pain	Very confident
Not embarrassed at all	Some discomfort/pain	Confident
Not embarrassed at all	Some discomfort/pain	Confident
Not embarrassed at all	No discomfort/pain	Confident
Not embarrassed	No discomfort/pain	Confident
Not embarrassed	Some discomfort/pain	Neutral
Not embarrassed at all	No discomfort/pain	Confident
Not embarrassed at all	No discomfort/pain at all	Very confident
Not embarrassed at all	Neutral	Very confident
Not embarrassed at all	Neutral	Very confident
Not embarrassed at all	No discomfort/pain at all	Confident
Not embarrassed at all	No discomfort/pain at all	Very confident
Not embarrassed at all	No discomfort/pain	Confident
Not embarrassed	Neutral	Confident
Not embarrassed	No discomfort/pain at all	Confident
Not embarrassed at all	No discomfort/pain	Confident
Not embarrassed at all	Neutral	Confident
Not embarrassed	No discomfort/pain	Confident
Not embarrassed at all	No discomfort/pain at all	Very confident
Not embarrassed	Neutral	Neutral

Not embarrassed	No discomfort/pain	Confident
Not embarrassed	No discomfort/pain	Confident
Not embarrassed	Some discomfort/pain	Confident
Not embarrassed	Some discomfort/pain	Confident
Not embarrassed	Some discomfort/pain	Confident
Not embarrassed	No discomfort/pain	Confident
Not embarrassed	No discomfort/pain	Confident
Not embarrassed	No discomfort/pain at all	Very confident
Not embarrassed at all	No discomfort/pain	Very confident
Not embarrassed	Severe discomfort/pain	Confident
Not embarrassed	No discomfort/pain at all	Confident
Not embarrassed	No discomfort/pain at all	Confident
Not embarrassed	No discomfort/pain at all	Very confident
Not embarrassed	No discomfort/pain at all	Very confident
Not embarrassed	Neutral	Confident
Not embarrassed at all	Some discomfort/pain	Very confident
Not embarrassed at all	No discomfort/pain	Very confident
Not embarrassed	Some discomfort/pain	Confident
Not embarrassed	Some discomfort/pain	Confident
Not embarrassed	Neutral	Confident
Not embarrassed at all	No discomfort/pain	Very confident
Not embarrassed at all	No discomfort/pain at all	Very confident
Not embarrassed at all	No discomfort/pain at all	Confident
Not embarrassed at all	No discomfort/pain at all	Very confident
Not embarrassed at all	No discomfort/pain	Confident
Not embarrassed	Some discomfort/pain	Not confident
Not embarrassed at all	No discomfort/pain	Confident
Not embarrassed at all	No discomfort/pain	Confident
Neutral	Neutral	Confident
Not embarrassed at all	Some discomfort/pain	Confident
Not embarrassed	Some discomfort/pain	Confident
Not embarrassed at all	Some discomfort/pain	Confident
Not embarrassed at all	No discomfort/pain at all	Very confident

Not embarrassed at all	Neutral	Confident
Not embarrassed	No discomfort/pain	Neutral
Not embarrassed	No discomfort/pain at all	Confident
Not embarrassed at all	Neutral	Confident
Not embarrassed at all	No discomfort/pain at all	Very confident
Not embarrassed at all	Some discomfort/pain	Neutral
Not embarrassed at all	No discomfort/pain at all	Very confident
Not embarrassed at all	Some discomfort/pain	Very confident
Not embarrassed at all	No discomfort/pain	Confident
Not embarrassed at all	No discomfort/pain	Confident
Embarrassed	Some discomfort/pain	Confident
Not embarrassed	Some discomfort/pain	Not confident
Not embarrassed at all	No discomfort/pain	Very confident
Not embarrassed at all	Some discomfort/pain	Confident
Not embarrassed at all	Neutral	Confident
Not embarrassed at all	Neutral	Confident
Not embarrassed at all	No discomfort/pain at all	Very confident
Not embarrassed at all	No discomfort/pain	Neutral
Not embarrassed at all	Some discomfort/pain	Neutral
Not embarrassed	Neutral	Neutral
Not embarrassed at all	Some discomfort/pain	Not confident
Not embarrassed at all	No discomfort/pain	Confident
Not embarrassed at all	Some discomfort/pain	Neutral
Not embarrassed at all	No discomfort/pain at all	Confident
Not embarrassed at all	No discomfort/pain at all	Very confident
Not embarrassed at all	Some discomfort/pain	Confident
Not embarrassed at all	No discomfort/pain at all	Neutral
Not embarrassed	Some discomfort/pain	Confident
Not embarrassed	No discomfort/pain	Neutral
Neutral	Some discomfort/pain	Not confident at all
Not embarrassed at all	Some discomfort/pain	Confident
Not embarrassed at all	No discomfort/pain at all	Very confident
Not embarrassed	No discomfort/pain	Confident

Not embarrassed at all	No discomfort/pain at all	Very confident
Not embarrassed at all	Some discomfort/pain	Very confident
Not embarrassed at all	No discomfort/pain at all	Very confident
Not embarrassed at all	No discomfort/pain at all	Confident
Not embarrassed at all	Neutral	Neutral
Not embarrassed at all	No discomfort/pain at all	Confident
Not embarrassed	Some discomfort/pain	Neutral
Not embarrassed at all	No discomfort/pain at all	Confident
Not embarrassed at all	No discomfort/pain	Confident
Not embarrassed	Some discomfort/pain	Confident
Not embarrassed at all	Some discomfort/pain	Confident
Not embarrassed at all	No discomfort/pain	Neutral
Not embarrassed at all	Some discomfort/pain	Very confident
Not embarrassed at all	No discomfort/pain at all	Confident
Not embarrassed at all	No discomfort/pain	Confident
Not embarrassed at all	Neutral	Very confident
Not embarrassed at all	Some discomfort/pain	Neutral
Not embarrassed at all	Some discomfort/pain	Very confident
Embarrassed	Some discomfort/pain	Confident
Not embarrassed at all	Some discomfort/pain	Very confident
Not embarrassed at all	No discomfort/pain	Confident
Not embarrassed at all	No discomfort/pain at all	Neutral
Not embarrassed at all	Severe discomfort/pain	Neutral
Not embarrassed at all	No discomfort/pain at all	Very confident
Not embarrassed at all	No discomfort/pain	Very confident
Not embarrassed at all	Some discomfort/pain	Not confident
Not embarrassed at all	No discomfort/pain at all	Very confident
Not embarrassed at all	No discomfort/pain at all	Very confident
Not embarrassed at all	No discomfort/pain	Confident

Not embarrassed	No discomfort/pain	Confident
Not embarrassed at all	No discomfort/pain	Very confident
Not embarrassed at all	No discomfort/pain at all	Very confident
Not embarrassed at all	Some discomfort/pain	Very confident
Not embarrassed at all	Neutral	Confident
Not embarrassed at all	Neutral	Confident
Not embarrassed	No discomfort/pain	Very confident
Not embarrassed	Some discomfort/pain	Not confident
Neutral	Some discomfort/pain	Neutral
Not embarrassed at all	No discomfort/pain at all	Confident
Not embarrassed	No discomfort/pain	Neutral
Not embarrassed at all	Some discomfort/pain	Confident
Not embarrassed at all	No discomfort/pain	Confident
Not embarrassed at all	No discomfort/pain at all	Neutral
Not embarrassed at all	No discomfort/pain	Very confident
Not embarrassed at all	No discomfort/pain at all	Very confident
Not embarrassed at all	No discomfort/pain at all	Very confident
Neutral	No discomfort/pain at all	Very confident
Neutral	Some discomfort/pain	Neutral
Not embarrassed at all	No discomfort/pain at all	Very confident
Not embarrassed at all	No discomfort/pain at all	Not confident
Not embarrassed at all	No discomfort/pain	Very confident
Not embarrassed at all	No discomfort/pain	Confident
Not embarrassed at all	No discomfort/pain at all	Very confident
Not embarrassed at all	Some discomfort/pain	Neutral
Not embarrassed at all	Some discomfort/pain	Confident
Not embarrassed at all	No discomfort/pain at all	Very confident
Not embarrassed at all	No discomfort/pain at all	Very confident
Not embarrassed at all	No discomfort/pain at all	Very confident
Not embarrassed at all	No discomfort/pain at all	Very confident
Not embarrassed	No discomfort/pain at all	Very confident
Not embarrassed at all	Some discomfort/pain	Confident
Not embarrassed at all	No discomfort/pain	Confident

Not embarrassed at all	No discomfort/pain	Confident
Not embarrassed at all	Some discomfort/pain	Very confident
Not embarrassed at all	Some discomfort/pain	Confident
Not embarrassed at all	No discomfort/pain at all	Very confident
Not embarrassed at all	Neutral	Very confident
Not embarrassed at all	No discomfort/pain at all	Confident
Not embarrassed	Neutral	Confident
Not embarrassed	Some discomfort/pain	Confident
Not embarrassed at all	No discomfort/pain	Confident
Not embarrassed at all	Some discomfort/pain	Confident
Not embarrassed at all	Some discomfort/pain	Neutral
Not embarrassed at all	No discomfort/pain	Confident
Not embarrassed at all	No discomfort/pain at all	Very confident
Not embarrassed at all	No discomfort/pain at all	Confident
Not embarrassed at all	No discomfort/pain	Confident
Neutral	No discomfort/pain	Confident
Not embarrassed	Some discomfort/pain	Not confident
Not embarrassed at all	No discomfort/pain	Confident
Not embarrassed at all	Some discomfort/pain	Confident
Neutral	No discomfort/pain	Confident
Not embarrassed	Some discomfort/pain	Confident
Not embarrassed	Some discomfort/pain	Neutral
Not embarrassed at all	No discomfort/pain	Very confident
Not embarrassed at all	Neutral	Not confident
Not embarrassed at all	Some discomfort/pain	Confident
Not embarrassed	Some discomfort/pain	Confident
Not embarrassed	No discomfort/pain	Neutral
Not embarrassed at all	No discomfort/pain	Very confident
Not embarrassed at all	No discomfort/pain at all	Very confident
Not embarrassed at all	Some discomfort/pain	Confident
Not embarrassed at all	Some discomfort/pain	Very confident
Not embarrassed at all	No discomfort/pain	Confident
Not embarrassed at all	No discomfort/pain at all	Very confident

Not embarrassed at all	No discomfort/pain at all	Very confident
Not embarrassed at all	No discomfort/pain at all	Confident
Not embarrassed at all	No discomfort/pain	Confident
Not embarrassed at all	No discomfort/pain at all	Confident
Not embarrassed at all	No discomfort/pain	Confident
Not embarrassed at all	Some discomfort/pain	Confident
Not embarrassed at all	Some discomfort/pain	Confident
Not embarrassed at all	No discomfort/pain	Confident
Not embarrassed at all	No discomfort/pain	Confident
Not embarrassed at all	No discomfort/pain at all	Neutral
Neutral	Neutral	Confident
Not embarrassed at all	No discomfort/pain at all	Confident
Not embarrassed at all	No discomfort/pain at all	Very confident
Not embarrassed at all	No discomfort/pain	Confident
Not embarrassed at all	No discomfort/pain at all	Very confident
Not embarrassed	No discomfort/pain at all	Confident
Not embarrassed at all	Some discomfort/pain	Confident
Not embarrassed at all	No discomfort/pain at all	Confident
Not embarrassed at all	Some discomfort/pain	Neutral
Not embarrassed at all	No discomfort/pain	Confident
Not embarrassed at all	No discomfort/pain at all	Not confident
Not embarrassed at all	Some discomfort/pain	Confident
Not embarrassed at all	Some discomfort/pain	Confident
Not embarrassed at all	No discomfort/pain at all	Very confident
Not embarrassed at all	No discomfort/pain at all	Confident
Not embarrassed at all	Some discomfort/pain	Neutral
Not embarrassed at all	Neutral	Confident
Not embarrassed at all	No discomfort/pain at all	Very confident
Not embarrassed at all	Some discomfort/pain	Confident
Not embarrassed at all	No discomfort/pain at all	Confident
Not embarrassed at all	No discomfort/pain at all	Confident
Not embarrassed at all	Some discomfort/pain	Confident
Not embarrassed at all	No discomfort/pain at all	Confident

Not embarrassed at all	No discomfort/pain	Confident
Not embarrassed at all	Some discomfort/pain	Confident
Not embarrassed at all	No discomfort/pain	Confident
Not embarrassed at all	Neutral	Confident
Not embarrassed at all	No discomfort/pain at all	Very confident
Not embarrassed at all	No discomfort/pain at all	Very confident
Not embarrassed at all	Some discomfort/pain	Not confident
Not embarrassed at all	No discomfort/pain at all	Confident
Not embarrassed at all	No discomfort/pain at all	Very confident
Not embarrassed at all	Some discomfort/pain	Confident
Not embarrassed	Some discomfort/pain	Neutral
Not embarrassed at all	No discomfort/pain at all	Very confident
Not embarrassed at all	No discomfort/pain at all	Very confident
Not embarrassed at all	No discomfort/pain at all	Very confident
Not embarrassed at all	No discomfort/pain at all	Very confident
Not embarrassed at all	No discomfort/pain at all	Very confident
Not embarrassed at all	No discomfort/pain	Very confident
Not embarrassed at all	No discomfort/pain at all	Confident
Not embarrassed at all	No discomfort/pain at all	Very confident
Not embarrassed at all	No discomfort/pain	Confident
Not embarrassed at all	Some discomfort/pain	Neutral
Not embarrassed at all	Some discomfort/pain	Confident
Not embarrassed at all	No discomfort/pain at all	Confident
Not embarrassed at all	Some discomfort/pain	Not confident
Not embarrassed at all	Some discomfort/pain	Confident
Not embarrassed at all	No discomfort/pain at all	Neutral
Not embarrassed at all	Some discomfort/pain	Very confident
Not embarrassed at all	No discomfort/pain at all	Confident
Neutral	Some discomfort/pain	Confident
Not embarrassed at all	No discomfort/pain at all	Confident
Not embarrassed at all	No discomfort/pain at all	Very confident
Not embarrassed at all	No discomfort/pain	Confident
Not embarrassed at all	No discomfort/pain at all	Confident

Not embarrassed at all	No discomfort/pain at all	Confident
Not embarrassed at all	Some discomfort/pain	Confident
Not embarrassed at all	No discomfort/pain at all	Confident
Not embarrassed at all	Some discomfort/pain	Confident
Not embarrassed at all	No discomfort/pain at all	Confident
Not embarrassed at all	No discomfort/pain at all	Confident
Not embarrassed at all	Some discomfort/pain	Confident
Not embarrassed at all	No discomfort/pain at all	Very confident
Not embarrassed at all	No discomfort/pain at all	Confident
Not embarrassed at all	Neutral	Confident
Not embarrassed at all	No discomfort/pain at all	Very confident
Not embarrassed at all	Some discomfort/pain	Neutral
Not embarrassed at all	No discomfort/pain at all	Confident
Not embarrassed at all	No discomfort/pain	Not confident
Not embarrassed at all	No discomfort/pain at all	Confident
Not embarrassed at all	No discomfort/pain at all	Very confident
Not embarrassed at all	Neutral	Confident
Not embarrassed at all	No discomfort/pain	Confident
Not embarrassed at all	No discomfort/pain	Confident
Not embarrassed at all	No discomfort/pain at all	Very confident
Not embarrassed at all	Neutral	Confident
Not embarrassed	Some discomfort/pain	Confident
Not embarrassed at all	No discomfort/pain at all	Very confident
Not embarrassed at all	No discomfort/pain at all	Very confident
Not embarrassed at all	Some discomfort/pain	Confident
Neutral	Some discomfort/pain	Confident
Not embarrassed at all	Neutral	Confident
Not embarrassed at all	No discomfort/pain at all	Confident
Not embarrassed at all	No discomfort/pain	Confident
Not embarrassed at all	No discomfort/pain	Very confident
Not embarrassed at all	Some discomfort/pain	Neutral
Not embarrassed at all	No discomfort/pain at all	Confident
Not embarrassed at all	Some discomfort/pain	Confident

Not embarrassed	Neutral	Confident
Not embarrassed at all	No discomfort/pain at all	Very confident
Not embarrassed at all	Some discomfort/pain	Confident
Not embarrassed at all	No discomfort/pain	Not confident
Not embarrassed at all	Some discomfort/pain	Confident
Not embarrassed at all	No discomfort/pain at all	Confident
Not embarrassed at all	No discomfort/pain at all	Confident
Not embarrassed at all	Neutral	Confident
Not embarrassed at all	No discomfort/pain at all	Confident
Not embarrassed at all	No discomfort/pain at all	Confident
Not embarrassed at all	Neutral	Confident
Not embarrassed at all	No discomfort/pain at all	Confident
Not embarrassed at all	No discomfort/pain	Not confident
Not embarrassed at all	No discomfort/pain at all	Confident
Not embarrassed at all	No discomfort/pain	Confident
Not embarrassed at all	Some discomfort/pain	Neutral
Not embarrassed at all	Some discomfort/pain	Confident
Not embarrassed at all	No discomfort/pain at all	Confident
Not embarrassed at all	No discomfort/pain at all	Very confident
Not embarrassed at all	Some discomfort/pain	Not confident
Not embarrassed at all	No discomfort/pain at all	Confident
Not embarrassed at all	No discomfort/pain at all	Confident
Not embarrassed at all	Neutral	Very confident
Not embarrassed at all	Some discomfort/pain	Confident
Not embarrassed at all	No discomfort/pain at all	Confident
Not embarrassed at all	No discomfort/pain	Confident
Not embarrassed at all	No discomfort/pain at all	Confident
Not embarrassed at all	No discomfort/pain at all	Very confident
Not embarrassed at all	No discomfort/pain at all	Very confident
Not embarrassed at all	No discomfort/pain at all	Very confident
Not embarrassed at all	Neutral	Confident
Not embarrassed at all	No discomfort/pain	Confident
Not embarrassed at all	Some discomfort/pain	Confident

Not embarrassed at all	Neutral	Confident
Not embarrassed at all	No discomfort/pain	Neutral
Not embarrassed at all	No discomfort/pain at all	Confident
Not embarrassed at all	No discomfort/pain at all	Confident
Not embarrassed at all	Neutral	Confident
Not embarrassed at all	No discomfort/pain at all	Very confident
Not embarrassed at all	No discomfort/pain at all	Very confident
Not embarrassed at all	No discomfort/pain at all	Very confident
Not embarrassed at all	No discomfort/pain	Confident
Not embarrassed at all	No discomfort/pain at all	Confident
Not embarrassed at all	No discomfort/pain	Confident
Not embarrassed at all	No discomfort/pain at all	Confident
Not embarrassed at all	Some discomfort/pain	Confident
Not embarrassed at all	Some discomfort/pain	Not confident
Not embarrassed at all	Neutral	Neutral
Not embarrassed at all	Neutral	Confident
Neutral	Neutral	Neutral
Not embarrassed at all	Some discomfort/pain	Confident
Not embarrassed at all	Some discomfort/pain	Neutral
Not embarrassed at all	Some discomfort/pain	Confident
Not embarrassed at all	No discomfort/pain at all	Very confident
Not embarrassed at all	No discomfort/pain at all	Confident
Not embarrassed at all	No discomfort/pain at all	Very confident
Not embarrassed at all	Neutral	Very confident
Not embarrassed at all	No discomfort/pain at all	Very confident
Not embarrassed at all	No discomfort/pain at all	Very confident
Not embarrassed at all	Some discomfort/pain	Confident
Not embarrassed at all	No discomfort/pain at all	Neutral
Not embarrassed at all	No discomfort/pain at all	Neutral
Not embarrassed at all	Neutral	Confident
Not embarrassed at all	Some discomfort/pain	Confident
Not embarrassed at all	Some discomfort/pain	Confident
Not embarrassed at all	Neutral	Confident

Not embarrassed at all	Neutral	Very confident
Embarrassed	Some discomfort/pain	Not confident
Not embarrassed at all	No discomfort/pain at all	Confident
Not embarrassed at all	No discomfort/pain at all	Very confident
Not embarrassed at all	Some discomfort/pain	Confident
Not embarrassed at all	No discomfort/pain	Confident
Not embarrassed at all	Some discomfort/pain	Neutral
Not embarrassed at all	No discomfort/pain at all	Confident
Not embarrassed at all	Neutral	Confident
Not embarrassed at all	No discomfort/pain at all	Confident
Not embarrassed at all	No discomfort/pain	Confident
Not embarrassed at all	Some discomfort/pain	Very confident
Not embarrassed at all	No discomfort/pain at all	Very confident
Not embarrassed at all	Some discomfort/pain	Confident
Not embarrassed at all	Some discomfort/pain	Confident
Not embarrassed at all	No discomfort/pain at all	Very confident
Not embarrassed at all	Neutral	Confident
Not embarrassed at all	Some discomfort/pain	Very confident
Not embarrassed at all	Some discomfort/pain	Not confident
Not embarrassed at all	No discomfort/pain at all	Very confident
Not embarrassed at all	Some discomfort/pain	Very confident
Not embarrassed at all	No discomfort/pain at all	Very confident
Not embarrassed at all	Neutral	Not confident
Not embarrassed at all	Neutral	Confident
Not embarrassed at all	No discomfort/pain at all	Very confident
Not embarrassed at all	Some discomfort/pain	Confident
Not embarrassed at all	Some discomfort/pain	Confident
Not embarrassed at all	Some discomfort/pain	Confident
Not embarrassed at all	No discomfort/pain at all	Confident
Not embarrassed at all	Neutral	Very confident
Not embarrassed at all	No discomfort/pain at all	Confident

Not embarrassed at all	No discomfort/pain	Neutral
Not embarrassed at all	No discomfort/pain at all	Confident
Not embarrassed at all	No discomfort/pain at all	Very confident
Not embarrassed at all	No discomfort/pain at all	Very confident
Not embarrassed at all	No discomfort/pain at all	Confident
Not embarrassed at all	Neutral	Neutral
Neutral	Neutral	Confident
Not embarrassed at all	Some discomfort/pain	Neutral
Not embarrassed at all	No discomfort/pain at all	Confident
Not embarrassed at all	No discomfort/pain	Confident
Not embarrassed at all	Neutral	Neutral
Not embarrassed at all	No discomfort/pain	Confident
Not embarrassed at all	No discomfort/pain at all	Confident
Not embarrassed at all	No discomfort/pain at all	Very confident
Not embarrassed at all	Some discomfort/pain	Confident
Not embarrassed at all	No discomfort/pain at all	Confident
Not embarrassed at all	No discomfort/pain	Confident
Not embarrassed at all	Neutral	Neutral
Not embarrassed at all	No discomfort/pain at all	Confident
Not embarrassed at all	No discomfort/pain at all	Confident
Not embarrassed at all	No discomfort/pain at all	Confident
Not embarrassed at all	Neutral	Confident
Not embarrassed at all	Neutral	Confident
Not embarrassed at all	No discomfort/pain	Confident
Not embarrassed at all	No discomfort/pain at all	Very confident
Not embarrassed at all	No discomfort/pain at all	Confident
Not embarrassed at all	No discomfort/pain	Confident
Not embarrassed	Some discomfort/pain	Not confident
Not embarrassed at all	Some discomfort/pain	Neutral
Not embarrassed at all	No discomfort/pain	Confident
Not embarrassed	Some discomfort/pain	Confident
Not embarrassed at all	No discomfort/pain	Very confident
Neutral	Neutral	Confident

Not embarrassed at all	No discomfort/pain at all	Confident
Not embarrassed at all	Some discomfort/pain	Very confident
Not embarrassed at all	Some discomfort/pain	Confident
Not embarrassed at all	Some discomfort/pain	Confident
Not embarrassed	No discomfort/pain	Confident
Not embarrassed at all	No discomfort/pain	Confident
Not embarrassed at all	Some discomfort/pain	Neutral
Not embarrassed at all	No discomfort/pain at all	Confident
Not embarrassed at all	No discomfort/pain	Confident
Not embarrassed	No discomfort/pain	Confident
Not embarrassed at all	No discomfort/pain at all	Very confident
Not embarrassed at all	No discomfort/pain at all	Very confident
Not embarrassed at all	No discomfort/pain at all	Confident
Not embarrassed at all	No discomfort/pain at all	Very confident
Not embarrassed at all	Some discomfort/pain	Very confident
Not embarrassed	Neutral	Confident
Not embarrassed at all	No discomfort/pain at all	Confident
Not embarrassed at all	Some discomfort/pain	Confident
Not embarrassed	Some discomfort/pain	Confident
Not embarrassed at all	No discomfort/pain at all	Confident
Not embarrassed at all	No discomfort/pain	Very confident
Not embarrassed at all	No discomfort/pain	Confident
Not embarrassed at all	Some discomfort/pain	Confident
Not embarrassed at all	No discomfort/pain at all	Very confident
Not embarrassed at all	No discomfort/pain at all	Very confident
Not embarrassed	Some discomfort/pain	Confident
Not embarrassed at all	Some discomfort/pain	Confident
Not embarrassed at all	No discomfort/pain at all	Confident
Not embarrassed at all	Some discomfort/pain	Very confident
Not embarrassed at all	Neutral	Confident
Not embarrassed at all	No discomfort/pain	Confident
Not embarrassed at all	No discomfort/pain at all	Very confident
Not embarrassed at all	No discomfort/pain at all	Confident

Not embarrassed at all	Neutral	Confident
Not embarrassed at all	No discomfort/pain at all	Neutral
Not embarrassed at all	No discomfort/pain at all	Confident
Not embarrassed at all	No discomfort/pain at all	Very confident
Not embarrassed at all	No discomfort/pain	Confident
Not embarrassed at all	Some discomfort/pain	Neutral
Not embarrassed at all	No discomfort/pain at all	Confident
Not embarrassed at all	Some discomfort/pain	Confident
Neutral	Neutral	Confident
Not embarrassed at all	No discomfort/pain at all	Confident
Not embarrassed at all	Some discomfort/pain	Confident
Not embarrassed at all	No discomfort/pain at all	Confident
Not embarrassed at all	No discomfort/pain at all	Very confident
Not embarrassed	Some discomfort/pain	Neutral
Not embarrassed at all	No discomfort/pain at all	Confident
Neutral	Neutral	Confident
Not embarrassed at all	Some discomfort/pain	Neutral
Embarrassed	No discomfort/pain at all	Confident
Not embarrassed at all	Some discomfort/pain	Very confident
Not embarrassed	Some discomfort/pain	Confident
Not embarrassed at all	Neutral	Confident
Not embarrassed	No discomfort/pain at all	Confident
Not embarrassed at all	No discomfort/pain	Confident
Not embarrassed at all	No discomfort/pain	Confident
Not embarrassed at all	No discomfort/pain at all	Very confident
Not embarrassed at all	No discomfort/pain at all	Confident
Not embarrassed at all	No discomfort/pain at all	Confident
Not embarrassed at all	No discomfort/pain	Confident
Neutral	Some discomfort/pain	Confident
Not embarrassed at all	No discomfort/pain at all	Very confident
Not embarrassed at all	No discomfort/pain at all	Very confident
Not embarrassed	Neutral	Confident
Not embarrassed at all	No discomfort/pain at all	Very confident

Not embarrassed at all	Some discomfort/pain	Confident
Not embarrassed at all	No discomfort/pain at all	Confident
Not embarrassed at all	Some discomfort/pain	Confident
Not embarrassed at all	No discomfort/pain at all	Very confident
Not embarrassed at all	No discomfort/pain at all	Very confident
Not embarrassed at all	No discomfort/pain	Not confident
Not embarrassed at all	Some discomfort/pain	Not confident
Not embarrassed	No discomfort/pain at all	Very confident
Not embarrassed at all	No discomfort/pain at all	Confident
Not embarrassed at all	No discomfort/pain	Confident
Not embarrassed	Some discomfort/pain	Confident
Not embarrassed at all	No discomfort/pain at all	Very confident
Not embarrassed at all	No discomfort/pain at all	Confident
Not embarrassed at all	No discomfort/pain at all	Confident
Not embarrassed at all	Some discomfort/pain	Confident
Not embarrassed at all	Some discomfort/pain	Confident
Not embarrassed at all	No discomfort/pain at all	Neutral
Not embarrassed at all	No discomfort/pain	Confident
Not embarrassed at all	Some discomfort/pain	Confident
Not embarrassed at all	No discomfort/pain at all	Confident
Not embarrassed	No discomfort/pain	Confident
Not embarrassed	Some discomfort/pain	Not confident
Not embarrassed	No discomfort/pain	Confident
Not embarrassed at all	No discomfort/pain at all	Very confident
Not embarrassed at all	No discomfort/pain	Very confident
Not embarrassed at all	No discomfort/pain at all	Confident
Not embarrassed	No discomfort/pain	Confident
Not embarrassed at all	Some discomfort/pain	Not confident
Not embarrassed at all	No discomfort/pain at all	Very confident
Not embarrassed	No discomfort/pain at all	Confident
Not embarrassed	No discomfort/pain	Confident
Not embarrassed	Some discomfort/pain	Neutral
Not embarrassed at all	No discomfort/pain at all	Confident

Not embarrassed at all	Neutral	Confident
Not embarrassed at all	No discomfort/pain at all	Confident
Not embarrassed at all	No discomfort/pain	Confident
Not embarrassed	Some discomfort/pain	Neutral
Not embarrassed at all	No discomfort/pain	Very confident
Not embarrassed at all	Some discomfort/pain	Confident
Not embarrassed	No discomfort/pain	Confident
Not embarrassed at all	No discomfort/pain at all	Confident
Not embarrassed at all	No discomfort/pain	Confident
Not embarrassed at all	Neutral	Confident
Not embarrassed	No discomfort/pain	Confident
Not embarrassed at all	Neutral	Not confident
Not embarrassed at all	No discomfort/pain at all	Very confident
Not embarrassed at all	No discomfort/pain at all	Confident
Not embarrassed	No discomfort/pain	Confident
Not embarrassed at all	Neutral	Confident
Not embarrassed at all	No discomfort/pain at all	Very confident
Not embarrassed	No discomfort/pain at all	Confident
Not embarrassed at all	No discomfort/pain at all	Very confident
Not embarrassed at all	No discomfort/pain at all	Very confident
Not embarrassed at all	No discomfort/pain	Confident
Not embarrassed at all	No discomfort/pain at all	Very confident
Not embarrassed	Some discomfort/pain	Confident
Not embarrassed	No discomfort/pain	Confident
Not embarrassed	No discomfort/pain	Confident
Not embarrassed at all	Some discomfort/pain	Not confident
Not embarrassed at all	No discomfort/pain at all	Confident
Not embarrassed at all	No discomfort/pain at all	Confident
Not embarrassed at all	No discomfort/pain	Confident
Not embarrassed at all	No discomfort/pain at all	Confident
Not embarrassed	No discomfort/pain at all	Confident
Not embarrassed at all	No discomfort/pain at all	Very confident
Not embarrassed at all	No discomfort/pain at all	Confident

Not embarrassed at all	No discomfort/pain at all	Neutral
Not embarrassed at all	No discomfort/pain at all	Confident
Not embarrassed at all	No discomfort/pain	Very confident
Not embarrassed at all	Neutral	Neutral
Not embarrassed at all	No discomfort/pain at all	Confident
Not embarrassed at all	No discomfort/pain	Confident
Not embarrassed at all	No discomfort/pain	Very confident
Not embarrassed at all	Some discomfort/pain	Confident
Not embarrassed at all	No discomfort/pain at all	Confident
Not embarrassed at all	No discomfort/pain	Confident
Not embarrassed at all	No discomfort/pain at all	Confident
Not embarrassed at all	No discomfort/pain at all	Confident
Not embarrassed at all	No discomfort/pain at all	Very confident
Not embarrassed at all	No discomfort/pain at all	Very confident
Not embarrassed at all	No discomfort/pain at all	Confident
Not embarrassed at all	No discomfort/pain	Neutral
Not embarrassed at all	No discomfort/pain at all	Very confident
Not embarrassed at all	No discomfort/pain at all	Confident
Not embarrassed at all	No discomfort/pain at all	Confident
Not embarrassed at all	No discomfort/pain at all	Confident
Not embarrassed at all	Some discomfort/pain	Neutral
Not embarrassed at all	Some discomfort/pain	Confident
Not embarrassed at all	Neutral	Confident
Not embarrassed at all	Neutral	Neutral
Not embarrassed at all	Some discomfort/pain	Confident
Not embarrassed at all	Some discomfort/pain	Confident
Not embarrassed	Some discomfort/pain	Confident
Not embarrassed at all	No discomfort/pain at all	Very confident
Not embarrassed at all	No discomfort/pain	Confident
Not embarrassed at all	Some discomfort/pain	Confident
Not embarrassed at all	No discomfort/pain	Neutral
Not embarrassed at all	No discomfort/pain at all	Very confident
Not embarrassed at all	Some discomfort/pain	Confident

Neutral	Some discomfort/pain	Neutral
Not embarrassed at all	No discomfort/pain at all	Very confident
Not embarrassed at all	No discomfort/pain	Confident
Not embarrassed	No discomfort/pain	Confident
Not embarrassed at all	Some discomfort/pain	Neutral
Not embarrassed at all	No discomfort/pain at all	Very confident
Not embarrassed at all	No discomfort/pain at all	Very confident
Not embarrassed at all	No discomfort/pain	Confident
Not embarrassed at all	No discomfort/pain at all	Very confident
Not embarrassed at all	No discomfort/pain	Very confident
Not embarrassed at all	No discomfort/pain at all	Very confident
Not embarrassed at all	No discomfort/pain	Confident
Not embarrassed at all	No discomfort/pain at all	Confident
Not embarrassed at all	No discomfort/pain at all	Very confident
Not embarrassed at all	Some discomfort/pain	Not confident
Not embarrassed at all	Some discomfort/pain	Confident
Not embarrassed at all	No discomfort/pain at all	Very confident
Not embarrassed at all	Some discomfort/pain	Confident
Not embarrassed at all	No discomfort/pain at all	Confident
Not embarrassed at all	No discomfort/pain at all	Confident
Not embarrassed at all	No discomfort/pain	Neutral
Not embarrassed at all	Neutral	Confident
Not embarrassed at all	No discomfort/pain at all	Very confident
Not embarrassed at all	No discomfort/pain at all	Confident
Not embarrassed at all	No discomfort/pain at all	Confident
Not embarrassed	No discomfort/pain	Confident
Not embarrassed at all	Some discomfort/pain	Confident
Not embarrassed at all	Some discomfort/pain	Confident
Not embarrassed at all	No discomfort/pain at all	Very confident
Not embarrassed	No discomfort/pain at all	Confident
Not embarrassed at all	Neutral	Confident
Not embarrassed	Some discomfort/pain	Not confident
Not embarrassed at all	No discomfort/pain at all	Very confident

Not embarrassed at all	No discomfort/pain at all	Very confident
Not embarrassed at all	No discomfort/pain	Confident
Not embarrassed at all	Neutral	Very confident
Not embarrassed	Some discomfort/pain	Neutral
Not embarrassed at all	Some discomfort/pain	Confident
Not embarrassed at all	No discomfort/pain at all	Very confident
Not embarrassed	Some discomfort/pain	Confident
Not embarrassed at all	Some discomfort/pain	Not confident
Not embarrassed	Some discomfort/pain	Not confident
Not embarrassed at all	No discomfort/pain	Confident
Not embarrassed	No discomfort/pain	Confident
Not embarrassed at all	No discomfort/pain at all	Confident
Not embarrassed at all	Some discomfort/pain	Confident
Not embarrassed at all	No discomfort/pain at all	Confident
Not embarrassed at all	No discomfort/pain at all	Confident
Not embarrassed at all	No discomfort/pain at all	Very confident
Not embarrassed at all	Some discomfort/pain	Confident
Not embarrassed at all	No discomfort/pain at all	Very confident
Not embarrassed at all	No discomfort/pain at all	Confident
Not embarrassed at all	No discomfort/pain at all	Very confident
Not embarrassed at all	No discomfort/pain at all	Very confident
Not embarrassed at all	No discomfort/pain	Very confident
Not embarrassed at all	No discomfort/pain at all	Very confident
Not embarrassed at all	No discomfort/pain at all	Confident
Not embarrassed at all	No discomfort/pain at all	Confident
Not embarrassed at all	No discomfort/pain at all	Very confident
Not embarrassed	No discomfort/pain at all	Very confident
Not embarrassed at all	Some discomfort/pain	Confident
Not embarrassed at all	No discomfort/pain at all	Confident
Not embarrassed at all	Neutral	Confident
Not embarrassed at all	No discomfort/pain at all	Confident
Not embarrassed at all	No discomfort/pain at all	Very confident

Not embarrassed at all	Neutral	Neutral
Not embarrassed at all	No discomfort/pain at all	Very confident
Embarrassed	Some discomfort/pain	Neutral
Not embarrassed at all	No discomfort/pain	Confident
Not embarrassed	No discomfort/pain	Confident
Not embarrassed at all	Neutral	Confident
Not embarrassed at all	Neutral	Very confident
Not embarrassed at all	Some discomfort/pain	Confident
Not embarrassed at all	No discomfort/pain at all	Very confident
Not embarrassed at all	No discomfort/pain at all	Very confident
Not embarrassed at all	No discomfort/pain at all	Very confident
Not embarrassed at all	No discomfort/pain at all	Confident
Not embarrassed at all	No discomfort/pain at all	Very confident
Embarrassed	Neutral	Confident
Not embarrassed at all	Some discomfort/pain	Neutral
Not embarrassed at all	No discomfort/pain at all	Very confident
Not embarrassed at all	No discomfort/pain at all	Confident
Not embarrassed at all	No discomfort/pain at all	Confident
Not embarrassed at all	No discomfort/pain at all	Very confident
Not embarrassed at all	No discomfort/pain at all	Very confident
Not embarrassed at all	No discomfort/pain at all	Neutral
Not embarrassed at all	No discomfort/pain at all	Very confident
Not embarrassed at all	No discomfort/pain	Neutral
Not embarrassed at all	No discomfort/pain at all	Confident
Not embarrassed at all	No discomfort/pain at all	Very confident
Not embarrassed	No discomfort/pain at all	Confident
Not embarrassed	Some discomfort/pain	Confident
Not embarrassed at all	No discomfort/pain at all	Very confident
Not embarrassed at all	No discomfort/pain at all	Very confident
Not embarrassed	No discomfort/pain	Confident
Not embarrassed at all	No discomfort/pain at all	Very confident
Not embarrassed at all	Some discomfort/pain	Confident

Not embarrassed at all	Some discomfort/pain	Confident
Not embarrassed at all	No discomfort/pain	Confident
Not embarrassed at all	Neutral	Confident
Not embarrassed at all	No discomfort/pain at all	Confident
Not embarrassed at all	Some discomfort/pain	Confident
Not embarrassed	No discomfort/pain at all	Confident
Not embarrassed at all	No discomfort/pain	Neutral
Not embarrassed at all	No discomfort/pain at all	Very confident
Not embarrassed at all	No discomfort/pain at all	Very confident
Not embarrassed	Some discomfort/pain	Confident
Not embarrassed at all	No discomfort/pain at all	Very confident
Not embarrassed at all	No discomfort/pain at all	Very confident
Not embarrassed	Neutral	Confident
Not embarrassed at all	No discomfort/pain at all	Confident
Not embarrassed at all	No discomfort/pain at all	Very confident
Not embarrassed at all	No discomfort/pain	Confident
Not embarrassed at all	No discomfort/pain at all	Very confident
Not embarrassed at all	Some discomfort/pain	Confident
Not embarrassed at all	No discomfort/pain at all	Confident
Not embarrassed at all	No discomfort/pain	Neutral
Not embarrassed at all	No discomfort/pain at all	Very confident
Not embarrassed at all	No discomfort/pain at all	Very confident
Not embarrassed at all	No discomfort/pain at all	Very confident
Not embarrassed at all	Some discomfort/pain	Confident
Not embarrassed at all	Neutral	Neutral
Not embarrassed at all	No discomfort/pain at all	Very confident
Not embarrassed at all	No discomfort/pain at all	Very confident
Not embarrassed at all	No discomfort/pain at all	Confident
Not embarrassed at all	No discomfort/pain at all	Confident
Not embarrassed at all	No discomfort/pain at all	Confident
Not embarrassed at all	No discomfort/pain at all	Very confident
Not embarrassed at all	No discomfort/pain at all	Very confident
Not embarrassed at all	No discomfort/pain at all	Confident

Not embarrassed at all	Neutral	Confident
Not embarrassed at all	Neutral	Confident
Not embarrassed at all	Some discomfort/pain	Not confident
Embarrassed	Some discomfort/pain	Confident
Not embarrassed	Some discomfort/pain	Confident
Not embarrassed at all	No discomfort/pain at all	Very confident
Not embarrassed at all	Neutral	Confident
Not embarrassed at all	No discomfort/pain at all	Confident
Not embarrassed at all	No discomfort/pain at all	Very confident
Not embarrassed at all	No discomfort/pain at all	Confident
Not embarrassed at all	No discomfort/pain at all	Confident
Not embarrassed at all	Some discomfort/pain	Not confident
Not embarrassed at all	Some discomfort/pain	Not confident
Not embarrassed at all	Some discomfort/pain	Confident
Not embarrassed at all	No discomfort/pain at all	Very confident
Not embarrassed at all	No discomfort/pain at all	Very confident
Not embarrassed at all	No discomfort/pain at all	Confident
Not embarrassed	No discomfort/pain	Confident
Not embarrassed at all	No discomfort/pain	Very confident
Not embarrassed at all	No discomfort/pain at all	Very confident
Not embarrassed at all	No discomfort/pain at all	Very confident
Not embarrassed	No discomfort/pain	Confident
Not embarrassed at all	Some discomfort/pain	Confident
Not embarrassed at all	No discomfort/pain	Confident
Not embarrassed at all	No discomfort/pain	Confident
Not embarrassed at all	No discomfort/pain at all	Very confident
Not embarrassed at all	No discomfort/pain	Neutral
Not embarrassed at all	No discomfort/pain	Confident
Not embarrassed at all	No discomfort/pain	Confident
Not embarrassed at all	Some discomfort/pain	Not confident
Not embarrassed	No discomfort/pain	Confident
Not embarrassed at all	No discomfort/pain at all	Very confident
Not embarrassed at all	No discomfort/pain at all	Confident

Not embarrassed at all	No discomfort/pain at all	Confident
Not embarrassed	No discomfort/pain at all	Not confident
Not embarrassed at all	Some discomfort/pain	Confident
Not embarrassed at all	No discomfort/pain at all	Very confident
Not embarrassed at all	No discomfort/pain at all	Confident
Not embarrassed	Some discomfort/pain	Confident
Not embarrassed at all	No discomfort/pain at all	Very confident
Not embarrassed at all	No discomfort/pain at all	Very confident
Not embarrassed at all	Some discomfort/pain	Confident
Not embarrassed	No discomfort/pain at all	Confident
Not embarrassed	Some discomfort/pain	Neutral
Not embarrassed at all	No discomfort/pain	Confident
Not embarrassed at all	No discomfort/pain at all	Confident
Not embarrassed at all	Some discomfort/pain	Confident
Not embarrassed	No discomfort/pain	Confident
Neutral	Neutral	Confident
Not embarrassed	No discomfort/pain	Confident
Not embarrassed at all	No discomfort/pain at all	Confident
Not embarrassed at all	No discomfort/pain at all	Very confident
Not embarrassed at all	No discomfort/pain at all	Confident
Not embarrassed at all	No discomfort/pain at all	Confident
Not embarrassed	Neutral	Confident
Not embarrassed	Some discomfort/pain	Confident
Not embarrassed at all	No discomfort/pain	Confident
Not embarrassed at all	No discomfort/pain at all	Very confident
Not embarrassed	No discomfort/pain at all	Very confident
Not embarrassed at all	No discomfort/pain	Confident
Not embarrassed at all	No discomfort/pain at all	Very confident
Not embarrassed at all	No discomfort/pain at all	Very confident
Not embarrassed at all	No discomfort/pain at all	Confident
Not embarrassed at all	No discomfort/pain	Confident
Not embarrassed at all	Neutral	Confident
Not embarrassed at all	No discomfort/pain at all	Very confident

Not embarrassed at all	No discomfort/pain at all	Confident
Not embarrassed	Some discomfort/pain	Confident
Not embarrassed at all	Some discomfort/pain	Confident
Not embarrassed at all	No discomfort/pain at all	Very confident
Not embarrassed	No discomfort/pain	Confident
Not embarrassed at all	Some discomfort/pain	Confident
Not embarrassed at all	No discomfort/pain at all	Confident
Not embarrassed at all	Some discomfort/pain	Confident
Not embarrassed at all	No discomfort/pain	Confident
Not embarrassed at all	No discomfort/pain at all	Confident
Not embarrassed at all	Neutral	Confident
Not embarrassed	Some discomfort/pain	Confident
Not embarrassed at all	Neutral	Confident
Not embarrassed	Some discomfort/pain	Confident
Not embarrassed at all	No discomfort/pain	Confident
Not embarrassed at all	No discomfort/pain at all	Confident
Not embarrassed	Some discomfort/pain	Neutral
Not embarrassed at all	No discomfort/pain at all	Confident
Not embarrassed	No discomfort/pain	Confident
Not embarrassed at all	No discomfort/pain at all	Confident
Not embarrassed at all	No discomfort/pain at all	Very confident
Not embarrassed	Some discomfort/pain	Confident
Not embarrassed at all	No discomfort/pain	Very confident
Not embarrassed at all	Neutral	Confident
Not embarrassed	No discomfort/pain	Confident
Not embarrassed at all	No discomfort/pain at all	Confident
Not embarrassed at all	No discomfort/pain at all	Very confident
Not embarrassed at all	No discomfort/pain	Confident
Not embarrassed	No discomfort/pain	Confident
Not embarrassed at all	No discomfort/pain at all	Confident
Not embarrassed at all	No discomfort/pain at all	Very confident
Not embarrassed	No discomfort/pain	Confident
Not embarrassed at all	No discomfort/pain	Confident

Not embarrassed at all	No discomfort/pain at all	Very confident
Not embarrassed at all	No discomfort/pain	Confident
Not embarrassed at all	No discomfort/pain at all	Very confident
Not embarrassed at all	No discomfort/pain at all	Very confident
Not embarrassed at all	Some discomfort/pain	Confident
Not embarrassed	Some discomfort/pain	Confident
Not embarrassed at all	No discomfort/pain at all	Very confident
Not embarrassed	Some discomfort/pain	Confident
Not embarrassed at all	Some discomfort/pain	Confident
Not embarrassed at all	No discomfort/pain at all	Very confident
Not embarrassed	Some discomfort/pain	Confident
Not embarrassed	Some discomfort/pain	Confident
Not embarrassed	No discomfort/pain	Neutral
Not embarrassed at all	No discomfort/pain at all	Confident
Not embarrassed at all	No discomfort/pain at all	Very confident
Not embarrassed	No discomfort/pain at all	Confident
Not embarrassed at all	No discomfort/pain	Confident
Not embarrassed at all	No discomfort/pain	Confident
Not embarrassed at all	No discomfort/pain at all	Very confident
Not embarrassed at all	No discomfort/pain at all	Very confident
Not embarrassed at all	No discomfort/pain	Confident
Not embarrassed	Some discomfort/pain	Confident
Not embarrassed at all	No discomfort/pain	Confident
Not embarrassed at all	Some discomfort/pain	Confident
Not embarrassed at all	No discomfort/pain at all	Very confident
Not embarrassed at all	No discomfort/pain at all	Very confident
Not embarrassed at all	Some discomfort/pain	Very confident
Not embarrassed at all	Some discomfort/pain	Confident
Not embarrassed at all	Some discomfort/pain	Confident
Not embarrassed at all	No discomfort/pain	Confident
Not embarrassed	Some discomfort/pain	Confident
Not embarrassed at all	Some discomfort/pain	Confident
Not embarrassed at all	No discomfort/pain at all	Not confident

Not embarrassed	Some discomfort/pain	Not confident
Not embarrassed at all	No discomfort/pain at all	Confident
Not embarrassed	No discomfort/pain	Confident
Not embarrassed	No discomfort/pain	Not confident
Not embarrassed at all	No discomfort/pain	Very confident
Not embarrassed	No discomfort/pain	Confident
Not embarrassed at all	No discomfort/pain at all	Confident
Not embarrassed at all	Some discomfort/pain	Not confident
Not embarrassed at all	No discomfort/pain at all	Very confident
Not embarrassed at all	No discomfort/pain at all	Very confident
Not embarrassed	Some discomfort/pain	Not confident
Not embarrassed	Some discomfort/pain	Neutral
Not embarrassed at all	No discomfort/pain at all	Very confident
Not embarrassed at all	No discomfort/pain at all	Very confident
Not embarrassed at all	No discomfort/pain at all	Very confident
Not embarrassed	No discomfort/pain	Confident
Not embarrassed	No discomfort/pain	Confident
Not embarrassed at all	No discomfort/pain at all	Very confident
Not embarrassed at all	No discomfort/pain at all	Confident
Not embarrassed at all	No discomfort/pain at all	Very confident
Not embarrassed	No discomfort/pain	Confident
Not embarrassed at all	No discomfort/pain	Confident
Not embarrassed	No discomfort/pain at all	Confident
Not embarrassed	No discomfort/pain	Confident
Not embarrassed at all	No discomfort/pain at all	Neutral
Not embarrassed at all	Some discomfort/pain	Very confident
Not embarrassed	No discomfort/pain	Confident
Not embarrassed	Some discomfort/pain	Confident
Not embarrassed at all	No discomfort/pain at all	Confident
Not embarrassed	No discomfort/pain	Confident
Neutral	No discomfort/pain	Not confident
Not embarrassed at all	No discomfort/pain at all	Very confident

Not embarrassed at all	No discomfort/pain at all	Very confident
Not embarrassed at all	No discomfort/pain at all	Confident
Not embarrassed at all	No discomfort/pain	Confident
Not embarrassed at all	Some discomfort/pain	Confident
Not embarrassed at all	No discomfort/pain at all	Very confident
Not embarrassed at all	No discomfort/pain	Confident
Not embarrassed	No discomfort/pain	Neutral
Not embarrassed at all	No discomfort/pain	Confident
Not embarrassed at all	No discomfort/pain at all	Confident
Not embarrassed	No discomfort/pain at all	Neutral
Not embarrassed at all	Some discomfort/pain	Confident
Not embarrassed at all	Some discomfort/pain	Neutral
Not embarrassed	Neutral	Confident
Not embarrassed at all	No discomfort/pain at all	Confident
Not embarrassed	Some discomfort/pain	Confident
Not embarrassed at all	No discomfort/pain	Confident
Not embarrassed at all	Some discomfort/pain	Confident
Not embarrassed at all	No discomfort/pain	Confident
Not embarrassed	Neutral	Confident
Not embarrassed	Some discomfort/pain	Confident
Not embarrassed at all	No discomfort/pain at all	Confident
Not embarrassed at all	Neutral	Neutral
Not embarrassed at all	No discomfort/pain at all	Confident
Not embarrassed at all	Neutral	Neutral
Not embarrassed	Some discomfort/pain	Confident
Not embarrassed at all	No discomfort/pain	Neutral
Not embarrassed	No discomfort/pain	Confident
Not embarrassed	No discomfort/pain	Confident
Not embarrassed at all	Some discomfort/pain	Neutral
Not embarrassed	No discomfort/pain	Very confident
Not embarrassed	No discomfort/pain	Confident
Not embarrassed	No discomfort/pain	Confident
Not embarrassed at all	No discomfort/pain	Confident

Not embarrassed at all	No discomfort/pain at all	Confident
Not embarrassed	Neutral	Confident
Not embarrassed at all	Some discomfort/pain	Confident
Not embarrassed	Some discomfort/pain	Confident
Not embarrassed	No discomfort/pain	Confident
Not embarrassed at all	No discomfort/pain at all	Very confident
Not embarrassed at all	No discomfort/pain at all	Confident
Not embarrassed	No discomfort/pain	Confident
Not embarrassed	No discomfort/pain	Confident
Not embarrassed at all	No discomfort/pain	Confident
Neutral	Some discomfort/pain	Not confident
Not embarrassed at all	Some discomfort/pain	Confident
Not embarrassed at all	No discomfort/pain	Very confident
Not embarrassed	Some discomfort/pain	Confident
Not embarrassed at all	Neutral	Confident
Not embarrassed	No discomfort/pain	Neutral
Not embarrassed at all	Some discomfort/pain	Neutral
Not embarrassed at all	No discomfort/pain at all	Very confident
Not embarrassed at all	No discomfort/pain at all	Confident
Not embarrassed	Some discomfort/pain	Confident
Not embarrassed at all	No discomfort/pain at all	Very confident
Not embarrassed	Some discomfort/pain	Confident
Not embarrassed at all	Some discomfort/pain	Confident
Not embarrassed	Some discomfort/pain	Confident
Not embarrassed at all	No discomfort/pain at all	Confident
Not embarrassed at all	No discomfort/pain at all	Confident
Not embarrassed at all	No discomfort/pain at all	Confident
Not embarrassed	No discomfort/pain	Confident
Not embarrassed	No discomfort/pain	Confident
Not embarrassed	No discomfort/pain	Confident
Not embarrassed at all	No discomfort/pain	Confident
Not embarrassed	No discomfort/pain	Confident
Not embarrassed at all	No discomfort/pain	Confident

Not embarrassed	No discomfort/pain	Confident
Not embarrassed	No discomfort/pain	Confident
Not embarrassed at all	No discomfort/pain at all	Very confident
Not embarrassed at all	No discomfort/pain at all	Confident
Not embarrassed	Some discomfort/pain	Confident
Not embarrassed	No discomfort/pain	Confident
Not embarrassed at all	No discomfort/pain at all	Confident
Not embarrassed at all	No discomfort/pain at all	Confident
Not embarrassed	Some discomfort/pain	Neutral
Not embarrassed	No discomfort/pain	Confident
Not embarrassed at all	No discomfort/pain at all	Very confident
Not embarrassed at all	No discomfort/pain at all	Confident
Not embarrassed	No discomfort/pain	Confident
Neutral	No discomfort/pain	Confident
Not embarrassed at all	No discomfort/pain at all	Confident
Not embarrassed at all	No discomfort/pain at all	Very confident
Not embarrassed	No discomfort/pain	Confident
Not embarrassed at all	No discomfort/pain	Confident
Not embarrassed	No discomfort/pain	Confident
Not embarrassed	Some discomfort/pain	Confident
Not embarrassed at all	Some discomfort/pain	Confident
Not embarrassed	No discomfort/pain	Confident
Not embarrassed at all	No discomfort/pain	Confident
Not embarrassed at all	No discomfort/pain	Confident
Not embarrassed at all	No discomfort/pain at all	Confident
Not embarrassed at all	No discomfort/pain	Neutral
Not embarrassed	No discomfort/pain	Confident
Not embarrassed at all	No discomfort/pain at all	Very confident
Embarrassed	No discomfort/pain at all	Confident
Not embarrassed at all	No discomfort/pain at all	Confident
Not embarrassed at all	No discomfort/pain at all	Confident
Not embarrassed	No discomfort/pain	Confident
Not embarrassed at all	Some discomfort/pain	Confident

Not embarrassed at all	No discomfort/pain at all	Confident
Not embarrassed at all	No discomfort/pain at all	Confident
Not embarrassed	No discomfort/pain	Confident
Not embarrassed at all	Neutral	Confident
Not embarrassed	Some discomfort/pain	Confident
Not embarrassed at all	No discomfort/pain	Neutral
Not embarrassed	No discomfort/pain	Confident
Not embarrassed	No discomfort/pain	Confident
Not embarrassed at all	Neutral	Confident
Not embarrassed at all	No discomfort/pain at all	Very confident
Not embarrassed	No discomfort/pain	Very confident
Not embarrassed at all	Some discomfort/pain	Confident
Not embarrassed at all	No discomfort/pain	Very confident
Not embarrassed at all	No discomfort/pain at all	Very confident
Not embarrassed	Some discomfort/pain	Confident
Not embarrassed	No discomfort/pain	Confident
Not embarrassed at all	No discomfort/pain at all	Confident
Not embarrassed at all	No discomfort/pain at all	Very confident
Not embarrassed	No discomfort/pain	Very confident
Not embarrassed at all	No discomfort/pain at all	Very confident
Not embarrassed	No discomfort/pain	Confident
Not embarrassed at all	No discomfort/pain at all	Confident
Not embarrassed at all	No discomfort/pain	Confident
Not embarrassed	Some discomfort/pain	Very confident
Not embarrassed	Some discomfort/pain	Confident
Not embarrassed at all	No discomfort/pain at all	Confident
Not embarrassed at all	No discomfort/pain	Confident
Not embarrassed	No discomfort/pain	Neutral
Not embarrassed at all	No discomfort/pain	Confident
Not embarrassed	No discomfort/pain	Confident
Not embarrassed at all	No discomfort/pain at all	Confident
Not embarrassed	No discomfort/pain	Confident
Not embarrassed at all	No discomfort/pain at all	Confident

Not embarrassed	Some discomfort/pain	Confident
Not embarrassed at all	Some discomfort/pain	Confident
Not embarrassed	Some discomfort/pain	Confident
Not embarrassed at all	Neutral	Neutral
Not embarrassed	Neutral	Confident
Not embarrassed at all	No discomfort/pain at all	Confident
Not embarrassed at all	No discomfort/pain at all	Very confident
Not embarrassed	No discomfort/pain	Confident
Not embarrassed at all	No discomfort/pain at all	Very confident
Not embarrassed at all	No discomfort/pain at all	Very confident
Not embarrassed at all	No discomfort/pain at all	Confident
Not embarrassed	No discomfort/pain	Confident
Not embarrassed at all	No discomfort/pain at all	Confident
Not embarrassed	No discomfort/pain	Confident
Not embarrassed at all	No discomfort/pain at all	Confident
Not embarrassed at all	No discomfort/pain at all	Very confident
Not embarrassed	No discomfort/pain	Confident
Not embarrassed	No discomfort/pain	Confident
Not embarrassed at all	No discomfort/pain at all	Confident
Not embarrassed	Some discomfort/pain	Confident
Not embarrassed	No discomfort/pain	Neutral
Not embarrassed at all	No discomfort/pain at all	Confident
Not embarrassed at all	No discomfort/pain at all	Confident
Not embarrassed	No discomfort/pain at all	Very confident
Not embarrassed at all	Neutral	Confident
Not embarrassed at all	No discomfort/pain at all	Confident
Not embarrassed	Some discomfort/pain	Confident
Not embarrassed at all	No discomfort/pain at all	Confident
Not embarrassed	No discomfort/pain at all	Confident
Not embarrassed	No discomfort/pain	Confident
Not embarrassed at all	No discomfort/pain at all	Very confident
Not embarrassed at all	Some discomfort/pain	Confident
Not embarrassed	No discomfort/pain	Confident

Not embarrassed at all	No discomfort/pain	Neutral
Not embarrassed at all	No discomfort/pain at all	Confident
Not embarrassed	No discomfort/pain	Neutral
Not embarrassed at all	No discomfort/pain at all	Confident
Not embarrassed at all	No discomfort/pain at all	Very confident
Not embarrassed	No discomfort/pain	Confident
Not embarrassed	No discomfort/pain	Very confident
Not embarrassed	No discomfort/pain	Very confident
Not embarrassed	Some discomfort/pain	Very confident
Not embarrassed	Some discomfort/pain	Confident
Not embarrassed	Some discomfort/pain	Confident
Not embarrassed	No discomfort/pain	Confident
Not embarrassed	No discomfort/pain	Confident
Not embarrassed	No discomfort/pain at all	Confident
Not embarrassed	No discomfort/pain	Confident
Not embarrassed	No discomfort/pain at all	Confident
Not embarrassed	No discomfort/pain	Confident
Neutral	Some discomfort/pain	Neutral
Not embarrassed at all	No discomfort/pain at all	Very confident
Not embarrassed	No discomfort/pain	Confident
Not embarrassed at all	No discomfort/pain at all	Very confident
Not embarrassed	Some discomfort/pain	Confident
Not embarrassed	Some discomfort/pain	Confident
Not embarrassed at all	No discomfort/pain at all	Very confident
Not embarrassed	Some discomfort/pain	Confident
Not embarrassed	No discomfort/pain	Confident
Not embarrassed	No discomfort/pain	Confident
Not embarrassed	No discomfort/pain	Confident
Not embarrassed	No discomfort/pain	Confident
Not embarrassed	No discomfort/pain	Confident
Embarrassed	No discomfort/pain	Very confident
Not embarrassed	No discomfort/pain at all	Neutral
Not embarrassed at all	No discomfort/pain at all	Very confident

Not embarrassed	No discomfort/pain	Confident
Not embarrassed	No discomfort/pain at all	Very confident
Not embarrassed	No discomfort/pain	Confident
Not embarrassed	No discomfort/pain	Confident
Not embarrassed	No discomfort/pain	Confident
Not embarrassed	No discomfort/pain	Confident
Not embarrassed	No discomfort/pain	Confident
Not embarrassed	No discomfort/pain	Confident
Not embarrassed	No discomfort/pain	Confident
Not embarrassed	No discomfort/pain	Confident
Not embarrassed at all	No discomfort/pain at all	Very confident
Not embarrassed	No discomfort/pain	Confident
Not embarrassed	No discomfort/pain	Confident
Not embarrassed	No discomfort/pain	Confident
Not embarrassed	No discomfort/pain	Confident
Not embarrassed	No discomfort/pain	Confident
Not embarrassed at all	No discomfort/pain at all	Very confident
Not embarrassed	Some discomfort/pain	Not confident
Not embarrassed at all	No discomfort/pain	Confident
Not embarrassed	No discomfort/pain	Very confident
Not embarrassed	No discomfort/pain	Confident
Not embarrassed	No discomfort/pain	Confident
Not embarrassed	No discomfort/pain	Confident
Not embarrassed	No discomfort/pain	Confident
Not embarrassed	No discomfort/pain	Confident
Not embarrassed	No discomfort/pain	Very confident
Not embarrassed	Some discomfort/pain	Not confident
Not embarrassed	No discomfort/pain	Confident
Not embarrassed	No discomfort/pain	Confident
Not embarrassed	Some discomfort/pain	Neutral
Not embarrassed	No discomfort/pain	Confident

Not embarrassed	No discomfort/pain	Very confident
Not embarrassed at all	No discomfort/pain at all	Very confident
Not embarrassed	Some discomfort/pain	Not confident
Not embarrassed	No discomfort/pain	Confident
Not embarrassed	No discomfort/pain	Confident
Not embarrassed	No discomfort/pain	Confident
Not embarrassed	No discomfort/pain	Neutral
Not embarrassed	No discomfort/pain	Confident
Not embarrassed	No discomfort/pain	Confident
Not embarrassed	No discomfort/pain	Confident
Not embarrassed	No discomfort/pain	Confident
Not embarrassed	No discomfort/pain	Confident
Not embarrassed	No discomfort/pain	Confident
Not embarrassed	No discomfort/pain	Confident
Not embarrassed	No discomfort/pain	Neutral
Not embarrassed	Some discomfort/pain	Very confident
Not embarrassed	No discomfort/pain	Very confident
Not embarrassed	No discomfort/pain	Confident
Not embarrassed	No discomfort/pain	Confident
Not embarrassed	No discomfort/pain	Confident
Not embarrassed	No discomfort/pain	Confident
Not embarrassed	No discomfort/pain	Confident
Not embarrassed	No discomfort/pain	Confident
Not embarrassed	No discomfort/pain	Confident
Not embarrassed	No discomfort/pain	Confident
Not embarrassed	Some discomfort/pain	Not confident
Not embarrassed	No discomfort/pain	Confident
Not embarrassed	No discomfort/pain	Confident
Not embarrassed	No discomfort/pain	Confident
Not embarrassed	Some discomfort/pain	Very confident
Not embarrassed	Some discomfort/pain	Confident
Not embarrassed	No discomfort/pain at all	Very confident

Not embarrassed	Some discomfort/pain	Confident
Not embarrassed at all	No discomfort/pain at all	Very confident
Not embarrassed	Some discomfort/pain	Confident
Not embarrassed	No discomfort/pain	Confident
Not embarrassed	No discomfort/pain	Confident
Not embarrassed at all	No discomfort/pain	Very confident
Not embarrassed	No discomfort/pain	Confident
Not embarrassed	No discomfort/pain	Confident
Not embarrassed	Severe discomfort/pain	Not confident
Not embarrassed	No discomfort/pain	Confident
Not embarrassed	No discomfort/pain	Confident
Not embarrassed	Some discomfort/pain	Confident
Not embarrassed	No discomfort/pain	Confident
Not embarrassed	No discomfort/pain	Confident
Not embarrassed at all	No discomfort/pain at all	Very confident
Not embarrassed	Some discomfort/pain	Not confident
Not embarrassed	No discomfort/pain	Confident
Not embarrassed	Some discomfort/pain	Confident
Not embarrassed	No discomfort/pain	Confident
Not embarrassed	Some discomfort/pain	Confident
Not embarrassed	No discomfort/pain	Confident
Not embarrassed	Some discomfort/pain	Confident
Not embarrassed	Some discomfort/pain	Confident
Not embarrassed	No discomfort/pain at all	Confident
Not embarrassed	No discomfort/pain	Confident
Not embarrassed	No discomfort/pain	Not confident
Not embarrassed	No discomfort/pain	Confident
Not embarrassed	No discomfort/pain	Confident
Not embarrassed	Some discomfort/pain	Confident
Not embarrassed	Some discomfort/pain	Confident
Not embarrassed	No discomfort/pain	Very confident
Not embarrassed	No discomfort/pain	Confident
Not embarrassed at all	Some discomfort/pain	Confident

Not embarrassed	No discomfort/pain	Confident
Not embarrassed	No discomfort/pain	Very confident
Not embarrassed	No discomfort/pain	Confident
Not embarrassed	Some discomfort/pain	Neutral
Not embarrassed at all	No discomfort/pain at all	Confident
Not embarrassed	Some discomfort/pain	Confident
Not embarrassed	Some discomfort/pain	Confident
Not embarrassed	No discomfort/pain	Confident
Not embarrassed	No discomfort/pain	Confident
Not embarrassed	No discomfort/pain	Confident
Not embarrassed	No discomfort/pain	Confident
Not embarrassed at all	Some discomfort/pain	Confident
Not embarrassed	No discomfort/pain	Confident
Not embarrassed	Some discomfort/pain	Neutral
Not embarrassed	No discomfort/pain	Confident
Not embarrassed	No discomfort/pain	Confident
Not embarrassed	Some discomfort/pain	Confident
Not embarrassed	No discomfort/pain	Confident
Not embarrassed	No discomfort/pain	Confident
Not embarrassed	Some discomfort/pain	Not confident
Not embarrassed	No discomfort/pain at all	Confident
Not embarrassed	Some discomfort/pain	Confident
Not embarrassed	Some discomfort/pain	Confident
Not embarrassed	Some discomfort/pain	Neutral
Not embarrassed	No discomfort/pain	Confident
Not embarrassed at all	No discomfort/pain	Confident
Not embarrassed	No discomfort/pain	Neutral
Not embarrassed	Some discomfort/pain	Confident
Not embarrassed	No discomfort/pain	Confident
Not embarrassed	Some discomfort/pain	Confident
Not embarrassed	No discomfort/pain	Neutral
Not embarrassed	Some discomfort/pain	Confident
Not embarrassed	Some discomfort/pain	Confident

Not embarrassed	No discomfort/pain at all	Very confident
Not embarrassed	No discomfort/pain	Confident
Not embarrassed	No discomfort/pain at all	Very confident
Not embarrassed	No discomfort/pain	Confident
Not embarrassed	No discomfort/pain at all	Very confident
Not embarrassed	No discomfort/pain	Confident
Not embarrassed	No discomfort/pain	Confident
Not embarrassed	No discomfort/pain	Confident
Not embarrassed	No discomfort/pain	Confident
Not embarrassed	No discomfort/pain	Confident
Not embarrassed	No discomfort/pain	Confident
Not embarrassed	No discomfort/pain	Very confident
Not embarrassed	Some discomfort/pain	Confident
Not embarrassed	No discomfort/pain	Confident
Not embarrassed	No discomfort/pain	Confident
Not embarrassed	No discomfort/pain	Confident
Not embarrassed	No discomfort/pain	Confident
Not embarrassed	No discomfort/pain at all	Neutral
Not embarrassed	No discomfort/pain	Confident
Not embarrassed	No discomfort/pain	Confident
Not embarrassed at all	No discomfort/pain at all	Confident
Not embarrassed	Some discomfort/pain	Confident
Not embarrassed	No discomfort/pain at all	Confident
Not embarrassed	No discomfort/pain	Confident
Not embarrassed	No discomfort/pain	Confident
Not embarrassed	No discomfort/pain	Confident
Not embarrassed	No discomfort/pain	Confident
Not embarrassed	No discomfort/pain	Confident
Not embarrassed	Some discomfort/pain	Confident
Not embarrassed	Some discomfort/pain	Confident
Not embarrassed	No discomfort/pain	Confident

Not embarrassed	No discomfort/pain at all	Very confident
Not embarrassed	No discomfort/pain	Neutral
Not embarrassed	No discomfort/pain	Confident
Not embarrassed	Some discomfort/pain	Confident
Not embarrassed at all	No discomfort/pain at all	Very confident
Not embarrassed	No discomfort/pain	Confident
Not embarrassed	No discomfort/pain at all	Confident
Not embarrassed at all	No discomfort/pain at all	Confident
Not embarrassed	No discomfort/pain	Confident
Not embarrassed	No discomfort/pain	Very confident
Not embarrassed	Some discomfort/pain	Confident
Not embarrassed at all	No discomfort/pain at all	Very confident
Not embarrassed	No discomfort/pain at all	Very confident
Not embarrassed	No discomfort/pain	Confident
Not embarrassed	No discomfort/pain	Confident
Not embarrassed	No discomfort/pain	Confident
Not embarrassed	Some discomfort/pain	Confident
Not embarrassed at all	No discomfort/pain at all	Confident
Not embarrassed	No discomfort/pain	Confident
Not embarrassed	No discomfort/pain	Confident
Not embarrassed at all	No discomfort/pain at all	Confident
Not embarrassed	No discomfort/pain	Confident
Not embarrassed at all	No discomfort/pain	Confident
Not embarrassed at all	No discomfort/pain at all	Confident
Not embarrassed at all	Some discomfort/pain	Confident
Not embarrassed	Some discomfort/pain	Confident
Not embarrassed	Some discomfort/pain	Confident
Not embarrassed	No discomfort/pain	Confident
Not embarrassed at all	No discomfort/pain at all	Very confident
Not embarrassed at all	No discomfort/pain	Confident
Not embarrassed at all	No discomfort/pain	Confident
Not embarrassed	No discomfort/pain	Confident

Not embarrassed	No discomfort/pain	Confident
Not embarrassed	No discomfort/pain	Confident
Not embarrassed	No discomfort/pain	Confident
Not embarrassed	No discomfort/pain	Confident
Not embarrassed	No discomfort/pain	Confident
Not embarrassed	No discomfort/pain	Very confident
Not embarrassed	No discomfort/pain	Confident
Not embarrassed	Some discomfort/pain	Confident
Not embarrassed	No discomfort/pain	Confident
Not embarrassed	No discomfort/pain	Confident
Not embarrassed	No discomfort/pain	Confident
Not embarrassed	No discomfort/pain	Confident
Not embarrassed	Some discomfort/pain	Confident
Not embarrassed	No discomfort/pain	Confident
Not embarrassed	No discomfort/pain	Confident
Not embarrassed	No discomfort/pain at all	Very confident
Not embarrassed	No discomfort/pain	Neutral
Not embarrassed	No discomfort/pain	Confident
Not embarrassed	Some discomfort/pain	Neutral
Not embarrassed	Some discomfort/pain	Not confident

Which method do you prefer the MOST for cervical screei	Would you be willing to do the HPV self-sampling test aga	HPV result (Cobas X4800)	Colposcopy date
Self-collecting vaginal swab for HPV testing	Yes	Negative	
Self-collecting vaginal swab for HPV testing	Yes	Negative	
Self-collecting vaginal swab for HPV testing	Yes	Negative	
Self-collecting vaginal swab for HPV testing	Yes	Negative	
Self-collecting vaginal swab for HPV testing	Yes	Positive Non-HPV 16/18	2/11/2021
		Negative	
Self-collecting vaginal swab for HPV testing	Yes	Negative	
Self-collecting vaginal swab for HPV testing	Yes	Invalid	
Self-collecting vaginal swab for HPV testing	Yes	Negative	
Self-collecting vaginal swab for HPV testing	Yes	Negative	
Healthcare professional-collected vaginal swab for HPV tes	Yes	Negative	
Self-collecting vaginal swab for HPV testing	Yes	Negative	
Self-collecting vaginal swab for HPV testing	Yes	Negative	
Self-collecting vaginal swab for HPV testing	Yes	Negative	
Self-collecting vaginal swab for HPV testing	Yes	Negative	
Self-collecting vaginal swab for HPV testing	Yes	Negative	
Self-collecting vaginal swab for HPV testing	Yes	Negative	
Self-collecting vaginal swab for HPV testing	Yes	Negative	
Self-collecting vaginal swab for HPV testing	Yes	Negative	
Self-collecting vaginal swab for HPV testing	Yes	Negative	
Self-collecting vaginal swab for HPV testing	Yes	Negative	
Self-collecting vaginal swab for HPV testing	Yes	Negative	
Self-collecting vaginal swab for HPV testing	Yes	Positive Non-HPV 16/18	14/12/2021
Healthcare professional-collected vaginal swab for HPV tes	No	Negative	
Healthcare professional-collected vaginal swab for HPV tes	Yes	Positive HPV 18	27/10/2021
Self-collecting vaginal swab for HPV testing	Yes	Negative	
Self-collecting vaginal swab for HPV testing	Yes	Negative	
Self-collecting vaginal swab for HPV testing	Yes	Negative	
Self-collecting vaginal swab for HPV testing	Yes	Negative	
Self-collecting vaginal swab for HPV testing	Yes	Negative	
Self-collecting vaginal swab for HPV testing	Yes	Negative	
Healthcare professional-collected vaginal swab for HPV tes	Yes	Positive Non-HPV 16/18	1/12/2022

		Negative	
		Negative	
Self-collecting vaginal swab for HPV testing	Yes	Positive Non-HPV 16/18	17/11/2021
Self-collecting vaginal swab for HPV testing	Yes	Negative	
Self-collecting vaginal swab for HPV testing	Yes	Negative	
Pap smear - physician conducting a speculum examination	Yes	Negative	
Self-collecting vaginal swab for HPV testing	Yes	Negative	
Self-collecting vaginal swab for HPV testing	Yes	Negative	
Self-collecting vaginal swab for HPV testing	Yes	Positive Non-HPV 16/18	15/12/2021
Self-collecting vaginal swab for HPV testing	Yes	Negative	
Self-collecting vaginal swab for HPV testing	Yes	Negative	
Self-collecting vaginal swab for HPV testing	Yes	Negative	
Healthcare professional-collected vaginal swab for HPV tes	Yes	Negative	
Self-collecting vaginal swab for HPV testing	Yes	Negative	
Self-collecting vaginal swab for HPV testing	Yes	Negative	
No preference	Yes	Negative	
Self-collecting vaginal swab for HPV testing	Yes	Negative	
No preference	Yes	Negative	
Self-collecting vaginal swab for HPV testing	Yes	Negative	
Self-collecting vaginal swab for HPV testing	Yes	Negative	
No preference	Yes	Negative	
Healthcare professional-collected vaginal swab for HPV tes	Yes	Negative	
Self-collecting vaginal swab for HPV testing	Yes	Negative	
Self-collecting vaginal swab for HPV testing	Yes	Positive Non-HPV 16/18	15/2/2022
Self-collecting vaginal swab for HPV testing	Yes	Negative	
Self-collecting vaginal swab for HPV testing	Yes	Negative	
Self-collecting vaginal swab for HPV testing	Yes	Negative	
		Negative	
Self-collecting vaginal swab for HPV testing	Yes	Negative	
Self-collecting vaginal swab for HPV testing	Yes	Positive Non-HPV 16/18	
Self-collecting vaginal swab for HPV testing	Yes	Negative	
Self-collecting vaginal swab for HPV testing	Yes	Positive Non-HPV 16/18	11/1/2022
Self-collecting vaginal swab for HPV testing	Yes	Negative	

Self-collecting vaginal swab for HPV testing	Yes	Positive Non-HPV 16/18	20/4/2022
Self-collecting vaginal swab for HPV testing	Yes	Negative	
Healthcare professional-collected vaginal swab for HPV tes	Yes	Negative	
Self-collecting vaginal swab for HPV testing	Yes	Negative	
Self-collecting vaginal swab for HPV testing	Yes	Negative	
Self-collecting vaginal swab for HPV testing	Yes	Negative	
Self-collecting vaginal swab for HPV testing	Yes	Negative	
Pap smear - physician conducting a speculum examination	Yes	Negative	
Self-collecting vaginal swab for HPV testing	Yes	Negative	
Self-collecting vaginal swab for HPV testing	Yes	Negative	
Self-collecting vaginal swab for HPV testing	Yes	Negative	
Self-collecting vaginal swab for HPV testing	Yes	Negative	
Self-collecting vaginal swab for HPV testing	Yes	Negative	
Self-collecting vaginal swab for HPV testing	Yes	Negative	
Self-collecting vaginal swab for HPV testing	Yes	Negative	
Self-collecting vaginal swab for HPV testing	Yes	Negative	
Self-collecting vaginal swab for HPV testing	Yes	Negative	
Self-collecting vaginal swab for HPV testing	Yes	Negative	
Self-collecting vaginal swab for HPV testing	Yes	Negative	
Pap smear - physician conducting a speculum examination	Yes	Negative	
Self-collecting vaginal swab for HPV testing	Yes	Negative	
Self-collecting vaginal swab for HPV testing	Yes	Negative	
Healthcare professional-collected vaginal swab for HPV tes	Yes	Negative	
Self-collecting vaginal swab for HPV testing	Yes	Invalid	
Self-collecting vaginal swab for HPV testing	Yes	Negative	
Self-collecting vaginal swab for HPV testing	Yes	Positive Non-HPV 16/18	2/3/2022
Self-collecting vaginal swab for HPV testing	Yes	Negative	
Self-collecting vaginal swab for HPV testing	Yes	Negative	
Self-collecting vaginal swab for HPV testing	Yes	Negative	
Self-collecting vaginal swab for HPV testing	Yes	Negative	
Self-collecting vaginal swab for HPV testing	Yes	Negative	
Self-collecting vaginal swab for HPV testing	Yes	Negative	
Self-collecting vaginal swab for HPV testing	Yes	Negative	

Self-collecting vaginal swab for HPV testing	Yes	Negative	
Self-collecting vaginal swab for HPV testing	Yes	Negative	
Self-collecting vaginal swab for HPV testing	Yes	Positive Non-HPV 16/18	
Self-collecting vaginal swab for HPV testing	Yes	Positive HPV 16	20/7/2022
Self-collecting vaginal swab for HPV testing	Yes	Negative	
Self-collecting vaginal swab for HPV testing	Yes	Negative	
Self-collecting vaginal swab for HPV testing	Yes	Positive HPV 16	15/3/2022
Self-collecting vaginal swab for HPV testing	Yes	Positive Non-HPV 16/18	4/1/2022
Self-collecting vaginal swab for HPV testing	Yes	Negative	
Self-collecting vaginal swab for HPV testing	Yes	Positive Non-HPV 16/18	8/3/2022
Self-collecting vaginal swab for HPV testing	Yes	Negative	
Self-collecting vaginal swab for HPV testing	Yes	Negative	
Healthcare professional-collected vaginal swab for HPV tes	Yes	Negative	
Self-collecting vaginal swab for HPV testing	Yes	Negative	
Self-collecting vaginal swab for HPV testing	Yes	Negative	
Self-collecting vaginal swab for HPV testing	Yes	Negative	
Self-collecting vaginal swab for HPV testing	Yes	Negative	
Self-collecting vaginal swab for HPV testing	Yes	Negative	
Self-collecting vaginal swab for HPV testing	Yes	Negative	
Self-collecting vaginal swab for HPV testing	Yes	Invalid	
Self-collecting vaginal swab for HPV testing	Yes	Negative	
Self-collecting vaginal swab for HPV testing	Yes	Negative	
Self-collecting vaginal swab for HPV testing	Yes	Positive HPV 16 & Non-HPV 16/18	15/2/2022
Self-collecting vaginal swab for HPV testing	Yes	Negative	
Self-collecting vaginal swab for HPV testing	Yes	Negative	
Self-collecting vaginal swab for HPV testing	Yes	Negative	
Self-collecting vaginal swab for HPV testing	Yes	Negative	
Self-collecting vaginal swab for HPV testing	Yes	Negative	
Self-collecting vaginal swab for HPV testing	Yes	Negative	
Self-collecting vaginal swab for HPV testing	Yes	Negative	
Self-collecting vaginal swab for HPV testing	Yes	Negative	
Self-collecting vaginal swab for HPV testing	Yes	Negative	
Self-collecting vaginal swab for HPV testing	Yes	Negative	

Self-collecting vaginal swab for HPV testing	Yes	Negative	
Self-collecting vaginal swab for HPV testing	Yes	Negative	
Self-collecting vaginal swab for HPV testing	Yes	Negative	
Self-collecting vaginal swab for HPV testing	Yes	Negative	
Self-collecting vaginal swab for HPV testing	Yes	Negative	
Self-collecting vaginal swab for HPV testing	Yes	Negative	
Self-collecting vaginal swab for HPV testing	Yes	Negative	
Self-collecting vaginal swab for HPV testing	Yes	Negative	
Self-collecting vaginal swab for HPV testing	Yes	Negative	
Healthcare professional-collected vaginal swab for HPV tes	Yes	Negative	
Self-collecting vaginal swab for HPV testing	Yes	Negative	
Self-collecting vaginal swab for HPV testing	Yes	Positive Non-HPV 16/18	9/2/2022
Self-collecting vaginal swab for HPV testing	Yes	Negative	
Self-collecting vaginal swab for HPV testing	Yes	Negative	
Self-collecting vaginal swab for HPV testing	Yes	Negative	
Self-collecting vaginal swab for HPV testing	No	Negative	
Self-collecting vaginal swab for HPV testing	Yes	Negative	
Healthcare professional-collected vaginal swab for HPV tes	Yes	Negative	
Self-collecting vaginal swab for HPV testing	Yes	Negative	
Self-collecting vaginal swab for HPV testing	Yes	Negative	
Self-collecting vaginal swab for HPV testing	Yes	Negative	
Self-collecting vaginal swab for HPV testing	Yes	Negative	
Self-collecting vaginal swab for HPV testing	Yes	Negative	
Self-collecting vaginal swab for HPV testing	Yes	Negative	
Self-collecting vaginal swab for HPV testing	Yes	Negative	
Self-collecting vaginal swab for HPV testing	Yes	Negative	
Self-collecting vaginal swab for HPV testing	Yes	Negative	
Self-collecting vaginal swab for HPV testing	Yes	Negative	
Self-collecting vaginal swab for HPV testing	Yes	Negative	
Healthcare professional-collected vaginal swab for HPV tes	Yes	Positive Non-HPV 16/18	8/2/2022
Self-collecting vaginal swab for HPV testing	Yes	Negative	
Self-collecting vaginal swab for HPV testing	Yes	Negative	
Self-collecting vaginal swab for HPV testing	Yes	Negative	

Self-collecting vaginal swab for HPV testing	Yes	Negative
Self-collecting vaginal swab for HPV testing	Yes	Negative
Self-collecting vaginal swab for HPV testing	No	Negative
Self-collecting vaginal swab for HPV testing	Yes	Negative
Healthcare professional-collected vaginal swab for HPV tes	Yes	Negative
Self-collecting vaginal swab for HPV testing	Yes	Negative
Self-collecting vaginal swab for HPV testing	Yes	Negative
Self-collecting vaginal swab for HPV testing	Yes	Negative
Healthcare professional-collected vaginal swab for HPV tes	No	Negative
Self-collecting vaginal swab for HPV testing	Yes	Negative
Self-collecting vaginal swab for HPV testing	Yes	Negative
Self-collecting vaginal swab for HPV testing	Yes	Negative
Self-collecting vaginal swab for HPV testing	Yes	Negative
Self-collecting vaginal swab for HPV testing	Yes	Negative
Self-collecting vaginal swab for HPV testing	Yes	Negative
Self-collecting vaginal swab for HPV testing	Yes	Negative
Self-collecting vaginal swab for HPV testing	Yes	Negative
Self-collecting vaginal swab for HPV testing	Yes	Negative
Self-collecting vaginal swab for HPV testing	Yes	Negative
Self-collecting vaginal swab for HPV testing	Yes	Negative
Self-collecting vaginal swab for HPV testing	Yes	Negative
Self-collecting vaginal swab for HPV testing	Yes	Negative
Self-collecting vaginal swab for HPV testing	Yes	Negative
Self-collecting vaginal swab for HPV testing	Yes	Negative
Self-collecting vaginal swab for HPV testing	Yes	Negative
Self-collecting vaginal swab for HPV testing	Yes	Negative
Self-collecting vaginal swab for HPV testing	Yes	Negative
Healthcare professional-collected vaginal swab for HPV tes	Yes	Negative
Self-collecting vaginal swab for HPV testing	Yes	Negative
Self-collecting vaginal swab for HPV testing	Yes	Negative
Self-collecting vaginal swab for HPV testing	Yes	Negative
Self-collecting vaginal swab for HPV testing	Yes	Negative
Self-collecting vaginal swab for HPV testing	Yes	Negative

Self-collecting vaginal swab for HPV testing	Yes	Negative
Self-collecting vaginal swab for HPV testing	Yes	Negative
Self-collecting vaginal swab for HPV testing	Yes	Negative
Self-collecting vaginal swab for HPV testing	Yes	Negative
Self-collecting vaginal swab for HPV testing	Yes	Negative
Self-collecting vaginal swab for HPV testing	Yes	Negative
Self-collecting vaginal swab for HPV testing	Yes	Negative
Healthcare professional-collected vaginal swab for HPV tes	Yes	Negative
Self-collecting vaginal swab for HPV testing	Yes	Negative
Self-collecting vaginal swab for HPV testing	Yes	Negative
Self-collecting vaginal swab for HPV testing	Yes	Negative
Self-collecting vaginal swab for HPV testing	Yes	Negative
Self-collecting vaginal swab for HPV testing	Yes	Negative
Self-collecting vaginal swab for HPV testing	Yes	Negative
Self-collecting vaginal swab for HPV testing	Yes	Negative
Self-collecting vaginal swab for HPV testing	Yes	Negative
Self-collecting vaginal swab for HPV testing	Yes	Negative
Self-collecting vaginal swab for HPV testing	Yes	Negative
Self-collecting vaginal swab for HPV testing	Yes	Negative
Healthcare professional-collected vaginal swab for HPV tes	Yes	Invalid
Self-collecting vaginal swab for HPV testing	Yes	Negative
Self-collecting vaginal swab for HPV testing	Yes	Negative
Self-collecting vaginal swab for HPV testing	Yes	Negative
Self-collecting vaginal swab for HPV testing	Yes	Negative
Self-collecting vaginal swab for HPV testing	Yes	Negative
Self-collecting vaginal swab for HPV testing	Yes	Negative
Self-collecting vaginal swab for HPV testing	Yes	Negative
Self-collecting vaginal swab for HPV testing	Yes	Negative
Self-collecting vaginal swab for HPV testing	Yes	Negative
Self-collecting vaginal swab for HPV testing	Yes	Negative
Self-collecting vaginal swab for HPV testing	Yes	Negative
Self-collecting vaginal swab for HPV testing	Yes	Negative
Self-collecting vaginal swab for HPV testing	Yes	Negative

Self-collecting vaginal swab for HPV testing	Yes	Negative	
Self-collecting vaginal swab for HPV testing	Yes	Negative	
Self-collecting vaginal swab for HPV testing	Yes	Negative	
Self-collecting vaginal swab for HPV testing	Yes	Negative	
Self-collecting vaginal swab for HPV testing	Yes	Negative	
Self-collecting vaginal swab for HPV testing	Yes	Negative	
Self-collecting vaginal swab for HPV testing	Yes	Negative	
Self-collecting vaginal swab for HPV testing	Yes	Negative	
Self-collecting vaginal swab for HPV testing	Yes	Negative	
Self-collecting vaginal swab for HPV testing	Yes	Negative	
Self-collecting vaginal swab for HPV testing	Yes	Positive Non-HPV 16/18	5/10/2022
Self-collecting vaginal swab for HPV testing	Yes	Negative	
Self-collecting vaginal swab for HPV testing	Yes	Negative	
Self-collecting vaginal swab for HPV testing	Yes	Negative	
Self-collecting vaginal swab for HPV testing	Yes	Negative	
Self-collecting vaginal swab for HPV testing	Yes	Negative	
Self-collecting vaginal swab for HPV testing	Yes	Negative	
Self-collecting vaginal swab for HPV testing	Yes	Negative	
Self-collecting vaginal swab for HPV testing	Yes	Invalid	
Self-collecting vaginal swab for HPV testing	Yes	Invalid	
Self-collecting vaginal swab for HPV testing	Yes	Negative	
Self-collecting vaginal swab for HPV testing	Yes	Positive Non-HPV 16/18	5/7/2023
Self-collecting vaginal swab for HPV testing	Yes	Negative	
Self-collecting vaginal swab for HPV testing	Yes	Negative	
Self-collecting vaginal swab for HPV testing	Yes	Negative	
Self-collecting vaginal swab for HPV testing	Yes	Negative	
Self-collecting vaginal swab for HPV testing	Yes	Negative	
Self-collecting vaginal swab for HPV testing	Yes	Negative	
Self-collecting vaginal swab for HPV testing	Yes	Negative	
Self-collecting vaginal swab for HPV testing	Yes	Negative	
Self-collecting vaginal swab for HPV testing	Yes	Negative	
Self-collecting vaginal swab for HPV testing	Yes	Negative	
Self-collecting vaginal swab for HPV testing	Yes	Positive Non-HPV 16/18	

Self-collecting vaginal swab for HPV testing	Yes	Negative
Self-collecting vaginal swab for HPV testing	Yes	Negative
Self-collecting vaginal swab for HPV testing	Yes	Negative
Self-collecting vaginal swab for HPV testing	Yes	Negative
Self-collecting vaginal swab for HPV testing	Yes	Negative
Self-collecting vaginal swab for HPV testing	Yes	Negative
Healthcare professional-collected vaginal swab for HPV tes	Yes	Negative
Self-collecting vaginal swab for HPV testing	Yes	Negative
Self-collecting vaginal swab for HPV testing	Yes	Negative
Self-collecting vaginal swab for HPV testing	Yes	Negative
Self-collecting vaginal swab for HPV testing	Yes	Negative
Self-collecting vaginal swab for HPV testing	Yes	Negative
Self-collecting vaginal swab for HPV testing	Yes	Negative
Self-collecting vaginal swab for HPV testing	Yes	Negative
Self-collecting vaginal swab for HPV testing	Yes	Negative
Self-collecting vaginal swab for HPV testing	Yes	Negative
Self-collecting vaginal swab for HPV testing	Yes	Negative
Self-collecting vaginal swab for HPV testing	Yes	Negative
Self-collecting vaginal swab for HPV testing	Yes	Negative
Self-collecting vaginal swab for HPV testing	Yes	Negative
Self-collecting vaginal swab for HPV testing	Yes	Negative
Self-collecting vaginal swab for HPV testing	Yes	Negative
Self-collecting vaginal swab for HPV testing	Yes	Negative
Self-collecting vaginal swab for HPV testing	Yes	Negative
Self-collecting vaginal swab for HPV testing	Yes	Negative
Self-collecting vaginal swab for HPV testing	Yes	Negative
Self-collecting vaginal swab for HPV testing	Yes	Negative
Healthcare professional-collected vaginal swab for HPV tes	Yes	Negative
Self-collecting vaginal swab for HPV testing	Yes	Negative
Self-collecting vaginal swab for HPV testing	Yes	Negative
Self-collecting vaginal swab for HPV testing	Yes	Negative
Self-collecting vaginal swab for HPV testing	Yes	Invalid
Self-collecting vaginal swab for HPV testing	Yes	Negative

Self-collecting vaginal swab for HPV testing	Yes	Negative	
Healthcare professional-collected vaginal swab for HPV tes	Yes	Negative	
Self-collecting vaginal swab for HPV testing	Yes	Negative	
Self-collecting vaginal swab for HPV testing	Yes	Negative	
Self-collecting vaginal swab for HPV testing	Yes	Negative	
Self-collecting vaginal swab for HPV testing	Yes	Negative	
Healthcare professional-collected vaginal swab for HPV tes	Yes	Negative	
Self-collecting vaginal swab for HPV testing	Yes	Negative	
Self-collecting vaginal swab for HPV testing	Yes	Negative	
Self-collecting vaginal swab for HPV testing	Yes	Negative	
Self-collecting vaginal swab for HPV testing	Yes	Negative	
Healthcare professional-collected vaginal swab for HPV tes	Yes	Positive Non-HPV 16/18	
Self-collecting vaginal swab for HPV testing	Yes	Negative	
Self-collecting vaginal swab for HPV testing	Yes	Negative	
Self-collecting vaginal swab for HPV testing	Yes	Negative	
Self-collecting vaginal swab for HPV testing	Yes	Negative	
Self-collecting vaginal swab for HPV testing	Yes	Negative	
Self-collecting vaginal swab for HPV testing	Yes	Negative	
Healthcare professional-collected vaginal swab for HPV tes	Yes	Negative	
Self-collecting vaginal swab for HPV testing	Yes	Negative	
Self-collecting vaginal swab for HPV testing	Yes	Positive HPV 16	15/8/2023
Self-collecting vaginal swab for HPV testing	Yes	Negative	
Self-collecting vaginal swab for HPV testing	Yes	Negative	
Self-collecting vaginal swab for HPV testing	Yes	Negative	
Self-collecting vaginal swab for HPV testing	Yes	Negative	
Healthcare professional-collected vaginal swab for HPV tes	Yes	Negative	
Self-collecting vaginal swab for HPV testing	Yes	Negative	
Self-collecting vaginal swab for HPV testing	Yes	Negative	
Self-collecting vaginal swab for HPV testing	Yes	Negative	
Self-collecting vaginal swab for HPV testing	Yes	Negative	
Self-collecting vaginal swab for HPV testing	Yes	Invalid	
Self-collecting vaginal swab for HPV testing	Yes	Positive Non-HPV 16/18	7/11/2023
Self-collecting vaginal swab for HPV testing	Yes	Negative	

Self-collecting vaginal swab for HPV testing	Yes	Invalid	
Self-collecting vaginal swab for HPV testing	Yes	Invalid	
Self-collecting vaginal swab for HPV testing	Yes	Negative	
Healthcare professional-collected vaginal swab for HPV tes	Yes	Negative	
Self-collecting vaginal swab for HPV testing	Yes	Negative	
Self-collecting vaginal swab for HPV testing	Yes	Negative	
Self-collecting vaginal swab for HPV testing	Yes	Negative	
Self-collecting vaginal swab for HPV testing	Yes	Negative	
Self-collecting vaginal swab for HPV testing	Yes	Negative	
Self-collecting vaginal swab for HPV testing	Yes	Negative	
Self-collecting vaginal swab for HPV testing	Yes	Negative	
Self-collecting vaginal swab for HPV testing	Yes	Positive Non-HPV 16/18	24/10/2023
Self-collecting vaginal swab for HPV testing	Yes	Negative	
Self-collecting vaginal swab for HPV testing	Yes	Negative	
Self-collecting vaginal swab for HPV testing	Yes	Negative	
Self-collecting vaginal swab for HPV testing	Yes	Negative	
Self-collecting vaginal swab for HPV testing	Yes	Negative	
Healthcare professional-collected vaginal swab for HPV tes	No	Negative	
Self-collecting vaginal swab for HPV testing	Yes	Negative	
Self-collecting vaginal swab for HPV testing	Yes	Negative	
Self-collecting vaginal swab for HPV testing	Yes	Positive Non-HPV 16/18	30/11/2022
Self-collecting vaginal swab for HPV testing	Yes	Invalid	
Self-collecting vaginal swab for HPV testing	Yes	Negative	
Self-collecting vaginal swab for HPV testing	Yes	Negative	
Self-collecting vaginal swab for HPV testing	Yes	Positive Non-HPV 16/18	17/1/2022
Healthcare professional-collected vaginal swab for HPV tes	Yes	Negative	
Self-collecting vaginal swab for HPV testing	Yes	Negative	
Self-collecting vaginal swab for HPV testing	Yes	Negative	
Self-collecting vaginal swab for HPV testing	Yes	Negative	
Self-collecting vaginal swab for HPV testing	Yes	Negative	
Self-collecting vaginal swab for HPV testing	Yes	Negative	
Self-collecting vaginal swab for HPV testing	Yes	Negative	
Self-collecting vaginal swab for HPV testing	Yes	Negative	

Self-collecting vaginal swab for HPV testing	Yes	Negative	
Self-collecting vaginal swab for HPV testing	Yes	Negative	
Self-collecting vaginal swab for HPV testing	Yes	Negative	
Self-collecting vaginal swab for HPV testing	Yes	Negative	
Self-collecting vaginal swab for HPV testing	Yes	Negative	
Healthcare professional-collected vaginal swab for HPV tes	Yes	Negative	
Self-collecting vaginal swab for HPV testing	Yes	Negative	
Self-collecting vaginal swab for HPV testing	Yes	Negative	
Self-collecting vaginal swab for HPV testing	Yes	Invalid	
Self-collecting vaginal swab for HPV testing	Yes	Negative	
Self-collecting vaginal swab for HPV testing	Yes	Negative	
Healthcare professional-collected vaginal swab for HPV tes	No	Positive Non-HPV 16/18	21/2/2023
Self-collecting vaginal swab for HPV testing	Yes	Negative	
Self-collecting vaginal swab for HPV testing	Yes	Positive Non-HPV 16/18	
Self-collecting vaginal swab for HPV testing	Yes	Negative	
Healthcare professional-collected vaginal swab for HPV tes	Yes	Negative	
Self-collecting vaginal swab for HPV testing	Yes	Negative	
Self-collecting vaginal swab for HPV testing	Yes	Negative	
Healthcare professional-collected vaginal swab for HPV tes	Yes	Invalid	
Self-collecting vaginal swab for HPV testing	Yes	Negative	
Self-collecting vaginal swab for HPV testing	Yes	Invalid	
Self-collecting vaginal swab for HPV testing	Yes	Negative	
Healthcare professional-collected vaginal swab for HPV tes	Yes	Positive HPV 16	9/8/2023
Self-collecting vaginal swab for HPV testing	Yes	Negative	
No preference	Yes	Negative	
Self-collecting vaginal swab for HPV testing	Yes	Negative	
Self-collecting vaginal swab for HPV testing	Yes	Negative	
Self-collecting vaginal swab for HPV testing	Yes	Negative	
Self-collecting vaginal swab for HPV testing	Yes	Negative	
Healthcare professional-collected vaginal swab for HPV tes	No	Negative	
Self-collecting vaginal swab for HPV testing	Yes	Negative	
Self-collecting vaginal swab for HPV testing	Yes	Negative	
Self-collecting vaginal swab for HPV testing	Yes	Negative	

Self-collecting vaginal swab for HPV testing	Yes	Invalid	
		Positive Non-HPV 16/18	23/5/2023
Self-collecting vaginal swab for HPV testing	Yes	Negative	
		Negative	
		Negative	
		Negative	
Self-collecting vaginal swab for HPV testing	Yes	Negative	
Healthcare professional-collected vaginal swab for HPV tes	Yes	Negative	
No preference	Yes	Negative	
Self-collecting vaginal swab for HPV testing	Yes	Negative	
Healthcare professional-collected vaginal swab for HPV tes	Yes	Invalid	
Self-collecting vaginal swab for HPV testing	Yes	Negative	
Healthcare professional-collected vaginal swab for HPV tes	Yes	Negative	
Healthcare professional-collected vaginal swab for HPV tes	Yes	Negative	
Healthcare professional-collected vaginal swab for HPV tes	Yes	Positive Non-HPV 16/18	7/11/2023
Healthcare professional-collected vaginal swab for HPV tes	Yes	Negative	
Self-collecting vaginal swab for HPV testing	Yes	Negative	
Self-collecting vaginal swab for HPV testing	Yes	Negative	
Self-collecting vaginal swab for HPV testing	Yes	Negative	
Self-collecting vaginal swab for HPV testing	Yes	Negative	
Self-collecting vaginal swab for HPV testing	Yes	Negative	
Self-collecting vaginal swab for HPV testing	Yes	Negative	
Healthcare professional-collected vaginal swab for HPV tes	Yes	Negative	
Self-collecting vaginal swab for HPV testing	Yes	Negative	
Self-collecting vaginal swab for HPV testing	Yes	Invalid	
Healthcare professional-collected vaginal swab for HPV tes	Yes	Positive Non-HPV 16/18	15/3/2023
Healthcare professional-collected vaginal swab for HPV tes	No	Negative	
Self-collecting vaginal swab for HPV testing	Yes	Negative	
Self-collecting vaginal swab for HPV testing	Yes	Negative	
Healthcare professional-collected vaginal swab for HPV tes	No	Negative	
Self-collecting vaginal swab for HPV testing	Yes	Negative	
Self-collecting vaginal swab for HPV testing	Yes	Negative	
Self-collecting vaginal swab for HPV testing	Yes	Negative	

Self-collecting vaginal swab for HPV testing	Yes	Invalid	
Self-collecting vaginal swab for HPV testing	Yes	Invalid	
Self-collecting vaginal swab for HPV testing	Yes	Positive Non-HPV 16/18	15/8/2023
Self-collecting vaginal swab for HPV testing	Yes	Negative	
Self-collecting vaginal swab for HPV testing	Yes	Negative	
Self-collecting vaginal swab for HPV testing	Yes	Negative	
Self-collecting vaginal swab for HPV testing	Yes	Negative	
Healthcare professional-collected vaginal swab for HPV tes	Yes	Negative	
Self-collecting vaginal swab for HPV testing	Yes	Negative	
Self-collecting vaginal swab for HPV testing	Yes	Negative	
Self-collecting vaginal swab for HPV testing	Yes	Invalid	
Self-collecting vaginal swab for HPV testing	Yes	Negative	
Self-collecting vaginal swab for HPV testing	Yes	Negative	
Self-collecting vaginal swab for HPV testing	Yes	Negative	
Self-collecting vaginal swab for HPV testing	Yes	Negative	
Self-collecting vaginal swab for HPV testing	Yes	Negative	
Self-collecting vaginal swab for HPV testing	Yes	Negative	
Self-collecting vaginal swab for HPV testing	Yes	Negative	
Self-collecting vaginal swab for HPV testing	Yes	Negative	
Self-collecting vaginal swab for HPV testing	Yes	Negative	
Healthcare professional-collected vaginal swab for HPV tes	Yes	Negative	
Self-collecting vaginal swab for HPV testing	Yes	Negative	
Self-collecting vaginal swab for HPV testing	Yes	Negative	
Healthcare professional-collected vaginal swab for HPV tes	Yes	Negative	
Healthcare professional-collected vaginal swab for HPV tes	Yes	Invalid	
Self-collecting vaginal swab for HPV testing	Yes	Negative	
Self-collecting vaginal swab for HPV testing	Yes	Negative	
Self-collecting vaginal swab for HPV testing	Yes	Negative	
Self-collecting vaginal swab for HPV testing	Yes	Negative	
Self-collecting vaginal swab for HPV testing	Yes	Negative	
Self-collecting vaginal swab for HPV testing	Yes	Positive Non-HPV 16/18	16/5/2023
Self-collecting vaginal swab for HPV testing	Yes	Positive HPV 16	6/6/2023
Healthcare professional-collected vaginal swab for HPV tes	Yes	Negative	

Self-collecting vaginal swab for HPV testing	Yes	Negative
Self-collecting vaginal swab for HPV testing	Yes	Negative
Self-collecting vaginal swab for HPV testing	Yes	Negative
Self-collecting vaginal swab for HPV testing	Yes	Negative
Self-collecting vaginal swab for HPV testing	Yes	Negative
Self-collecting vaginal swab for HPV testing	Yes	Negative
Self-collecting vaginal swab for HPV testing	Yes	Negative
Self-collecting vaginal swab for HPV testing	Yes	Negative
Self-collecting vaginal swab for HPV testing	Yes	Negative
Self-collecting vaginal swab for HPV testing	Yes	Negative
Self-collecting vaginal swab for HPV testing	Yes	Negative
Self-collecting vaginal swab for HPV testing	Yes	Negative
Self-collecting vaginal swab for HPV testing	Yes	Invalid
Self-collecting vaginal swab for HPV testing	Yes	Negative
Self-collecting vaginal swab for HPV testing	Yes	Negative
Self-collecting vaginal swab for HPV testing	Yes	Negative
Healthcare professional-collected vaginal swab for HPV tes	Yes	Negative
Self-collecting vaginal swab for HPV testing	Yes	Negative
Self-collecting vaginal swab for HPV testing	Yes	Negative
Self-collecting vaginal swab for HPV testing	Yes	Negative
Healthcare professional-collected vaginal swab for HPV tes	Yes	Invalid
Self-collecting vaginal swab for HPV testing	Yes	Negative
Self-collecting vaginal swab for HPV testing	Yes	Negative
Healthcare professional-collected vaginal swab for HPV tes	Yes	Negative
Self-collecting vaginal swab for HPV testing	Yes	Negative
Healthcare professional-collected vaginal swab for HPV tes	Yes	Negative
Self-collecting vaginal swab for HPV testing	Yes	Negative
Self-collecting vaginal swab for HPV testing	Yes	Negative
Self-collecting vaginal swab for HPV testing	Yes	Negative
Self-collecting vaginal swab for HPV testing	Yes	Invalid
Self-collecting vaginal swab for HPV testing	Yes	Negative
Self-collecting vaginal swab for HPV testing	Yes	Negative
Self-collecting vaginal swab for HPV testing	Yes	Negative

Self-collecting vaginal swab for HPV testing	Yes	Negative	
Self-collecting vaginal swab for HPV testing	Yes	Negative	
Self-collecting vaginal swab for HPV testing	Yes	Negative	
Self-collecting vaginal swab for HPV testing	Yes	Negative	
Self-collecting vaginal swab for HPV testing	Yes	Negative	
Self-collecting vaginal swab for HPV testing	Yes	Negative	
Self-collecting vaginal swab for HPV testing	Yes	Negative	
Self-collecting vaginal swab for HPV testing	Yes	Negative	
Self-collecting vaginal swab for HPV testing	Yes	Positive Non-HPV 16/18	29/11/2023
Self-collecting vaginal swab for HPV testing	Yes	Negative	
Self-collecting vaginal swab for HPV testing	Yes	Negative	
Self-collecting vaginal swab for HPV testing	Yes	Negative	
Self-collecting vaginal swab for HPV testing	Yes	Positive Non-HPV 16/18	
Self-collecting vaginal swab for HPV testing	Yes	Negative	
Self-collecting vaginal swab for HPV testing	Yes	Negative	
Healthcare professional-collected vaginal swab for HPV tes	Yes	Negative	
Self-collecting vaginal swab for HPV testing	Yes	Negative	
Self-collecting vaginal swab for HPV testing	Yes	Negative	
Healthcare professional-collected vaginal swab for HPV tes	Yes	Negative	
Self-collecting vaginal swab for HPV testing	Yes	Negative	
Self-collecting vaginal swab for HPV testing	Yes	Negative	
Self-collecting vaginal swab for HPV testing	Yes	Negative	
Self-collecting vaginal swab for HPV testing	Yes	Negative	
Self-collecting vaginal swab for HPV testing	Yes	Negative	
Self-collecting vaginal swab for HPV testing	Yes	Negative	
Healthcare professional-collected vaginal swab for HPV tes	Yes	Negative	
Self-collecting vaginal swab for HPV testing	Yes	Negative	
Self-collecting vaginal swab for HPV testing	Yes	Negative	
Self-collecting vaginal swab for HPV testing	Yes	Negative	
Self-collecting vaginal swab for HPV testing	Yes	Negative	
Self-collecting vaginal swab for HPV testing	Yes	Negative	
Self-collecting vaginal swab for HPV testing	Yes	Negative	
Self-collecting vaginal swab for HPV testing	Yes	Negative	

Self-collecting vaginal swab for HPV testing	Yes	Invalid	
No preference	Yes	Negative	
Self-collecting vaginal swab for HPV testing	Yes	Negative	
Self-collecting vaginal swab for HPV testing	Yes	Negative	
Self-collecting vaginal swab for HPV testing	Yes	Negative	
No preference	Yes	Negative	
Self-collecting vaginal swab for HPV testing	Yes	Negative	
Self-collecting vaginal swab for HPV testing	Yes	Negative	
Self-collecting vaginal swab for HPV testing	Yes	Negative	
Self-collecting vaginal swab for HPV testing	Yes	Negative	
Self-collecting vaginal swab for HPV testing	Yes	Negative	
Self-collecting vaginal swab for HPV testing	Yes	Negative	
Self-collecting vaginal swab for HPV testing	Yes	Negative	
Self-collecting vaginal swab for HPV testing	Yes	Negative	
Self-collecting vaginal swab for HPV testing	Yes	Negative	
Self-collecting vaginal swab for HPV testing	Yes	Negative	
Self-collecting vaginal swab for HPV testing	Yes	Negative	
Self-collecting vaginal swab for HPV testing	Yes	Negative	
Self-collecting vaginal swab for HPV testing	Yes	Negative	
Self-collecting vaginal swab for HPV testing	Yes	Negative	
Self-collecting vaginal swab for HPV testing	Yes	Negative	
Self-collecting vaginal swab for HPV testing	Yes	Positive Non-HPV 16/18	2/6/2023
Self-collecting vaginal swab for HPV testing	Yes	Negative	
Self-collecting vaginal swab for HPV testing	Yes	Negative	
Self-collecting vaginal swab for HPV testing	Yes	Negative	
Self-collecting vaginal swab for HPV testing	Yes	Negative	
Self-collecting vaginal swab for HPV testing	Yes	Negative	
Self-collecting vaginal swab for HPV testing	Yes	Negative	
No preference	Yes	Negative	
Self-collecting vaginal swab for HPV testing	Yes	Negative	
Self-collecting vaginal swab for HPV testing	Yes	Negative	
Self-collecting vaginal swab for HPV testing	Yes	Negative	
Self-collecting vaginal swab for HPV testing	Yes	Negative	

Self-collecting vaginal swab for HPV testing	Yes	Negative	
Self-collecting vaginal swab for HPV testing	Yes	Negative	
Self-collecting vaginal swab for HPV testing	Yes	Negative	
Self-collecting vaginal swab for HPV testing	Yes	Negative	
Self-collecting vaginal swab for HPV testing	Yes	Negative	
Self-collecting vaginal swab for HPV testing	Yes	Negative	
Self-collecting vaginal swab for HPV testing	Yes	Negative	
Self-collecting vaginal swab for HPV testing	Yes	Negative	
Self-collecting vaginal swab for HPV testing	Yes	Negative	
Self-collecting vaginal swab for HPV testing	Yes	Negative	
Self-collecting vaginal swab for HPV testing	Yes	Negative	
Self-collecting vaginal swab for HPV testing	Yes	Positive Non-HPV 16/18	15/3/2023
Self-collecting vaginal swab for HPV testing	Yes	Negative	
Self-collecting vaginal swab for HPV testing	Yes	Negative	
Self-collecting vaginal swab for HPV testing	Yes	Negative	
Self-collecting vaginal swab for HPV testing	Yes	Negative	
Self-collecting vaginal swab for HPV testing	Yes	Negative	
Self-collecting vaginal swab for HPV testing	Yes	Negative	
Self-collecting vaginal swab for HPV testing	Yes	Negative	
Self-collecting vaginal swab for HPV testing	Yes	Negative	
Self-collecting vaginal swab for HPV testing	Yes	Negative	
Self-collecting vaginal swab for HPV testing	Yes	Negative	
Self-collecting vaginal swab for HPV testing	Yes	Negative	
Self-collecting vaginal swab for HPV testing	Yes	Negative	
Self-collecting vaginal swab for HPV testing	Yes	Negative	
Self-collecting vaginal swab for HPV testing	Yes	Positive Non-HPV 16/18	17/3/2023
Self-collecting vaginal swab for HPV testing	Yes	Positive Non-HPV 16/18	14/3/2023
Self-collecting vaginal swab for HPV testing	Yes	Invalid	
Self-collecting vaginal swab for HPV testing	Yes	Negative	
Self-collecting vaginal swab for HPV testing	Yes	Negative	
Self-collecting vaginal swab for HPV testing	Yes	Negative	
Healthcare professional-collected vaginal swab for HPV tes	Yes	Negative	
Self-collecting vaginal swab for HPV testing	Yes	Negative	

Healthcare professional-collected vaginal swab for HPV tes	Yes	Negative	
Self-collecting vaginal swab for HPV testing	Yes	Negative	
Self-collecting vaginal swab for HPV testing	Yes	Negative	
Self-collecting vaginal swab for HPV testing	Yes	Negative	
Self-collecting vaginal swab for HPV testing	Yes	Positive HPV 16 & Non-HPV 16/18	10/10/2023
Self-collecting vaginal swab for HPV testing	Yes	Negative	
Self-collecting vaginal swab for HPV testing	Yes	Negative	
No preference	Yes	Negative	
Self-collecting vaginal swab for HPV testing	Yes	Negative	
Self-collecting vaginal swab for HPV testing	Yes	Negative	
Self-collecting vaginal swab for HPV testing	Yes	Negative	
Self-collecting vaginal swab for HPV testing	Yes	Negative	
Self-collecting vaginal swab for HPV testing	Yes	Negative	
Self-collecting vaginal swab for HPV testing	Yes	Negative	
Self-collecting vaginal swab for HPV testing	Yes	Invalid	
Self-collecting vaginal swab for HPV testing	Yes	Negative	
Self-collecting vaginal swab for HPV testing	Yes	Negative	
Self-collecting vaginal swab for HPV testing	Yes	Negative	
Self-collecting vaginal swab for HPV testing	Yes	Negative	
No preference	Yes	Negative	
Self-collecting vaginal swab for HPV testing	Yes	Negative	
Self-collecting vaginal swab for HPV testing	Yes	Positive Non-HPV 16/18	23/5/2023
Self-collecting vaginal swab for HPV testing	Yes	Negative	
Self-collecting vaginal swab for HPV testing	Yes	Negative	
Self-collecting vaginal swab for HPV testing	Yes	Negative	
Self-collecting vaginal swab for HPV testing	Yes	Negative	
Self-collecting vaginal swab for HPV testing	Yes	Invalid	
Self-collecting vaginal swab for HPV testing	Yes	Negative	
Self-collecting vaginal swab for HPV testing	Yes	Negative	
Self-collecting vaginal swab for HPV testing	Yes	Negative	
Self-collecting vaginal swab for HPV testing	Yes	Invalid	
Self-collecting vaginal swab for HPV testing	Yes	Negative	
Healthcare professional-collected vaginal swab for HPV tes	Yes	Negative	

Self-collecting vaginal swab for HPV testing	Yes	Negative	
No preference	Yes	Negative	
Self-collecting vaginal swab for HPV testing	Yes	Negative	
Self-collecting vaginal swab for HPV testing	Yes	Negative	
Self-collecting vaginal swab for HPV testing	Yes	Negative	
Self-collecting vaginal swab for HPV testing	Yes	Negative	
Self-collecting vaginal swab for HPV testing	Yes	Negative	
Self-collecting vaginal swab for HPV testing	Yes	Negative	
Self-collecting vaginal swab for HPV testing	Yes	Negative	
Self-collecting vaginal swab for HPV testing	Yes	Positive HPV 16	
Self-collecting vaginal swab for HPV testing	Yes	Negative	
Self-collecting vaginal swab for HPV testing	Yes	Negative	
Healthcare professional-collected vaginal swab for HPV tes	Yes	Negative	
Self-collecting vaginal swab for HPV testing	Yes	Negative	
Self-collecting vaginal swab for HPV testing	Yes	Negative	
Self-collecting vaginal swab for HPV testing	Yes	Positive Non-HPV 16/18	22/3/2023
Healthcare professional-collected vaginal swab for HPV tes	Yes	Negative	
Self-collecting vaginal swab for HPV testing	Yes	Negative	
Self-collecting vaginal swab for HPV testing	Yes	Negative	
No preference	Yes	Negative	
Self-collecting vaginal swab for HPV testing	Yes	Negative	
Self-collecting vaginal swab for HPV testing	Yes	Negative	
Self-collecting vaginal swab for HPV testing	Yes	Negative	
Self-collecting vaginal swab for HPV testing	Yes	Negative	
Self-collecting vaginal swab for HPV testing	Yes	Negative	
Self-collecting vaginal swab for HPV testing	Yes	Negative	
Self-collecting vaginal swab for HPV testing	Yes	Negative	
Self-collecting vaginal swab for HPV testing	Yes	Negative	
Healthcare professional-collected vaginal swab for HPV tes	Yes	Negative	
Self-collecting vaginal swab for HPV testing	Yes	Negative	
Self-collecting vaginal swab for HPV testing	Yes	Negative	
Self-collecting vaginal swab for HPV testing	Yes	Negative	
Self-collecting vaginal swab for HPV testing	Yes	Negative	

Self-collecting vaginal swab for HPV testing	Yes	Negative	
Healthcare professional-collected vaginal swab for HPV tes	Yes	Negative	
Self-collecting vaginal swab for HPV testing	Yes	Negative	
Self-collecting vaginal swab for HPV testing	Yes	Negative	
Self-collecting vaginal swab for HPV testing	Yes	Negative	
Self-collecting vaginal swab for HPV testing	Yes	Negative	
Self-collecting vaginal swab for HPV testing	Yes	Negative	
Self-collecting vaginal swab for HPV testing	Yes	Negative	
		Negative	
Healthcare professional-collected vaginal swab for HPV tes	Yes	Negative	
Self-collecting vaginal swab for HPV testing	Yes	Negative	
Self-collecting vaginal swab for HPV testing	Yes	Negative	
Self-collecting vaginal swab for HPV testing	Yes	Negative	
		Negative	
Self-collecting vaginal swab for HPV testing	Yes	Negative	
Healthcare professional-collected vaginal swab for HPV tes	Yes	Positive Non-HPV 16/18	2/5/2023
Self-collecting vaginal swab for HPV testing	Yes	Negative	
No preference	Yes	Invalid	
Self-collecting vaginal swab for HPV testing	Yes	Negative	
Self-collecting vaginal swab for HPV testing	Yes	Negative	
Healthcare professional-collected vaginal swab for HPV tes	Yes	Negative	
Self-collecting vaginal swab for HPV testing	Yes	Negative	
No preference	Yes	Negative	
Self-collecting vaginal swab for HPV testing	Yes	Negative	
Self-collecting vaginal swab for HPV testing	Yes	Negative	
Self-collecting vaginal swab for HPV testing	Yes	Negative	
Self-collecting vaginal swab for HPV testing	Yes	Negative	
Self-collecting vaginal swab for HPV testing	Yes	Negative	
Self-collecting vaginal swab for HPV testing	Yes	Negative	
Self-collecting vaginal swab for HPV testing	Yes	Negative	
Self-collecting vaginal swab for HPV testing	Yes	Negative	
Self-collecting vaginal swab for HPV testing	Yes	Negative	
Self-collecting vaginal swab for HPV testing	Yes	Invalid	

Self-collecting vaginal swab for HPV testing	Yes	Negative	
Self-collecting vaginal swab for HPV testing	Yes	Positive HPV 18	28/3/2023
Self-collecting vaginal swab for HPV testing	Yes	Negative	
Self-collecting vaginal swab for HPV testing	Yes	Positive Non-HPV 16/18	30/5/2023
Self-collecting vaginal swab for HPV testing	Yes	Negative	
Healthcare professional-collected vaginal swab for HPV tes	Yes	Negative	
Self-collecting vaginal swab for HPV testing	Yes	Negative	
Self-collecting vaginal swab for HPV testing	Yes	Negative	
Self-collecting vaginal swab for HPV testing	Yes	Negative	
Self-collecting vaginal swab for HPV testing	Yes	Negative	
Self-collecting vaginal swab for HPV testing	Yes	Negative	
No preference	Yes	Negative	
Self-collecting vaginal swab for HPV testing	Yes	Negative	
Self-collecting vaginal swab for HPV testing	Yes	Negative	
Self-collecting vaginal swab for HPV testing	Yes	Positive Non-HPV 16/18	
Self-collecting vaginal swab for HPV testing	Yes	Invalid	
Self-collecting vaginal swab for HPV testing	Yes	Negative	
Self-collecting vaginal swab for HPV testing	Yes	Negative	
Self-collecting vaginal swab for HPV testing	Yes	Negative	
Self-collecting vaginal swab for HPV testing	Yes	Negative	
Self-collecting vaginal swab for HPV testing	Yes	Negative	
Self-collecting vaginal swab for HPV testing	Yes	Negative	
Pap smear - physician conducting a speculum examination	Yes	Negative	
No preference	Yes	Negative	
Self-collecting vaginal swab for HPV testing	Yes	Negative	
Self-collecting vaginal swab for HPV testing	Yes	Negative	
Self-collecting vaginal swab for HPV testing	Yes	Negative	
Healthcare professional-collected vaginal swab for HPV tes	Yes	Negative	
Self-collecting vaginal swab for HPV testing	Yes	Negative	
Self-collecting vaginal swab for HPV testing	Yes	Negative	
Self-collecting vaginal swab for HPV testing	Yes	Negative	
Self-collecting vaginal swab for HPV testing	Yes	Negative	
Self-collecting vaginal swab for HPV testing	Yes	Positive Non-HPV 16/18	7/6/2023

Self-collecting vaginal swab for HPV testing	Yes	Positive HPV 16	9/8/2023
Self-collecting vaginal swab for HPV testing	Yes	Negative	
Healthcare professional-collected vaginal swab for HPV tes	Yes	Negative	
Self-collecting vaginal swab for HPV testing	Yes	Negative	
No preference	Yes	Negative	
No preference	Yes	Negative	
Healthcare professional-collected vaginal swab for HPV tes	Yes	Negative	
Self-collecting vaginal swab for HPV testing	Yes	Negative	
Self-collecting vaginal swab for HPV testing	Yes	Negative	
Self-collecting vaginal swab for HPV testing	Yes	Negative	
Self-collecting vaginal swab for HPV testing	Yes	Negative	
Self-collecting vaginal swab for HPV testing	Yes	Negative	
Self-collecting vaginal swab for HPV testing	Yes	Negative	
Self-collecting vaginal swab for HPV testing	Yes	Negative	
Self-collecting vaginal swab for HPV testing	Yes	Negative	
Self-collecting vaginal swab for HPV testing	Yes	Negative	
Self-collecting vaginal swab for HPV testing	Yes	Negative	
Self-collecting vaginal swab for HPV testing	Yes	Negative	
Self-collecting vaginal swab for HPV testing	Yes	Negative	
Pap smear - physician conducting a speculum examination	Yes	Negative	
Self-collecting vaginal swab for HPV testing	Yes	Negative	
Self-collecting vaginal swab for HPV testing	Yes	Negative	
Self-collecting vaginal swab for HPV testing	Yes	Negative	
Self-collecting vaginal swab for HPV testing	Yes	Negative	
Self-collecting vaginal swab for HPV testing	Yes	Negative	
No preference	Yes	Negative	
Healthcare professional-collected vaginal swab for HPV tes	Yes	Negative	
Self-collecting vaginal swab for HPV testing	Yes	Negative	
Self-collecting vaginal swab for HPV testing	Yes	Negative	
Self-collecting vaginal swab for HPV testing	Yes	Negative	
Self-collecting vaginal swab for HPV testing	Yes	Negative	
No preference	Yes	Negative	
Self-collecting vaginal swab for HPV testing	Yes	Positive Non-HPV 16/18	

Healthcare professional-collected vaginal swab for HPV tes	Yes	Negative
Self-collecting vaginal swab for HPV testing	Yes	Negative
Self-collecting vaginal swab for HPV testing	Yes	Positive HPV 16
Self-collecting vaginal swab for HPV testing	Yes	Negative
Self-collecting vaginal swab for HPV testing	Yes	Negative
Self-collecting vaginal swab for HPV testing	Yes	Negative
Self-collecting vaginal swab for HPV testing	Yes	Negative
Self-collecting vaginal swab for HPV testing	Yes	Negative
Healthcare professional-collected vaginal swab for HPV tes	Yes	Negative
Self-collecting vaginal swab for HPV testing	Yes	Invalid
Self-collecting vaginal swab for HPV testing	Yes	Negative
No preference	Yes	Negative
Self-collecting vaginal swab for HPV testing	Yes	Negative
Self-collecting vaginal swab for HPV testing	Yes	Invalid
Self-collecting vaginal swab for HPV testing	Yes	Negative
Self-collecting vaginal swab for HPV testing	Yes	Negative
Self-collecting vaginal swab for HPV testing	Yes	Negative
Self-collecting vaginal swab for HPV testing	Yes	Negative
Self-collecting vaginal swab for HPV testing	Yes	Negative
No preference	Yes	Negative
Self-collecting vaginal swab for HPV testing	Yes	Negative
Self-collecting vaginal swab for HPV testing	Yes	Negative
Self-collecting vaginal swab for HPV testing	Yes	Negative
Self-collecting vaginal swab for HPV testing	Yes	Negative
Self-collecting vaginal swab for HPV testing	Yes	Negative
Self-collecting vaginal swab for HPV testing	Yes	Negative
Self-collecting vaginal swab for HPV testing	Yes	Negative
Self-collecting vaginal swab for HPV testing	Yes	Negative
Healthcare professional-collected vaginal swab for HPV tes	Yes	Negative
Self-collecting vaginal swab for HPV testing	Yes	Negative
Self-collecting vaginal swab for HPV testing	Yes	Negative
Self-collecting vaginal swab for HPV testing	Yes	Negative
Self-collecting vaginal swab for HPV testing	Yes	Negative

Self-collecting vaginal swab for HPV testing	Yes	Negative
Self-collecting vaginal swab for HPV testing	Yes	Negative
No preference	Yes	Negative
Self-collecting vaginal swab for HPV testing	Yes	Negative
Self-collecting vaginal swab for HPV testing	Yes	Negative
Self-collecting vaginal swab for HPV testing	Yes	Negative
Healthcare professional-collected vaginal swab for HPV tes	Yes	Negative
Self-collecting vaginal swab for HPV testing	Yes	Negative
Self-collecting vaginal swab for HPV testing	Yes	Negative
Self-collecting vaginal swab for HPV testing	Yes	Negative
Self-collecting vaginal swab for HPV testing	Yes	Negative
Self-collecting vaginal swab for HPV testing	Yes	Negative
Self-collecting vaginal swab for HPV testing	Yes	Negative
Self-collecting vaginal swab for HPV testing	Yes	Negative
Self-collecting vaginal swab for HPV testing	Yes	Negative
Self-collecting vaginal swab for HPV testing	Yes	Negative
Healthcare professional-collected vaginal swab for HPV tes	Yes	Negative
Self-collecting vaginal swab for HPV testing	Yes	Negative
Self-collecting vaginal swab for HPV testing	Yes	Negative
No preference	Yes	Negative
Self-collecting vaginal swab for HPV testing	Yes	Negative
Healthcare professional-collected vaginal swab for HPV tes	Yes	Negative
Self-collecting vaginal swab for HPV testing	Yes	Negative
Self-collecting vaginal swab for HPV testing	Yes	Negative
Self-collecting vaginal swab for HPV testing	Yes	Negative
Self-collecting vaginal swab for HPV testing	Yes	Negative
Self-collecting vaginal swab for HPV testing	Yes	Negative
Healthcare professional-collected vaginal swab for HPV tes	Yes	Negative
Self-collecting vaginal swab for HPV testing	Yes	Negative
Self-collecting vaginal swab for HPV testing	Yes	Negative
Self-collecting vaginal swab for HPV testing	Yes	Negative
Self-collecting vaginal swab for HPV testing	Yes	Negative
Self-collecting vaginal swab for HPV testing	Yes	Negative

Self-collecting vaginal swab for HPV testing	Yes	Negative	
Self-collecting vaginal swab for HPV testing	Yes	Negative	
Self-collecting vaginal swab for HPV testing	Yes	Negative	
Self-collecting vaginal swab for HPV testing	Yes	Negative	
Self-collecting vaginal swab for HPV testing	Yes	Negative	
Self-collecting vaginal swab for HPV testing	Yes	Negative	
Self-collecting vaginal swab for HPV testing	Yes	Negative	
Self-collecting vaginal swab for HPV testing	Yes	Negative	
Self-collecting vaginal swab for HPV testing	Yes	Negative	
Self-collecting vaginal swab for HPV testing	Yes	Positive HPV 16 & Non-HPV 16/18	4/7/2023
Self-collecting vaginal swab for HPV testing	Yes	Negative	
Self-collecting vaginal swab for HPV testing	Yes	Negative	
Self-collecting vaginal swab for HPV testing	Yes	Negative	
Self-collecting vaginal swab for HPV testing	Yes	Negative	
Self-collecting vaginal swab for HPV testing	Yes	Negative	
Self-collecting vaginal swab for HPV testing	Yes	Negative	
Self-collecting vaginal swab for HPV testing	Yes	Negative	
Self-collecting vaginal swab for HPV testing	Yes	Negative	
Self-collecting vaginal swab for HPV testing	Yes	Negative	
Self-collecting vaginal swab for HPV testing	Yes	Negative	
Self-collecting vaginal swab for HPV testing	Yes	Negative	
Self-collecting vaginal swab for HPV testing	Yes	Negative	
Self-collecting vaginal swab for HPV testing	Yes	Negative	
Self-collecting vaginal swab for HPV testing	Yes	Negative	
Self-collecting vaginal swab for HPV testing	Yes	Negative	
Healthcare professional-collected vaginal swab for HPV tes	Yes	Negative	
Self-collecting vaginal swab for HPV testing	Yes	Negative	
Self-collecting vaginal swab for HPV testing	Yes	Negative	
Self-collecting vaginal swab for HPV testing	Yes	Negative	
Self-collecting vaginal swab for HPV testing	Yes	Positive HPV 16	
Self-collecting vaginal swab for HPV testing	Yes	Negative	
Self-collecting vaginal swab for HPV testing	Yes	Negative	
Self-collecting vaginal swab for HPV testing	Yes	Positive Non-HPV 16/18	30/7/2024

Healthcare professional-collected vaginal swab for HPV tes	Yes	Negative	
Self-collecting vaginal swab for HPV testing	Yes	Negative	
Self-collecting vaginal swab for HPV testing	Yes	Negative	
Self-collecting vaginal swab for HPV testing	Yes	Negative	
Self-collecting vaginal swab for HPV testing	Yes	Negative	
Self-collecting vaginal swab for HPV testing	Yes	Negative	
Self-collecting vaginal swab for HPV testing	Yes	Negative	
Self-collecting vaginal swab for HPV testing	Yes	Negative	
Self-collecting vaginal swab for HPV testing	Yes	Negative	
No preference	Yes	Negative	
Self-collecting vaginal swab for HPV testing	Yes	Positive HPV 18	
Self-collecting vaginal swab for HPV testing	Yes	Negative	
Self-collecting vaginal swab for HPV testing	Yes	Negative	
Self-collecting vaginal swab for HPV testing	Yes	Invalid	
Self-collecting vaginal swab for HPV testing	Yes	Negative	
No preference	Yes	Invalid	
Self-collecting vaginal swab for HPV testing	Yes	Negative	
Self-collecting vaginal swab for HPV testing	Yes	Negative	
No preference	Yes	Negative	
No preference	Yes	Negative	
Self-collecting vaginal swab for HPV testing	Yes	Negative	
Self-collecting vaginal swab for HPV testing	Yes	Negative	
Self-collecting vaginal swab for HPV testing	Yes	Negative	
Self-collecting vaginal swab for HPV testing	Yes	Negative	
Healthcare professional-collected vaginal swab for HPV tes	Yes	Negative	
Self-collecting vaginal swab for HPV testing	Yes	Negative	
Self-collecting vaginal swab for HPV testing	Yes	Positive Non-HPV 16/18	14/6/2023
Self-collecting vaginal swab for HPV testing	Yes	Negative	
No preference	Yes	Negative	
Self-collecting vaginal swab for HPV testing	Yes	Negative	
No preference	Yes	Negative	
Self-collecting vaginal swab for HPV testing	Yes	Negative	
Self-collecting vaginal swab for HPV testing	Yes	Negative	

Healthcare professional-collected vaginal swab for HPV tes	Yes	Negative
Self-collecting vaginal swab for HPV testing	Yes	Negative
Self-collecting vaginal swab for HPV testing	Yes	Negative
Self-collecting vaginal swab for HPV testing	Yes	Negative
Self-collecting vaginal swab for HPV testing	Yes	Negative
Self-collecting vaginal swab for HPV testing	Yes	Negative
Self-collecting vaginal swab for HPV testing	Yes	Negative
Self-collecting vaginal swab for HPV testing	Yes	Negative
Self-collecting vaginal swab for HPV testing	Yes	Negative
Self-collecting vaginal swab for HPV testing	Yes	Negative
Self-collecting vaginal swab for HPV testing	Yes	Negative
Self-collecting vaginal swab for HPV testing	Yes	Negative
Self-collecting vaginal swab for HPV testing	Yes	Negative
Self-collecting vaginal swab for HPV testing	Yes	Negative
Self-collecting vaginal swab for HPV testing	Yes	Negative
Self-collecting vaginal swab for HPV testing	Yes	Negative
Self-collecting vaginal swab for HPV testing	Yes	Negative
Self-collecting vaginal swab for HPV testing	Yes	Negative
Self-collecting vaginal swab for HPV testing	Yes	Negative
Self-collecting vaginal swab for HPV testing	Yes	Negative
Self-collecting vaginal swab for HPV testing	Yes	Negative
Self-collecting vaginal swab for HPV testing	Yes	Negative
Self-collecting vaginal swab for HPV testing	Yes	Negative
Self-collecting vaginal swab for HPV testing	Yes	Negative
Self-collecting vaginal swab for HPV testing	Yes	Negative
Self-collecting vaginal swab for HPV testing	Yes	Negative
Self-collecting vaginal swab for HPV testing	Yes	Negative
Self-collecting vaginal swab for HPV testing	Yes	Negative
Self-collecting vaginal swab for HPV testing	Yes	Negative
Self-collecting vaginal swab for HPV testing	Yes	Negative
Self-collecting vaginal swab for HPV testing	Yes	Negative
Self-collecting vaginal swab for HPV testing	Yes	Negative
Self-collecting vaginal swab for HPV testing	Yes	Negative

Self-collecting vaginal swab for HPV testing	Yes	Negative	
No preference	Yes	Negative	
Self-collecting vaginal swab for HPV testing	Yes	Negative	
Self-collecting vaginal swab for HPV testing	Yes	Negative	
No preference	Yes	Negative	
Self-collecting vaginal swab for HPV testing	Yes	Negative	
Healthcare professional-collected vaginal swab for HPV tes	Yes	Negative	
Healthcare professional-collected vaginal swab for HPV tes	Yes	Invalid	
Healthcare professional-collected vaginal swab for HPV tes	No	Negative	
Healthcare professional-collected vaginal swab for HPV tes	Yes	Negative	
Healthcare professional-collected vaginal swab for HPV tes	Yes	Positive Non-HPV 16/18	8/8/2023
Self-collecting vaginal swab for HPV testing	Yes	Negative	
Self-collecting vaginal swab for HPV testing	Yes	Negative	
Self-collecting vaginal swab for HPV testing	Yes	Negative	
Self-collecting vaginal swab for HPV testing	Yes	Negative	
Self-collecting vaginal swab for HPV testing	Yes	Negative	
Self-collecting vaginal swab for HPV testing	Yes	Negative	
Self-collecting vaginal swab for HPV testing	Yes	Negative	
Self-collecting vaginal swab for HPV testing	Yes	Negative	
Self-collecting vaginal swab for HPV testing	Yes	Invalid	
Self-collecting vaginal swab for HPV testing	Yes	Negative	
Self-collecting vaginal swab for HPV testing	Yes	Negative	
Self-collecting vaginal swab for HPV testing	Yes	Negative	
Self-collecting vaginal swab for HPV testing	Yes	Negative	
Self-collecting vaginal swab for HPV testing	Yes	Negative	
Self-collecting vaginal swab for HPV testing	Yes	Negative	
No preference	Yes	Negative	
No preference	Yes	Negative	
Self-collecting vaginal swab for HPV testing	Yes	Negative	
Self-collecting vaginal swab for HPV testing	Yes	Negative	
Self-collecting vaginal swab for HPV testing	Yes	Negative	
		Negative	
Self-collecting vaginal swab for HPV testing	Yes	Negative	

Healthcare professional-collected vaginal swab for HPV tes	Yes	Negative	
Self-collecting vaginal swab for HPV testing	Yes	Negative	
Healthcare professional-collected vaginal swab for HPV tes	Yes	Negative	
Self-collecting vaginal swab for HPV testing	Yes	Negative	
Self-collecting vaginal swab for HPV testing	Yes	Negative	
Self-collecting vaginal swab for HPV testing	Yes	Negative	
Self-collecting vaginal swab for HPV testing	Yes	Negative	
Self-collecting vaginal swab for HPV testing	Yes	Negative	
Self-collecting vaginal swab for HPV testing	Yes	Negative	
Self-collecting vaginal swab for HPV testing	Yes	Negative	
Healthcare professional-collected vaginal swab for HPV tes	Yes	Negative	
Self-collecting vaginal swab for HPV testing	Yes	Negative	
Self-collecting vaginal swab for HPV testing	Yes	Negative	
Self-collecting vaginal swab for HPV testing	Yes	Negative	
No preference	Yes	Negative	
		Negative	
Self-collecting vaginal swab for HPV testing	Yes	Negative	
Self-collecting vaginal swab for HPV testing	Yes	Negative	
Self-collecting vaginal swab for HPV testing	Yes	Negative	
Self-collecting vaginal swab for HPV testing	Yes	Negative	
Self-collecting vaginal swab for HPV testing	Yes	Negative	
Healthcare professional-collected vaginal swab for HPV tes	Yes	Negative	
Self-collecting vaginal swab for HPV testing	Yes	Negative	
Self-collecting vaginal swab for HPV testing	Yes	Negative	
No preference	Yes	Positive Non-HPV 16/18	25/10/2023
Self-collecting vaginal swab for HPV testing	Yes	Positive Non-HPV 16/18	
Self-collecting vaginal swab for HPV testing	Yes	Negative	
No preference	Yes	Negative	
Self-collecting vaginal swab for HPV testing	Yes	Negative	
Self-collecting vaginal swab for HPV testing	Yes	Negative	
Self-collecting vaginal swab for HPV testing	Yes	Negative	
Self-collecting vaginal swab for HPV testing	Yes	Negative	
Self-collecting vaginal swab for HPV testing	Yes	Negative	

Self-collecting vaginal swab for HPV testing	Yes	Negative	
Self-collecting vaginal swab for HPV testing	Yes	Negative	
Self-collecting vaginal swab for HPV testing	Yes	Negative	
Self-collecting vaginal swab for HPV testing	Yes	Negative	
Self-collecting vaginal swab for HPV testing	Yes	Negative	
Self-collecting vaginal swab for HPV testing	Yes	Negative	
Self-collecting vaginal swab for HPV testing	Yes	Negative	
Self-collecting vaginal swab for HPV testing	Yes	Negative	
Self-collecting vaginal swab for HPV testing	Yes	Negative	
Self-collecting vaginal swab for HPV testing	Yes	Negative	
Self-collecting vaginal swab for HPV testing	Yes	Negative	
Self-collecting vaginal swab for HPV testing	Yes	Invalid	
Self-collecting vaginal swab for HPV testing	Yes	Negative	
Self-collecting vaginal swab for HPV testing	Yes	Negative	
Self-collecting vaginal swab for HPV testing	Yes	Negative	
No preference	Yes	Negative	
Self-collecting vaginal swab for HPV testing	Yes	Negative	
Healthcare professional-collected vaginal swab for HPV tes	Yes	Negative	
Self-collecting vaginal swab for HPV testing	Yes	Negative	
Self-collecting vaginal swab for HPV testing	Yes	Negative	
Self-collecting vaginal swab for HPV testing	Yes	Negative	
Self-collecting vaginal swab for HPV testing	Yes	Negative	
Self-collecting vaginal swab for HPV testing	Yes	Negative	
Self-collecting vaginal swab for HPV testing	Yes	Negative	
No preference	Yes	Positive Non-HPV 16/18	10/10/2023
Self-collecting vaginal swab for HPV testing	Yes	Negative	
Self-collecting vaginal swab for HPV testing	Yes	Negative	
Self-collecting vaginal swab for HPV testing	Yes	Positive HPV 18	27/9/2023
Self-collecting vaginal swab for HPV testing	Yes	Negative	
Self-collecting vaginal swab for HPV testing	Yes	Negative	
Self-collecting vaginal swab for HPV testing	Yes	Negative	
Self-collecting vaginal swab for HPV testing	Yes	Negative	
Self-collecting vaginal swab for HPV testing	Yes	Invalid	

Healthcare professional-collected vaginal swab for HPV tes	Yes	Negative	
Self-collecting vaginal swab for HPV testing	Yes	Negative	
Healthcare professional-collected vaginal swab for HPV tes	No	Negative	
Self-collecting vaginal swab for HPV testing	Yes	Negative	
Healthcare professional-collected vaginal swab for HPV tes	Yes	Negative	
Self-collecting vaginal swab for HPV testing	Yes	Negative	
Self-collecting vaginal swab for HPV testing	Yes	Negative	
Self-collecting vaginal swab for HPV testing	Yes	Negative	
Self-collecting vaginal swab for HPV testing	Yes	Negative	
Self-collecting vaginal swab for HPV testing	Yes	Negative	
Self-collecting vaginal swab for HPV testing	Yes	Negative	
Healthcare professional-collected vaginal swab for HPV tes	Yes	Positive HPV 18 & Non-HPV 16/18	5/7/2023
Self-collecting vaginal swab for HPV testing	Yes	Negative	
Self-collecting vaginal swab for HPV testing	Yes	Negative	
Self-collecting vaginal swab for HPV testing	Yes	Invalid	
Self-collecting vaginal swab for HPV testing	Yes	Negative	
Self-collecting vaginal swab for HPV testing	Yes	Negative	
No preference	Yes	Negative	
Self-collecting vaginal swab for HPV testing	Yes	Negative	
Self-collecting vaginal swab for HPV testing	Yes	Negative	
Self-collecting vaginal swab for HPV testing	Yes	Negative	
Self-collecting vaginal swab for HPV testing	Yes	Negative	
Self-collecting vaginal swab for HPV testing	Yes	Negative	
Self-collecting vaginal swab for HPV testing	Yes	Negative	
Self-collecting vaginal swab for HPV testing	Yes	Negative	
Self-collecting vaginal swab for HPV testing	Yes	Negative	
Self-collecting vaginal swab for HPV testing	Yes	Negative	
Self-collecting vaginal swab for HPV testing	Yes	Negative	
Self-collecting vaginal swab for HPV testing	Yes	Negative	
No preference	Yes	Negative	
Self-collecting vaginal swab for HPV testing	Yes	Negative	
Self-collecting vaginal swab for HPV testing	Yes	Negative	
Self-collecting vaginal swab for HPV testing	Yes	Negative	

Self-collecting vaginal swab for HPV testing	Yes	Negative	
Pap smear - physician conducting a speculum examination	No	Negative	
Self-collecting vaginal swab for HPV testing	Yes	Negative	
Self-collecting vaginal swab for HPV testing	Yes	Negative	
Self-collecting vaginal swab for HPV testing	Yes	Negative	
Self-collecting vaginal swab for HPV testing	Yes	Negative	
Self-collecting vaginal swab for HPV testing	Yes	Negative	
Self-collecting vaginal swab for HPV testing	Yes	Negative	
Self-collecting vaginal swab for HPV testing	Yes	Negative	
Self-collecting vaginal swab for HPV testing	Yes	Negative	
Healthcare professional-collected vaginal swab for HPV tes	Yes	Invalid	
Self-collecting vaginal swab for HPV testing	Yes	Negative	
Self-collecting vaginal swab for HPV testing	Yes	Negative	
Self-collecting vaginal swab for HPV testing	Yes	Negative	
Self-collecting vaginal swab for HPV testing	Yes	Negative	
Self-collecting vaginal swab for HPV testing	Yes	Negative	
Self-collecting vaginal swab for HPV testing	Yes	Negative	
Self-collecting vaginal swab for HPV testing	Yes	Negative	
Self-collecting vaginal swab for HPV testing	Yes	Negative	
Self-collecting vaginal swab for HPV testing	Yes	Negative	
Self-collecting vaginal swab for HPV testing	Yes	Negative	
Self-collecting vaginal swab for HPV testing	Yes	Negative	
Self-collecting vaginal swab for HPV testing	Yes	Negative	
Self-collecting vaginal swab for HPV testing	Yes	Negative	
Self-collecting vaginal swab for HPV testing	Yes	Negative	
Self-collecting vaginal swab for HPV testing	Yes	Invalid	
Self-collecting vaginal swab for HPV testing	Yes	Negative	
Self-collecting vaginal swab for HPV testing	Yes	Negative	
Self-collecting vaginal swab for HPV testing	Yes	Negative	
Self-collecting vaginal swab for HPV testing	Yes	Positive Non-HPV 16/18	27/6/2023
Self-collecting vaginal swab for HPV testing	Yes	Negative	
Self-collecting vaginal swab for HPV testing	Yes	Negative	
Self-collecting vaginal swab for HPV testing	Yes	Negative	

Self-collecting vaginal swab for HPV testing	Yes	Negative	
Self-collecting vaginal swab for HPV testing	Yes	Positive HPV 16	11/10/2023
Healthcare professional-collected vaginal swab for HPV tes	Yes	Negative	
Self-collecting vaginal swab for HPV testing	Yes	Negative	
No preference	Yes	Negative	
Self-collecting vaginal swab for HPV testing	Yes	Negative	
Self-collecting vaginal swab for HPV testing	Yes	Positive Non-HPV 16/18	30/8/2023
Self-collecting vaginal swab for HPV testing	Yes	Negative	
Self-collecting vaginal swab for HPV testing	Yes	Positive Non-HPV 16/18	7/11/2023
Self-collecting vaginal swab for HPV testing	Yes	Negative	
Self-collecting vaginal swab for HPV testing	Yes	Negative	
Self-collecting vaginal swab for HPV testing	Yes	Negative	
Self-collecting vaginal swab for HPV testing	Yes	Positive Non-HPV 16/18	20/6/2023
Self-collecting vaginal swab for HPV testing	Yes	Negative	
Self-collecting vaginal swab for HPV testing	Yes	Negative	
Self-collecting vaginal swab for HPV testing	Yes	Negative	
Healthcare professional-collected vaginal swab for HPV tes	Yes	Negative	
Self-collecting vaginal swab for HPV testing	Yes	Negative	
Self-collecting vaginal swab for HPV testing	Yes	Negative	
Self-collecting vaginal swab for HPV testing	Yes	Negative	
Self-collecting vaginal swab for HPV testing	Yes	Negative	
Self-collecting vaginal swab for HPV testing	Yes	Negative	
Self-collecting vaginal swab for HPV testing	Yes	Negative	
Self-collecting vaginal swab for HPV testing	Yes	Negative	
Self-collecting vaginal swab for HPV testing	Yes	Negative	
Self-collecting vaginal swab for HPV testing	Yes	Negative	
Self-collecting vaginal swab for HPV testing	Yes	Negative	
Self-collecting vaginal swab for HPV testing	Yes	Negative	
Self-collecting vaginal swab for HPV testing	Yes	Negative	
Self-collecting vaginal swab for HPV testing	Yes	Negative	
Self-collecting vaginal swab for HPV testing	Yes	Negative	
Self-collecting vaginal swab for HPV testing	Yes	Negative	
Self-collecting vaginal swab for HPV testing	Yes	Negative	

Self-collecting vaginal swab for HPV testing	Yes	Negative	
Self-collecting vaginal swab for HPV testing	Yes	Negative	
Self-collecting vaginal swab for HPV testing	Yes	Negative	
Self-collecting vaginal swab for HPV testing	Yes	Negative	
Self-collecting vaginal swab for HPV testing	Yes	Negative	
Self-collecting vaginal swab for HPV testing	Yes	Negative	
Self-collecting vaginal swab for HPV testing	Yes	Negative	
Self-collecting vaginal swab for HPV testing	Yes	Negative	
Self-collecting vaginal swab for HPV testing	Yes	Negative	
Self-collecting vaginal swab for HPV testing	Yes	Negative	
Self-collecting vaginal swab for HPV testing	Yes	Invalid	
No preference	Yes	Negative	
Healthcare professional-collected vaginal swab for HPV tes	Yes	Negative	
Self-collecting vaginal swab for HPV testing	Yes	Negative	
Self-collecting vaginal swab for HPV testing	Yes	Negative	
Self-collecting vaginal swab for HPV testing	Yes	Negative	
No preference	Yes	Negative	
Self-collecting vaginal swab for HPV testing	Yes	Negative	
Self-collecting vaginal swab for HPV testing	Yes	Negative	
Self-collecting vaginal swab for HPV testing	Yes	Negative	
No preference	Yes	Negative	
Self-collecting vaginal swab for HPV testing	Yes	Negative	
Self-collecting vaginal swab for HPV testing	Yes	Negative	
No preference	Yes	Negative	
Self-collecting vaginal swab for HPV testing	Yes	Negative	
Self-collecting vaginal swab for HPV testing	Yes	Negative	
Self-collecting vaginal swab for HPV testing	Yes	Negative	
Self-collecting vaginal swab for HPV testing	Yes	Positive Non-HPV 16/18	30/8/2023
Healthcare professional-collected vaginal swab for HPV tes	Yes	Negative	
Healthcare professional-collected vaginal swab for HPV tes	Yes	Negative	
Healthcare professional-collected vaginal swab for HPV tes	Yes	Negative	
Self-collecting vaginal swab for HPV testing	Yes	Positive Non-HPV 16/18	6/9/2023
Self-collecting vaginal swab for HPV testing	Yes	Negative	

Self-collecting vaginal swab for HPV testing	Yes	Negative	
		Negative	
Self-collecting vaginal swab for HPV testing	Yes	Negative	
Self-collecting vaginal swab for HPV testing	Yes	Negative	
Healthcare professional-collected vaginal swab for HPV tes	No	Negative	
Self-collecting vaginal swab for HPV testing	Yes	Invalid	
Self-collecting vaginal swab for HPV testing	Yes	Negative	
Self-collecting vaginal swab for HPV testing	Yes	Negative	
Self-collecting vaginal swab for HPV testing	Yes	Negative	
Self-collecting vaginal swab for HPV testing	Yes	Negative	
Self-collecting vaginal swab for HPV testing	Yes	Negative	
Pap smear - physician conducting a speculum examination	Yes	Positive Non-HPV 16/18	27/6/2023
Healthcare professional-collected vaginal swab for HPV tes	Yes	Negative	
Self-collecting vaginal swab for HPV testing	Yes	Negative	
Self-collecting vaginal swab for HPV testing	Yes	Negative	
Self-collecting vaginal swab for HPV testing	Yes	Negative	
Self-collecting vaginal swab for HPV testing	Yes	Negative	
Self-collecting vaginal swab for HPV testing	Yes	Negative	
Self-collecting vaginal swab for HPV testing	Yes	Negative	
Self-collecting vaginal swab for HPV testing	Yes	Negative	
Self-collecting vaginal swab for HPV testing	Yes	Negative	
Self-collecting vaginal swab for HPV testing	Yes	Negative	
No preference	Yes	Negative	
Self-collecting vaginal swab for HPV testing	Yes	Negative	
No preference	Yes	Negative	
Self-collecting vaginal swab for HPV testing	Yes	Negative	
Self-collecting vaginal swab for HPV testing	Yes	Negative	
Self-collecting vaginal swab for HPV testing	Yes	Negative	
Self-collecting vaginal swab for HPV testing	Yes	Positive Non-HPV 16/18	
Self-collecting vaginal swab for HPV testing	Yes	Negative	
Self-collecting vaginal swab for HPV testing	Yes	Negative	
Healthcare professional-collected vaginal swab for HPV tes	Yes	Negative	
Self-collecting vaginal swab for HPV testing	Yes	Negative	

Self-collecting vaginal swab for HPV testing	Yes	Negative	
Self-collecting vaginal swab for HPV testing	Yes	Negative	
Self-collecting vaginal swab for HPV testing	Yes	Negative	
Self-collecting vaginal swab for HPV testing	Yes	Negative	
Self-collecting vaginal swab for HPV testing	Yes	Negative	
Self-collecting vaginal swab for HPV testing	Yes	Negative	
Self-collecting vaginal swab for HPV testing	Yes	Negative	
Self-collecting vaginal swab for HPV testing	Yes	Negative	
Self-collecting vaginal swab for HPV testing	Yes	Negative	
Self-collecting vaginal swab for HPV testing	Yes	Negative	
Self-collecting vaginal swab for HPV testing	Yes	Negative	
Self-collecting vaginal swab for HPV testing	Yes	Negative	
Self-collecting vaginal swab for HPV testing	Yes	Negative	
Self-collecting vaginal swab for HPV testing	Yes	Negative	
Self-collecting vaginal swab for HPV testing	Yes	Negative	
Self-collecting vaginal swab for HPV testing	Yes	Negative	
Self-collecting vaginal swab for HPV testing	Yes	Negative	
Self-collecting vaginal swab for HPV testing	Yes	Negative	
Self-collecting vaginal swab for HPV testing	Yes	Negative	
Self-collecting vaginal swab for HPV testing	Yes	Negative	
No preference	Yes	Negative	
Self-collecting vaginal swab for HPV testing	Yes	Negative	
Self-collecting vaginal swab for HPV testing	Yes	Negative	
Healthcare professional-collected vaginal swab for HPV tes	Yes	Negative	
Self-collecting vaginal swab for HPV testing	Yes	Negative	
Self-collecting vaginal swab for HPV testing	Yes	Negative	
Self-collecting vaginal swab for HPV testing	Yes	Negative	
Self-collecting vaginal swab for HPV testing	Yes	Negative	
Self-collecting vaginal swab for HPV testing	Yes	Negative	
Self-collecting vaginal swab for HPV testing	Yes	Negative	
Healthcare professional-collected vaginal swab for HPV tes	Yes	Negative	
Self-collecting vaginal swab for HPV testing	Yes	Positive Non-HPV 16/18	11/10/2023
Self-collecting vaginal swab for HPV testing	Yes	Negative	

Self-collecting vaginal swab for HPV testing	Yes	Negative
Self-collecting vaginal swab for HPV testing	Yes	Negative
Self-collecting vaginal swab for HPV testing	Yes	Negative
Self-collecting vaginal swab for HPV testing	Yes	Negative
Self-collecting vaginal swab for HPV testing	Yes	Negative
Self-collecting vaginal swab for HPV testing	Yes	Negative
Self-collecting vaginal swab for HPV testing	Yes	Negative
Self-collecting vaginal swab for HPV testing	Yes	Invalid
Self-collecting vaginal swab for HPV testing	Yes	Negative
Self-collecting vaginal swab for HPV testing	Yes	Negative
Healthcare professional-collected vaginal swab for HPV tes	No	Negative
Self-collecting vaginal swab for HPV testing	Yes	Negative
Self-collecting vaginal swab for HPV testing	Yes	Negative
Healthcare professional-collected vaginal swab for HPV tes	Yes	Negative
Self-collecting vaginal swab for HPV testing	Yes	Negative
No preference	Yes	Negative
No preference	Yes	Negative
Self-collecting vaginal swab for HPV testing	Yes	Negative
Self-collecting vaginal swab for HPV testing	Yes	Negative
Self-collecting vaginal swab for HPV testing	Yes	Negative
Self-collecting vaginal swab for HPV testing	Yes	Negative
Self-collecting vaginal swab for HPV testing	Yes	Negative
Self-collecting vaginal swab for HPV testing	Yes	Negative
Self-collecting vaginal swab for HPV testing	Yes	Negative
Self-collecting vaginal swab for HPV testing	Yes	Negative
Self-collecting vaginal swab for HPV testing	Yes	Negative
Self-collecting vaginal swab for HPV testing	Yes	Negative
Self-collecting vaginal swab for HPV testing	Yes	Negative
Self-collecting vaginal swab for HPV testing	Yes	Negative
Self-collecting vaginal swab for HPV testing	Yes	Negative
Self-collecting vaginal swab for HPV testing	Yes	Positive Non-HPV 16/18
Self-collecting vaginal swab for HPV testing	Yes	Negative
Self-collecting vaginal swab for HPV testing	Yes	Negative

Self-collecting vaginal swab for HPV testing	Yes	Negative
Self-collecting vaginal swab for HPV testing	Yes	Negative
Self-collecting vaginal swab for HPV testing	Yes	Negative
Self-collecting vaginal swab for HPV testing	Yes	Negative
Healthcare professional-collected vaginal swab for HPV tes	Yes	Negative
Self-collecting vaginal swab for HPV testing	Yes	Negative
Self-collecting vaginal swab for HPV testing	Yes	Negative
Self-collecting vaginal swab for HPV testing	Yes	Negative
Self-collecting vaginal swab for HPV testing	Yes	Negative
Healthcare professional-collected vaginal swab for HPV tes	Yes	Negative
Self-collecting vaginal swab for HPV testing	Yes	Negative
Self-collecting vaginal swab for HPV testing	Yes	Negative
Self-collecting vaginal swab for HPV testing	Yes	Negative
Self-collecting vaginal swab for HPV testing	Yes	Negative
Self-collecting vaginal swab for HPV testing	Yes	Negative
Self-collecting vaginal swab for HPV testing	Yes	Negative
Pap smear - physician conducting a speculum examination	Yes	Negative
Self-collecting vaginal swab for HPV testing	Yes	Negative
Self-collecting vaginal swab for HPV testing	Yes	Negative
Self-collecting vaginal swab for HPV testing	Yes	Negative
Self-collecting vaginal swab for HPV testing	Yes	Negative
Self-collecting vaginal swab for HPV testing	Yes	Negative
Self-collecting vaginal swab for HPV testing	Yes	Negative
Self-collecting vaginal swab for HPV testing	Yes	Negative
Self-collecting vaginal swab for HPV testing	Yes	Negative
Healthcare professional-collected vaginal swab for HPV tes	Yes	Negative
Self-collecting vaginal swab for HPV testing	Yes	Negative
Self-collecting vaginal swab for HPV testing	Yes	Negative
Self-collecting vaginal swab for HPV testing	Yes	Negative
Self-collecting vaginal swab for HPV testing	Yes	Negative
Self-collecting vaginal swab for HPV testing	Yes	Negative
Self-collecting vaginal swab for HPV testing	Yes	Negative
Self-collecting vaginal swab for HPV testing	Yes	Negative

Self-collecting vaginal swab for HPV testing	Yes	Negative	
Self-collecting vaginal swab for HPV testing	Yes	Negative	
Self-collecting vaginal swab for HPV testing	Yes	Negative	
Self-collecting vaginal swab for HPV testing	Yes	Negative	
Self-collecting vaginal swab for HPV testing	Yes	Negative	
Self-collecting vaginal swab for HPV testing	Yes	Negative	
Self-collecting vaginal swab for HPV testing	Yes	Negative	
Self-collecting vaginal swab for HPV testing	Yes	Negative	
Self-collecting vaginal swab for HPV testing	Yes	Negative	
Self-collecting vaginal swab for HPV testing	Yes	Negative	
Self-collecting vaginal swab for HPV testing	Yes	Negative	
Self-collecting vaginal swab for HPV testing	Yes	Negative	
Self-collecting vaginal swab for HPV testing	Yes	Negative	
Self-collecting vaginal swab for HPV testing	Yes	Negative	
Self-collecting vaginal swab for HPV testing	Yes	Negative	
Self-collecting vaginal swab for HPV testing	Yes	Positive Non-HPV 16/18	
Self-collecting vaginal swab for HPV testing	Yes	Negative	
Self-collecting vaginal swab for HPV testing	Yes	Negative	
Self-collecting vaginal swab for HPV testing	Yes	Negative	
Self-collecting vaginal swab for HPV testing	Yes	Negative	
Self-collecting vaginal swab for HPV testing	Yes	Negative	
Self-collecting vaginal swab for HPV testing	Yes	Positive Non-HPV 16/18	3/10/2023
Self-collecting vaginal swab for HPV testing	Yes	Negative	
Self-collecting vaginal swab for HPV testing	Yes	Negative	
Healthcare professional-collected vaginal swab for HPV tes	Yes	Negative	
Self-collecting vaginal swab for HPV testing	Yes	Negative	
Self-collecting vaginal swab for HPV testing	Yes	Negative	
Self-collecting vaginal swab for HPV testing	Yes	Negative	
Self-collecting vaginal swab for HPV testing	Yes	Negative	
No preference	Yes	Negative	
Self-collecting vaginal swab for HPV testing	Yes	Invalid	
Self-collecting vaginal swab for HPV testing	Yes	Negative	
Self-collecting vaginal swab for HPV testing	Yes	Negative	

Self-collecting vaginal swab for HPV testing	Yes	Negative	
Self-collecting vaginal swab for HPV testing	Yes	Invalid	
Self-collecting vaginal swab for HPV testing	Yes	Negative	
Healthcare professional-collected vaginal swab for HPV tes	Yes	Negative	
Self-collecting vaginal swab for HPV testing	Yes	Negative	
Self-collecting vaginal swab for HPV testing	Yes	Negative	
Self-collecting vaginal swab for HPV testing	Yes	Negative	
Self-collecting vaginal swab for HPV testing	Yes	Negative	
No preference	Yes	Invalid	
Self-collecting vaginal swab for HPV testing	Yes	Negative	
Self-collecting vaginal swab for HPV testing	Yes	Negative	
Self-collecting vaginal swab for HPV testing	Yes	Negative	
Self-collecting vaginal swab for HPV testing	Yes	Negative	
Self-collecting vaginal swab for HPV testing	Yes	Negative	
Self-collecting vaginal swab for HPV testing	Yes	Negative	
Self-collecting vaginal swab for HPV testing	Yes	Negative	
Self-collecting vaginal swab for HPV testing	Yes	Negative	
Self-collecting vaginal swab for HPV testing	Yes	Negative	
Healthcare professional-collected vaginal swab for HPV tes	Yes	Negative	
Self-collecting vaginal swab for HPV testing	Yes	Positive HPV 18	
Healthcare professional-collected vaginal swab for HPV tes	Yes	Negative	
Self-collecting vaginal swab for HPV testing	Yes	Negative	
Self-collecting vaginal swab for HPV testing	Yes	Negative	
Self-collecting vaginal swab for HPV testing	Yes	Negative	
Self-collecting vaginal swab for HPV testing	Yes	Negative	
Self-collecting vaginal swab for HPV testing	Yes	Negative	
Self-collecting vaginal swab for HPV testing	Yes	Positive Non-HPV 16/18	20/2/2024
Self-collecting vaginal swab for HPV testing	Yes	Negative	
Self-collecting vaginal swab for HPV testing	Yes	Negative	
Self-collecting vaginal swab for HPV testing	Yes	Negative	
Self-collecting vaginal swab for HPV testing	Yes	Invalid	
Self-collecting vaginal swab for HPV testing	Yes	Invalid	
Self-collecting vaginal swab for HPV testing	Yes	Negative	

No preference	Yes	Negative	
Self-collecting vaginal swab for HPV testing	Yes	Negative	
Healthcare professional-collected vaginal swab for HPV tes	Yes	Negative	
Self-collecting vaginal swab for HPV testing	Yes	Negative	
Self-collecting vaginal swab for HPV testing	Yes	Negative	
Self-collecting vaginal swab for HPV testing	Yes	Negative	
Healthcare professional-collected vaginal swab for HPV tes	Yes	Negative	
Self-collecting vaginal swab for HPV testing	Yes	Negative	
Self-collecting vaginal swab for HPV testing	Yes	Positive Non-HPV 16/18	
Self-collecting vaginal swab for HPV testing	Yes	Negative	
Self-collecting vaginal swab for HPV testing	Yes	Negative	
Self-collecting vaginal swab for HPV testing	Yes	Negative	
Self-collecting vaginal swab for HPV testing	Yes	Negative	
Self-collecting vaginal swab for HPV testing	Yes	Negative	
Self-collecting vaginal swab for HPV testing	Yes	Negative	
Self-collecting vaginal swab for HPV testing	Yes	Negative	
Self-collecting vaginal swab for HPV testing	Yes	Invalid	
No preference	Yes	Negative	
Self-collecting vaginal swab for HPV testing	Yes	Negative	
Self-collecting vaginal swab for HPV testing	Yes	Negative	
Self-collecting vaginal swab for HPV testing	Yes	Negative	
Self-collecting vaginal swab for HPV testing	Yes	Positive Non-HPV 16/18	13/9/2023
Self-collecting vaginal swab for HPV testing	Yes	Negative	
Self-collecting vaginal swab for HPV testing	Yes	Negative	
Self-collecting vaginal swab for HPV testing	Yes	Negative	
Self-collecting vaginal swab for HPV testing	Yes	Negative	
Self-collecting vaginal swab for HPV testing	Yes	Negative	
Healthcare professional-collected vaginal swab for HPV tes	Yes	Negative	
Self-collecting vaginal swab for HPV testing	Yes	Negative	
Self-collecting vaginal swab for HPV testing	Yes	Negative	
Self-collecting vaginal swab for HPV testing	Yes	Negative	
Self-collecting vaginal swab for HPV testing	Yes	Negative	
Pap smear - physician conducting a speculum examination	Yes	Negative	

Self-collecting vaginal swab for HPV testing	Yes	Negative	
Self-collecting vaginal swab for HPV testing	Yes	Negative	
Self-collecting vaginal swab for HPV testing	Yes	Negative	
Healthcare professional-collected vaginal swab for HPV tes	Yes	Negative	
Self-collecting vaginal swab for HPV testing	Yes	Negative	
		Negative	
Self-collecting vaginal swab for HPV testing	Yes	Negative	
Self-collecting vaginal swab for HPV testing	Yes	Negative	
Self-collecting vaginal swab for HPV testing	Yes	Invalid	
Self-collecting vaginal swab for HPV testing	Yes	Negative	
Self-collecting vaginal swab for HPV testing	Yes	Negative	
Self-collecting vaginal swab for HPV testing	Yes	Positive Non-HPV 16/18	11/10/2023
Self-collecting vaginal swab for HPV testing	Yes	Positive Non-HPV 16/18	
Self-collecting vaginal swab for HPV testing	Yes	Negative	
Self-collecting vaginal swab for HPV testing	Yes	Negative	
Self-collecting vaginal swab for HPV testing	Yes	Negative	
Self-collecting vaginal swab for HPV testing	Yes	Negative	
Self-collecting vaginal swab for HPV testing	Yes	Negative	
Healthcare professional-collected vaginal swab for HPV tes	Yes	Negative	
Self-collecting vaginal swab for HPV testing	Yes	Negative	
Self-collecting vaginal swab for HPV testing	Yes	Negative	
Self-collecting vaginal swab for HPV testing	Yes	Negative	
		Negative	
Self-collecting vaginal swab for HPV testing	Yes	Invalid	
Self-collecting vaginal swab for HPV testing	Yes	Negative	
Self-collecting vaginal swab for HPV testing	Yes	Invalid	
Self-collecting vaginal swab for HPV testing	Yes	Negative	
Self-collecting vaginal swab for HPV testing	Yes	Negative	
Healthcare professional-collected vaginal swab for HPV tes	Yes	Negative	
Self-collecting vaginal swab for HPV testing	Yes	Negative	
Self-collecting vaginal swab for HPV testing	Yes	Negative	
Self-collecting vaginal swab for HPV testing	Yes	Negative	
Self-collecting vaginal swab for HPV testing	Yes	Negative	

Self-collecting vaginal swab for HPV testing	Yes	Negative	
Self-collecting vaginal swab for HPV testing	Yes	Negative	
Healthcare professional-collected vaginal swab for HPV tes	No	Invalid	
Self-collecting vaginal swab for HPV testing	Yes	Negative	
No preference	Yes	Negative	
Self-collecting vaginal swab for HPV testing	Yes	Negative	
Self-collecting vaginal swab for HPV testing	Yes	Negative	
Self-collecting vaginal swab for HPV testing	Yes	Positive Non-HPV 16/18	
Self-collecting vaginal swab for HPV testing	Yes	Negative	
Self-collecting vaginal swab for HPV testing	Yes	Negative	
Self-collecting vaginal swab for HPV testing	Yes	Negative	
Self-collecting vaginal swab for HPV testing	Yes	Negative	
Self-collecting vaginal swab for HPV testing	Yes	Negative	
Self-collecting vaginal swab for HPV testing	Yes	Negative	
Healthcare professional-collected vaginal swab for HPV tes	Yes	Negative	
Self-collecting vaginal swab for HPV testing	Yes	Negative	
Self-collecting vaginal swab for HPV testing	Yes	Invalid	
		Negative	
Self-collecting vaginal swab for HPV testing	Yes	Negative	
Pap smear - physician conducting a speculum examination	Yes	Negative	
Self-collecting vaginal swab for HPV testing	Yes	Invalid	
Self-collecting vaginal swab for HPV testing	Yes	Negative	
Self-collecting vaginal swab for HPV testing	Yes	Negative	
Self-collecting vaginal swab for HPV testing	Yes	Negative	
Self-collecting vaginal swab for HPV testing	Yes	Negative	
Self-collecting vaginal swab for HPV testing	Yes	Positive HPV 16	8/12/2023
Healthcare professional-collected vaginal swab for HPV tes	No	No sample	
No preference	Yes	Positive Non-HPV 16/18	20/2/2024
Self-collecting vaginal swab for HPV testing	Yes	Negative	
Self-collecting vaginal swab for HPV testing	Yes	Negative	
Healthcare professional-collected vaginal swab for HPV tes	No	Invalid	
Self-collecting vaginal swab for HPV testing	Yes	Negative	
Self-collecting vaginal swab for HPV testing	Yes	Negative	

Self-collecting vaginal swab for HPV testing	Yes	Negative	
Self-collecting vaginal swab for HPV testing	Yes	Negative	
No preference	Yes	Negative	
Self-collecting vaginal swab for HPV testing	Yes	Invalid	
Self-collecting vaginal swab for HPV testing	Yes	Negative	
Self-collecting vaginal swab for HPV testing	Yes	Negative	
Self-collecting vaginal swab for HPV testing	Yes	Negative	
Self-collecting vaginal swab for HPV testing	Yes	Negative	
Healthcare professional-collected vaginal swab for HPV tes	No	Negative	
Self-collecting vaginal swab for HPV testing	Yes	Negative	
Self-collecting vaginal swab for HPV testing	Yes	Invalid	
Self-collecting vaginal swab for HPV testing	Yes	Invalid	
Self-collecting vaginal swab for HPV testing	Yes	Negative	
Self-collecting vaginal swab for HPV testing	Yes	Negative	
Self-collecting vaginal swab for HPV testing	Yes	Negative	
Healthcare professional-collected vaginal swab for HPV tes	Yes	Negative	
Self-collecting vaginal swab for HPV testing	Yes	Negative	
Self-collecting vaginal swab for HPV testing	Yes	Positive Non-HPV 16/18	
Self-collecting vaginal swab for HPV testing	Yes	Positive HPV 16 & Non-HPV 16/18	10/1/2024
Self-collecting vaginal swab for HPV testing	Yes	Negative	
Self-collecting vaginal swab for HPV testing	Yes	Negative	
Self-collecting vaginal swab for HPV testing	Yes	Positive Non-HPV 16/18	
Self-collecting vaginal swab for HPV testing	Yes	Negative	
Self-collecting vaginal swab for HPV testing	Yes	Negative	
Self-collecting vaginal swab for HPV testing	Yes	Negative	
Healthcare professional-collected vaginal swab for HPV tes	No	Negative	
Self-collecting vaginal swab for HPV testing	Yes	Negative	
Self-collecting vaginal swab for HPV testing	Yes	Negative	
Self-collecting vaginal swab for HPV testing	Yes	Negative	
Self-collecting vaginal swab for HPV testing	Yes	Negative	
Self-collecting vaginal swab for HPV testing	Yes	Negative	
Self-collecting vaginal swab for HPV testing	Yes	Negative	
Self-collecting vaginal swab for HPV testing	Yes	Negative	

Self-collecting vaginal swab for HPV testing	Yes	Negative	
Self-collecting vaginal swab for HPV testing	Yes	Negative	
Self-collecting vaginal swab for HPV testing	Yes	Negative	
Healthcare professional-collected vaginal swab for HPV tes	Yes	Negative	
Self-collecting vaginal swab for HPV testing	Yes	Negative	
Self-collecting vaginal swab for HPV testing	Yes	Negative	
Self-collecting vaginal swab for HPV testing	Yes	Negative	
Self-collecting vaginal swab for HPV testing	Yes	Negative	
Self-collecting vaginal swab for HPV testing	Yes	Negative	
Self-collecting vaginal swab for HPV testing	Yes	Negative	
Self-collecting vaginal swab for HPV testing	Yes	Negative	
Self-collecting vaginal swab for HPV testing	Yes	Negative	
Self-collecting vaginal swab for HPV testing	Yes	Negative	
Self-collecting vaginal swab for HPV testing	Yes	Negative	
Self-collecting vaginal swab for HPV testing	Yes	Negative	
Self-collecting vaginal swab for HPV testing	Yes	Negative	
Self-collecting vaginal swab for HPV testing	Yes	Negative	
Self-collecting vaginal swab for HPV testing	Yes	Negative	
Self-collecting vaginal swab for HPV testing	Yes	Negative	
Healthcare professional-collected vaginal swab for HPV tes	Yes	Negative	
Self-collecting vaginal swab for HPV testing	Yes	Negative	
Self-collecting vaginal swab for HPV testing	Yes	Negative	
Self-collecting vaginal swab for HPV testing	Yes	Negative	
No preference	Yes	Negative	
Self-collecting vaginal swab for HPV testing	Yes	Positive Non-HPV 16/18	
Self-collecting vaginal swab for HPV testing	Yes	Negative	
Self-collecting vaginal swab for HPV testing	Yes	Positive Non-HPV 16/18	
Self-collecting vaginal swab for HPV testing	Yes	Negative	
Self-collecting vaginal swab for HPV testing	Yes	Negative	
Self-collecting vaginal swab for HPV testing	Yes	Negative	
No preference	Yes	Positive Non-HPV 16/18	12/12/2023
Self-collecting vaginal swab for HPV testing	Yes	Negative	
Self-collecting vaginal swab for HPV testing	Yes	Negative	

Self-collecting vaginal swab for HPV testing	Yes	Invalid	
Self-collecting vaginal swab for HPV testing	Yes	Negative	
Self-collecting vaginal swab for HPV testing	Yes	Negative	
Self-collecting vaginal swab for HPV testing	Yes	Negative	
Self-collecting vaginal swab for HPV testing	Yes	Negative	
Self-collecting vaginal swab for HPV testing	Yes	Negative	
Self-collecting vaginal swab for HPV testing	Yes	Negative	
No preference	Yes	Negative	
		Negative	
Self-collecting vaginal swab for HPV testing	Yes	Negative	
Healthcare professional-collected vaginal swab for HPV tes	Yes	Negative	
Self-collecting vaginal swab for HPV testing	Yes	Negative	
Self-collecting vaginal swab for HPV testing	Yes	Negative	
Self-collecting vaginal swab for HPV testing	Yes	Negative	
Self-collecting vaginal swab for HPV testing	Yes	Positive Non-HPV 16/18	31/1/2024
Self-collecting vaginal swab for HPV testing	Yes	Negative	
Self-collecting vaginal swab for HPV testing	Yes	Negative	
Healthcare professional-collected vaginal swab for HPV tes	No	Negative	
Self-collecting vaginal swab for HPV testing	Yes	Negative	
Self-collecting vaginal swab for HPV testing	Yes	Negative	
Self-collecting vaginal swab for HPV testing	Yes	Positive HPV 16	9/1/2024
Self-collecting vaginal swab for HPV testing	Yes	Negative	
Self-collecting vaginal swab for HPV testing	Yes	Negative	
Self-collecting vaginal swab for HPV testing	Yes	Negative	
Self-collecting vaginal swab for HPV testing	Yes	Negative	
Self-collecting vaginal swab for HPV testing	Yes	Negative	
Self-collecting vaginal swab for HPV testing	Yes	Negative	
Self-collecting vaginal swab for HPV testing	Yes	Invalid	
Self-collecting vaginal swab for HPV testing	Yes	Positive Non-HPV 16/18	
Self-collecting vaginal swab for HPV testing	Yes	Invalid	
		Positive Non-HPV 16/18	
Healthcare professional-collected vaginal swab for HPV tes	Yes	Negative	
Self-collecting vaginal swab for HPV testing	Yes	Negative	

Self-collecting vaginal swab for HPV testing	Yes	Negative	
Self-collecting vaginal swab for HPV testing	Yes	Negative	
Self-collecting vaginal swab for HPV testing	Yes	Negative	
Self-collecting vaginal swab for HPV testing	Yes	Negative	
Self-collecting vaginal swab for HPV testing	Yes	Negative	
Self-collecting vaginal swab for HPV testing	Yes	Negative	
Self-collecting vaginal swab for HPV testing	Yes	Negative	
Self-collecting vaginal swab for HPV testing	Yes	Negative	
Self-collecting vaginal swab for HPV testing	Yes	Invalid	
Self-collecting vaginal swab for HPV testing	Yes	Negative	
Self-collecting vaginal swab for HPV testing	Yes	Positive HPV 16	29/5/2024
Self-collecting vaginal swab for HPV testing	Yes	Negative	
Self-collecting vaginal swab for HPV testing	Yes	Negative	
Self-collecting vaginal swab for HPV testing	Yes	Negative	
Healthcare professional-collected vaginal swab for HPV tes	Yes	Negative	
Self-collecting vaginal swab for HPV testing	Yes	Negative	
Self-collecting vaginal swab for HPV testing	Yes	Negative	
Self-collecting vaginal swab for HPV testing	Yes	Negative	
Self-collecting vaginal swab for HPV testing	Yes	Negative	
Self-collecting vaginal swab for HPV testing	Yes	Negative	
Self-collecting vaginal swab for HPV testing	Yes	Negative	
Self-collecting vaginal swab for HPV testing	Yes	Negative	
Self-collecting vaginal swab for HPV testing	Yes	Negative	
Self-collecting vaginal swab for HPV testing	Yes	Negative	
Self-collecting vaginal swab for HPV testing	Yes	Negative	
		Invalid	
Self-collecting vaginal swab for HPV testing	Yes	Negative	
Self-collecting vaginal swab for HPV testing	Yes	Positive Non-HPV 16/18	
Self-collecting vaginal swab for HPV testing	Yes	Negative	
Self-collecting vaginal swab for HPV testing	Yes	Negative	
Self-collecting vaginal swab for HPV testing	Yes	Negative	
Self-collecting vaginal swab for HPV testing	Yes	Invalid	
Self-collecting vaginal swab for HPV testing	Yes	Negative	

Healthcare professional-collected vaginal swab for HPV tes	Yes	Negative	
Self-collecting vaginal swab for HPV testing	Yes	Negative	
Self-collecting vaginal swab for HPV testing	Yes	Negative	
Self-collecting vaginal swab for HPV testing	Yes	Invalid	
Self-collecting vaginal swab for HPV testing	Yes	Negative	
Self-collecting vaginal swab for HPV testing	Yes	Negative	
Self-collecting vaginal swab for HPV testing	Yes	Invalid	
Self-collecting vaginal swab for HPV testing	Yes	Negative	
Self-collecting vaginal swab for HPV testing	Yes	Positive Non-HPV 16/18	29/5/2024
Self-collecting vaginal swab for HPV testing	Yes	Negative	
Self-collecting vaginal swab for HPV testing	Yes	Negative	
Self-collecting vaginal swab for HPV testing	Yes	Negative	
Self-collecting vaginal swab for HPV testing	Yes	Negative	
Self-collecting vaginal swab for HPV testing	Yes	Invalid	
Self-collecting vaginal swab for HPV testing	Yes	Negative	
Self-collecting vaginal swab for HPV testing	Yes	Negative	
Self-collecting vaginal swab for HPV testing	Yes	Negative	
Self-collecting vaginal swab for HPV testing	Yes	Negative	
Self-collecting vaginal swab for HPV testing	Yes	Negative	
Self-collecting vaginal swab for HPV testing	Yes	Negative	

Did participant come for her colposcopy appointment?	Result of repeat self-sampling HPV test	HPV positive type
Yes	Positive	Non-HPV 16/18
Yes	Negative	
Yes	Negative	
No		

Yes	Positive	Non-HPV 16/18
Yes	Negative	
Yes	Positive	Non-HPV 16/18
No		
No		

Yes	Positive	Non-HPV 16/18
Yes	Positive	Non-HPV 16/18

No		
Yes	Positive	HPV 16
Yes	Positive	HPV 16 & Non-HPV 16/18
Yes	Negative	
Yes	Negative	
Yes	Positive	HPV 16 & Non-HPV 16/18

No

Yes Negative

Yes	Negative
Yes	Negative
No	

No	
Yes	Negative
Yes	Negative

Yes	Positive	Non-HPV 16/18
Yes	Negative	
No		

No		
No		
Yes	Positive	HPV 16

Yes	Negative	
Yes	Negative	
Yes	Positive	Non-HPV 16/18

Yes	Positive	Non-HPV 16/18
No		
Yes	Negative	

Yes	Negative
No	

Yes	Positive	Non-HPV 16/18

Yes	Negative	
Yes	Positive	Non-HPV 16/18
Yes	Positive	Non-HPV 16/18

Yes	Negative
Yes	Negative

No		
Yes	Positive	Non-HPV 16/18

Yes	Negative

No		
No		
No		
Yes	Positive	Non-HPV 16/18

Yes	Positive	Non-HPV 16/18
No		

No

Yes	Positive	Non-HPV 16/18
No		
Yes	Negative	

No

Yes

Negative

Yes	Negative

Yes	Positive	Non-HPV 16/18
No		

Yes	Invalid
Yes	Negative

Yes	Positive	HPV 18

Yes	Positive	Non-HPV 16/18

Yes	Positive	HPV 16
Yes	Positive	Non-HPV 16/18
Yes	Positive	Non-HPV 16/18
Yes	Positive	Non-HPV 16/18

Yes	Positive	Non-HPV 16/18
Yes	Positive	Non-HPV 16/18

Yes

Negative

No

Yes	Positive	Non-HPV 16/18

No

No		
Yes	Positive	Non-HPV 16/18

No		
Yes	Positive	Non-HPV 16/18

No

Yes

Negative

Yes	Positive	Non-HPV 16/18
No		

No		
Yes	Positive	HPV 16
Yes	Negative	

No		
Yes	Positive	HPV 16
No		

No		
No		
Yes	Positive	Non-HPV 16/18

Yes	Positive	Non-HPV 16/18
Yes	Positive	HPV 16
No		
No		

Yes	Negative
No	

Yes
